# Methods to increase response to postal and electronic questionnaires

**DOI:** 10.1002/14651858.MR000008.pub5

**Published:** 2023-11-30

**Authors:** Philip James Edwards, Ian Roberts, Mike J Clarke, Carolyn DiGuiseppi, Benjamin Woolf, Chloe Perkins

**Affiliations:** Faculty of Epidemiology and Population HealthLondon School of Hygiene & Tropical MedicineLondonUK; Centre for Public HealthQueens University BelfastBelfastUK; Colorado School of Public HealthUniversity of Colorado Anschutz Medical CampusAuroraCOUSA; School of Psychological ScienceUniversity of BristolBristolUK; LondonUK

## Abstract

**Background:**

Self‐administered questionnaires are widely used to collect data in epidemiological research, but non‐response reduces the effective sample size and can introduce bias. Finding ways to increase response to postal and electronic questionnaires would improve the quality of epidemiological research.

**Objectives:**

To identify effective strategies to increase response to postal and electronic questionnaires.

**Search methods:**

We searched 14 electronic databases up to December 2021 and manually searched the reference lists of relevant trials and reviews. We contacted the authors of all trials or reviews to ask about unpublished trials; where necessary, we also contacted authors to confirm the methods of allocation used and to clarify results presented.

**Selection criteria:**

Randomised trials of methods to increase response to postal or electronic questionnaires. We assessed the eligibility of each trial using pre‐defined criteria.

**Data collection and analysis:**

We extracted data on the trial participants, the intervention, the number randomised to intervention and comparison groups and allocation concealment. For each strategy, we estimated pooled odds ratios (OR) and 95% confidence intervals (CI) in a random‐effects model. We assessed evidence for selection bias using Egger's weighted regression method and Begg's rank correlation test and funnel plot. We assessed heterogeneity amongst trial odds ratios using a Chi^2^ test and quantified the degree of inconsistency between trial results using the I^2^ statistic.

**Main results:**

**Postal**

We found 670 eligible trials that evaluated over 100 different strategies of increasing response to postal questionnaires. We found substantial heterogeneity amongst trial results in half of the strategies.

The odds of response almost doubled when: using monetary incentives (odds ratio (OR) 1.86; 95% confidence interval (CI) 1.73 to 1.99; heterogeneity I^2^ = 85%); using a telephone reminder (OR 1.96; 95% CI 1.03 to 3.74); and when clinical outcome questions were placed last (OR 2.05; 95% CI 1.00 to 4.24).

The odds of response increased by about half when: using a shorter questionnaire (OR 1.58; 95% CI 1.40 to 1.78); contacting participants before sending questionnaires (OR 1.36; 95% CI 1.23 to 1.51; I^2^ = 87%); incentives were given with questionnaires (i.e. unconditional) rather than when given only after participants had returned their questionnaire (i.e. conditional on response) (OR 1.53; 95% CI 1.35 to 1.74); using personalised SMS reminders (OR 1.53; 95% CI 0.97 to 2.42); using a special (recorded) delivery service (OR 1.68; 95% CI 1.36 to 2.08; I^2^ = 87%); using electronic reminders (OR 1.60; 95% CI 1.10 to 2.33); using intensive follow‐up (OR 1.69; 95% CI 0.93 to 3.06); using a more interesting/salient questionnaire (OR 1.73; 95% CI 1.12 to 2.66); and when mentioning an obligation to respond (OR 1.61; 95% CI 1.16 to 2.22). The odds of response also increased with: non‐monetary incentives (OR 1.16; 95% CI 1.11 to 1.21; I^2^ = 80%); a larger monetary incentive (OR 1.24; 95% CI 1.15 to 1.33); a larger non‐monetary incentive (OR 1.15; 95% CI 1.00 to 1.33); when a pen was included (OR 1.44; 95% CI 1.38 to 1.50); using personalised materials (OR 1.15; 95% CI 1.09 to 1.21; I^2^ = 57%); using a single‐sided rather than a double‐sided questionnaire (OR 1.13; 95% CI 1.02 to 1.25); using stamped return envelopes rather than franked return envelopes (OR 1.23; 95% CI 1.13 to 1.33; I^2^ = 69%), assuring confidentiality (OR 1.33; 95% CI 1.24 to 1.42); using first‐class outward mailing (OR 1.11; 95% CI 1.02 to 1.21); and when questionnaires originated from a university (OR 1.32; 95% CI 1.13 to 1.54).

The odds of response were reduced when the questionnaire included questions of a sensitive nature (OR 0.94; 95% CI 0.88 to 1.00).

**Electronic**

We found 88 eligible trials that evaluated over 30 different ways of increasing response to electronic questionnaires. We found substantial heterogeneity amongst trial results in half of the strategies. The odds of response tripled when: using a brief letter rather than a detailed letter (OR 3.26; 95% CI 1.79 to 5.94); and when a picture was included in an email (OR 3.05; 95% CI 1.84 to 5.06; I^2^ = 19%).

The odds of response almost doubled when: using monetary incentives (OR 1.88; 95% CI 1.31 to 2.71; I^2^ = 79%); and using a more interesting topic (OR 1.85; 95% CI 1.52 to 2.26). The odds of response increased by half when: using non‐monetary incentives (OR 1.60; 95% CI 1.25 to 2.05); using shorter e‐questionnaires (OR 1.51; 95% CI 1.06 to 2.16; I^2^ = 94%); and using a more interesting e‐questionnaire (OR 1.85; 95% CI 1.52 to 2.26). The odds of response increased by a third when: offering survey results as an incentive (OR 1.36; 95% CI 1.16 to 1.59); using a white background (OR 1.31; 95% CI 1.10 to 1.56); and when stressing the benefits to society of response (OR 1.38; 95% CI 1.07 to 1.78; I^2^ = 41%).

The odds of response also increased with: personalised e‐questionnaires (OR 1.24; 95% CI 1.17 to 1.32; I^2^ = 41%); using a simple header (OR 1.23; 95% CI 1.03 to 1.48); giving a deadline (OR 1.18; 95% CI 1.03 to 1.34); and by giving a longer time estimate for completion (OR 1.25; 95% CI 0.96 to 1.64).

The odds of response were reduced when: "Survey" was mentioned in the e‐mail subject (OR 0.81; 95% CI 0.67 to 0.97); when the email or the e‐questionnaire was from a male investigator, or it included a male signature (OR 0.55; 95% CI 0.38 to 0.80); and by using university sponsorship (OR 0.84; 95%CI 0.69 to 1.01).

The odds of response using a postal questionnaire were over twice those using an e‐questionnaire (OR 2.33; 95% CI 2.25 to 2.42; I^2^ = 98%). Response also increased when: providing a choice of response mode (electronic or postal) rather than electronic only (OR 1.76 95% CI 1.67 to 1.85; I^2^ = 97%); and when administering the e‐questionnaire by computer rather than by smartphone (OR 1.62 95% CI 1.36 to 1.94).

**Authors' conclusions:**

Researchers using postal and electronic questionnaires can increase response using the strategies shown to be effective in this Cochrane review.

## Plain language summary

**How can response to postal or web questionnaires be increased?**

**Key messages**

Response to questionnaires can be increased by contacting people before they are sent a questionnaire;

Response to questionnaires can be increased by making questionnaires, letters, and emails more personal, and preferably kept short;

Response to questionnaires can be increased by giving an incentive, for example, a small amount of money, or a non‐monetary incentive such as a pen.

**Why is response to questionnaires important?**

Postal and electronic questionnaires are a relatively inexpensive way to collect information from people for research purposes. If people do not reply (so called ’non‐responders’), the research results will tend to be less accurate.

**What did we want to find out?**

We wanted to find effective ways to increase response to postal and electronic questionnaires.

**What did we do?**  We searched for studies that examined any way of increasing questionnaire response.

We summarised the results of the studies.

**What did we find?**

A very large amount of research has been done to try to identify ways to increase response, and we have included 758 studies in this Cochrane methodology review update. The studies included a wide range of people asked to complete a questionnaire, from patients, doctors, university students, and professors, to marketing managers, accountants, and grocery store managers.

We found that response will be increased by contacting people before they are sent a questionnaire. We also found that response to postal questionnaires will be increased if they are sent by a university. Response can also be increased by giving an incentive, for example, a small amount of money, or a non‐monetary incentive such as a pen. Response may be higher using a postal questionnaire rather than an electronic one, or by providing a choice of response modes (electronic or postal). Response can be increased by making questionnaires, letters, and emails more personal, and preferably kept short.

**What are the limitations of the evidence?**

We had to exclude some studies because we could not confirm that they were free from bias.

**How up‐to‐date is this evidence?**

This review updates our previous review. The evidence is up‐to‐date to December 2021.

## Background

Self‐administered questionnaires are widely used to collect data in epidemiological research (Van Gelder 2010). When collecting information from large, geographically dispersed populations, a self‐administered questionnaire, delivered by post or electronically, is often the only financially viable option. Non‐response to questionnaires reduces the effective sample size and can introduce bias (Armstrong 1995). This review updates our previous version, which was published in 2009 based on searches performed in Feb 2008 (Edwards 2009).

### Description of the methods being investigated

A previous review (Yammarino 1991) suggested that repeated contacts (e.g. preliminary notification and follow‐up), appeals in letters, inclusion of a return envelope, types of postage, monetary incentives, and shorter questionnaires can increase response.

### How these methods might work

Some methods (e.g. a shorter questionnaire or inclusion of a return envelope) might reduce the burden faced by individuals when completing and returning a questionnaire, leading to an increase in response. Other methods (e.g. incentives) might induce a sense of reciprocity in individuals such that they will complete and return a questionnaire in return for benefits received (Molm 2010).

### Why it is important to do this review

The identification of effective strategies to increase response to postal and electronic questionnaires will help to maintain power and reduce the risk of bias in study results, thus improving the quality of epidemiological research.

## Objectives

To identify effective strategies to increase response to postal and electronic questionnaires.

## Methods

### Criteria for considering studies for this review

#### Types of studies

All unconfounded randomised trials of methods designed to increase response to postal or electronic questionnaires were eligible. A postal questionnaire was defined as a questionnaire that is delivered to a person’s home or work address by a distribution system. This includes questionnaires delivered by any postal service, including internal organisational mail and those hand‐delivered to a person’s address; It does not include questionnaires distributed at, for example, a shop or in a doctor’s office. An electronic questionnaire was defined as a questionnaire that is delivered electronically by email or by SMS and includes those administered online over the internet.

#### Types of data

Any population (e.g. patients or healthcare providers, and including any participants of non‐health studies) were eligible.

#### Types of methods

Any methods designed to increase response to postal or electronic questionnaires were eligible. Strategies requiring telephone contact as a follow‐up technique were included but those requiring home visits were not.

#### Types of outcome measures

##### Primary outcomes

- Proportion of completed, or partially completed postal questionnaires returned after all mailings.
- Proportion of participants completing or submitting the online questionnaire.

##### Secondary outcomes

- Proportion of completed, or partially completed questionnaires returned after the first mailing.
- Proportion of participants logging in or clicking the hyperlink to visit the online questionnaire.

### Search methods for identification of studies

#### Electronic searches

We identified trials by searching 14 electronic bibliographic databases. We ran these searches in December 2021 and have agreed with Cochrane Methodology that the search should not be updated further after this time. This is because the very large number of reports identified in the December 2021 search and the stability of our overall conclusions means that updating our searches would be of much less relevance than it would be for Cochrane Reviews focusing on clinical questions.

#### Searching other resources

We handsearched two journals (Public Opinion Quarterly, from 1960 to 1998; American Journal of Epidemiology, from 1948 to 1999). We also searched the reference lists of all identified trials, the reference lists of relevant meta‐analyses, and contacted the authors of the included trials. Full details of the search strategies used for all review versions are illustrated in Appendix 1.

### Data collection and analysis

#### Selection of studies

Two authors independently examined the titles, abstracts and keywords of all records identified from the electronic searches. We obtained full‐text articles (where available) of all selected abstracts and used an eligibility form to determine final study selection. We resolved any disagreements through discussion.

#### Data extraction and management

Two authors independently extracted data from eligible reports using a standard pro forma, with disagreements resolved by a third author. We extracted data on the type of intervention evaluated, the numbers randomised to intervention or control groups, the quality of allocation concealment, the types of participants, and the materials and follow‐up methods used. Two outcomes were used for each method of delivery to estimate the effect of each intervention on the questionnaire response. For postal, the proportion of questionnaires returned after the first mailing, and the proportion returned after all follow‐up contacts were measured. For electronic, the proportion of participants logging‐in or clicking the hyperlink to visit the online questionnaire, and the proportion of participants completing or submitting the online questionnaire were measured. We excluded trials in which we could not confirm that random allocation had been used to allocate participants. For this 2023 update, we used the online screening and data extraction tool in Covidence and then exported the data from Covidence into a spreadsheet for entry into Review Manager 5 (Review Manager 2020).

As per the original review and in all subsequent updates, we used the first author’s name with the publication year of the reference as the trial identifier. When more than one trial was reported in the paper, we identified these separately by adding letters a, b, c, etc. For example:

- Allen 2016 reported two independent experiments, which we have included separately as Allen 2016 and Allen 2016a.
- Roszkowski 1990 reported independent replications of the same trial in 14 different populations and we have reported them as Roszkowski 1990a; Roszkowski 1990b; Roszkowski 1990c; Roszkowski 1990d; Roszkowski 1990e; Roszkowski 1990f; Roszkowski 1990g; Roszkowski 1990h; Roszkowski 1990i; Roszkowski 1990j; Roszkowski 1990k; Roszkowski 1990l; Roszkowski 1990m; Roszkowski 1990n.
- Gibson 1999 randomised participants to different monetary incentives and reported the results separately for a sample of Medicaid participants (Gibson 1999a) and for a sample of Basic Health Plan participants (Gibson 1999b). All non‐respondents were randomised to a reminder sent using either Certified postal delivery or standard postal delivery (Gibson 1999c).

#### Assessment of risk of bias in included studies

In the original review version and in the prior update versions (Edwards 2003; Edwards 2007; Edwards 2009), we focused on the integrity of allocation concealment and two authors independently scored methodological quality on the scale used by Schulz (Schulz 1995) as shown below, assigning 'A' to best quality and 'C' to poorest quality:

- A ‐ trials deemed to have taken adequate measures to conceal allocation (i.e. central randomisation; computer‐generated address labels; or other description that contained elements that would ensure concealment);
- B ‐ trials in which the authors either did not report an allocation concealment approach at all or reported an approach that did not fall into one of the other categories;
- C‐ trials in which concealment was inadequate (such as alternation or reference to case record numbers or to dates of birth).

Where the methods used to conceal allocation were not clearly reported, the study authors were contacted, if possible, for clarification. We then compared the scores allocated and resolved differences by discussion. For the current third update version, the risk of bias judgements from the original review and its subsequent updates were carried over (Edwards 2003; Edwards 2007; Edwards 2009), and two authors used the Cochrane Collaboration’s tool for assessing the risk of bias (Higgins 2011) to assess the risk of bias for each newly added study as 'high', 'low', or 'unclear' risk of bias for the seven domains below:

1. Sequence generation;
2. Allocation concealment;
3. Blinding of participants and personnel;
4. Blinding of outcome assessment;
5. Incomplete outcome data;
6. Selective reporting;
7. Other sources of bias.

We reported the results relating to allocation concealment in all the studies included in this update, and results relating to the other domains for the newly added studies only.

#### Measures of the effect of the methods

We classified and analysed methods under broad strategies to increase questionnaire response, for example: **Incentives** ‐ What are participants offered? (e.g. monetary incentive vs. no incentive, unconditional incentive vs. conditional incentive, incentive with first vs. subsequent mailing); **Appearance** ‐ How does the questionnaire look? (e.g. more personalised vs. less, teaser on envelope vs. none); **Delivery** ‐ How are the questionnaires received or returned? (e.g. stamped vs. franked outward envelope, certified/special delivery vs. regular outward mailing); **Contact** ‐ Methods and number of requests for participation (e.g. pre‐contact vs. no pre‐contact, follow‐up vs. no follow‐up); **Content** ‐ Nature and style of questions (e.g. sensitive questions vs. no/fewer/less sensitive questions asked, demographic items first vs. last, horizontal vs. vertical orientation of response options); **Origin** ‐ Who sent the questionnaire? (e.g., University sponsor/source vs. other, sent or signed by more vs. less senior/well‐known person); **Communication** ‐ What are participants told? (e.g. assurance of confidentiality vs. none, participants told completion time 10 mins vs. 30 mins); **Length** ‐ How long is the questionnaire? (e.g. shorter vs. longer questionnaire, double postcard vs. one page).

In trials with factorial designs, we classified methods under two or more strategies. When methods were evaluated at more than two levels (e.g. highly, moderately and slightly personalised questionnaires), we combined the upper levels, creating a dichotomy. For example, we compared response to the least personalised questionnaire with the combined response to the moderately and highly personalised questionnaires. Monetary incentives were defined as any incentive that could be used by participants as money (e.g. cash). Incentives such as a donation to charity, entrance into a lottery, or a gift (e.g. a pen) were classified as ’non‐monetary’ incentives.

For each included study, we calculated an odds ratio and its 95% confidence interval as the measure of the effect of the method to increase questionnaire response.

#### Unit of analysis issues

In the small minority of studies that had used a cluster‐randomised design, we did not adjust the results to allow for clustering, but we noted that these were at risk of ‘Other sources of bias’.

#### Dealing with missing data

We wrote to the authors of reports where required information was missing. See Data extraction and management.

#### Assessment of heterogeneity

We assessed heterogeneity amongst trial odds ratios using a Chi‐squared test at a 5% significance level, and the degree of inconsistency between trial results was quantified using the I^2^ statistic (Higgins 2003). The I^2^ statistic measures the percentage of variation across studies, which is due to heterogeneity. We used the following categories for our interpretation of the I^2^ statistic (Higgins 2022):

- 0‐40% might not be important;
- 30‐60% moderate heterogeneity;
- 50‐90% substantial heterogeneity;
- 75‐100% considerable heterogeneity.

#### Assessment of reporting biases

We assessed evidence for small study bias (such as reporting bias) using Egger’s weighted regression method and Begg’s rank correlation test and funnel plots.

#### Data synthesis

For each of the broad strategies to increase questionnaire response, we estimated pooled odds ratios in Review Manager 2020 using a random‐effects model. We calculated 95% confidence intervals and two‐sided P values for each outcome.

#### Subgroup analysis and investigation of heterogeneity

As per our earlier review versions (Edwards 2003; Edwards 2007; Edwards 2009), no subgroup analyses or meta‐regressions were planned/performed.

#### Sensitivity analysis

As per our earlier review versions (Edwards 2003; Edwards 2007; Edwards 2009), no sensitivity analyses were planned/performed.

## Results

### Description of studies

#### Results of the search

For this update: of 92,453 identified abstracts and titles, we removed duplicates and screened 68,193 records/references for eligibility for inclusion. We selected 395 potentially eligible references for independent eligibility assessment of the full‐text reports. After screening full‐text reports, 114 were excluded (reasons for exclusion are summarised in Characteristics of excluded studies). Of the remaining 281 records, 32 were subsequently found to be duplicates, and 26 required further contact with the authors and are listed in Characteristics of studies awaiting classification (also lists the 23 studies awaiting classification from the previous update, Edwards 2009).

Therefore, our updated search identified a total of 223 full‐text records reporting on 245 new trials, bringing the total number of included trials to 758 (513 included in the previous update, Edwards 2009) (Characteristics of included studies).

#### Included studies

##### Postal questionnaires

We have identified a total of 670 eligible trials that evaluated over 100 different strategies for increasing response to postal questionnaires. See Characteristics of included studies for further details.

##### Electronic questionnaires

We have identified a total of 88 eligible trials that evaluated over 30 different strategies for increasing response to electronic questionnaires. See Characteristics of included studies for further details.

#### Excluded studies

From the latest updated search, we excluded 114 studies: 73 were not a randomised trial, 34 did not use a postal questionnaire, 6 were confounded trials, and 1 was a trial protocol. See Characteristics of excluded studies for further details (also lists the reasons for excluding 87 studies from the previous update, Edwards 2009).

### Risk of bias in included studies

See Figure 1 for our risk of bias assessments on the domain 'allocation concealment' in all the included studies in this update, and results relating to the other domains for the newly added studies only.

**1:**
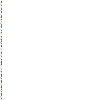


#### Allocation

Of the 513 previously identified trials, allocation concealment was classified as C (inadequate) in 76 trials, A (adequate) in 83 trials, and as B (unclear) in the remaining trials. Of the 245 newly added trials, we judged five trials to be at high risk of bias, 112 trials to be at low risk of bias, and there was unclear risk of bias in the remaining trials.

For sequence generation, of the 245 newly added trials, we judged five trials to be at high risk of bias, 110 trials to be at low risk of bias, and there was unclear risk of bias in the remaining trials.

#### Blinding

Of the 245 newly added trials, participants were not blinded to the intervention in 131 trials. No outcomes were assessed on the participants in any of the included trials (only the counts of responses in the experimental and control groups were reported), and so blinding of outcome assessors was not a risk of bias in any of the newly added trials.

#### Incomplete outcome data

Of the 245 newly added trials, exclusions were not reported in 34 trials. The remaining trials were at low risk of bias due to follow‐up and exclusions.

#### Selective reporting

Of the 245 newly added trials, 235 trials reported outcomes in full.

#### Other potential sources of bias

Of the 245 newly added trials, seven were judged to be at a ‘high’ risk of bias due to results not being adjusted for clustering.

### Effect of methods

Table 1 presents a summary of the main findings of this review update.

**1:** Summary of main results

**Strategy**	**Delivery method**	**No. of studies (no. of participants)**	**Effect size**
Monetary incentive	Postal	111 (226,209)	OR 1.86 (95% CI 1.73 to 1.99)
	Electronic	5 (6446)	OR 1.88 (95% CI 1.31 to 2.71)
Telephone reminder	Postal	4 (15,143)	OR 1.96 (95% CI 1.03 to 3.74)
Shorter questionnaire	Postal	72 (84,954)	OR 1.58 (95% CI 1.40 to 1.78)
	Electronic	5 (12,325)	OR 1.51 (95% CI 1.06 to 2.16)
Contact participants before sending questionnaires	Postal	59 (89,146)	OR 1.36 (95% CI 1.23 to 1.51)
Unconditional incentive	Postal	35 (48,850)	OR 1.53 (95% CI 1.35 to 1.74)
	Electronic	3 (1401)	OR 1.08 (95% CI 0.77 to 1.50)
Personalised SMS reminder	Postal	2 (901)	OR 1.53 (95% CI 0.97 to 2.42)
Special delivery service (e.g. recorded, registered, or certified delivery)	Postal	19 (30,492)	OR 1.68 (95% CI 1.36 to 2.08)
Electronic reminder (e.g. SMS or email)	Postal	2 (582)	OR 1.60 (95% CI 1.10 to 2.33)
Intensive follow‐up (e.g. questionnaires at 1, 6 and 12 months)	Postal	1 (431)	OR 1.69 (95% CI 0.93 to 3.06)
More ’interesting’ or high salient questionnaire (e.g. asking questions particularly relevant to the study participants)	Postal	4 (6491)	OR 1.73 (95% CI 1.12 to 2.66)
	Electronic	1 (2176)	OR 1.85 (95% CI 1.52 to 2.26)
Mention an obligation to respond	Postal	3 (600)	OR 1.61 (95% CI 1.16 to 2.22)
Non‐monetary incentive (e.g. Scratch‐card, donation to charity, offer of study results, candy, etc.)	Postal	146 (277,802)	OR 1.16 (95% CI 1.11 to 1.21)
	Electronic	16 (38,901)	OR 1.60 (95% CI 1.25 to 2.05)
Larger monetary incentive	Postal	50 (137,457)	OR 1.24 (95% CI 1.15 to 1.33)
Pen included with questionnaire	Postal	14 (46,096)	OR 1.32 (95% CI 1.14 to 1.53
Offering study results as an incentive	Electronic	2 (2884)	OR 1.36 (95% CI 1.16 to 1.59)
Personalised materials	Postal	75 (98,285)	OR 1.15 (95% CI 1.09 to 1.21)
	Electronic	12 (48,910)	OR 1.24 (95% CI 1.17 to 1.32)
White background in the email compared to black	Electronic	1 (6090)	(OR 1.31; 95% CI 1.10 to 1.56)
Simple header	Electronic	1 (5075)	OR 1.23 (95% CI 1.03 to 1.48)
Single‐sided questionnaire	Postal	5 (9383)	OR 1.13 (95% CI 1.02 to 1.25)
Stamped return envelope	Postal	28 (55,550)	OR 1.23 (95% CI 1.13 to 1.33)
Assurance of confidentiality	Postal	1 (25,000)	OR 1.33 (95% CI 1.24 to 1.42)
First‐class postage	Postal	2 (8300)	OR 1.11 (95% CI 1.02 to 1.21)
University sponsorship	Postal	14 (21,628)	OR 1.32 (95% CI 1.13 to 1.54)
	Electronic	2 (3845)	OR 0.84 (95% CI 0.69 to 1.01)
Stressing benefits to society	Electronic	3 (3536)	OR 1.38 (95% CI 1.07 to 1.78)
Giving a deadline	Electronic	1 (8586)	OR 1.18 (95% CI 1.03 to 1.34)
Telling participants it would take 30 minutes to complete compared with telling them that it would take 10 mins	Electronic	1 (2358)	OR 1.25 (95% CI 0.96 to 1.64)
“Survey” as subject compared to a blank subject line	Electronic	2 (3845)	OR 0.81 (95% CI 0.67 to 0.97)
Including a ’sensitive’ question	Postal	10 (21,393)	OR 0.94 (95% CI 0.88 to 1.00)

SMS: Short message service

#### Incentives ‐ What are participants offered? (Strategies 1‐22)

##### Postal questionnaires

One hundred and eleven trials (226,209 participants) evaluated the effect of a monetary incentive on questionnaire response. The odds of response were almost doubled using monetary incentives (odds ratio (OR) 1.86; 95% confidence interval (CI) 1.73 to 1.99). There was, however, considerable heterogeneity amongst the trial results (I^2^ = 85%) (Analysis 1.2). Fifty trials (137,457 participants) evaluated the effect of a larger rather than a smaller monetary incentive on questionnaire response. The odds of response were a quarter higher when a larger monetary incentive was used (OR 1.24; 95% CI 1.15 to 1.33). There was considerable heterogeneity amongst the trial results (I^2^ = 82%) (Analysis 2.2). Seventeen trials (28,212 participants) evaluated the effect of offering a monetary rather than a non‐monetary incentive on questionnaire response. The odds of response were increased by over half when a monetary incentive rather than a non‐monetary incentive was used (OR 95% CI 1.67; 95% CI 1.47 to 1.90). There was considerable heterogeneity amongst the trial results (I^2^ = 75%) (Analysis 3.2).

One hundred and forty‐six trials (277,802 participants) evaluated the effect of a non‐monetary incentive (e.g. key‐ring, lottery participation, donation to charity, offer of study results, candy, etc.) on questionnaire response. The odds of response were increased by over a tenth when a non‐monetary incentive was used (OR 1.16; 95% CI 1.11 to 1.21). There was considerable heterogeneity amongst the results of non‐monetary incentive trials (I^2^ = 80%) (Analysis 4.2). Eleven trials (18,688 participants) evaluated the effect of a larger rather than a smaller non‐monetary incentive on questionnaire response. There was a possibility that using a larger non‐monetary incentive may increase the odds of response (OR 1.15; 95% CI 1.00 to 1.33; P = 0.05). However, there was considerable heterogeneity amongst the trial results (I^2^ = 77%) (Analysis 5.2).

Thirty‐five trials (48,850 participants) evaluated the timing of incentives on questionnaire response. The odds of response increased by more than a half when incentives were given with questionnaires (i.e. unconditional) rather than when only given after participants had returned their questionnaires (i.e. conditional on response) (OR 1.53; 95% CI 1.35 to 1.74). There was considerable heterogeneity amongst the trial results (I^2^ = 89%) (Analysis 6.2). Four trials (8942 participants) evaluated the effect of offering an incentive with the first rather than a subsequent mailing. The odds of response were increased by over a tenth when the incentive was offered with the first mailing (OR 1.14; 95% CI 1.03 to 1.26). There was no evidence of heterogeneity amongst the trial results (I^2^ = 0%) (Analysis 7.2). Thirteen trials (20,052 participants) evaluated the effect of offering survey results as an incentive. There was no evidence for an effect on response of offering the study results (OR 0.91; 95% CI 0.78 to 1.05). There was considerable heterogeneity amongst the trial results (I^2^ = 76%) (Analysis 8.2).

Fourteen trials (46,096 participants) evaluated the effect on questionnaire response of sending a pen with the questionnaire compared to sending the questionnaire without a pen. The odds of response were increased by a third when a pen was included (OR 1.32; 95% CI 1.14 to 1.53). There was considerable heterogeneity amongst the trial results (I^2^ = 89%) (Analysis 9.2). A single trial (6167 participants) evaluated the effect of sending the questionnaire with a more expensive pen compared to sending the questionnaire with a cheaper pen. There was no evidence for an effect on response of sending a questionnaire with a more expensive pen (OR 1.03; 95% CI 0.81 to 1.31) (Analysis 10.2). The same trial (6167 participants) evaluated the effect of sending the questionnaire with a pen in a box compared to sending the questionnaire with an unboxed pen. The odds of response were increased by a tenth when the pen was in a box (OR 1.10; 95% CI 1.00 to 1.22) (Analysis 11.2). A single trial (2342 participants) evaluated the effect of sending a monetary incentive with the follow‐up mailing compared to no incentive with the follow‐up. There was no evidence for an effect on response of sending a monetary incentive with the follow‐up mailing (OR 0.97; 95% CI 0.82 to 1.16) (Analysis 12.2). A single trial (444 participants) evaluated the effect of sending the questionnaire with non‐monetary incentives compared to sending the questionnaire with the promise of making a charitable donation. The odds of response were nearly one half greater with a non‐monetary incentive than with a donation to charity (OR 1.44; 95% CI 0.98 to 2.12) (Analysis 13.1). A single trial (531 participants) evaluated the effect of sending the questionnaire with a cheque incentive that required the participant to give their social security number ID to cash it, compared to sending the questionnaire with a cheque incentive that did not require a social security number ID. There was no evidence for an effect on response when participants were required to give their social security number ID (OR 0.75; 95% CI 0.41 to 1.37) (Analysis 14.1). Eleven trials (19,981 participants) evaluated the effect on questionnaire response via including a study brochure with the questionnaire compared to no brochure. There was no evidence for an effect on response of including a study brochure (OR 0.97; 95% CI 0.83 to 1.13). There was moderate heterogeneity between the trial results (I^2^ = 64%) (Analysis 15.3). A single trial (303 participants) evaluated the effect of sending a cheque incentive compared to sending the questionnaire with a cashcard (i.e. a reloadable debit card). The odds of response were greater with a cheque than with a cashcard (OR 1.81; 95% CI 1.15 to 2.86) (Analysis 16.2). A single trial (2856 participants) evaluated the effect of sending a monetary incentive comprising multiple banknotes compared to a single note. There was no evidence for an effect on response with multiple banknotes compared to a single note (OR 1.08; 95% CI 0.94 to 1.26) (Analysis 17.2).

##### Electronic questionnaires

Five trials (6446 participants) evaluated the effect of a monetary incentive on electronic questionnaire response. The odds of response were almost doubled using monetary incentives (OR 1.88; 95% CI 1.31 to 2.71). There was considerable heterogeneity between the trial results (I^2^ = 79%) (Analysis 1.4). Three trials (3614 participants) evaluated the effect of a monetary rather than a non‐monetary incentive on e‑questionnaire response. There was no evidence for an effect on response of using a monetary rather than a non‐monetary incentive (OR 0.89; 95% CI 0.63 to 1.26). There was considerable heterogeneity between the trial results (I^2^ = 81%) (Analysis 3.4). Sixteen trials (38,901 participants) evaluated the effect of a non‐monetary incentive (e.g. Amazon gift cards, lottery participation, personal digital assistant, early grade feedback, etc.) on e‐questionnaire response. The odds of response were almost doubled when a non‐monetary incentive was used (OR 1.60; 95% CI 1.25 to 2.05). There was considerable heterogeneity amongst the trial results (I^2^ = 93%) (Analysis 4.4). Ten trials (37,382 participants) evaluated the effect of a larger rather than a smaller non‐monetary incentive on e‐questionnaire response. There was no evidence for an effect on response of using a larger non‐monetary incentive (OR 1.07; 95% CI 0.87 to 1.32). There was considerable heterogeneity amongst the trial results (I^2^ = 89%) (Analysis 5.4).

Three trials (1401 participants) evaluated the timing of incentives on e‐questionnaire response. There was no evidence for an effect on response when incentives were given with e‐questionnaires (i.e. unconditional) rather than only given after participants had submitted their e‐questionnaire (i.e. conditional on response) (OR 1.08; 95% CI 0.77 to 1.50) (Analysis 6.4). Two trials (2884 participants) evaluated the effect on e‐questionnaire response of offering survey results as an incentive. The odds of response increased by over a third when an offer of results was used (OR 1.36; 95% CI 1.16 to 1.59) (Analysis 8.3).

One trial (2233 participants) evaluated the effect of immediate notification of lottery results compared to delayed notification on e‐questionnaire response. The odds of response were increased by almost half when lottery results were immediately notified (OR 1.37; 95% CI 1.13 to 1.65) (Analysis 20.1). Two trials (4721 participants) evaluated the effect of higher denominations of currencies in a monetary lottery compared to lower denominations on e‐questionnaire response. There was no evidence for an effect on response of offering higher denominations in a monetary lottery (OR 1.00; 95% CI 0.87 to 1.14) (Analysis 18.1). One trial (1061 participants) evaluated the combined effect of conditional and unconditional incentives on e‐questionnaire response compared to conditional incentives alone. There was no evidence for an effect on response of using the combined incentives (OR 1.19; 95% CI 0.92 to 1.54) (Analysis 19.1). Another trial (3000 participants) evaluated the combined effect of conditional and unconditional incentives on e‐questionnaire response compared to unconditional incentives alone. This trial found evidence that response was increased using the combined incentives (OR 1.23; 95% CI 1.06 to 1.44) (Analysis 21.4). A single trial (130 participants) evaluated the effect on response to a smart‐phone daily diary app that included a game that gave in‐game rewards when a daily diary was completed. There was no evidence that the inclusion of the game increased response (OR 0.51; 95% CI 0.19 to 1.40) (Analysis 22.2)

#### Appearance ‐ How do the questionnaires look? (Strategies 23‐60)

##### Postal questionnaires

Seventy‐four trials (97,674 participants) evaluated the effect on questionnaire response of making questionnaire materials more personal, such as signing letters by hand. The odds of response were increased by more than a tenth with a more personalised approach to participants (OR 1.15; 95% CI 1.09 to 1.21). There was, however, considerable heterogeneity amongst the results of these trials (I^2^ = 57%) (Analysis 23.2). Fourteen trials (15,006 participants) evaluated the effect of cover letters bearing a handwritten signature compared to those that are typed or scanned or printed. The odds of response increased by a quarter using handwritten signatures (OR 1.24; 95% CI 1.08 to 1.41) (Analysis 24.2). Nine trials (6030 participants) evaluated the effect of handwritten address labels compared to computer‐printed labels. The odds of response increased by a quarter when using the handwritten labelled questionnaire (OR 1.23; 95% CI 1.09 to 1.37) (Analysis 25.2). Three trials (1364 participants) evaluated the presence of a signature within the questionnaire. There was some evidence for an effect on response of using a signature within the questionnaire (OR 1.35; 95% CI 1.04 to 1.76) (Analysis 26.2).

Ten trials (5297 participants) evaluated the effect of including an identifying feature, such as a participant’s name or identity number, on questionnaire response. There was no evidence for an effect on response of using an identifying feature (OR 1.03; 95% CI 0.81 to 1.32). There was considerable heterogeneity between the trial results (I^2^ = 71%) (Analysis 27.2). One trial (741 participants) evaluated the effect on response of an identifying number on the returned questionnaire compared with another identifier. There was no evidence for an effect on response of using an identifying number (OR 1.00; 95% CI 0.68 to 1.46) (Analysis 28.2).

Fifteen trials (43,754 participants) evaluated the effect on response of using questionnaires printed on coloured paper. There was no evidence for an effect on response of using a coloured questionnaire (OR 1.03; 95% CI 0.98 to 1.09) (Analysis 29.2). Three trials (7040 participants) evaluated the effect of using coloured ink, compared with black or blue ink, on questionnaire response. There was no evidence for an effect on response of using coloured ink (OR 1.16; 95% CI 0.95 to 1.42). There was moderate heterogeneity between the trial results (I^2^ = 67%) (Analysis 30.2). Two trials (2356 participants) evaluated the effect of a coloured letterhead compared to a black and white letterhead. There was no evidence for an effect on response of using a coloured letterhead (OR 1.08; 95% CI 0.91 to 1.28) (Analysis 31.2). A single trial (320 participants) evaluated the effect of an illustration on the cover of the questionnaire largely in black, versus largely in white. The odds of response increased by more than a half when using an illustration on the cover of the questionnaire that was largely in black (OR 1.62; 95% CI 1.04 to 2.53) (Analysis 32.1). Three trials (5681 participants) evaluated the effect on response of using a booklet compared to stapled pages. There was no evidence for an effect on response of using a booklet (OR 1.10; 95% CI 0.99 to 1.23) (Analysis 33.2). Two trials (2145 participants) evaluated the effect of the paper size of the questionnaire on response. There was no evidence for an effect on response of using a large paper size (OR 0.88; 95% CI 0.56 to 1.39) (Analysis 34.2). A single trial (176 participants) evaluated the effect on questionnaire response of printing the questionnaire using dot‐matrix compared to a letter‐quality print. There was no evidence for an effect of response of using the dot‐matrix print (OR 1.15; 95% CI 0.63 to 2.10) (Analysis 35.1).

Three trials (3372 participants) evaluated the effect of the questionnaire being printed on a high quality or thicker paper, compared to standard quality or thin paper. There was no evidence for an effect on response of using a high quality or a thicker paper (OR 0.83; 95% CI 0.68 to 1.02) (Analysis 36.1). Five trials (9383 participants) evaluated the effect of using a single‐sided questionnaire compared to a double‐sided questionnaire. The odds of response increased by a tenth when a single‐sided questionnaire was used (OR 1.13; 95% CI 1.02 to 1.25) (Analysis 37.2). One trial (650 participants) evaluated the effect on response of using a larger font compared to a smaller font. There was no evidence for an effect on response of using larger fonts (OR 1.26; 95% CI 0.87 to 1.82) (Analysis 38.1). A single trial (1000 participants) compared the presence of a study logo on several items in the mailing package to its presence in the questionnaire only. There was no evidence for an effect on response of using the study logo on several items in the mailing package (OR 0.92; 95% CI 0.72 to 1.18) (Analysis 39.1). Five trials (3956 participants) evaluated the effect of the presence of a picture in the questionnaire. There was no evidence for an effect on response of using a picture (OR 1.03; 95% CI 0.70 to 1.51) (Analysis 40.2). One trial (280 participants) evaluated the effect on response of including a cartoon in the questionnaire. There was no evidence for an effect on response of including a cartoon (OR 1.00; 95% CI 0.62 to 1.62) (Analysis 42.1). Two trials (2904 participants) evaluated the effect on response of questionnaires having a more professional design compared with a standard design. There was no evidence for an effect on response of questionnaires having a more professional design (OR 1.00; 95% CI 0.58 to 1.72). There was considerable heterogeneity between the trial results (I^2^ = 88%) (Analysis 43.2).

Two trials (901 participants) evaluated the effect on questionnaire response of sending personalised SMS reminders to non‐respondents compared with standard SMS reminders. There was some evidence that the odds of response were increased with personalised SMS reminders (OR 1.53; 95% CI 0.97 to 2.43) (Analysis 44.2). A single trial (231 participants) evaluated the effect on response of using "Action Required" as the subject line of an email reminder compared with “Questionnaire reminder” as the subject. There was no evidence for an effect on response of using "Action Required" as the subject line (OR 0.61; 95% CI 0.23 to 1.63) (Analysis 45.2). Two trials (3895 participants) evaluated the effect on response of including a message about an incentive on the envelope compared with none. There was no evidence for an effect on response by including a message (OR 0.91; 95% CI 0.80 to 1.04) (Analysis 46.2). A single trial (1569 participants) evaluated the effect on response of a health message on the envelope compared with a monetary incentive message. There was no evidence that response differed between the health or the monetary incentive messages (OR 1.05; 95% CI 0.86 to 1.29) (Analysis 47.2). Five trials (23,621 participants) evaluated the effect of including a ‘teaser’ on the envelope. There was no evidence for an effect on response when a teaser was used (OR 1.08; 95% CI 0.95 to 1.22) There was moderate heterogeneity amongst the trial results (I^2^ = 37%) (Analysis 48.2). Two trials (1678 participants) evaluated the effect of using a more readable/concise/info‐mapped letter on response. There was no evidence for an effect on response of using a more readable/concise/info‐mapped letter (OR 0.88; 95% CI 0.72 to 1.09) (Analysis 49.2). A single trial (517 participants) evaluated the effect on response of a study logo sticker on the envelope compared with no sticker. There was no evidence that response was increased with the sticker (OR 1.06; 95% CI 0.75 to 1.49) (Analysis 50.1).

##### Electronic

Twelve trials (48,910 participants) evaluated the effect on e‐questionnaire response by addressing the salutations in the cover letters accompanying the questionnaires personally, or by giving a touch of personalisation to the cover letters. The odds of response were increased by about a quarter when a personalised approach was adopted (OR 1.24; 95% CI 1.17 to 1.32). There was moderate heterogeneity between trial results (I^2^ = 41%) (Analysis 23.4). Two trials (720 participants) evaluated the effect of the presence of a picture in the email. The odds of response tripled when a picture was sent in the email (OR 3.05; 95% CI 1.84 to 5.06) (Analysis 40.3). The same trials (520 participants) evaluated the effect of response when a more attractive picture was used compared to a less attractive picture. There was little evidence for an effect on response of using a more attractive picture (OR 3.44; 95% CI 0.72 to 16.49) (Analysis 41.1).

Two trials (6152 participants) evaluated the presence of a topic in the subject line of the email compared to a blank subject line. There was no evidence for an effect on response of using a topic in the subject line (OR 0.84; 95% CI 0.71 to 1.01) (Analysis 51.2). Two trials (3845 participants) evaluated the presence of “Survey” as the subject line compared to a blank subject line. The odds of response decreased by a fifth when “Survey” was mentioned in the subject line (OR 0.81; 95% CI 0.67 to 0.97) (Analysis 52.2).

One trial (6090 participants) evaluated the effect of sending emails in text‐file format compared to HTML. There was no evidence for an effect on response of using text file format (OR 1.00; 95% CI 0.84 to 1.19) (Analysis 53.1). The same trial (6090 participants) evaluated the presence of using a white background in the email compared to a black background. The odds of response increased by over a quarter when a white background was used (OR 1.31; 95% CI 1.10 to 1.56) (Analysis 54.1). The same trial (6090 participants) also evaluated the effect of including a header compared to no header in the email. There was no evidence for an effect on response of using a header (OR 1.13; 95% CI 0.90 to 1.41) (Analysis 55.1). The same trial (5075 participants) also evaluated the effect of a simple header compared to a complex header. The odds of response increased by almost a quarter when a simple header was used (OR 1.23; 95% CI 1.03 to 1.48) (Analysis 56.1).

One trial (5413 participants) evaluated the effect of textual presentation of response categories compared to visual presentation of response categories. The odds of response increased by almost a fifth when textual presentation was used (OR 1.19; 95% CI 1.05 to 1.36) (Analysis 57.1). A single trial (517 participants) evaluated the effect on response of formatting a web survey as a single scrollable page compared with multiple pages. There was no evidence for an effect on response of using a single scrollable page (OR 0.93; 95% CI 0.66 to 1.32) (Analysis 58.1). Two trials (3676 participants) evaluated the effect on e‐questionnaire response of an email subject line that emphasised an incentive compared with no such emphasis on the subject. There was no evidence for an effect on response when the email subject emphasised an incentive (OR 2.19; 95% CI 0.58 to 8.27). There was considerable heterogeneity between the trial results (I^2^ = 97%) (Analysis 59.2). A single trial (2963 participants) evaluated the effect on e‐questionnaire response of an email reminder including humour compared to a standard email. There was no evidence for an effect on response when including humour (OR 1.17; 95% CI 0.99 to 1.38) (Analysis 60.1).

#### Delivery ‐ How are the questionnaires received or returned? (Strategies 61‐93)

##### Postal

Six trials (13,964 participants) evaluated the effect on questionnaire response of using stamps on outgoing envelopes compared to franked envelopes. There was no evidence for an effect on response of using stamps on outgoing envelopes (OR 0.95; 95% CI 0.88 to 1.03) (Analysis 61.2). Two trials (8300 participants) evaluated the effect on questionnaire response of using first class compared to other classes of postage. The odds of response were increased by over one‐tenth using first‐class postage (OR 1.11; 95% CI 1.02 to 1.21) (Analysis 62.2).

Five trials (5461 participants) evaluated the effect on questionnaire response of using commemorative stamps rather than standard stamps on return envelopes. There was no evidence for an effect on response of using commemorative stamps (OR 0.92; 95% CI 0.81 to 1.06) (Analysis 63.2). Nineteen trials (30,492 participants) evaluated the effect on questionnaire response of using a special delivery service (e.g. recorded, registered, or certified delivery), rather than standard delivery. The odds of response increased by more than half when special delivery was used (OR 1.68; 95% CI 1.36 to 2.08). Results were considerably heterogeneous (I^2^ = 87%) (Analysis 64.2). Twenty‐eight trials (55,550 participants) evaluated the effect on questionnaire response of using a stamped return envelope compared to a pre‐paid business or franked reply envelope. The odds of response increased by a quarter when stamps were used (OR 1.23; 95% CI 1.13 to 1.33). There was moderate heterogeneity between the trial results (I^2^ = 69%) (Analysis 65.2). One trial (205 participants) evaluated the effect of using priority stamps on return envelopes compared to using a first‐class stamp. The odds of response decreased by more than a half when priority stamps were used (OR 0.26; 95% CI 0.14 to 0.46) (Analysis 66.1). One trial (800 participants) evaluated the effect of using a first‐class stamp on return envelopes compared to a second‐class stamp. There was no evidence for an effect on response of using a first‐class stamp on the return envelope (OR 0.91; 95% CI 0.69 to 1.21) (Analysis 67.1).

A single trial (510 participants) evaluated the use of multiple stamps on return envelopes compared to a single stamp. The odds of response increased by almost half when multiple stamps were used (OR 1.44; 95% CI 1.01 to 2.04) (Analysis 68.1). Four trials (4094 participants) evaluated the effect on questionnaire response of providing any sort of pre‐paid return envelope rather than none. There was no evidence for an effect on response of including pre‐paid envelopes (OR 1.09; 95% CI 0.71 to 1.68). There was considerable heterogeneity amongst the trial results (I^2^ = 87%) (Analysis 69.2). A single trial (147 participants) evaluated the effect of including a stamped addressed return envelope compared to only including an address label. This trial provided no evidence for an effect on response of using a stamped addressed return envelope (OR 0.86; 95% CI 0.45 to 1.65) (Analysis 70.1).

Two trials (1140 participants) evaluated the effect on response of sending questionnaires to the participant’s work address rather than to their home address. There was no evidence for an effect on response of sending questionnaires to work addresses (OR 1.16; 95% CI 0.89 to 1.52) (Analysis 71.2). Two trials (11,781 participants) evaluated the effect of using a window envelope on questionnaire response. There was no evidence for an effect on response of using window envelopes (OR 0.96; 95% CI 0.61 to 1.49). There was considerable heterogeneity between the trial results (I^2^ = 75%) (Analysis 72.2). A single trial (1200 participants) evaluated the effect on questionnaire response of sending the questionnaire in a larger envelope compared to a standard or smaller envelope. There was no evidence for an effect on response of using larger envelopes (OR 0.93; 95% CI 0.74 to 1.17) (Analysis 73.1).

Six trials (9756 participants) evaluated the effect on questionnaire response of using brown envelopes compared to white. There was no evidence for an effect on response of using a brown envelope (OR 1.25; 95% CI 0.86 to 1.80). There was considerable heterogeneity between the trial results (I^2^ = 92%) (Analysis 181.2). Two trials (1843 participants) evaluated the effect of questionnaires being mailed on Monday compared to being sent on Friday. There was no evidence for an effect on response of sending the questionnaire on Monday (OR 0.84; 95% CI 0.70 to 1.01) (Analysis 74.2). Two trials (2324 participants) evaluated the effect on response of questionnaires being sent one to five weeks after discharge from hospital, compared to being sent after 9 to 14 weeks. There was little evidence for an effect on response of questionnaires being sent sooner after discharge from hospital (OR 2.26; 95% CI 0.69 to 7.37). There was considerable heterogeneity between the trial results (I^2^ = 83%) (Analysis 76.1).

One trial (460 participants) evaluated the effect of a questionnaire being received on a Monday, compared to being received on a Friday. There was no evidence for an effect on response of questionnaires being received on a Monday (OR 1.00; 95% CI 0.64 to 1.56) (Analysis 75.1). One trial (1600 participants) evaluated the effect on response of using a padded envelope compared to a priority mail envelope. There was no evidence for an effect on response of using a padded envelope (OR 0.88; 95% CI 0.72 to 1.07) (Analysis 77.1).

A small trial (135 participants) evaluated the effect on response of the questionnaire being hand‐delivered by a person known to the recipient compared to standard postal delivery. The odds of response were more than doubled when the questionnaire was hand‐delivered by a known person (OR 2.60; 95% CI 1.29 to 5.23) (Analysis 78.1). Two trials (937 participants) evaluated the effect on questionnaire response of hand delivery compared to postal delivery. There was no evidence overall that response was increased using hand delivery (OR 1.44; 95% CI 0.50 to 4.15). There was considerable heterogeneity amongst the trial results (I^2^ = 88%) (Analysis 79.1). One trial (199 participants) evaluated the effect on response of sending a postal questionnaire compared with sending it by fax. The odds of response were almost halved when sending by fax (OR 0.58; 95% CI 0.29 to 1.14) (Analysis 80.2).

##### Electronic

Twenty‐seven trials (66,118 participants) evaluated the effect on response of sending a postal questionnaire compared with sending an e‐questionnaire. The odds of response were almost doubled using a postal questionnaire (OR 1.76; 95% CI 1.34 to 2.32). . There was, however, considerable heterogeneity between the trial results (I^2^ = 98%) (Analysis 81.2). Eight trials (20,909 participants) evaluated the effect of providing a choice of response modes (i.e. postal with optional electronic response) compared to postal only. There was no evidence for an effect on response by providing an optional electronic response mode (OR 0.94; 95% CI 0.86 to 1.02). There was moderate heterogeneity amongst the trial results (I^2^ = 51%) (Analysis 82.2).

Four trials (2958 participants) evaluated the effect on response of sending a postal questionnaire first with electronic follow‐up compared to an e‐questionnaire first with postal follow‐up. There was no evidence for an effect on response of sending a postal questionnaire first (OR 1.19; 95% CI 0.76 to 1.87). There was considerable heterogeneity between the trial results (I^2^ = 85%) (Analysis 83.2). Ten trials (39,523 participants) evaluated the effect on response of providing a choice of response modes (electronic or postal response) compared to electronic only. Response was increased when providing a choice of response modes (electronic or postal response) compared to electronic only (OR 1.63; 95% CI 1.18 to 2.26). There was considerable heterogeneity between the trial results (I^2^ = 97%) (Analysis 84.2). A single trial (6188 participants) evaluated the effect on response of asking participants to request their desired type of questionnaire compared to offering a choice of a postal or an e‐questionnaire immediately. The odds of response were one‐half greater when offering the choice of postal or an e‐questionnaire immediately (OR 1.59; 95% CI 1.43 to 1.77) (Analysis 85.2).

A single trial (2774 participants) evaluated the effect on response of administering the e‐questionnaire by computer compared to by smartphone. The odds of response were increased when using a computer rather than a smartphone (OR 1.62; 95% CI 1.36 to 1.94) (Analysis 86.2). A single trial (620 participants) evaluated the effect on response of administering the questionnaire by smartphone and Web, compared to by post with email follow‐up contacts. There was no evidence for an effect on response of administering the questionnaire by smartphone and Web (OR 1.02; 95% CI 0.50 to 2.08) (Analysis 87.2). One trial (195 participants) evaluated the effect on response of sending an e‐questionnaire compared with sending it by fax. The odds of response were almost four times greater with an e‐questionnaire than with a fax (OR 3.87; 95% CI 2.0 to 7.49) (Analysis 88.2).

A single trial (382 participants) evaluated the effect on response of administering the questionnaire by SMS compared to by post. There was no evidence for an effect on response of administering the questionnaire by SMS (OR 1.19; 95% CI 0.60 to 2.32) (Analysis 89.2). One trial (1943 participants) evaluated the effect of an e‐questionnaire being received on a Monday or Tuesday, compared to being received on a Friday. There was no evidence for an effect on response of e‐questionnaires being received on a Monday or Tuesday (OR 0.96; 95% CI 0.66 to 1.40) (Analysis 75.3). A single trial (21,473 participants) evaluated the effect on response to a Web survey of varying the days on which invitation emails and reminders were sent. The odds of response were greater when fixing the days on which invitation emails and reminders are sent (OR 1.08; 95% CI 1.03 to 1.14) (Analysis 90.2). The same trial evaluated the effect on response to a Web survey using a model to predict the best day on which to send invitation emails and reminders. The odds of response were greater when fixing the days on which invitation emails and reminders were sent (OR 1.05; 95% CI 1.00 to 1.11) (Analysis 91.2).

One trial (1999 participants) evaluated the effect on response of sending a postal follow‐up to an e‐questionnaire compared with follow‐up using interactive voice response. The odds of response were over three‐quarters greater with postal follow‐up (OR 1.77; 95% CI 1.48 to 2.11) (Analysis 92.2). One trial (353 participants) evaluated the effect on response of administering the questionnaire by SMS compared to by Web. There was no evidence for an effect on response of administering the questionnaire by SMS (OR 0.68; 95% CI 0.31 to 1.49) (Analysis 93.2).

#### Contact ‐ Methods and number of requests for participation (Strategies 94‐121)

##### Postal

Fifty‐nine trials (89,146 participants) evaluated the effect on response of contacting participants before sending questionnaires. The odds of response were increased by a third when participants were pre‐notified (OR 1.36; 95% CI 1.23 to 1.51). There was considerable heterogeneity amongst the trial results (I^2^ = 87%) (Analysis 94.2). Seven trials (3322 participants) evaluated the effect on response of pre‐notification by telephone compared to by post. There was no evidence for an effect on response when participants were pre‐contacted by telephone instead of by post (OR 1.18; 95% CI 0.77 to 1.80). There was considerable heterogeneity amongst the trial results (I^2^ = 85%) (Analysis 95.2). Twenty‐four trials (53,555 participants) evaluated the effect on questionnaire response of follow‐up contact (e.g. repeat mailings or telephone calls) with participants who did not respond to the initial questionnaire. The odds of response increased by more than a quarter when follow‐up contact was used (OR 1.33; 95% CI 1.18 to 1.49). There was considerable heterogeneity amongst the results (I^2^ = 75%) and both Begg’s and Egger’s tests indicated evidence of selection bias (Analysis 96.2).

Thirteen trials (11,456 participants) evaluated the effect on response of providing participants with another copy of the questionnaire during postal follow‐up. The odds of response were increased by nearly a half when questionnaires were included during postal follow‐up (OR 1.41; 95% CI 1.13 to 1.77). There was considerable heterogeneity amongst these results (I^2^ = 82%) (Analysis 97.2). Eight trials (4057 participants) evaluated the effect on questionnaire response of using telephone rather than postal follow‐up. There was no evidence for an effect on response of using telephone follow‐up (OR 1.02; 95% CI 0.76 to1.38). There was moderate heterogeneity amongst the trial results (I^2^ = 66%) (Analysis 98.2).

Four trials (15,143 participants) evaluated the effect on response of a telephone reminder compared to no reminder. There was good evidence for an effect on response of using a telephone reminder (OR 1.96; 95% CI 1.03 to 3.74). There was considerable heterogeneity amongst the trial results (I^2^ = 90%) (Analysis 99.2). Six trials (7520 participants) evaluated the effect on questionnaire response of using a higher frequency follow‐up interval compared to a lower frequency follow‐up interval. The odds of response were increased by over one‐tenth using a higher frequency follow‐up interval (OR 1.13; 95% CI 1.02 to 1.25) (Analysis 100.2). One trial (780 participants) evaluated the effect on response of contacting participants by letter before sending questionnaires compared to pre‐contact by postcard. There was no evidence for an effect on response when participants were pre‐contacted by postcard instead of by letter (OR 0.98; 95% CI 0.74 to 1.30) (Analysis 101.2).

One trial (581 participants) evaluated the effect on response of contacting participants by letter before sending questionnaires compared to pre‐contact by email. There was no evidence for an effect on response when participants were pre‐contacted by letter instead of by email (OR 1.25; 95% CI 0.83 to 1.88) (Analysis 102.2). One trial (930 participants) evaluated the effect on response of contacting participants by fax before sending questionnaires compared to pre‐contact by post. There was no evidence for an effect on response when participants were pre‐contacted by fax instead of by post (OR 0.92; 95% CI 0.71 to 1.20) (Analysis 103.2). Two trials (582 participants) evaluated the effect on questionnaire response of follow‐up contact with participants by SMS or email, compared with no reminders. There was no evidence for an effect on response when electronic reminders were used (OR 1.80; 95% CI 0.88 to 3.68). There was moderate heterogeneity between the trial results (I^2^ = 34%) (Analysis 104.2).

Two trials (3824 participants) evaluated the effect on response of push‐to‐web (i.e. initial requests sent by post and participants are asked to complete questionnaires over the Web) compared to mail‐push (initial mail contact with reminders of the paper questionnaire and an option to complete the survey online). There was no evidence for an effect on response with mail‐push (OR 1.10; 95% CI 0.87 to 1.39). There was moderate heterogeneity between the trial results (I^2^ = 60%) (Analysis 105.2). Four trials (3998 participants) evaluated the effect on response of sending a mixed‐mode reminder compared to a postal reminder. There was no evidence for an effect on response when non‐respondents were sent a mixed‐mode reminder (OR 1.13; 95% CI 0.83 to 1.52). There was moderate heterogeneity amongst the trial results (I^2^ = 52%) (Analysis 106.1).

Four trials (520 participants) evaluated the effect on response of a telephone reminder in addition to a postal reminder compared to a postal reminder only. The odds of response were increased by more than one‐half when a telephone reminder was included (OR 1.63; 95% CI 1.06 to 2.50). There was moderate heterogeneity amongst the trial results (I^2^ = 26%) (Analysis 107.2). Five trials (24,373 participants) evaluated the effect on response to a web survey of an email invitation compared to a postal invitation. There was no evidence for an effect on response when using a postal invitation (OR 1.81; 95% CI 0.81 to 4.01). There was considerable heterogeneity amongst the trial results (I^2^ = 98%) (Analysis 108.2). A single trial (431 participants) evaluated the effect on response of intensive follow‐up (i.e. questionnaires at 1, 6 and 12 months) compared with limited follow‐up (one questionnaire at 12 months). There was no evidence for an effect on response with intensive follow‐up (OR 1.69; 95% CI 0.93 to 3.06) (Analysis 109.1).

Two trials (771 participants) evaluated the effect on response of a pre‐contact SMS (on the day of mailing) compared to a post‐notification SMS (a few days following mailing). There was no evidence for an effect on response when a post‐notification SMS was used (OR 1.29; 95% CI 0.66 to 2.54). There was moderate heterogeneity amongst the trial results (I^2^ = 56%) (Analysis 110.2). A single trial (5837 participants) evaluated the effect on response of sending a postal questionnaire with an electronic reminder compared to sending an e‐questionnaire with a postal reminder. There was no evidence of an effect on response when participants were sent an e‐questionnaire with a postal reminder (OR 1.05; 95% CI 0.95 to 1.16) (Analysis 111.2). A single trial (296 participants) evaluated the effect on response of giving study participants a calendar with prompts for when to return questionnaires. There was no evidence for an effect on response when participants were given a calendar with prompts (OR 1.00; 95% CI 0.57 to 1.73) (Analysis 112.2).

##### Electronic

Three trials (3,049 participants) evaluated the effect on e‐questionnaire response of contacting participants before sending questionnaires. The odds of response were almost doubled when participants were pre‐notified (OR 1.85; 95% CI 0.99 to 3.45). There was considerable heterogeneity amongst the trial results (I^2^ = 80%) (Analysis 94.4).

Three trials (9947 participants) evaluated the effect of an SMS reminder compared to a postcard reminder. The odds of response increased by half when an SMS reminder was used (OR 1.49; 95% CI 1.23 to 1.81). There was moderate heterogeneity amongst the trial results (I^2^ = 61%) (Analysis 113.1). Three trials (7,159 participants) evaluated the effect on questionnaire response of follow‐up contact with participants by email compared with a mixed‐mode reminder (email and postal). The odds of response were doubled when a mixed‐mode reminder was used (OR 1.96; 95% CI 0.89 to 4.31). There was, however, considerable heterogeneity between the trial results (I^2^ = 94%) (Analysis 114.2).

A single trial (734 participants) evaluated the effect on response of using a mixed‐mode first contact compared to electronic only. The odds of response were increased by half with a mixed‐mode first contact (OR 1.54; 95% CI 1.15 to 2.07) (Analysis 115.2).

One trial (500 participants) evaluated the effect on e‐questionnaire response of contacting participants by letter before sending e‐questionnaires compared to pre‐contact by postcard. There was no evidence for an effect on response when participants were pre‐contacted by postcard instead of by letter (OR 1.38; 95% CI 0.76 to 2.49) (Analysis 101.3). Four trials (26,482 participants) evaluated the effect on response of push‐to‐web (i.e. where initial requests are sent by post and participants are asked to complete questionnaires over the Web) compared to providing a choice of response modes (i.e. electronic or postal response). There was no evidence for an effect on response when providing a choice of response modes (electronic or postal response) compared to push‐to‐web (OR 1.09; 95% CI 0.99 to 1.20). There was moderate heterogeneity between the trial results (I^2^ = 34%) (Analysis 116.2).

A single trial (3508 participants) evaluated the effect on response of push‐to‐web (i.e. where initial requests are sent by post and participants are asked to complete questionnaires over the Web) compared to mail only. The odds of response were increased by one‐quarter using mail only (OR 1.26; 95% CI 1.10 to 1.45) (Analysis 117.2). The same trial evaluated the effect on response of mail‐push (initial mail contact with reminders of the paper questionnaire and an option to complete the survey online) compared to mail only. There was no evidence for an effect on response when mail‐push was used (OR 0.96; 95% CI 0.84 to 1.10) (Analysis 118.2).

A single trial (2982 participants) evaluated the effect on response of email augmentation of push‐to‐web (i.e. the addition of emailed versions of the advance letter and reminders, where participants are asked to complete questionnaires over the Web) compared with push‐to‐web without email augmentation. There was no evidence for an effect on response with email augmentation (OR 1.13; 95% CI 0.98 to 1.31) (Analysis 119.1).

A single trial (178 participants) evaluated the effect on response of sending an SMS reminder with a link to the e‐questionnaire compared to sending an SMS reminder without a link. There was no evidence for an effect on response when participants were sent an SMS reminder with a link (OR 1.00; 95% CI 0.55 to 1.82) (Analysis 120.2). A single trial (125 participants) evaluated the effect on response of sending an electronic prompt (email or SMS). There was no evidence for an effect on response when participants were sent an electronic prompt (OR 1.27; 95% CI 0.47 to 3.48) (Analysis 121.1).

#### Content ‐ Nature and style of questions (Strategies 122‐143)

##### Postal

Ten trials (21,393 participants) evaluated the effect on response of including a ’sensitive’ question in a questionnaire. The odds of response were reduced by nearly one‐tenth when sensitive questions were included (OR 0.94; 95% CI 0.88 to 1.00) (Analysis 122.2). A single trial (5817 participants) evaluated the effect on response of placing the more relevant questions at the start of the questionnaire. The odds of response were increased by a quarter when more relevant questions were placed first (OR 1.23; 95% CI 1.10 to 1.37) (Analysis 123.2). Three trials (11,435 participants) evaluated the effect on response of placing the most general questions at the start of the questionnaire. There was no evidence for an effect on response of placing general questions first (OR 0.95; 95% CI 0.83 to 1.09) (Analysis 124.1). Five trials (10,565 participants) evaluated the effect on questionnaire response of placing questions asking for demographic information first. There was no evidence for an effect on response of placing demographic items first (OR 1.02; 95% CI 0.90 to 1.16) (Analysis 125.2). Two trials (3182 participants) evaluated the effect on response of placing the easiest questions at the start of the questionnaire. The odds of response were increased by over a half when the easiest questions were presented first (OR 1.61; 95% CI 1.14 to 2.26) (Analysis 126.2). Two trials (4087 participants) evaluated the effect on response of using a more ’user‐friendly’ questionnaire. The odds of response were increased by almost half using user‐friendly questionnaires (OR 1.47; 95% CI 1.25 to 1.73) (Analysis 127.2). Four trials (6491 participants) evaluated the effect on response of using a more ’interesting’ or high salient questionnaire (e.g. asking questions particularly relevant to the study participants). The odds of response were nearly doubled using more interesting/salient questionnaires (OR 1.73; 95% CI 1.12 to 2.66). There was considerable heterogeneity between the trial results (I^2^ = 91%) (Analysis 128.2). Four trials (3092 participants) evaluated the effect on questionnaire response of using open‐ended rather than closed questions. The odds of response were reduced by more than half when open‐ended questions were used (OR 0.43; 95% CI 0.19 to 0.98). There was considerable heterogeneity between the trial results (I^2^ = 95%) (Analysis 129.2). One trial (300 participants) evaluated the effect of using open‐ended items first compared to other items first. There was no evidence for an effect on response of using open‐ended items first (OR 1.26; 95% CI 0.73 to 2.19) (Analysis 130.2). The same trial (300 participants) evaluated the effect of using closed‐ended items first compared to other items first. There was no evidence for an effect on response of using closed‐ended items first (OR 0.93; 95% CI 0.54 to 1.59) (Analysis 131.2). A single trial (1360 participants) evaluated the effect on response of including ’don’t know’ boxes for questions. There was no evidence for an effect on response of including ’don’t know’ boxes (OR 1.03; 95% CI 0.82 to 1.29) (Analysis 132.1). Two trials (1125 participants) evaluated the effect on response of using a “circle answer” rather than “tick box” format on question responses. There was no evidence for an effect on response of using a circle answer format (OR 0.96; 95% CI 0.74 to 1.26) (Analysis 133.1). A single trial (6783 participants) evaluated the effect of listing response options in increasing order on questionnaire response. There was no evidence for an effect on response of listing response options in increasing order (OR 1.06; 95% CI 0.94 to 1.18) (Analysis 134.1). Two trials (3882 participants) evaluated the effect on response of using high‐frequency response alternatives compared to medium‐frequency response alternatives. There was no evidence for an effect on response when high‐frequency response alternatives were used (OR 1.40; 95% CI 0.58 to 3.38). There was considerable heterogeneity between the trial results (I^2^ = 85%) (Analysis 135.1). Another trial (654 participants) evaluated the effect on questionnaire response of using a 5‐step response scale compared to a 10‐step response scale. There was no evidence for an effect on response of using a 5‐step response scale (OR 0.78; 95% CI 0.52 to 1.19) (Analysis 136.1). A single trial (1500 participants) evaluated the effect of using an individual‐item rather than a stem‐and‐leaf format on questionnaire response. There was no evidence for an effect on response of using an individual item format (OR 0.88; 95% CI 0.70 to 1.10) (Analysis 137.1). One trial (400 participants) evaluated the horizontal orientation of response options compared to vertical orientation of response options. The odds of response were three times greater when horizontal rather than vertical orientation was used (OR 3.12; 95% CI 1.63 to 5.96) (Analysis 138.1). Four trials (7345 participants) evaluated the effect on response of using a conventional mode of response technique compared to a randomised response technique. There was no evidence for an effect on response of using the conventional mode of response technique (OR 1.52; 95% CI 0.85 to 2.72) (Analysis 139.2). A single trial (1280 participants) evaluated the effect on response of asking ’factual’ questions only compared to factual and attitudinal questions. The odds of response were increased by more than a quarter using factual questions only (OR 1.34; 95% CI 1.01 to 1.77) (Analysis 140.1). One trial (200 participants) evaluated the effect on response of using a multi‐option consent form compared to a standard consent form. There was no evidence for an effect on response of using a multi‐option consent form (OR 0.91; 95% CI 0.49 to 1.68) (Analysis 141.1). One trial (259 participants) evaluated the effect on response of questions ordered by time period compared to those not ordered by time period. There was no evidence for an effect on response of using questionnaires where questions are ordered by time period (OR 1.48; 95% CI 0.84 to 2.59) (Analysis 142.1). Two trials (226 participants) evaluated the effect on response of placing clinical outcome questions first compared to placing them last. The odds of response were doubled when clinical outcome questions were last (OR 2.05; 95% CI 0.99 to 4.25) (Analysis 143.2).

##### Electronic

One trial (2176 participants) evaluated the effect on response of using a more 'interesting' e‐questionnaire (e.g. asking questions particularly relevant to the study participants). The odds of response were almost doubled using a more interesting e‐questionnaire (OR 1.85; 95% CI 1.52 to 2.26) (Analysis 128.3).

#### Origin ‐ Who sent the questionnaire? (Strategies 144‐150)

##### Postal

Fourteen trials (21,628 participants) evaluated the effect on response of university sponsorship. The odds of response were increased by more than a quarter when questionnaires originated from a university rather than an alternative source, such as a government department or commercial organisation (OR 1.32; 95% CI 1.13 to 1.54). There was considerable heterogeneity between trial results (I^2^ = 83%) (Analysis 144.2). Eleven trials (5686 participants) evaluated the effect on response when questionnaires were sent or signed by a more senior or well‐known person. There was no evidence for an effect on response when a more senior or well‐known person sent or signed the questionnaire (OR 1.05; 95% CI 0.90 to 1.23). There was moderate heterogeneity between the trial results (I^2^ = 41%) (Analysis 147.2).

A single trial (500 participants) evaluated the effect on questionnaire response of sending the questionnaire in a university‐printed envelope. There was no evidence for an effect on response of sending the questionnaire in a university‐printed envelope (OR 0.88; 95% CI 0.61 to 1.28) (Analysis 146.2). Two trials (924 participants) evaluated the effect on response of pre‐contact by a medical researcher compared to a nonmedical researcher. There was no evidence for an effect on response of pre‐contact by a medical researcher (OR 1.01; 95% CI 0.55 to 1.86). There was considerable heterogeneity between the trial results (I^2^ = 72%) (Analysis 148.1). Two trials (1106 participants) evaluated the effect on response when questionnaires were sent from a GP rather than a research group. There was no evidence for an effect on response of sending questionnaires by a GP (OR 1.52; 95% CI 0.73 to 3.15). There was considerable heterogeneity between the trial results (I^2^ = 84%) (Analysis 149.2). Five trials (5959 participants) evaluated the effect on response of whether the ethnicity of the name of the person sending the questionnaire was identifiable. There was no evidence for an effect on response when names were ethnically identifiable (OR 1.07; 95% CI 0.90 to 1.27) (Analysis 180.2). Two trials (3146 participants) evaluated the effect of sending the questionnaire from a male investigator compared to a female investigator. There was no evidence for an effect on response of sending the questionnaire from a male investigator (OR 1.07; 95% CI 0.72 to 1.58) (Analysis 150.2).

##### Electronic

Two trials (3845 participants) evaluated the effect on e‐questionnaire response of university sponsorship. There was no evidence for an effect on e‐questionnaire response by using university sponsorship (OR 0.84; 95% CI 0.69 to 1.01) (Analysis 144.4). Two trials (658 participants) evaluated the effect on e‐questionnaire response of ‘higher’ university sponsorship (i.e. university logo featured prominently on every page of the surveys) compared with ‘lower’ university sponsorship (i.e. university logo did not appear anywhere on the surveys, although its name was mentioned in the information sheets). There was no evidence for an effect on response of using ‘higher’ university sponsorship (OR 0.96; 95% CI 0.63 to 1.45) (Analysis 145.1).

Two trials (720 participants) evaluated the effect of sending the e‐questionnaire from a male compared to a female investigator. The odds of response decreased by over a half when the e‐questionnaire was from a male investigator (OR 0.55; 95% CI 0.38 to 0.80) (Analysis 150.3). Six trials (28,162 participants) evaluated the effect on response when e‐questionnaires were sent or signed by a more senior or well‐known person. There was no evidence for an effect on response when a more senior or well‐known person sent or signed the e‐questionnaire (OR 1.09; 95% CI 0.96 to 1.25). There was moderate heterogeneity amongst the trial results (I^2^ = 41%; I^2^ = 69%) (Analysis 147.4).

#### Communication ‐ What are participants told? (Strategies 151‐178)

##### Postal

One trial (25,000 participants) evaluated the effect on questionnaire response of providing participants with an assurance of confidentiality. The odds of response were increased by more than a quarter with an assurance of confidentiality (OR 1.33; 95% CI 1.24 to 1.42) (Analysis 151.1). One trial (468 participants) evaluated the effect on questionnaire response of including a statement that others had responded to. There was no evidence for an effect on response when the statement was included (OR 1.12; 95% CI 0.76 to 1.65) (Analysis 152.2). Five trials (5544 participants) evaluated the effect on questionnaire response of offering participants the choice to opt out of the study. There was no evidence for an effect on response when participants could opt out (OR 0.96; 95% CI 0.74 to 1.25). There was considerable heterogeneity between the trial results (I^2^ = 80%) (Analysis 153.2).

A single trial (2000 participants) evaluated the effect on response of providing instructions for completion of the questionnaire. There was no evidence for an effect on response when instructions were given (OR 0.89; 95% CI 0.74 to 1.06) (Analysis 154.1). Six trials (5661 participants) evaluated the effect on response of giving participants a deadline by which to respond. There was no evidence for an effect on response of giving a deadline (OR 1.00; 95% CI 0.84 to 1.19). There was moderate heterogeneity between the trial results (I^2^ = 48%) (Analysis 155.2). Three trials (600 participants) evaluated the effect on response of mention of an obligation to respond compared to no mention of an obligation to respond. The odds of response increased by more than half with the mention of an obligation to respond (OR 1.61; 95% CI 1.16 to 2.22) (Analysis 156.2).

One trial (702 participants) evaluated the effect on response of questionnaires including a request for a telephone number. There was no evidence for an effect on response of requesting a telephone number (OR 1.00; 95% CI 0.65 to 1.54) (Analysis 157.2). One trial (200 participants) evaluated the effect of asking participants to respond on the questionnaire itself compared to asking them to respond on a separate form. There was no evidence for an effect on response of asking the participants to respond on the questionnaire rather than on a separate form (OR 1.13; 95% CI 0.57 to 2.27) (Analysis 158.2).

Seven trials (7053 participants) evaluated the effect on questionnaire response of telling participants that they would be contacted again if they did not respond. There was no evidence for an effect on response if mention of follow‐up was used (OR 1.02; 95% CI 0.91 to 1.15) (Analysis 159.2). Two trials (1907 participants) evaluated the effect on questionnaire response of requesting an explanation for non‐participation. There was no evidence for an effect on response of requesting an explanation for non‐participation (OR 1.14; 95% CI 0.83 to 1.57). There was moderate heterogeneity amongst the trial results (I^2^ = 62%) (Analysis 160.2). One trial (600 participants) evaluated the effect on response of providing a time estimate for completion of the questionnaire. There was no evidence for an effect on response when a time estimation was provided (OR 1.10; 95% CI 0.76 to 1.58) (Analysis 161.2).

One trial (500 participants) evaluated the effect on response of a detailed cover letter compared to a brief cover letter. There was no evidence for an effect on response by using the detailed cover letter (OR 1.08; 95% CI 0.74 to 1.58) (Analysis 162.2). Two trials (1251 participants) evaluated the effect on response of the presence of an appeal or a pleading factor in the cover letter. There was no evidence for an effect on response of using an appeal (OR 1.06; 95% CI 0.79 to 1.42) (Analysis 163.1). One trial (100 participants) evaluated the effect of a note requesting participants not to remove an ID Code. The odds of response decreased by more than a half when the note was added (OR 0.37; 95% CI 0.14 to 0.96) (Analysis 164.2).

A single trial (201 participants) evaluated the effect on response of a request for the participant’s signature. There was no evidence of an effect on response when participants’ signatures were requested (OR 1.19; 95% CI 0.65 to 2.18) (Analysis 165.1). Another trial (395 participants) evaluated the effect of endorsing the questionnaire by eminent professionals in the field. The odds of response decreased by more than a quarter when an endorsement was used (OR 0.63; 95% CI 0.43 to 0.94) (Analysis 166.2). One trial (671 participants) evaluated the effect of a veiled threat in follow‐up letters. The odds of response doubled when a veiled threat was used (OR 2.09; 95% CI 1.49 to 2.93) (Analysis 167.2). Eight trials (10,908 participants) evaluated the effect on questionnaire response of stressing how response would benefit the sponsor. There was no evidence for an effect on response when stressing the benefits to the sponsor (OR 0.99; 95% CI 0.86 to 1.13). There was moderate heterogeneity amongst the trial results (I^2^ = 56%) and both Begg’s and Egger’s tests indicated evidence of selection bias (Analysis 168.2). Ten trials (15,159 participants) evaluated the effect on questionnaire response of stressing how response would benefit the participant. There was no evidence for an effect on response when stressing the benefits to participants (OR 1.00; 95% CI 0.85 to 1.17). There was considerable heterogeneity amongst the trial results (I^2^ = 78%) (Analysis 169.2).

Fourteen trials (36,107 participants) evaluated the effect on questionnaire response of stressing how response would benefit society. There was no evidence for an effect on response of stressing the benefits to society (OR 1.07; 95% CI 0.95 to 1.20). There was considerable heterogeneity between trial results (I^2^ = 72%) and both Begg’s and Egger’s tests indicated evidence of selection bias (Analysis 170.2). Two trials (2070 participants) evaluated the effect on response of questionnaires remaining anonymous compared with being identifiable. There was no evidence for an effect on response of questionnaires remaining anonymous (OR 0.96; 95% CI 0.66 to 1.39). There was considerable heterogeneity between the trial results (I^2^ = 72%) (Analysis 171.1).

Two trials (27,119 participants) evaluated the effect on response of using a cover letter that highlighted salience. There was no evidence for an effect on response of a letter that highlighted salience (OR 1.06; 95% CI 0.75 to 1.50). There was considerable heterogeneity between the trial results (I^2^ = 75%) (Analysis 172.2). A single trial (2180 participants) evaluated the effect on response by using a cover letter that highlighted salience in the first mailing compared with using one during follow‐up. The odds of response when using a cover letter that highlights salience in the first mailing were over twice the odds of response when one was used during follow‐up (OR 2.49; 95% CI 1.82 to 3.40) (Analysis 173.3).

One trial (4447 participants) evaluated the effect on questionnaire response of informing participants that their responses were being monitored. The odds of response were increased by more than a tenth when the letter stated that responses were being monitored (OR 1.15; 95% CI 1.01 to 1.31) (Analysis 174.2). A single trial (1418 participants) evaluated the effect on response of using a cover letter that emphasised harm prevention compared with one that emphasised health promotion. There was no evidence of an effect on response when the letter emphasised harm prevention (OR 1.19; 95% CI 0.83 to 1.72) (Analysis 175.2). The same trial evaluated the effect on response of using a cover letter that emphasised harm prevention compared with one that contained a neutral message. The odds of response were increased by more than two‐fifths when the letter emphasised harm prevention (OR 1.44; 95% CI 0.98 to 2.12) (Analysis 176.2).

One trial (1192 participants) evaluated the effect on questionnaire response by sending a letter with behaviour change techniques in the text. The odds of response were increased by more than a quarter using the letter with behaviour change techniques in the text (OR 1.39; 95% CI 1.08 to 1.77) (Analysis 177.1). A single trial (1316 participants) evaluated the effect on response of using a culturally sensitive cover letter. There was no evidence for an effect on response using a culturally sensitive cover letter (OR 1.00; 95% CI 0.74 to 1.34) (Analysis 179.1).

##### Electronic

Three trials (23,777 participants) evaluated the effect on e‐questionnaire response by including a statement that others had responded to. There was no evidence for an effect on response when the statement was included (OR 1.14; 95% CI 0.83 to 1.56). There was considerable heterogeneity between the trial results (I^2^ = 94%) (Analysis 152.4).

A single trial (8586 participants) evaluated the effect on e‐questionnaire response of giving participants a deadline by which to respond. The odds of response increased by over a tenth when given a deadline (OR 1.18; 95% CI 1.03 to 1.34) (Analysis 155.4). Three trials (3536 participants) evaluated the effect on e‐questionnaire response by stressing how responses would benefit society. There was no evidence for an effect on response when stressing the benefits to society (OR 1.35; 95% CI 0.95 to 1.91). There was moderate heterogeneity between trial results (I^2^ = 41%) (Analysis 170.3). Four trials (5915 participants) evaluated the effect of including an appeal, such as “request for help” in the subject line of the email. There was no evidence of an effect on response by including an appeal in the subject line (OR 1.07; 95% CI 0.79 to 1.47). There was considerable heterogeneity between the trial results (I^2^ = 71%) (Analysis 163.3).

A single trial (1250 participants) evaluated the effect on e‐questionnaire response of a detailed letter compared to a brief letter. The odds of response were over three times greater when using the brief letter (OR 3.26; 95% CI 1.79 to 5.94) (Analysis 162.3). One trial (2358 participants) evaluated the effect on response of telling participants that the e‐questionnaire would take 10 minutes to complete compared with telling them that it would take 30 minutes. There was no evidence for an effect on response by giving a longer time estimate (OR 1.25; 95% CI 0.96 to 1.64) (Analysis 178.2).

#### Length ‐ How long is the questionnaire? (Strategies 183‐87)

##### Postal

Seventy‐two trials (84,954 participants), including two unpublished trials, evaluated the effect of questionnaire length on response. The odds of response increased by more than half using shorter questionnaires (OR 1.58; 95% CI 1.40 to 1.78). Heterogeneity amongst trial results was apparent on inspection of the forest plot, and in the Chi2 test result (P < 0.00001) and I^2^ result (93%) (Analysis 183.2). One trial (600 participants) evaluated the effect on questionnaire response of using a double postcard compared to one page. The odds of response decreased by half when a double postcard was used (OR 0.47; 95% CI 0.34 to 0.66) (Analysis 184.2). A single trial (1795 participants) evaluated the effect of sending the questionnaire with a supplement compared to sending the questionnaire alone. There was no evidence for an effect on response of sending a questionnaire with a supplement (OR 0.86; 95% CI 0.70 to 1.07) (Analysis 185.1). Two trials (4943 participants) evaluated the effect on response of including a questionnaire for relatives. The odds of response were reduced by one‐third when a questionnaire for relatives was included (OR 0.67; 95% CI 0.60 to 0.76) (Analysis 186.1). One trial (414 participants) evaluated the effect of including a consent form with the questionnaire. There was no evidence for an effect on response of including a consent form (OR 1.32; 95% CI 0.89 to 1.95) (Analysis 187.2).

##### Electronic

Five trials (12,325 participants) evaluated the effect of the length of electronic questionnaires on response. The odds of response increased by half when using shorter e‐questionnaires (OR 1.51; 95% CI 1.06 to 2.16). There was considerable heterogeneity amongst the trial results (I^2^ = 94%) (Analysis 183.4).

## Discussion

### Summary of main results

This updated review identified a total of 758 eligible studies that evaluated 187 strategies to increase response to postal and electronic questionnaires, adding 245 new trials to the 513 studies included in the previously published version (Edwards 2009). We found substantial heterogeneity amongst trial results in half of the strategies.

The findings relevant to increasing questionnaire response include: contacting people before they are sent the questionnaire, sending postal questionnaires by first‐class post or by a special (recorded) delivery service, and providing a stamped‐return envelope. Questionnaires, letters, and emails can be made more personal, and kept short; incentives can be offered with a postal questionnaire, for example, a small amount of money, or a non‐monetary incentive such as a pen; one or more reminders can be sent with a copy of the questionnaire to people who do not reply; response to postal questionnaires can also be increased if they originate from a university. Using postal rather than electronic questionnaires or providing people with a choice of response modes (electronic or postal) can increase response. Response to an electronic questionnaire can be increased if it is administered over a computer rather than a smartphone. Monetary and non‐monetary incentives can also help to increase response to electronic questionnaires.

We have chosen to use odds ratios in our analyses for methodological reasons. However, the practical implication of the odds ratio for a strategy is difficult to interpret without knowing the baseline response rate (without the strategy). Moreover, the odds ratio for a strategy might vary in relationship to the baseline response rate. Therefore, those conducting postal and electronic surveys should scrutinise the data in the relevant results tables closely if the magnitude of the effect that they might expect from using a specific strategy is an important consideration for them in deciding whether to use the strategy. A table showing the conversion of odds ratios to response proportions for a range of different baselines is included in Appendix 2.

#### Summary of evidence since last published version

Many of the 245 new trials added to this review update evaluated previously identified strategies (e.g. incentives, length, and personalisation). Many of the effect estimates of strategies to increase questionnaire response presented in this update are similar to those reported in the last published version of the review (Edwards 2009) but are now more precise (i.e. confidence intervals are narrower now than before). In a few cases, the addition of new trials changed our conclusions: for example, there is now some evidence for an effect on postal questionnaire response by using a larger non‐monetary incentive; also, there is now evidence that monetary incentives increase electronic questionnaire response. Strategies that emerged in this update that were not reported previously are: using postal rather than electronic questionnaires and providing people with a choice of response modes (electronic or postal) can increase response.

### Overall completeness and applicability of evidence

We found 670 eligible trials with postal questionnaires that evaluated over 100 different ways of increasing response and 88 eligible trials with electronic questionnaires that evaluated over 30 different ways of increasing response. The types of participants in these trials include a wide range of people likely to be asked to complete a questionnaire, from clinical trial participants, patients and healthcare providers, university students and faculty, to marketing managers, industrial accountants, microwave oven owners, and grocery store managers. All trials reported the required outcomes: the proportions of participants responding to the first or final mailings of a postal questionnaire, and the proportions of participants logging‐in, clicking a hyperlink, or submitting an online questionnaire.

Inadequate allocation concealment can bias the results of clinical trials (Schulz 1995). In our review, information on allocation concealment was unavailable for most of the included trials. If they were inadequately concealed, this may have biased the results. Blinding of outcome assessors reduces detection bias (Higgins 2022). However, in the eligible trials in our review update, no outcomes were assessed because we were only interested in the counts of responses in the experimental and control groups, and so there was little or no risk of detection bias in this review.

### Quality of the evidence

As all the included studies were randomised trials, the overall quality of the body of evidence presented in this review is ‘high’. However, we found considerable statistical heterogeneity amongst trial results in half of the strategies, and for these, the pooled odds ratios may not be meaningful. Variation between trial interventions and populations is likely to explain some of the heterogeneity. For example, amongst trials evaluating non‐monetary incentives, the types of incentives used are considerably heterogeneous, including things such as donations to charity, lottery participation, and a free key‐ring or pen. Amongst trials evaluating monetary incentives, the amounts of money offered to participants varied between trials. A meta‐regression analysis has shown that monetary incentives can increase response to postal questionnaires but that the relationship between the amount of money and response is not linear (Edwards 2005). Amongst the trials evaluating shorter and longer questionnaires, the length of the questionnaires used varied between trials, some comparing a single page with a two‐page alternative, and others comparing four or more pages with longer alternatives. In a meta‐regression analysis, most of the heterogeneity in trial results was explained by variation in the length of the questionnaires used in each trial (Edwards 2004). A subgroup analysis of the trials of personalisation in postal questionnaires found that response was increased by addressing participants by name on cover letters, and that the effect appeared to be enhanced by including a handwritten signature (Scott 2006). Due to the remaining unexplained heterogeneity in other strategies, we downgraded the overall quality rating of the body of evidence presented in this review to ‘moderate’.

### Potential biases in the review process

The identification and inclusion of all relevant trials in systematic reviews reduces random error in meta‐analyses and, because ease of identification of trials is associated with intervention effects, complete ascertainment may also reduce bias (Clarke 1994). We excluded some trials because we could not confirm that participants had been randomly allocated to intervention and control groups, and we have not examined whether the results of these trials differed systematically from the included trials. Although tests for selection bias were significant in fifteen strategies, these results may be due to true heterogeneity between trial results, rather than bias in the selection of trials (Egger 1997).

### Agreements and disagreements with other studies or reviews

Two other systematic reviews and one meta‐analysis of methods to increase questionnaire response have appeared in the survey research literature during the 30 years prior to this review.

The largest of these (Yammarino 1991) included 115 studies published between 1940 and 1988. It also found evidence that: repeated contacts (preliminary notification and follow‐up), appeals in letters, inclusion of a return envelope, types of postage, monetary incentives (particularly $0.50 or less), and shorter questionnaires increased response. However, it did not find evidence that either sponsorship or non‐monetary incentives increased response. It is unclear in this meta‐analysis whether only RCTs were included, which, in addition to the smaller number of included studies, may explain why its findings differ from those of our review.

The next largest (Price 2022) included 40 randomised trials of patient experience surveys only, conducted in the US. It presented a descriptive account of the included studies with no meta‐analysis. As in our review, it concluded the following: that pre‐notification, special delivery, and monetary incentives (particularly unconditional ones) increased response; it also found evidence that questionnaires administered using web‐based modes only resulted in lower response rates than those administered by mail. Unlike our review, however, it was uncertain about any effects of questionnaire length on response.

The third (Nakash 2006) included 13 randomised trials of healthcare studies on only patient populations. As in our review, it found evidence that follow‐up (particularly more intense follow‐up), and shorter questionnaires increased response. Unlike our review, however, it found no evidence for any effects of incentives on response. The reason for this different result may be that it only included four trials of non‐monetary incentives. Our review includes 146 trials of non‐monetary incentives and shows that the odds of response can be increased by over a tenth when a non‐monetary incentive is used.

The most likely reason for differences in the findings of our review with those of the reviews described above is the huge difference in the number of studies included.

## Authors' conclusions

**Implication for systematic reviews and evaluations of healthcare**

Researchers can increase response to postal and electronic questionnaires by using the strategies shown to be effective in this systematic review. Some strategies will require additional materials or administrative time whereas others can be implemented at little or no extra cost. For example, researchers may be able to double the odds of response by offering participants payment for completion of questionnaires or by using recorded delivery, both of which will add substantially to costs for large studies. The use of non‐monetary incentives, on the other hand, may be more affordable, but is likely to be less effective in encouraging response.

**Implication for methodological research**

Further analyses (for example, using random‐effects meta‐regressions) may reveal important sources of variation, for example, due to methodological quality, questionnaire topic, the years in which each study was done, or types of population. In this review, our aim was to systematically identify and critically appraise eligible trials, and to present the relevant data. We did not intend to produce single effect estimates for every strategy. For many strategies, although there was statistical heterogeneity, the directions of the effects were similar. For these strategies, we cannot be sure about the size of the effect, but we can be reasonably confident that there was an effect on response.

The results of this review show that questionnaire length has a substantial impact on non‐response, particularly when questionnaires are very short. In the context of outcome data collection in a clinical trial, the use of a short questionnaire would be expected to minimise non‐response, thus increasing the effective sample size and reducing sampling error. However, if the use of short questionnaires reduces the accuracy of the measurement process, the reduction in random error achieved by increased follow‐up would have to be traded‐off against increased random error due to using less precise measurements. Further research is underway by the authors to quantify this trade‐off, so that outcome measures can be designed for use in clinical trials that minimise total random error (sampling error and measurement error).

This review examined the effectiveness of 187 different strategies to increase the response to postal and electronic questionnaires. The outcome of interest in this review was the overall response proportion, and we did not examine the impact of factors that may influence the completeness of the returned questionnaires. However, factors that influence the readability of questionnaires, such as the number of syllables per word, words per sentence, typeface and font size may have an important effect on both the proportion of questions that are answered and indeed the overall response proportion.

One‐third of clinical trials, case‐control and cohort studies collect data from participants using a questionnaire, and more than a quarter collect data using interviews with participants (Van Gelder 2010). If those who are eligible for a study or those who agree to take part do not respond to these questionnaires or refuse to take part in interviews, this will reduce study power and may introduce bias in the results that makes them misleading and useless. Good evidence exists in this review for some methods that might be used to increase response to self‐completed questionnaires, and this evidence has been used to achieve over 90% data completion in some biomedical research studies (Butler 2013; Edwards 2005b; Free 2011). However, less is known about effective methods to increase participation in interviews, and we plan to conduct a Cochrane Methodology Review to address this gap in the evidence.

## What's new

**Table:** 

**Date**	**Event**	**Description**
22 December 2021	New search has been performed	Third update of the review (new search December 2021).
22 December 2021	New citation required but conclusions have not changed	This is the third update of the review (new search December 2021). The current update now includes 670 eligible trials that evaluated over 100 different strategies for increasing response to postal questionnaires as well as 88 eligible trials that evaluated over 30 different strategies for increasing response to electronic questionnaires. There has been a change in authorship, with two new authors having been added and five previous authors agreeing to be acknowledged in this updated version. An important methodological finding in this update is that the response rate is increased using postal rather than electronic questionnaires (Analysis 81.1; Analysis 81.2).

## History

Protocol first published: Issue 2, 1999 Review first published: Issue 3, 2001

**Table:** 

**Date**	**Event**	**Description**
12 May 2009	New citation required but conclusions have not changed	The current update includes randomised controlled trials of questionnaires distributed by electronic mail, and strategies designed to improve response to online or web surveys.
10 December 2008	New search has been performed	This review has been updated (new search December 2007). The current update includes 481 eligible trials that evaluated 110 different strategies for increasing response to postal questionnaires and 32 eligible trials that evaluated 27 different strategies for increasing response to electronic questionnaires. A new search was re‐run February 2009 in MEDLINE and Psychinfo and 23 possibly eligible trials are listed under Studies awaiting classification.
27 December 2007	Amended	Converted to new review format.

## Appendices

### Appendix 1. Search strategies

#### Original review

Search strategies were developed for use in a range of electronic bibliographic databases in Edwards 2003.

##### Database time period or version

Cochrane Controlled Trials Register 1999.3 CINAHL 1982 ‐ 1999.07 ERIC 1982 ‐ 1998.09 PsycLit 1887 ‐ 1999.09 Dissertation Abstracts 1861 ‐ 1999.08 MEDLINE 1966 ‐ 1999 EMBASE 1980 ‐ 1999.08

A. questionnair* or survey* or data collection B. respon* or return* C. remind* or letter* or postcard* or incentiv* or reward* or money* or monetary or payment* or lottery or raffle or prize or personalis* or sponsor* or anonym* or length or style* or format or appearance or color or colour or stationery or envelope or stamp* or postage or certified or registered or telephon* or telefon* or notice or dispatch* or deliver* or deadline or sensitive D. control* or randomi* or blind* or mask* or trial* or compar* or experiment* or "exp" or factorial E. A and B and C and D

Social Science Citation Index 1981 ‐ 1999 Science Citation Indes 1981 ‐ 1999 [(survey* or questionnair*) and (return* or respon*)]

Social Psychological Educational Criminological Trials Register (SPECTR) 1950 ‐ 1998 EconLit 1969 ‐ 2000 Sociological Abstracts 1963 ‐ 2000

((survey$ or questionn$) and (return$ or respon$)).ti or ((survey$ or questionn$) and (mail$ or post$)).ti or ((return$ or respon$) and (mail$ or post$)).ti

Index to Scientific & Technical Proceedings 1982 ‐ 2000

((survey*, questionn*)+(return*,respon*))@TI, ((return*,respon*)+(mail,mailed,postal))@TI, ((survey*,questionn*)+(mail,mailed,postal))@TI

National Research Register (Web version): 2000.1

((survey*:ti or questionn*:ti) and (return*:ti or respon*:ti)) or ((return*:ti or respon*:ti) and (mail:ti or mailed:ti or postal:ti)) or ((survey*:ti or questionn*:ti) and (mail:ti or mailed:ti or postal:ti))

The following literature reviews and meta‐analyses were inspected for eligible trials:

- Armstrong JS. Monetary incentives in mail surveys. Public Opinion Quarterly 1975;39:111‐6.
- Armstrong S. Return postage in mail surveys: a meta analysis. Public Opinion Quarterly 1987;51:233‐48.
- Bogen K. The effects of questionnaire length on response rates ‐ a review of the literature. American Statistical Association 1996;1020‐5.
- Boser JA. Reviewing the research on mail survey response rates: descriptive study. Paper presented at the annual meeting of the American Educational Research Association New York. April 1996.
- Boser JA. Factors influencing mail survey response rates: What do we really know? Paper presented at the Annual meeting of the Mid‐South Educational Research Association. November 1995.
- Brehm J. Stubbing our toes for a foot in the door? Prior contact, incentives and survey response. International Journal of Public Opinion Research 1994;6(1):45‐63.
- Church AH. Estimating the effect of incentives on mail survey response rates: a meta‐analysis. Public Opinion Quarterly 1993;57:62‐79.
- Cox WE. Response patterns to mail surveys. Journal of Marketing Research 1966;3:392‐7.
- Downs PE. Recent evidence on the relationship between anonymity and response variables for mail surveys. Journal of the Academy of Marketing Science 1986;14(1):72‐82.
- Duncan WJ. Mail questionnaires in survey research: a review of response inducement techniques. Journal of Management 1979;5(1):39‐55.
- Erdos PL. Visible vs. disguised keying on questionnaires. Journal of Advertising Research 1977;17(1):13‐8.
- Fox RJ. Mail survey response rate. A meta‐analysis of selected techniques for inducing response. Public Opinion Quarterly 1988;52:467‐91.
- Francel EG. Mail‐administered questionnaires: a success story. Journal of Marketing Research 1966;3:89‐92.
- Goyder JC. Further evidence on factors affecting response rates to mailed questionnaires. American Sociological Review 1982;47:550‐3.
- Green KE. Reviewing the research on mail survey response rates: a meta‐analysis. Paper presented at the Annual Meeting of the American Educational Research Association. April 1996.
- Greenwald HP. Issues in survey data on medical practice: some empirical comparisons. Public Health Reports 1986;101(5):514‐46.
- Guffey H. Stamps versus postal permits: a decisional guide for return postage in mail questionnaires. Journal of Academy of Marketing Science 1980;8(3): 234‐42.
- Harvey L. Factors affecting response rates to mailed questionnaires: a comprehensive literature review. Journal of the Market Research Society 1987;29:341‐53.
- Heberlein TA. Factors affecting response rates to mailed questionnaires. American Sociological Review 1978;43(4):447‐62.
- Hopkins KD. Response rates in survey research: a meta‐analysis of the effects of monetary gratuities. Journal of Experimental Education 1992;61:52‐62.
- Houston MJ. Broadening the scope of methodological research on mail surveys. Journal of Marketing Research 1976;13:397‐403.
- Jobber D. Improving response rates in industrial mail surveys. Industrial Marketing Management 1986;15:183‐95.
- Jobber D. Modelling the effects of prepaid monetary incentives on mail survey response. Journal of the Operational Research Society 1988;39:365‐72.
- Jobber D. Questionnaire factors and mail survey response rates. European Research. 1985;(July)124‐9.
- Jobber D. Maximizing response rates in industrial mail surveys: a review of the evidence. Advances in Business Marketing 1990;4:121‐46.
- Kanuk L. Mail surveys and response rates: a literature review. Journal of Marketing Research 1975;12:440‐53.
- King FW. Anonymous versus identifiable questionnaires in drug usage surveys. American Psychologist 1970;25:982‐5.
- Leslie L. Increasing response rates to long questionnaires. Journal of Educational Research 1970;63:347‐50.
- Linsky AS. Stimulating responses to mailed questionnaires: a review. Public Opinion Quarterly 1975;39:82‐101.
- Mayer EN. Postage stamps do affect results of your mailing. Printers' Ink 1946;217:91.
- Nowack KM. Getting them out and getting them back. Training Development Journal 1990;(April)82‐5.
- Ransdell LB. Maximising response rate in questionnaire research. American Journal of Health Behaviour 1996;20:50‐6.
- Robin S. A procedure for securing returns to mail questionnaires. Sociology and Social Research 1965;50:24‐35
- Roth PL. Response rates in HRM/OB survey research: norms and correlates, 1990‐1994. Journal of Management 1998;24:97‐117.
- Schlegelmilch BB. Prenotification and mail survey response rates: a quantitative integration of the literature. Journal of the Market Research Society 1991;33(3):243‐55.
- Scott C. Research on mail surveys. Journal of the Royal Statistical Society 1961;124:143‐205.
- Singer E. Confidentiality assurances and response: a quantitative review of the experimental literature. Public Opinion Quarterly 1995;59: 66‐77.
- Vaux A. Conducting mail surveys. Psychology Research Handbook. 1996:(Chapter 10).
- Wiseman F. A reassessment of the effects of personalization on response patterns in mail wurveys. Journal of Marketing Research 1976;3I:110‐1.
- Yammarino FJ. Understanding mail survey response behaviour: a meta‐analysis. Public Opinion Quarterly 1991;55: 613‐39.
- Young JM. Improving survey response rates: a meta‐analysis of the effectiveness of an advance telephone prompt from a medical peer. Medical Journal of Australia 1999;170: 339.
- Yu J. A quantitative review of research design effects on response rates to questionnaires. Journal of Marketing Research 1983;XX:36‐44.
- Zelnio RN. Data collection techniques: mail questionnaires. American Journal of Hospital Pharmacy 1980;37:1113‐9.

The following journals were searched by hand:

- Public Opinion Quarterly 1960 to 1998;
- American Journal of Epidemiology 1948 to 1999.

##### Reliability of screening for eligible trials

The electronic bibliographic searches outlined above yielded several thousand records of potentially relevant reports that were then screened to determine eligibility. Because exclusion of reports during screening would mean that they would not be considered again, we assessed the accuracy and reliability of screening for relevant trials using the records retrieved by a search of ten databases.

A search of ten electronic bibliographic databases yielded 26,937 records of potentially relevant reports that were downloaded into a ProCite database. After removing duplicate records, there were 22,571 records of potentially relevant reports. These records were divided into six approximately equal sets (A to F) and each of four reviewers was allocated three of the sets to screen. The six sets were allocated such that two reviewers examined each record, and identification of trials by each reviewer could be compared with each of the other reviewers. Agreement between reviewers was assessed using Cohen's kappa statistic (k) which adjusts the proportion of records in which there was agreement between reviewers by the amount of agreement that is expected by chance alone. Ascertainment intersection (capture‐recapture) methods (Hook 1992) were then used to estimate the likely number of relevant records missed by all four reviewers. When screening was complete, full copies of the reports identified by at least one reviewer as potentially relevant were requested. Each report obtained was assessed independently by two reviewers for eligibility for inclusion in the systematic review. Disagreements about eligibility were referred to a third reviewer. Eligible reports were used as the 'gold standard' against which an assessment was made about the accuracy of screening by reviewers.

After screening, 301 of 22,571 records were identified by at least one reviewer as potentially relevant. Of the six possible comparisons between reviewers, kappa coefficients of agreement ranged from 0.59 (95% CI 0.56 to 0.62) to 0.93 (95% CI 0.90 to 0.96). Agreement was 'almost perfect' (k > 0.81) between two pairs, 'substantial' (k > 0.61) between three pairs, and 'moderate' (k > 0.41) between one pair. Ascertainment intersection methods suggest that, on average, pairs of reviewers missed 4% (range 0% to 6%) of potentially relevant records. In contrast, single reviewers missed on average 22% (range 3% to 55%). Twenty‐eight reports were not available by the time of the ascertainment intersection analysis. Of the 273 reports that were available, 156 (57%) met the inclusion criteria for the systematic review. Ascertainment intersection methods estimated that pairs of reviewers had missed very few eligible records (0 records missed, 95% CI 0 to 3 records). In the light of these results, we believe that very few eligible trials were inappropriately excluded during screening.

##### Sensitivity of combined search strategy

The sensitivity of the search strategy was assessed by handsearching the Public Opinion Quarterly and comparing the trials identified by handsearching with those identified by the combined search strategy. Of the 40 eligible trials identified by hand searching, 15 trials had been identified from electronic bibliographic databases and 23 had been identified from the reference lists of identified trials and relevant meta‐analyses. Two studies identified by handsearching were not identified by any part of the combined search strategy. On the basis of these results, electronic bibliographic database searching had a sensitivity of 38% (15/40), searching reference lists of identified trials and relevant meta‐analyses had a sensitivity of 58% (23/40), and the combined search strategy had a sensitivity of 95% (38/40), (95% CI 84% to 99%).

** Notes on study selection: The review authors did not record the number of records/references excluded or reasons for their exclusion at the full‐text stage during the development of*Edwards 2003*. There were 372 included studies and 40 studies awaiting classification.*

#### First review update

In Edwards 2007, the following databases were searched again using the appropriate strategies detailed above.

##### Database time period or version

Cochrane Controlled Trials Register 2002.4 CINAHL 1999.07 ‐ 2003.02 ERIC 1998.09 ‐ 2003.01 PsycLit 1999.09 ‐ 2003.02 Dissertation Abstracts 1999.08 ‐ 2003.02 MEDLINE 1999 ‐ 2003 EMBASE 1999.08 ‐ 2003.02 Science Citation Index 1999 ‐ 2003 Social Science Citation Index 1999 ‐ 2003 Social Psychological Educational Criminological Trials Register (SPECTR) 1998 ‐ 2003 EconLit 2000 ‐ 2003.01 Sociological Abstracts 2000 ‐ 2002.12 Index to Scientific & Technical Proceedings 2000 ‐ 2003 National Research Register (Web version): 2003.2

A search of these databases yielded 6423 records of potentially relevant reports that were downloaded into a ProCite database. Two reviewers examined each record so that identification of trials by each reviewer could be compared. After screening, 194 of 6423 records were identified by at least one reviewer as potentially relevant.

During the update, attempts were made to obtain sufficient information on studies awaiting assessment to be able to include or exclude them from the review. This included writing to or emailing the authors of all potentially eligible trials and those in studies awaiting assessment.

** Notes on study selection: The review authors did not record the number of records/references excluded (with reasons for exclusion) at the full‐text stage during the development of*Edwards 2007*. This first update version included 372 trials. There were 40 studies awaiting classification.*

#### Second review update

In Edwards 2009, the following databases were searched again using the appropriate strategies detailed above. The search also included electronic‐based questionnaires such as those sent via email, and online surveys.

Cochrane Library Online Issue 4 2007 CENTRAL Cochrane Library Online Issue 4 2007 Methodology studies (CMR) CINAHL 2003 ‐ 2007.12 ERIC 2003 ‐ 2007.12 PsycINFO 2003 ‐ 2008.01 MEDLINE 2003 ‐ 2007.11 EMBASE 2003 ‐ 2007.10 Science Citation Index 2003 ‐ 2008.01 Social Science Citation Index 2003 ‐ 2008.01 Social Psychological Educational Criminological Trials Register (SPECTR) 2003 ‐ 2007.12 EconLit 2003 ‐ 2007.12 Sociological Abstracts 2003 ‐ 2007.12 Dissertation & Theses 2003 ‐ 2008.01 Index to Scientific & Technical Proceedings 2003 ‐ 2008.01 National Research Register (Web version): 2008.02

A search of these databases yielded 19,826 records of potentially relevant reports that were downloaded into an EndNote database. After removing duplicates, we identified 14,792 records. Two reviewers examined each record so that identification of trials by each reviewer could be compared. After screening, 253 of 14,792 records were identified by at least one reviewer as potentially relevant and their full texts were sought.

During the update, attempts were made to obtain sufficient information on studies awaiting assessment to be able to include or exclude them from the review. This included writing to or emailing the authors of all potentially eligible trials and those studies awaiting assessment.

** Notes on study selection: The review authors did not record the number of records/references excluded (with reasons for exclusion) at the full‐text stage during the development of*Edwards 2009*. This second review update included 513 trials. There were 23 studies awaiting classification.*

#### Third review update (current version)

In 2021, an Information Specialist advised us of some adjustments to improve our search strategy. We reviewed the terms used to search each of the databases and included the additional use of Medical Subject Headings (MeSH) and Emtree terms in relevant databases, which were exploded or expanded appropriately to capture as many relevant articles as possible.

We added the RCT search filters developed by the Cochrane Collaboration, including the Cochrane Highly Sensitive Search Strategies for identifying randomised trials in MEDLINE (sensitivity‐maximising version) and controlled trials in Embase.

We used proximity searching and increased truncation of terms to increase search sensitivity while maintaining precision; searches of databases in title only were removed from the search strategy, and unnecessary restrictions were removed (e.g. MEDLINE searches were expanded to include all possible methods to increase questionnaire response by the removal of Part C of the original search strategy which had restricted results to known methods only).

Several changes to databases also occurred since the second update, so further changes were necessary: out‐of‐date sources were removed; C2‐SPECTR, Scientific & Technical Proceedings were replaced with Web of Science; trial register searches were expanded to include the Clinical Trials Register and the WHO International Clinical Trial Registry Platform. We also added databases to increase the breadth of the search: Global Index Medicus database from WHO (specialising in research from the global south) and Scopus.

Example search terms:

Ovid MEDLINE(R) ALL <1946 to August 13, 2021>

1 randomized controlled trial.pt.

2 controlled clinical trial.pt.

3 randomized.ab.

4 placebo.ab.

5 clinical trials as topic.sh.

6 randomly.ab.

7 trial.ti.

8 or/1‐7

9 ((questionnair* or survey* or (data adj1 collect*)) adj4 (respon* or return* or participat* or completion)).ti,ab,kf.

10 "Surveys and Questionnaires"/ or Data Collection/

11 exp Community Participation/sn [Statistics & Numerical Data]

12 9 or (10 and 11)

13 8 and 12

Notes:

- Lines 1‐8 are the Cochrane Highly Sensitive Search Strategy for identifying randomised trials in MEDLINE: sensitivity‐ and precision‐maximising version.
- Line 9 uses proximity searching to look for terms for questionnaires within 4 words of terms for response etc.
- Lines 10 and 11 use MeSH terms to look for papers on specific subjects.
- Line 12 combines the topic searches together.
- Line 12 finds RCTs on the topic.

### Appendix 2. Conversion of odds ratios to response rates from different baseline rates

**Table:**

	**Odds ratio** **0.50**	**0.75**	**1.00**	**1.25**	**1.50**	**1.75**	**2.00**	**2.25**	**2.50**	**2.75**	**3.00**
**Baseline rate (%)** **10**	5	8	10	12	14	16	18	20	22	23	25
**20**	11	16	20	24	27	30	33	36	38	41	43
**30**	18	24	30	35	39	43	46	49	52	54	56
**40**	25	33	40	45	50	54	57	60	63	65	67
**50**	33	43	50	56	60	64	67	69	71	73	75
**60**	43	53	60	65	69	72	75	77	79	80	82
**65**	48	58	65	70	74	76	79	81	82	84	85
**70**	54	64	70	74	78	80	82	84	85	87	88
**75**	60	69	75	79	82	84	86	87	88	89	90
**80**	67	75	80	83	86	88	89	90	91	92	92
**85**	74	81	85	88	89	91	92	93	93	94	94
**90**	82	87	90	92	93	94	95	95	96	96	96
**95**	90	93	95	96	97	97	97	98	98	98	98

### Appendix 3. Observations from the Stakeholder Advisory Group

During the 2021‐23 update, a Stakeholder Advisory Group was recruited to comment on the work as it progressed. Members of the group were drawn from a variety of disciplines and experience, including academic and action research, community development, public health, campaigning, communications, local government and local community services. The interests of SAG members in survey and questionnaire design were heavily oriented towards social science research and, in particular, to issues such as poverty and deprivation, health and disability, race and gender. Discussions were wide‐ranging and raised considerations that could only be partly accommodated during the review update, or not at all.

There was a high level of dissatisfaction, especially amongst members working in community settings, with externally generated questionnaire surveys, as opposed to surveys generated from within fieldwork practice or service delivery. Particular points included: mistrust of motivation or intent of contractors or commissioners; researchers with poor awareness of their own personal biases or prejudicial attitudes towards target populations; and identification of ‘Questionnaire fatigue’ as potential important influences.

Strong commitment to understanding of the demography and social circumstances of target audiences and proposed collection methods was identified as key factor in achieving an inclusive sample. For social science studies, one useful mechanism against which to assess survey structure and methodology can be cross‐reference to ‘protected characteristics’ embodied in the UK’s Equality Act. A 10^th^ characteristic of poverty and declining social mobility could also be added to this.

In addition to the many aspects of questionnaire design covered in this review which are likely to influence response rates, additional considerations were raised against which the usefulness of data collected can be assessed. These included, for example, the issue of whether language, neurodiversity, and literacy issues were accommodated. There is evidence of further exclusion of some respondent cohorts by the move to online, paperless surveys.

Failure to record extraneous factors in research study design, such as different demographics, cultural assumptions, literacy, or language differences and the extent to which more marginalised groups respond, can undermine survey outcomes. The ability to interrogate ‘intersectionality’ in results can assist in drilling down to these underlying factors. The evidence of absence as well as evidence of presence can be significant.

There is a responsibility to make dissemination accessible, and to understand the power and impact of communication strategies.

## Data and analyses

**Comparison 1:** Monetary incentive vs. no incentive

Outcome or subgroup title	No. of studies	No. of participants	Statistical method	Effect size
1.1 First response	64	72237	Odds Ratio (M‐H, Random, 95% CI)	2.16 [1.95, 2.40]
1.2 Final response	111	226209	Odds Ratio (M‐H, Random, 95% CI)	1.86 [1.73, 1.99]
1.3 e ‐ Log	3	3209	Odds Ratio (M‐H, Random, 95% CI)	1.83 [0.91, 3.69]
1.4 e ‐ Submission	5	6446	Odds Ratio (M‐H, Random, 95% CI)	1.88 [1.31, 2.71]

**Comparison 2:** Larger vs. smaller monetary incentive

Outcome or subgroup title	No. of studies	No. of participants	Statistical method	Effect size
2.1 First response	22	20722	Odds Ratio (M‐H, Random, 95% CI)	1.21 [1.11, 1.33]
2.2 Final response	50	137457	Odds Ratio (M‐H, Random, 95% CI)	1.24 [1.15, 1.33]

**Comparison 3:** Monetary vs. non‐monetary incentive

Outcome or subgroup title	No. of studies	No. of participants	Statistical method	Effect size
3.1 First response	6	9589	Odds Ratio (M‐H, Random, 95% CI)	1.85 [1.35, 2.55]
3.2 Final response	17	28212	Odds Ratio (M‐H, Random, 95% CI)	1.67 [1.47, 1.90]
3.3 e ‐ Login	1	1100	Odds Ratio (M‐H, Random, 95% CI)	0.66 [0.50, 0.87]
3.4 e ‐ Submission	3	3614	Odds Ratio (M‐H, Random, 95% CI)	0.89 [0.63, 1.26]

**Comparison 4:** Non‐monetary incentive vs. no incentive

Outcome or subgroup title	No. of studies	No. of participants	Statistical method	Effect size
4.1 First response	74	117750	Odds Ratio (M‐H, Random, 95% CI)	1.20 [1.13, 1.27]
4.2 Final response	146	277802	Odds Ratio (M‐H, Random, 95% CI)	1.16 [1.11, 1.21]
4.3 e ‐ Login	6	22565	Odds Ratio (M‐H, Random, 95% CI)	1.19 [1.10, 1.28]
4.4 e ‐ Submission	16	38901	Odds Ratio (M‐H, Random, 95% CI)	1.60 [1.25, 2.05]

**Comparison 5:** Larger non‐monetary incentive vs. smaller

Outcome or subgroup title	No. of studies	No. of participants	Statistical method	Effect size
5.1 First response	4	7570	Odds Ratio (M‐H, Random, 95% CI)	1.31 [1.15, 1.49]
5.2 Final response	11	18688	Odds Ratio (M‐H, Random, 95% CI)	1.15 [1.00, 1.33]
5.3 e ‐ Login	4	13250	Odds Ratio (M‐H, Random, 95% CI)	1.18 [1.00, 1.38]
5.4 e ‐ Submission	10	37382	Odds Ratio (M‐H, Random, 95% CI)	1.07 [0.87, 1.32]

**Comparison 6:** Unconditional incentive vs. conditional

Outcome or subgroup title	No. of studies	No. of participants	Statistical method	Effect size
6.1 First response	16	29435	Odds Ratio (M‐H, Random, 95% CI)	1.83 [1.48, 2.25]
6.2 Final response	35	48850	Odds Ratio (M‐H, Random, 95% CI)	1.53 [1.35, 1.74]
6.3 e ‐ Log	1	736	Odds Ratio (M‐H, Random, 95% CI)	0.90 [0.64, 1.27]
6.4 e ‐ Submission	3	1401	Odds Ratio (M‐H, Random, 95% CI)	1.08 [0.77, 1.50]

**Comparison 7:** Incentive with first vs. subsequent mailing

Outcome or subgroup title	No. of studies	No. of participants	Statistical method	Effect size
7.1 First response	4	8942	Odds Ratio (M‐H, Random, 95% CI)	1.96 [1.53, 2.53]
7.2 Final response	4	8942	Odds Ratio (M‐H, Random, 95% CI)	1.14 [1.03, 1.26]

**Comparison 8:** Offer of survey results vs. no offer

Outcome or subgroup title	No. of studies	No. of participants	Statistical method	Effect size
8.1 First response	7	11095	Odds Ratio (M‐H, Random, 95% CI)	1.01 [0.85, 1.20]
8.2 Final response	13	20052	Odds Ratio (M‐H, Random, 95% CI)	0.91 [0.78, 1.05]
8.3 e ‐ Submission	2	2884	Odds Ratio (M‐H, Random, 95% CI)	1.36 [1.16, 1.59]

**Comparison 9:** Pen vs. no pen

Outcome or subgroup title	No. of studies	No. of participants	Statistical method	Effect size
9.1 First Response	12	31820	Odds Ratio (M‐H, Random, 95% CI)	1.31 [1.16, 1.47]
9.2 Final Response	14	46096	Odds Ratio (M‐H, Random, 95% CI)	1.32 [1.14, 1.53]

**Comparison 10:** Cheap pen vs. expensive pen

Outcome or subgroup title	No. of studies	No. of participants	Statistical method	Effect size
10.1 First response	1	6167	Odds Ratio (M‐H, Random, 95% CI)	1.03 [0.81, 1.31]
10.2 Final Response	1	6167	Odds Ratio (M‐H, Random, 95% CI)	1.03 [0.81, 1.31]

**Comparison 11:** Boxed pen vs. unboxed

Outcome or subgroup title	No. of studies	No. of participants	Statistical method	Effect size
11.1 First Response	1	6167	Odds Ratio (M‐H, Random, 95% CI)	1.10 [1.00, 1.22]
11.2 Final Response	1	6167	Odds Ratio (M‐H, Random, 95% CI)	1.10 [1.00, 1.22]

**Comparison 12:** Monetary incentive with follow‐up vs. no incentive with follow‐up

Outcome or subgroup title	No. of studies	No. of participants	Statistical method	Effect size
12.1 First response	1	2342	Odds Ratio (M‐H, Random, 95% CI)	0.99 [0.84, 1.17]
12.2 Final response	1	2342	Odds Ratio (M‐H, Random, 95% CI)	0.97 [0.82, 1.16]

**Comparison 13:** Non‐monetary incentive vs. charitable donation

Outcome or subgroup title	No. of studies	No. of participants	Statistical method	Effect size
13.1 Final response	1	444	Odds Ratio (M‐H, Random, 95% CI)	1.44 [0.98, 2.12]

**Comparison 14:** Cheque incentive requiring ID vs. no ID

Outcome or subgroup title	No. of studies	No. of participants	Statistical method	Effect size
14.1 Final response	1	531	Odds Ratio (M‐H, Random, 95% CI)	0.75 [0.41, 1.37]

**Comparison 15:** Study brochure vs. no brochure

Outcome or subgroup title	No. of studies	No. of participants	Statistical method	Effect size
15.1 First response	3	7908	Odds Ratio (M‐H, Random, 95% CI)	0.89 [0.64, 1.24]
15.3 Final response	11	19981	Odds Ratio (M‐H, Random, 95% CI)	0.97 [0.83, 1.13]

**Comparison 16:** Cheque incentive vs. cashcard

Outcome or subgroup title	No. of studies	No. of participants	Statistical method	Effect size
16.2 First Response	1	303	Odds Ratio (M‐H, Random, 95% CI)	1.81 [1.15, 2.86]

**Comparison 17:** Single cash note vs. multiple notes

Outcome or subgroup title	No. of studies	No. of participants	Statistical method	Effect size
17.2 Final Response	1	2856	Odds Ratio (M‐H, Random, 95% CI)	1.08 [0.94, 1.26]

**Comparison 18:** Higher denominations in monetary lottery incentives vs. lower

Outcome or subgroup title	No. of studies	No. of participants	Statistical method	Effect size
18.1 e ‐ Submission	2	4721	Odds Ratio (M‐H, Random, 95% CI)	1.00 [0.87, 1.14]

**Comparison 19:** Unconditional and conditional incentives vs. conditional incentives

Outcome or subgroup title	No. of studies	No. of participants	Statistical method	Effect size
19.1 e ‐ Submission	1	1061	Odds Ratio (M‐H, Random, 95% CI)	1.19 [0.92, 1.54]

**Comparison 20:** Immediate notification of lottery results vs. delayed notification

Outcome or subgroup title	No. of studies	No. of participants	Statistical method	Effect size
20.1 e ‐ Submission	1	2233	Odds Ratio (M‐H, Random, 95% CI)	1.37 [1.13, 1.65]

**Comparison 21:** Unconditional and conditional incentives vs. unconditional incentive

Outcome or subgroup title	No. of studies	No. of participants	Statistical method	Effect size
21.3 e ‐ Login	1	3000	Odds Ratio (M‐H, Random, 95% CI)	1.16 [1.00, 1.36]
21.4 e ‐ Submission	1	3000	Odds Ratio (M‐H, Random, 95% CI)	1.23 [1.06, 1.44]

**Comparison 22:** Electronic with game vs. no game

Outcome or subgroup title	No. of studies	No. of participants	Statistical method	Effect size
22.2 Final Response	1	130	Odds Ratio (M‐H, Random, 95% CI)	0.51 [0.19, 1.40]

**Comparison 23:** More vs. less personalised

Outcome or subgroup title	No. of studies	No. of participants	Statistical method	Effect size
23.1 First response	40	38004	Odds Ratio (M‐H, Random, 95% CI)	1.22 [1.11, 1.33]
23.2 Final response	74	97674	Odds Ratio (M‐H, Random, 95% CI)	1.15 [1.09, 1.21]
23.3 e ‐ Login	5	24557	Odds Ratio (M‐H, Random, 95% CI)	1.26 [1.13, 1.40]
23.4 e ‐ Submission	12	48910	Odds Ratio (M‐H, Random, 95% CI)	1.24 [1.17, 1.32]

**Comparison 24:** Handwritten vs. typed/facsimile/scanned/printed signature on covering letter

Outcome or subgroup title	No. of studies	No. of participants	Statistical method	Effect size
24.1 First response	1	590	Odds Ratio (M‐H, Random, 95% CI)	1.08 [0.75, 1.54]
24.2 Final response	14	15006	Odds Ratio (M‐H, Random, 95% CI)	1.24 [1.08, 1.41]

**Comparison 25:** Handwritten address vs. computer‐printed

Outcome or subgroup title	No. of studies	No. of participants	Statistical method	Effect size
25.1 First response	4	1789	Odds Ratio (M‐H, Random, 95% CI)	1.35 [1.04, 1.74]
25.2 Final response	9	6030	Odds Ratio (M‐H, Random, 95% CI)	1.23 [1.09, 1.37]

**Comparison 26:** Signed vs. unsigned

Outcome or subgroup title	No. of studies	No. of participants	Statistical method	Effect size
26.1 First response	1	334	Odds Ratio (M‐H, Random, 95% CI)	1.38 [0.86, 2.22]
26.2 Final response	3	1364	Odds Ratio (M‐H, Random, 95% CI)	1.35 [1.04, 1.76]

**Comparison 27:** Identifying feature on return vs. none

Outcome or subgroup title	No. of studies	No. of participants	Statistical method	Effect size
27.1 First response	5	3923	Odds Ratio (M‐H, Random, 95% CI)	1.00 [0.72, 1.39]
27.2 Final response	10	5297	Odds Ratio (M‐H, Random, 95% CI)	1.03 [0.81, 1.32]

**Comparison 28:** Identifying number on return vs. other identifier

Outcome or subgroup title	No. of studies	No. of participants	Statistical method	Effect size
28.1 First response	1	741	Odds Ratio (M‐H, Random, 95% CI)	1.00 [0.68, 1.46]
28.2 Final response	1	741	Odds Ratio (M‐H, Random, 95% CI)	1.00 [0.68, 1.46]

**Comparison 29:** Coloured vs. white questionnaire

Outcome or subgroup title	No. of studies	No. of participants	Statistical method	Effect size
29.1 First response	6	14005	Odds Ratio (M‐H, Random, 95% CI)	1.07 [0.99, 1.15]
29.2 Final response	15	43754	Odds Ratio (M‐H, Random, 95% CI)	1.03 [0.98, 1.09]

**Comparison 30:** Coloured vs. standard (black/blue) ink

Outcome or subgroup title	No. of studies	No. of participants	Statistical method	Effect size
30.1 First response	2	6064	Odds Ratio (M‐H, Random, 95% CI)	1.25 [1.03, 1.53]
30.2 Final response	3	7040	Odds Ratio (M‐H, Random, 95% CI)	1.16 [0.95, 1.42]

**Comparison 31:** Coloured vs. black & white letterhead

Outcome or subgroup title	No. of studies	No. of participants	Statistical method	Effect size
31.1 First response	1	1650	Odds Ratio (M‐H, Random, 95% CI)	0.99 [0.80, 1.24]
31.2 Final response	2	2356	Odds Ratio (M‐H, Random, 95% CI)	1.08 [0.91, 1.28]

**Comparison 32:** Illustration on cover of q'aire largely in black vs. largely in white

Outcome or subgroup title	No. of studies	No. of participants	Statistical method	Effect size
32.1 Final response	1	320	Odds Ratio (M‐H, Random, 95% CI)	1.62 [1.04, 2.53]

**Comparison 33:** Folder or booklet vs. stapled pages

Outcome or subgroup title	No. of studies	No. of participants	Statistical method	Effect size
33.1 First response	2	1845	Odds Ratio (M‐H, Random, 95% CI)	1.17 [0.94, 1.45]
33.2 Final response	3	5681	Odds Ratio (M‐H, Random, 95% CI)	1.10 [0.99, 1.23]

**Comparison 34:** Large paper size vs. small

Outcome or subgroup title	No. of studies	No. of participants	Statistical method	Effect size
34.1 First response	1	2000	Odds Ratio (M‐H, Random, 95% CI)	0.83 [0.67, 1.03]
34.2 Final response	2	2145	Odds Ratio (M‐H, Random, 95% CI)	0.88 [0.56, 1.39]

**Comparison 35:** Dot matrix print vs. letter quality print

Outcome or subgroup title	No. of studies	No. of participants	Statistical method	Effect size
35.1 Final response	1	176	Odds Ratio (M‐H, Random, 95% CI)	1.15 [0.63, 2.10]

**Comparison 36:** Questionnaire printed on high vs. standard quality paper or thick paper vs. thin

Outcome or subgroup title	No. of studies	No. of participants	Statistical method	Effect size
36.1 Final response	3	3372	Odds Ratio (M‐H, Random, 95% CI)	0.83 [0.68, 1.02]

**Comparison 37:** Single vs. double‐sided questionnaire

Outcome or subgroup title	No. of studies	No. of participants	Statistical method	Effect size
37.1 First response	2	5025	Odds Ratio (M‐H, Random, 95% CI)	1.14 [0.95, 1.36]
37.2 Final response	5	9383	Odds Ratio (M‐H, Random, 95% CI)	1.13 [1.02, 1.25]

**Comparison 38:** Large font size vs. small

Outcome or subgroup title	No. of studies	No. of participants	Statistical method	Effect size
38.1 Final response	1	650	Odds Ratio (M‐H, Random, 95% CI)	1.26 [0.87, 1.82]

**Comparison 39:** Study logo on several items in the mailing package vs. on questionnaire only

Outcome or subgroup title	No. of studies	No. of participants	Statistical method	Effect size
39.1 Final response	1	1000	Odds Ratio (M‐H, Random, 95% CI)	0.92 [0.72, 1.18]

**Comparison 40:** Picture of researcher/images vs. none

Outcome or subgroup title	No. of studies	No. of participants	Statistical method	Effect size
40.1 First response	2	630	Odds Ratio (M‐H, Random, 95% CI)	1.10 [0.72, 1.69]
40.2 Final response	5	3956	Odds Ratio (M‐H, Random, 95% CI)	1.03 [0.70, 1.51]
40.3 e ‐ Submission	2	720	Odds Ratio (M‐H, Random, 95% CI)	3.05 [1.84, 5.06]

**Comparison 41:** Attractive vs. less attractive picture

Outcome or subgroup title	No. of studies	No. of participants	Statistical method	Effect size
41.1 e ‐ Submission	2	520	Odds Ratio (M‐H, Random, 95% CI)	3.44 [0.72, 16.49]

**Comparison 42:** Cartoons included vs. not included

Outcome or subgroup title	No. of studies	No. of participants	Statistical method	Effect size
42.1 Final response	1	280	Odds Ratio (M‐H, Random, 95% CI)	1.00 [0.62, 1.62]

**Comparison 43:** Professional design vs. standard

Outcome or subgroup title	No. of studies	No. of participants	Statistical method	Effect size
43.1 First response	2	2904	Odds Ratio (M‐H, Random, 95% CI)	1.00 [0.58, 1.72]
43.2 Final outcome	2	2904	Odds Ratio (M‐H, Random, 95% CI)	1.00 [0.58, 1.72]

**Comparison 44:** Personalised SMS reminder vs. standard SMS reminder

Outcome or subgroup title	No. of studies	No. of participants	Statistical method	Effect size
44.1 First Response	2	901	Odds Ratio (M‐H, Random, 95% CI)	1.37 [1.00, 1.88]
44.2 Final Response	2	901	Odds Ratio (M‐H, Random, 95% CI)	1.53 [0.97, 2.43]

**Comparison 45:** "Action Required" subject line vs. "Reminder"

Outcome or subgroup title	No. of studies	No. of participants	Statistical method	Effect size
45.1 First response	1	231	Odds Ratio (M‐H, Random, 95% CI)	0.49 [0.28, 0.83]
45.2 Final response	1	231	Odds Ratio (M‐H, Random, 95% CI)	0.61 [0.23, 1.63]

**Comparison 46:** Message about incentive on envelope vs. no message

Outcome or subgroup title	No. of studies	No. of participants	Statistical method	Effect size
46.1 First response	2	3895	Odds Ratio (M‐H, Random, 95% CI)	0.92 [0.75, 1.14]
46.2 Final response	2	3895	Odds Ratio (M‐H, Random, 95% CI)	0.91 [0.80, 1.04]

**Comparison 47:** Health message vs. monetary message

Outcome or subgroup title	No. of studies	No. of participants	Statistical method	Effect size
47.1 First response	1	1569	Odds Ratio (M‐H, Random, 95% CI)	1.11 [0.91, 1.35]
47.2 Final response	1	1569	Odds Ratio (M‐H, Random, 95% CI)	1.05 [0.86, 1.29]

**Comparison 48:** Teaser on envelope vs. no teaser

Outcome or subgroup title	No. of studies	No. of participants	Statistical method	Effect size
48.1 First response	3	4013	Odds Ratio (M‐H, Random, 95% CI)	1.45 [0.96, 2.20]
48.2 Final response	5	23621	Odds Ratio (M‐H, Random, 95% CI)	1.08 [0.95, 1.22]

**Comparison 49:** More readable/concise/info mapped letter vs. standard

Outcome or subgroup title	No. of studies	No. of participants	Statistical method	Effect size
49.2 Final response	2	1678	Odds Ratio (M‐H, Random, 95% CI)	0.88 [0.72, 1.09]

**Comparison 50:** Logo sticker on envelope vs. no sticker

Outcome or subgroup title	No. of studies	No. of participants	Statistical method	Effect size
50.1 First response	1	517	Odds Ratio (M‐H, Random, 95% CI)	1.06 [0.75, 1.49]

**Comparison 51:** Subject line vs. blank

Outcome or subgroup title	No. of studies	No. of participants	Statistical method	Effect size
51.1 e ‐ Login	2	6152	Odds Ratio (M‐H, Random, 95% CI)	0.83 [0.70, 0.99]
51.2 e ‐ Submission	2	6152	Odds Ratio (M‐H, Random, 95% CI)	0.84 [0.71, 1.01]

**Comparison 52:** "Survey" subject line vs. blank

Outcome or subgroup title	No. of studies	No. of participants	Statistical method	Effect size
52.1 e ‐ Login	2	3845	Odds Ratio (M‐H, Random, 95% CI)	0.80 [0.67, 0.97]
52.2 e ‐ Submission	2	3845	Odds Ratio (M‐H, Random, 95% CI)	0.81 [0.67, 0.97]

**Comparison 53:** Text vs. HTML file formats

Outcome or subgroup title	No. of studies	No. of participants	Statistical method	Effect size
53.1 e ‐ Submission	1	6090	Odds Ratio (M‐H, Random, 95% CI)	1.00 [0.84, 1.19]

**Comparison 54:** White background vs. black

Outcome or subgroup title	No. of studies	No. of participants	Statistical method	Effect size
54.1 e ‐ Submission	1	6090	Odds Ratio (M‐H, Random, 95% CI)	1.31 [1.10, 1.56]

**Comparison 55:** Header vs. no header

Outcome or subgroup title	No. of studies	No. of participants	Statistical method	Effect size
55.1 e ‐ Submission	1	6090	Odds Ratio (M‐H, Random, 95% CI)	1.13 [0.90, 1.41]

**Comparison 56:** Simple vs. complex header

Outcome or subgroup title	No. of studies	No. of participants	Statistical method	Effect size
56.1 e ‐ Submission	1	5075	Odds Ratio (M‐H, Random, 95% CI)	1.23 [1.03, 1.48]

**Comparison 57:** Textual presentation of response categories vs. visual presentation

Outcome or subgroup title	No. of studies	No. of participants	Statistical method	Effect size
57.1 e ‐ Submission	1	5413	Odds Ratio (M‐H, Random, 95% CI)	1.19 [1.05, 1.36]

**Comparison 58:** Scrollable web page vs. multiple web pages

Outcome or subgroup title	No. of studies	No. of participants	Statistical method	Effect size
58.1 Final response	1	517	Odds Ratio (M‐H, Random, 95% CI)	0.93 [0.66, 1.32]

**Comparison 59:** Subject line emphasises incentive vs. no emphasis

Outcome or subgroup title	No. of studies	No. of participants	Statistical method	Effect size
59.1 e ‐ Login	2	3676	Odds Ratio (M‐H, Random, 95% CI)	2.32 [0.59, 9.13]
59.2 e ‐ Submission	2	3676	Odds Ratio (M‐H, Random, 95% CI)	2.19 [0.58, 8.27]

**Comparison 60:** Email reminder including humour vs. standard email

Outcome or subgroup title	No. of studies	No. of participants	Statistical method	Effect size
60.1 e ‐ Submission	1	2963	Odds Ratio (M‐H, Random, 95% CI)	1.17 [0.99, 1.38]

**Comparison 61:** Stamped vs. franked outward envelope

Outcome or subgroup title	No. of studies	No. of participants	Statistical method	Effect size
61.1 First response	2	930	Odds Ratio (M‐H, Random, 95% CI)	1.04 [0.79, 1.37]
61.2 Final response	6	13964	Odds Ratio (M‐H, Random, 95% CI)	0.95 [0.88, 1.03]

**Comparison 62:** First vs. second/third class outward mailing

Outcome or subgroup title	No. of studies	No. of participants	Statistical method	Effect size
62.1 First response	1	7370	Odds Ratio (M‐H, Random, 95% CI)	1.12 [1.02, 1.23]
62.2 Final response	2	8300	Odds Ratio (M‐H, Random, 95% CI)	1.11 [1.02, 1.21]

**Comparison 63:** Commemorative/race‐specific vs. ordinary stamp on return envelope

Outcome or subgroup title	No. of studies	No. of participants	Statistical method	Effect size
63.1 First response	3	2430	Odds Ratio (M‐H, Random, 95% CI)	0.91 [0.66, 1.24]
63.2 Final response	5	5461	Odds Ratio (M‐H, Random, 95% CI)	0.92 [0.81, 1.06]

**Comparison 64:** Certified/special delivery vs. regular outward mailing

Outcome or subgroup title	No. of studies	No. of participants	Statistical method	Effect size
64.1 First response	12	18075	Odds Ratio (M‐H, Random, 95% CI)	2.12 [1.61, 2.79]
64.2 Final response	19	30492	Odds Ratio (M‐H, Random, 95% CI)	1.68 [1.36, 2.08]

**Comparison 65:** Stamped return vs. business reply/franked envelope

Outcome or subgroup title	No. of studies	No. of participants	Statistical method	Effect size
65.1 First response	16	34172	Odds Ratio (M‐H, Random, 95% CI)	1.21 [1.12, 1.32]
65.2 Final response	28	55550	Odds Ratio (M‐H, Random, 95% CI)	1.23 [1.13, 1.33]

**Comparison 66:** Priority stamps vs. first‐class stamps on return envelope

Outcome or subgroup title	No. of studies	No. of participants	Statistical method	Effect size
66.1 Final response	1	205	Odds Ratio (M‐H, Random, 95% CI)	0.26 [0.14, 0.46]

**Comparison 67:** First vs. second‐class stamp on return envelope

Outcome or subgroup title	No. of studies	No. of participants	Statistical method	Effect size
67.1 Final response	1	800	Odds Ratio (M‐H, Random, 95% CI)	0.91 [0.69, 1.21]

**Comparison 68:** Multiple stamps vs. single stamp on return envelope

Outcome or subgroup title	No. of studies	No. of participants	Statistical method	Effect size
68.1 Final response	1	510	Odds Ratio (M‐H, Random, 95% CI)	1.44 [1.01, 2.04]

**Comparison 69:** Pre‐paid return envelope vs. not pre‐paid

Outcome or subgroup title	No. of studies	No. of participants	Statistical method	Effect size
69.1 First response	3	2740	Odds Ratio (M‐H, Random, 95% CI)	1.23 [0.78, 1.95]
69.2 Final response	4	4094	Odds Ratio (M‐H, Random, 95% CI)	1.09 [0.71, 1.68]

**Comparison 70:** Stamped addressed return envelope vs. address label only included

Outcome or subgroup title	No. of studies	No. of participants	Statistical method	Effect size
70.1 Final response	1	147	Odds Ratio (M‐H, Random, 95% CI)	0.86 [0.45, 1.65]

**Comparison 71:** Questionnaire sent to work vs. home address

Outcome or subgroup title	No. of studies	No. of participants	Statistical method	Effect size
71.1 First response	2	1140	Odds Ratio (M‐H, Random, 95% CI)	1.16 [0.89, 1.52]
71.2 Final response	2	1140	Odds Ratio (M‐H, Random, 95% CI)	1.16 [0.89, 1.52]

**Comparison 72:** Window vs. regular envelope

Outcome or subgroup title	No. of studies	No. of participants	Statistical method	Effect size
72.1 First response	2	11781	Odds Ratio (M‐H, Random, 95% CI)	0.85 [0.68, 1.06]
72.2 Final response	2	11781	Odds Ratio (M‐H, Random, 95% CI)	0.96 [0.61, 1.49]

**Comparison 73:** Q'aire mailed in large vs. standard/small envelope

Outcome or subgroup title	No. of studies	No. of participants	Statistical method	Effect size
73.1 Final response	1	1200	Odds Ratio (M‐H, Random, 95% CI)	0.93 [0.74, 1.17]

**Comparison 74:** Questionnaire mailed on Monday vs. Friday

Outcome or subgroup title	No. of studies	No. of participants	Statistical method	Effect size
74.1 First response	2	1843	Odds Ratio (M‐H, Random, 95% CI)	0.84 [0.70, 1.01]
74.2 Final response	2	1843	Odds Ratio (M‐H, Random, 95% CI)	0.84 [0.70, 1.01]

**Comparison 75:** Questionnaire received on Monday/Tuesday vs. Friday

Outcome or subgroup title	No. of studies	No. of participants	Statistical method	Effect size
75.1 Final response	1	460	Odds Ratio (M‐H, Random, 95% CI)	1.00 [0.64, 1.56]
75.2 e ‐ Login	1	1943	Odds Ratio (M‐H, Random, 95% CI)	1.12 [0.80, 1.57]
75.3 e ‐ Submission	1	1943	Odds Ratio (M‐H, Random, 95% CI)	0.96 [0.66, 1.40]

**Comparison 76:** Q'aire sent 1‐5 weeks vs. 9‐14 weeks after hospital discharge

Outcome or subgroup title	No. of studies	No. of participants	Statistical method	Effect size
76.1 Final response	2	2324	Odds Ratio (M‐H, Random, 95% CI)	2.26 [0.69, 7.37]

**Comparison 77:** Padded envelope vs. priority mail envelope

Outcome or subgroup title	No. of studies	No. of participants	Statistical method	Effect size
77.1 First response	1	1600	Odds Ratio (M‐H, Random, 95% CI)	0.88 [0.72, 1.07]

**Comparison 78:** Hand delivery by known person vs. postal delivery

Outcome or subgroup title	No. of studies	No. of participants	Statistical method	Effect size
78.1 Final response	1	135	Odds Ratio (M‐H, Random, 95% CI)	2.60 [1.29, 5.23]

**Comparison 79:** Hand delivery vs. postal delivery

Outcome or subgroup title	No. of studies	No. of participants	Statistical method	Effect size
79.1 Final response	2	937	Odds Ratio (M‐H, Random, 95% CI)	1.44 [0.50, 4.15]

**Comparison 80:** Postal vs. fax

Outcome or subgroup title	No. of studies	No. of participants	Statistical method	Effect size
80.1 First Response	1	199	Odds Ratio (M‐H, Random, 95% CI)	0.58 [0.29, 1.14]
80.2 Final Response	1	199	Odds Ratio (M‐H, Random, 95% CI)	0.58 [0.29, 1.14]

**Comparison 81:** Postal vs. electronic

Outcome or subgroup title	No. of studies	No. of participants	Statistical method	Effect size
81.1 First Response	21	62576	Odds Ratio (M‐H, Random, 95% CI)	1.93 [1.36, 2.72]
81.2 Final Response	27	66118	Odds Ratio (M‐H, Random, 95% CI)	1.76 [1.34, 2.32]

**Comparison 82:** Choice (electronic/postal) vs. only postal response

Outcome or subgroup title	No. of studies	No. of participants	Statistical method	Effect size
82.1 First response	4	19983	Odds Ratio (M‐H, Random, 95% CI)	1.02 [0.96, 1.09]
82.2 Final response	8	20909	Odds Ratio (M‐H, Random, 95% CI)	0.94 [0.86, 1.02]

**Comparison 83:** Postal with electronic follow‐up vs. electronic with postal follow‐up

Outcome or subgroup title	No. of studies	No. of participants	Statistical method	Effect size
83.1 First response	3	2325	Odds Ratio (M‐H, Random, 95% CI)	1.45 [0.31, 6.70]
83.2 Final response	4	2958	Odds Ratio (M‐H, Random, 95% CI)	1.19 [0.76, 1.87]

**Comparison 84:** Electronic vs. choice (postal/electronic)

Outcome or subgroup title	No. of studies	No. of participants	Statistical method	Effect size
84.1 First response	9	45079	Odds Ratio (M‐H, Random, 95% CI)	0.56 [0.39, 0.80]
84.2 Final response	10	39523	Odds Ratio (M‐H, Random, 95% CI)	1.63 [1.18, 2.26]

**Comparison 85:** Pre‐selected choice vs. choice

Outcome or subgroup title	No. of studies	No. of participants	Statistical method	Effect size
85.2 Final response	1	6188	Odds Ratio (M‐H, Random, 95% CI)	1.59 [1.43, 1.77]

**Comparison 86:** Mobile‐based web survey vs. computer

Outcome or subgroup title	No. of studies	No. of participants	Statistical method	Effect size
86.1 Started questionnaire	1	2774	Odds Ratio (M‐H, Random, 95% CI)	1.58 [1.32, 1.89]
86.2 Completed questionnaire	1	2774	Odds Ratio (M‐H, Random, 95% CI)	1.62 [1.36, 1.94]

**Comparison 87:** Mobile and web vs. paper and email

Outcome or subgroup title	No. of studies	No. of participants	Statistical method	Effect size
87.1 First Response	1	620	Odds Ratio (M‐H, Random, 95% CI)	1.02 [0.50, 2.08]
87.2 Final Response	1	620	Odds Ratio (M‐H, Random, 95% CI)	1.02 [0.50, 2.08]

**Comparison 88:** Electronic vs. fax

Outcome or subgroup title	No. of studies	No. of participants	Statistical method	Effect size
88.1 First Response	1	195	Odds Ratio (M‐H, Random, 95% CI)	3.87 [2.00, 7.49]
88.2 Final Response	1	195	Odds Ratio (M‐H, Random, 95% CI)	3.87 [2.00, 7.49]

**Comparison 89:** SMS vs. postal

Outcome or subgroup title	No. of studies	No. of participants	Statistical method	Effect size
89.2 Final Response	1	382	Odds Ratio (M‐H, Random, 95% CI)	1.19 [0.60, 2.32]

**Comparison 90:** Emails and reminders sent on varying vs. fixed days

Outcome or subgroup title	No. of studies	No. of participants	Statistical method	Effect size
90.2 Final response	1	21473	Odds Ratio (M‐H, Random, 95% CI)	1.08 [1.03, 1.14]

**Comparison 91:** Emails and reminders sent on best predicted days vs. fixed days

Outcome or subgroup title	No. of studies	No. of participants	Statistical method	Effect size
91.2 Final response	1	21531	Odds Ratio (M‐H, Random, 95% CI)	1.05 [1.00, 1.11]

**Comparison 92:** Electronic with mail FU vs. electronic with interactive voice response FU

Outcome or subgroup title	No. of studies	No. of participants	Statistical method	Effect size
92.2 Final response	1	1999	Odds Ratio (M‐H, Random, 95% CI)	1.77 [1.48, 2.11]

**Comparison 93:** SMS vs. web

Outcome or subgroup title	No. of studies	No. of participants	Statistical method	Effect size
93.2 Final Response	1	353	Odds Ratio (M‐H, Random, 95% CI)	0.68 [0.31, 1.49]

**Comparison 94:** Pre‐contact vs. no pre‐contact

Outcome or subgroup title	No. of studies	No. of participants	Statistical method	Effect size
94.1 First response	37	1362390	Odds Ratio (M‐H, Random, 95% CI)	1.32 [1.16, 1.49]
94.2 Final response	59	89146	Odds Ratio (M‐H, Random, 95% CI)	1.36 [1.23, 1.51]
94.3 e ‐ Login	2	1199	Odds Ratio (M‐H, Random, 95% CI)	1.30 [0.74, 2.26]
94.4 e ‐ Submission	3	3049	Odds Ratio (M‐H, Random, 95% CI)	1.85 [0.99, 3.45]

**Comparison 95:** Pre‐contact by phone vs. mail

Outcome or subgroup title	No. of studies	No. of participants	Statistical method	Effect size
95.1 First response	3	978	Odds Ratio (M‐H, Random, 95% CI)	1.40 [1.02, 1.93]
95.2 Final response	7	3322	Odds Ratio (M‐H, Random, 95% CI)	1.18 [0.77, 1.80]

**Comparison 96:** Follow‐up vs. no follow‐up

Outcome or subgroup title	No. of studies	No. of participants	Statistical method	Effect size
96.1 First response	13	1315514	Odds Ratio (M‐H, Random, 95% CI)	1.52 [1.25, 1.84]
96.2 Final response	24	53555	Odds Ratio (M‐H, Random, 95% CI)	1.33 [1.18, 1.49]

**Comparison 97:** Postal follow‐up including vs. excluding q'aire

Outcome or subgroup title	No. of studies	No. of participants	Statistical method	Effect size
97.1 First response	7	6108	Odds Ratio (M‐H, Random, 95% CI)	1.22 [0.94, 1.60]
97.2 Final response	13	11456	Odds Ratio (M‐H, Random, 95% CI)	1.41 [1.13, 1.77]

**Comparison 98:** Follow‐up by phone vs. mail

Outcome or subgroup title	No. of studies	No. of participants	Statistical method	Effect size
98.1 First Response	5	2631	Odds Ratio (M‐H, Random, 95% CI)	0.82 [0.48, 1.39]
98.2 Final Response	8	4057	Odds Ratio (M‐H, Random, 95% CI)	1.02 [0.76, 1.38]

**Comparison 99:** Telephone reminder vs. no reminder

Outcome or subgroup title	No. of studies	No. of participants	Statistical method	Effect size
99.1 First response	2	1364	Odds Ratio (M‐H, Random, 95% CI)	15.57 [0.59, 410.80]
99.2 Final response	4	15143	Odds Ratio (M‐H, Random, 95% CI)	1.96 [1.03, 3.74]

**Comparison 100:** Higher frequency follow‐up interval vs. lower

Outcome or subgroup title	No. of studies	No. of participants	Statistical method	Effect size
100.1 First response	2	1608	Odds Ratio (M‐H, Random, 95% CI)	0.87 [0.50, 1.50]
100.2 Final response	6	7520	Odds Ratio (M‐H, Random, 95% CI)	1.13 [1.02, 1.25]

**Comparison 101:** Pre‐contact by letter vs. postcard

Outcome or subgroup title	No. of studies	No. of participants	Statistical method	Effect size
101.1 First Response	1	780	Odds Ratio (M‐H, Random, 95% CI)	1.07 [0.80, 1.43]
101.2 Final Response	1	780	Odds Ratio (M‐H, Random, 95% CI)	0.98 [0.74, 1.30]
101.3 e‐Submission	1	500	Odds Ratio (M‐H, Fixed, 95% CI)	1.38 [0.76, 2.49]

**Comparison 102:** Pre‐contact letter vs email

Outcome or subgroup title	No. of studies	No. of participants	Statistical method	Effect size
102.1 First Response	1	581	Odds Ratio (M‐H, Random, 95% CI)	1.27 [0.71, 2.27]
102.2 Final Response	1	581	Odds Ratio (M‐H, Random, 95% CI)	1.25 [0.83, 1.88]

**Comparison 103:** Pre‐contact by fax vs. mail

Outcome or subgroup title	No. of studies	No. of participants	Statistical method	Effect size
103.1 First response	1	930	Odds Ratio (M‐H, Random, 95% CI)	1.01 [0.74, 1.36]
103.2 Final response	1	930	Odds Ratio (M‐H, Random, 95% CI)	0.92 [0.71, 1.20]

**Comparison 104:** Electronic reminder vs. no reminder

Outcome or subgroup title	No. of studies	No. of participants	Statistical method	Effect size
104.1 First response	2	582	Odds Ratio (M‐H, Random, 95% CI)	1.80 [0.88, 3.68]
104.2 Final response	2	582	Odds Ratio (M‐H, Random, 95% CI)	1.80 [0.88, 3.68]

**Comparison 105:** Push‐to‐web (web, web/paper reminder) vs. mail push (mail, mail/web reminder)

Outcome or subgroup title	No. of studies	No. of participants	Statistical method	Effect size
105.2 Final Response	2	3824	Odds Ratio (M‐H, Random, 95% CI)	1.10 [0.87, 1.39]

**Comparison 106:** Postal vs mixed‐mode reminder (electronic/postal)

Outcome or subgroup title	No. of studies	No. of participants	Statistical method	Effect size
106.1 Final Response	4	3998	Odds Ratio (M‐H, Random, 95% CI)	1.13 [0.83, 1.52]

**Comparison 107:** Telephone reminder vs. standard reminder

Outcome or subgroup title	No. of studies	No. of participants	Statistical method	Effect size
107.1 First Response	1	176	Odds Ratio (M‐H, Random, 95% CI)	2.29 [1.17, 4.51]
107.2 Final Response	4	520	Odds Ratio (M‐H, Random, 95% CI)	1.63 [1.06, 2.50]

**Comparison 108:** Email invitation to web survey vs. postal invitation

Outcome or subgroup title	No. of studies	No. of participants	Statistical method	Effect size
108.1 First Response	3	18748	Odds Ratio (M‐H, Random, 95% CI)	2.26 [2.03, 2.53]
108.2 Final Response	5	24373	Odds Ratio (M‐H, Random, 95% CI)	1.81 [0.81, 4.01]

**Comparison 109:** Intensive follow‐up vs. limited

Outcome or subgroup title	No. of studies	No. of participants	Statistical method	Effect size
109.1 Final response	1	431	Odds Ratio (M‐H, Random, 95% CI)	1.69 [0.93, 3.06]

**Comparison 110:** Pre‐notification SMS vs. post‐notification SMS

Outcome or subgroup title	No. of studies	No. of participants	Statistical method	Effect size
110.2 Final response	2	771	Odds Ratio (M‐H, Random, 95% CI)	1.29 [0.66, 2.54]

**Comparison 111:** Electronic with postal reminder vs. postal with electronic reminder

Outcome or subgroup title	No. of studies	No. of participants	Statistical method	Effect size
111.2 Final Response	1	5837	Odds Ratio (M‐H, Random, 95% CI)	1.05 [0.95, 1.16]

**Comparison 112:** Study calendar with prompts vs. no calendar

Outcome or subgroup title	No. of studies	No. of participants	Statistical method	Effect size
112.2 Final Response	1	296	Odds Ratio (M‐H, Random, 95% CI)	1.00 [0.57, 1.73]

**Comparison 113:** SMS vs. postcard reminder

Outcome or subgroup title	No. of studies	No. of participants	Statistical method	Effect size
113.1 Final response	3	9947	Odds Ratio (M‐H, Random, 95% CI)	1.49 [1.23, 1.81]

**Comparison 114:** Electronic vs. mixed‐mode reminder (email and postal)

Outcome or subgroup title	No. of studies	No. of participants	Statistical method	Effect size
114.1 First Response	2	3999	Odds Ratio (M‐H, Random, 95% CI)	1.40 [0.76, 2.59]
114.2 Final Response	3	7159	Odds Ratio (M‐H, Random, 95% CI)	1.96 [0.89, 4.31]

**Comparison 115:** Electronic first vs. mixed‐mode first (mixed‐mode FU)

Outcome or subgroup title	No. of studies	No. of participants	Statistical method	Effect size
115.1 First Response	1	734	Odds Ratio (M‐H, Random, 95% CI)	1.44 [1.03, 2.01]
115.2 Final Response	1	734	Odds Ratio (M‐H, Random, 95% CI)	1.54 [1.15, 2.07]

**Comparison 116:** Push‐to‐web vs. choice

Outcome or subgroup title	No. of studies	No. of participants	Statistical method	Effect size
116.2 Final Response	4	26482	Odds Ratio (M‐H, Random, 95% CI)	1.09 [0.99, 1.20]

**Comparison 117:** Push‐to‐web (web, web/paper reminder) vs. mail only

Outcome or subgroup title	No. of studies	No. of participants	Statistical method	Effect size
117.2 Final Response	1	3508	Odds Ratio (M‐H, Random, 95% CI)	1.26 [1.10, 1.45]

**Comparison 118:** Mail push (mail, mail/web reminder) vs. mail only

Outcome or subgroup title	No. of studies	No. of participants	Statistical method	Effect size
118.2 Final Response	1	3508	Odds Ratio (M‐H, Random, 95% CI)	0.96 [0.84, 1.10]

**Comparison 119:** Push‐to‐web with email augmentation vs. no email augmentation

Outcome or subgroup title	No. of studies	No. of participants	Statistical method	Effect size
119.1 Final response	1	2982	Odds Ratio (M‐H, Random, 95% CI)	1.13 [0.98, 1.31]

**Comparison 120:** SMS reminder with URL vs. without

Outcome or subgroup title	No. of studies	No. of participants	Statistical method	Effect size
120.1 First Response	1	178	Odds Ratio (M‐H, Random, 95% CI)	0.70 [0.30, 1.61]
120.2 Final Response	1	178	Odds Ratio (M‐H, Fixed, 95% CI)	1.00 [0.55, 1.82]

**Comparison 121:** Electronic prompt vs. none

Outcome or subgroup title	No. of studies	No. of participants	Statistical method	Effect size
121.1 Final Response	1	125	Odds Ratio (M‐H, Random, 95% CI)	1.27 [0.47, 3.48]

**Comparison 122:** Sensitive questions vs. no/fewer/less sensitive questions asked

Outcome or subgroup title	No. of studies	No. of participants	Statistical method	Effect size
122.1 First response	5	11292	Odds Ratio (M‐H, Random, 95% CI)	0.98 [0.90, 1.07]
122.2 Final response	10	21393	Odds Ratio (M‐H, Random, 95% CI)	0.94 [0.88, 1.00]

**Comparison 123:** More relevant questions first vs. last

Outcome or subgroup title	No. of studies	No. of participants	Statistical method	Effect size
123.1 First response	1	5817	Odds Ratio (M‐H, Random, 95% CI)	1.28 [1.15, 1.42]
123.2 Final response	1	5817	Odds Ratio (M‐H, Random, 95% CI)	1.23 [1.10, 1.37]

**Comparison 124:** Most general questions first vs. last

Outcome or subgroup title	No. of studies	No. of participants	Statistical method	Effect size
124.1 Final response	3	11435	Odds Ratio (M‐H, Random, 95% CI)	0.95 [0.83, 1.09]

**Comparison 125:** Demographic items first vs. last

Outcome or subgroup title	No. of studies	No. of participants	Statistical method	Effect size
125.1 First response	2	1040	Odds Ratio (M‐H, Random, 95% CI)	1.06 [0.83, 1.36]
125.2 Final response	5	10565	Odds Ratio (M‐H, Random, 95% CI)	1.02 [0.90, 1.16]

**Comparison 126:** Easier questions first vs. last

Outcome or subgroup title	No. of studies	No. of participants	Statistical method	Effect size
126.1 First response	1	240	Odds Ratio (M‐H, Random, 95% CI)	1.80 [0.91, 3.56]
126.2 Final response	2	3182	Odds Ratio (M‐H, Random, 95% CI)	1.61 [1.14, 2.26]

**Comparison 127:** User friendly vs. standard questionnaire

Outcome or subgroup title	No. of studies	No. of participants	Statistical method	Effect size
127.1 First response	1	3540	Odds Ratio (M‐H, Random, 95% CI)	1.46 [1.21, 1.75]
127.2 Final response	2	4087	Odds Ratio (M‐H, Random, 95% CI)	1.47 [1.25, 1.73]

**Comparison 128:** More interesting vs. less or high salient topic vs. low

Outcome or subgroup title	No. of studies	No. of participants	Statistical method	Effect size
128.1 First response	2	2151	Odds Ratio (M‐H, Random, 95% CI)	2.44 [1.99, 3.01]
128.2 Final response	4	6491	Odds Ratio (M‐H, Random, 95% CI)	1.73 [1.12, 2.66]
128.3 e ‐ Submission	1	2176	Odds Ratio (M‐H, Random, 95% CI)	1.85 [1.52, 2.26]

**Comparison 129:** Open‐ended vs. closed questions

Outcome or subgroup title	No. of studies	No. of participants	Statistical method	Effect size
129.1 First response	1	372	Odds Ratio (M‐H, Random, 95% CI)	0.38 [0.25, 0.59]
129.2 Final response	4	3092	Odds Ratio (M‐H, Random, 95% CI)	0.43 [0.19, 0.98]

**Comparison 130:** Open‐ended items first vs. other items first

Outcome or subgroup title	No. of studies	No. of participants	Statistical method	Effect size
130.1 First response	1	300	Odds Ratio (M‐H, Random, 95% CI)	0.89 [0.55, 1.44]
130.2 Final response	1	300	Odds Ratio (M‐H, Random, 95% CI)	1.26 [0.73, 2.19]

**Comparison 131:** Closed‐ended items first vs. other items first

Outcome or subgroup title	No. of studies	No. of participants	Statistical method	Effect size
131.1 First response	1	300	Odds Ratio (M‐H, Random, 95% CI)	1.15 [0.71, 1.86]
131.2 Final response	1	300	Odds Ratio (M‐H, Random, 95% CI)	0.93 [0.54, 1.59]

**Comparison 132:** 'Don't know' boxes included vs. not

Outcome or subgroup title	No. of studies	No. of participants	Statistical method	Effect size
132.1 Final response	1	1360	Odds Ratio (M‐H, Random, 95% CI)	1.03 [0.82, 1.29]

**Comparison 133:** Circle answer vs. tick box format

Outcome or subgroup title	No. of studies	No. of participants	Statistical method	Effect size
133.1 Final response	2	1125	Odds Ratio (M‐H, Random, 95% CI)	0.96 [0.74, 1.26]

**Comparison 134:** Response options listed in increasing vs. decreasing order

Outcome or subgroup title	No. of studies	No. of participants	Statistical method	Effect size
134.1 Final response	1	6783	Odds Ratio (M‐H, Random, 95% CI)	1.06 [0.94, 1.18]

**Comparison 135:** High vs. medium frequency response alternatives

Outcome or subgroup title	No. of studies	No. of participants	Statistical method	Effect size
135.1 Final response	2	3882	Odds Ratio (M‐H, Random, 95% CI)	1.40 [0.58, 3.38]

**Comparison 136:** 5‐step vs. 10‐step response scale

Outcome or subgroup title	No. of studies	No. of participants	Statistical method	Effect size
136.1 Final response	1	654	Odds Ratio (M‐H, Random, 95% CI)	0.78 [0.52, 1.19]

**Comparison 137:** Individual item vs. stem & leaf format

Outcome or subgroup title	No. of studies	No. of participants	Statistical method	Effect size
137.1 Final response	1	1500	Odds Ratio (M‐H, Random, 95% CI)	0.88 [0.70, 1.10]

**Comparison 138:** Horizontal vs. vertical orientation of response options

Outcome or subgroup title	No. of studies	No. of participants	Statistical method	Effect size
138.1 Final response	1	400	Odds Ratio (M‐H, Random, 95% CI)	3.12 [1.63, 5.96]

**Comparison 139:** Conventional vs. randomised response technique

Outcome or subgroup title	No. of studies	No. of participants	Statistical method	Effect size
139.1 First response	2	5830	Odds Ratio (M‐H, Random, 95% CI)	1.89 [1.69, 2.11]
139.2 Final response	4	7345	Odds Ratio (M‐H, Random, 95% CI)	1.52 [0.85, 2.72]

**Comparison 140:** Factual questions only vs. factual and attitudinal questions

Outcome or subgroup title	No. of studies	No. of participants	Statistical method	Effect size
140.1 Final response	1	1280	Odds Ratio (M‐H, Random, 95% CI)	1.34 [1.01, 1.77]

**Comparison 141:** Multi‐option vs. standard consent form

Outcome or subgroup title	No. of studies	No. of participants	Statistical method	Effect size
141.1 Final response	1	200	Odds Ratio (M‐H, Random, 95% CI)	0.91 [0.49, 1.68]

**Comparison 142:** Questions ordered by time period vs. other order

Outcome or subgroup title	No. of studies	No. of participants	Statistical method	Effect size
142.1 Final response	1	259	Odds Ratio (M‐H, Random, 95% CI)	1.48 [0.84, 2.59]

**Comparison 143:** Clinical outcome questions first vs. last

Outcome or subgroup title	No. of studies	No. of participants	Statistical method	Effect size
143.1 First Response	2	226	Odds Ratio (M‐H, Random, 95% CI)	2.62 [0.81, 8.50]
143.2 Final Response	2	226	Odds Ratio (M‐H, Random, 95% CI)	2.05 [0.99, 4.25]

**Comparison 144:** University sponsor/source vs. other

Outcome or subgroup title	No. of studies	No. of participants	Statistical method	Effect size
144.1 First response	4	5241	Odds Ratio (M‐H, Random, 95% CI)	1.35 [0.88, 2.08]
144.2 Final response	14	21628	Odds Ratio (M‐H, Random, 95% CI)	1.32 [1.13, 1.54]
144.3 e ‐ Login	2	3845	Odds Ratio (M‐H, Random, 95% CI)	0.80 [0.67, 0.96]
144.4 e ‐ Submission	2	3845	Odds Ratio (M‐H, Random, 95% CI)	0.84 [0.69, 1.01]

**Comparison 145:** Higher university sponsorship vs. lower

Outcome or subgroup title	No. of studies	No. of participants	Statistical method	Effect size
145.1 e ‐ Submission	2	658	Odds Ratio (M‐H, Random, 95% CI)	0.96 [0.63, 1.45]

**Comparison 146:** University printed envelope vs. plain

Outcome or subgroup title	No. of studies	No. of participants	Statistical method	Effect size
146.1 First response	1	500	Odds Ratio (M‐H, Random, 95% CI)	1.10 [0.77, 1.57]
146.2 Final response	1	500	Odds Ratio (M‐H, Random, 95% CI)	0.88 [0.61, 1.28]

**Comparison 147:** Sent or signed by more vs. less senior/well‐known person

Outcome or subgroup title	No. of studies	No. of participants	Statistical method	Effect size
147.1 First response	4	1526	Odds Ratio (M‐H, Random, 95% CI)	1.06 [0.85, 1.31]
147.2 Final response	11	5686	Odds Ratio (M‐H, Random, 95% CI)	1.05 [0.90, 1.23]
147.3 e ‐ Login	1	17346	Odds Ratio (M‐H, Random, 95% CI)	0.98 [0.91, 1.06]
147.4 e ‐ Submission	6	28162	Odds Ratio (M‐H, Random, 95% CI)	1.09 [0.96, 1.25]

**Comparison 148:** Pre‐contact by medical researcher vs. non medical researcher

Outcome or subgroup title	No. of studies	No. of participants	Statistical method	Effect size
148.1 Final response	2	924	Odds Ratio (M‐H, Random, 95% CI)	1.01 [0.55, 1.86]

**Comparison 149:** Q'aire sent by GP vs. by research group

Outcome or subgroup title	No. of studies	No. of participants	Statistical method	Effect size
149.1 First response	1	409	Odds Ratio (M‐H, Random, 95% CI)	1.49 [1.00, 2.24]
149.2 Final response	2	1106	Odds Ratio (M‐H, Random, 95% CI)	1.52 [0.73, 3.15]

**Comparison 150:** Male vs. female investigator or male vs. female signature

Outcome or subgroup title	No. of studies	No. of participants	Statistical method	Effect size
150.1 First response	1	204	Odds Ratio (M‐H, Random, 95% CI)	1.42 [0.76, 2.64]
150.2 Final response	2	3146	Odds Ratio (M‐H, Random, 95% CI)	1.07 [0.72, 1.58]
150.3 e ‐ Submission	2	720	Odds Ratio (M‐H, Random, 95% CI)	0.55 [0.38, 0.80]

**Comparison 151:** Assurance of confidentiality vs. none

Outcome or subgroup title	No. of studies	No. of participants	Statistical method	Effect size
151.1 Final response	1	25000	Odds Ratio (M‐H, Random, 95% CI)	1.33 [1.24, 1.42]

**Comparison 152:** Included statement that others had responded vs. no statement

Outcome or subgroup title	No. of studies	No. of participants	Statistical method	Effect size
152.1 First response	1	468	Odds Ratio (M‐H, Random, 95% CI)	1.12 [0.76, 1.65]
152.2 Final response	1	468	Odds Ratio (M‐H, Random, 95% CI)	1.12 [0.76, 1.65]
152.3 e ‐ Login	1	8586	Odds Ratio (M‐H, Random, 95% CI)	1.41 [1.28, 1.56]
152.4 e ‐ Submission	3	23777	Odds Ratio (M‐H, Random, 95% CI)	1.14 [0.83, 1.56]

**Comparison 153:** Choice to opt‐out from study vs. none

Outcome or subgroup title	No. of studies	No. of participants	Statistical method	Effect size
153.1 First response	1	515	Odds Ratio (M‐H, Random, 95% CI)	1.10 [0.77, 1.56]
153.2 Final response	5	5544	Odds Ratio (M‐H, Random, 95% CI)	0.96 [0.74, 1.25]

**Comparison 154:** Instructions given vs. not

Outcome or subgroup title	No. of studies	No. of participants	Statistical method	Effect size
154.1 Final response	1	2000	Odds Ratio (M‐H, Random, 95% CI)	0.89 [0.74, 1.06]

**Comparison 155:** Response deadline given vs. no deadline

Outcome or subgroup title	No. of studies	No. of participants	Statistical method	Effect size
155.1 First response	3	2575	Odds Ratio (M‐H, Random, 95% CI)	1.25 [0.93, 1.69]
155.2 Final response	6	5661	Odds Ratio (M‐H, Random, 95% CI)	1.00 [0.84, 1.19]
155.3 e ‐ Login	1	8586	Odds Ratio (M‐H, Random, 95% CI)	1.20 [1.07, 1.35]
155.4 e ‐ Submission	1	8586	Odds Ratio (M‐H, Random, 95% CI)	1.18 [1.03, 1.34]

**Comparison 156:** Mention of obligation to respond vs. none

Outcome or subgroup title	No. of studies	No. of participants	Statistical method	Effect size
156.1 First response	3	600	Odds Ratio (M‐H, Random, 95% CI)	1.61 [1.16, 2.22]
156.2 Final response	3	600	Odds Ratio (M‐H, Random, 95% CI)	1.61 [1.16, 2.22]

**Comparison 157:** Request for telephone number vs. none

Outcome or subgroup title	No. of studies	No. of participants	Statistical method	Effect size
157.1 First response	1	702	Odds Ratio (M‐H, Random, 95% CI)	1.00 [0.65, 1.54]
157.2 Final response	1	702	Odds Ratio (M‐H, Random, 95% CI)	1.00 [0.65, 1.54]

**Comparison 158:** Respond on questionnaire vs. on separate form

Outcome or subgroup title	No. of studies	No. of participants	Statistical method	Effect size
158.1 First response	1	200	Odds Ratio (M‐H, Random, 95% CI)	1.11 [0.59, 2.07]
158.2 Final response	1	200	Odds Ratio (M‐H, Random, 95% CI)	1.13 [0.57, 2.27]

**Comparison 159:** Mention of follow‐up contact vs. none

Outcome or subgroup title	No. of studies	No. of participants	Statistical method	Effect size
159.1 First response	5	4553	Odds Ratio (M‐H, Random, 95% CI)	1.16 [1.02, 1.33]
159.2 Final response	7	7053	Odds Ratio (M‐H, Random, 95% CI)	1.02 [0.91, 1.15]

**Comparison 160:** Explanation for non‐participation requested vs. not

Outcome or subgroup title	No. of studies	No. of participants	Statistical method	Effect size
160.1 First response	1	667	Odds Ratio (M‐H, Random, 95% CI)	0.97 [0.71, 1.32]
160.2 Final response	2	1907	Odds Ratio (M‐H, Random, 95% CI)	1.14 [0.83, 1.57]

**Comparison 161:** Time estimate for completion given vs. not

Outcome or subgroup title	No. of studies	No. of participants	Statistical method	Effect size
161.1 First response	1	600	Odds Ratio (M‐H, Random, 95% CI)	1.10 [0.76, 1.58]
161.2 Final response	1	600	Odds Ratio (M‐H, Random, 95% CI)	1.10 [0.76, 1.58]

**Comparison 162:** Detailed vs. brief cover letter

Outcome or subgroup title	No. of studies	No. of participants	Statistical method	Effect size
162.2 Final response	1	500	Odds Ratio (M‐H, Random, 95% CI)	1.08 [0.74, 1.58]
162.3 e‐Submission	1	1250	Odds Ratio (M‐H, Random, 95% CI)	3.26 [1.79, 5.94]

**Comparison 163:** Appeal vs. none

Outcome or subgroup title	No. of studies	No. of participants	Statistical method	Effect size
163.1 Final response	2	1251	Odds Ratio (M‐H, Random, 95% CI)	1.06 [0.79, 1.42]
163.2 e ‐ Login	3	4351	Odds Ratio (M‐H, Random, 95% CI)	0.94 [0.76, 1.16]
163.3 e ‐ Submission	4	5915	Odds Ratio (M‐H, Random, 95% CI)	1.07 [0.79, 1.47]

**Comparison 164:** Note requesting not to remove ID code vs. none

Outcome or subgroup title	No. of studies	No. of participants	Statistical method	Effect size
164.1 First response	1	100	Odds Ratio (M‐H, Random, 95% CI)	0.37 [0.14, 0.96]
164.2 Final response	1	100	Odds Ratio (M‐H, Random, 95% CI)	0.37 [0.14, 0.96]

**Comparison 165:** Request for participant signature vs. none

Outcome or subgroup title	No. of studies	No. of participants	Statistical method	Effect size
165.1 Final response	1	201	Odds Ratio (M‐H, Random, 95% CI)	1.19 [0.65, 2.18]

**Comparison 166:** Questionnaire endorsed vs. not endorsed

Outcome or subgroup title	No. of studies	No. of participants	Statistical method	Effect size
166.1 First response	1	395	Odds Ratio (M‐H, Random, 95% CI)	0.33 [0.15, 0.74]
166.2 Final response	1	395	Odds Ratio (M‐H, Random, 95% CI)	0.63 [0.43, 0.94]

**Comparison 167:** Veiled threat in follow‐up letter vs. none

Outcome or subgroup title	No. of studies	No. of participants	Statistical method	Effect size
167.1 First response	1	671	Odds Ratio (M‐H, Random, 95% CI)	2.09 [1.49, 2.93]
167.2 Final response	1	671	Odds Ratio (M‐H, Random, 95% CI)	2.09 [1.49, 2.93]

**Comparison 168:** Appeal stresses benefit to sponsor vs. other

Outcome or subgroup title	No. of studies	No. of participants	Statistical method	Effect size
168.1 First response	3	2376	Odds Ratio (M‐H, Random, 95% CI)	1.20 [0.88, 1.63]
168.2 Final response	8	10908	Odds Ratio (M‐H, Random, 95% CI)	0.99 [0.86, 1.13]

**Comparison 169:** Appeal stresses benefit to respondent vs. other

Outcome or subgroup title	No. of studies	No. of participants	Statistical method	Effect size
169.1 First response	1	1500	Odds Ratio (M‐H, Random, 95% CI)	0.97 [0.78, 1.21]
169.2 Final response	10	15159	Odds Ratio (M‐H, Random, 95% CI)	1.00 [0.85, 1.17]

**Comparison 170:** Appeal stresses benefit to society vs. other

Outcome or subgroup title	No. of studies	No. of participants	Statistical method	Effect size
170.1 First response	3	3906	Odds Ratio (M‐H, Random, 95% CI)	0.98 [0.82, 1.17]
170.2 Final response	14	36107	Odds Ratio (M‐H, Random, 95% CI)	1.07 [0.95, 1.20]
170.3 e‐Submission	3	3536	Odds Ratio (M‐H, Random, 95% CI)	1.35 [0.95, 1.91]

**Comparison 171:** Anonymous vs. not anonymous

Outcome or subgroup title	No. of studies	No. of participants	Statistical method	Effect size
171.1 Final response	2	2070	Odds Ratio (M‐H, Random, 95% CI)	0.96 [0.66, 1.39]

**Comparison 172:** Cover letter highlights salience vs. standard cover letter

Outcome or subgroup title	No. of studies	No. of participants	Statistical method	Effect size
172.1 First response	2	27119	Odds Ratio (M‐H, Random, 95% CI)	1.06 [0.75, 1.50]
172.2 Final response	2	27119	Odds Ratio (M‐H, Random, 95% CI)	1.06 [0.75, 1.50]

**Comparison 173:** Salient cover letter message on 1st mailing vs. follow‐up

Outcome or subgroup title	No. of studies	No. of participants	Statistical method	Effect size
173.1 First response	1	2180	Odds Ratio (M‐H, Random, 95% CI)	2.49 [1.82, 3.40]
173.3 Final response	1	2180	Odds Ratio (M‐H, Random, 95% CI)	2.49 [1.82, 3.40]

**Comparison 174:** Letter states responses being monitored vs. standard letter

Outcome or subgroup title	No. of studies	No. of participants	Statistical method	Effect size
174.1 First response	2	5202	Odds Ratio (M‐H, Random, 95% CI)	1.14 [1.01, 1.29]
174.2 Final response	1	4447	Odds Ratio (M‐H, Random, 95% CI)	1.15 [1.01, 1.31]

**Comparison 175:** Letter emphasises health promotion vs. harm prevention

Outcome or subgroup title	No. of studies	No. of participants	Statistical method	Effect size
175.1 First response	1	1418	Odds Ratio (M‐H, Random, 95% CI)	1.19 [0.83, 1.72]
175.2 Final response	1	1418	Odds Ratio (M‐H, Random, 95% CI)	1.19 [0.83, 1.72]

**Comparison 176:** Letter emphasises harm prevention vs. neutral message

Outcome or subgroup title	No. of studies	No. of participants	Statistical method	Effect size
176.1 First response	1	1410	Odds Ratio (M‐H, Random, 95% CI)	1.44 [0.98, 2.12]
176.2 Final response	1	1410	Odds Ratio (M‐H, Random, 95% CI)	1.44 [0.98, 2.12]

**Comparison 177:** Behaviour change letter vs. standard

Outcome or subgroup title	No. of studies	No. of participants	Statistical method	Effect size
177.1 First response	1	1192	Odds Ratio (M‐H, Random, 95% CI)	1.39 [1.08, 1.77]

**Comparison 178:** Participants told completion time 10 mins vs. 30 mins

Outcome or subgroup title	No. of studies	No. of participants	Statistical method	Effect size
178.1 e ‐ Login	1	2358	Odds Ratio (M‐H, Random, 95% CI)	0.55 [0.46, 0.66]
178.2 e ‐ Submission	1	2358	Odds Ratio (M‐H, Random, 95% CI)	1.25 [0.96, 1.64]

**Comparison 179:** Culturally sensitive letter vs. generic

Outcome or subgroup title	No. of studies	No. of participants	Statistical method	Effect size
179.1 Final response	1	1316	Odds Ratio (M‐H, Random, 95% CI)	1.00 [0.74, 1.34]

**Comparison 180:** Ethnically unidentifiable/white vs. other name

Outcome or subgroup title	No. of studies	No. of participants	Statistical method	Effect size
180.1 First response	1	600	Odds Ratio (M‐H, Random, 95% CI)	1.12 [0.79, 1.59]
180.2 Final response	5	5959	Odds Ratio (M‐H, Random, 95% CI)	1.07 [0.90, 1.27]

**Comparison 181:** Brown vs. white envelope

Outcome or subgroup title	No. of studies	No. of participants	Statistical method	Effect size
181.1 First response	3	5423	Odds Ratio (M‐H, Random, 95% CI)	1.44 [0.73, 2.83]
181.2 Final response	6	9756	Odds Ratio (M‐H, Random, 95% CI)	1.25 [0.86, 1.80]

**Comparison 182:** Check categories or specify numbers vs. check categories only

Outcome or subgroup title	No. of studies	No. of participants	Statistical method	Effect size
182.1 First response	1	740	Odds Ratio (M‐H, Random, 95% CI)	0.80 [0.60, 1.06]
182.2 Final response	1	740	Odds Ratio (M‐H, Random, 95% CI)	0.80 [0.60, 1.06]

**Comparison 183:** Shorter vs. longer questionnaire

Outcome or subgroup title	No. of studies	No. of participants	Statistical method	Effect size
183.1 First response	30	1347691	Odds Ratio (M‐H, Random, 95% CI)	1.13 [1.06, 1.21]
183.2 Final response	72	84954	Odds Ratio (M‐H, Random, 95% CI)	1.58 [1.40, 1.78]
183.3 e ‐ Login	2	3456	Odds Ratio (M‐H, Random, 95% CI)	1.79 [1.55, 2.07]
183.4 e ‐ Submission	5	12325	Odds Ratio (M‐H, Random, 95% CI)	1.51 [1.06, 2.16]

**Comparison 184:** Double postcard vs. one page

Outcome or subgroup title	No. of studies	No. of participants	Statistical method	Effect size
184.1 First response	1	600	Odds Ratio (M‐H, Random, 95% CI)	0.66 [0.48, 0.91]
184.2 Final response	1	600	Odds Ratio (M‐H, Random, 95% CI)	0.47 [0.34, 0.66]

**Comparison 185:** Questionnaire sent with supplement vs. alone

Outcome or subgroup title	No. of studies	No. of participants	Statistical method	Effect size
185.1 Final response	1	1795	Odds Ratio (M‐H, Random, 95% CI)	0.86 [0.70, 1.07]

**Comparison 186:** Extra questionnaire for relatives included vs. not

Outcome or subgroup title	No. of studies	No. of participants	Statistical method	Effect size
186.1 Final response	2	4943	Odds Ratio (M‐H, Random, 95% CI)	0.67 [0.60, 0.76]

**Comparison 187:** Consent form included vs. not

Outcome or subgroup title	No. of studies	No. of participants	Statistical method	Effect size
187.1 First response	1	414	Odds Ratio (M‐H, Random, 95% CI)	1.21 [0.81, 1.81]
187.2 Final response	1	414	Odds Ratio (M‐H, Random, 95% CI)	1.32 [0.89, 1.95]

## Characteristics of studies

### Characteristics of included studies [ordered by study ID]

**Table:** Aadahl 2003

***Study characteristics***		
Methods	Random allocation: random numbers using SAS	
Data	Random sample of 2543 men and women from the Danish Civil Registration System (Copenhagen County, Denmark)	
Comparisons	1. Lottery (25 euro voucher) 2. Control	
Outcomes	Response at 4 weeks	
Topic	Health: Self‐rated health, physical activity, and sociodemographics	
Mode of Administration	Postal	
Notes	Mean age: 40.5 years; mainly females	
***Risk of bias***		
**Item**	**Authors' judgement**	**Support for judgement**
Allocation concealment?	Unclear	B ‐ unclear

**Table:** Abdulaziz 2015

***Study characteristics***		
Methods	Randomly allocated by computer‐generated random numbers	
Data	178 emergency physicians, 178 geriatricians and 178 family physicians	
Comparisons	Incentive $10 coffee card with questionnaire vs no incentive	
Outcomes	Response after 3‐weekly reminders plus a final reminder sent by express courier	
Topic	Health services (treatment of elderly patients with minor injuries, i.e. lacerations, contusions, nonoperative fractures, etc.)	
Mode of Administration	Postal	
Notes	13 questions; 5 sections; 2 pages; single‐sided. The survey package consisted of a cover letter, a questionnaire, and a prepaid business reply mail envelope. Personalised prenotification (1 wk prior) and cover letters were used (all hand‐signed, plus physician name, area of expertise and affiliation were printed on the cover letter).	
***Risk of bias***		
**Item**	**Authors' judgement**	**Support for judgement**
Sequence generation	Yes	Random allocation: computer‐generated
Allocation concealment?	Yes	Random allocation: computer‐generated
Blinding of participants and personnel	Yes	Respondents were blind to the intervention and so would not be aware that others may have received a different or no incentive.
Blinding of outcome assessment	Yes	No outcome assessment used (counts of responses only)
Incomplete outcome data	Yes	Of the 534 physicians surveyed, 27 were not reachable because they had moved and 42 were ineligible as they were no longer practising or were not seeing elderly patients. Of the 465 eligible physicians, 265 completed and returned the survey (including the 12 of the 16 physicians from the local pilot survey) resulting in an overall response rate of 57%.
Selective reporting	Yes	The authors specified: "The primary outcome was the physician response rate", and no other outcomes were reported.
Other sources of bias	Yes	No grounds for concern about other sources of bias from reading the study report

**Table:** Adams 1982

***Study characteristics***		
Methods	Random allocation: method not specified	
Data	Full‐time students (Brigham Young University, US)	
Comparisons	1. 1‐page questionnaire 2. 3‐page questionnaire 3. 5‐page questionnaire	
Outcomes	Response at 3 months	
Topic	Non‐health	
Mode of Administration	Postal	
Notes	—	
***Risk of bias***		
**Item**	**Authors' judgement**	**Support for judgement**
Allocation concealment?	Unclear	B ‐ unclear

**Table:** Agarwal 2016

***Study characteristics***		
Methods	Computer‐generated (Excel) random sequence of numbers	
Data	Chairpersons of all academic Departments of Medicine in the United States	
Comparisons	Unconditional $5 coffee card ("for administrative assistants time") vs no incentive	
Outcomes	First mailing response, response after a follow‐up letter, and response after a follow‐up phone call	
Topic	Non‐health; how departments measure faculty productivity for the purpose of salary compensation	
Mode of Administration	Postal	
Notes	8 questions (23 tabulated closed‐ended items); "short questionnaire focusing on factual questions" Initially, we notified potential participants via the listserv of Chairs of Internal Medicine about the upcoming survey. Then, we mailed participants a survey package including a personalised cover letter, the survey questionnaire, and a pre‐addressed stamped return envelope. Two weeks after the initial mailing, we sent non‐responders a follow‐up letter. Two weeks later, we attempted to contact non‐responders by phone.	
***Risk of bias***		
**Item**	**Authors' judgement**	**Support for judgement**
Sequence generation	Yes	Random allocation: computer‐generated (Excel) random sequence of numbers
Allocation concealment?	Yes	Random allocation: Microsoft Excel to generate a random sequence of numbers
Blinding of participants and personnel	Unclear	Blinding of personnel not described. Participants were not blinded to intervention.
Blinding of outcome assessment	Yes	No outcome assessment used (counts of responses only)
Incomplete outcome data	Yes	Exclusions reported
Selective reporting	No	First response was not reported.
Other sources of bias	Yes	No grounds for concern about other sources of bias from reading the study report

**Table:** Akl 2005

***Study characteristics***		
Methods	Random allocation: method not specified	
Data	Residents and faculty of the University at Buffalo Internal Medicine Residency programme	
Comparisons	Electronic vs postal surveys	
Outcomes	Response at day 30 after 2 reminders (at days 10 and 20)	
Topic	Non‐health: academic issues	
Mode of Administration	Electronic	
Notes	28 questions (residents) or 23 (faculty) addressing demographic data, academic issues and survey mailing method. Except demographic questions, all had seven‐point Likert scale answer formats. Participants also had the option of providing narrative comments. The website that hosted the e‐survey generated automatic reminder emails.	
***Risk of bias***		
**Item**	**Authors' judgement**	**Support for judgement**
Sequence generation	Unclear	Random allocation: method not specified
Allocation concealment?	Unclear	Random allocation: method not specified
Blinding of participants and personnel	Unclear	Blinding of personnel not specified. Participants were not blinded.
Blinding of outcome assessment	Yes	No outcome assessment used (counts of responses only)
Incomplete outcome data	Yes	Exclusions reported
Selective reporting	Yes	Response after 2 reminders reported in full
Other sources of bias	Yes	No grounds for concern about other sources of bias from reading the study report

**Table:** Akl 2011

***Study characteristics***		
Methods	Computer‐generated (Excel)	
Data	Directors of Family Medicine and Internal Medicine residency programs in the US	
Comparisons	Mailing day (Friday vs. Monday) Response tracking (Yes vs. No)	
Outcomes	First response, response after a reminder at 5 weeks, response after a fax reminder at 9 weeks	
Topic	Health (training of residents in the implementation of clinical practice guidelines)	
Mode of Administration	Postal	
Notes	2 single‐sided pages with 15 questions about the curriculum, the characteristics of the programme director and the characteristics of the residency programme Initial invitation April 2007. To maximise response, the investigators included: university sponsorship, personalised cover letter, coloured ink, stamped return envelope, first‐class mailing, follow‐up mail, including a questionnaire in the follow‐up mail, and a short user‐friendly questionnaire with factual questions.	
***Risk of bias***		
**Item**	**Authors' judgement**	**Support for judgement**
Sequence generation	Yes	Random allocation: computer‐generated (Excel)
Allocation concealment?	Yes	Random allocation: computer‐generated (Excel)
Blinding of participants and personnel	Unclear	Blinding of personnel not described. Participants were not blinded to intervention.
Blinding of outcome assessment	Yes	No outcome assessment used (counts of responses only)
Incomplete outcome data	No	No attrition/exclusion reported
Selective reporting	No	Only reported numbers responding by the time of the 2nd reminder by intervention group
Other sources of bias	Yes	No grounds for concern about other sources of bias from reading the study report

**Table:** Albaum 1987

***Study characteristics***		
Methods	Random allocation: method not specified	
Data	Members of public employees credit group	
Comparisons	1. University source; open code 2. Research firm source; open code 3. Credit union source; open code 4. University source; no code 5. Research firm source; no code 6. Credit union source; no code Mailed reminder notification and follow‐up	
Outcomes	Response period not specified	
Topic	Not specified	
Mode of Administration	Postal	
Notes	—	
***Risk of bias***		
**Item**	**Authors' judgement**	**Support for judgement**
Allocation concealment?	Unclear	B ‐ unclear

**Table:** Albaum 1989

***Study characteristics***		
Methods	Random allocation: method not specified	
Data	Marketing managers of manufacturing firms (Denmark)	
Comparisons	1. Pre‐contact by letter; brochure explaining the study in depth 2. Pre‐contact; no brochure 3. No pre‐contact; brochure 4. No pre‐contact; no brochure	
Outcomes	Response within 67 days	
Topic	Non‐health: business, employment, and finance	
Mode of Administration	Postal	
Notes	—	
***Risk of bias***		
**Item**	**Authors' judgement**	**Support for judgement**
Allocation concealment?	Unclear	B ‐ unclear

**Table:** Allen 2016

***Study characteristics***		
Methods	Computer‐generated (a Perl script automatically randomised each participant to one of the versions of the survey. The only way to detect the presence of the Perl script on this page was to examine its source code)	
Data	A convenience sample of 498 adults recruited via face‐to‐face (e.g. flyers) and electronic (e.g. email messages and links on websites) methods. 60% were students. Most had access to broadband Internet.	
Comparisons	High uni sponsorship, mandatory questions vs low uni sponsorship, mandatory questions; high uni sponsorship, optional questions vs low uni sponsorship, optional questions	
Outcomes	Dropout ‐ whether or not the participant clicked the submit button at the end of the survey. Item non‐response ‐ the number of items (out of 78 and 65) that the participant provided a response to	
Topic	Internet piracy	
Mode of Administration	Electronic (web survey)	
Notes	78‐item questionnaire with a 10‐page online survey No compensation was provided for participation; however, participants were offered the opportunity to enter a prize draw as a token of appreciation for their time.	
***Risk of bias***		
**Item**	**Authors' judgement**	**Support for judgement**
Sequence generation	Yes	Perl script (Wright, 1996) automatically randomised each participant to one of the versions of the survey.
Allocation concealment?	Yes	Computer‐generated (a Perl script automatically randomised each participant to one of the versions of the survey. The only way to detect the presence of the Perl script on this page was to examine its source code).
Blinding of participants and personnel	Yes	The information page that preceded each version of the survey described the research as investigating factors influencing Internet piracy and survey completion behaviours, but did not explicitly mention the experimental manipulation. Personnel not involved in the process
Blinding of outcome assessment	Yes	No outcome assessment used (counts of responses only)
Incomplete outcome data	No	No mention of exclusions
Selective reporting	Yes	First response to survey was reported (no follow‐up)
Other sources of bias	Yes	No grounds for concern about other sources of bias from reading the study report

**Table:** Allen 2016a

***Study characteristics***		
Methods	Computer‐generated (a Perl script automatically randomised each participant to one of the versions of the survey. The only way to detect the presence of the Perl script on this page was to examine its source code)	
Data	A convenience sample of 159 adults was recruited via face‐to‐face (e.g. flyers) and electronic (e.g. email messages and links on websites) methods in mid‐2011. 70 percent were female.	
Comparisons	High uni sponsorship vs low uni sponsorship	
Outcomes	Dropout ‐ whether or not the participant clicked the submit button at the end of the survey. Item non‐response ‐ the number of items (out of 78 and 65) that the participant provided a response to	
Topic	Internet behaviour survey	
Mode of Administration	Electronic (web survey)	
Notes	65‐item questionnaire, 7‐page online survey Participants were not offered any incentives or compensation for participation.	
***Risk of bias***		
**Item**	**Authors' judgement**	**Support for judgement**
Sequence generation	Yes	Perl script (Wright, 1996) automatically randomised each participant to one of the versions of the survey.
Allocation concealment?	Yes	Computer‐generated (a Perl script automatically randomised each participant to one of the versions of the survey. The only way to detect the presence of the Perl script on this page was to examine its source code).
Blinding of participants and personnel	Yes	The information page that preceded each version of the survey described the research as investigating factors influencing Internet piracy and survey completion behaviours, but did not explicitly mention the experimental manipulation. Personnel not involved in the process
Blinding of outcome assessment	Yes	No outcome assessment used (counts of responses only)
Incomplete outcome data	No	No mention of exclusions
Selective reporting	Yes	First response to survey was reported (no follow‐up)
Other sources of bias	Yes	No grounds for concern about other sources of bias from reading the study report

**Table:** Alutto 1970

***Study characteristics***		
Methods	Random allocation: alternation	
Data	Members of a western New York State Chamber of Commerce	
Comparisons	1. Questionnaire sent to work address 2. Questionnaire sent to home address	
Outcomes	Response period not specified	
Topic	Non‐health: attitudes towards universities	
Mode of Administration	Postal	
Notes	—	
***Risk of bias***		
**Item**	**Authors' judgement**	**Support for judgement**
Allocation concealment?	No	C ‐inadequate

**Table:** Andreasen 1970

***Study characteristics***		
Methods	Random allocation: method not specified	
Data	New York State lottery winners	
Comparisons	1. Mimeographed salutation; follow‐up mimeographed 2. Mimeographed salutation; follow‐up handwritten 3. Hand‐typed salutation; follow‐up mimeographed 4. Hand‐typed salutation; follow‐up handwritten 5. Hand‐typed salutation using name of participant with handwritten postscript; follow‐up mimeographed 6. Hand‐typed salutation using name of participant with handwritten postscript; follow‐up handwritten Follow‐up letters sent after 3 weeks Follow‐up questionnaires sent after 4 weeks	
Outcomes	Response period not specified	
Topic	Non‐health	
Mode of Administration	Postal	
Notes	—	
***Risk of bias***		
**Item**	**Authors' judgement**	**Support for judgement**
Allocation concealment?	Unclear	B ‐ unclear

**Table:** Antoun 2017

***Study characteristics***		
Methods	Random allocation: method unspecified	
Data	Members of the LISS panel (Longitudinal Internet studies for the Social Sciences)	
Comparisons	Smartphone vs computer	
Outcomes	Started questionnaire; completed questionnaire	
Topic	Topics ranging from health to politics	
Mode of Administration	Electronic	
Notes	46 questions; 32 survey pages Survey invitations sent by email, normal LISS cash incentive provided for participation	
***Risk of bias***		
**Item**	**Authors' judgement**	**Support for judgement**
Sequence generation	Unclear	Random allocation: method unspecified
Allocation concealment?	Unclear	Random allocation: method unspecified
Blinding of participants and personnel	Unclear	No mention of blinding. Participants were not blinded.
Blinding of outcome assessment	Yes	No outcome assessment used (counts of responses only)
Incomplete outcome data	Yes	Participants who completed the Web questionnaire using a different device than they were assigned, perhaps because they failed to carefully read the instructions in their invitation letters or because they had a strong preference for one device over the other, were not included in the analysis.
Selective reporting	Yes	Responses after two periods were fully reported.
Other sources of bias	Yes	No grounds for concern about other sources of bias from reading the study report

**Table:** Arai 2016

***Study characteristics***		
Methods	Computer (Excel)‐generated random numbers	
Data	People registered to vote in 123 municipalities in Japan in September 2012	
Comparisons	Pen incentive (cheap vs. expensive; boxed vs. unboxed)	
Outcomes	Response period not specified	
Topic	Non‐health (whether levels of public goods and services had declined in rural communities that experienced municipal mergers in the recent past)	
Mode of Administration	Postal	
Notes	18 questions plus several additional demographic questions, one piece of A3 paper, with questions printed on both sides of the paper) Pre‐notification postcard; one week later posted questionnaire by direct mail service with return envelope and pen (high price/low, boxed/unboxed) or no pen	
***Risk of bias***		
**Item**	**Authors' judgement**	**Support for judgement**
Sequence generation	Yes	Computer (Excel)‐generated random numbers
Allocation concealment?	Yes	Computer (Excel)‐generated random numbers
Blinding of participants and personnel	Yes	Blinding of personnel not described. Participants were not blinded to intervention.
Blinding of outcome assessment	Yes	No outcome assessment used (counts of responses only)
Incomplete outcome data	Yes	Of the 12,309, we were able to contact 11,914 voters, as there were approximately 400 voters who were unreachable.
Selective reporting	Yes	No follow‐up mailings were sent, so only one response was possible (final response) and this was reported.
Other sources of bias	Yes	No grounds for concern about other sources of bias from reading the study report

**Table:** Arzheimer 1999

***Study characteristics***		
Methods	Random allocation: method not specified	
Data	A random sample of people listed on registration file, Hamburg, Germany	
Comparisons	1. Phonecard worth 6 Deutsch marks included 2. No incentive	
Outcomes	Response in first wave of mailing	
Topic	Non‐health: voting behaviour	
Mode of Administration	Postal	
Notes	—	
***Risk of bias***		
**Item**	**Authors' judgement**	**Support for judgement**
Allocation concealment?	Unclear	B ‐ unclear

**Table:** Asch 1996

***Study characteristics***		
Methods	Random allocation: method not specified	
Data	Subscribers to nursing who had previously indicated practice in critical care settings (US)	
Comparisons	1. Questionnaire sent 3 times 2. Questionnaire sent with postcard. If postcard was returned, participant received no follow‐up mailings.	
Outcomes	Response period not specified	
Topic	Health	
Mode of Administration	Postal	
Notes	Author contacted: no further information on allocation concealment	
***Risk of bias***		
**Item**	**Authors' judgement**	**Support for judgement**
Allocation concealment?	Unclear	B ‐ unclear

**Table:** Asch 1998

***Study characteristics***		
Methods	Random allocation: method not specified	
Data	Primary care physicians identified through the American Medical Association Physician Master File (US)	
Comparisons	1. $2 incentive sent with questionnaire 2. $5 incentive sent with questionnaire	
Outcomes	Response period not specified	
Topic	Health	
Mode of Administration	Postal	
Notes	Author contacted: no further information on allocation concealment	
***Risk of bias***		
**Item**	**Authors' judgement**	**Support for judgement**
Allocation concealment?	Unclear	B ‐ unclear

**Table:** Ashby 2011

***Study characteristics***		
Methods	Randomly‐generated numbers used to allocate participants to intervention and control groups	
Data	Participants who were taking part in an RCT investigating the effect of a food elimination diet based on the enzyme‐linked immunosorbent assay (ELISA) test for food sensitivity for the prevention of migraine	
Comparisons	Electronic reminder (SMS/email/both) vs no reminder	
Outcomes	Response within 39 days	
Topic	Health (migraine)	
Mode of Administration	Postal	
Notes	A two‐page, double‐sided questionnaire, which contained the HIT‐6 (Version 1.1) (Headache Impact Test) and Migraine Disability Assessment Test. These are questionnaires used to measure the impact headaches have on a participant's life. A return date was located on the front page of all questionnaires sent out to participants. Participants in the intervention group were sent an ER (electronic reminder) in the form of an SMS, email message, or both. The SMS text message read "You should have a new diary and a questionnaire by now. The questionnaire is important so please send it back with your first diary asap. Thanks." The content of the email reminder was "Thank you for taking part in the Migraine study. This is an automatic reminder. You should have received your 4‐week questionnaire by today and your second diary. It is important that we receive your first diary and your 4‐week questionnaire back as soon as possible."	
***Risk of bias***		
**Item**	**Authors' judgement**	**Support for judgement**
Sequence generation	Yes	Randomly‐generated numbers were used to list all participants by ID number. The first half of participants contained within this list (74 of 148) were allocated to the intervention group, whereas the remaining participants (74 of 148) were allocated to the control group.
Allocation concealment?	Yes	An independent data manager at the York Trials Unit was responsible for generating the allocation sequence and assigning participants into intervention and control groups.
Blinding of participants and personnel	Unclear	Blinding of personnel not described. Participants were not blinded to intervention.
Blinding of outcome assessment	Yes	No outcome assessment used (counts of responses only)
Incomplete outcome data	Yes	Participants excluded (n = 26/174). No email address or mobile telephone number was available.
Selective reporting	Yes	Response within 39 days was reported.
Other sources of bias	Yes	No grounds for concern about other sources of bias from reading the study report

**Table:** Ashing‐Giwa 2000

***Study characteristics***		
Methods	Random allocation: method not specified	
Data	A sample of African‐American and white American breast cancer patients diagnosed in 1989 and 1990	
Comparisons	1. $5 gift certificate sent with questionnaire 2. Promise of $5 gift certificate on response	
Outcomes	Response period not specified	
Topic	Health: quality of life in long‐term breast cancer survivors	
Mode of Administration	Postal	
Notes	Mean age: 63.6 years; mainly females	
***Risk of bias***		
**Item**	**Authors' judgement**	**Support for judgement**
Allocation concealment?	Unclear	B ‐ unclear

**Table:** Aveyard 2001

***Study characteristics***		
Methods	Random allocation: participants randomly sorted and then the first 150 given intervention	
Data	300 smokers selected randomly from 2 general practices in the United Kingdom	
Comparisons	1. Pencil and eraser sent with questionnaire 2. No pencil or eraser sent with questionnaire	
Outcomes	Response period not specified	
Topic	Health: recruitment for a smoking cessation programme	
Mode of Administration	Postal	
Notes	—	
***Risk of bias***		
**Item**	**Authors' judgement**	**Support for judgement**
Allocation concealment?	Unclear	B ‐ unclear

**Table:** Bachman 1987

***Study characteristics***		
Methods	Random allocation: method not specified	
Data	Taxpayers (Missouri)	
Comparisons	1. Student sponsor; social appeal 2. Student sponsor; help the sponsor appeal 3. Business sponsor; social appeal 4. Business sponsor; help the sponsor appeal 5. Commercial sponsor; social appeal 6. Commercial sponsor; help the sponsor appeal	
Outcomes	Response period not specified	
Topic	Non‐health: public attitude towards Missouri Department of Revenue	
Mode of Administration	Postal	
Notes	Author contacted: no further information on allocation concealment	
***Risk of bias***		
**Item**	**Authors' judgement**	**Support for judgement**
Allocation concealment?	Unclear	B ‐ unclear

**Table:** Bakan 2014

***Study characteristics***		
Methods	Unspecified	
Data	Prostate cancer survivors, 2 to 8 years post‐prostate cancer diagnosis	
Comparisons	Unconditional incentive ($5 food gift card), priority mail, incentive and priority mail or control	
Outcomes	Surveys returned within 48 days of the first survey mailing were considered responders to the first mailing, while surveys returned between 49 and 100 days after the first survey mailing were considered responders to the second mailing.	
Topic	Health outcomes in prostate cancer survivors (participant's demographic and medical characteristics, cancer treatment history, health, and quality of life)	
Mode of Administration	Postal	
Notes	10‐page questionnaire Survey package included 1st class pre‐notification letter. Mailings to all groups were addressed to the intended participant using a typed label, and included a personalised cover letter, the 10‐page survey, a resource sheet, and a postage‐paid, pre‐addressed return envelope.	
***Risk of bias***		
**Item**	**Authors' judgement**	**Support for judgement**
Sequence generation	Unclear	Random allocation ‐ method unspecified
Allocation concealment?	Unclear	Random allocation ‐ method unspecified
Blinding of participants and personnel	Unclear	Blinding of personnel not described. Participants were not blinded to intervention.
Blinding of outcome assessment	Yes	No outcome assessment used (counts of responses only)
Incomplete outcome data	Yes	Eight survivors declined study participation after the introductory letter, but prior to the first mailing, and were excluded from analyses. Nine surveys were returned as undeliverable, and 7 were returned with notification of patient death, yielding an evaluable sample of 976.
Selective reporting	Yes	Two mailings were conducted and both were reported.
Other sources of bias	Yes	No grounds for concern about other sources of bias from reading the study report

**Table:** Barker 1996

***Study characteristics***		
Methods	Random allocation: using random number generation	
Data	Individuals randomly selected from electoral registers (Solihull, UK)	
Comparisons	1. Question on sexual health included 2. Question on sexual health not included Reminder letter and questionnaire sent to non‐responders 3 weeks after initial mailing	
Outcomes	Response period not specified	
Topic	Health: sexual health	
Mode of Administration	Postal	
Notes	Method of allocation ascertained through contact with author	
***Risk of bias***		
**Item**	**Authors' judgement**	**Support for judgement**
Allocation concealment?	Yes	A ‐ adequate

**Table:** Barra 2016

***Study characteristics***		
Methods	Each participant was assigned an internal identification number. A list of the identification numbers were sent to a statistician external to the project who generated the control and intervention groups by random allocation.	
Data	Stroke patients discharged from the Stroke Unit of Akerhus University Hospital	
Comparisons	Pre‐contact by telephone vs. no pre‐contact	
Outcomes	Response at 45 days and response at 365 days	
Topic	Health services; patient's levels of contentment with, and benefit from, consumption of health services over the year following hospitalisation	
Mode of Administration	Postal	
Notes	Two questionnaires in the envelope (16 (patient) and 8 (caregiver) pages). Survey package included an envelope with handwritten address in the name of the patient and, where available, the name of the spouse. The envelope contained two questionnaires and a cover letter explaining the purpose of the study, ensuring confidentiality, and equipped with contact information for any queries. The cover letter was hand‐signed. The two questionnaires were made out for the patient and for a caregiver. 2 SAEs for each questionnaire.	
***Risk of bias***		
**Item**	**Authors' judgement**	**Support for judgement**
Sequence generation	Unclear	Random allocation: method unspecified
Allocation concealment?	Unclear	Random allocation: method unspecified
Blinding of participants and personnel	Unclear	Blinding of personnel not described. Participants could not be blinded to intervention.
Blinding of outcome assessment	Yes	No outcome assessment used (counts of responses only)
Incomplete outcome data	Yes	During the intervention period, it was discovered through the ongoing data collection that 6 patients (3 in each arm) suffered from known dementia, and these were excluded from the study. 10 patients (7 in the intervention group and 3 in the control group) died before 12 months had passed from discharge from hospital; these were also excluded. Furthermore, one patient in the intervention group was discovered to have been misdiagnosed with stroke, and one patient in the control group was discovered to be a very frail nursing home patient, who had been mis‐assessed as eligible for inclusion. 105 subjects in the intervention groupand 112 controls remained.
Selective reporting	Yes	Primary and secondary outcomes reported
Other sources of bias	Yes	No grounds for concern about other sources of bias from reading the study report

**Table:** Basnov 2009

***Study characteristics***		
Methods	Random allocation: method unspecified	
Data	Women referred for mammography (up to retirement age, 67), Denmark	
Comparisons	Electronic vs. postal	
Outcomes	Response period not specified	
Topic	Health (Short Form Health Survey and The Hospital Anxiety and Depression Scale)	
Mode of Administration	Electronic and postal	
Notes	Questionnaires: 17 pages and 119 items. The letter to women randomised to answer the internet version of the questionnaire included a guideline on how to answer the Web‐based questionnaire, whereas those allocated to the pen‐and‐paper version were asked to fill in and return the questionnaire in a prepaid envelope. The layout of the pen‐and‐paper version of SF‐36 was in accordance with the Danish manual of SF‐36 (Short Form‐36), and the layout of the internet version was as close to the paper version as possible.	
***Risk of bias***		
**Item**	**Authors' judgement**	**Support for judgement**
Sequence generation	Unclear	Random allocation: method unspecified
Allocation concealment?	Unclear	Random allocation: method unspecified
Blinding of participants and personnel	Unclear	Blinding of personnel not reported. Participants were not blinded.
Blinding of outcome assessment	Yes	No outcome assessment used (counts of responses only)
Incomplete outcome data	No	Exclusions not reported
Selective reporting	No	Only one response proportion was reported.
Other sources of bias	Yes	No grounds for concern about other sources of bias from reading the study report

**Table:** Bauer 2004

***Study characteristics***		
Methods	Random allocation	
Data	People who participated both in the COMMunity Intervention Trial for smoking cessation (COMMIT) as well as the follow‐up study	
Comparisons	1. US$ 2 Cheque 2. US$ 10 Cheque 3. No incentive	
Outcomes	Response period not specified	
Topic	Health: smoking cessation	
Mode of Administration	Postal	
Notes	Age: Mostly 48‐57 years; mainly females	
***Risk of bias***		
**Item**	**Authors' judgement**	**Support for judgement**
Allocation concealment?	Unclear	B ‐ unclear

**Table:** Bech 2009

***Study characteristics***		
Methods	Random allocation: method unspecified	
Data	Individuals aged 50‐75 years drawn from the Central National Register of the Danish population	
Comparisons	Web vs. postal	
Outcomes	Response period not specified	
Topic	Non‐health (mostly attitudinal questions about the design of nursing homes and associated facilities)	
Mode of Administration	Postal and electronic (web)	
Notes	35 closed‐ended categorical questions; postal 16 pages Questionnaire and SAE, or letter with web‐link	
***Risk of bias***		
**Item**	**Authors' judgement**	**Support for judgement**
Sequence generation	Unclear	Random allocation: method unspecified
Allocation concealment?	Unclear	Random allocation: method unspecified
Blinding of participants and personnel	Unclear	Blinding of personnel not mentioned. Participants were not blind to intervention.
Blinding of outcome assessment	Yes	No outcome assessment used (counts of responses only)
Incomplete outcome data	Yes	100 individuals from the original random draw were used in a pilot test of the Web‐based survey.
Selective reporting	Yes	Final response reported
Other sources of bias	Yes	No grounds for concern about other sources of bias from reading the study report

**Table:** Becker 2000a

***Study characteristics***		
Methods	Random allocation: method not specified	
Data	BSN alumni who graduated between 1989 and 1997 who had not returned an initial survey	
Comparisons	1. Second questionnaire sent as follow‐up 2. Postcard follow‐up (no second questionnaire)	
Outcomes	Response period not specified	
Topic	Non‐health: professional experience since graduation and perceptions of academic preparation	
Mode of Administration	Postal	
Notes	Additional data obtained from author	
***Risk of bias***		
**Item**	**Authors' judgement**	**Support for judgement**
Allocation concealment?	Unclear	B ‐ unclear

**Table:** Becker 2000b

***Study characteristics***		
Methods	Random allocation: method not specified	
Data	Alumni who graduated with a PhD or MSN between 1988 and 1997 who had not returned an initial survey	
Comparisons	1. Second questionnaire sent as follow‐up 2. Postcard follow‐up (no second questionnaire)	
Outcomes	Response period not specified	
Topic	Non‐health: professional experience since graduation and perceptions of academic preparation	
Mode of Administration	Postal	
Notes	Additional data obtained from author	
***Risk of bias***		
**Item**	**Authors' judgement**	**Support for judgement**
Allocation concealment?	Unclear	B ‐ unclear

**Table:** Beebe 2005a

***Study characteristics***		
Methods	Random allocation: using RANUNI function in SAS	
Data	Medicaid enrollees. Simple random sample	
Comparisons	1. US$ 2 bill 2. No incentive	
Outcomes	Response period not specified	
Topic	Health: racial and ethnic disparities in the use of health services and barriers to care	
Mode of Administration	Postal	
Notes	Method of allocation confirmed through contact with author	
***Risk of bias***		
**Item**	**Authors' judgement**	**Support for judgement**
Allocation concealment?	Yes	A ‐ adequate

**Table:** Beebe 2005b

***Study characteristics***		
Methods	Random allocation: using RANUNI function in SAS	
Data	Medicaid enrollees. American‐Indian	
Comparisons	1. US$ 2 bill 2. No incentive	
Outcomes	Response period not specified	
Topic	Health: racial and ethnic disparities in the use of health services and barriers to care	
Mode of Administration	Postal	
Notes	Method of allocation confirmed through contact with author	
***Risk of bias***		
**Item**	**Authors' judgement**	**Support for judgement**
Allocation concealment?	Yes	A ‐ adequate

**Table:** Beebe 2005c

***Study characteristics***		
Methods	Random allocation: using RANUNI function in SAS	
Data	Medicaid enrollees	
Comparisons	1. US$ 2 bill 2. No incentive	
Outcomes	Response period not specified	
Topic	Health: racial and ethnic disparities in the use of health services and barriers to care	
Mode of Administration	Postal	
Notes	Method of allocation confirmed through contact with author	
***Risk of bias***		
**Item**	**Authors' judgement**	**Support for judgement**
Allocation concealment?	Yes	A ‐ adequate

**Table:** Beebe 2005d

***Study characteristics***		
Methods	Random allocation: using RANUNI function in SAS	
Data	Medicaid enrollees. Somali	
Comparisons	1. US$ 2 bill 2. No incentive	
Outcomes	Response period not specified	
Topic	Health: racial and ethnic disparities in the use of health services and barriers to care	
Mode of Administration	Postal	
Notes	Method of allocation confirmed through contact with author	
***Risk of bias***		
**Item**	**Authors' judgement**	**Support for judgement**
Allocation concealment?	Yes	A ‐ adequate

**Table:** Beebe 2005e

***Study characteristics***		
Methods	Random allocation: using RANUNI function in SAS	
Data	Medicaid enrollees. Latino	
Comparisons	1. US$ 2 bill 2. No incentive	
Outcomes	Response period not specified	
Topic	Health: racial and ethnic disparities in the use of health services and barriers to care	
Mode of Administration	Postal	
Notes	Method of allocation confirmed through contact with author	
***Risk of bias***		
**Item**	**Authors' judgement**	**Support for judgement**
Allocation concealment?	Yes	A ‐ adequate

**Table:** Beebe 2005f

***Study characteristics***		
Methods	Random allocation: using RANUNI function in SAS	
Data	Medicaid enrollees. African‐American	
Comparisons	1. US$ 2 bill 2. No incentive	
Outcomes	Response period not specified	
Topic	Health: racial and ethnic disparities in the use of health services and barriers to care	
Mode of Administration	Postal	
Notes	Method of allocation confirmed through contact with author	
***Risk of bias***		
**Item**	**Authors' judgement**	**Support for judgement**
Allocation concealment?	Yes	A ‐ adequate

**Table:** Beebe 2007

***Study characteristics***		
Methods	Random allocation: using RANUNI function in SAS	
Data	Mayo clinic patients	
Comparisons	1. Small booklet (6 1/8 X 8 1/4") 2. Large booklet (8 1/4 X 11") 3. Blue booklet 8. White booklet	
Outcomes	Response period not specified	
Topic	Health: measure awareness and knowledge of privacy practices, and general opinions on privacy and health care	
Mode of Administration	Postal	
Notes	Method of allocation confirmed through contact with author; mean age: 57.6 years; mainly females	
***Risk of bias***		
**Item**	**Authors' judgement**	**Support for judgement**
Sequence generation	Yes	Random allocation: computer‐generated
Allocation concealment?	Yes	Random allocation: computer‐generated
Blinding of participants and personnel	Unclear	No mention of blinding of personnel. Participants were not blind to intervention.
Blinding of outcome assessment	Yes	No outcome assessment used (counts of responses only)
Incomplete outcome data	Yes	Two cases (one in the small/blue condition and one in the large/white condition) were excluded due to their being deceased or physically impaired, leaving roughly 500 cases assigned to each of the four conditions.
Selective reporting	Yes	Responses after all mailings were reported.
Other sources of bias	Yes	No grounds for concern about other sources of bias from reading the study report

**Table:** Beebe 2007a

***Study characteristics***		
Methods	Random allocation: method unspecified	
Data	500 primary care physicians and specialists (allergists, cardiologists, gastroenterologists, haematologists, nephrologists, pulmonologists, and rheumatologists) from the 12 different divisions within the Mayo Clinic Dept Of Medicine	
Comparisons	Mailed survey first, then web survey follow‐up to non‐respondents vs. web survey first, then mailed survey follow‐up to non‐respondents	
Outcomes	First response and response after one reminder	
Topic	Health services: designed to elicit opinions of Dept of Medicine members about the Mayo Clinic Electronic Medical Record (EMR)	
Mode of Administration	Postal and electronic	
Notes	The instrument contained approximately 20 Likert‐style items measuring general comfort using computers and various aspects of the Electronic Medical Record, including: level of use, adequacy of training, comfort level, helpfulness, satisfaction, and preference over paper medical records. The web survey design and layout was made to be as comparable to the paper version as possible. The mailed survey was sent to physician's offices via inter‐office mail and the web survey was distributed by an email message to the physician with an embedded link to the web survey. The reminder was sent in the medium corresponding to the initial mailing (electronically or via inter‐office mail). For the web condition, the reminder did not contain an embedded link to the web survey for comparability to its mailed counterpart. For those not responding to the initial mailing, the medium in which the follow‐up survey was sent was switched. Specifically, those non‐respondents in the web first condition received their follow‐up survey via mail; those in the mail first condition received the follow‐up via email. One week before the close of data collection, another email message was sent from the Dept of Medicine chair to all Dept of Medicine members encouraging them to respond to the survey.	
***Risk of bias***		
**Item**	**Authors' judgement**	**Support for judgement**
Sequence generation	Unclear	Random allocation: method unspecified
Allocation concealment?	Unclear	Random allocation: method unspecified
Blinding of participants and personnel	Unclear	Blinding of personnel not reported. Participants were not blind to intervention.
Blinding of outcome assessment	Yes	No outcome assessment used (counts of responses only)
Incomplete outcome data	Yes	11 cases were removed from the sample due to ineligibility or duplicate listing.
Selective reporting	Yes	First response and response after one reminder were both reported.
Other sources of bias	Yes	Supporting analysis and consideration of non‐response bias

**Table:** Beebe 2010

***Study characteristics***		
Methods	Computer‐generated (subjects were randomly assigned to four conditions using the RANUNI function in SAS v. 9.1. software according to a 2√ó 2 factorial design)	
Data	Residents of Olmsted County, Minnesota, aged 25‐65 years old registered in the Rochester Epidemiology Project (a medical records linkage system that captures medical data from electronic and paper medical and autopsy records for patients using the Mayo Clinic, Olmsted Medical Center, their affiliated hospitals, or one private practice provider)	
Comparisons	Shorter (2 pages) vs. longer (4 pages); pre‐notification letter vs. postcard	
Outcomes	Response after first mailing, response after one reminder (4 wks after first mailing)	
Topic	Health (gastrointestinal symptoms and functional gastrointestinal disorder (FGID) diagnoses)	
Mode of Administration	Postal	
Notes	The varied questionnaire length versions were based on the Talley Bowel Disease Questionnaire (Talley‐BDQ), a self‐report instrument to measure symptoms experienced over the past year. For this experiment, the full 16‐page Talley‐BDQ was shortened to a 4‐page version and then to a 2‐page version. The letter and postcard pre‐notifications contained the same text. Both identified the survey sponsor and described the purpose of the study. All subjects were sent either a letter or a postcard one week prior to mailing the survey package. A week after pre‐notification, a survey package was sent to all potential respondents. The package included a cover letter, a pen incentive, and one of two versions of the modified Talley‐BDQ. Reminder letters, along with another survey, were sent to non‐responders 4 weeks after the first mailing. Subjects who indicated at any point that they did not want to be contacted further were excluded from the study.	
***Risk of bias***		
**Item**	**Authors' judgement**	**Support for judgement**
Sequence generation	Yes	Random allocation: computer‐generated (RANUNI function in SAS v. 9.1. software)
Allocation concealment?	Yes	Random allocation: computer‐generated (RANUNI function in SAS v. 9.1. software)
Blinding of participants and personnel	Unclear	Blinding of personnel not described. Participants were not blinded to intervention.
Blinding of outcome assessment	Yes	No outcome assessment used (counts of responses only)
Incomplete outcome data	Yes	After randomisation, it was discovered that 120 cases were ineligible due to residence outside of Olmsted County or deceased status.
Selective reporting	Yes	Responses at all time points were reported.
Other sources of bias	Yes	No grounds for concern about other sources of bias from reading the study report

**Table:** Beebe 2018

***Study characteristics***		
Methods	Random allocation: method unspecified	
Data	Primary care clinicians, including physicians, nurse practitioners, and physician assistants, US	
Comparisons	Mixed‐mode (mail with web follow‐up) vs. mixed‐mode (web with mail follow‐up) Mail‐only vs. web‐only	
Outcomes	Response after 2 reminders	
Topic	Health (clinician knowledge, clinician barriers, and perceived parental barriers regarding human papillomavirus **[**HPV] vaccination).	
Mode of Administration	Postal & electronic	
Notes	Questionnaire not described. A cover letter on Mayo Clinic letter head detailing the survey purpose, the survey booklet, and a SAE. Body of email same; included link to web survey	
***Risk of bias***		
**Item**	**Authors' judgement**	**Support for judgement**
Sequence generation	Unclear	Random allocation: method unspecified
Allocation concealment?	Unclear	Random allocation: method unspecified
Blinding of participants and personnel	Unclear	Blinding of personnel not described. Participants were not blinded to intervention.
Blinding of outcome assessment	Yes	No outcomes were assessed (counts of responses only).
Incomplete outcome data	Yes	At the end of the study, two responders were found to be non‐clinicians and were excluded from the denominator.
Selective reporting	Yes	Response after 2 reminders reported in full
Other sources of bias	Yes	No grounds for concern about other sources of bias from reading the study report

**Table:** Bell 2004

***Study characteristics***		
Methods	Random allocation: computerised random number generation	
Data	People who had signed up for the 'Adventist Health Study‐2'	
Comparisons	1. Follow‐up phone call 2. No follow‐up phone call	
Outcomes	Response within approximately 6 months	
Topic	Health: dietary habits and risk of cancer	
Mode of Administration	Postal	
Notes	Mean age: 67.5 years; additional data obtained from author	
***Risk of bias***		
**Item**	**Authors' judgement**	**Support for judgement**
Allocation concealment?	Yes	A ‐ adequate

**Table:** Bell 2016

***Study characteristics***		
Methods	Random allocation: computer‐generated	
Data	Women aged 70‐75 years in screening of older women for prevention of fracture trial (SCOOP) recruited through GPs, UK	
Comparisons	Pen vs. no pen	
Outcomes	First response and response after one reminder (3 weeks)	
Topic	Health (self‐reported fracture of any bone in previous 12 months)	
Mode of Administration	Postal	
Notes	EQ‐5D (2 pages) and SF‐12 (3 pages). SCOOP participants received trial‐branded pen with the 60‐month follow‐up questionnaire, or received questionnaire alone. Reminder notices were sent approximately 18 days after the initial questionnaire. After continued non‐response, a follow‐up telephone call was administered approximately 12 days after the follow‐up reminder notice. After three attempts to contact participants by telephone, the participant was considered a non‐responder. Trial participants were followed up using postal questionnaires at 6 and 12 months post‐randomisation and then annually up to 5 years. The pen trial was initiated in the fifth year of follow‐up when participants were considered most at risk of becoming lost to follow‐up. The final response reported was the number that provided complete primary outcome data.	
***Risk of bias***		
**Item**	**Authors' judgement**	**Support for judgement**
Sequence generation	Yes	Random allocation: computer‐generated
Allocation concealment?	Yes	Random allocation: computer‐generated
Blinding of participants and personnel	Unclear	Blinding of personnel not described. Participants were not blinded to intervention.
Blinding of outcome assessment	Yes	No outcomes were assessed (counts of responses only).
Incomplete outcome data	Yes	Exclusions reported
Selective reporting	Yes	First response and response after one reminder (3 weeks) reported in full
Other sources of bias	Yes	No grounds for concern about other sources of bias from reading the study report

**Table:** Bellizzi 1986

***Study characteristics***		
Methods	Random allocation: random draw	
Data	People randomly selected from a local city telephone directory, USA	
Comparisons	1. $1 bill included with questionnaire 2. No incentive	
Outcomes	Response period not specified	
Topic	Non‐health: supermarket shopping	
Mode of Administration	Postal	
Notes	Method of allocation ascertained through contact with author	
***Risk of bias***		
**Item**	**Authors' judgement**	**Support for judgement**
Allocation concealment?	Unclear	B ‐ unclear

**Table:** Berdie 1973

***Study characteristics***		
Methods	Random allocation: method not specified	
Data	Staff of University of Minnesota, including professors of each rank	
Comparisons	1. 1‐page questionnaire 2. 2‐page questionnaire 3. 4‐page questionnaire	
Outcomes	Response within 20 days	
Topic	Non‐health: current social problems	
Mode of Administration	Postal	
Notes	—	
***Risk of bias***		
**Item**	**Authors' judgement**	**Support for judgement**
Allocation concealment?	Unclear	B ‐ unclear

**Table:** Bergen 1957

***Study characteristics***		
Methods	Random allocation: method not specified	
Data	Teachers in municipal elementary schools (Amsterdam)	
Comparisons	1. Pre‐notification 2. None	
Outcomes	Response period not specified	
Topic	Not specified	
Mode of Administration	Postal	
Notes	—	
***Risk of bias***		
**Item**	**Authors' judgement**	**Support for judgement**
Allocation concealment?	Unclear	B ‐ unclear

**Table:** Bergeson 2013

***Study characteristics***		
Methods	Random allocation: method unspecified	
Data	Patients receiving care at six clinics over a four‐month time frame. The clinics represented a mix of rural and urban locations drawn from a large clinic and hospital system in Minnesota.	
Comparisons	Web versus mail	
Outcomes	Response 88 days post‐visit (1 reminder in mail group, 2 reminders in electronic group)	
Topic	Health (patient experiences with care)	
Mode of Administration	Postal and electronic	
Notes	Consumer Assessment of Healthcare Providers and Systems (CAHPS) Clinician and Group hybrid survey, which includes 22 questions Patients from the clinic sites were randomised to receive either an emailed (web mode) or mailed (mail mode) invitation to complete the survey from the Executive Vice President of the organisation.	
***Risk of bias***		
**Item**	**Authors' judgement**	**Support for judgement**
Sequence generation	Unclear	Random allocation: method unspecified
Allocation concealment?	Unclear	Random allocation: method unspecified
Blinding of participants and personnel	Unclear	Blinding of personnel not specified. Participants were not blinded to intervention.
Blinding of outcome assessment	Yes	No outcome assessment used (counts of responses only)
Incomplete outcome data	No	No exclusions reported
Selective reporting	Yes	Response 88 days post‐visit (1 reminder in mail group, 2 reminders in electronic group) reported in full
Other sources of bias	Yes	No grounds for concern about other sources of bias from reading the study report

**Table:** Berk 1993

***Study characteristics***		
Methods	Random allocation: method not specified	
Data	Physicians	
Comparisons	1. $10 with first mailing; Follow‐up questionnaire and letter mentioning the incentive 2. No incentive with first mailing; follow‐up questionnaire with a $10 incentive and letter explaining the importance of the study 3. No mention of $10 incentive in either first or second mailing Follow‐up sent after 3 weeks	
Outcomes	Response period not specified	
Topic	Health: Cost‐effectiveness of 2 alternative methods of diagnosing allergies	
Mode of Administration	Postal	
Notes	—	
***Risk of bias***		
**Item**	**Authors' judgement**	**Support for judgement**
Allocation concealment?	Unclear	B ‐ unclear

**Table:** Berry 1987

***Study characteristics***		
Methods	Random allocation: method not specified	
Data	Physician members of the American Medical Association	
Comparisons	1. Cheque sent with first mailing 2. Promise of cheque with first mailing Non‐responders received a second mailing followed by a telephone call. If they no longer had the questionnaire, a third copy was sent.	
Outcomes	Response period not specified	
Topic	Health: evaluation of National Institute of Health Consensus Development Programme	
Mode of Administration	Postal	
Notes	Mean age: 48 years; mainly males	
***Risk of bias***		
**Item**	**Authors' judgement**	**Support for judgement**
Allocation concealment?	Unclear	B ‐ unclear

**Table:** Beydoun 2006

***Study characteristics***		
Methods	Random allocation: using computerised database	
Data	Women of reproductive age residing in Iowa county	
Comparisons	1. Unconditional $5 telephone card + conditional $25 check 2. Conditional $30 check	
Outcomes	Response period not specified	
Topic	Not specified	
Mode of Administration	Electronic: CATI	
Notes	Age: 18‐49 years; mainly females	
***Risk of bias***		
**Item**	**Authors' judgement**	**Support for judgement**
Allocation concealment?	Unclear	B ‐ unclear

**Table:** Bhandari 2003

***Study characteristics***		
Methods	Random allocation: computerised random number generation	
Data	Surgeon members of the Orthopaedic Trauma Association	
Comparisons	1. Survey endorsed in cover letter by 'opinion leaders' (high‐profile surgeons) 2. Survey not endorsed	
Outcomes	Response period not specified	
Topic	Health: evaluate surgeons opinions regarding optimal treatment of fractures of the tibial shaft	
Mode of Administration	Postal	
Notes	Mean age: 30.5 years; mainly males	
***Risk of bias***		
**Item**	**Authors' judgement**	**Support for judgement**
Allocation concealment?	Yes	A ‐ adequate

**Table:** Biner 1988

***Study characteristics***		
Methods	Random allocation: method not specified	
Data	Residents of a mid‐western US city	
Comparisons	1. Reactance appeal; $1 incentive 2. Reactance appeal; no incentive 3. No reactance appeal; $1 incentive 4. No reactance appeal; no incentive	
Outcomes	Response within 3 weeks	
Topic	Non‐health: residents' attitudes about the city	
Mode of Administration	Postal	
Notes	—	
***Risk of bias***		
**Item**	**Authors' judgement**	**Support for judgement**
Allocation concealment?	Unclear	B ‐ unclear

**Table:** Biner 1990

***Study characteristics***		
Methods	Random allocation: method not specified	
Data	Residents of a mid‐western US city	
Comparisons	1. $1 incentive; obligatory cover letter 2. $1 incentive; appreciative cover letter 3. $0.25 incentive; obligatory cover letter 4. $0.25 incentive; appreciative cover letter	
Outcomes	Response within 3 weeks	
Topic	Non‐health: residents' attitudes about the city	
Mode of Administration	Postal	
Notes	—	
***Risk of bias***		
**Item**	**Authors' judgement**	**Support for judgement**
Allocation concealment?	Unclear	B ‐ unclear

**Table:** Biner 1994

***Study characteristics***		
Methods	Random allocation: method not specified	
Data	Residents of a mid‐western US city	
Comparisons	1. Short questionnaire; $1 incentive and obligatory cover letter 2. Short questionnaire; $1 incentive and appreciative cover letter 3. Long questionnaire; $1 incentive and obligatory cover letter 4. Long questionnaire; $1 incentive and appreciative cover letter	
Outcomes	Response within 3 weeks	
Topic	Non‐health: residents' attitudes about the city	
Mode of Administration	Postal	
Notes	—	
***Risk of bias***		
**Item**	**Authors' judgement**	**Support for judgement**
Allocation concealment?	Unclear	B ‐ unclear

**Table:** Birnholtz 2004

***Study characteristics***		
Methods	Random allocation: computerised random number generation	
Data	Users of a collaboratory* for earthquake engineering research	
Comparisons	1. $5 bill 2. Gift certificate for Amazon.com	
Outcomes	Response period was 6 weeks	
Topic	Non‐health: participants research work and perception of a set of collaboration tools	
Mode of Administration	Electronic: web‐based	
Notes	Additional data obtained from the author * From the full‐text report: *“A collaboratory is a kind of laboratory without walls that connects scientists, instruments, and data via computer networks.”*	
***Risk of bias***		
**Item**	**Authors' judgement**	**Support for judgement**
Sequence generation	Yes	Random allocation: computerised random number generation
Allocation concealment?	Yes	Random allocation: computerised random number generation
Blinding of participants and personnel	Unclear	Blinding of personnel unspecified. Participants were not blinded.
Blinding of outcome assessment	Yes	No outcome assessment used (counts of responses only)
Incomplete outcome data	No	Exclusions not specified
Selective reporting	Yes	Response at 6 weeks was specified and reported.
Other sources of bias	Yes	No grounds for concern about other sources of bias from reading the study report

**Table:** Bjertnaes 2012

***Study characteristics***		
Methods	Random allocation: method unspecified	
Data	Women who gave birth at a university hospital	
Comparisons	Electronic and postal options for first and second mailing vs. electronic only first mailing and electronic and postal options for second mailing	
Outcomes	Response after 1 reminder (after 3 weeks)	
Topic	Health (experiences with healthcare services during pregnancy and birth, and after birth)	
Mode of Administration	Postal & electronic	
Notes	Questionnaires 16 pages; 141 questions. The information letter in the first survey request was the same for both groups, the only exception being the inclusion of an Internet link, user name, and password for electronic response in first mailing group. The second survey request was made to non‐respondents 3 weeks after posting the initial request, with both groups presented with an opportunity to answer via either post or the Internet.	
***Risk of bias***		
**Item**	**Authors' judgement**	**Support for judgement**
Sequence generation	Unclear	Random allocation: method unspecified
Allocation concealment?	Unclear	Random allocation: method unspecified
Blinding of participants and personnel	Unclear	Blinding of personnel not described. Participants were not blinded to intervention.
Blinding of outcome assessment	Yes	No outcomes were assessed (counts of responses only)
Incomplete outcome data	Yes	9 women failed to receive the survey due to wrong contact information.
Selective reporting	Yes	Response after 1 reminder (after 3 weeks) reported in full
Other sources of bias	Yes	Non‐response and other sources of bias considered

**Table:** Bjertnaes 2018

***Study characteristics***		
Methods	Random allocation: method unspecified	
Data	Parents of 2606 patients registered in the Norwegian Childhood Diabetes Registry	
Comparisons	Postal only vs. mixed‐mode (postal/electronic) vs. electronic only	
Outcomes	Response after 6 weeks (2 reminders)	
Topic	Health (parent experiences with hospital outpatient care for child and adolescent diabetes)	
Mode of Administration	Postal and electronic	
Notes	4 pages with 40 questions (5 sociodemographic, 35 topic), and an additional page for comments relating to experiences with the clinic or the questionnaire Parents of the 2606 patients registered in the Norwegian Childhood Diabetes Registry were sent an invitation letter to participate in the study. Two reminders were sent to non‐respondents. First reminder sent approximately 3 weeks after posting the initial request, the second approximately 3 weeks after first. Parents were randomised into the following data collection groups: i) postal with pen‐and‐paper questionnaire; ii) postal with pen‐and‐paper questionnaire and electronic response option; iii) postal with electronic response option.	
***Risk of bias***		
**Item**	**Authors' judgement**	**Support for judgement**
Sequence generation	Unclear	Random allocation: method unspecified
Allocation concealment?	Unclear	Random allocation: method unspecified
Blinding of participants and personnel	Unclear	Blinding of personnel not described. Participants were not blinded to intervention.
Blinding of outcome assessment	Yes	No outcomes were assessed (counts of responses only)
Incomplete outcome data	Yes	Due to wrong addresses, 80 patients were excluded.
Selective reporting	Yes	Responses before reminders and after 6 weeks (2 reminders) reported in full
Other sources of bias	Yes	No grounds for concern about other sources of bias from reading the study report

**Table:** Blass 1981

***Study characteristics***		
Methods	Random allocation: method not specified	
Data	Certified psychologists who did not respond to previous mailing of the questionnaire	
Comparisons	1. Consensus statement; threat of follow‐up 2. Consensus statement; no threat of follow‐up 3. No consensus statement; threat of follow‐up 4. No consensus statement; no threat of follow‐up	
Outcomes	Response period not specified	
Topic	Health: psychologist behaviour and attitudes towards continuing education	
Mode of Administration	Postal	
Notes	—	
***Risk of bias***		
**Item**	**Authors' judgement**	**Support for judgement**
Allocation concealment?	Unclear	B ‐ unclear

**Table:** Blass‐Wilhems 1982

***Study characteristics***		
Methods	Random allocation: random walk sampling	
Data	Not known	
Comparisons	1. Real postage stamp 2. Postage paid reply	
Outcomes	—	
Topic	Not specified	
Mode of Administration	Postal	
Notes	—	
***Risk of bias***		
**Item**	**Authors' judgement**	**Support for judgement**
Allocation concealment?	Unclear	B ‐ unclear

**Table:** Blomberg 1996

***Study characteristics***		
Methods	Random allocation: using a list of numbers between 1‐99 selected in a 'random' order by a researcher	
Data	Patients at the Stockholm County Council Institute of Psychotherapy	
Comparisons	1. Questionnaire with promise of lottery ticket on response 2. Questionnaire with lottery ticket enclosed 3. Questionnaire with no incentive All non‐respondents were sent reminders at 3, 6 and 10 weeks after initial mailing. At 14 weeks, non‐responders were sent a brief questionnaire regarding their reasons for not responding.	
Outcomes	Response within 12 weeks. Response period for second questionnaire not specified	
Topic	Health: psychotherapy measures ‐ General Symptom Index, Sense of Coherence, and Change in Target Complaints	
Mode of Administration	Postal	
Notes	Method of allocation ascertained through contact with author. Author confirmed allocation concealment.	
***Risk of bias***		
**Item**	**Authors' judgement**	**Support for judgement**
Allocation concealment?	Yes	A ‐ adequate

**Table:** Blumenberg 2019

***Study characteristics***		
Methods	Random allocation: method unspecified	
Data	Mothers of 5914 hospital births occurring in Pelotas (Brazil) in 1982 (Pelotas birth cohort study)	
Comparisons	Short vs. longer questionnaire high (every 15 days) vs. low (every 30 days) frequency of reminders (by email, Whatsapp, or Facebook message)	
Outcomes	Response after 2 reminders	
Topic	Health (alcohol consumption, physical activity, Internet use, violence, and smoking)	
Mode of Administration	Electronic (Web)	
Notes	Short questionnaires included 11 to 17 items (4 mins to complete); long questionnaires included 21 to 33 items (14 mins to complete) Applied 5 web‐questionnaires using 2 lengths (short and long version). Reminders were sent to non‐respondents using the same methods used for recruitment: emails, Whatsapp and Facebook messages; we sent, at most, two reminders for each questionnaire. If the registered individual had responded to that questionnaire, no further reminders were sent until a new questionnaire was published on the platform. Individuals allocated to the high‐frequency group received reminders every 15 days, while those in the low‐frequency group received reminders every 30 days. (Outcome data estimated from Fig 1a)	
***Risk of bias***		
**Item**	**Authors' judgement**	**Support for judgement**
Sequence generation	Unclear	Random allocation: method unspecified
Allocation concealment?	Unclear	Random allocation: method unspecified
Blinding of participants and personnel	Yes	The randomisation process was blind, and registered individuals did not know to which group they were allocated.
Blinding of outcome assessment	Yes	No outcomes were assessed (counts of responses only)
Incomplete outcome data	No	Exclusions not reported
Selective reporting	Yes	Response after 2 reminders reported in full
Other sources of bias	Yes	No grounds for concern about other sources of bias from reading the study report

**Table:** Blythe 1986

***Study characteristics***		
Methods	Random allocation: method not specified	
Data	Social workers	
Comparisons	1. Questionnaire with an opportunity to enter a lottery 2. Questionnaire without lottery offer Reminder letter sent after 1 week. Non‐respondents followed up at 3 and 7 weeks with offer to participate in the lottery.	
Outcomes	Response within 30 days	
Topic	Health: application of clinical evaluation tools in practice	
Mode of Administration	Postal	
Notes	—	
***Risk of bias***		
**Item**	**Authors' judgement**	**Support for judgement**
Allocation concealment?	Unclear	B ‐ unclear

**Table:** Bolt 2014

***Study characteristics***		
Methods	Random allocation: method unspecified	
Data	2500 physicians: 1000 medical specialists (250 internists, 150 cardiologists, 150 intensive care physicians, 150 neurologists, 150 pulmonologists, and 150 surgeons), 1100 GPs, and 400 elderly care physicians	
Comparisons	Long (4 double pages) vs short (2 double pages) questionnaire	
Outcomes	Response after 3 months and after 2 reminders	
Topic	Healthcare; physician questionnaire on end‐of‐life decision‐making	
Mode of Administration	Postal	
Notes	Four double‐page questionnaire (54 questions) or a shorter version consisting of two double pages (27 questions); all physicians were asked to return a response card stating whether they would participate and, if not, they were asked for their reason for not participating. After 1 month, physicians who had not returned this card received a reminder package containing the same questionnaire and a letter with a link to a questionnaire online. After 3 months, the remaining non‐responders received a reminder package containing a one double‐page questionnaire (18 questions). The original questionnaire consisted of four double pages (2727 to 2891 words, 54 to 58 questions, depending on specialty). The 1000 medical specialists were randomly assigned to receive the four double‐page questionnaire (2730 words, 54 questions) or a shorter version consisting of two double pages (1471 words, 27 questions) in which in‐depth questions and questions about the most recent request for euthanasia were omitted.	
***Risk of bias***		
**Item**	**Authors' judgement**	**Support for judgement**
Sequence generation	Unclear	Random allocation: method unspecified
Allocation concealment?	Unclear	Random allocation: method unspecified
Blinding of participants and personnel	Unclear	Blinding of personnel not described. Participants were not blinded to intervention.
Blinding of outcome assessment	Yes	No outcomes were assessed (counts of responses only)
Incomplete outcome data	Yes	Of the 2500 physicians, 231 were not traceable or not working in patient care in one of the included specialities.
Selective reporting	Yes	Response after 3 months and after 2 reminders reported in full
Other sources of bias	Yes	No grounds for concern about other sources of bias from reading the study report

**Table:** Bond 2020

***Study characteristics***		
Methods	Random allocation: computer random number generator	
Data	Breastfeeding women in a trial of an oral probiotic versus a placebo for preventing mastitis	
Comparisons	Mobile phone automated system (MPAS) vs. paper and email data collection (PEDC)	
Outcomes	Outcome data for the first 8 weeks and follow‐up questionnaires at 2, 6, and 12 months	
Topic	Health: prevention of mastitis	
Mode of Administration	Electronic	
Notes	Questionnaire not described in this report Short daily and slightly longer weekly questionnaires during the first 8 weeks following birth and longer follow‐up questionnaires at 2, 6, and 12 months [Arm 1]: The MPAS sent automated text messages to the participants' mobile phones with links to self‐administered web‐based surveys. Each survey link was embedded with the participants' unique identifier, enabling comparison across multiple surveys. A maximum of 2 automated reminders were integrated into the system if a participant did not respond after 3 days. [Arm 2]: The PEDC included a combination of an 8‐week calendar diary provided to participants at the time of trial entry and emailed links to weekly and follow‐up surveys. The calendar diaries were identified with the participant study number at the time of treatment randomisation, and the start date was manually entered. The A4‐size calendar was preserved with a waterproof coating, allowing for daily entries by pen. Participants were encouraged to hang the calendar in a prominent place at home. PEDC users were supplied with a stamped, addressed envelope to post the calendar back to the trial co‐ordinating centre at the end of the treatment phase.	
***Risk of bias***		
**Item**	**Authors' judgement**	**Support for judgement**
Sequence generation	Yes	Random allocation: computer‐generated random number
Allocation concealment?	Yes	Random allocation: computer‐generated random number
Blinding of participants and personnel	Unclear	Blinding of personnel not specified. Participants were not blind to intervention.
Blinding of outcome assessment	Yes	No outcome assessment used (counts of responses only)
Incomplete outcome data	No	Exclusions not reported
Selective reporting	Yes	Losses to follow‐up reported for both arms of the trial
Other sources of bias	Yes	No grounds for concern about other sources of bias from reading the study report

**Table:** Bonevski 2011

***Study characteristics***		
Methods	Random allocation: method unspecified	
Data	Random sample of 500 GPs practising in New South Wales (NSW), Australia	
Comparisons	Standard (Cancer Council NSW) invitation letter vs. standard invitation letter plus local Hunter urban Division of General Practice (GP access) cover letter signed by the chief executive	
Outcomes	First response, second response after reminder sent 4‐6 weeks after first mailing	
Topic	Healthcare: knowledge and attitudes about/practices around vitamin D of GPs	
Mode of Administration	Postal	
Notes	31 items about vitamin D printed on bright yellow paper. Study materials were mailed to all GPs. An information letter informed them of the study, and a note (with tea and coffee sachets attached) asked GPs to 'take a break from their busy day' and complete the survey. General practitioners were offered two options for completion and return of the survey: online, using a website address provided in the information letter; or hardcopy, using the paper version sent with the letter (with a reply‐paid envelope and a facsimile number included for return of the paper version of the survey). Completion of the survey constituted consent. General practitioners who completed the survey were offered the chance to receive a holiday voucher valued at $500. About 4‐6 weeks after the initial mailout, a reminder mailout was sent to GPs who had not responded to the survey.	
***Risk of bias***		
**Item**	**Authors' judgement**	**Support for judgement**
Sequence generation	Unclear	Random allocation: method unspecified
Allocation concealment?	Unclear	Random allocation: method unspecified
Blinding of participants and personnel	Unclear	Blinding of personnel not described. Participants were not blinded to intervention.
Blinding of outcome assessment	Yes	No outcome assessment used (counts of responses only)
Incomplete outcome data	Yes	Of the 1666 GPs selected in the sample, 52 were ineligible (retired, no longer working in general practice, or moved from practice).
Selective reporting	Yes	Responses to both mailouts and reminder telephone calls were reported in full.
Other sources of bias	Yes	No grounds for concern about other sources of bias from reading the study report

**Table:** Bonevski 2011a

***Study characteristics***		
Methods	Random allocation: method unspecified	
Data	Random sample of 500 GPs practising in New South Wales (NSW), Australia	
Comparisons	Telephone reminder vs. no reminder	
Outcomes	First response, second response after reminder sent 4‐6 weeks after first mailing	
Topic	Healthcare: knowledge and attitudes about/practices around vitamin D of GPs	
Mode of Administration	Postal	
Notes	31 items about vitamin D printed on bright yellow paper. Study materials were mailed to all GPs. An information letter informed them of the study, and a note (with tea and coffee sachets attached) asked GPs to 'take a break from their busy day' and complete the survey. General practitioners were offered two options for completion and return of the survey: online, using a website address provided in the information letter; or hardcopy, using the paper version sent with the letter (with a reply‐paid envelope and a facsimile number included for return of the paper version of the survey). Completion of the survey constituted consent. General practitioners who completed the survey were offered the chance to receive a holiday voucher valued at $500. About 4‐6 weeks after the initial mailout, a reminder mailout was sent to GPs who had not responded to the survey.	
***Risk of bias***		
**Item**	**Authors' judgement**	**Support for judgement**
Sequence generation	Unclear	Random allocation: method unspecified
Allocation concealment?	Unclear	Random allocation: method unspecified
Blinding of participants and personnel	Unclear	Blinding of personnel not described. Participants were not blinded to intervention.
Blinding of outcome assessment	Yes	No outcome assessment used (counts of responses only)
Incomplete outcome data	Yes	Of the 1666 GPs selected in the sample, 52 were ineligible (retired, no longer working in general practice, or moved from practice).
Selective reporting	Yes	Responses to both mailouts and reminder telephone calls were reported in full.
Other sources of bias	Yes	No grounds for concern about other sources of bias from reading the study report

**Table:** Boser 1990

***Study characteristics***		
Methods	Random allocation: alternation	
Data	Graduates from the College of Education of a major university (US)	
Comparisons	1. Questionnaire in folder format 2. Questionnaire in stapled format	
Outcomes	Response within 4 weeks	
Topic	Non‐health: teaching	
Mode of Administration	Postal	
Notes	—	
***Risk of bias***		
**Item**	**Authors' judgement**	**Support for judgement**
Allocation concealment?	No	C ‐ inadequate

**Table:** Bosnjak 2003

***Study characteristics***		
Methods	Random allocation: computerised random number generation	
Data	Local professional sales association members in the mid‐Atlantic US	
Comparisons	1. Unconditional $2 via PayPal 2. Conditional $2 3. Conditional prize draw (two $50 and four $25 prizes) 4. No incentive	
Outcomes	Response period not specified	
Topic	Non‐health: trends and concerns in real estates	
Mode of Administration	Electronic: Web survey	
Notes	Method of allocation ascertained through contact with author	
***Risk of bias***		
**Item**	**Authors' judgement**	**Support for judgement**
Allocation concealment?	Yes	A ‐ adequate

**Table:** Boulianne 2012

***Study characteristics***		
Methods	Random allocation: method unspecified	
Data	First‐year students at Grant MacEwan University, Alberta, Canada	
Comparisons	Unconditional $5 incentive vs. unconditional $10 incentive	
Outcomes	Break‐offs and completed surveys	
Topic	Non‐health: community attachment, membership in various groups, civic ethics, civic engagement, political engagement, and media usage	
Mode of Administration	Web survey	
Notes	The survey instrument included questions about community attachment, membership in various groups, civic ethics, civic engagement, political engagement, and media usage Pre notification letter with cash attached, hand‐signed and printed on university letterhead. Followed by email survey invitation	
***Risk of bias***		
**Item**	**Authors' judgement**	**Support for judgement**
Sequence generation	Unclear	Random allocation: method unspecified
Allocation concealment?	Unclear	Random allocation: method unspecified
Blinding of participants and personnel	Unclear	Blinding of personnel not reported. Participants were not blinded to intervention
Blinding of outcome assessment	Yes	No outcome assessment used (counts of responses only).
Incomplete outcome data	Yes	Exclusions clearly reported
Selective reporting	Yes	Breakoffs and completed surveys reported in full
Other sources of bias	Yes	No grounds for concern about other sources of bias from reading the study report

**Table:** Boyd 2015

***Study characteristics***		
Methods	Random allocation: computer‐generated (random number generator within Stata)	
Data	Index children of the Avon Longitudinal Study of Parents and Children cohort (ALSPAC)	
Comparisons	Prior‐notification postcard vs. no contact; standard vs. professionally designed consent pack; phone (phone call, SMS, or email) reminder vs. postcard reminder	
Outcomes	Response after 3 weeks; response after reminder	
Topic	Health: consent to be enrolled into the ALSPAC cohort study including linking their health records	
Mode of Administration	Postal	
Notes	A single consent form (which asked multiple, separate, consent questions). Information materials split into: a covering letter, a four‐page summary leaflet, a 32‐page detailed booklet supported by detailed Web pages. A prepaid envelope was included.	
***Risk of bias***		
**Item**	**Authors' judgement**	**Support for judgement**
Sequence generation	Yes	Random allocation: computer‐generated (random number generator within Stata)
Allocation concealment?	Yes	Random allocation: computer‐generated (random number generator within Stata)
Blinding of participants and personnel	Yes	At the point of allocation, the participants were identified using the pseudonym key and K.H. administered the distribution and collation of the prior‐notification and the information pack interventions.
Blinding of outcome assessment	Yes	No outcome asessment used (counts of responses only)
Incomplete outcome data	Yes	Where there was evidence that the mailing was not received (the mailing being returned "addressee not known, by the postal service or participants requesting replacement mailings), we excluded the individual from the analysis [n = 548 (2.4%)], resulting in an analysable sample of 1950.
Selective reporting	Yes	Response after one reminder was reported.
Other sources of bias	Yes	No grounds for concern about other sources of bias from reading the study report

**Table:** Boyle 2012

***Study characteristics***		
Methods	Random allocation: method unspecified	
Data	People aged between 40 and 79 randomly selected from the electoral roll, Australia	
Comparisons	Unconditional $2 lottery scratch ticket vs. nothing	
Outcomes	Non‐responders were sent a reminder letter 3 weeks after the initial invitation.	
Topic	Health: colorectal cancer	
Mode of Administration	Postal	
Notes	Consent form for the Western Australian Bowel Health Study (WABOHS) (presumably 2 or 3 questions) Invitation letter, an information sheet about the study, a consent form, and a reply‐paid envelope Participants who consented to take part in the WABOHS were then sent a study pack that consisted of two questionnaires and a DNA self‐extraction kit. These participants were randomised further into three groups when sent their study packs, ignoring their initial allocation to unconditional $2 lottery scratch ticket. Group 0 did not get a lottery scratch ticket, Group 1 received a $2 lottery scratch ticket, and Group 2 was promised a $2 lottery scratch ticket on return of their completed study pack.	
***Risk of bias***		
**Item**	**Authors' judgement**	**Support for judgement**
Sequence generation	Unclear	Method of randomisation unspecified
Allocation concealment?	Unclear	Method of randomisation unspecified
Blinding of participants and personnel	Unclear	Blinding of personnel not specified. Participants were not blind to the intervention.
Blinding of outcome assessment	Yes	No outcome asessment used (counts of responses only)
Incomplete outcome data	Yes	4 participants were reported ineligible.
Selective reporting	Yes	All response outcomes were reported.
Other sources of bias	Yes	No grounds for concern about other sources of bias from reading the study report

**Table:** Bradshaw 2020

***Study characteristics***		
Methods	Random allocation: computer‐generated	
Data	Parents who had given consent for their infant to be randomised into the BEEP host trial	
Comparisons	Pre‐contact by SMS at 3 m, 6 m, 12 m and 18 m vs. no SMS	
Outcomes	Response 28 days after SMS	
Topic	Health (effect of applying emollient on development of eczema in high‐risk infants)	
Mode of Administration	Electronic (web‐link sent by email) with postal option	
Notes	Questionnaires had 14 items on one page. Electronic (web‐link sent by email) with postal alternative for parents who did not want to respond online Due to the lower than expected completion of questionnaires, the protocol was amended in May 2016 to allow members of the host trial team to telephone participants where questionnaires had not yet been completed but were still active. Text messages or emails were also sent by the trial team when they were unable to reach participants by telephone.	
***Risk of bias***		
**Item**	**Authors' judgement**	**Support for judgement**
Sequence generation	Yes	Random allocation: computer‐generated
Allocation concealment?	Yes	Random allocation: computer‐generated
Blinding of participants and personnel	Yes	Participants were informed in the host trial information sheet about the randomized controlled study within a trial (SWAT) for SMS notification for questionnaires and timing of the voucher for the 24‐month visit but were not informed at the time of their randomisation of their allocated groups for the SWAT.
Blinding of outcome assessment	Yes	No outcomes were assessed (counts of responses only)
Incomplete outcome data	No	Exclusions not reported
Selective reporting	Yes	Response data reported for all follow‐up times
Other sources of bias	Yes	No grounds for concern about other sources of bias from reading the study report

**Table:** Bray 2017

***Study characteristics***		
Methods	Randomisation of participants was carried out using the runiform function in Stata with anonymous identifiers.	
Data	Young people in the Avon Longitudinal Study of Parents and Children (ALSPAC) birth cohort study, UK	
Comparisons	Online questionnaire vs. choice (online or postal)	
Outcomes	The primary outcome measure was the number of questionnaires returned (with at least one question answered) in each arm of the trial. For the purposes of this analysis, return rates were calculated 30 weeks after the initial mailing.	
Topic	Health: gambling, self‐harm, employment, education & training, and tobacco & alcohol	
Mode of Administration	Electronic and postal	
Notes	The paper version of the questionnaire was an A5 booklet of 44 pages; the online questionnaire was designed to be as similar as possible to the paper questionnaire, acknowledging that certain functions, such as skip statements, would be different because participants would be automatically directed to the next relevant question. This also affected the numbering of questions, which would have been non‐consecutive if not allowed to be dynamic in the online version. Generally, the number of questions per page was less in the online version than in the paper version. The first reminder (after 3 weeks) was by email, but if an email address was not recorded, then a text was used. If neither electronic means of contact was possible, then a postcard reminder was used. Two weeks later, a different mode of reminder was sent (unless a postcard had already been used in which case no other reminder was sent, to avoid multiple reminders of the same method). Eight weeks after the initial letter, a reminder letter was sent to all non‐respondents, with a paper copy of the questionnaire enclosed. Another brief reminder (email, text, or postcard) was sent if necessary 2 weeks later. A Facebook reminder was also posted 12 weeks after the original letter. Finally, a phone call reminder was attempted for all those who had not responded between 12 and 19 weeks after the initial letter was sent out. Initially, an attempt was made to contact the participant using the landline number held on record. If this was not successful and if a mobile number was also recorded, then this number was also rung. If neither attempt was successful, then a message was left on both landline and mobile phones, wherever possible. If contact was made with a family member but the participant was not at home, then a message was left. A reminder was sent only if a paper questionnaire had not been received from the participant and the online submission was not complete (i.e. at least one section of the questionnaire had not been submitted online). The exception to this was the reminder at 8 weeks enclosing a paper questionnaire; this was only sent if a paper questionnaire had not been received and an online submission had not been initiated (i.e. no sections had been submitted online). If a participant contacted ALSPAC to request a paper questionnaire at any stage in the process, then this was recorded and one was sent. A £10 Amazon voucher was offered to compensate participants for their time and to encourage response.	
***Risk of bias***		
**Item**	**Authors' judgement**	**Support for judgement**
Sequence generation	Yes	Random allocation: conputer‐generated (runiform function in Stata with anonymous identifiers)
Allocation concealment?	Yes	Random allocation: conputer‐generated (runiform function in Stata with anonymous identifiers)
Blinding of participants and personnel	Yes	ALSPAC staff compiling the completion statistics were blinded to group assignment.
Blinding of outcome assessment	Yes	No outcome assessment used (counts of responses only)
Incomplete outcome data	Yes	It was discovered that 13 participants in the ‘‘online first’’ arm (0.3%) and 17 in the ‘‘choice’’arm (0.4%) were not eligible (e.g. due to changes in family circumstances or requests not to be contacted) and were therefore not mailed.
Selective reporting	Yes	Responses after 30 weeks from initial mailing were reported in full.
Other sources of bias	Yes	No grounds for concern about other sources of bias from reading the study report

**Table:** Bredart 2002

***Study characteristics***		
Methods	Random allocation: method not specified	
Data	Breast cancer patients undergoing surgical treatment within the surgery department of the European Institute of Oncology in Milan	
Comparisons	1. Questionnaire sent 2 weeks after hospital discharge 2. Questionnaire sent 3 months after hospital discharge	
Outcomes	Response period not specified	
Topic	Health	
Mode of Administration	Postal	
Notes	—	
***Risk of bias***		
**Item**	**Authors' judgement**	**Support for judgement**
Allocation concealment?	Unclear	B ‐ unclear

**Table:** Breen 2010

***Study characteristics***		
Methods	Random allocation: method unspecified	
Data	People aged 18‐37 years who did not respond to three mailings of a postal community survey investigating alcohol consumption and harm in rural NSW, Australia	
Comparisons	Follow‐up phone call vs. no further contact	
Outcomes	Response after 9 weeks (after 2 reminders)	
Topic	Health: alcohol consumption and harm	
Mode of Administration	Postal	
Notes	Questionnaire not described. Questionnaire sent with a personalised cover letter; returns were tracked; reminders sent after 1 week and after 2 weeks.	
***Risk of bias***		
**Item**	**Authors' judgement**	**Support for judgement**
Sequence generation	Unclear	Random allocation: method unspecified
Allocation concealment?	Unclear	Random allocation: method unspecified
Blinding of participants and personnel	Unclear	Blinding of personnel not described. Participants were not blinded to intervention.
Blinding of outcome assessment	Yes	No outcome assessment used (counts of responses only)
Incomplete outcome data	Yes	12 participants were overseas, away, or had died.
Selective reporting	Yes	Responses after reminders and intervention phone call reported in full
Other sources of bias	Yes	No grounds for concern about other sources of bias from reading the study report

**Table:** Brehaut 2006

***Study characteristics***		
Methods	Random allocation: computerised random number generation	
Data	Members of the Canadian Association of Emergency Physicians	
Comparisons	1. Single‐sided print format 2. Double‐sided print format 3. Known sender recognition 4. Unknown sender recognition	
Outcomes	Response period not specified	
Topic	Health: clinical decision rules	
Mode of Administration	Postal	
Notes	Method of allocation ascertained through contact with author	
***Risk of bias***		
**Item**	**Authors' judgement**	**Support for judgement**
Sequence generation	Yes	Random allocation: computerised random number generation
Allocation concealment?	Yes	Random allocation: computerised random number generation
Blinding of participants and personnel	Unclear	Blinding of personnel not described. Participants were not blinded to intervention.
Blinding of outcome assessment	Yes	No outcome assessment used (counts of responses only)
Incomplete outcome data	Yes	Our initial sample consisted of 400 names. Of those, one was ineligible due to an address outside of Canada..
Selective reporting	Yes	Response after three mailings was reported.
Other sources of bias	Yes	No grounds for concern about other sources of bias from reading the study report

**Table:** Brems 2006

***Study characteristics***		
Methods	Random allocation: alternation	
Data	Licenced healthcare professionals from Alaska and New Mexico in the US	
Comparisons	1. First‐class mail 2. Priority mail	
Outcomes	Response period not specified	
Topic	Health: range of treatment used by physical and behavioural healthcare providers, ethical issues	
Mode of Administration	Postal	
Notes	—	
***Risk of bias***		
**Item**	**Authors' judgement**	**Support for judgement**
Allocation concealment?	No	C ‐ inadequate

**Table:** Brennan 1991

***Study characteristics***		
Methods	Random allocation: method not specified	
Data	People listed on 1 of the 57 electoral rolls representing the main urban centres, New Zealand	
Comparisons	1. Control ‐ no incentive 2. 20c coin with first mailing 3. 50c coin with first mailing 4. $1 note with first mailing 5. 20c coin with second mailing 6. 50c coin with second mailing 7. $1 note with second mailing 8. Entry into prize draw for $200 cash offered with each mailout 9. Entry into prize draw for $200 gift voucher offered with each mailout	
Outcomes	Response within 21 days of the third mailing (49 days after initial mailing)	
Topic	Non‐health: personal finance status	
Mode of Administration	Postal	
Notes	Randomisation confirmed through contact with author	
***Risk of bias***		
**Item**	**Authors' judgement**	**Support for judgement**
Allocation concealment?	Unclear	B ‐ unclear

**Table:** Brennan 1992a

***Study characteristics***		
Methods	Random allocation: method not specified	
Data	People listed on a financial service company's 'hot prospect' list	
Comparisons	1. $0.50 incentive 2. No incentive	
Outcomes	Response period not specified	
Topic	Non‐health: finances and shopping behaviours	
Mode of Administration	Postal	
Notes	—	
***Risk of bias***		
**Item**	**Authors' judgement**	**Support for judgement**
Allocation concealment?	Unclear	B ‐ unclear

**Table:** Brennan 1992b

***Study characteristics***		
Methods	Random allocation: method not specified	
Data	People listed on the electoral roll (New Zealand)	
Comparisons	1. $0.50 incentive 2. No incentive	
Outcomes	Response period not specified	
Topic	Non‐health: finances and shopping behaviours	
Mode of Administration	Postal	
Notes	—	
***Risk of bias***		
**Item**	**Authors' judgement**	**Support for judgement**
Allocation concealment?	Unclear	B ‐ unclear

**Table:** Brennan 1992c

***Study characteristics***		
Methods	Random allocation: method not specified	
Data	People listed on the electoral roll (New Zealand)	
Comparisons	1. $0.50 incentive 2. No incentive	
Outcomes	Response period not specified	
Topic	Non‐health: finances and shopping behaviours	
Mode of Administration	Postal	
Notes	—	
***Risk of bias***		
**Item**	**Authors' judgement**	**Support for judgement**
Allocation concealment?	Unclear	B ‐ unclear

**Table:** Brennan 1993a

***Study characteristics***		
Methods	Random allocation: method not specified	
Data	Dairy and beef farmers	
Comparisons	1. $0.50 coin with first mailing 2. $1 coin with first mailing 3. $1 lottery ticket with first mailing 4. No incentive	
Outcomes	Response period not specified	
Topic	Non‐health: marketing	
Mode of Administration	Postal	
Notes	—	
***Risk of bias***		
**Item**	**Authors' judgement**	**Support for judgement**
Allocation concealment?	Unclear	B ‐ unclear

**Table:** Brennan 1993b

***Study characteristics***		
Methods	Random allocation: method not specified	
Data	People listed on the electoral roll (New Zealand)	
Comparisons	1. $0.50 coin with first mailing 2. $1 coin with first mailing 3. Promise that $1 would be donated to a charity for each valid return (in each of 3 mailings) 4. No incentive	
Outcomes	Response period not specified	
Topic	Non‐health: marketing	
Mode of Administration	Postal	
Notes	—	
***Risk of bias***		
**Item**	**Authors' judgement**	**Support for judgement**
Allocation concealment?	Unclear	B ‐ unclear

**Table:** Brennan 2009

***Study characteristics***		
Methods	Random allocation: method unspecified	
Data	New Zealand residents selected from the electoral roll	
Comparisons	Unconditional chocolate incentive vs. none	
Outcomes	Response after two follow‐ups	
Topic	Non‐health (Reality TV)	
Mode of Administration	Postal	
Notes	Eight‐page questionnaire on the topic of Reality TV was in the form of an A4 international standard size booklet (A3 international standard equivalent folded), in one of four bold colours (green, blue, red, and purple). A reply‐paid envelope was provided whenever a questionnaire was supplied. The one‐page cover letter was printed on the university letterhead. It explained what the survey was about and why it was being conducted; assured respondents that the survey was simple and not trying to trick them or sell them anything; explained how they were selected; stressed confidentiality; explained the purpose of the ID number; informed them of the reply‐paid envelope; acknowledged that they were busy and expressed appreciation in advance for their assistance. Shortened forms of this letter, emphasising the importance of a response and appreciation of their assistance, were used in the follow‐up mailouts. All envelopes had the university logo printed in the top left‐hand side corner. The incentive was a small (45 mm x 55 mm x 6 mm), flat, individually foil‐wrapped high‐quality milk chocolate bar. This chocolate remains solid at temperatures considerably higher than those likely to be encountered by the mail, so there was minimal likelihood of the chocolate melting. The colours of the packaging (blue and gold) co‐incidentally matched the colours of the university letterhead used for the cover letters, making for an appealing package. The incentive (when used) was attached to the letter with double‐sided adhesive tape, and the following statement was added to the letter: 'As a token of our appreciation, we hope you will enjoy the attached sample of Whittaker's chocolate.' Whittaker's is a long‐established, well‐known New Zealand chocolate manufacturer.	
***Risk of bias***		
**Item**	**Authors' judgement**	**Support for judgement**
Sequence generation	Unclear	Random allocation: method unspecified
Allocation concealment?	Unclear	Random allocation: method unspecified
Blinding of participants and personnel	Unclear	Blinding of personnel not described. Participants were not blinded to intervention.
Blinding of outcome assessment	Yes	No outcomes were assessed (counts of responses only)
Incomplete outcome data	No	Exclusions not reported
Selective reporting	Yes	Response after two follow‐ups reported in full
Other sources of bias	Yes	No grounds for concern about other sources of bias from reading the study report

**Table:** Bright 2002

***Study characteristics***		
Methods	Random allocation: method not specified	
Data	US Marinas	
Comparisons	1. Offer of entry into a prize draw and summary of study results on return of questionnaire 2. No incentive offered	
Outcomes	Response period not specified	
Topic	Non‐health: perceptions of decision‐makers at US Marinas	
Mode of Administration	Postal	
Notes	—	
***Risk of bias***		
**Item**	**Authors' judgement**	**Support for judgement**
Allocation concealment?	Unclear	B ‐ unclear

**Table:** Brook 1978

***Study characteristics***		
Methods	Random allocation: alternation	
Data	People who had been interviewed when shopping (Southampton, UK)	
Comparisons	1. First‐class stamp out; first‐class stamp return 2. First‐class stamp out; second‐class stamp return 3. Second‐class stamp out; first‐class stamp return 4. Second‐class stamp out; second‐class stamp return 5. First‐class stamp out; second‐class business reply return 6. Second‐class stamp out; second‐class business reply return	
Outcomes	Response within 2 weeks	
Topic	Non‐health: marketing	
Mode of Administration	Postal	
Notes	—	
***Risk of bias***		
**Item**	**Authors' judgement**	**Support for judgement**
Allocation concealment?	No	C ‐ inadequate

**Table:** Brookes 2018a

***Study characteristics***		
Methods	Random allocation: computer‐generated schedule	
Data	130 consenting living patients who had undergone oesophagectomy from 2 UK hospital trusts	
Comparisons	Patient‐reported outcomes (PROs) first and clinical outcomes last vs. clinical outcomes first and PROs last Brookes 2018a in patients; Brookes 2018b in healthcare professionals	
Outcomes	Response after reminders	
Topic	Health (outcomes following oesophageal cancer surgery)	
Mode of Administration	Postal	
Notes	8 PROs and 30 clinical outcomes Patients were posted an invitation letter and information leaflet and asked to return a consent form indicating willingness to participate in the study. Consenting patients were then sent a postal survey with an SAE.	
***Risk of bias***		
**Item**	**Authors' judgement**	**Support for judgement**
Sequence generation	Yes	Random allocation: computer‐generated schedule
Allocation concealment?	Yes	Allocation schedule was used (within a mail‐merge) to automatically generate the allocated survey for each participant
Blinding of participants and personnel	Unclear	Blinding of personnel not described. Participants were not blinded to intervention.
Blinding of outcome assessment	Yes	No outcomes were assessed (counts of responses only)
Incomplete outcome data	No	Exclusions not reported
Selective reporting	Yes	Response after reminders reported in full
Other sources of bias	Yes	No grounds for concern about other sources of bias from reading the study report

**Table:** Brookes 2018b

***Study characteristics***		
Methods	Random allocation: computer‐generated schedule	
Data	96 relevant health professionals (from Association of Upper GastroIntestinal Surgeons of Great Britain and Ireland)	
Comparisons	Patient‐reported outcomes (PROs) first and clinical outcomes last vs. clinical outcomes first and PROs last Brookes 2018a in patients; Brookes 2018b in healthcare professionals	
Outcomes	Response after reminders	
Topic	Health (outcomes following oesophageal cancer surgery)	
Mode of Administration	Postal	
Notes	8 PROs and 30 clinical outcomes Professionals were notified by email about the study and sent a survey through the post with an SAE. Reminders were sent via post or email (for patients and professionals, respectively) to non‐responders.	
***Risk of bias***		
**Item**	**Authors' judgement**	**Support for judgement**
Sequence generation	Yes	Random allocation: computer‐generated schedule
Allocation concealment?	Yes	Allocation schedule was used (within a mail‐merge) to automatically generate the allocated survey for each participant
Blinding of participants and personnel	Unclear	Blinding of personnel not described. Participants were not blinded to intervention.
Blinding of outcome assessment	Yes	No outcomes were assessed (counts of responses only)
Incomplete outcome data	No	Exclusions not reported
Selective reporting	Yes	Response after reminders reported in full
Other sources of bias	Yes	No grounds for concern about other sources of bias from reading the study report

**Table:** Brown 1965

***Study characteristics***		
Methods	Random allocation: method not specified	
Data	Non‐paediatric physicians (US)	
Comparisons	1. 2‐page questionnaire (first page was letter with 2 cystic fibrosis screening questions; second page asked for details of patients seen) 2. 1‐page cover letter and postcard with 2 cystic fibrosis screening questions	
Outcomes	Response period not specified	
Topic	Health: cystic fibrosis	
Mode of Administration	Postal	
Notes	—	
***Risk of bias***		
**Item**	**Authors' judgement**	**Support for judgement**
Allocation concealment?	Unclear	B ‐ unclear

**Table:** Brown 1975

***Study characteristics***		
Methods	Random allocation: method not specified	
Data	Officers and enlisted men	
Comparisons	1. Pre‐notification; randomised enquiry method 2. No pre‐notification; randomised enquiry method 3. Pre‐notification; conventional method 4. No pre‐notification; conventional method	
Outcomes	Response period not specified	
Topic	Health: assessment of illicit drug use	
Mode of Administration	Postal	
Notes	—	
***Risk of bias***		
**Item**	**Authors' judgement**	**Support for judgement**
Allocation concealment?	Unclear	B ‐ unclear

**Table:** Bruce 2000

***Study characteristics***		
Methods	Random allocation: method not specified	
Data	People randomly selected from households in central Sydney (Australia) who had agreed to participate during an earlier phone interview	
Comparisons	1. Phone call reminder to non‐responders 2. Postcard reminder to non‐responders	
Outcomes	Response period not specified	
Topic	Health: colorectal cancer screening	
Mode of Administration	Postal	
Notes	—	
***Risk of bias***		
**Item**	**Authors' judgement**	**Support for judgement**
Allocation concealment?	Unclear	B ‐ unclear

**Table:** Brøgger 2007

***Study characteristics***		
Methods	Random allocation: method not specified	
Data	Permanent residents of Norway	
Comparisons	1. Postal plus optional Internet response 2. Only postal response	
Outcomes	Response period not specified	
Topic	Health: respiratory survey (to establish the occurrence and risk factors for asthma and allergies)	
Mode of Administration	Postal	
Notes	Mean age: 30.7 years; mainly males	
***Risk of bias***		
**Item**	**Authors' judgement**	**Support for judgement**
Sequence generation	Unclear	Random allocation: method unspecified
Allocation concealment?	Unclear	Random allocation: method unspecified
Blinding of participants and personnel	Yes	Participants were blinded to the randomised nature of the study.
Blinding of outcome assessment	Yes	No outcome assessment used (counts of responses only)
Incomplete outcome data	Yes	Exclusions and attrition reported
Selective reporting	Yes	Outcomes reported fully
Other sources of bias	Yes	No grounds for concern about other sources of bias from reading the study report

**Table:** Buchman 1982

***Study characteristics***		
Methods	Random allocation: alternation	
Data	Certified public accountants	
Comparisons	1. Conventional questionnaire 2. Randomised response technique employed for each question	
Outcomes	Response period not specified	
Topic	Non‐health: audit procedures	
Mode of Administration	Postal	
Notes	—	
***Risk of bias***		
**Item**	**Authors' judgement**	**Support for judgement**
Allocation concealment?	No	C ‐ inadequate

**Table:** Burgess 2012

***Study characteristics***		
Methods	Random allocation: method unspecified	
Data	Practice managers of general practices in two inner London boroughs, UK	
Comparisons	Electronic reminders vs. electronic and postal reminders	
Outcomes	Response after 2 reminders (after 1 and 2 weeks)	
Topic	Health; evaluation of implementation of NHS Health Checks	
Mode of Administration	Electronic and mixed‐mode (electronic and postal)	
Notes	33 questions. (i) computer‐delivered survey only with email reminders after 1 and 2 weeks; (ii) sequential, mixed‐mode with computer‐delivered survey followed by both email and postal reminder letters after 1 and 2 weeks. The postal reminder included a paper copy of the questionnaire.	
***Risk of bias***		
**Item**	**Authors' judgement**	**Support for judgement**
Sequence generation	Unclear	Random allocation: method unspecified
Allocation concealment?	Unclear	Random allocation: method unspecified
Blinding of participants and personnel	Unclear	Blinding not reported. Participants could not be blinded.
Blinding of outcome assessment	Yes	No outcome assessment used (counts of responses only)
Incomplete outcome data	No	Exclusions not reported
Selective reporting	Yes	Responses after reminders fully reported
Other sources of bias	Yes	No grounds for concern about other sources of bias from reading the study report

**Table:** Burns 1980

***Study characteristics***		
Methods	Random allocation: method not specified	
Data	A random sample of bank and savings and loan chief executive officers, USA	
Comparisons	1. No incentive; no follow‐up 2. 25 cent incentive; no follow‐up 3. 25 cent incentive; follow‐up postcard sent 10 days after initial mailing 4. No incentive; follow‐up postcard sent 10 days after initial mailing	
Outcomes	Response period not specified	
Topic	Non‐health: commercial population	
Mode of Administration	Postal	
Notes	Method of allocation ascertained through contact with author	
***Risk of bias***		
**Item**	**Authors' judgement**	**Support for judgement**
Allocation concealment?	Unclear	B ‐ unclear

**Table:** Buttle 1997

***Study characteristics***		
Methods	Random allocation	
Data	Managing Directors of the companies listed on the DTI Quality Assurance Register 1995	
Comparisons	1. Questionnaires printed on white paper 2. Questionnaires printed on yellow paper	
Outcomes	Response period not specified	
Topic	Non‐health: perceived costs and benefits of ISO 9000 in certified organisations	
Mode of Administration	Postal	
Notes	—	
***Risk of bias***		
**Item**	**Authors' judgement**	**Support for judgement**
Allocation concealment?	Unclear	B ‐ unclear

**Table:** Cabana 2000

***Study characteristics***		
Methods	Random allocation: method not specified	
Data	Paediatricians listed as general paediatricians in the American Medical Association master file	
Comparisons	1. Survey logo on questionnaire only 2. Survey logo on cover letter, return envelope, questionnaire and outer envelope	
Outcomes	Response period not specified	
Topic	Health	
Mode of Administration	Postal	
Notes	—	
***Risk of bias***		
**Item**	**Authors' judgement**	**Support for judgement**
Allocation concealment?	Unclear	B ‐ unclear

**Table:** Campbell 1990

***Study characteristics***		
Methods	Random allocation: method not specified	
Data	People listed on the electoral roll (Southampton, UK)	
Comparisons	1. Participants told replies would be anonymous 2. Participants told replies would not be anonymous and would be followed up after 3 weeks	
Outcomes	Response period not specified	
Topic	Health: knowledge of AIDS	
Mode of Administration	Postal	
Notes	—	
***Risk of bias***		
**Item**	**Authors' judgement**	**Support for judgement**
Allocation concealment?	Unclear	B ‐ unclear

**Table:** Camunas 1990

***Study characteristics***		
Methods	Random allocation: method not specified	
Data	Nurses who were members of the New York State Nurses Association	
Comparisons	Experiment 1: 1. Questionnaire, cover letter and brochure 2. Questionnaire, cover letter with an invitation to join the Nursing Association and brochure 3. Questionnaire and cover letter only Experiment 2: 1. Questionnaire, cover letter and $1 bill incentive 2. Questionnaire and cover letter only Questionnaires were colour‐coded for each group. No pre‐contact or follow‐up used	
Outcomes	Response period not specified	
Topic	Health: professional membership behaviour	
Mode of Administration	Postal	
Notes	—	
***Risk of bias***		
**Item**	**Authors' judgement**	**Support for judgement**
Allocation concealment?	Unclear	B ‐ unclear

**Table:** Carling 2004

***Study characteristics***		
Methods	Random allocation: method not specified	
Data	Journalists in the health field	
Comparisons	1. International postal vouchers 2. No International postal vouchers	
Outcomes	Response period not specified	
Topic	Health: barriers and facilitators to high‐quality health journalism	
Mode of Administration	Postal	
Notes	—	
***Risk of bias***		
**Item**	**Authors' judgement**	**Support for judgement**
Allocation concealment?	Unclear	B ‐ unclear

**Table:** Carpenter 1974

***Study characteristics***		
Methods	Random allocation: method not specified	
Data	People listed on an automobile registration list (Arizona, US)	
Comparisons	1. Least personalised questionnaire 2. Somewhat personalised questionnaire 3. Most personalised questionnaire 4. Control group	
Outcomes	Response period not specified	
Topic	Non‐health: migration behaviour	
Mode of Administration	Postal	
Notes	—	
***Risk of bias***		
**Item**	**Authors' judgement**	**Support for judgement**
Allocation concealment?	No	C ‐ inadequate

**Table:** Carpenter 1977

***Study characteristics***		
Methods	Random allocation: alternation	
Data	Heads of households and their spouses selected from the annually compiled auto registration list	
Comparisons	1. 2 questionnaires allocated per household 2. 1 questionnaire allocated per household	
Outcomes	Response within 7 weeks	
Topic	Not specified	
Mode of Administration	Postal	
Notes	—	
***Risk of bias***		
**Item**	**Authors' judgement**	**Support for judgement**
Allocation concealment?	No	C ‐ inadequate

**Table:** Cartwright 1986

***Study characteristics***		
Methods	Random allocation: method not specified	
Data	Recent mothers	
Comparisons	Experiment 1: 1. Long questionnaire about facts and attitudes 2. Medium questionnaire about facts and attitudes 3. Short questionnaire about facts and attitudes 4. Long questionnaire about facts only 5. Medium questionnaire about facts only 6. Short questionnaire about facts only Experiment 2: 1. Government department sponsor (OPCS, UK) 2. University sponsor (Institute for Social Studies in Medical Care) Experiment 3: 1. Asked to tick boxes in response 2. Asked to ring pre‐codes in response	
Outcomes	—	
Topic	Health: maternity	
Mode of Administration	Postal	
Notes	—	
***Risk of bias***		
**Item**	**Authors' judgement**	**Support for judgement**
Allocation concealment?	Unclear	B ‐ unclear

**Table:** Cartwright 1987

***Study characteristics***		
Methods	Random allocation: systematic division	
Data	Elderly people from the electoral registers in Woodford and Wanstead, London and Blackley, Manchester, UK	
Comparisons	1. Shorter questionnaire (2 questions) 2. Longer questionnaire (5 questions)	
Outcomes	—	
Topic	Health: medication and relationship with GPs	
Mode of Administration	Postal	
Notes	—	
***Risk of bias***		
**Item**	**Authors' judgement**	**Support for judgement**
Allocation concealment?	No	C ‐ inadequate

**Table:** Chan 2003

***Study characteristics***		
Methods	Random allocation: computerised random number generation	
Data	Chinese Medicine Practitioners registered with the Chinese Medicine Council of Hong Kong	
Comparisons	1. HK $20 2. HK $30 3. No Incentives	
Outcomes	Response period not specified	
Topic	Health: knowledge, attitudes, and practices on computers and computer use in clinical practice	
Mode of Administration	Postal	
Notes	Age: Mostly 40‐59 years; mainly males	
***Risk of bias***		
**Item**	**Authors' judgement**	**Support for judgement**
Allocation concealment?	Unclear	B ‐ unclear

**Table:** Chan 2018

***Study characteristics***		
Methods	Random allocation: computer‐generated list	
Data	Registered medical doctors of the Medical Council of Hong Kong	
Comparisons	Standard invitation letter vs. invitation with motivational message vs. motivational message on follow‐up reminder	
Outcomes	Response after 2 weeks	
Topic	Health (evaluation of physicians' attitudes toward people with mental illness)	
Mode of Administration	Mixed‐mode (postal or web)	
Notes	Questionnaire 20 mins; 5 pages; double‐sided Invitation letter with URL mailed, with consent form, questionnaire and SAE. Conditional coupon (HK$50) offered on returned questionnaire Promotion‐focused message (emphasising the promotion of mental healthcare through survey participation), prevention‐focused message (emphasising the reduction of psychiatric stigma by survey participation) or neutral message. Message on 1st mailing or on reminder	
***Risk of bias***		
**Item**	**Authors' judgement**	**Support for judgement**
Sequence generation	Yes	Random allocation: computer‐generated list
Allocation concealment?	Yes	Random allocation: computer‐generated list
Blinding of participants and personnel	Yes	Individuals invited to participate in the survey were blinded to the design of this randomized trial.
Blinding of outcome assessment	Yes	No outcomes were assessed (counts of responses only)
Incomplete outcome data	No	Exclusions not reported
Selective reporting	Yes	Response after 2 weeks reported in full
Other sources of bias	Yes	No grounds for concern about other sources of bias from reading the study report

**Table:** Chebat 1991

***Study characteristics***		
Methods	Random allocation: method not specified	
Data	The Quebec population within the legal driving age	
Comparisons	1. Pre‐notification; non‐monetary incentive 2. Pre‐notification; no incentive 3. No pre‐notification; non‐monetary incentive 4. No pre‐notification; no incentive	
Outcomes	Response period not specified	
Topic	Non‐health: driving behaviour	
Mode of Administration	Postal	
Notes	Method of allocation and concealment ascertained through contact with author	
***Risk of bias***		
**Item**	**Authors' judgement**	**Support for judgement**
Allocation concealment?	Yes	A ‐ adequate

**Table:** Chen 1984

***Study characteristics***		
Methods	Random allocation: method not specified	
Data	Students from 4 Taipei Universities	
Comparisons	1. Long questionnaire ‐ 5 pages 2. Short questionnaire ‐ 2 pages 3. High salient topic ‐ cutting‐class behaviours in undergraduates 4. Low salient topic ‐ cutting‐class behaviours in PhD students 5. High authority researcher ‐ university professor with a PhD in Psychology 6. Low authority researcher ‐ student from the Psychology department	
Outcomes	Response period within 10 days	
Topic	Non‐health: class‐cutting behaviour	
Mode of Administration	Postal	
Notes	Language of publication is Chinese.	
***Risk of bias***		
**Item**	**Authors' judgement**	**Support for judgement**
Allocation concealment?	Unclear	B ‐ unclear

**Table:** Childers 1979

***Study characteristics***		
Methods	Random allocation: method not specified	
Data	Agents of large Midwest‐based multiple‐line insurance company (US)	
Comparisons	1. Advance letter with commitment postcard. Asked to return the postcard to say if will participate and how long they will take to respond. 'Yes' responses then sent a questionnaire. 2. Advance letter with commitment postcard. Asked to return the postcard only to say if will participate. 'Yes' responses then sent questionnaire. 3. Control ‐ no prior commitment sought. All sent questionnaires Reminder postcards sent after 4 days. Non‐respondents sent another questionnaire after 3 weeks	
Outcomes	—	
Topic	Non‐health: insurance	
Mode of Administration	Postal	
Notes	—	
***Risk of bias***		
**Item**	**Authors' judgement**	**Support for judgement**
Allocation concealment?	Unclear	B ‐ unclear

**Table:** Childers 1980a

***Study characteristics***		
Methods	Random allocation: method not specified	
Data	Academics on the American Marketing Association Roster	
Comparisons	1. Egoistic appeal; handwritten postscript 2. Egoistic appeal; typed postscript 3. Help the sponsor appeal; handwritten postscript 4. Help the sponsor appeal; typed postscript 5. Social utility appeal; handwritten postscript 6. Social utility appeal; typed postscript All participants received reminders after 1 week.	
Outcomes	Response period not specified	
Topic	Non‐health: marketing texts	
Mode of Administration	Postal	
Notes	—	
***Risk of bias***		
**Item**	**Authors' judgement**	**Support for judgement**
Allocation concealment?	Unclear	B ‐ unclear

**Table:** Childers 1980b

***Study characteristics***		
Methods	Random allocation: method not specified	
Data	Business practitioners on a mailing list of a major south‐western university (US)	
Comparisons	1. Egoistic appeal; handwritten postscript 2. Egoistic appeal; typed postscript 3. Help the sponsor appeal; handwritten postscript 4. Help the sponsor appeal; typed postscript 5. Social utility appeal; handwritten postscript 6. Social utility appeal; typed postscript All participants received reminders after 1 week.	
Outcomes	Response period not specified	
Topic	Non‐health: marketing texts	
Mode of Administration	Postal	
Notes	—	
***Risk of bias***		
**Item**	**Authors' judgement**	**Support for judgement**
Allocation concealment?	Unclear	B ‐ unclear

**Table:** Childers 1985

***Study characteristics***		
Methods	Random allocation: method not specified	
Data	Policyholders of a national insurance company (US)	
Comparisons	1. Computer‐printed outgoing envelope; computer‐printed return‐envelope; cover letter explained name and address were for research only. 2. Computer‐printed outgoing envelope; computer‐printed return‐envelope; cover letter did not explain name and address were for research only. 3. Computer‐printed outgoing envelope; participants given provision to write own name and address on return‐envelope; cover letter explained name and address were for research only. 4. Computer‐printed outgoing envelope; participants given provision to write own name and address on return‐envelope; cover letter did not explain name and address were for research only. 5. Labelled address on outgoing envelope; computer‐printed return address; cover letter explained name and address were for research only. 6. Labelled address on outgoing envelope; computer‐printed return address; cover letter did not explain name and address were for research only. 7. Labelled address on outgoing envelope; participants given provision to write own name and address on return‐envelope; cover letter explained name and address were for research only. 8. Labelled address on outgoing envelope; participants given provision to write own name and address on return‐envelope; cover letter did not explain name and address were for research only.	
Outcomes	Response within 12 days	
Topic	Non‐health: payment of car insurance	
Mode of Administration	Postal	
Notes	—	
***Risk of bias***		
**Item**	**Authors' judgement**	**Support for judgement**
Allocation concealment?	Unclear	B ‐ unclear

**Table:** Childers TL 1979

***Study characteristics***		
Methods	Random allocation: alternation	
Data	American marketing association practitioner members	
Comparisons	1. Small paper size (8½ X 11") 2. Large paper size (8½ X 14") 3. Single‐sided 4. Double‐sided	
Outcomes	—	
Topic	Non‐health: marketing concepts, employment features	
Mode of Administration	Postal	
Notes	Method of allocation ascertained through contact with author	
***Risk of bias***		
**Item**	**Authors' judgement**	**Support for judgement**
Allocation concealment?	Yes	A ‐ adequate

**Table:** Choi 1990

***Study characteristics***		
Methods	Random allocation: computerised random number generation	
Data	Members of the Ontario Nurses' Association	
Comparisons	1. No stamp on return envelope 2. Business‐reply stamp 3. Metered stamp 4. Small regular stamp 5. Large commemorative stamp	
Outcomes	Response within 92 days	
Topic	—	
Mode of Administration	Postal	
Notes	Method of allocation ascertained through contact with author	
***Risk of bias***		
**Item**	**Authors' judgement**	**Support for judgement**
Allocation concealment?	Yes	A ‐ adequate

**Table:** Choudhury 2012

***Study characteristics***		
Methods	Random allocation: web‐based random number programme	
Data	White British/Irish and Bangladeshi residents in Tower Hamlets, London, UK	
Comparisons	Handwritten envelope vs. word‐processed address in window envelope	
Outcomes	Response period not specified	
Topic	Health (chronic pain)	
Mode of Administration	Postal	
Notes	2‐page questionnaire The Tower Hamlets Pain Study (TOPAS), to compare the population burden of chronic pain in White British/Irish and Bangladeshi residents in Tower Hamlets, the third most economically deprived part of the United Kingdom, where many people do not speak English, and literacy levels are poor (Tower Hamlets Public Health Report, 2007). We translated our postal questionnaires into Bengali and produced phonetic translations into Sylheti for use by the researchers in face‐to‐face interviews and over the telephone.	
***Risk of bias***		
**Item**	**Authors' judgement**	**Support for judgement**
Sequence generation	Yes	Random allocation: web‐based random number programme
Allocation concealment?	Yes	Random allocation: web‐based random number programme
Blinding of participants and personnel	Unclear	Blinding of personnel was not reported, and participants were not blind to intervention.
Blinding of outcome assessment	Yes	No outcome assessment used (counts of responses only)
Incomplete outcome data	Yes	Attrition and exclusions reported for all outcomes
Selective reporting	Unclear	Response period and number of reminders used not reported
Other sources of bias	Yes	No grounds for concern about other sources of bias from reading the study report

**Table:** Christensen 2019

***Study characteristics***		
Methods	Random allocation: method unspecified	
Data	Adults resident in Denmark January 2017	
Comparisons	Motivational sentence in the cover letter intended to heighten perceptions of relevance of the survey vs. no motivational sentence in the cover letter	
Outcomes	Response period not specified	
Topic	Health (Danish Health and Morbidity Survey)	
Mode of Administration	Postal	
Notes	Questionnaire included a minimum of 52 questions. Included questions on, for example, sociodemographic factors, quality of life, long‐standing illness, health behaviour, contact with health services and social relations, the 12‐Item Short‐Form Health Survey v2, the Perceived Stress Scale and a screening tool for alcohol abuse (the CAGE‐C test. Two reminders were sent via the regular postal service. In the introductory letter and the second reminder, a paper questionnaire and a prepaid return envelope were enclosed.	
***Risk of bias***		
**Item**	**Authors' judgement**	**Support for judgement**
Sequence generation	Unclear	Random allocation: method unspecified
Allocation concealment?	Unclear	Random allocation: method unspecified
Blinding of participants and personnel	Unclear	Blinding of personnel not described. Participants were not blinded to intervention.
Blinding of outcome assessment	Yes	No outcome assessment used (counts of responses only)
Incomplete outcome data	No	Exclusions were not reported.
Selective reporting	Yes	The overall response rate was reported for both arms of the trial. No other response times were measured.
Other sources of bias	Yes	No grounds for concern about other sources of bias from reading the study report

**Table:** Christie 1985

***Study characteristics***		
Methods	Random allocation: alternation	
Data	People aged 18+ years listed in the 1984 Auckland telephone directory, New Zealand	
Comparisons	1. Handwritten signature on covering letter (HW); actual age and income asked for (AAI); typed address on outgoing envelope (Ty) 2. HW; AAI; handwritten address on outgoing envelope (HE) 3. HW; age and income bracket asked for (AIB); Ty 4. HW; AIB; HE 5. Typed signature on covering letter (T); AAI; Ty 6. T; AAI; HE 7. T; AIB; Ty 8. T; AIB; HE	
Outcomes	Response period not specified	
Topic	Non‐health: marketing ‐ awareness of macadamia nuts, purchase behaviour	
Mode of Administration	Postal	
Notes	—	
***Risk of bias***		
**Item**	**Authors' judgement**	**Support for judgement**
Allocation concealment?	No	C ‐ inadequate

**Table:** Church 2004

***Study characteristics***		
Methods	Random allocation: method not specified	
Data	Residents of Wright County in Minnesota, US	
Comparisons	1. Only questionnaire 2. Questionnaire + Faecal Occult Blood Test (FOBT) ‐ no reminder 3. Questionnaire + Faecal Occult Blood Test (FOBT) + reminder	
Outcomes	Response period not specified	
Topic	Health: colorectal screening	
Mode of Administration	Postal	
Notes	Mean age: 63 years; mainly females; 49% of participants belonging to group 2 were inadvertently delivered the 1st reminder.

**Table:** Clark 2001

***Study characteristics***		
Methods	Random allocation: computerised random number generation	
Data	All consultants listed on the Royal College of Obstetricians and Gynaecologists database (UK)	
Comparisons	1. Simple plastic ballpoint pen sent with questionnaire 2. No pen	
Outcomes	Response period not specified	
Topic	Health: views on gynaecological endoscopy	
Mode of Administration	Postal	
Notes	1 reminder was sent to all non‐responders 3 months after initial mailing	
***Risk of bias***		
**Item**	**Authors' judgement**	**Support for judgement**
Allocation concealment?	Yes	A ‐ adequate

**Table:** Clark 2011

***Study characteristics***		
Methods	Random allocation: method not specified	
Data	Director of Nursing and the Administrator in 205 U.S. nursing homes (at least 30 beds, across the 48 contiguous states (excluding Alaska, Hawaii, and Washington, DC)	
Comparisons	Mailed vs. Web Short vs. long $35 vs. $50 incentive	
Outcomes	Response after 8 weeks (at least one respondent, i.e. Director of Nursing or the Administrator)	
Topic		
Mode of Administration	Postal or electronic (web)	
Notes	Questionnaire not described. Web option participants were mailed a cover letter with URL; mailed option mailed a cover letter with paper questionnaire and SAE; short questionnaire 5‐10 min questionnaire, vs long 20‐40 mins We were specifically interested in comparing mail versus Internet data collection amongst nursing home providers because of previous studies suggesting particularly little information technology in nursing homes and the concern that nursing home providers may lack Internet access during normal business hours. Each selected facility was contacted by telephone to obtain the names and contact information for the Director of Nursing and the Administrator.	
***Risk of bias***		
**Item**	**Authors' judgement**	**Support for judgement**
Sequence generation	Unclear	Random allocation: method unspecified
Allocation concealment?	Unclear	Random allocation: method unspecified
Blinding of participants and personnel	Unclear	Blinding of personnel not described. Participants were not blinded to intervention.
Blinding of outcome assessment	Yes	No outcomes were assessed (counts of responses only)
Incomplete outcome data	Yes	Exclusions discussed
Selective reporting	Yes	Response after 8 weeks reported
Other sources of bias	Yes	No grounds for concern about other sources of bias from reading the study report

**Table:** Clark 2015

***Study characteristics***		
Methods	Random allocation: method unspecified	
Data	Current smokers aged 35 or more at participating general practices across the Yorkshire and Humberside area, in the UK	
Comparisons	Electronic prompt (2 days after the questionnaire was sent) via SMS or email vs. control	
Outcomes	Response after 8 weeks	
Topic	Health (chronic obstructive pulmonary disease)	
Mode of Administration	Postal (with or without electronic reminders)	
Notes	Questionnaire design not described. Two reminder letters were sent in an attempt to encourage response. The first reminder letter was sent 2 weeks after the follow‐up questionnaire, and the second reminder was sent 2 weeks later (i.e. 4 weeks after the follow‐up questionnaire). Study participants received an additional electronic prompt (email and/or text messages) to return their questionnaire or to receive no additional prompt. This was in addition to the two reminder letters that all DOC participants received. Participants were those who supplied mobile phone numbers and/or email addresses.	
***Risk of bias***		
**Item**	**Authors' judgement**	**Support for judgement**
Sequence generation	Unclear	Random allocation: method unspecified
Allocation concealment?	Unclear	Random allocation: method unspecified
Blinding of participants and personnel	Unclear	Blinding of personnel not described. Participants were not blinded to intervention.
Blinding of outcome assessment	Yes	No outcome assessment used (counts of responses only)
Incomplete outcome data	No	Exclusions not reported
Selective reporting	Yes	Response after 8 weeks reported
Other sources of bias	Yes	No grounds for concern about other sources of bias from reading the study report

**Table:** Clark TJ 2001

***Study characteristics***		
Methods	Random allocation: computerised random number generation	
Data	All gynaecologists identified from the British Society of Gynaecological Endoscopy database of members	
Comparisons	1. Questionnaire and covering letter printed on standard quality white paper 2. Questionnaire and covering letter printed on high‐quality white paper	
Outcomes	Response period not specified	
Topic	Health: hysteroscopy	
Mode of Administration	Postal	
Notes	—	
***Risk of bias***		
**Item**	**Authors' judgement**	**Support for judgement**
Allocation concealment?	Yes	A ‐ adequate

**Table:** Clarke 1998

***Study characteristics***		
Methods	Random allocation: computerised random number generation	
Data	Study survivors resident in 3 health authority areas	
Comparisons	1. 3 extra questions on current sources of income included 2. Extra questions not included 3. Extra questionnaire on cognitive functioning included 4. Extra questionnaire not included	
Outcomes	Response period not specified	
Topic	Health: Whitehall study	
Mode of Administration	Postal	
Notes	Method of allocation ascertained through contact with author; mean age: 77 years; mainly males	
***Risk of bias***		
**Item**	**Authors' judgement**	**Support for judgement**
Allocation concealment?	Yes	A ‐ adequate

**Table:** Clarke 2007

***Study characteristics***		
Methods	A randomisation list was prepared by using a series of coin flips	
Data	Presidents and Deans of medical royal colleges and Deans of postgraduate medical and dental schools in the UK	
Comparisons	Sender's knighthood explicit on letter vs not	
Outcomes	Response after one reminder letter sent after 4 weeks	
Topic	Non‐health (institution's attitudes to critical appraisal and systematic reviews)	
Mode of Administration	Postal	
Notes	The letters were one and a half pages long, so that the signature and the 'Sir Iain Chalmers' or 'Iain Chalmers' appeared about halfway down the second page. IC signed each letter with his usual signature. The front page of the letter was on UK Cochrane Centre‐headed note paper, which did not contain IC's name.	
***Risk of bias***		
**Item**	**Authors' judgement**	**Support for judgement**
Sequence generation	Yes	The series of coin flips to determine a randomisation group was written down with no knowledge of the intended recipients of the letters and in advance of the preparation of an alphabetic list of these people.
Allocation concealment?	Yes	The series of coin flips to determine a randomisation group was written down with no knowledge of the intended recipients of the letters and in advance of the preparation of an alphabetic list of these people.
Blinding of participants and personnel	Yes	The randomisation list was prepared by a personal assistant and then passed to MC who applied the random sequence to the alphabetic list. LH then prepared the letters in accordance with the allocation.
Blinding of outcome assessment	Yes	No outcome assessment used (counts of responses only)
Incomplete outcome data	No	No exclusions reported
Selective reporting	Yes	Response after one reminder letter sent after 4 weeks was reported.
Other sources of bias	Yes	No grounds for concern about other sources of bias from reading the study report

**Table:** Clausen 1947

***Study characteristics***		
Methods	Random allocation: systematic division	
Data	Non‐respondents to an earlier survey	
Comparisons	1. Impersonal salutation; handwritten signature; franked outward envelope 2. Impersonal salutation; facsimile signature; franked outward envelope 3. Personal salutation; facsimile signature; franked outward envelope 4. Personal salutation; handwritten signature; franked outward envelope 5. Personal salutation; handwritten signature; airmail and special delivery outward envelope	
Outcomes	Response within 4 weeks	
Topic	Health: National Service Life Insurance (NSLI)	
Mode of Administration	Postal	
Notes	—	
***Risk of bias***		
**Item**	**Authors' judgement**	**Support for judgement**
Allocation concealment?	No	C ‐ inadequate

**Table:** Claycomb 2000

***Study characteristics***		
Methods	Random allocation: method not specified	
Data	Marketing executives and managers representing a geographic cross‐section of the US	
Comparisons	Intervals between the original and 2 rounds of follow‐up mailings: 1. 3 days 2. 6 days 3. 9 days 4. 12 days 5. 15 days 6. 18 days 7. 21 days 8. 24 days 9. 27 days 10. 30 days 11. 33 days 12. 36 days 13. 39 days 14. 42 days 15. 45 days 16. 48 days 17. 51 days 18. 54 days 19. 57 days 20. 60 days	
Outcomes	Response after 3 mailings	
Topic	Non‐health: companies' customer relation practices	
Mode of Administration	Postal	
Notes	Dates of initial mailings randomised to prevent seasonal biases	
***Risk of bias***		
**Item**	**Authors' judgement**	**Support for judgement**
Allocation concealment?	Unclear	B ‐ unclear

**Table:** Cleopas 2006

***Study characteristics***		
Methods	Random allocation: computerised random number generation	
Data	Adults discharged from teaching hospital system in Geneva	
Comparisons	1. 2‐original response format (yes/no) 2. 3‐point similarity format (applies completely/in part/not at all) 3. 5‐point intensity format (completely true to completely false) 4. 5‐point frequency format (all the time to never)	
Outcomes	Response period not specified	
Topic	Health: patient‐based outcome measure (Nottingham Health Profile)	
Mode of Administration	Postal	
Notes	Method of allocation ascertained through contact with author; mainly females	
***Risk of bias***		
**Item**	**Authors' judgement**	**Support for judgement**
Allocation concealment?	Yes	A ‐ adequate

**Table:** Coast 2006

***Study characteristics***		
Methods	Random allocation: methods unspecified	
Data	Patients eligible for care by a GP with a special interest in dermatology	
Comparisons	Shorter questionnaire (11 pages) vs. longer (15 pages)	
Outcomes	Response after 2 reminders	
Topic	Health (access to dermatology secondary care services)	
Mode of Administration	Postal	
Notes	Shorter questionnaire (11 pages); longer questionnaire (15 pages) Patients were sent a letter asking for their consent to participate in the research. On receipt of the signed consent forms, a study number was allocated to each respondent and a pre‐numbered envelope containing one of the two versions of the questionnaire was sent. Questionnaires were randomly selected. Up to 2 reminders were sent to non‐respondents.	
***Risk of bias***		
**Item**	**Authors' judgement**	**Support for judgement**
Sequence generation	Unclear	Random allocation: methods unspecified
Allocation concealment?	Unclear	Random allocation: methods unspecified
Blinding of participants and personnel	Yes	Randomised and placed in envelope by a member of research team not involved in subsequent allocation of study numbers and posting of questionnaire
Blinding of outcome assessment	Yes	No outcomes were assessed (counts of responses only)
Incomplete outcome data	Yes	Exclusions reported in full
Selective reporting	Yes	Response after 3 reminders reported in full
Other sources of bias	Yes	No grounds for concern about other sources of bias from reading the study report

**Table:** Cobanoglu 2001

***Study characteristics***		
Methods	Random allocation: method unspecified	
Data	300 hospitality professors randomly chosen from the Council on hotel, restaurant, and institutional education members with email addresses, US	
Comparisons	Postal vs. fax vs. web	
Outcomes	Surveys returned	
Topic	Non‐health (hospitality education)	
Mode of Administration	Postal, web and fax	
Notes	Questionnaire not described. A cover letter introducing the survey was personally addressed, with specific instructions on how to respond. For the mail group, personalised cover letters were printed on university letterhead using a mail merge program and folded with a printed survey and business reply envelope. The faxed version included a personalised cover letter, with a university letterhead logo embedded into the software, and a survey. For the web‐based survey, an email message was sent to the professors along with a cover letter and the website address. The respondents were informed that they could request a paper copy of the survey should they have problems accessing the survey online. A unique website address was created for each respondent with the help of a common gateway interface protocol.	
***Risk of bias***		
**Item**	**Authors' judgement**	**Support for judgement**
Sequence generation	Unclear	Random allocation: method unspecified
Allocation concealment?	Unclear	Random allocation: method unspecified
Blinding of participants and personnel	Unclear	Blinding of personnel not specified. Participants were not blind to intervention.
Blinding of outcome assessment	Yes	No outcome assessment used (counts of responses only)
Incomplete outcome data	Yes	Of the original 300 surveys, only six surveys came back as undelivered. One mail survey was returned as having gone to the wrong address; all the fax surveys went through successfully; and five of the email invitations were returned as undeliverable.
Selective reporting	Yes	One outcome: surveys returned fully reported
Other sources of bias	Yes	No grounds for concern about other sources of bias from reading the study report

**Table:** Cobanoglu 2003

***Study characteristics***		
Methods	Random allocation: computerised random number generation	
Data	Managers who are members of the American Management Association (AMA)	
Comparisons	1. Luggage tag (LT) 2. Prize draw for a personal digital assistant (PDA) 3. Prize draw for both LT and PDA 4. Control	
Outcomes	Response period not specified	
Topic	Not specified	
Mode of Administration	Electronic: online survey	
Notes	Method of allocation ascertained through contact with author	
***Risk of bias***		
**Item**	**Authors' judgement**	**Support for judgement**
Allocation concealment?	Yes	adequate

**Table:** Cochrane 2020

***Study characteristics***		
Methods	Computer‐generated using Stata version 13.0	
Data	OTIS study participants (aged 65 years or over, willing to receive a home visit from an OT, community dwelling, at risk of a fall in the next 12 months), UK	
Comparisons	Personalised SMS reminder vs standard SMS	
Outcomes	Proportion of questionnaires returned to York Trials Unit at four months post‐randomisation after one reminder letter	
Topic	Health (prevention of falls in older people)	
Mode of Administration	Postal with electronic prompts	
Notes	Questionnaire not described	
***Risk of bias***		
**Item**	**Authors' judgement**	**Support for judgement**
Sequence generation	Yes	Random allocation: computer‐generated using Stata version 13.0
Allocation concealment?	Yes	Randomc allocation: computer‐generated using Stata version 13.0
Blinding of participants and personnel	Yes	Blinding of personnel not described. Participants were not aware of their involvement within this SWAT; only to the OTIS trial group allocation. Study team members performing administrative, statistical or health economic roles were not blinded, but data entry staff were.
Blinding of outcome assessment	Yes	No outcome assessment used (counts of responses only)
Incomplete outcome data	Yes	Delays in setting‐up the text messaging system meant no texts were sent prior to 7th December 2017. In total, 120 (29.8%) randomised participants were due texts before this date. These participants are therefore excluded from the analysis.
Selective reporting	Yes	Proportion of questionnaires returned to York Trials Unit at four months post‐randomisation after one reminder letter was reported for both arms of the trial.
Other sources of bias	Yes	No grounds for concern about other sources of bias from reading the study report

**Table:** Cockayne 2005

***Study characteristics***		
Methods	Random allocation	
Data	Community‐dwelling women aged over 70 years living in the York and Cumbria area	
Comparisons	1. Offer of study results 2. Control	
Outcomes	Response period not specified	
Topic	Health: calcium and vitamin D supplementation for fracture prevention	
Mode of Administration	Postal	
Notes	Independent researchers from the York Trials Unit randomised the eligible women. Administration of the questionnaire was not blind to group allocation. Age: Above 70 years; mainly females	
***Risk of bias***		
**Item**	**Authors' judgement**	**Support for judgement**
Allocation concealment?	Yes	adequate

**Table:** Cohen 2019

***Study characteristics***		
Methods	Random allocation: computer‐generated (random number generator in Stata)	
Data	Authors and editors involved in online publishing totalling 2426 participants from 111 countries	
Comparisons	$100 gift card incentive vs. $2.50 altruistic donation to rotary club vs. no incentive	
Outcomes	Response after 1 reminder sent to non‐responders 10 days after the initial survey was sent	
Topic	Non‐health (author and editor attitudes regarding predatory publishing)	
Mode of Administration	Electronic	
Notes	Questionnaire not described. An automated survey invitation was sent to each email stating the incentive, as such participants were not blind. However, subjects were unaware there were different incentives for other invitees. Each email invitation was personalised with the individual's name in an automated fashion to increase the likelihood of individuals reading the email and completing the survey. 1 automated reminder sent to non‐responders 10 days after the initial survey was sent.	
***Risk of bias***		
**Item**	**Authors' judgement**	**Support for judgement**
Sequence generation	Yes	Random allocation: subject randomised using a random number generator in Stata
Allocation concealment?	Yes	Random allocation: subject randomised using a random number generator in Stata
Blinding of participants and personnel	Yes	An automated survey invitation was sent to each email stating the incentive, as such participants were not blind. However, subjects were unaware there were different incentives for other invitees. Authors were blinded to group assignments while surveys were administered. Data analysis took place in a blinded fashion based on three groups of unknown incentives.
Blinding of outcome assessment	Yes	No outcome assessment used (counts of responses only)
Incomplete outcome data	Yes	199 (8%) of email contact information resulted in a return to sender response, leaving a final 2227 participants.
Selective reporting	Yes	Response after 1 reminder reported
Other sources of bias	Yes	No grounds for concern about other sources of bias from reading the study report

**Table:** Collins 2000

***Study characteristics***		
Methods	Random allocation: method not specified	
Data	Members of the RAND adolescent/young adult panel study drawn from schools across the US	
Comparisons	1. $20 cash with mailing 2. $20 cash promised on return of questionnaire 3. $25 cash promised on return of questionnaire	
Outcomes	Response within approximately 4 months	
Topic	Health: substance use, problem behaviour, predictors of risk behaviour, attitudes and beliefs	
Mode of Administration	Postal	
Notes	Mainly females	
***Risk of bias***		
**Item**	**Authors' judgement**	**Support for judgement**
Allocation concealment?	Unclear	B ‐ unclear

**Table:** Conner 2017

***Study characteristics***		
Methods	Random allocation: using a random number generator	
Data	13,803 participants aged 65+ recruited from 7 GPs in Northern England not participating in flu vaccine invitation scheme	
Comparisons	Post‐it note attached to the questionnaire with blue (imitation handwritten) message ('Please take a few minutes to complete this for us. Thank you!') vs. no post‐it note	
Outcomes	Response after reminders	
Topic	Health (influenza vaccination attitudes and intentions)	
Mode of Administration	Postal	
Notes	Letter and SAE with questionnaire and option to enter a £200 prize draw. Questionnaires included a code number to allow them to be matched to patient records. Materials were sent one month before the influenza vaccinations were available.	
***Risk of bias***		
**Item**	**Authors' judgement**	**Support for judgement**
Sequence generation	Yes	Random allocation: using a random number generator
Allocation concealment?	Yes	Random allocation: using a random number generator
Blinding of participants and personnel	Unclear	Blinding of personnel not described. Participants were not blinded to intervention.
Blinding of outcome assessment	Yes	No outcomes were assessed (counts of responses only)
Incomplete outcome data	Yes	Exclusions reported
Selective reporting	Yes	Response after reminders reported in full
Other sources of bias	Yes	No grounds for concern about other sources of bias from reading the study report

**Table:** Converse 2008

***Study characteristics***		
Methods	Random allocation: method unspecified	
Data	1500 pre‐K through Grade 12 teachers awarded certification by the National Board for Professional Teaching Standards (NBPTS) in the states of Ohio and South Carolina (750 teachers from each state), US	
Comparisons	(a) Mail then email/Web follow‐up vs. (b) email/Web then mail follow‐up	
Outcomes	Postal‐postal pre‐notification (with unconditional $2), followed by questionnaire and SAE. Postal reminder, then a 2nd postal questionnaire and email with URL. Electronic‐postal pre‐notification (with unconditional $2), followed by email and questionnaire URL, email reminder followed by 2nd email reminder and postal questionnaire with SAE	
Topic	Non Health (voluntary evaluation for recognising accomplished educators)	
Mode of Administration	Postal and electronic	
Notes	4 sections; 53 core items, 42 additional (branching); mixed format (Likert scale, yes/no, commentary, tick applicable); paper‐based version and web‐based to preserve appearance and content of paper The mail‐email/Web group received the following contacts: (a) a pre‐notice letter, addressed to the participant and sent via US mail, explaining that a survey would be arriving soon, along with a $2 bill as a token of appreciation; (b) a questionnaire, letter of instruction, and a pre‐addressed stamped envelope for return via US mail; (c) a stamped postcard reminder via US mail; (d) a second questionnaire in an envelope and with a letter similar to the initial questionnaire mailing; and (e) a special contact via email that directed the participant to the Web‐based questionnaire. The email/Web‚ mail group received the following contacts: (a) a pre‐notice letter, addressed to the participant and sent via US mail, explaining that a survey would be arriving soon, along with a $2 bill as a token of appreciation; (b) an email that directed the participant to the Web‐based questionnaire; (c) an email reminder; (d) a second email reminder; and (e) a special contact consisting of a US mail questionnaire that was identical to that sent as the initial mail‐mail/Web group questionnaire.	
***Risk of bias***		
**Item**	**Authors' judgement**	**Support for judgement**
Sequence generation	Unclear	Random allocation: method unspecified
Allocation concealment?	Unclear	Random allocation: method unspecified
Blinding of participants and personnel	Unclear	Blinding of personnel not described. Participants were not blinded to intervention.
Blinding of outcome assessment	Yes	No outcome assessment used (counts of responses only)
Incomplete outcome data	Yes	Six individuals from the original sample were removed for these analyses: 4 individuals were not National Board Certified Teachers (NBCTs), 1 indicated he or she did not wish to participate, and 1 entered a duplicate ID number when responding by Web. The analyses were based on the remaining 1494 individuals.
Selective reporting	Yes	Responses after all contacts are reported
Other sources of bias	Yes	No grounds for concern about other sources of bias from reading the study report

**Table:** Cook 2016

***Study characteristics***		
Methods	Computer‐generated (randomisation for both interventions was done by a research assistant using a random number generator in Microsoft Excel)	
Data	Licenced physicians in the United States	
Comparisons	Conditional non‐monetary incentive (book) vs no incentive; letter & email reminder vs postcard & email reminder vs email only reminder	
Outcomes	Response after 6 reminders	
Topic	Health (physician opinions regarding maintenance of certification and continuing medical education)	
Mode of Administration	Electronic	
Notes	Questionnaire not described (other than asking for opinions regarding maintenance of certification and continuing medical education) 7 days after the first email, we sent a reminder via paper mail to a subset of invitees using two formats. We timed this mailing to arrive at approximately the same time as the first email reminder. The invitees in one group received a personalised letter, printed on institution letterhead bonded paper and sealed in an envelope, asking them to complete the survey using the link they had received via email, or to contact the study investigators if they had not received or had deleted the email. A second group received a similar message via a personalised postcard that included the institution logo on both sides and was signed by one of the investigators. A third group received no paper reminder.	
***Risk of bias***		
**Item**	**Authors' judgement**	**Support for judgement**
Sequence generation	Yes	Random allocation: randomisation using a random number generator (Microsoft Excel)
Allocation concealment?	Yes	Random allocation: randomisation using a random number generator (Microsoft Excel)
Blinding of participants and personnel	Unclear	Blinding of personnel not described. Participants were not blinded to intervention.
Blinding of outcome assessment	Yes	No outcome assessment used (counts of responses only)
Incomplete outcome data	Yes	We received notification that 682 emails were undeliverable, leaving 3966 potential respondents.
Selective reporting	Yes	Response after reminders was reported.
Other sources of bias	Yes	No grounds for concern about other sources of bias from reading the study report

**Table:** Corcoran 1985

***Study characteristics***		
Methods	Random allocation: method not specified	
Data	Masters level social workers	
Comparisons	1. First‐class stamped return envelope 2. Reply permit return envelope Follow‐up postcard sent to all subjects 3 to 4 weeks after original mailing	
Outcomes	Response period not specified	
Topic	—	
Mode of Administration	Postal	
Notes	—	
***Risk of bias***		
**Item**	**Authors' judgement**	**Support for judgement**
Allocation concealment?	Unclear	B ‐ unclear

**Table:** Coryn 2020

***Study characteristics***		
Methods	Random allocation: method unspecified	
Data	American Evaluation Association (AEA) Members	
Comparisons	No incentive vs. lottery (for $800 tablet) vs. non‐monetary incentive ($5 voucher) vs. philanthropic donation ($5 to the AEA Graduate Education Diversity Internship programme)	
Outcomes	Response after 4 reminders	
Topic	Non‐health (research on evaluation)	
Mode of Administration	Electronic (Internet surveys)	
Notes	7 survey items; 4 closed‐response (one with matrix of 18 factors to be rated as to the extent to which they positively or negatively influence whether to complete a survey questionnaire). A pre‐survey notification email sent 1 week prior to survey. Weekly reminders (four in all) were sent to non‐responders. All communications sent to four groups were identical, except for the following: in lottery group: "As a token of our gratitude for participating in this study, you will be entered in a lottery to have an opportunity to receive a tablet (e.g. Apple iPad, Microsoft Surface) of your choice valued at up to US$800"; in token group: "As a token of our gratitude for participating in this study, you will receive a US$5 Amazon.com gift card"; philanthropic donation group: "As a token of our gratitude for participating in this study, we will donate US$5 to the AEA Graduate Education Diversity Internship (GEDI) program on your behalf"; control group: "This study can only be successful with the generous help of our fellow evaluators" (also communicated in the messages sent to the other three conditions). The fact that alternative incentives existed or were offered was intentionally withheld. The AEA member database used was de‐identified, thus preventing the use of potential respondents‚ names or titles in communications.	
***Risk of bias***		
**Item**	**Authors' judgement**	**Support for judgement**
Sequence generation	Unclear	Random allocation: method unspecified
Allocation concealment?	Unclear	Random allocation: method unspecified
Blinding of participants and personnel	Yes	Blinding of personnel not described. Participants were blinded to intervention, the fact that alternative incentives existed or were offered was intentionally withheld.
Blinding of outcome assessment	Yes	No outcomes were assessed (counts of responses only)
Incomplete outcome data	No	Exclusions not reported
Selective reporting	Yes	Response after 4 reminders reported in full
Other sources of bias	Yes	No grounds for concern about other sources of bias from reading the study report

**Table:** Cosgrove 2018

***Study characteristics***		
Methods	Random allocation: method unspecified	
Data	Professional social workers and volunteer community mediators	
Comparisons	Unconditional monetary ($2) incentive vs no incentive	
Outcomes	Response after 6 weeks (42 days)	
Topic	Health (factors associated with burnout and intention to leave amongst professional social workers and volunteer community mediators)	
Mode of Administration	Postal and electronic	
Notes	116 item survey; serif font for paper and sans serif for web; fewer page items for web; cover page topical photo for both; 20 mins to complete The paper survey was mailed to all participants, along with a cover letter signed by all members of the research team explaining that participation was voluntary and anonymous, and an addressed and stamped envelope to return the completed survey. The incentive was included with the initial mailing of the survey, and was not contingent upon response. Fourteen days after the initial mailing, a follow‐up postcard was sent to all participants that thanked them for completing the survey, encouraged those who had not completed the survey to do so, and included a web address to complete the survey online. The surveys were printed on different colours of paper to distinguish the responses of participants who did and did not receive the incentive.	
***Risk of bias***		
**Item**	**Authors' judgement**	**Support for judgement**
Sequence generation	Unclear	Random allocation: method unspecified
Allocation concealment?	Unclear	Random allocation: method unspecified
Blinding of participants and personnel	Unclear	Blinding of personnel not reported. Participants were not blind to intervention.
Blinding of outcome assessment	Yes	No outcome assessment used (counts of responses only)
Incomplete outcome data	Yes	Survey mailings were returned‐to‐sender for 20 VCMs and 10 LBSWs.
Selective reporting	Yes	Response after 6 weeks reported for both arms of the trial
Other sources of bias	Yes	No grounds for concern about other sources of bias from reading the study report

**Table:** Cotterill 2017

***Study characteristics***		
Methods	Computer‐generated	
Data	Older people with long‐term conditions and social‐care needs participating in the Comprehensive Longitudinal Assessment of Salford Integrated Care (CLASSIC) cohort study	
Comparisons	Social pressure letter (letting the recipient know that their previous response had been noted and that future responses would be noted by the researcher) vs. letter without the social pressure text	
Outcomes	The primary outcome was retention in the host study, defined as return of both surveys, sent at 6 and 12 months after baseline. At each stage, non‐responders sent reminder letter after 3 weeks	
Topic	Health (brief measures of service experience, health and care outcomes and service utilisation)	
Mode of Administration	Postal	
Notes	Questionnaire not described. Participants who did not return a questionnaire were sent a second copy with a reminder letter three weeks later. Participants in both groups were offered an incentive of a £10 voucher for completion of the first (baseline) questionnaire and £5 for completion of the third questionnaire, but no incentive was given for completion of the second questionnaire.	
***Risk of bias***		
**Item**	**Authors' judgement**	**Support for judgement**
Sequence generation	Yes	Random allocation: computer‐generated
Allocation concealment?	Yes	Random allocation: computer‐generated
Blinding of participants and personnel	Yes	Participants were blinded to their participation in the embedded study. They were not informed that other households were being sent differently worded letters: we expected that their actions might change if they knew what others received. The research team were not blinded to the intervention, but had minimal contact with trial participants.
Blinding of outcome assessment	Yes	No outcome assessment used (counts of responses only)
Incomplete outcome data	Yes	Exclusions reported
Selective reporting	Yes	Response to both questionnaires reported in full
Other sources of bias	Yes	No grounds for concern about other sources of bias from reading the study report

**Table:** Cottrell 2015

***Study characteristics***		
Methods	Random allocation: method unspecified	
Data	Fully qualified GPs who had managed a patient with chronic knee pain in the last six months	
Comparisons	Incentive (entry into a prize draw to win a £100 voucher vs. none) Length (eight sides of A4) vs. four sides of A4	
Outcomes	Response period not specified	
Topic	Non‐health (attitudes, beliefs and reported clinical management of GPs regarding exercise for chronic knee pain)	
Mode of Administration	Postal	
Notes	The standard questionnaire was eight sides of A4 in length; contained 85 items. The questionnaire was presented in an A4 booklet format, created from folded and stapled A3 pages; the abbreviated questionnaire contained 36 items. The questionnaires were printed as booklets on white paper with the institution logo on the front cover. Initial mailing included a personalised combined cover letter and information sheet along with a questionnaire and SAE. Non‐responders sent reminder postcard after 2 weeks on A5 yellow card. Non‐responders further mailed a personalised reminder letter with 2nd questionnaire and SAE after a further 2 weeks (4 weeks)	
***Risk of bias***		
**Item**	**Authors' judgement**	**Support for judgement**
Sequence generation	Unclear	Random allocation: method unspecified
Allocation concealment?	Unclear	Random allocation: method unspecified
Blinding of participants and personnel	Unclear	Blinding of personnel not reported. Participants were not blinded.
Blinding of outcome assessment	Yes	No outcome asessment used (counts of responses only)
Incomplete outcome data	Yes	19 GPs did not meet inclusion criteria.
Selective reporting	Yes	Responses reported in full
Other sources of bias	Yes	No grounds for concern about other sources of bias from reading the study report

**Table:** Coughlin 2011

***Study characteristics***		
Methods	Random allocation: method unspecified	
Data	Recent veterans who had been deployed in support of Operation Enduring Freedom/Operation Iraqi Freedom, US	
Comparisons	Unconditional $5 cheque incentive vs. conditional $5 cheque incentive vs. no incentive	
Outcomes	Response period not specified	
Topic	Topic not specified	
Mode of Administration	Postal and web	
Notes	16‐page questionnaire. Packet contained 16‐page paper questionnaire, introductory letter signed by senior VA official, consent form and SAE. Also included URL. 2nd questionnaire sent 2 weeks later, and final questionnaire a further 4 weeks later. Reminder/thank you postcards sent one week after each mailing	
***Risk of bias***		
**Item**	**Authors' judgement**	**Support for judgement**
Sequence generation	Unclear	Random allocation: method unspecified
Allocation concealment?	Unclear	Random allocation: method unspecified
Blinding of participants and personnel	Unclear	Blinding of personnel not specified. Participants were not blind to intervention.
Blinding of outcome assessment	Yes	No outcome assessment used (counts of responses only)
Incomplete outcome data	Yes	Received 137 refusals and learned of 2 deaths, bringing the number of contacted veterans to 783.
Selective reporting	Yes	Final response reported
Other sources of bias	Yes	No grounds for concern about other sources of bias from reading the study report

**Table:** Cox 1974

***Study characteristics***		
Methods	Random allocation: method not specified	
Data	Residents of a southwestern city listed in the metropolitan telephone directory (US)	
Comparisons	1. Personalised cover letter; follow‐up postcard after 3 days 2. Personalised cover letter; no follow‐up postcard 3. No personalised cover letter; follow‐up postcard after 3 days 4. No personalised cover letter; no follow‐up postcard	
Outcomes	Response within 16 days	
Topic	Non‐health: finance ‐ appraise consumer evaluations of financial institutions	
Mode of Administration	Postal	
Notes	—	
***Risk of bias***		
**Item**	**Authors' judgement**	**Support for judgement**
Allocation concealment?	Unclear	B ‐ unclear

**Table:** Crittenden 1985

***Study characteristics***		
Methods	Random allocation: method not specified	
Data	Alumni members of a National Business School	
Comparisons	1. White questionnaire 2. Yellow questionnaire 3. Questionnaire using letter‐quality printer 4. Questionnaire using Dot‐matrix printer	
Outcomes	Response period not specified	
Topic	Non‐health: education	
Mode of Administration	Postal	
Notes	2 x 2 factorial design	
***Risk of bias***		
**Item**	**Authors' judgement**	**Support for judgement**
Allocation concealment?	Unclear	B ‐ unclear

**Table:** Cunningham‐Burley 2020

***Study characteristics***		
Methods	Random allocation: method unspecified	
Data	Healthcare workers participating in a research study about slip‐resistant footwear in the NHS workplace	
Comparisons	Pen vs. no pen	
Outcomes	Response proportion after 4 months	
Topic	Health (slips in the workplace)	
Mode of Administration	Postal	
Notes	10‐page questionnaire It may be that, in this group of participants, the pen failed to act as a facilitator or was not a sufficient incentive to return the questionnaire, given the fact that participants in the trial already received a free pair of shoes (although offer of shoes was not conditional on returning the questionnaire).	
***Risk of bias***		
**Item**	**Authors' judgement**	**Support for judgement**
Sequence generation	Unclear	Random allocation: method unspecified
Allocation concealment?	Unclear	Random allocation: method unspecified
Blinding of participants and personnel	Yes	Participants were not aware of their involvement in this SWAT but due to the nature of the intervention participants and study team members could not be blinded to group allocation.
Blinding of outcome assessment	Yes	No outcomes were assessed (counts of responses only)
Incomplete outcome data	Yes	In total, 13 participants withdrew from the main SSHeW trial after they had been randomised into the SWAT but before being sent their follow‐up questionnaire.
Selective reporting	Yes	All response outcomes reported
Other sources of bias	Yes	No grounds for concern about other sources of bias from reading the study report

**Table:** Cureton 2021

***Study characteristics***		
Methods	Computer‐generated randomisation list	
Data	Adults with rotator cuff disorder, UK	
Comparisons	Personalised vs standard text message reminder	
Outcomes	Response at 125 days	
Topic	Health (shoulder pain and function over 12 months after randomisation in the GRASP trial measured using the Shoulder Pain and Disability Index)	
Mode of Administration	Postal (electronic reminder option for non‐responders)	
Notes	Questionnaire not described The text message was sent to trial participants at the same time as their 6‐month follow‐up postal questionnaire was sent by the trial team; therefore, it would arrive a few days before the participant received their follow‐up questionnaire. Participants needed to have the use of a mobile telephone. Participants were willing to provide a mobile telephone number and consented for contact to be made by the trial team using this number.	
***Risk of bias***		
**Item**	**Authors' judgement**	**Support for judgement**
Sequence generation	Yes	Random allocation: computer‐generated
Allocation concealment?	Yes	Random allocation: computer‐generated
Blinding of participants and personnel	Unclear	Blinding of personnel not specified. Participants were not blinded to intervention.
Blinding of outcome assessment	Yes	No outcome assessment used (counts of responses only)
Incomplete outcome data	Yes	Six participants were not sent the 6‐month follow‐up questionnaire; of these five withdrew from the host trial prior to the follow‐up time point, and one was missed from the mailout and SMS list in error.
Selective reporting	Yes	Response at 125 days reported for both arms
Other sources of bias	Yes	No grounds for concern about other sources of bias from reading the study report

**Table:** Cycyota 2002

***Study characteristics***		
Methods	Random allocation: method not specified	
Data	Businesses from the state sales and use of licence records in the southwestern region of US	
Comparisons	1. US $1 bill 2. No incentive 3. Advance notice 4. No advance notice 5. Personalised salutation 6. No personalised salutation 7. Telephone follow‐up 8. No telephone follow‐up	
Outcomes	Response period not specified	
Topic	Non‐health: employment	
Mode of Administration	Postal	
Notes	2 X 2 X 2 X 2 X 2 fully crossed factorial design; mainly males	
***Risk of bias***		
**Item**	**Authors' judgement**	**Support for judgement**
Allocation concealment?	Unclear	B ‐ unclear

**Table:** Danko 2019

***Study characteristics***		
Methods	Computer‐generated random allocation sequence in R	
Data	Contact authors of all included studies (published studies on diabetes quality improvement interventions) in a systematic review who had not responded to initial email requests for information (international)	
Comparisons	Telephone reminder vs repeat email reminders only for non‐responders.	
Outcomes	Primary outcome was the response proportion, within 3 weeks, defined as the number of authors who completed the survey divided by the total number of authors assigned to the intervention. Tel intervention: authors called up to three times by telephone to request they complete online survey, and following up via email with the survey link and study PDF. Email intervention: up to three additional email requests (one request per week) to complete the survey. Emails included the survey link and study PDF.	
Topic	Health: diabetes	
Mode of Administration	Web‐based survey	
Notes	Questionnaire not described, but assumed under 20 questions on: study intervention components, populations, and settings Web‐based survey sent once a week via email to corresponding authors until they responded, requested not to be contacted further, or completed a maximum of three contact attempts. Emails to contact authors were sent from the email address of a senior investigator on the research team (J.M.G.). Provided an incentive: authors who completed the survey were entered in a draw for one of five $100 (CAD) gift certificates.	
***Risk of bias***		
**Item**	**Authors' judgement**	**Support for judgement**
Sequence generation	Yes	Computer‐generated random allocation sequence in R
Allocation concealment?	Yes	Computer‐generated random allocation sequence in R
Blinding of participants and personnel	Unclear	Blinding of personnel not specified. Participants were not blinded to intervention.
Blinding of outcome assessment	Yes	No outcome assessment used (counts of responses only)
Incomplete outcome data	Yes	Exclusions reported
Selective reporting	Yes	Outcomes reported in full
Other sources of bias	Yes	No grounds for concern about other sources of bias from reading the study report

**Table:** Deehan 1997

***Study characteristics***		
Methods	Random allocation: method not specified	
Data	GPs who did not respond to 2 mailings of a questionnaire (UK)	
Comparisons	Third mailing: 1. No incentive 2. £5 charity donation 3. £10 charity donation 4. £5 payment 5. £10 payment Fourth mailing to non‐responders in control group of third mailing: 1. £5 payment 2. £10 payment	
Outcomes	Response period not specified	
Topic	Health: information on clinical work with alcohol‐misusing patients	
Mode of Administration	Postal	
Notes	—	
***Risk of bias***		
**Item**	**Authors' judgement**	**Support for judgement**
Allocation concealment?	Unclear	B ‐ unclear

**Table:** Del Valle 1997

***Study characteristics***		
Methods	Random allocation: method not specified	
Data	Members of the American Association of Neurologists who did not respond to 2 earlier mailings	
Comparisons	1. Questionnaire sent by certified mail with return receipt request postcard 2. Questionnaire sent by first‐class mail	
Outcomes	Response period not specified	
Topic	—	
Mode of Administration	Postal	
Notes	Mainly males	
***Risk of bias***		
**Item**	**Authors' judgement**	**Support for judgement**
Allocation concealment?	Unclear	B ‐ unclear

**Table:** Delnevo 2004

***Study characteristics***		
Methods	Random allocation: using a statistical software package	
Data	New Jersey internists, general practitioners, family physicians, paediatricians, and obstetricians and gynaecologists	
Comparisons	1. Up‐front $25 gift card 2. Promised $25 gift card	
Outcomes	Response period not specified	
Topic	Health: smoking cessation ‐ attitudes and practice	
Mode of Administration	Postal	
Notes	The investigators were not blinded to the treatment allocation ‐ confirmed by the author	
***Risk of bias***		
**Item**	**Authors' judgement**	**Support for judgement**
Allocation concealment?	No	C ‐ inadequate

**Table:** Delnevo 2021

***Study characteristics***		
Methods	Random allocation: method unspecified	
Data	Practising physicians, US	
Comparisons	Postal invitation to Web survey vs. postal survey	
Outcomes	Response after 2 months	
Topic	Health (physicians' attitudes and beliefs regarding tobacco use, smoking cessation and electronic cigarettes)	
Mode of Administration	Postal & electronic	
Notes	Questionnaire not described 1st mailing ‐ cover letter, $25 gift card (coffee chain), paper survey for mail mode/web‐push received URL in cover letter. Reminder postcard after 1 week to non‐respondents, 3rd mailing as 1st, and 4th and final mailing to non‐responders included URL and paper questionnaire.	
***Risk of bias***		
**Item**	**Authors' judgement**	**Support for judgement**
Sequence generation	Unclear	Random allocation: method unspecified
Allocation concealment?	Unclear	Random allocation: method unspecified
Blinding of participants and personnel	Unclear	Blinding of personnel not described. Participants were not blinded to intervention.
Blinding of outcome assessment	Yes	No outcomes were assessed (counts of responses only)
Incomplete outcome data	Yes	158 cases were determined to be ineligible (i.e. death, retirement, no active medical license in state, not board certified, not providing outpatient care). More ineligible cases were identified in the web‐push condition (83 vs. 75).
Selective reporting	Yes	Response after 2 months reported in full
Other sources of bias	Yes	No grounds for concern about other sources of bias from reading the study report

**Table:** Denton 1988

***Study characteristics***		
Methods	Random allocation: method not specified	
Data	Graduates from the Department of Educational Curriculum and Instruction at a large university in the southwest (US)	
Comparisons	1. No incentive 2. Newsletter 3. $0.25 4. $0.25 and newsletter	
Outcomes	Response period not specified	
Topic	Non‐health: education	
Mode of Administration	Postal	
Notes	—	
***Risk of bias***		
**Item**	**Authors' judgement**	**Support for judgement**
Allocation concealment?	Unclear	B ‐ unclear

**Table:** Denton 1991

***Study characteristics***		
Methods	Random allocation: method not specified	
Data	Experiments 1 and 2: graduates from the Department of Educational Curriculum and Instruction at a large university in the south‐west (US)	
Comparisons	Experiment 1: 1. No incentive 2. Newsletter 3. $0.25 4. $0.25 and newsletter Experiment 2: 1. No incentive 2. $0.25 3. $0.50 4. $1 5. Raffle	
Outcomes	Experiment 2: response within 2 months	
Topic	Non‐health: classroom teachers pedagogical knowledge and skills	
Mode of Administration	Postal	
Notes	—	
***Risk of bias***		
**Item**	**Authors' judgement**	**Support for judgement**
Allocation concealment?	Unclear	B ‐ unclear

**Table:** Deutskens 2004a

***Study characteristics***		
Methods	Random allocation: using random number generation in SPSS	
Data	Participants of the multi‐client attitude and usage study in the Netherlands	
Comparisons	1. 2 euros vouchers for an online book and CD store 2. 5 euros vouchers for an online book and CD store 3. Lotteries to win vouchers worth 25 euros 4. Lotteries to win vouchers worth 50 euros 5. Charity donation of 500 euros to either World Wide Fund for Nature (WWF), Amnesty International, or a Cancer Association 6. Short version of the questionnaire 7. Long version of the questionnaire 8. Visual presentation of response categories 9. Textual presentation of response categories 10. Early follow‐up (after 1 week) 11. Late follow‐up (after 2 weeks)	
Outcomes	Response period not specified	
Topic	Non‐health: marketing	
Mode of Administration	Electronic: online survey	
Notes	3 X 2 X 2 X 2 factorial design Method of allocation ascertained through contact with author Age: Mostly 35‐49; mainly males	
***Risk of bias***		
**Item**	**Authors' judgement**	**Support for judgement**
Allocation concealment?	Yes	A ‐ adequate

**Table:** Deutskens 2004b

***Study characteristics***		
Methods	Random allocation: using random number generation in SPSS	
Data	University students	
Comparisons	1. Lottery to win 1 out of 10 vouchers of 25 euros 2. Lottery to win 1 out of 5 vouchers of 50 euros 3. Lottery to win a DVD player	
Outcomes	Response period not specified	
Topic	Non‐health: education	
Mode of Administration	Electronic: online	
Notes	Method of allocation ascertained through contact with author; mainly males	
***Risk of bias***		
**Item**	**Authors' judgement**	**Support for judgement**
Allocation concealment?	Yes	A: adequate

**Table:** Dillman 1974a

***Study characteristics***		
Methods	Random allocation: method not specified	
Data	A group of Washington State University alumni	
Comparisons	1. Personalised cover letter 2. Non‐personalised cover letter	
Outcomes	Response after 4 mailings	
Topic	Non‐health: feelings and concerns about Washington State University	
Mode of Administration	Postal	
Notes	Method of allocation ascertained to be random through contact with author	
***Risk of bias***		
**Item**	**Authors' judgement**	**Support for judgement**
Allocation concealment?	Unclear	B ‐ unclear

**Table:** Dillman 1974b

***Study characteristics***		
Methods	Random allocation: method not specified	
Data	A systematic sample of people listed in the phone directories of Washington state, USA	
Comparisons	1. No pre‐contact 2. Telephone pre‐contact	
Outcomes	Response period not specified	
Topic	Non‐health: feelings and concerns about Washington State University	
Mode of Administration	Postal	
Notes	Method of allocation ascertained to be random through contact with author	
***Risk of bias***		
**Item**	**Authors' judgement**	**Support for judgement**
Allocation concealment?	Unclear	B ‐ unclear

**Table:** Dillman 1993

***Study characteristics***		
Methods	Random allocation: method not specified	
Data	Housing units identified by the census bureau's address control file	
Comparisons	1. 1990 short form (control) questionnaire 2. Booklet 3. Micro form 4. Micro form requesting SSN 5. Roster form	
Outcomes	Response period not specified	
Topic	Non‐health: census	
Mode of Administration	Postal	
Notes	—	
***Risk of bias***		
**Item**	**Authors' judgement**	**Support for judgement**
Allocation concealment?	Unclear	B ‐ unclear

**Table:** Dillman 1996

***Study characteristics***		
Methods	Random allocation: method not specified	
Data	National probability sample of households in the USA	
Comparisons	1. Control group 2. Benefit appeal on envelope and insert; strong confidentiality assurance 3. Benefit appeal on envelope and insert; standard confidentiality assurance 4. Mandatory appeal on envelope and insert; strong confidentiality assurance 5. Mandatory appeal on envelope and insert; standard confidentiality assurance 6. Mandatory appeal on envelope only; no confidentiality assurance	
Outcomes	Response period not specified	
Topic	Non‐health: census	
Mode of Administration	Postal	
Notes	—	
***Risk of bias***		
**Item**	**Authors' judgement**	**Support for judgement**
Allocation concealment?	Unclear	D ‐ not used

**Table:** Dinglas 2015

***Study characteristics***		
Methods	Random allocation: computer‐generated random numbers	
Data	Acute lung injury survivors who were enrolled in randomised trials of novel interventional therapies funded by the National Heart, Lung, and Blood Institute (NHLBI) ARDS Network, US	
Comparisons	Personalised, stamped envelopes vs. typed, franked envelopes	
Outcomes	Response after 12 weeks (maximum of 4 biweekly mailings and 4 weekly phone calls)	
Topic	Health (outcomes in acute lung injury survivors)	
Mode of Administration	Postal	
Notes	One‐page health insurance survey Participants were contacted using a multi‐step, structured protocol starting with mailed letters and then telephone calls, as needed for non‐responders to mailings. Participants were mailed the insurance survey every 2 weeks until the survey was completed or the participant was sent a total of 4 mailings. For these mailings, trial participants were randomised to receive either a personal format letter in which their mailing address and the return address were handwritten and a traditional stamp was stamped using the envelope versus a business format letter in which the addresses were typed and the postage was affixed by a commercial stamp‐machine. In all other respects, the envelopes were identical (i.e. 9 x 12‐inch manila envelopes) and included an identical cover letter, insurance survey, and SAE. Starting 20 days after the end of the mail trial, a telephone trial was initiated.	
***Risk of bias***		
**Item**	**Authors' judgement**	**Support for judgement**
Sequence generation	Yes	Randomisation was performed by a statistician using computer‐generated random numbers.
Allocation concealment?	Yes	Randomisation was performed by a statistician using computer‐generated random numbers.
Blinding of participants and personnel	Unclear	Blinding of personnel not described. Participants were not blinded to intervention.
Blinding of outcome assessment	Yes	No outcomes were assessed (counts of responses only).
Incomplete outcome data	Yes	Outcomes reported in full including attrition and exclusions
Selective reporting	Yes	Responses after 4 contacts reported
Other sources of bias	Yes	No grounds for concern about other sources of bias from reading the study report

**Table:** Dirmaier 2007

***Study characteristics***		
Methods	Random allocation: using a computer‐assisted algorithm	
Data	Patients admitted for psychotherapeutic treatment	
Comparisons	1. Long questionnaire 2. Short questionnaire 3. 5 German Mark bill 4. No incentive	
Outcomes	Response period not specified	
Topic	Health: mental health outcome and treatment research	
Mode of Administration	Postal	
Notes	Age: Mostly 40‐59; mainly females	
***Risk of bias***		
**Item**	**Authors' judgement**	**Support for judgement**
Sequence generation	Yes	Random allocation: Patients were randomised using a computer‐assisted algorithm.
Allocation concealment?	Yes	Random allocation: Patients were randomised using a computer‐assisted algorithm.
Blinding of participants and personnel	Unclear	Blinding of personnel not described. Participants were not blinded to intervention.
Blinding of outcome assessment	Yes	No outcomes were assessed (counts of responses only).
Incomplete outcome data	No	Exclusions not reported
Selective reporting	Yes	Response after one reminder at 4 weeks reported fully
Other sources of bias	Yes	No grounds for concern about other sources of bias from reading the study report

**Table:** Dodd 1987

***Study characteristics***		
Methods	Random allocation: using a table of random numbers	
Data	Women employed full‐time in various civil servant positions at a university (US)	
Comparisons	1. Hand‐signed, professor status, female author 2. Hand‐signed, student status, female author 3. Hand‐signed, professor status, male author 4. Hand‐signed, student status, male author 5. Photocopied signature, professor status, female author 6. Photocopied signature, student status, female author 7. Photocopied signature, professor status, male author 8. Photocopied signature, student status, male author	
Outcomes	Response period not specified	
Topic	Not specified	
Mode of Administration	Postal	
Notes	Method of allocation ascertained through contact with author. Allocation concealment not described; mean age: 42 years; mainly females	
***Risk of bias***		
**Item**	**Authors' judgement**	**Support for judgement**
Allocation concealment?	Unclear	B ‐ unclear

**Table:** Doerfling 2010

***Study characteristics***		
Methods	Random allocation: method unspecified	
Data	Members of the Canadian Association of Retired Persons.	
Comparisons	$1000 lottery incentive vs. no incentive	
Outcomes	Outcomes: clicked on hyperlink and completed Survey (responses after two weeks with 2 reminder emails)	
Topic	Health (survey of physical activity and joint health)	
Mode of Administration	Electronic	
Notes	33 questions on demographics, socioeconomic status, physical activity (sport, occupational and domestic), diagnosis of arthritis, previous and current knee pain, and computer usage Emails contained a personal letter from principal investigator, a short description of the study, a contact email address for questions. Half of the subjects were randomly allocated to receive an email that contained the incentive, while the other half received a message with no financial incentive.	
***Risk of bias***		
**Item**	**Authors' judgement**	**Support for judgement**
Sequence generation	Unclear	Random allocation: method unspecified
Allocation concealment?	Unclear	Random allocation: method unspecified
Blinding of participants and personnel	Unclear	Blinding of personnel not described. Participants were not blinded to intervention.
Blinding of outcome assessment	Yes	No outcomes were assessed (counts of responses only).
Incomplete outcome data	No	Exclusions not reported
Selective reporting	Yes	Responses after two weeks with 2 reminder emails reported in full
Other sources of bias	Yes	No grounds for concern about other sources of bias from reading the study report

**Table:** Dommeyer 1980a

***Study characteristics***		
Methods	Random allocation: method not specified	
Data	Individuals listed in the Cincinnati telephone directory (US)	
Comparisons	1. Questionnaire with low threat follow‐up 2. Questionnaire with low‐moderate follow‐up 3. Questionnaire with low‐moderate follow‐up (different to above) 4. Questionnaire with moderate follow‐up 5. Questionnaire with follow‐up with moderate appeal 6. Questionnaire with prepaid incentive of 25 cents in follow‐up 7. Personally asked to compare the relative noxiousness of the threat of appeals sent to groups 1‐4 Non‐respondents to the initial mailings were followed up	
Outcomes	Response within 31 days	
Topic	Non‐health: attitudes towards questionnaire, socio‐demographics	
Mode of Administration	Postal	
Notes	—	
***Risk of bias***		
**Item**	**Authors' judgement**	**Support for judgement**
Allocation concealment?	Unclear	B ‐ unclear

**Table:** Dommeyer 1980b

***Study characteristics***		
Methods	Random allocation: alternation	
Data	Individuals listed in the Cincinnati telephone directory (US)	
Comparisons	1. Questionnaire with ID number typed on lower righthand corner of last page 2. As above, with words: 'Please do not remove identifying code number' typed next to the ID number	
Outcomes	Response within 13 days	
Topic	Non‐health: attitudes towards questionnaire, sociodemographics	
Mode of Administration	Postal	
Notes	—	
***Risk of bias***		
**Item**	**Authors' judgement**	**Support for judgement**
Allocation concealment?	No	C ‐ inadequate

**Table:** Dommeyer 1985

***Study characteristics***		
Methods	Random allocation: method not specified	
Data	Undergraduate business students (US)	
Comparisons	1. Interesting questionnaire; no summary of results offered 2. Interesting questionnaire; results summary offered 3. Uninteresting questionnaire; no summary of results offered 4. Uninteresting questionnaire; results summary offered	
Outcomes	—	
Topic	Non‐health: finance ‐ tax survey; Mind Inventory Catalogue	
Mode of Administration	Postal	
Notes	—	
***Risk of bias***		
**Item**	**Authors' judgement**	**Support for judgement**
Allocation concealment?	Unclear	B ‐ unclear

**Table:** Dommeyer 1987

***Study characteristics***		
Methods	Random allocation using alternation	
Data	Telephone owners in Cincinnati	
Comparisons	1. Negative appeal mention of follow‐up 2. Usual mail 3. Prepaid incentive of 25 cents each	
Outcomes	Response to be received by 28th June	
Topic	Non‐health: attitudes and familiarity towards mail; education, employment	
Mode of Administration	Postal	
Notes	Method of allocation ascertained through contact with author	
***Risk of bias***		
**Item**	**Authors' judgement**	**Support for judgement**
Allocation concealment?	No	C ‐ inadequate

**Table:** Dommeyer 1988

***Study characteristics***		
Methods	Random allocation: method not specified	
Data	Individuals listed in the Chicago and Phoenix telephone directory	
Comparisons	Different postscripts used in letter depending on intervention: 1. No incentive 2. 25 cent coin 3. 25 cent cheque 4. 25 cent money order 5. Early bird ‐ get a share in an incentive ($25) if send questionnaire back quickly 6. Sweepstake (entered into sweepstake to win $25 if return questionnaire by deadline) All participants sent cover letter and questionnaire in window envelope	
Outcomes	—	
Topic	Non‐health: product tampering and Morality Conscience Guilt Scale	
Mode of Administration	Postal	
Notes	—	
***Risk of bias***		
**Item**	**Authors' judgement**	**Support for judgement**
Allocation concealment?	Unclear	B ‐ unclear

**Table:** Dommeyer 1989

***Study characteristics***		
Methods	Random allocation: method not specified	
Data	Personal computer owners, manufacturers and retailers	
Comparisons	1. Cover letter stressed importance of response and emphasised that respondents' names would never be placed on the questionnaire (control group). 2. Second paragraph offered respondents a summary of the results. 3. Standard cover letter. Offer of a copy of the results made in a separate 'lift' letter	
Outcomes	Response within 3 weeks	
Topic	Non‐health: knowledge and attitudes towards computer counterfeiting	
Mode of Administration	Postal	
Notes	—	
***Risk of bias***		
**Item**	**Authors' judgement**	**Support for judgement**
Allocation concealment?	Unclear	B ‐ unclear

**Table:** Dommeyer 1991

***Study characteristics***		
Methods	Random allocation: method not specified	
Data	Californian residents who were entitled to a refund	
Comparisons	1. Teaser printed on envelope 2. No teaser on envelope	
Outcomes	Response within 2 weeks	
Topic	Non‐health: finance ‐ awareness and attitudes towards insurance refunds	
Mode of Administration	Postal	
Notes	"A teaser is typically a short question printed on the outer envelope that is designed to lure the recipient into the examining [of] the content of the envelope".	
***Risk of bias***		
**Item**	**Authors' judgement**	**Support for judgement**
Allocation concealment?	Unclear	B ‐ unclear

**Table:** Dommeyer 1996

***Study characteristics***		
Methods	Random allocation: method not specified	
Data	People listed in a telephone directory (Los Angeles, USA)	
Comparisons	1. Photograph of an 'attractive' researcher printed on cover letter 2. No photo printed on cover letter	
Outcomes	—	
Topic	Non‐health: attitudes towards music censorship and warning stickers on music albums	
Mode of Administration	Postal	
Notes	—	
***Risk of bias***		
**Item**	**Authors' judgement**	**Support for judgement**
Allocation concealment?	Unclear	B ‐ unclear

**Table:** Dommeyer 2004

***Study characteristics***		
Methods	Random allocation: using randomised incomplete block design	
Data	Undergraduate business major students at California State University	
Comparisons	1. Grade incentive 2. In‐class demonstration of the web survey 3. Early grade feedback 4. Control	
Outcomes	Response period not specified	
Topic	Non‐health: education	
Mode of Administration	Electronic: online survey	
Notes	—	
***Risk of bias***		
**Item**	**Authors' judgement**	**Support for judgement**
Allocation concealment?	Unclear	B ‐ unclear

**Table:** Donaldson 1999

***Study characteristics***		
Methods	Random allocation: method not specified	
Data	Physicians randomly selected from a list of US physicians actively caring for at least 1 transplant patient	
Comparisons	1. $5 check with initial mailing; no follow‐up call 2. $5 check with initial mailing; follow‐up call to non‐responders 4 weeks after initial mailing 3. No incentive; no follow‐up call 4. No incentive; follow‐up call to non‐responders 4 weeks after initial mailing	
Outcomes	—	
Topic	Health	
Mode of Administration	Postal	
Notes	Mean age: 47 years; mainly males	
***Risk of bias***		
**Item**	**Authors' judgement**	**Support for judgement**
Allocation concealment?	Unclear	B ‐ unclear

**Table:** Doob 1971a

***Study characteristics***		
Methods	Random allocation: method not specified	
Data	Individuals listed in a phone book (Toronto and Ontario, Canada)	
Comparisons	1. No reactance (letter written normally); no incentive 2. No reactance; dime incentive 3. Reactance (request written to make participants feel an attempt was being made to limit their freedom); no money 4. Reactance; dime incentive	
Outcomes	Response within 2 weeks	
Topic	Health: knowledge that smoking causes cancer	
Mode of Administration	Postal	
Notes	—	
***Risk of bias***		
**Item**	**Authors' judgement**	**Support for judgement**
Allocation concealment?	Unclear	B ‐ unclear

**Table:** Doob 1971b

***Study characteristics***		
Methods	Random allocation: method not specified	
Data	Individuals listed in a phone book (Toronto and Ontario, Canada)	
Comparisons	1. No reactance (letter written normally); no incentive 2. No reactance; dime incentive 3. Reactance (request written to make participants feel an attempt was being made to limit their freedom); no money 4. Reactance; dime incentive	
Outcomes	Response within 2 weeks	
Topic	Health: knowledge that smoking causes cancer	
Mode of Administration	Postal	
Notes	—	
***Risk of bias***		
**Item**	**Authors' judgement**	**Support for judgement**
Allocation concealment?	Unclear	B ‐ unclear

**Table:** Doob 1971c

***Study characteristics***		
Methods	Random allocation: method not specified	
Data	Individuals listed in a phone book (Toronto and Ontario, Canada)	
Comparisons	1. No reactance (letter written normally); no incentive 2. No reactance; 20 cents incentive 3. Reactance (request written to make participants feel an attempt was being made to limit their freedom); no money 4. Reactance; 20 cents incentive	
Outcomes	Response within 2 weeks	
Topic	Health: knowledge that smoking causes cancer	
Mode of Administration	Postal	
Notes	—	
***Risk of bias***		
**Item**	**Authors' judgement**	**Support for judgement**
Allocation concealment?	Unclear	B ‐ unclear

**Table:** Doob 1973

***Study characteristics***		
Methods	Random allocation: method not specified	
Data	Individuals listed in a telephone directory (Canada)	
Comparisons	1. 20 cents incentive 2. 5 cents incentive 3. No incentive 1. University sponsor 2. Industrial sponsor	
Outcomes	Response period not specified	
Topic	Non‐health: automobile ownership, duration spent on watching TV	
Mode of Administration	Postal	
Notes	—	
***Risk of bias***		
**Item**	**Authors' judgement**	**Support for judgement**
Allocation concealment?	Unclear	B ‐ unclear

**Table:** Doody 2003a

***Study characteristics***		
Methods	Random allocation: method not specified	
Data	US radiologist technologists who had not responded to 2 earlier mailings of a questionnaire	
Comparisons	1. US first‐class mail; no incentive 2. US first‐class mail; $1 bill 3. US first‐class mail; $2 bill 4. US first‐class mail; $2 check 5. US first‐class mail; $5 check	
Outcomes	Response period not specified	
Topic	Health	
Mode of Administration	Postal	
Notes	All subjects received a pre‐notification letter; age: mostly 40‐49 years; mainly females	
***Risk of bias***		
**Item**	**Authors' judgement**	**Support for judgement**
Sequence generation	Unclear	Random allocation: method unspecified
Allocation concealment?	Unclear	Random allocation: method unspecified
Blinding of participants and personnel	Unclear	Blinding of personnel not described. Participants were not blinded to intervention
Blinding of outcome assessment	Yes	No outcomes were assessed (counts of responses only).
Incomplete outcome data	Yes	Exclusions reported
Selective reporting	Yes	Outcomes reported in full
Other sources of bias	Yes	No grounds for concern about other sources of bias from reading the study report

**Table:** Doody 2003b

***Study characteristics***		
Methods	Random allocation: method not specified	
Data	US radiologist technologists who had not responded to 2 earlier mailings of a questionnaire	
Comparisons	1. Federal express; no incentive 2. Federal express; $1 bill 3. Federal express; $2 bill 4. Federal express; $2 check	
Outcomes	Response period not specified	
Topic	Health	
Mode of Administration	Postal	
Notes	Age: mostly 40‐49 years; mainly females	
***Risk of bias***		
**Item**	**Authors' judgement**	**Support for judgement**
Sequence generation	Unclear	Random allocation: method unspecified
Allocation concealment?	Unclear	Random allocation: method unspecified
Blinding of participants and personnel	Unclear	Blinding of personnel not described. Participants were not blinded to intervention.
Blinding of outcome assessment	Yes	No outcomes were assessed (counts of responses only).
Incomplete outcome data	Yes	Exclusions reported
Selective reporting	Yes	Outcomes reported in full
Other sources of bias	Yes	No grounds for concern about other sources of bias from reading the study report

**Table:** Dorman 1997

***Study characteristics***		
Methods	Random allocation: using an allocation code generated by an adaptive randomisation algorithm	
Data	Patients who had been entered into the International stroke trial between 2 March 1993 and 31 May 1995	
Comparisons	1. Questionnaire incorporating the EuroQoL 2. Questionnaire incorporating the SF‐36 Questionnaires were identical in all respects other than the nature of the HRQoL instrument. EuroQoL has 7 questions, SF‐36 has 36. Both had same number of pages, but the first questionnaire had fewer questions. Reminders sent to non‐responders after 2 weeks	
Outcomes	—	
Topic	Health: SF‐36, EuroQoL	
Mode of Administration	Postal	
Notes	The randomisation algorithm used aimed to balance the 2 groups for age, sex, stroke syndrome and the time from stroke onset to follow‐up	
***Risk of bias***		
**Item**	**Authors' judgement**	**Support for judgement**
Allocation concealment?	Yes	A ‐ adequate

**Table:** Downes‐Le Guin 2002

***Study characteristics***		
Methods	Random allocation: using random number generation	
Data	IT managers in US businesses	
Comparisons	1. Unconditional Amazon gift certificate ($15) 2. Unconditional Amazon gift certificate ($25) 3. Conditional Amazon gift certificate ($15) 4. Conditional Amazon gift certificate ($25)	
Outcomes	Response period not specified	
Topic	Non‐health: marketing	
Mode of Administration	Electronic: online survey	
Notes	Method of allocation ascertained through contact with author	
***Risk of bias***		
**Item**	**Authors' judgement**	**Support for judgement**
Allocation concealment?	Yes	A ‐ adequate

**Table:** Drummond 2008

***Study characteristics***		
Methods	Random allocation: using random number generation	
Data	Primary care physicians working in Ireland	
Comparisons	1. Pre‐contact via mail 2. No pre‐contact 3. Questionnaire order: version 1, demographics first 4. Questionnaire order: version 2, topic‐specific questions first (prostate‐specific antigen testing)	
Outcomes	Response period not specified	
Topic	Health: views and practices about prostate‐specific testing (PSA)	
Mode of Administration	Postal	
Notes	Method of allocation ascertained through contact with author; mainly males	
***Risk of bias***		
**Item**	**Authors' judgement**	**Support for judgement**
Sequence generation	Yes	Random allocation: computer‐generated random numbers
Allocation concealment?	Yes	Random allocation: computer‐generated random numbers
Blinding of participants and personnel	Unclear	Blinding of personnel not described. Participants were not blinded to intervention.
Blinding of outcome assessment	Yes	No outcomes were assessed (counts of responses only).
Incomplete outcome data	Yes	During the trial, 132 of the 1599 samples were found to be ineligible and were excluded; 65 had retired, 38 had died, and 29 did not see appropriate patients.
Selective reporting	Yes	Response after 2 reminders (8 weeks) reported in full
Other sources of bias	Yes	No grounds for concern about other sources of bias from reading the study report

**Table:** Drummond 2014

***Study characteristics***		
Methods	Random allocation: method unspecified	
Data	1650 Irish general practitioners	
Comparisons	Unconditional monetary incentive (5 EU) vs. no incentive; Conditional non‐monetary incentive (1 in 50 chance for 300 EU) vs. no incentive	
Outcomes	Response after 2 reminders	
Topic	Health (primary care physicians' practice and costs in relation to prostate‐specific antigen (PSA) testing)	
Mode of Administration	Postal	
Notes	20‐item survey over 2 pages With their questionnaire, primary care physicians received: (1) EUR 5 and a cover letter stating that this was a token of appreciation (cash arm); (2) a cover letter stating that they would be entered into a draw for one of three EUR 300 vouchers, with a 1‐in‐50 chance of winning, on return of completed questionnaire (prize arm); or (3) a cover letter (no‐incentive arm). Comparison of unconditional vs. unconditional incentives is confounded by amount. Primary care physicians received personalised letters on university‐headed paper and questionnaires were printed with coloured ink on coloured paper. Prepaid preaddressed envelopes for questionnaire return were included with each mailing. Up to two written reminders were sent to non‐responders at approximately two‐weekly intervals with another questionnaire in the second reminder. Both reminder letters to the prize arm mentioned the incentive. No reference to the EUR 5 was made in reminder letters to the cash arm.	
***Risk of bias***		
**Item**	**Authors' judgement**	**Support for judgement**
Sequence generation	Unclear	Random allocation: method unspecified
Allocation concealment?	Unclear	Random allocation: method unspecified
Blinding of participants and personnel	Unclear	Blinding of personnel not described. Participants were not blinded to intervention.
Blinding of outcome assessment	Yes	No outcomes were assessed (counts of responses only).
Incomplete outcome data	Yes	After questionnaire dispatch, PCPs who died, retired, relocated (and no forwarding address was available), and those without male patients aged 40 years and older (n = 5223) were deemed ineligible and removed from the denominator of the relevant arm.
Selective reporting	Yes	Response after 2 reminders reported in full
Other sources of bias	Yes	No grounds for concern about other sources of bias from reading the study report

**Table:** Duffy 2001

***Study characteristics***		
Methods	Random allocation: twin numbered 01 in each pair received single stamp; the other received the intervention	
Data	Twins who are volunteer members of the Australian NHMRC Twin Registry	
Comparisons	1. Single stamp on enclosed return envelope 2. Multiple stamps (3‐5) on enclosed return envelope	
Outcomes	Response period not specified	
Topic	Health: asthma, psoriasis	
Mode of Administration	Postal	
Notes	—	
***Risk of bias***		
**Item**	**Authors' judgement**	**Support for judgement**
Allocation concealment?	No	C ‐ inadequate

**Table:** Duhan 1990

***Study characteristics***		
Methods	Random allocation: method not specified	
Data	Industrial marketing executives	
Comparisons	1. Pre‐notification 2. No prior notification	
Outcomes	Response period not specified	
Topic	Not specified	
Mode of Administration	Postal	
Notes	Method of allocation ascertained through contact with author	
***Risk of bias***		
**Item**	**Authors' judgement**	**Support for judgement**
Allocation concealment?	Unclear	B ‐ unclear

**Table:** Dunn 2003

***Study characteristics***		
Methods	Random allocation: using random number generation	
Data	Patients aged 30‐59 years with back pain in the UK	
Comparisons	1. Traditional questionnaire ‐ generic questionnaires first followed by disease‐specific ones 2. Chronological questionnaire ‐ individual questions arranged in sections according to the period of time that they ask about	
Outcomes	Response period not specified	
Topic	Health: patient‐based outcome measures ‐ chronic pain grade, SF‐36, Hospital & Anxiety Scale, Roland‐Morris Disability Questionnaire	
Mode of Administration	Postal	
Notes	Mean age: 45 years	
***Risk of bias***		
**Item**	**Authors' judgement**	**Support for judgement**
Allocation concealment?	Yes	A ‐ adequate

**Table:** Dykema 2011

***Study characteristics***		
Methods	Random allocation: method unspecified.	
Data	Doctors of internal medicine from the American Medical Association Masterfilm, US	
Comparisons	Pre‐notification letter with $2 incentive vs. pre‐notification letter only Pre‐notification letter vs. none Non‐monetary incentive ($200 lottery, conditional $50 or $100 cheque) vs. no incentive	
Outcomes	Response after 4 email reminders and a postal letter with URL	
Topic	Health: physicians understanding of human genetic variation	
Mode of Administration	Electronic (Web survey)	
Notes	80‐item survey. Study procedures included up to seven points of contact with sample members. First, selected respondents were mailed a postal pre‐notification letter that used study‐specific stationery, bore sponsors' names, described the study's purpose, and noted respondents would be sent an email invitation to complete the web survey. A $2 bill for respondents assigned to the prepaid group. Approximately 1 week later, all respondents were sent an email invitation to participate that included a hot‐linked (clickable) URL. For the third to sixth contacts, email reminders containing the hotlinked URL were sent. For the seventh contact, non‐responding physicians were sent a postal letter that included a manual URL, which the physician could type into a browser.	
***Risk of bias***		
**Item**	**Authors' judgement**	**Support for judgement**
Sequence generation	Unclear	Random allocation: method unspecified
Allocation concealment?	Unclear	Random allocation: method unspecified
Blinding of participants and personnel	Unclear	Blinding of personnel not described. Participants were not blinded to intervention.
Blinding of outcome assessment	Yes	No outcomes were assessed (counts of responses only).
Incomplete outcome data	No	Exclusions not reported
Selective reporting	Yes	Outcomes reported
Other sources of bias	Yes	No grounds for concern about other sources of bias from reading the study report

**Table:** Dykema 2012

***Study characteristics***		
Methods	Random allocation: method unspecified	
Data	Non‐Hispanic African‐American mothers participating in the Pregnancy Risk Assessment Monitoring System, US	
Comparisons	Monetary incentive ($5) vs. non‐monetary ($6 diaper coupon) Monetary incentive ($5) vs. none Non‐monetary incentive ($6 diaper coupon) vs. none	
Outcomes	Response after 3 postal reminders and one phone reminder	
Topic	Health (attitudes and behaviours of mothers before, during, and after pregnancy)	
Mode of Administration	Postal	
Notes	An introductory pre‐notification letter was mailed to each woman in the sample 24 months after the infants' birth. The initial PRAMS packet was mailed a few days later. The packet contained several items including a 13‐page questionnaire, cover letter, consent form, FAQ, and token inserts (a calendar, door hanger, brochure for the Wisconsin Maternal and Child Health Hotline, post‐it notes, and bath thermometer). Following the initial mailing, mothers were sent a note thanking those who had participated and reminding those who had not. Non‐responders received up to two additional mailings. After three mailings, we attempted to contact the remaining non‐respondents by telephone to complete the interview by phone.	
***Risk of bias***		
**Item**	**Authors' judgement**	**Support for judgement**
Sequence generation	Unclear	Random allocation: method unspecified
Allocation concealment?	Unclear	Random allocation: method unspecified
Blinding of participants and personnel	Yes	Telephone interviewers who called non‐respondents to the mail survey were blind to the experimental conditions, and respondents were not reminded of the incentive in the phone phase.
Blinding of outcome assessment	Yes	No outcomes were assessed (counts of responses only).
Incomplete outcome data	Yes	Exclusions reported
Selective reporting	Yes	Response after 3 postal reminders and one phone reminder reported in full
Other sources of bias	Yes	No grounds for concern about other sources of bias from reading the study report

**Table:** Dykema 2013

***Study characteristics***		
Methods	Random allocation: method unspecified	
Data	University faculty members from departments in biology, chemistry, mathematics, physics, and physiology at the University of Wisconsin‚ US	
Comparisons	Mailed invitation letter (with URL); vs. emailed invitation (clickable URL) Unconditional $2 cash incentive vs. no incentive	
Outcomes	Responses after initial invitation and responses after 2 email reminders	
Topic	Non‐health (attitudes and beliefs about the challenges and constraints faced when teaching in science, technology, engineering and mathematics (STEM) fields)	
Mode of Administration	Electronic (Web survey)	
Notes	151 questions, although the number of questions answered by any one respondent varied considerably due to skip patterns The letter was printed on study‐specific stationery, described the study‚ purpose and funder, and included a URL and authentication credentials, which the sample member could manually type into a browser in order to access the survey instrument. The mailed invitation letters in the incentive and no incentive groups were identical except that the former made reference to the incentive using the text, 'Please accept the enclosed $2.00 as our way of thanking you in advance for your participation'. The mailed invitation was sent first class via the U.S. Postal Service to sample members‚ postal addresses on campus. Email invitations were sent 5 days after the mailed invitations. They contained a clickable URL with embedded authentication credentials as well as a description of the study and funder. The email's subject line referenced the content of the survey (e.g. 'Culture, Cognition and Evaluation of STEM Higher Education Reform Survey') All sample members had campus postal and email addresses on file. The questionnaire could only be completed over the Internet. The survey was administered by the University of Wisconsin Survey Center from December 2009 to January 2010 on behalf of the University of Wisconsin‚ Center for Education Research.	
***Risk of bias***		
**Item**	**Authors' judgement**	**Support for judgement**
Sequence generation	Unclear	Random allocation: method unspecified
Allocation concealment?	Unclear	Random allocation: method unspecified
Blinding of participants and personnel	Unclear	Blinding of personnel not described. Participants were not blinded to intervention.
Blinding of outcome assessment	Yes	No outcomes were assessed (counts of responses only).
Incomplete outcome data	No	Exclusions not reported
Selective reporting	Yes	Outcomes reported in full
Other sources of bias	Yes	No grounds for concern about other sources of bias from reading the study report

**Table:** Dykema 2015a

***Study characteristics***		
Methods	Random allocation: method unspecified	
Data	2608 household addresses randomly selected from 2 counties in Wisconsin (participants were aged between 18 and 74 years), US	
Comparisons	Unconditional $2 pre‐incentive vs. $5 Message on envelope ('Thank You! A cash gift is enclosed') vs. no message Unconditional $2 on follow‐up vs. no incentive	
Outcomes	Response after 2 reminders over 2 months	
Topic	Health (health‐ and community‐related topics, including health behaviours, food purchasing routines, and community satisfaction)	
Mode of Administration	Postal	
Notes	124 items on 12 pages in La Crosse county; 164 items on 18 pages in Wood county Sampled households received up to three contacts by mail. The initial packet contained a cover letter bearing the county seal, cash incentive, questionnaire, and self‐addressed first‐class stamped return envelope. Materials were sent in a 10" x 13" envelope with a first‐class stamp. Approximately a week after the initial mailing, all households received a reminder postcard. Approximately one month after the initial mailing, all non‐responders were sent an additional packet containing a cover letter, second incentive (if relevant), questionnaire, and return envelope.	
***Risk of bias***		
**Item**	**Authors' judgement**	**Support for judgement**
Sequence generation	Unclear	Random allocation: method unspecified
Allocation concealment?	Unclear	Random allocation: method unspecified
Blinding of participants and personnel	Unclear	Blinding of personnel not described. Participants were not blinded to intervention.
Blinding of outcome assessment	Yes	No outcomes were assessed (counts of responses only).
Incomplete outcome data	Yes	Participants not eligible reported
Selective reporting	Yes	Response after 2 reminders over 2 months reported in full
Other sources of bias	Yes	No grounds for concern about other sources of bias from reading the study report

**Table:** Dykema 2015b

***Study characteristics***		
Methods	Random allocation: method unspecified	
Data	2608 household addresses randomly selected from 2 counties in Wisconsin (participants were aged between 18 and 74 years), US	
Comparisons	Envelope with no message vs. monetary incentive message vs. health message follow‐up prepaid $0 incentive vs. follow‐up prepaid $5	
Outcomes	Response after 2 reminders over 2 months	
Topic	Health (health‐ and community‐related topics, including health behaviours, food purchasing routines, and community satisfaction)	
Mode of Administration	Postal	
Notes	Questionnaires varied between the counties (124 items on 12 pages in La Crosse versus 164 items on 18 pages in Wood). All study materials were written and completed in English. Sampled households received up to three contacts by mail. The initial packet contained a cover letter bearing the county seal, cash incentive, questionnaire, and self‐addressed first‐class stamped return envelope. Materials were sent in a 10" x 13" envelope with a first‐class stamp. Approximately a week after the initial mailing, all households received a reminder postcard. Approximately one month after the initial mailing, all non‐responders were sent an additional packet containing a cover letter, second incentive (if relevant), questionnaire, and return envelope. The target population for Dykema 2015b was identical to Dykema 2015a: adults (18–74 years) residing in the same two targeted counties. The design of Dykema 2015b mirrored Dykema 2015a.	
***Risk of bias***		
**Item**	**Authors' judgement**	**Support for judgement**
Sequence generation	Unclear	Random allocation: method unspecified
Allocation concealment?	Unclear	Random allocation: method unspecified
Blinding of participants and personnel	Unclear	Blinding of personnel not described. Participants were not blinded to intervention.
Blinding of outcome assessment	Yes	No outcomes were assessed (counts of responses only).
Incomplete outcome data	Yes	Participants not eligible reported
Selective reporting	Yes	Response after 2 reminders over 2 months reported in full
Other sources of bias	Yes	No grounds for concern about other sources of bias from reading the study report

**Table:** Dykema 2021

***Study characteristics***		
Methods	Random allocation: method unspecified	
Data	1500 practising paediatricians, US	
Comparisons	Unconditional $5 monetary incentive in first mailing vs. unconditional $10 monetary incentive in first mailing	
Outcomes	Response after 3 mailings over 3 months	
Topic	Health (paediatricians' attitudes, training needs, and practices regarding advising parents about child discipline and related parenting issues)	
Mode of Administration	Postal	
Notes	84 items formatted on 8 pages. Sampled physicians received up to 4 contacts by mail. The initial mailing packet contained a cover letter, cash incentive, questionnaire, and a self‐addressed first‐class‐stamped return envelope. Materials were sent in a 10" x 13" envelope with a first‐class stamp. Approximately a week after the initial mailing, a reminder postcard sent. 1 month after the initial mailing, non‐responders sent a second mailing containing a cover letter, second incentive (if relevant), questionnaire, and return envelope. Approximately 3 weeks later, all remaining non‐responders were sent a final mailing packet that contained a cover letter, questionnaire, and return envelope. The field period extended from March to June 2016.	
***Risk of bias***		
**Item**	**Authors' judgement**	**Support for judgement**
Sequence generation	Unclear	Random allocation: method unspecified
Allocation concealment?	Unclear	Random allocation: method unspecified
Blinding of participants and personnel	Unclear	Blinding of personnel not described. Participants were not blinded to intervention.
Blinding of outcome assessment	Yes	No outcomes were assessed (counts of responses only).
Incomplete outcome data	Yes	Exclusions reported
Selective reporting	Yes	Outcomes reported in full
Other sources of bias	Unclear	No grounds for concern about other sources of bias from reading the study report

**Table:** Eaker 1998

***Study characteristics***		
Methods	Random allocation: method not specified	
Data	Men and women living in Sweden in 1995	
Comparisons	1. Preliminary notification (PN); long questionnaire (LQ); mention of telephone contact (MTC) 2. PN; short questionnaire (SQ); MTC 3. PN; LQ; no MTC 4. PN; SQ; no MTC 5. No PN; LQ; MTC 6. No PN; SQ; no MTC 7. No PN; LQ; no MTC 8. No PN; SQ; no MTC Reminders sent to all after 1 week	
Outcomes	Response within 75 days	
Topic	Health: medical history, physical activity, eating and drinking habits, reproductive history	
Mode of Administration	Postal	
Notes	Age: Mostly above 45 years; equal male and females	
***Risk of bias***		
**Item**	**Authors' judgement**	**Support for judgement**
Allocation concealment?	Unclear	B ‐ unclear

**Table:** Easton 1997

***Study characteristics***		
Methods	Random allocation: method not specified	
Data	Paediatricians listed in the American Academy of Paediatrics Directory	
Comparisons	1. Information booklet 2. $1 incentive	
Outcomes	Response period not specified	
Topic	Health: counselling about sun protection	
Mode of Administration	Postal	
Notes	—	
***Risk of bias***		
**Item**	**Authors' judgement**	**Support for judgement**
Allocation concealment?	Unclear	B ‐ unclear

**Table:** Edelman 2013

***Study characteristics***		
Methods	Random allocation: online random number generator	
Data	Rural older (> 65 yrs) adults participating in a rural county home‐delivered meals programme, US	
Comparisons	Hand‐delivered by someone known vs. by Postal Service delivery	
Outcomes	Response after one reminder	
Topic	Health (injury risk for older adults)	
Mode of Administration	Postal/electronic	
Notes	The survey was designed with older adults in mind and incorporated some strategies to improve recruitment, including a sans serif typeface, a minimum font size of 12 points, double‐spacing, and white‐space allowances (National Institute on Aging, 2012). The survey contained 48 closed‐ended items and one item asking if respondents would rather answer a paper‐ or Web‐based survey. Letter printed on the principal investigator's official stationery and signed by the investigator, the questionnaire, and an SAE. Pre‐notification letters, followed by the survey 5 days later, and a reminder/thank‐you postcard 6 days after the survey. Each questionnaire was numbered according to whether it was hand or postal delivered. The return date was recorded for each survey.	
***Risk of bias***		
**Item**	**Authors' judgement**	**Support for judgement**
Sequence generation	Yes	Random allocation: online random number generator
Allocation concealment?	Yes	Random allocation: online random number generator
Blinding of participants and personnel	Unclear	Blinding of personnel not described. Participants were not blinded to intervention.
Blinding of outcome assessment	Yes	No outcomes were assessed (counts of responses only).
Incomplete outcome data	Yes	Exclusions reported
Selective reporting	Yes	Outcomes reported in full
Other sources of bias	Yes	No grounds for concern about other sources of bias from reading the study report

**Table:** Edwards 2001

***Study characteristics***		
Methods	Random allocation: central randomisation	
Data	Head injured adults in the CRASH trial (UK)	
Comparisons	1. 1‐page questionnaire 2. 3‐page questionnaire	
Outcomes	Response within 3 months	
Topic	Health: disability after traumatic brain injury	
Mode of Administration	Postal	
Notes	—	
***Risk of bias***		
**Item**	**Authors' judgement**	**Support for judgement**
Allocation concealment?	Yes	A ‐ adequate

**Table:** Edwards 2009

***Study characteristics***		
Methods	Random allocation: computerised random‐number table	
Data	1587 American Indian adults living in the southwestern US aged 18 and over who completed a baseline study visit as part of Navajo EARTH cohort	
Comparisons	Long (18 pages) vs short (3 pages) follow‐up postal questionnaire	
Outcomes	Response after one month (2 reminders)	
Topic	Health (medical history and physical activity)	
Mode of Administration	Postal	
Notes	Each questionnaire included a one‐page medical history questionnaire asking the participant to update his or her health information. The short questionnaire also included the short format International Physical Activity Questionnaire making it 3 pages in length, while the long questionnaire included a 17‐page physical activity log developed for the Navajo EARTH Study physical activity validation study, making it 18 pages in length. Both follow‐up questionnaires were in English only. Cover letter and SAE were included with the questionnaire mailout packet, a postcard reminder was sent after the first mailing, a letter and second questionnaire were mailed to all non‐respondents approximately 1 month after the postcard was sent. The recommended mailing intervals were adjusted due to the potential delays in picking up mail. We included a $5 incentive with the first mailing and a sharpened pencil with each mailing.	
***Risk of bias***		
**Item**	**Authors' judgement**	**Support for judgement**
Sequence generation	Yes	Random allocation: computerised random‐number table
Allocation concealment?	Yes	Random allocation: computerised random‐number table
Blinding of participants and personnel	No	The study design did not allow for staff and participants to be blinded.
Blinding of outcome assessment	Yes	No outcomes were assessed (counts of responses only).
Incomplete outcome data	Yes	Exclusions reported
Selective reporting	Yes	Response after one month (2 reminders) reported in full
Other sources of bias	Yes	No grounds for concern about other sources of bias from reading the study report

**Table:** Edwards 2016a

***Study characteristics***		
Methods	Random allocation: method unspecified	
Data	Patients with depression and raised risk of cardiovascular disease	
Comparisons	Pre‐calling participants vs. no pre‐call	
Outcomes	Response after 3 reminders (6 weeks)	
Topic	Health (depression ‐ PHQ‐9)	
Mode of Administration	Postal/electronic	
Notes	Questionnaires included the same questions (e.g. depression (PHQ‐9), anxiety, treatment satisfaction, health service use), and took approximately 40 minutes to complete. A few days before the 4‐month follow‐up was due, participants were automatically sent the questionnaire in the same format (online or postal) that they had chosen to complete at baseline. For those who did not respond promptly, a standard procedure was followed in terms of sending out reminders for all follow‐ups. This involved: sending an email reminder; phoning the participant; posting a questionnaire (a second copy was posted for those who were completing the paper version); posting just the primary outcome questions (the PHQ‐9); finally, phoning participants to ask them to post back the PHQ‐9 or offering to complete it over the phone. After this final phone reminder was completed, no further attempts were made to collect data for that follow‐up time point.	
***Risk of bias***		
**Item**	**Authors' judgement**	**Support for judgement**
Sequence generation	Unclear	Random allocation: method unspecified
Allocation concealment?	Unclear	Random allocation: method unspecified
Blinding of participants and personnel	Unclear	Blinding of personnel not described. Participants were not blinded to intervention.
Blinding of outcome assessment	Yes	No outcomes were assessed (counts of responses only).
Incomplete outcome data	Yes	Exclusions reported
Selective reporting	Yes	Response after 3 reminders (6 weeks) reported in full
Other sources of bias	Yes	No grounds for concern about other sources of bias from reading the study report

**Table:** Edwards 2016b

***Study characteristics***		
Methods	Random allocation: method unspecified	
Data	Patients with depression and raised risk of cardiovascular disease	
Comparisons	Colour photograph of the research team vs. no photo	
Outcomes	Response after 3 reminders (6 weeks)	
Topic	Health (depression ‐ PHQ‐9)	
Mode of Administration	Postal/electronic	
Notes	Questionnaires included the same questions (e.g. depression (PHQ‐9), anxiety, treatment satisfaction, health service use), and took approximately 40 minutes to complete. A few days before the 4‐month follow‐up was due, participants were automatically sent the questionnaire in the same format (online or postal) that they had chosen to complete at baseline. For those who did not respond promptly, a standard procedure was followed in terms of sending out reminders for all follow‐ups. This involved: sending an email reminder; phoning the participant; posting a questionnaire (a second copy was posted for those who were completing the paper version); posting just the primary outcome questions (the PHQ‐9); finally, phoning participants to ask them to post back the PHQ‐9 or offering to complete it over the phone. After this final phone reminder was completed, no further attempts were made to collect data for that follow‐up time point.	
***Risk of bias***		
**Item**	**Authors' judgement**	**Support for judgement**
Sequence generation	Unclear	Random allocation: method unspecified
Allocation concealment?	Unclear	Random allocation: method unspecified
Blinding of participants and personnel	Unclear	Blinding of personnel not described. Participants were not blinded to intervention.
Blinding of outcome assessment	Yes	No outcomes were assessed (counts of responses only).
Incomplete outcome data	Yes	Exclusions reported
Selective reporting	Yes	Response after 3 reminders (6 weeks) reported in full
Other sources of bias	Yes	No grounds for concern about other sources of bias from reading the study report

**Table:** Edwards 2016c

***Study characteristics***		
Methods	Random allocation: method unspecified	
Data	Patients with depression and raised risk of cardiovascular disease	
Comparisons	'ACTION REQUIRED' subject in initial email reminder vs. 'Questionnaire reminder' in email subject	
Outcomes	Response after 3 reminders (6 weeks)	
Topic	Health (depression ‐ PHQ‐9)	
Mode of Administration	Postal/electronic	
Notes	Questionnaires included the same questions (e.g. depression (PHQ‐9), anxiety, treatment satisfaction, health service use), and took approximately 40 minutes to complete. A few days before the 4‐month follow‐up was due, participants were automatically sent the questionnaire in the same format (online or postal) that they had chosen to complete at baseline. For those who did not respond promptly, a standard procedure was followed in terms of sending out reminders for all follow‐ups. This involved: sending an email reminder; phoning the participant; posting a questionnaire (a second copy was posted for those who were completing the paper version); posting just the primary outcome questions (the PHQ‐9); finally, phoning participants to ask them to post back the PHQ‐9 or offering to complete it over the phone. After this final phone reminder was completed, no further attempts were made to collect data for that follow‐up time point.	
***Risk of bias***		
**Item**	**Authors' judgement**	**Support for judgement**
Sequence generation	Unclear	Random allocation: method unspecified
Allocation concealment?	Unclear	Random allocation: method unspecified
Blinding of participants and personnel	Unclear	Blinding of personnel not described. Participants were not blinded to intervention.
Blinding of outcome assessment	Yes	No outcomes were assessed (counts of responses only).
Incomplete outcome data	Yes	Exclusions reported
Selective reporting	Yes	Response after 3 reminders (6 weeks) reported in full
Other sources of bias	Yes	No grounds for concern about other sources of bias from reading the study report

**Table:** Elkind 1986

***Study characteristics***		
Methods	Random allocation: using alternation	
Data	Psychologists with APA membership	
Comparisons	1. Plain covering envelope with rubber‐stamped return address 2. University‐printed envelope 3. Postage‐stamped 4. Business reply	
Outcomes	1. Response rate at 6 weeks 2. Response rate after 12 weeks	
Topic	Health: patients' violence and harassment	
Mode of Administration	Postal: first‐class mail	
Notes	Method confirmed by the author; mainly males	
***Risk of bias***		
**Item**	**Authors' judgement**	**Support for judgement**
Allocation concealment?	No	C ‐ inadequate

**Table:** Enger 1993

***Study characteristics***		
Methods	Random allocation: method not specified	
Data	College graduates	
Comparisons	1. 2‐page questionnaire; stamped return envelope 2. 1‐page questionnaire; stamped return envelope 3. 1‐page questionnaire designed as a self‐mailer	
Outcomes	Response period not specified	
Topic	—	
Mode of Administration	Postal	
Notes	—	
***Risk of bias***		
**Item**	**Authors' judgement**	**Support for judgement**
Allocation concealment?	Unclear	B ‐ unclear

**Table:** Erdogan 2002

***Study characteristics***		
Methods	Random allocation: systematic assignment	
Data	Advertising agency managers who had not responded to a questionnaire mailed 2 weeks previously	
Comparisons	1. Original replacement follow‐up mailing: a colour department‐headed cover letter, original questionnaire plus self‐addressed, first‐class stamped return envelope 2. Photocopy replacement follow‐up mailing: a colour department‐headed cover letter, photocopied questionnaire plus self‐addressed, first‐class stamped return envelope 3. Post card: colour departmental follow‐up postcard only 4. Letter: Colour department‐headed follow‐up letter only	
Outcomes	Response period not specified	
Topic	Not specified	
Mode of Administration	Postal	
Notes	—	
***Risk of bias***		
**Item**	**Authors' judgement**	**Support for judgement**
Allocation concealment?	No	C ‐ inadequate

**Table:** Ernst 2018

***Study characteristics***		
Methods	Random allocation: using a random number algorithm in Microsoft Excel	
Data	Primary care gynaecologists, Germany	
Comparisons	Email with link to survey vs. postal letter with link to survey vs. mailed questionnaire	
Outcomes	Response after 6 weeks (reminder email and postal reminder with paper questionnaire)	
Topic	Health (case‐control study on care‐related factors associated with antepartal diagnosis of intrauterine growth restrictions)	
Mode of Administration	Electronic (Web survey)/postal	
Notes	Survey comprised Internet‐based or paper questionnaire; 21 items targeting gynaecologists' practice routine regarding intrauterine growth restriction (IUGR). Estimated 5‐10 minutes for completion Participants in the mixed‐mode (email/postal) group received an invitation to the Internet‐based survey via email (with URL). Non‐responders sent a reminder email with URL after 3 weeks and postal letter with a paper‐based questionnaire after 6 weeks. The mixed‐mode group (postal/email) received a postal invitation to Web survey, with written URL. Non‐responders sent reminder letter after 3 weeks, and postal letter with questionnaire after 6 weeks. The online survey design and layout was made to be as comparable to the postal version as possible. The questions were displayed in the same order and format. A professional web designer developed the study website that hosted the survey.	
***Risk of bias***		
**Item**	**Authors' judgement**	**Support for judgement**
Sequence generation	Yes	Random allocation: using a random number algorithm in Microsoft Excel
Allocation concealment?	Yes	Random allocation: using a random number algorithm in Microsoft Excel
Blinding of participants and personnel	Unclear	Blinding of personnel not described. Participants were not blinded to intervention.
Blinding of outcome assessment	Yes	No outcomes were assessed (counts of responses only).
Incomplete outcome data	Yes	Exclusions reported
Selective reporting	Yes	Outcomes reported in full
Other sources of bias	Yes	No grounds for concern about other sources of bias from reading the study report

**Table:** Etter 1996

***Study characteristics***		
Methods	Random allocation: computerised random number generation	
Data	French‐speaking patients at a medical practice in the suburbs of Geneva who had recently consulted a physician and who lived in Geneva at the time of data collection	
Comparisons	1. University letterhead; cover letter signed by the researchers; bsiness reply envelope addressed to the University of Geneva 2. Medical practice letterhead; cover letter signed by the director of the medical practice; business reply envelope addressed to the practice Packages sent to non‐respondents every 10 days up to a maximum of 4 times Reminder postcards sent 2 days after first and second mailings	
Outcomes	Response within 45 days	
Topic	Health: patient satisfaction	
Mode of Administration	Postal	
Notes	Method of allocation ascertained through contact with author	
***Risk of bias***		
**Item**	**Authors' judgement**	**Support for judgement**
Allocation concealment?	Yes	A ‐ adequate

**Table:** Etter 1998a

***Study characteristics***		
Methods	Random allocation: computerised random number generation	
Data	Students, faculty, administrative and technical staff of a university (Geneva, Switzerland)	
Comparisons	1. Sent saliva vial; offered participation in lottery; pen incentive 2. Saliva vial; pen incentive 3. Saliva vial; offered participation in lottery 4. Saliva vial 5. Offered participation in lottery; pen incentive 6. Pen incentive 7. Offered participation in lottery 8. None Best response intervention was sent as follow‐up.	
Outcomes	—	
Topic	Health: health status using SF‐36, smoking habits, self‐efficacy	
Mode of Administration	Postal	
Notes	Method of allocation ascertained through contact with author; mean age: 28.5 years; mainly females	
***Risk of bias***		
**Item**	**Authors' judgement**	**Support for judgement**
Allocation concealment?	Yes	A ‐ adequate

**Table:** Etter 1998b

***Study characteristics***		
Methods	Random allocation: computerised random number generation	
Data	Residents of Geneva	
Comparisons	1. Professional layout; prior feedback letter 2. Professional layout; no prior feedback letter 3. Standard layout; prior feedback letter 4. Standard layout; prior feedback letter	
Outcomes	—	
Topic	Health: use of health services, satisfaction with medical care	
Mode of Administration	Postal	
Notes	Method of allocation ascertained through contact with author; mean age: 32 years; equal male and females	
***Risk of bias***		
**Item**	**Authors' judgement**	**Support for judgement**
Allocation concealment?	Yes	A ‐ adequate

**Table:** Etter 2002

***Study characteristics***		
Methods	Random allocation: method not specified	
Data	Members of various health insurance plans aged 19‐45 (Geneva, Switzerland)	
Comparisons	1. Light green paper questionnaire 2. White paper questionnaire 5 follow‐up reminder questionnaires were used.	
Outcomes	Response within 50 days	
Topic	Health: health status, health related lifestyles, use of medical services, satisfaction with medical care, sociodemographics	
Mode of Administration	Postal	
Notes	Age: 19‐45 years	
***Risk of bias***		
**Item**	**Authors' judgement**	**Support for judgement**
Allocation concealment?	Unclear	B ‐ unclear

**Table:** Etzel 1974

***Study characteristics***		
Methods	Random allocation: method not specified	
Data	Random sample of bank credit card holders on a list provided by a bank, USA	
Comparisons	1. No follow‐up 2. Follow‐up without duplicate questionnaire and return envelope sent 5 days after initial mailing 3. Follow‐up with duplicates sent 5 days after initial mailing	
Outcomes	Response within 17 days	
Topic	Non‐health: finance ‐ credit care usage	
Mode of Administration	Postal	
Notes	Method of allocation ascertained through contact with author	
***Risk of bias***		
**Item**	**Authors' judgement**	**Support for judgement**
Allocation concealment?	Unclear	B ‐ unclear

**Table:** Evans 2004

***Study characteristics***		
Methods	Random allocation: method not specified	
Data	Men diagnosed with prostate cancer	
Comparisons	1. Unconditional 30 minutes prepaid phone card 2. Conditional 30 minutes prepaid phone card	
Outcomes	Response period not specified	
Topic	Health: Dietary supplementation use in cancer patients	
Mode of Administration	Postal	
Notes	Mainly males	
***Risk of bias***		
**Item**	**Authors' judgement**	**Support for judgement**
Sequence generation	Unclear	Random allocation: method not specified
Allocation concealment?	Unclear	Random allocation: method not specified
Blinding of participants and personnel	Unclear	Blinding of personnel not described. Participants were not blinded to intervention.
Blinding of outcome assessment	Yes	No outcome assessment used (counts of responses only)

**Table:** Falthzik 1971

***Study characteristics***		
Methods	Random allocation: odd numbered firms received closed question; even numbered firms received open‐ended question	
Data	Personnel departments of 200 firms listed in Fortune magazine's list of the 500 largest firms in the US	
Comparisons	1. Closed question 2. Open‐ended question	
Outcomes	Response period not specified	
Topic	Non‐health: characteristics while hiring college graduates	
Mode of Administration	Postal	
Notes	Random allocation unclear	
***Risk of bias***		
**Item**	**Authors' judgement**	**Support for judgement**
Allocation concealment?	No	C ‐ inadequate

**Table:** Faria 1990

***Study characteristics***		
Methods	Random allocation: alternation	
Data	Homeowners residing in a medium‐sized US city on the 'city property owners' listing	
Comparisons	1. Telephone pre‐contact 1‐3 days before questionnaire mailing 2. Letter pre‐notification sent 2 days before questionnaire mailing 3. No pre‐contact	
Outcomes	Response period not specified	
Topic	Not specified	
Mode of Administration	Postal	
Notes	—	
***Risk of bias***		
**Item**	**Authors' judgement**	**Support for judgement**
Allocation concealment?	No	C ‐ inadequate

**Table:** Faria 1992

***Study characteristics***		
Methods	Random allocation: alternation	
Data	Individuals listed on the company mailing list of a major manufacturer	
Comparisons	1. University sponsor; no promised contribution to charity 2. University sponsor; promised contribution to a specified charity 3. University sponsor; promised contribution to 1 of 3 charities selected by respondent 4. Commercial sponsor; no promised contribution to charity 5. Commercial sponsor; promised contribution to charity 6. Commercial sponsor; promised contribution to 1 of 3 charities selected by respondent	
Outcomes	Response within 23 days	
Topic	Not specified	
Mode of Administration	Postal	
Notes	Method of allocation ascertained through contact with author	
***Risk of bias***		
**Item**	**Authors' judgement**	**Support for judgement**
Allocation concealment?	Yes	A ‐ adequate

**Table:** Faria 1997

***Study characteristics***		
Methods	Random allocation: method not specified	
Data	Patients with acute stroke	
Comparisons	1. Questionnaire with stamped return envelope 2. Questionnaire with free post‐return envelope	
Outcomes	—	
Topic	Health	
Mode of Administration	Postal	
Notes	—	
***Risk of bias***		
**Item**	**Authors' judgement**	**Support for judgement**
Allocation concealment?	Unclear	B ‐ unclear

**Table:** Farley 2014

***Study characteristics***		
Methods	Random allocation: RAND function in Excel	
Data	Healthcare professionals involved in the care and management of patients with a psychosis diagnosis	
Comparisons	Shorter vs. longer questionnaire	
Outcomes	Response after 5 weeks (2 reminders)	
Topic	Health (identifying opinion leaders for behaviour‐change interventions in healthcare)	
Mode of Administration	Postal or electronic (choice for all)	
Notes	Longer ‐ 22 questions; shorter ‐ 20 questions Participants were given a choice of paper and online (accessed via a hyperlink in an email) versions of both questionnaires. Individualised pre‐notification letters were sent. A reminder email and link to the online questionnaire was sent two weeks later to those who had not responded. Three weeks later, a paper‐based reminder with a paper copy of the questionnaire was sent to all remaining non‐responders. The questionnaires contained different Opinion Leader identification questions, and as such these differences may have confounded the results.	
***Risk of bias***		
**Item**	**Authors' judgement**	**Support for judgement**
Sequence generation	Yes	Random allocation: RAND function in Excel
Allocation concealment?	Yes	Random allocation: RAND function in Excel
Blinding of participants and personnel	Unclear	Blinding of personnel not described. Participants were not blinded to intervention.
Blinding of outcome assessment	Yes	No outcomes were assessed (counts of responses only).
Incomplete outcome data	Yes	75 questionnaires were returned as staff had left the NHS Trust.
Selective reporting	Yes	Response after 5 weeks (2 reminders) reported in full
Other sources of bias	No	The questionnaires contained different Opinion Leader identification questions and, as such, these differences may have confounded the results.

**Table:** Farmer 2005

***Study characteristics***		
Methods	Random allocation: method unspecified	
Data	People recruited to a programme intended to characterise people with a family history of type 2 diabetes	
Comparisons	Limited follow‐up (single questionnaire at 1 year) vs. intensive follow‐up (different questionnaires at 1, 6 and 12 months)	
Outcomes	Response to a 1‐year follow‐up questionnaire	
Topic	Health (well‐being and anxiety questionnaires with diabetes screening)	
Mode of Administration	Postal	
Notes	Limited questionnaire follow‐up: participants received no additional questionnaires, first contact after the letter giving test results was 1‐year questionnaire. Intensive questionnaire follow‐up: following attendance for screening, further questionnaires were sent with the result letter at 1 month, 6 months, and 1 year of follow‐up. The questionnaire sent with the results letter was three pages long and asked about satisfaction with the procedures for testing encountered. A subsequent questionnaire at 6 months was two pages long in length and consisted of the WBQ‐12, the SSAI‐SF, and two questions asking whether the participants thought they might have, or be at risk of developing, diabetes.	
***Risk of bias***		
**Item**	**Authors' judgement**	**Support for judgement**
Sequence generation	Unclear	Random allocation: method unspecified
Allocation concealment?	Unclear	Random allocation: method unspecified
Blinding of participants and personnel	Unclear	Blinding of personnel not described. Participants were not blinded to intervention.
Blinding of outcome assessment	Yes	No outcomes were assessed (counts of responses only).
Incomplete outcome data	Yes	Exclusions were reported.
Selective reporting	Yes	Response to a 1‐year follow‐up questionnaire reported in full
Other sources of bias	Yes	No grounds for concern about other sources of bias from reading the study report

**Table:** Feigelson 2017

***Study characteristics***		
Methods	Random allocation: method unspecified	
Data	4000 colorectal cancer (CRC) survivors	
Comparisons	Email link to web survey with mail follow‐up vs. email link to web survey with interactive voice response (IVR) follow‐up.	
Outcomes	Response after one follow‐up	
Topic	Health (colorectal cancer)	
Mode of Administration	Postal/electronic	
Notes	17‐questions. All participants who completed the survey received a $10 gift card in the mail. Participants were informed of this compensation when they were first contacted and asked to participate in the study. Participants received up to 2 email letters of invitation (11 days apart) which included a link to a secure website to complete the survey. Non‐responders in the mail follow‐up arm received a mailed survey, and non‐responders in the interactive voice response (IVR) arm received the survey as an IVR call.	
***Risk of bias***		
**Item**	**Authors' judgement**	**Support for judgement**
Sequence generation	Unclear	Random allocation: method unspecified
Allocation concealment?	Unclear	Random allocation: method unspecified
Blinding of participants and personnel	Unclear	Blinding of personnel not described. Participants were not blinded to intervention.
Blinding of outcome assessment	Yes	No outcomes were assessed (counts of responses only).
Incomplete outcome data	No	Exclusions not reported
Selective reporting	Yes	Response after one follow‐up reported in full
Other sources of bias	Yes	No grounds for concern about other sources of bias from reading the study report

**Table:** Feild 1975

***Study characteristics***		
Methods	Random allocation: method not specified	
Data	Full‐time teaching faculty members of a large southeastern university (US)	
Comparisons	1. Signed by male investigator; sent to male subject 2. Signed by male investigator; sent to female subject 3. Signed by female investigator; sent to male subject 4. Signed by female investigator; sent to female subject 5. Signed by both male and female investigators; sent to male subject 6. Signed by both male and female investigators; sent to female subject	
Outcomes	Response period not specified	
Topic	Non‐health: short form of the attitudes towards women scale	
Mode of Administration	Postal	
Notes	Equal male and females	
***Risk of bias***		
**Item**	**Authors' judgement**	**Support for judgement**
Allocation concealment?	Unclear	B ‐ unclear

**Table:** Felix 2011

***Study characteristics***		
Methods	Random allocation: computer‐generated using the RANDBETWEEN command in Microsoft Excel	
Data	Authors who had published research on maternal health	
Comparisons	Pre‐notification email vs. no pre‐notification Pleading email invitation vs. non‐pleading email invitation	
Outcomes	e‐completion and e‐submission after 2 reminders over 5 weeks	
Topic	Health (perceptions of the generalisability of maternal health research from one setting to another in low‐income countries.)	
Mode of Administration	Electronic (web survey)	
Notes	23 questions (5 open‐ended). During piloting, the survey took about 10 minutes to complete. The email invitation contained a hyperlink to the Web‐based survey. Participants were required to answer questions by clicking points on a Likert scale or entering free text for open‐ended questions. On completion of the survey, participants were asked to click a submit button. The questionnaire was initially piloted by work colleagues of one author. Email reminders were sent to all non‐responders at weeks 2 and 5.	
***Risk of bias***		
**Item**	**Authors' judgement**	**Support for judgement**
Sequence generation	Yes	Random allocation: computer‐generated using the RANDBETWEEN() command in Microsoft Excel
Allocation concealment?	Yes	Random allocation: computer‐generated using the RANDBETWEEN() command in Microsoft Excel
Blinding of participants and personnel	Unclear	Blinding of personnel not described. Participants were not blinded to intervention.
Blinding of outcome assessment	Yes	No outcome assessment used (counts of responses only)
Incomplete outcome data	Yes	Exclusions reported
Selective reporting	Yes	Outcomes reported
Other sources of bias	Yes	No grounds for concern about other sources of bias from reading the study report

**Table:** Ferrell 1984

***Study characteristics***		
Methods	Random allocation: method not specified	
Data	Practitioners in managerial or administrative capacities listed in the American Marketing Association roster	
Comparisons	1. Questionnaire sent to home address 2. Questionnaire sent to work address	
Outcomes	Response period not specified	
Topic	Non‐health: marketing terms used by organisations, description of jobs, and their firm	
Mode of Administration	Postal	
Notes	—	
***Risk of bias***		
**Item**	**Authors' judgement**	**Support for judgement**
Allocation concealment?	Unclear	B ‐ unclear

**Table:** Finn 1983

***Study characteristics***		
Methods	Random allocation: method not specified	
Data	Heads of households in the trading area of certain financial institutions. The target area was a middle to high‐income section of the city and included all age groups and family sizes.	
Comparisons	1. Return envelope with standard first class stamp and typed return address 2. Pre‐printed business reply envelope	
Outcomes	Response within 29 days	
Topic	Non‐health: finance ‐ usage of financial institutions, attitudes about local banks, savings and loan associations	
Mode of Administration	Postal	
Notes	—	
***Risk of bias***		
**Item**	**Authors' judgement**	**Support for judgement**
Allocation concealment?	Unclear	B ‐ unclear

**Table:** Finsen 2006

***Study characteristics***		
Methods	Random allocation: alphabetical order	
Data	Norwegian residents aged between 40 and 65 years	
Comparisons	1. Unconditional; 1 scratch lottery worth 20 Norwegian Kroner (NOK) 2. Unconditional; 2 scratch lotteries each worth 20 Norwegian Kroner 3. Conditional; 2 scratch lotteries each worth 20 Norwegian Kroner on reply within one week 4. Unconditional; 50 NOK 5. Control	
Outcomes	Response rate at 6 weeks	
Topic	Health: history of surgery	
Mode of Administration	Postal	
Notes	Age: 51.4 years; mainly males	
***Risk of bias***		
**Item**	**Authors' judgement**	**Support for judgement**
Allocation concealment?	No	C ‐ inadequate

**Table:** Fiset 1994

***Study characteristics***		
Methods	Random allocation: alternation	
Data	Dentists insured by a major malpractice carrier in 2 western states (US)	
Comparisons	Experiment 1: 1. $5 incentive 2. $10 incentive Follow‐up with postcard after 1 week Experiment 2: 1. $5 incentive 2. $10 incentive Follow‐up with postcard after 1 week Questionnaire package sent to non‐responders again at 3 and 7 weeks	
Outcomes	Response period not specified	
Topic	Health: Dentist relationship of dental malpractice claims to decisions about clinical practice	
Mode of Administration	Postal	
Notes	Mean age: 37‐41.4 years; mainly females	
***Risk of bias***		
**Item**	**Authors' judgement**	**Support for judgement**
Allocation concealment?	No	C ‐ inadequate

**Table:** Fluss 2014

***Study characteristics***		
Methods	Random allocation: electronic randomisation programme	
Data	4600 residents in Grampian (north of Scotland, UK) aged 25 years and over	
Comparisons	Single‐sided vs. double‐sided questionnaire Electronic (Web) response option vs. mail response only	
Outcomes	Response after 2 weeks (1 reminder)	
Topic	Health (pain and pain management)	
Mode of Administration	Postal and electronic	
Notes	20‐page study questionnaire included questions on demographic characteristics (gender, age, and educational background), health (SF‐36), pain and pain management (and chronic pain grade). Either received a 10‐sheet (double‐sided) or a 20‐sheet (single‐sided) questionnaire. Selected persons were sent a notification letter that they had been selected for the study. One week later they were sent a survey pack comprising an invitation letter, an information sheet, the questionnaire and an SAE. Potential participants were advised to read the information sheet and to complete and return the questionnaire if they wished to take part. The invitation letter for those in the web option groups contained, in addition, the URL link to the electronic questionnaire and their individual ID number and password for its access. Non‐respondents were sent a second survey pack appropriate to their randomisation group, 3 weeks after the first contact (i.e. two weeks after the questionnaire distribution).	
***Risk of bias***		
**Item**	**Authors' judgement**	**Support for judgement**
Sequence generation	Yes	Random allocation: electronic randomisation programme
Allocation concealment?	Yes	Random allocation: electronic randomisation programme
Blinding of participants and personnel	Unclear	Blinding of personnel not described. Participants were not blinded to intervention.
Blinding of outcome assessment	Yes	No outcomes were assessed (counts of responses only).
Incomplete outcome data	Yes	183 questionnaires were found invalid (for example, due to an invited participant being deceased or no longer resident at the given address).
Selective reporting	Yes	Response after 2 weeks (1 reminder) reported in full
Other sources of bias	Yes	No grounds for concern about other sources of bias from reading the study report

**Table:** Ford 1967a

***Study characteristics***		
Methods	Random allocation: method not specified	
Data	Residents of Chenoa	
Comparisons	1. Advance letter 2. No advance letter	
Outcomes	Response within 30 days	
Topic	Non‐health: consumer shopping survey	
Mode of Administration	Postal	
Notes	Method of allocation ascertained through contact with author	
***Risk of bias***		
**Item**	**Authors' judgement**	**Support for judgement**
Allocation concealment?	Unclear	B ‐ unclear

**Table:** Ford 1967b

***Study characteristics***		
Methods	Random allocation: method not specified	
Data	Residents of Beardstown	
Comparisons	1. Advance letter 2. No advance letter	
Outcomes	Response within 30 days	
Topic	Non‐health: consumer shopping survey	
Mode of Administration	Postal	
Notes	Method of allocation ascertained through contact with author	
***Risk of bias***		
**Item**	**Authors' judgement**	**Support for judgement**
Allocation concealment?	Unclear	B ‐ unclear

**Table:** Ford 1968

***Study characteristics***		
Methods	Random allocation: method not specified	
Data	Households	
Comparisons	1. Questionnaire consisted of 1 sheet, printed on both sides which, when folded, had four 8.5 x 11" pages of questions. 2. Questionnaire mimeographed on 1 side only and stapled, so had four pages of 8.5 x 14". All participants were sent an advance letter 12 days before the questionnaire was sent.	
Outcomes	Response within 23 days	
Topic	Non‐health: consumer shopping survey	
Mode of Administration	Postal	
Notes	—	
***Risk of bias***		
**Item**	**Authors' judgement**	**Support for judgement**
Allocation concealment?	Unclear	B ‐ unclear

**Table:** Foushee 1990

***Study characteristics***		
Methods	Random allocation: using random number generation in SAS	
Data	Tour operators in Europe, South America, and Japan	
Comparisons	1. Early follow‐up with postcard (3 weeks) 2. Late follow up with postcard (6 weeks)	
Outcomes	Response period not specified	
Topic	Non‐health: potential for attracting and accommodating foreign visitors to national park	
Mode of Administration	Postal	
Notes	Method of allocation ascertained through contact with author	
***Risk of bias***		
**Item**	**Authors' judgement**	**Support for judgement**
Allocation concealment?	Yes	A ‐ adequate

**Table:** Fowler 2019

***Study characteristics***		
Methods	Random allocation: method unspecified	
Data	Patients age 18+ who had at least one primary care visit during the previous 6 months, Greater Boston, US	
Comparisons	Electronic vs. postal; electronic only vs. mixed‐mode reminder (push to web); postal only vs. mixed‐mode reminder (push to web)	
Outcomes	Response after reminders	
Topic	Health (medical care experiences)	
Mode of Administration	Electronic and mixed mode	
Notes	56 questions (standard Clinician and Group CAHPS survey); the paper questionnaire was 12 pages, including a cover page and unused back page. The layout of the Internet version was as close as possible to the paper version. Email/letter, one week follow‐up for non‐respondents and further reminder after an additional week. A. Internet: link through portal: patients were sent an email telling them that they had a new message in their portal, link to survey in the message. Second email was sent to everyone a week later. B. Internet: link without using portal. Patients were sent a personalised email that was similar to the letter patients received through their portal with link to survey. Non‐respondents sent a reminder email 1 week after the initial email and a second sent 1 week later. C. Sequential web‐mail protocol, with Internet link followed by mail to non‐respondents. Mail pre‐notification of email survey, then as protocol B. With final paper questionnaire by mail with cover letter and SAE. D. Mail only questionnaire with cover letter, and SAE, reminder postcards after 2 weeks. A further 2 weeks, non‐respondents sent 2nd questionnaire	
***Risk of bias***		
**Item**	**Authors' judgement**	**Support for judgement**
Sequence generation	Unclear	Random allocation: method unspecified
Allocation concealment?	Unclear	Random allocation: method unspecified
Blinding of participants and personnel	Unclear	Blinding of personnel not described. Participants were not blinded to intervention.
Blinding of outcome assessment	Yes	No outcomes were assessed (counts of responses only).
Incomplete outcome data	No	Exclusions not reported
Selective reporting	Yes	Response after reminders reported in full
Other sources of bias	Yes	No grounds for concern about other sources of bias from reading the study report

**Table:** Frederiks 2020

***Study characteristics***		
Methods	Random allocation: random integer generator	
Data	Randomly‐selected households in the state of Victoria, Australia	
Comparisons	Message printed in blue on the front of the envelope vs. no message Unconditional non‐monetary incentive (magnet with science agency logo) vs. conditional prize draw to win one of five $200 retail gift cards vs. no incentive Post‐it note with handwritten "thank you" vs. no post‐it Reminder postcard vs. no reminder	
Outcomes	Response period not specified	
Topic	Non‐health (household's energy consumption)	
Mode of Administration	Postal or electronic	
Notes	Single A3 page (folded in half to form a booklet) with a one‐page information sheet and 3 pages of questions. Information sheet for some versions of the survey referred to a prize draw (8 versions) or token fridge magnet (8 versions). Participants could complete the survey either by filling out and returning the paper form (using the reply‐paid envelope) or answering the questions online (using a survey URL printed on the form). The information sheet also included 'survey access code' unique to each of the 24 versions. This code was used by participants to access the online survey, as well as allowing the research team to determine which version of the survey each participant received. Envelope message "stating ‘Help create Australia's energy future’"	
***Risk of bias***		
**Item**	**Authors' judgement**	**Support for judgement**
Sequence generation	Yes	Random allocation: random integer generator
Allocation concealment?	Yes	Random allocation: random integer generator
Blinding of participants and personnel	Unclear	Blinding of personnel not described. Participants were not blinded to intervention.
Blinding of outcome assessment	Yes	No outcomes were assessed (counts of responses only).
Incomplete outcome data	Yes	Exclusions reported
Selective reporting	Yes	Responses reported in full
Other sources of bias	Yes	No grounds for concern about other sources of bias from reading the study report

**Table:** Fredrickson 2005

***Study characteristics***		
Methods	Random allocation: SAS programme	
Data	Participants in a Kansas Medicaid managed care plan, US	
Comparisons	Group 1 ‐ Standard mailing vs. group 2 ‐ User‐friendly (standard mailing plus Spanish‐language option and low‐literacy correspondence) vs. Group 3 ‐ User‐friendly mailing plus conditional non‐monetary incentive ($10 voucher)	
Outcomes	Response after 2 reminders (3 weeks)	
Topic	Health (satisfaction with healthcare plans)	
Mode of Administration	Postal	
Notes	Questionnaire not described. Mailings were sent to arrive on Friday or Saturday when respondents may have time to complete the questionnaire. A pre‐notification letter was followed 2 weeks later by the questionnaire with a cover letter and an SAE. A reminder postcard was sent 1 week later. Two weeks after the reminder postcard the questionnaire was sent again.	
***Risk of bias***		
**Item**	**Authors' judgement**	**Support for judgement**
Sequence generation	Yes	Random allocation: SAS programme
Allocation concealment?	Yes	Random allocation: SAS programme
Blinding of participants and personnel	Unclear	Blinding of personnel not described. Participants were not blinded to intervention.
Blinding of outcome assessment	Yes	No outcomes were assessed (counts of responses only).
Incomplete outcome data	Yes	Exclusions were reported.
Selective reporting	Yes	Response after 2 reminders (3 weeks) reported in full
Other sources of bias	Yes	No grounds for concern about other sources of bias from reading the study report

**Table:** Freise 2001

***Study characteristics***		
Methods	Random allocation: using dice	
Data	Last 1400 discharged patients of the University hospital of Cologne on 14/02/2000	
Comparisons	1. 12‐page questionnaire 2. 8‐page questionnaire 3. 4‐page questionnaire	
Outcomes	Response period not specified	
Topic	Health: Cologne patient questionnaire	
Mode of Administration	Postal	
Notes	Age: above 18 years	
***Risk of bias***		
**Item**	**Authors' judgement**	**Support for judgement**
Allocation concealment?	No	C ‐ inadequate

**Table:** Friedman 1975

***Study characteristics***		
Methods	Random allocation: method not specified	
Data	Travel agents who subscribed to a travel magazine	
Comparisons	1. Author had 'Hispanic' name 2. Author had 'Jewish' name 3. Ethnicity of author not identifiable	
Outcomes	Response period not specified	
Topic	Non‐health: ethnic identification	
Mode of Administration	Postal	
Notes	—	
***Risk of bias***		
**Item**	**Authors' judgement**	**Support for judgement**
Allocation concealment?	Unclear	B ‐ unclear

**Table:** Friedman 1979

***Study characteristics***		
Methods	Random allocation: method not specified	
Data	Individuals listed in telephone directories of 2 suburban areas in the greater New York Metropolitan area	
Comparisons	1. Black sponsor signature; 25 cents incentive 2. Black sponsor signature; no incentive 3. White sponsor signature; 25 cents incentive 4. White sponsor signature; no incentive	
Outcomes	Response within 2 weeks	
Topic	Non‐health: attitudes towards the Negroes Scale	
Mode of Administration	Postal	
Notes	—	
***Risk of bias***		
**Item**	**Authors' judgement**	**Support for judgement**
Allocation concealment?	Unclear	B ‐ unclear

**Table:** Furse 1982

***Study characteristics***		
Methods	Random allocation: method not specified	
Data	Microwave oven owners listed in a major manufacturer's warranty registration records	
Comparisons	1. No personal or charity incentive offered 2. Charity incentive (promise of $1 to charity of respondent's choice for returned questionnaire) 3. 50 cents enclosed with questionnaire 4. $1 enclosed with questionnaire 5. 50 cents enclosed with questionnaire and charity incentive (promise of $1 to charity of respondent's choice for returned questionnaire) 6. $1 enclosed with questionnaire and charity incentive (promise of $1 to charity of respondent's choice for returned questionnaire)	
Outcomes	Response period not specified	
Topic	Not specified	
Mode of Administration	Postal	
Notes	—	
***Risk of bias***		
**Item**	**Authors' judgement**	**Support for judgement**
Allocation concealment?	Unclear	B ‐ unclear

**Table:** Furst 1979

***Study characteristics***		
Methods	Random allocation: computerised random number generation	
Data	Secretaries of school principals	
Comparisons	1. Pre‐notification 2. No pre‐notification	
Outcomes	Response period not specified	
Topic	Health: Eysenck Personality Inventory (EPI)	
Mode of Administration	Postal	
Notes	Method of allocation confirmed through contact with author. Informed that allocation concealment was poor	
***Risk of bias***		
**Item**	**Authors' judgement**	**Support for judgement**
Allocation concealment?	No	C ‐ inadequate

**Table:** Futrell 1977

***Study characteristics***		
Methods	Random allocation: method not specified	
Data	Salesmen from a national hospital supply company	
Comparisons	1. Instruction to return the questionnaire unsigned 2. Asked to sign the questionnaire 2 follow‐up letters sent 10 days apart 2 weeks after questionnaire sent	
Outcomes	Response period not specified	
Topic	Non‐health: salesman's attitudes towards their job, evaluation of job performance by supervisors	
Mode of Administration	Postal	
Notes	—	
***Risk of bias***		
**Item**	**Authors' judgement**	**Support for judgement**
Allocation concealment?	Unclear	B ‐ unclear

**Table:** Futrell 1978

***Study characteristics***		
Methods	Random allocation: method not specified	
Data	Grocery store managers	
Comparisons	1. Allowed to remain anonymous 2. Required to sign questionnaire	
Outcomes	Response period not specified	
Topic	Non‐health: job attitudes	
Mode of Administration	Postal	
Notes	—	
***Risk of bias***		
**Item**	**Authors' judgement**	**Support for judgement**
Allocation concealment?	Unclear	B ‐ unclear

**Table:** Futrell 1981

***Study characteristics***		
Methods	Random allocation: method not specified	
Data	Farmers and ranchers	
Comparisons	1. Questionnaire and letter 2. Questionnaire and letter; non‐respondents after 2 weeks sent letter and questionnaire 3. Questionnaire and letter; non‐respondents after 2 weeks sent letter only 4. Questionnaire and letter; non‐respondents after 2 and 4 weeks sent letter and questionnaire 5. Questionnaire and letter; non‐respondents after 2 and 4 weeks sent letter only 6. Questionnaire and letter; non‐respondents after 2, 4 and 6 weeks sent letter and questionnaire 7. Questionnaire and letter; non‐respondents after 2, 4 and 6 weeks sent letter only	
Outcomes	—	
Topic	Non‐health: finance ‐ perceptions of agricultural producers regarding financial lending institutions	
Mode of Administration	Postal	
Notes	—	
***Risk of bias***		
**Item**	**Authors' judgement**	**Support for judgement**
Allocation concealment?	Unclear	B ‐ unclear

**Table:** Futrell 1982

***Study characteristics***		
Methods	Random allocation: method not specified	
Data	Industrial accountants	
Comparisons	1. No statement assuring anonymity; asked to return questionnaire the same day it was received 2. No statement assuring anonymity; asked to return questionnaire at their leisure 3. Statement assuring anonymity; asked to return questionnaire the same day it was received 4. Statement assuring anonymity; asked to return questionnaire at their leisure	
Outcomes	Response period not specified	
Topic	Non‐health: role conflict, role clarity, job tension, job satisfaction	
Mode of Administration	Postal	
Notes	—	
***Risk of bias***		
**Item**	**Authors' judgement**	**Support for judgement**
Allocation concealment?	Unclear	B ‐ unclear

**Table:** Gajic 2012

***Study characteristics***		
Methods	Random allocation: method unspecified	
Data	Random sample of 3000 Ontario residents	
Comparisons	No incentive vs. unconditional monetary incentive ($2) vs. lower conditional non‐monetary incentive (lottery: 10 prizes of $25) vs. higher conditional non‐monetary incentive (lottery: 2 prizes of $250)	
Outcomes	Response after 3 weeks (1 reminder)	
Topic	Health (presence and magnitude of health‐related externalities)	
Mode of Administration	Postal invitation to web survey	
Notes	The survey comprised four main parts, > 60 questions; 1) willingness to contribute money to a charity that provided financial assistance so recipients could obtain desired healthcare (12 questions); 2) subjects willingness to pay for their own treatment (16 questions); 3) instrument developed by social psychologists to measure an individual's social values orientation (24 questions); 4) socioeconomic and demographic questions The letter of invitation explained the research topic, invited respondents to participate in the study (with a response deadline) and provided both an individual ID code and a simple McMaster University web address to which to respond. Potential respondents were invited to complete the survey online. A second, follow‐up letter of invitation was mailed 3 weeks after the initial letter. Returned non‐deliverable letters were logged in a central database following the initial mailing. All invitation letters were identical except for minor wording differences associated with each incentive.	
***Risk of bias***		
**Item**	**Authors' judgement**	**Support for judgement**
Sequence generation	Unclear	Random allocation: method unspecified
Allocation concealment?	Unclear	Random allocation: method unspecified
Blinding of participants and personnel	Unclear	Blinding of personnel not described. Participants were not blinded to intervention.
Blinding of outcome assessment	Yes	No outcomes were assessed (counts of responses only).
Incomplete outcome data	Yes	Exclusions were reported.
Selective reporting	Yes	Response after 3 weeks (1 reminder) reported in full
Other sources of bias	Yes	No grounds for concern about other sources of bias from reading the study report

**Table:** Gajraj 1990

***Study characteristics***		
Methods	Random allocation: alternation	
Data	Customers of a major public utility, comprising households in southwestern Ontario	
Comparisons	1. No incentive 2. $0.50 included 3. Promise of $0.50 on return of completed questionnaire 4. Pen included 5. Promise of pen on return of completed questionnaire 6. Inclusion in share of winning from 5 Super Lotto lottery tickets 7. Promise of inclusion in share of 5 Super Lotto lottery tickets on return of completed questionnaire All sent same questionnaire, mailing envelope, computer‐printed label and return envelope. Cover letters varied only in stating the amount of incentives.	
Outcomes	Response within 25 days	
Topic	Non‐health: general area of energy conservation	
Mode of Administration	Postal	
Notes	Method of allocation confirmed through contact with author. Informed that allocation concealment was adequate	
***Risk of bias***		
**Item**	**Authors' judgement**	**Support for judgement**
Allocation concealment?	Yes	A ‐ adequate

**Table:** Galesic 2009

***Study characteristics***		
Methods	Random allocation: method unspecified	
Data	3472 participants accessed survey through banners on 2 major job‐search websites	
Comparisons	Respondents were told: "the survey lasts 10 minutes" vs. "20 minutes" vs. "30 minutes".	
Outcomes	Started questionnaire; completed questionnaire	
Topic	Health (quality of life, subjective health, attitudes, and behaviours of unemployed people in Croatia)	
Mode of Administration	Web survey	
Notes	The questionnaire, programmed in WARP‐IT software (RM Plus 2003), consisted of 10 screening questions on demographics, followed by about 180 questions divided into 20 blocks. Each question block occupied approximately one screen and contained questions on a related topic, e.g. on everyday activities or the respondents' financial situation. The study was conducted within a web survey on quality of life, subjective health, attitudes, and behaviours of unemployed people in Croatia. The survey was advertised via banners briefly describing the survey topic on two major job‐search sites. The respondents were volunteers who visited the sites and clicked on the banner. Respondents were randomly assigned to three different conditions regarding the announced length.	
***Risk of bias***		
**Item**	**Authors' judgement**	**Support for judgement**
Sequence generation	Unclear	Random allocation: method unspecified
Allocation concealment?	Unclear	Random allocation: method unspecified
Blinding of participants and personnel	Unclear	Blinding of personnel not described. Participants were not blinded to intervention.
Blinding of outcome assessment	Yes	No outcomes were assessed (counts of responses only).
Incomplete outcome data	No	Exclusions not reported
Selective reporting	Yes	Numbers of participants who started and completed questionnaire reported in full
Other sources of bias	Yes	No grounds for concern about other sources of bias from reading the study report

**Table:** Garcia 2014

***Study characteristics***		
Methods	Random allocation: method unspecified	
Data	Rheumatoid arthritis patients, Southern California, US	
Comparisons	Mail with follow‐up letter vs. mail with follow‐up telephone call	
Outcomes	Response within one month (with one reminder after 2 weeks)	
Topic	Health (Health Assessment Questionnaire disability index and pain scale, EuroQol 5)	
Mode of Administration	Postal	
Notes	7‐page survey. Patients were given the option to decline participation, and those who received mail surveys were also sent an "opt‐out" postcard should they choose not to participate.	
***Risk of bias***		
**Item**	**Authors' judgement**	**Support for judgement**
Sequence generation	Unclear	Random allocation: method unspecified
Allocation concealment?	Unclear	Random allocation: method unspecified
Blinding of participants and personnel	No	Participants and personnel were not blinded.
Blinding of outcome assessment	Yes	No outcomes were assessed (counts of responses only).
Incomplete outcome data	Yes	Exclusions were reported.
Selective reporting	Yes	Outcomes reported in full
Other sources of bias	Yes	No grounds for concern about other sources of bias from reading the study report

**Table:** Gaski 2004a

***Study characteristics***		
Methods	Random allocation: using coin toss	
Data	US "mass‐market" dealers of the Gillette company's paper mate division	
Comparisons	1. Dissertation referencing in the cover letter 2. No dissertation referencing in the cover letter	
Outcomes	Response period not specified	
Topic	Non‐health: behavioural relations between manufacturer and its distributor	
Mode of Administration	Postal	
Notes	—	
***Risk of bias***		
**Item**	**Authors' judgement**	**Support for judgement**
Allocation concealment?	No	C ‐ inadequate

**Table:** Gaski 2004b

***Study characteristics***		
Methods	Random allocation: using coin toss	
Data	Wholesalers serving stationery/ office supply and school supply stores	
Comparisons	1. Dissertation referencing in the cover letter 2. No dissertation referencing in the cover letter	
Outcomes	Response period not specified	
Topic	Non‐health: behavioural relations between a manufacturer and its distributor	
Mode of Administration	Postal	
Notes	—	
***Risk of bias***		
**Item**	**Authors' judgement**	**Support for judgement**
Allocation concealment?	No	C ‐ inadequate

**Table:** Gates 2009

***Study characteristics***		
Methods	Quasi‐randomisation: sequentially numbered	
Data	2144 participants in the Managing Injuries of the Neck Trial (MINT), presenting to ED with whiplash injuries	
Comparisons	Non‐monetary incentive (£5 gift voucher) with follow‐up vs. no incentive	
Outcomes	Response after 1 reminder and 3 telephone calls	
Topic	Health (neck injury management)	
Mode of Administration	Postal	
Notes	49 questions; 15 A4 pages; consisted of the Neck Disability Index (NDI), two standard quality of life measures (SF‐12 and EQ‐5D), and questions on resource use and beliefs about neck pain. Questionnaire was sent out with a personalised covering letter and an SAE. A second copy of the questionnaire was sent after 2 weeks, followed by up to 3 attempts to make contact by telephone to request return of the questionnaire. Finally, participants were offered the option to provide the most important outcome data by telephone.	
***Risk of bias***		
**Item**	**Authors' judgement**	**Support for judgement**
Sequence generation	Yes	Quasi‐randomisation: sequentially numbered
Allocation concealment?	Yes	Quasi‐randomisation: sequentially numbered
Blinding of participants and personnel	No	Neither participants nor personnel were blinded.
Blinding of outcome assessment	Yes	No outcomes were assessed (counts of responses only).
Incomplete outcome data	Yes	Exclusions were reported.
Selective reporting	Yes	Response after 1 reminder and 3 telephone calls reported in full
Other sources of bias	Yes	No grounds for concern about other sources of bias from reading the study report

**Table:** Gattellari 2001

***Study characteristics***		
Methods	Random allocation: computerised random number generation	
Data	All active fellows of the Royal Australasian College of Surgeons (RACS)	
Comparisons	1. Promise of a $A10 donation to RACS for every returned questionnaire 2. No offer of donation	
Outcomes	Response period not specified	
Topic	Health: need for evidence‐based clinical practice guidelines for the management of colorectal cancer	
Mode of Administration	Postal	
Notes	—	
***Risk of bias***		
**Item**	**Authors' judgement**	**Support for judgement**
Allocation concealment?	Yes	A ‐ adequate

**Table:** Gattellari 2004

***Study characteristics***		
Methods	Random allocation: using block randomisation	
Data	Men from general practice surgeries in Sydney, Australia	
Comparisons	1. Mention of deadline to return the questionnaire within 1 week 2. No mention of deadline	
Outcomes	Response period not specified	
Topic	Health: prostate cancer screening	
Mode of Administration	Postal	
Notes	—	
***Risk of bias***		
**Item**	**Authors' judgement**	**Support for judgement**
Allocation concealment?	Yes	A ‐ adequate

**Table:** Gattellari 2012

***Study characteristics***		
Methods	Random allocation: computer‐generated	
Data	1000 randomly selected Australian family physicians	
Comparisons	Fax pre‐contact vs. mail pre‐contact Label affixed to the seal of the envelope with printed request (Attention: to be opened by doctor only) vs. blank label Non‐responders re‐randomised: final reminder more personalised vs. less	
Outcomes	Response after 3 reminders (day 209)	
Topic	Health (management of nonvalvular atrial fibrillation)	
Mode of Administration	Postal	
Notes	Each questionnaire was printed in blue ink on a sand‐coloured 24‐page, saddle‐stitched, A4 sized booklet. All questionnaires were mailed in a yellow‐coloured envelope, with institutional title, logo, and return address printed on the top left‐hand corner. A business reply‐paid envelope was included. Faxed prompts were sent 8‐15 days before the mailout of questionnaires, and mailed advance prompts were sent 7 days before questionnaires were mailed. The first author prepared all mailouts, including the advance mailed prompt letters, whereas administrative assistants were responsible for faxing prompt letters.	
***Risk of bias***		
**Item**	**Authors' judgement**	**Support for judgement**
Sequence generation	Yes	Random allocation: computer‐generated
Allocation concealment?	Yes	Random allocation: computer‐generated
Blinding of participants and personnel	Unclear	Blinding of personnel not described. Participants were not blinded to intervention.
Blinding of outcome assessment	Yes	No outcomes were assessed (counts of responses only).
Incomplete outcome data	Yes	Exclusions were reported.
Selective reporting	Yes	Response after 3 reminders reported in full
Other sources of bias	Yes	No grounds for concern about other sources of bias from reading the study report

**Table:** Gendall 1996

***Study characteristics***		
Methods	Random allocation: using alternation	
Data	New Zealanders aged 18 and above, selected from the New Zealand electoral rolls	
Comparisons	1. Cover with simple graphic design in black letters 2. Cover with complex design in black and red letters 3. Cover with different complex design in black and red letters 4. Inclusion of a picture or a photo 5. Without a picture or a photo	
Outcomes	Response rate at 12 weeks	
Topic	Health: demography ‐ family and changing gender roles	
Mode of Administration	Postal	
Notes	—	
***Risk of bias***		
**Item**	**Authors' judgement**	**Support for judgement**
Allocation concealment?	No	C ‐ inadequate

**Table:** Gendall 1998

***Study characteristics***		
Methods	Random allocation: method not specified	
Data	Individuals on the electoral roll (New Zealand)	
Comparisons	1. Control 2. High quality foil‐wrapped tea bag included 3. $1 coin included 2 follow‐ups sent	
Outcomes	—	
Topic	Non‐health: role of government, attitudes to work orientations	
Mode of Administration	Postal	
Notes	—	
***Risk of bias***		
**Item**	**Authors' judgement**	**Support for judgement**
Allocation concealment?	Unclear	B ‐ unclear

**Table:** Gendall 2005a

***Study characteristics***		
Methods	Random allocation: using alternation	
Data	New Zealanders aged 18 and above, selected from the New Zealand electoral rolls	
Comparisons	1. Personalised cover letter 2. Non‐personalised cover letter	
Outcomes	Response period not specified	
Topic	Health: environmental issues, demographics	
Mode of Administration	Postal	
Notes	—	
***Risk of bias***		
**Item**	**Authors' judgement**	**Support for judgement**
Allocation concealment?	Unclear	B ‐ unclear

**Table:** Gendall 2005b

***Study characteristics***		
Methods	Random allocation: using alternation	
Data	New Zealanders aged 18 and above, selected from the New Zealand electoral rolls	
Comparisons	1. Cover design ‐ circle 2. Cover design ‐ blocks 3. Cover design ‐ no graphics	
Outcomes	Response period not specified	
Topic	Health: demographics, disability issues, families and friends, experiences of funerals	
Mode of Administration	Postal	
Notes	—	
***Risk of bias***		
**Item**	**Authors' judgement**	**Support for judgement**
Allocation concealment?	Unclear	B ‐ unclear

**Table:** Gendall 2005c

***Study characteristics***		
Methods	Random allocation: using alternation	
Data	New Zealanders aged 18 and above, selected from the New Zealand electoral rolls	
Comparisons	1. Two 45‐cent postage stamp 2. Foil‐wrapped Dilmah tea bag 3. Small foil‐wrapped gold coin with a 20 cent denomination 4. Large foil‐wrapped gold coin with either a 50 cent or $2 denomination 5. No incentives	
Outcomes	Response period not specified	
Topic	Non‐health: citizenship in new land	
Mode of Administration	Postal	
Notes	—	
***Risk of bias***		
**Item**	**Authors' judgement**	**Support for judgement**
Allocation concealment?	Unclear	B ‐ unclear

**Table:** Gendall 2008

***Study characteristics***		
Methods	Random allocation: method unspecified	
Data	New Zealand general public	
Comparisons	No incentive vs. two 45‐cent postage stamps vs. foil‐wrapped tea bag vs. foil‐wrapped gold chocolate coin vs. chocolate square	
Outcomes	Response after 2 reminders	
Topic	Non‐health (immigration, the role of government, advertising regulation, and work)	
Mode of Administration	Postal	
Notes	Questionnaires for each survey not described The surveys involved an initial posting followed by two reminder letters to non‐respondents, the second with a questionnaire, the first without	
***Risk of bias***		
**Item**	**Authors' judgement**	**Support for judgement**
Sequence generation	Unclear	Random allocation: method unspecified
Allocation concealment?	Unclear	Random allocation: method unspecified
Blinding of participants and personnel	Unclear	Blinding of personnel not described. Participants were not blinded to intervention.
Blinding of outcome assessment	Yes	No outcomes were assessed (counts of responses only).
Incomplete outcome data	No	Exclusions not reported
Selective reporting	Yes	Response after 2 reminders reported in full
Other sources of bias	Yes	No grounds for concern about other sources of bias from reading the study report

**Table:** Gibson 1999a

***Study characteristics***		
Methods	Random allocation: randomisation sequence generated using random number function in SAS	
Data	Medicaid subjects from families that included at least 1 member who had been enrolled continuously in the respective programme from July‐Dec 1993	
Comparisons	1. Medicaid; no incentive 2. Medicaid; $1 incentive 3. Medicaid; $2 incentive 4. Basic Health Plan; no incentive 5. Basic Health Plan; $1 6. Basic Health Plan; $2 Non‐respondents after second mailing were randomised to receive third mailing by certified mail or by 2‐day priority mail.	
Outcomes	—	
Topic	Health: access and use of health services, demographics	
Mode of Administration	Postal	
Notes	Method of allocation ascertained through contact with author. No information on allocation concealment provided	
***Risk of bias***		
**Item**	**Authors' judgement**	**Support for judgement**
Allocation concealment?	Unclear	B ‐ unclear

**Table:** Gibson 1999b

***Study characteristics***		
Methods	Random allocation: randomisation sequence generated using random number function in SAS	
Data	BHP subjects from all families that included at least 1 member who had been enrolled continuously in the respective programme from July‐Dec 1993	
Comparisons	1. Medicaid; no incentive 2. Medicaid; $1 incentive 3. Medicaid; $2 incentive 4. Basic Health Plan; no incentive 5. Basic Health Plan; $1 6. Basic Health Plan; $2 Non‐respondents after second mailing were randomised to receive third mailing by certified mail or by 2‐day priority mail.	
Outcomes	—	
Topic	Health: access to health services, use of services, satisfaction with services, demographics	
Mode of Administration	Postal	
Notes	Method of allocation ascertained through contact with author. No information on allocation concealment provided.	
***Risk of bias***		
**Item**	**Authors' judgement**	**Support for judgement**
Allocation concealment?	Unclear	B ‐ unclear

**Table:** Gibson 1999c

***Study characteristics***		
Methods	Random allocation: randomisation sequence generated using random number function in SAS	
Data	Non‐responding Medicaid subjects from families that included at least 1 member who had been enrolled continuously in the respective programme from July‐Dec 1993	
Comparisons	1. Certified mail 2. 2‐day priority mail	
Outcomes	—	
Topic	Health: access and use of health services, demographics	
Mode of Administration	Postal	
Notes	Method of allocation ascertained through contact with author. No information on allocation concealment provided	
***Risk of bias***		
**Item**	**Authors' judgement**	**Support for judgement**
Allocation concealment?	Unclear	B ‐ unclear

**Table:** Giles 1978

***Study characteristics***		
Methods	Random allocation: method not specified	
Data	Faculty members	
Comparisons	1. Combination format (CombF); satisfaction items first (S1st); 2 demographic items (DI) 2. CombF; S1st; 4DI 3. CombF; S1st; 6DI 4. CombF; S1st; 8DI 5. CombF; S1st; 10DI 6. CombF; Demographic items first (D1st); 2DI 7. CombF; D1st; 4DI 8. CombF; D1st; 6DI 9. CombF; D1st; 8DI 10. CombF; D1st; 10DI 11. Categorical Format (CategF); S1st; 2DI 12. CategF; S1st; 4DI 13. CategF; S1st; 6DI 14. CategF; S1st; 8DI 15. CategF; S1st; 10DI 16. CategF; D1st; 2DI 17. CategF; D1st; 4DI 18. CategF; D1st; 6DI 19. CategF; D1st; 8DI 20. CategF; D1st; 10DI No follow‐ups used	
Outcomes	Response period not specified	
Topic	Non‐health: job satisfaction	
Mode of Administration	Postal	
Notes	—	
***Risk of bias***		
**Item**	**Authors' judgement**	**Support for judgement**
Allocation concealment?	Unclear	B ‐ unclear

**Table:** Gillpatrick 1994

***Study characteristics***		
Methods	Random allocation: method not specified	
Data	Engineers identified from the subscriber list of a major trade journal	
Comparisons	1. $1 incentive 2. No incentive 3. Pre‐contact 4. No pre‐contact	
Outcomes	Response period not specified	
Topic	Non‐health: market perceptions about CAD workstations	
Mode of Administration	Postal	
Notes	—	
***Risk of bias***		
**Item**	**Authors' judgement**	**Support for judgement**
Allocation concealment?	Unclear	B ‐ unclear

**Table:** Gitelson 1992

***Study characteristics***		
Methods	Random allocation: method not specified	
Data	Spectators at the Pennsylvania Farm Show who had not responded to 3 previous mailings of the questionnaire	
Comparisons	1. Non‐personalised questionnaire; regular post 2. Personalised questionnaire; regular post 3. Personalised questionnaire; certified post	
Outcomes	Response period not specified	
Topic	Non‐health: economic impact of the farm show	
Mode of Administration	Postal	
Notes	—	
***Risk of bias***		
**Item**	**Authors' judgement**	**Support for judgement**
Allocation concealment?	Unclear	B ‐ unclear

**Table:** Gjostein 2016

***Study characteristics***		
Methods	Random allocation: method unspecified	
Data	Participants in a colonoscopy screening study for colorectal cancer	
Comparisons	No incentive vs. unconditional non‐monetary incentive (lottery scratch card) Unconditional non‐monetary incentive (lottery scratch card) vs. conditional non‐monetary incentive (prize draw for an iPad)	
Outcomes	Response after one reminder	
Topic	Health (questionnaire on risk factors for colon cancer)	
Mode of Administration	Mixed‐mode	
Notes	One‐page questionnaire: the questionnaire contained four domains: height and weight, self‐reported diagnoses, smoking and family history of colorectal cancer. Information letter (which also detailed the terms of consent for participation), questionnaire and an SAE. The questionnaire could be completed online. After 3 weeks, non‐respondents were contacted by telephone. Response rate could be influenced by two factors. Firstly, by the study group (those invited for screening and the control group). The incentive arm represented the other factor that could influence the response rate. six different groups ‐ three study arms with different incentives for those invited for screening and the control group respectively	
***Risk of bias***		
**Item**	**Authors' judgement**	**Support for judgement**
Sequence generation	Unclear	Random allocation: method unspecified
Allocation concealment?	Unclear	Random allocation: method unspecified
Blinding of participants and personnel	Unclear	Blinding of personnel not described. Participants were not blinded to intervention.
Blinding of outcome assessment	Yes	No outcomes were assessed (counts of responses only).
Incomplete outcome data	Unclear	Unclear if response rates adjusted for exclusions (54 postal returns)
Selective reporting	Yes	Response after one reminder reported in full
Other sources of bias	Yes	No grounds for concern about other sources of bias from reading the study report

**Table:** Glidewell 2012a

***Study characteristics***		
Methods	Random allocation: method unspecified	
Data	Study a: random sample of dental practitioners in Scotland	
Comparisons	Study a: Longer questionnaire vs. longer questionnaire plus unconditional non‐monetary incentive (£20 book voucher) vs. shorter questionnaire	
Outcomes	First response	
Topic	Health (psychological frameworks which would be predictive of evidence‐based clinical behaviour)	
Mode of Administration	Postal	
Notes	10‐page full length (81 questions) questionnaire Participants were mailed an invitation pack (letter of invitation, questionnaire, a form requesting consent to allow the research team to access the respondent's radiograph or prescribing data from centrally held databases, a study newsletter, and a reply‐paid envelope).	
***Risk of bias***		
**Item**	**Authors' judgement**	**Support for judgement**
Sequence generation	Unclear	Random allocation: method unspecified
Allocation concealment?	Unclear	Random allocation: method unspecified
Blinding of participants and personnel	Yes	Secretaries recording receipt of RCTA and RCTB survey packs were blinded to group assignment.
Blinding of outcome assessment	Yes	No outcomes were assessed (counts of responses only).
Incomplete outcome data	Yes	Exclusions reported
Selective reporting	Yes	Outcomes reported in full
Other sources of bias	Yes	No grounds for concern about other sources of bias from reading the study report

**Table:** Glidewell 2012b

***Study characteristics***		
Methods	Random allocation: method unspecified	
Data	Study b: Random sample of general practitioners in the UK who were non‐responders to a previous postal questionnaire (n = 847)	
Comparisons	Study b: questionnaire reminder vs. postcard reminder	
Outcomes	First response	
Topic	Health (psychological frameworks which would be predictive of evidence‐based clinical behaviour)	
Mode of Administration	Postal	
Notes	10‐page full length (81 questions) questionnaire Participants were mailed an invitation pack (letter of invitation, questionnaire, a form requesting consent to allow the research team to access the respondent's radiograph or prescribing data from centrally‐held databases, a study newsletter, and a reply‐paid envelope).	
***Risk of bias***		
**Item**	**Authors' judgement**	**Support for judgement**
Sequence generation	Unclear	Random allocation: method unspecified
Allocation concealment?	Unclear	Random allocation: method unspecified
Blinding of participants and personnel	Yes	Secretaries recording receipt of RCTA and RCTB survey packs were blinded to group assignment.
Blinding of outcome assessment	Yes	No outcomes were assessed (counts of responses only).
Incomplete outcome data	Yes	Exclusions reported
Selective reporting	Yes	Outcomes reported in full
Other sources of bias	Yes	No grounds for concern about other sources of bias from reading the study report

**Table:** Glisan 1982

***Study characteristics***		
Methods	Random allocation: method not specified	
Data	Farmers from 6 geographical regions	
Comparisons	1. Incentive ‐ monetary 2. Incentive ‐ results promised 3. Incentive ‐ control 4. Colour ‐ tan 5. Colour ‐ blue 6. Colour ‐ white 7. Stamp ‐ commemorative 8. Stamp ‐ regular	
Outcomes	Response period not specified	
Topic	Non‐health: farm operations and costs	
Mode of Administration	Postal	
Notes	—	
***Risk of bias***		
**Item**	**Authors' judgement**	**Support for judgement**
Allocation concealment?	Unclear	B ‐ unclear

**Table:** Godwin 1979

***Study characteristics***		
Methods	Random allocation: method not specified	
Data	Individuals in 60 countries	
Comparisons	1. No incentive 2. $25 3. $50 Participants were requested to return the questionnaire within 3 weeks. After 2 weeks, a single follow‐up letter with a copy of the questionnaire was sent to all respondents.	
Outcomes	Response period not specified	
Topic	Health: family planning programmes	
Mode of Administration	Postal	
Notes	—	
***Risk of bias***		
**Item**	**Authors' judgement**	**Support for judgement**
Allocation concealment?	Unclear	B ‐ unclear

**Table:** Goldstein 1975

***Study characteristics***		
Methods	Random allocation: method not specified	
Data	Subscribers to a travel magazine	
Comparisons	1. Postcard format first wave; postcard format second wave 2. Form first wave; form second wave 3. Postcard format first wave; form second wave 4. Form first wave; postcard format second wave	
Outcomes	Response period not specified	
Topic	Non‐health	
Mode of Administration	Postal	
Notes	—	
***Risk of bias***		
**Item**	**Authors' judgement**	**Support for judgement**
Allocation concealment?	Unclear	B ‐ unclear

**Table:** Goodstadt 1977

***Study characteristics***		
Methods	Random allocation: method not specified	
Data	Readers of Addictions Magazine	
Comparisons	1. 25 cent incentive 2. Free book incentive 3. Promise of free book 4. No incentive	
Outcomes	—	
Topic	Non‐health: reading habits, magazine function served, the range and depth of subjects covered, overall design	
Mode of Administration	Postal	
Notes	—	
***Risk of bias***		
**Item**	**Authors' judgement**	**Support for judgement**
Allocation concealment?	Unclear	B ‐ unclear

**Table:** Goodwin 2020

***Study characteristics***		
Methods	Random allocation: computer‐generated	
Data	Parents of a child born in Cumbria, UK 2014‐15, who had not responded to 2 waves of a survey	
Comparisons	Postcard reminder vs. telephone reminder vs. questionnaire reminder	
Outcomes	Final response	
Topic	Health (children's oral and general health)	
Mode of Administration	Postal	
Notes		
***Risk of bias***		
**Item**	**Authors' judgement**	**Support for judgement**
Sequence generation	Yes	Random allocation: computer‐generated
Allocation concealment?	Yes	Random allocation: computer‐generated
Blinding of participants and personnel	Unclear	Blinding of personnel not described. Participants were not blinded to intervention.
Blinding of outcome assessment	Yes	No outcomes were assessed (counts of responses only).
Incomplete outcome data	No	Exclusions were not reported.
Selective reporting	Yes	Final response reported in full
Other sources of bias	Yes	No grounds for concern about other sources of bias from reading the study report

**Table:** Goulao 2020a

***Study characteristics***		
Methods	Random allocation: automated, central randomisation service	
Data	Regular attenders to the dentists and with overall good oral health	
Comparisons	Study a: Sticker added to the top left corner of envelope vs. no sticker	
Outcomes	Response after 6 weeks	
Topic	Health (patient‐reported outcomes in oral health and dentistry)	
Mode of Administration	Postal	
Notes	Questionnaires were issued with a cover letter using a semi‐automated process; if not returned within 3 weeks of issue of the first questionnaire, a reminder letter and second questionnaire were sent. As well as testing whether the BCTs incorporated in a different format to the cover letter (i.e. a newsletter) encouraged return of questionnaires, we were able to test a second research question: does the timing of delivery of a newsletter affect response rates? The intervention group received the newsletter before the first questionnaires and the control group received it with the second (reminder) or after return of the first questionnaire.	
***Risk of bias***		
**Item**	**Authors' judgement**	**Support for judgement**
Sequence generation	Yes	Random allocation: automated, central randomisation service
Allocation concealment?	Yes	Random allocation: automated, central randomisation service
Blinding of participants and personnel	Unclear	Blinding of personnel not described. Participants were not blinded to intervention.
Blinding of outcome assessment	Yes	No outcomes were assessed (counts of responses only).
Incomplete outcome data	No	Exclusions not reported
Selective reporting	Yes	Response after 6 weeks reported in full
Other sources of bias	Yes	No grounds for concern about other sources of bias from reading the study report

**Table:** Goulao 2020b

***Study characteristics***		
Methods	Random allocation: automated, central randomisation service	
Data	Regular attenders to the dentists and with overall good oral health	
Comparisons	Study b: Standard cover letter vs. letter with behaviour change techniques in the text	
Outcomes	Response after 6 weeks	
Topic	Health (patient‐reported outcomes in oral health and dentistry)	
Mode of Administration	Postal	
Notes	Questionnaires were issued with a cover letter using a semi‐automated process; if not returned within 3 weeks of issue of the first questionnaire, a reminder letter and second questionnaire were sent. As well as testing whether the BCTs incorporated in a different format to the cover letter (i.e. a newsletter) encouraged return of questionnaires, we were able to test a second research question: does the timing of delivery of a newsletter affect response rates? The intervention group received the newsletter before the first questionnaires and the control group received it with the second (reminder) or after return of the first questionnaire.	
***Risk of bias***		
**Item**	**Authors' judgement**	**Support for judgement**
Sequence generation	Yes	Random allocation: automated, central randomisation service
Allocation concealment?	Yes	Random allocation: automated, central randomisation service
Blinding of participants and personnel	Unclear	Blinding of personnel not described. Participants were not blinded to intervention.
Blinding of outcome assessment	Yes	No outcomes were assessed (counts of responses only).
Incomplete outcome data	No	Exclusions not reported
Selective reporting	Yes	Response after 6 weeks reported in full
Other sources of bias	Yes	No grounds for concern about other sources of bias from reading the study report

**Table:** Goulao 2020c

***Study characteristics***		
Methods	Random allocation: automated, central randomisation service	
Data	Regular attenders to the dentists and with overall good oral health	
Comparisons	Study c: Pre‐contact study newsletter with behaviour change techniques in the text vs. newsletter with reminder	
Outcomes	Response after 6 weeks	
Topic	Health (patient‐reported outcomes in oral health and dentistry)	
Mode of Administration	Postal	
Notes	Questionnaires were issued with a cover letter using a semi‐automated process; if not returned within 3 weeks of issue of the first questionnaire, a reminder letter and second questionnaire were sent. As well as testing whether the BCTs incorporated in a different format to the cover letter (i.e. a newsletter) encouraged return of questionnaires, we were able to test a second research question: does the timing of delivery of a newsletter affect response rates? The intervention group received the newsletter before the first questionnaires and the control group received it with the second (reminder) or after return of the first questionnaire.	
***Risk of bias***		
**Item**	**Authors' judgement**	**Support for judgement**
Sequence generation	Yes	Random allocation: automated, central randomisation service
Allocation concealment?	Yes	Random allocation: automated, central randomisation service
Blinding of participants and personnel	Unclear	Blinding of personnel not described. Participants were not blinded to intervention.
Blinding of outcome assessment	Yes	No outcomes were assessed (counts of responses only).
Incomplete outcome data	No	Exclusions not reported
Selective reporting	Yes	Response after 6 weeks reported in full
Other sources of bias	Yes	No grounds for concern about other sources of bias from reading the study report

**Table:** Green 1986

***Study characteristics***		
Methods	Random allocation: method not specified	
Data	Teachers chosen from the Wyoming State Department of Education list of educators	
Comparisons	1. Personalised cover letter 2. Non‐personalised cover letter	
Outcomes	—	
Topic	Non‐health: courses taken in tests and measurement, attitudes towards standardised and classroom testing, interest in topics for inservice training	
Mode of Administration	Postal	
Notes	Mainly females	
***Risk of bias***		
**Item**	**Authors' judgement**	**Support for judgement**
Allocation concealment?	Unclear	B ‐ unclear

**Table:** Green 1989

***Study characteristics***		
Methods	Random allocation: method not specified	
Data	Inservice teachers from the states of Wyoming and Nebraska	
Comparisons	1. Offer of a summary of results vs none 2. Personalisation vs no personalisation	
Outcomes	—	
Topic	Non‐health: application of research methods and findings to classroom teaching	
Mode of Administration	Postal	
Notes	—	
***Risk of bias***		
**Item**	**Authors' judgement**	**Support for judgement**
Allocation concealment?	Unclear	B ‐ unclear

**Table:** Green 2000

***Study characteristics***		
Methods	Random allocation: method not specified	
Data	Randomly selected US social workers	
Comparisons	1. Demographic items placed at the beginning of the questionnaire 2. Demographic items placed at the end of the questionnaire	
Outcomes	—	
Topic	Health: attitudes and beliefs about roles of family interaction and biological factors in mental illness	
Mode of Administration	Postal	
Notes	Mean age: 44.5 years	
***Risk of bias***		
**Item**	**Authors' judgement**	**Support for judgement**
Allocation concealment?	Unclear	B ‐ unclear

**Table:** Greer 1994

***Study characteristics***		
Methods	Random allocation: method not specified	
Data	Senior sales executives (US)	
Comparisons	1. University sponsor; white questionnaire 2. University sponsor; yellow questionnaire 3. University sponsor; pink questionnaire 4. University sponsor; green questionnaire 5. Commercial research sponsor; white questionnaire 6. Commercial research sponsor; yellow questionnaire 7. Commercial research sponsor; pink questionnaire 8. Commercial research sponsor; green questionnaire 9. Academic honour society sponsor; white questionnaire 10. Academic honour society sponsor; yellow questionnaire 11. Academic honour society sponsor; pink questionnaire 12. Academic honour society sponsor; green questionnaire 13. No sponsor (PO Box); white questionnaire 14. No sponsor (PO Box); yellow questionnaire 15. No sponsor (PO Box); pink questionnaire 16. No sponsor (PO Box); green questionnaire Follow‐up sent after 3 weeks	
Outcomes	Response period not specified	
Topic	Non‐health: company's programme for sales people	
Mode of Administration	Postal	
Notes	—	
***Risk of bias***		
**Item**	**Authors' judgement**	**Support for judgement**
Allocation concealment?	Unclear	B ‐ unclear

**Table:** Griffin 2011

***Study characteristics***		
Methods	Random allocation: random numbers	
Data	Non‐respondents to a face‐to‐face survey of US veterans	
Comparisons	Smaller unconditional monetary incentive ($2) vs. larger unconditional monetary incentive ($5)	
Outcomes	First response and final response after two reminders (4 weeks)	
Topic	Non‐health (survey designed to characterise non‐respondents)	
Mode of Administration	Postal	
Notes	10‐item questionnaire (one page) Surveys were mailed using first‐class postage, including a cover letter, an SAE. A reminder postcard was mailed approximately one week after the first packet. A second questionnaire packet without an incentive was mailed to any subject who did not return a blank or completed survey within 3‐4 weeks of the first mailing.	
***Risk of bias***		
**Item**	**Authors' judgement**	**Support for judgement**
Sequence generation	Yes	Random allocation: random numbers
Allocation concealment?	Yes	Random allocation: random numbers
Blinding of participants and personnel	Unclear	Blinding of personnel not described. Participants were not blinded to intervention.
Blinding of outcome assessment	Yes	No outcomes were assessed (counts of responses only).
Incomplete outcome data	No	Exclusions were not reported.
Selective reporting	Yes	First response and final response after two reminders (4 weeks) reported in full
Other sources of bias	Yes	No grounds for concern about other sources of bias from reading the study report

**Table:** Griffith 1999

***Study characteristics***		
Methods	Random allocation: method not specified	
Data	General medical internists in 5 Canadian provinces	
Comparisons	1. Open‐ended questionnaire format 2. Close‐ended questionnaire format	
Outcomes	—	
Topic	Health: career satisfaction	
Mode of Administration	Postal	
Notes	Mean age: 51.9 years; mainly males	
***Risk of bias***		
**Item**	**Authors' judgement**	**Support for judgement**
Allocation concealment?	Unclear	B ‐ unclear

**Table:** Groeneman 1986

***Study characteristics***		
Methods	Random allocation: method not specified	
Data	People listed in the telephone directories of Toronto, Montreal, Vancouver and Winnipeg, Canada selected using 'distinctive Jewish name sampling'	
Comparisons	1. $1 bill enclosed 2. No incentive	
Outcomes	Response period not specified	
Topic	Non‐health: travel experience and attitudes towards future trips	
Mode of Administration	Postal	
Notes	Method of allocation ascertained through contact with author	
***Risk of bias***		
**Item**	**Authors' judgement**	**Support for judgement**
Allocation concealment?	Unclear	B ‐ unclear

**Table:** Groves 2000

***Study characteristics***		
Methods	Random allocation: method not specified	
Data	Therapeutic recreation co‐ordinators in the US	
Comparisons	1. Stamped addressed return envelope included 2. Self‐adhering return address level (no envelope or postage) included	
Outcomes	Response period not specified	
Topic	—	
Mode of Administration	Postal	
Notes	Method of allocation ascertained through contact with author	
***Risk of bias***		
**Item**	**Authors' judgement**	**Support for judgement**
Allocation concealment?	Unclear	B ‐ unclear

**Table:** Gueguen 2003a

***Study characteristics***		
Methods	Random allocation: using random number generation	
Data	University students	
Comparisons	1. Attractive photo in the email 2. Medium attractive photo in the email 3. No photo in the email 4. Male signature 5. Female signature	
Outcomes	Response period not specified	
Topic	Health: dietary habits	
Mode of Administration	Electronic: email	
Notes	Equal males and females; language of publication is French.	
***Risk of bias***		
**Item**	**Authors' judgement**	**Support for judgement**
Allocation concealment?	No	C ‐ inadequate

**Table:** Gueguen 2003b

***Study characteristics***		
Methods	Random allocation: using random number generation	
Data	Sample of individuals with email addresses ending in ".fr" picked up randomly from the Internet using specialised software	
Comparisons	1. Attractive photo in the email 2. Medium attractive photo in the email 3. Less attractive photo in the email 4. No photo in the email 5. Male signature 6. Female signature	
Outcomes	Response period not specified	
Topic	Health: dietary habits	
Mode of Administration	Electronic: email	
Notes	Language of publication is French.	
***Risk of bias***		
**Item**	**Authors' judgement**	**Support for judgement**
Allocation concealment?	No	C ‐ inadequate

**Table:** Gullahorn 1959

***Study characteristics***		
Methods	Random allocation: method not specified	
Data	Former Fulbright & Smith‐Mundt grantees who had not responded to the earlier mailing of the questionnaire	
Comparisons	1. Follow‐up mailings by special delivery 2. Follow‐up mailings by standard mail	
Outcomes	Response period not specified	
Topic	Non‐health: bibliography of works emanating form Fulbright and Smith‐Mundt awards	
Mode of Administration	Postal	
Notes	—	
***Risk of bias***		
**Item**	**Authors' judgement**	**Support for judgement**
Allocation concealment?	Unclear	B ‐ unclear

**Table:** Gullahorn 1963

***Study characteristics***		
Methods	Random allocation: method not specified	
Data	Former Fulbright and Smith‐Mundt grantees	
Comparisons	1. Questionnaire printed on green paper, sent by first‐class mail, with business‐reply return envelope 2. Green paper; first‐class, stamped return envelope 3. Green paper, third‐class mail, business‐reply return envelope 4. Green paper, third‐class mail, return envelope stamped 5. White paper, first‐class mail, business‐reply return envelope 6. White paper, first‐class, return envelope stamped 7. White paper, third‐class mail, business‐reply return envelope 8. White paper, third‐class mail, return envelope stamped	
Outcomes	—	
Topic	—	
Mode of Administration	Postal	
Notes	—	
***Risk of bias***		
**Item**	**Authors' judgement**	**Support for judgement**
Allocation concealment?	Unclear	B ‐ unclear

**Table:** Guo 2016

***Study characteristics***		
Methods	Random allocation: method unspecified	
Data	Randomly selected community‐dwelling adults in British Columbia	
Comparisons	Shorter vs. longer (LC incentive vs. LC short) No incentive vs. non‐monetary incentive (instant lottery) vs. monetary incentive group ($2 prepaid coin) vs. non‐monetary incentive plus monetary incentive (both instant lottery plus $2 prepaid coin)	
Outcomes	Response after 3 reminders	
Topic	Health (general health, quality of life, and use of health services, with an emphasis on osteoarthritis)	
Mode of Administration	Online	
Notes	Short (10 minutes); longer (30 minutes). Invitation letters sent (with experimental incentive conditions) followed by 3 reminders The instant lottery was for 10 prizes of $100 (with odds of 1 in 800), and a grand prize of $1000 (with odds of 1 in 8000).	
***Risk of bias***		
**Item**	**Authors' judgement**	**Support for judgement**
Sequence generation	Unclear	Random allocation: method unspecified
Allocation concealment?	Unclear	Random allocation: method unspecified
Blinding of participants and personnel	Unclear	Blinding of personnel not described. Participants were not blinded to intervention.
Blinding of outcome assessment	Yes	No outcomes were assessed (counts of responses only).
Incomplete outcome data	No	Exclusions were not reported.
Selective reporting	Yes	Outcomes reported in full
Other sources of bias	Yes	No grounds for concern about other sources of bias from reading the study report

**Table:** Gupta 1997

***Study characteristics***		
Methods	Random allocation: method not specified	
Data	Medical practitioners	
Comparisons	1. Telephone prompt by medical researcher 2. Telephone prompt by an experienced non‐medical research assistant	
Outcomes	—	
Topic	Health: views about clinical practice guidelines	
Mode of Administration	Postal	
Notes	—	
***Risk of bias***		
**Item**	**Authors' judgement**	**Support for judgement**
Allocation concealment?	Unclear	B ‐ unclear

**Table:** Göritz 2004a

***Study characteristics***		
Methods	Random allocation: using computerised random number generation	
Data	Panellists from the German commercial online access panel	
Comparisons	1. 7 X 100 (700) German Mark (DM) money lottery 2. 14 X 50 (700) DM money lottery 3. 5 X 100 (500) DM money lottery 4. 10 X 50 (500) DM money lottery 5. 3 X 100 (300) DM money lottery 6. 6 X 50 (300) DM money lottery 7. 1 X 100 (100) DM money lottery 8. 2 X 50 (100) DM money lottery 9. 8 Bonus point (BP) (1 BP is worth 50 DM) 10. 6 BP 11. 4 BP 12. 3 BP 13. Gift lottery (3 watches/5 CD‐jackets/5 alarm clocks/25 key‐ring torches).	
Outcomes	Response period not specified	
Topic	Non‐health: personal Internet usage	
Mode of Administration	Electronic: online survey	
Notes	Method of allocation ascertained through contact with author	
***Risk of bias***		
**Item**	**Authors' judgement**	**Support for judgement**
Allocation concealment?	Yes	A ‐ adequate

**Table:** Göritz 2004b

***Study characteristics***		
Methods	Random allocation: using computerised random number generation	
Data	Panellists from the German commercial online access panel	
Comparisons	1. 2 X 90 (180) German Mark (DM) 2. 6 X 30 DM money lottery 3. 1 X 90 DM money lottery 4. 3 X 30 DM money lottery	
Outcomes	Response period not specified	
Topic	Non‐health: evaluation of media contents	
Mode of Administration	Electronic: online survey	
Notes	Method of allocation ascertained through contact with author	
***Risk of bias***		
**Item**	**Authors' judgement**	**Support for judgement**
Allocation concealment?	Yes	A ‐ adequate

**Table:** Hackler 1973

***Study characteristics***		
Methods	Random allocation: alternation	
Data	Mothers of ninth or tenth grade students living in 1 neighbourhood of Edmonton	
Comparisons	1. No incentive 2. $1 bill incentive After 11 days, the no incentive group received $1 and the incentive group received a follow‐up phone call.	
Outcomes	—	
Topic	Non‐health: community cohesiveness	
Mode of Administration	Postal	
Notes	Mainly females	
***Risk of bias***		
**Item**	**Authors' judgement**	**Support for judgement**
Allocation concealment?	No	C ‐ inadequate

**Table:** Hall 2013

***Study characteristics***		
Methods	Random allocation: method unspecified	
Data	800 haematological cancer survivors (18‐80) diagnosed within last 3 years, from one Australian state cancer registry	
Comparisons	Enhanced invitation letter (more readable; shorter sentences, 8th grade reading level, sans serif typeface) vs. standard letter	
Outcomes	Response after one reminder (4 weeks)	
Topic	Health (survey of unmet needs and psychological disturbance of rural and urban haematological cancer survivors)	
Mode of Administration	Postal	
Notes	Included a number of standardised measures: the Survivor Unmet Needs Survey (SUNS), the Depression Anxiety and Stress Scale (DASS), the Distress Thermometer, and an adapted version of the Control Preferences Scale. Additional author‐derived questions assessing patient disease, treatment, sociodemographic, service utilisation, and Internet use Patients were contacted directly by the cancer registry without consent, as permitted by legislation and Human Research Ethics Committee approval. Initial contact involved the mailed invitation letter along with a study package that contained: an information statement, survivor questionnaire, non‐participation form, a brochure explaining the cancer registry, reply‐paid envelope, and a questionnaire package for their principal support person. The patient's physician was not involved in patient contact or recruitment. Survivors were assured that their decision to take part in this study was entirely their choice and their decision would not affect their access to care. Non‐responders were mailed a reminder letter and an additional study package approximately 4 weeks later. Return of the survey was taken as a voluntary consent to participate. Half were randomly allocated to receive the standard invitation letter (control group). The remaining half received a modified invitation letter, incorporating content and design characteristics recommended to improve written communication (intervention group).	
***Risk of bias***		
**Item**	**Authors' judgement**	**Support for judgement**
Sequence generation	Unclear	Random allocation: method unspecified
Allocation concealment?	Unclear	Random allocation: method unspecified
Blinding of participants and personnel	Unclear	Blinding of personnel not described. Participants were not blinded to intervention.
Blinding of outcome assessment	Yes	No outcomes were assessed (counts of responses only).
Incomplete outcome data	Yes	Exclusions reported; 68 (31 from the enhanced letter group and 37 from the standard letter group) were later deemed ineligible as they were either unable to be contacted (n = 556), had died (n = 58) or were misdiagnosed (n = 54).
Selective reporting	Yes	Response after one reminder (4 weeks) reported in full
Other sources of bias	Yes	No grounds for concern about other sources of bias from reading the study report

**Table:** Hall 2019

***Study characteristics***		
Methods	Random allocation: method unspecified	
Data	Facebook users who indicated that they were male, over 18 years of age, and interested in men	
Comparisons	No incentive vs. a conditional non‐monetary incentive ($20 Amazon voucher); altruistic incentive vs. none; dashboard incentive vs. none	
Outcomes	Survey completion during a 9‐day period of advertising on Facebook (advancing to completion screen, without skipping more than two consecutive questions)	
Topic	Health (HIV prevention and risk behaviours)	
Mode of Administration	Electronic	
Notes	Monetary incentive group were offered a $20 gift code to Amazon.com at the completion of the survey. Altruistic messaging group saw 8 banner messages throughout the survey, included facts related to HIV transmission (e.g. "Every 10.5 minutes, someone is infected with HIV in the United States") and statements to highlight the importance of the individual's participation (e.g. "By taking this survey, you are making your voice heard to help stop the HIV epidemic"). Dashboard group were shown a selection of participant responses from the survey and were offered a dashboard of their own data compared to responses by other men upon survey completion.	
***Risk of bias***		
**Item**	**Authors' judgement**	**Support for judgement**
Sequence generation	Unclear	Random allocation: method unspecified
Allocation concealment?	Unclear	Random allocation: method unspecified
Blinding of participants and personnel	Unclear	Blinding of personnel not described. Participants were not blinded to intervention.
Blinding of outcome assessment	Yes	No outcomes were assessed (counts of responses only).
Incomplete outcome data	Yes	Ineligible people reported
Selective reporting	Yes	Response during a 9‐day period of advertising on Facebook reported in full
Other sources of bias	Yes	No grounds for concern about other sources of bias from reading the study report

**Table:** Halpern 2002

***Study characteristics***		
Methods	Random allocation: method not specified	
Data	General internists and family practitioners randomly selected from the American Medical Association's master file of physicians	
Comparisons	1. $10, peppermint candy and large outgoing envelope 2. $10, no peppermint candy, large outgoing envelope 3. $10, peppermint candy, small outgoing envelope 4. $10, no peppermint candy, small outgoing envelope 5. $5, peppermint candy and large outgoing envelope 6. $5, no peppermint candy, large outgoing envelope 7. $5, peppermint candy, small outgoing envelope 8. $5, no peppermint candy, small outgoing envelope	
Outcomes	Response within 11 weeks	
Topic	Health: views about comparative merits of placebo‐controlled versus active‐controlled trials of antihypertensive drugs	
Mode of Administration	Postal	
Notes	—	
***Risk of bias***		
**Item**	**Authors' judgement**	**Support for judgement**
Allocation concealment?	Unclear	B ‐ unclear

**Table:** Halpern 2011a

***Study characteristics***		
Methods	Random allocation: random number generator	
Data	2477 critical care physicians	
Comparisons	Conditional non‐monetary incentive (lottery 0.001 chance of $5k) vs. conditional non‐monetary incentive (lottery 0.02 chance of $250) vs. no incentive	
Outcomes	Sent email reminders to non‐respondents at 5 and 10 days following initial invitation	
Topic	Health (organ donation after circulatory determination of death)	
Mode of Administration	Online survey	
Notes	Questionnaire completion required 5 minutes or fewer. Sent email reminders to non‐respondents at 5 and 10 days following the initial invitation.	
***Risk of bias***		
**Item**	**Authors' judgement**	**Support for judgement**
Sequence generation	Yes	Random allocation: random number generator
Allocation concealment?	Yes	Random allocation: random number generator
Blinding of participants and personnel	Unclear	Blinding of personnel not described. Participants were not blinded to intervention.
Blinding of outcome assessment	Yes	No outcomes were assessed (counts of responses only).
Incomplete outcome data	Yes	Exclusions reported
Selective reporting	Yes	Response after reminders reported in full
Other sources of bias	Yes	No grounds for concern about other sources of bias from reading the study report

**Table:** Halpern 2011b

***Study characteristics***		
Methods	Random allocation: random number generator	
Data	1000 critical care nurses	
Comparisons	Conditional non‐monetary incentive (lottery 0.0025 chance of $2k) vs. unconditional monetary incentive ($5)	
Outcomes	Single reminder sent Completed responses (> 80 items completed)	
Topic	Health (organ donation after circulatory determination of death)	
Mode of Administration	Postal survey	
Notes	Questionnaire completion required 5 minutes or fewer. Sent a single reminder	
***Risk of bias***		
**Item**	**Authors' judgement**	**Support for judgement**
Sequence generation	Yes	Random allocation: random number generator
Allocation concealment?	Yes	Random allocation: random number generator
Blinding of participants and personnel	Unclear	Blinding of personnel not described. Participants were not blinded to intervention.
Blinding of outcome assessment	Yes	No outcomes were assessed (counts of responses only).
Incomplete outcome data	Yes	Exclusions reported
Selective reporting	Yes	Response after reminders reported in full
Other sources of bias	Yes	No grounds for concern about other sources of bias from reading the study report

**Table:** Halpern 2011c

***Study characteristics***		
Methods	Random allocation: random number generator	
Data	758 resident physicians	
Comparisons	Conditional non‐monetary incentive (lottery 0.004 chance of $2,500) vs. conditional monetary incentive ($10)	
Outcomes	Sent email reminders to non‐respondents at 5 and 10 days following initial invitation Completed responses (> 80 items completed)	
Topic	Health (physicians practice when obtaining informed consent for blood transfusion)	
Mode of Administration	Online survey	
Notes	Questionnaire completion required 5 minutes or fewer. Sent email reminders to non‐respondents at 5 and 10 days following the initial invitation	
***Risk of bias***		
**Item**	**Authors' judgement**	**Support for judgement**
Sequence generation	Yes	Random allocation: random number generator
Allocation concealment?	Yes	Random allocation: random number generator
Blinding of participants and personnel	Unclear	Blinding of personnel not described. Participants were not blinded to intervention.
Blinding of outcome assessment	Yes	No outcomes were assessed (counts of responses only).
Incomplete outcome data	Yes	Exclusions reported
Selective reporting	Yes	Response after reminders reported in full
Other sources of bias	No	It is possible that some of the reported results were influenced by participants communicating with each other regarding their assigned incentives. Although such contamination is unlikely to have affected the national samples used in Trials 1 and 2, the residents sampled at three hospitals in Trial 3 could indeed have communicated with each other.

**Table:** Hammink 2010

***Study characteristics***		
Methods	Random allocation: method unspecified	
Data	880 patients of a general practice cooperative for out‐of‐hours care	
Comparisons	(1) Pre‐notification and follow‐up vs. (2) pre‐notification only vs. (3) follow‐up only	
Outcomes	First response (within 9 days) and final response (within 1 month) after one reminder	
Topic	Health (patients' experiences with quality aspects of care in GP cooperatives)	
Mode of Administration	Postal	
Notes	7 double‐sided pages, 91 items. Pre‐notification letter (in the pre‐contact arm) or the questionnaire, accompanied by a cover letter signed by the director of the GP co‐operative, and a prepaid envelope (in the other arms) were sent 2‐5 days after contact with the GP cooperative; after 5‐7 days, the questionnaire was sent to subjects who had only received a pre‐notification. The follow‐up letter, without the inclusion of a copy of the questionnaire, was delivered to non‐responders 7‐9 days after mailing of the questionnaire.	
***Risk of bias***		
**Item**	**Authors' judgement**	**Support for judgement**
Sequence generation	Unclear	Random allocation: method unspecified
Allocation concealment?	Unclear	Random allocation: method unspecified
Blinding of participants and personnel	Unclear	Blinding of personnel not described. Participants were not blinded to intervention.
Blinding of outcome assessment	Yes	Blinding of personnel not described. Participants were not blinded to intervention.
Incomplete outcome data	Yes	Exclusions were reported.
Selective reporting	Yes	Outcomes reported in full
Other sources of bias	Yes	No grounds for concern about other sources of bias from reading the study report

**Table:** Han 2013

***Study characteristics***		
Methods	Random allocation: method unspecified	
Data	Participants in the National Household Education Surveys	
Comparisons	Lower unconditional monetary incentive ($2) vs. higher unconditional monetary incentive ($5) FedEx/priority mail vs. first‐class mail No incentive vs. unconditional monetary incentive ($5, $10, $15, $20)	
Outcomes	Response after 2 reminders	
Topic	Non‐health (educational activities of children and families in the US)	
Mode of Administration	Postal	
Notes	A screener survey was used to identify households with eligible children. Items collected on the screener instrument included the age, gender, school enrolment, and grade for each person of age 20 or younger living at the sampled address. A topical questionnaire was sent to the parents/guardians of the sampled child to collect data on the care and education of the sampled child. All the sampled addresses were mailed an initial screener questionnaire, an information letter, and an SAE. A thank‐you/reminder postcard was mailed to all addresses. Non‐respondents to the initial mailing were mailed a second screener packet, and the remaining non‐respondents were mailed a final screener packet. The initial mailing and first non‐response follow‐up mailing were sent through first‐class mail. The second non‐response follow‐up mailing was sent using either FedEx delivery or priority mail (randomly assigned). No monetary incentive was provided during the two follow‐up mailings.	
***Risk of bias***		
**Item**	**Authors' judgement**	**Support for judgement**
Sequence generation	Unclear	Random allocation: method unspecified
Allocation concealment?	Unclear	Random allocation: method unspecified
Blinding of participants and personnel	Unclear	Blinding of personnel not described. Participants were not blinded to intervention.
Blinding of outcome assessment	Yes	No outcomes were assessed (counts of responses only).
Incomplete outcome data	Yes	Exclusions were not reported.
Selective reporting	Yes	Response after 2 reminders reported in full
Other sources of bias	No	Likely confounding of study, and unclear randomisation ‐ 'It is worth noting that the effects of the second follow‐up mailing were due in part to Priority Mail, and a different pattern might hold if First Class Mail had been used'.

**Table:** Hancock 1940

***Study characteristics***		
Methods	Random allocation: method not specified	
Data	Individuals listed on the personal tax records of the county assessors	
Comparisons	1. Questionnaire and cover letter 2. As above with 25 cents incentive 3. As above with promise of 25 cents on return of questionnaire 4. Personal interview	
Outcomes	Response period not specified	
Topic	—	
Mode of Administration	Postal	
Notes	—	
***Risk of bias***		
**Item**	**Authors' judgement**	**Support for judgement**
Allocation concealment?	Unclear	B ‐ unclear

**Table:** Hansen 1980a

***Study characteristics***		
Methods	Random allocation: method not specified	
Data	Individuals listed in the telephone directory (Columbus, Ohio)	
Comparisons	1. No pre‐contact; short form (SF) 2. No pre‐contact; long form (LF) 3. Yes/no foot in the door; SF 4. Yes/no foot in the door; LF 5. Probe foot in the door; SF 6. Probe foot in the door; LF	
Outcomes	Response within 35 days	
Topic	Non‐health: consumer's attitudes towards recent new car purchases	
Mode of Administration	Postal	
Notes	—	
***Risk of bias***		
**Item**	**Authors' judgement**	**Support for judgement**
Allocation concealment?	Unclear	B ‐ unclear

**Table:** Hansen 1980b

***Study characteristics***		
Methods	Random allocation: method not specified	
Data	Safety engineers employed by firms that require employees to wear safety hardhats	
Comparisons	1. 25 cent incentive 2. Pen incentive 3. Control group	
Outcomes	Response within 38 days	
Topic	Non‐health: product evaluation and information	
Mode of Administration	Postal	
Notes	—	
***Risk of bias***		
**Item**	**Authors' judgement**	**Support for judgement**
Allocation concealment?	Unclear	B ‐ unclear

**Table:** Hardigan 2012

***Study characteristics***		
Methods	Not specified	
Data	6000 dentists taken from the Florida Department of Health, Board of Dentistry	
Comparisons	Choice (mail or web‐based), postal mail, or web‐based	
Outcomes	Survey, 2‐week follow‐up with additional survey, 1 week later reminder postcard by mail	
Topic	Tobacco use by dental patients	
Mode of Administration	Postal/electronic (email and web)	
Notes	28 questions; 4 pages; A4 Dentists who were randomised to Group A received a mailing which contained a cover letter describing the study. The envelope also contained the Florida Tobacco Control Survey 2009; a pre‐paid, self‐addressed return envelope; and a refusal postcard. Dentists were given the choice to complete the survey via mail or online. They were provided the website address, as well as a unique survey number to type in on the home page of the online survey. After entering their survey number on the homepage, the respondents were directed through a series of questions. A response to each question was required in order for the survey to proceed to the next question. Once the survey was completed, results were stored in a master database. If no response was obtained by 2 weeks after the first mailing, an additional survey was sent by mail, which the dentist again had the option of completing online. The website was displayed at the top of the first page, along with the dentists' unique survey number. If no response was obtained 1 week later, a reminder postcard was sent via mail.	
***Risk of bias***		
**Item**	**Authors' judgement**	**Support for judgement**
Sequence generation	Unclear	Random allocation ‐ method unspecified
Allocation concealment?	Unclear	Random allocation ‐ method unspecified
Blinding of participants and personnel	Unclear	Blinding of personnel not described. Participants were not blinded to intervention.
Blinding of outcome assessment	Yes	No outcome assessment used (counts of responses only)
Incomplete outcome data	Yes	Exclusions reported
Selective reporting	Yes	Stated outcomes reported
Other sources of bias	Yes	Potential sources of bias considered

**Table:** Hardigan 2016

***Study characteristics***		
Methods	Random allocation: method unspecified	
Data	Random sample of 7200 US practicing pharmacists	
Comparisons	Shorter vs. longer questionnaire; demographic items first vs. last; postal vs electronic; email invitation to web survey vs. postal ('hybrid') invitation to web survey	
Outcomes	Final response (after 2 reminders)	
Topic	Non‐health (job satisfaction)	
Mode of Administration	Postal and electronic	
Notes	Longer questionnaire was 60 questions and 4 pages; shorter questionnaire was 36 questions and 2 pages. Pharmacists in the postal group were sent an envelope containing a letter describing the study, a questionnaire, an SAE, and a refusal postcard. If a response was not received within 2 weeks after the first mailing, another questionnaire was sent by mail. If no response was received 2 weeks after the second mailing, a reminder postcard was mailed. Pharmacists in the email group were sent an email inviting them to partake in the online survey. The message provided a URL directing them to the survey, as well as a refusal link if they preferred not to participate, or be contacted again. If the link to the survey was chosen, the participant was directed to the questionnaire. A response to each question was not required in order for the survey to proceed to the next question. Once the survey was completed, results were stored in a master database. If a response was not received within 2 weeks, a second email message was sent providing a survey link as well as a refusal link. If a response was not received 2 weeks later, a final email reminder was sent. Pharmacists in the hybrid group were mailed a postcard describing the study. This postcard provided a URL directing them to the survey, as well as a refusal link if they preferred not to participate or be contacted again. Once the survey was completed, results were stored in a master database. If a response was not received within 2 weeks after the first mailing, another postcard directing participants to the online survey was sent by mail.	
***Risk of bias***		
**Item**	**Authors' judgement**	**Support for judgement**
Sequence generation	Unclear	Random allocation: method unspecified
Allocation concealment?	Unclear	Random allocation: method unspecified
Blinding of participants and personnel	Unclear	Blinding of personnel not described. Participants were not blinded to intervention.
Blinding of outcome assessment	Yes	No outcome assessment used (counts of responses only)
Incomplete outcome data	Yes	Exclusions reported
Selective reporting	Yes	Outcomes reported in full
Other sources of bias	Yes	No grounds for concern about other sources of bias from reading the study report

**Table:** Hardy 2016

***Study characteristics***		
Methods	Random allocation: computer random number generation	
Data	First‐time mothers who chose to have an epidural, UK	
Comparisons	Conditional non‐monetary incentive (£10 gift voucher) with first mailing vs. with reminder mailings	
Outcomes	Response after one mailing (incentive with first contact group); response after 2 reminders (incentive with reminder group)	
Topic	Health (women's health, well‐being and health service use one year following the birth of their baby)	
Mode of Administration	Postal/electronic	
Notes	12‐page questionnaire. Cover letter included a sentence explaining that the voucher was to thank participants for their time and effort. For the incentive reminder letter group, the cover letter sent at first mailout did not mention the incentive. If the questionnaire was not returned, all reminder letters detailed the promise of a £10 gift voucher on return of a completed questionnaire. For both groups, women were additionally contacted electronically and via text message if the contact details were available.	
***Risk of bias***		
**Item**	**Authors' judgement**	**Support for judgement**
Sequence generation	Yes	Random allocation: computer random number generation
Allocation concealment?	Yes	Random allocation: computer random number generation
Blinding of participants and personnel	No	BUMPES trial staff were aware of the allocation due to the nature of the interventions, and the practicalities involved in sending the letters and the vouchers.
Blinding of outcome assessment	Yes	No outcomes were assessed (counts of responses only).
Incomplete outcome data	Yes	8 exclusions were reported.
Selective reporting	Yes	Outcomes reported in full
Other sources of bias	Yes	No grounds for concern about other sources of bias from reading the study report

**Table:** Harris 1978

***Study characteristics***		
Methods	Random allocation: method not specified	
Data	A randomly selected sample of respondents	
Comparisons	1. Business‐reply return envelope enclosed 2. Stamped‐reply envelope enclosed	
Outcomes	Response within 4 weeks	
Topic	Not specified	
Mode of Administration	Postal	
Notes	Method of allocation confirmed through contact with author	
***Risk of bias***		
**Item**	**Authors' judgement**	**Support for judgement**
Allocation concealment?	Unclear	B ‐ unclear

**Table:** Harris 2008

***Study characteristics***		
Methods	Random allocation: random number tables	
Data	1156 major trauma patients	
Comparisons	Conditional non‐monetary incentive (AUS $4 instant lottery ticket) vs. no incentive	
Outcomes	Response at 4 weeks after initial mailing and after 6 months, at the end of the study	
Topic	Health (outcomes after major trauma)	
Mode of Administration	Postal	
Notes	7‐page questionnaire containing 34 questions pertaining to general health (and possible demographic and socioeconomic predictors of health); took approximately 20 minutes to complete The major prize was AU$50,000 per year, for 20 years.	
***Risk of bias***		
**Item**	**Authors' judgement**	**Support for judgement**
Sequence generation	Yes	Random allocation: random number tables
Allocation concealment?	Yes	Random allocation: random number tables
Blinding of participants and personnel	Unclear	Blinding of personnel not described. Participants were not blinded to intervention.
Blinding of outcome assessment	Yes	No outcomes were assessed (counts of responses only).
Incomplete outcome data	Yes	Exclusions reported
Selective reporting	Yes	Outcomes reported in full
Other sources of bias	Yes	No grounds for concern about other sources of bias from reading the study report

**Table:** Harrison 2002

***Study characteristics***		
Methods	Random allocation: computerised random number generation	
Data	Adults selected from a Health Authority Register (North West England)	
Comparisons	1. Reply envelope with first‐class stamp 2. Pre‐paid business‐franked reply envelope	
Outcomes	—	
Topic	Health: health questionnaire	
Mode of Administration	Postal	
Notes	Age: Mostly 18‐45 years; equal male and females	
***Risk of bias***		
**Item**	**Authors' judgement**	**Support for judgement**
Allocation concealment?	Yes	A ‐ adequate

**Table:** Harrison 2004

***Study characteristics***		
Methods	Random allocation: using random number generation, method unspecified	
Data	Patients referred to a community‐based exercise referral scheme	
Comparisons	1. Pre‐warning letter 2.No pre‐warning letter	
Outcomes	Response rate at 6 weeks	
Topic	Health: quality of services offered at the community‐based referral scheme	
Mode of Administration	Postal	
Notes	—	
***Risk of bias***		
**Item**	**Authors' judgement**	**Support for judgement**
Sequence generation	Unclear	Random allocation: using random number generation, method unspecified
Allocation concealment?	Unclear	Random allocation: using random number generation, method unspecified
Blinding of participants and personnel	Yes	Participants remained unaware as to group allocation.
Blinding of outcome assessment	Yes	No outcomes were assessed (counts of responses only).
Incomplete outcome data	No	Exclusions not reported
Selective reporting	Yes	Response after 6 weeks (2 reminders) reported in full
Other sources of bias	Yes	No grounds for concern about other sources of bias from reading the study report

**Table:** Harvey 1986

***Study characteristics***		
Methods	Random allocation: alternation	
Data	A random sample of people living in the West Midlands, UK, listed on the electoral register	
Comparisons	1. Reply envelope with first‐class stamp 2. Reply envelope with second‐class stamp	
Outcomes	Response period not specified	
Topic	Non‐health: interest in fine art	
Mode of Administration	Postal	
Notes	Method of allocation confirmed through contact with author	
***Risk of bias***		
**Item**	**Authors' judgement**	**Support for judgement**
Allocation concealment?	No	C ‐ inadequate

**Table:** Hatch 2017

***Study characteristics***		
Methods	Random allocation: computer‐generated	
Data	Patients from 26 UK ICUs	
Comparisons	4 pages vs. 8 pages	
Outcomes	First response 3 months after ICU discharge	
Topic	Health (quality of life, the incidence of depression, and the incidence of post‐traumatic stress disorder following at least 24 h of treatment at an ICU)	
Mode of Administration	Postal	
Notes	4 pages vs. 8 pages, all pages were single‐sided and numbered Initial packs sent at 3 months contained a personally‐addressed letter inviting participation, a three‐page study information leaflet and a consent form. All documents prepared using a high‐quality laser printer, with invitation letter on Oxford University‐headed paper. Trial co‐ordinator signed each letter. We printed each questionnaire on different coloured paper and bound them with a removable clip, all pages single‐sided and numbered. Used a uniform design, large font size and generous spacing. All packs contained a freepost return envelope. All packs included an ICON branded pen and tea bag as incentives; the tea bag label invited participants to enjoy a cup of tea whilst completing questionnaire. We gave all patients a letter introducing the study at ICU discharge: it explained that they might receive mail from the study team. Patients were eligible if they received level 3 care (as defined by the Intensive Care Society, London) in an ICU for at least 24 h. We excluded patients if they were under 16 years old. The group A pack contained 4 questionnaire pages (one page EuroQol 5 dimensions‐3 level (EQ‐5D‐3 L) questionnaire, two pages each containing a single visual analogue scale, and a single page demographics questionnaire); the group B pack contained 8 questionnaire pages (the group A pack, plus a two‐page Hospital Anxiety and Depression Scale (HADS) questionnaire and a two‐page Post‐traumatic stress disorder Check List‚ Civilian version (PCL‐C) questionnaire	
***Risk of bias***		
**Item**	**Authors' judgement**	**Support for judgement**
Sequence generation	Yes	Random allocation: computer‐generated
Allocation concealment?	Yes	Random allocation: computer‐generated
Blinding of participants and personnel	Unclear	Blinding of personnel not described. Participants were not blinded to intervention.
Blinding of outcome assessment	Yes	No outcomes were assessed (counts of responses only).
Incomplete outcome data	Yes	Exclusions were reported.
Selective reporting	Yes	Outcomes reported in full
Other sources of bias	Yes	No grounds for concern about other sources of bias from reading the study report

**Table:** Hathaway 2021a

***Study characteristics***		
Methods	Random allocation: method unspecified	
Data	Cancer centre population: participants were those diagnosed with invasive and in situ cancer, benign diseases, and patients who were screened without a cancer diagnosis, US	
Comparisons	Study a: concise vs. wordy text in web survey email	
Outcomes	Response after 2 reminders	
Topic	Health (cancer centre patients experience during COVID‐19 pandemic)	
Mode of Administration	Electronic	
Notes	143 total items across 26 webpages. Email sent with a survey link for each trial condition. Up to two reminder emails were sent in 4‐day intervals.	
***Risk of bias***		
**Item**	**Authors' judgement**	**Support for judgement**
Sequence generation	Unclear	Random allocation: method unspecified
Allocation concealment?	Unclear	Random allocation: method unspecified
Blinding of participants and personnel	Unclear	Blinding of personnel not described. Participants were not blinded to intervention.
Blinding of outcome assessment	Yes	No outcomes were assessed (counts of responses only).
Incomplete outcome data	No	Exclusions/attrition not reported
Selective reporting	Yes	Response after 2 reminders reported in full
Other sources of bias	Yes	No grounds for concern about other sources of bias from reading the study report

**Table:** Hathaway 2021b

***Study characteristics***		
Methods	Random allocation: method unspecified	
Data	Cancer centre population: participants were those diagnosed with invasive and in situ cancer, benign diseases, and patients who were screened without a cancer diagnosis, US	
Comparisons	Study b: unconditional non‐monetary incentive vs. no incentive	
Outcomes	Response after 2 reminders	
Topic	Health (cancer centre patients experience during COVID‐19 pandemic)	
Mode of Administration	Electronic	
Notes	143 total items across 26 webpages. Email sent with a survey link for each trial condition. Up to two reminder emails were sent in 4‐day intervals.	
***Risk of bias***		
**Item**	**Authors' judgement**	**Support for judgement**
Sequence generation	Unclear	Random allocation: method unspecified
Allocation concealment?	Unclear	Random allocation: method unspecified
Blinding of participants and personnel	Unclear	Blinding of personnel not described. Participants were not blinded to intervention.
Blinding of outcome assessment	Yes	No outcomes were assessed (counts of responses only).
Incomplete outcome data	No	Exclusions/attrition not reported
Selective reporting	Yes	Response after 2 reminders reported in full
Other sources of bias	Yes	No grounds for concern about other sources of bias from reading the study report

**Table:** Hathaway 2021c

***Study characteristics***		
Methods	Random allocation: method unspecified	
Data	Cancer centre population: participants were those diagnosed with invasive and in situ cancer, benign diseases, and patients who were screened without a cancer diagnosis, US	
Comparisons	Study c: conditional non‐monetary incentive vs. no incentive	
Outcomes	Response after 2 reminders	
Topic	Health (cancer centre patients experience during COVID‐19 pandemic)	
Mode of Administration	Electronic	
Notes	143 total items across 26 webpages. Email sent with a survey link for each trial condition. Up to two reminder emails were sent in 4‐day intervals.	
***Risk of bias***		
**Item**	**Authors' judgement**	**Support for judgement**
Sequence generation	Unclear	Random allocation: method unspecified
Allocation concealment?	Unclear	Random allocation: method unspecified
Blinding of participants and personnel	Unclear	Blinding of personnel not described. Participants were not blinded to intervention.
Blinding of outcome assessment	Yes	No outcomes were assessed (counts of responses only).
Incomplete outcome data	No	Exclusions/attrition not reported
Selective reporting	Yes	Response after 2 reminders reported in full
Other sources of bias	Yes	No grounds for concern about other sources of bias from reading the study report

**Table:** Hathaway 2021d

***Study characteristics***		
Methods	Random allocation: method unspecified	
Data	Cancer centre population: participants were those diagnosed with invasive and in situ cancer, benign diseases, and patients who were screened without a cancer diagnosis, US	
Comparisons	[Study d: mailed pre‐notification letter vs. pre‐notification postcard	
Outcomes	Response after 2 reminders	
Topic	Health (cancer centre patients experience during COVID‐19 pandemic)	
Mode of Administration	Electronic	
Notes	143 total items across 26 webpages. Email sent with a survey link for each trial condition. Up to two reminder emails were sent in 4‐day intervals.	
***Risk of bias***		
**Item**	**Authors' judgement**	**Support for judgement**
Sequence generation	Unclear	Random allocation: method unspecified
Allocation concealment?	Unclear	Random allocation: method unspecified
Blinding of participants and personnel	Unclear	Blinding of personnel not described. Participants were not blinded to intervention.
Blinding of outcome assessment	Yes	No outcomes were assessed (counts of responses only).
Incomplete outcome data	No	Exclusions/attrition not reported
Selective reporting	Yes	Response after 2 reminders reported in full
Other sources of bias	Yes	No grounds for concern about other sources of bias from reading the study report

**Table:** Hauw‐Berlemont 2020

***Study characteristics***		
Methods	Random allocation: method unspecified	
Data	ICU providers (physicians and advanced practice providers) at a tertiary academic centre, NY, US	
Comparisons	Survey sent from more senior person (medical director) vs. sent from less senior (hospital administrator)	
Outcomes	Response after 3 reminders (6 weeks)	
Topic	Health (Readiness‐for‐change survey for ICU providers)	
Mode of Administration	Electronic	
Notes	Questionnaire comprised 30 items; 5 minutes to complete The first email and subsequent reminders were sent to each randomised group at the same time. Access to the survey was available for 57 days: three reminders were sent on days 15, 32, and 46.	
***Risk of bias***		
**Item**	**Authors' judgement**	**Support for judgement**
Sequence generation	Unclear	Random allocation: method unspecified
Allocation concealment?	Unclear	Random allocation: method unspecified
Blinding of participants and personnel	Unclear	Blinding of personnel not described. Participants were not blinded to intervention.
Blinding of outcome assessment	Yes	No outcomes were assessed (counts of responses only).
Incomplete outcome data	No	Exclusions not reported
Selective reporting	Yes	Response after 3 reminders (6 weeks) reported in full
Other sources of bias	Yes	No grounds for concern about other sources of bias from reading the study report

**Table:** Hawkins 1979

***Study characteristics***		
Methods	Random allocation: method not specified	
Data	Eugene residences listed in the Eugene‐Springfield telephone directory	
Comparisons	1. Department store sponsor; standard 2. Department store sponsor; disclosure 3. Research firm sponsor; standard 4. Research firm sponsor; disclosure 5. University sponsor, standard 6. University sponsor, disclosure	
Outcomes	Response period not specified	
Topic	Non‐health: women's attitudes to shopping, demographics	
Mode of Administration	Postal	
Notes	—	
***Risk of bias***		
**Item**	**Authors' judgement**	**Support for judgement**
Allocation concealment?	Unclear	B ‐ unclear

**Table:** Hawley 2009

***Study characteristics***		
Methods	Random allocation: method unspecified	
Data	Mental health providers, US	
Comparisons	No incentive vs. unconditional non‐monetary incentive (therapy magnet) vs. unconditional $1 vs. unconditional $2 vs. unconditional $5	
Outcomes	Response after 2 reminders	
Topic	Health (mental health assessment and treatment practices)	
Mode of Administration	Postal	
Notes	7‐page questionnaires. Personally addressed and hand‐signed cover letters with first‐class stamps on both the individually addressed outgoing envelope and the return envelope, photographs of children to increase salience and help the survey stand out from other mail, user‐friendly design (e.g. important words were bolded or italicised and each section of the survey was grouped using borders; return address information was placed on both the front and back cover of the survey), two follow‐up mailings including a thank you/reminder postcard sent to the entire sample and a 2nd survey sent to non‐respondents, both of which were also personally addressed and hand‐signed. In addition, a separate postage‐paid postcard was included that respondents could return with their completed address if they wished to receive a summary of the survey findings.	
***Risk of bias***		
**Item**	**Authors' judgement**	**Support for judgement**
Sequence generation	Unclear	Random allocation: method unspecified
Allocation concealment?	Unclear	Random allocation: method unspecified
Blinding of participants and personnel	Unclear	Blinding of personnel not described. Participants were not blinded to intervention.
Blinding of outcome assessment	Yes	No outcomes were assessed (counts of responses only).
Incomplete outcome data	Yes	6 surveys were undeliverable.
Selective reporting	Yes	Response after 2 reminders reported in full
Other sources of bias	Yes	No grounds for concern about other sources of bias from reading the study report

**Table:** Heaton 1965

***Study characteristics***		
Methods	Random allocation: method not specified	
Data	Individuals living in the Philadelphia, Pennsylvania area who had purchased a new 1959 Chevrolet within the previous 12‐16 weeks	
Comparisons	1. Preliminary letter 2. No preliminary letter	
Outcomes	Response within 2 weeks	
Topic	Non‐health: information on automobile ownership, shopping behaviour	
Mode of Administration	Postal	
Notes	—	
***Risk of bias***		
**Item**	**Authors' judgement**	**Support for judgement**
Allocation concealment?	Unclear	B ‐ unclear

**Table:** Heerwegh 2005a

***Study characteristics***		
Methods	Random allocation: using SAS RANUNI function	
Data	1st year students at the Katholieke Universiteit Leuven, Belgium	
Comparisons	1. Personalised salutations 2. Non‐personalised salutations	
Outcomes	Response period not specified	
Topic	Non‐health: adolescents' attitudes towards marriage and divorce	
Mode of Administration	Electronic: Web survey	
Notes	Method of allocation ascertained through contact with author; age: 17‐20 years	
***Risk of bias***		
**Item**	**Authors' judgement**	**Support for judgement**
Allocation concealment?	Yes	A ‐ adequate

**Table:** Heerwegh 2005b

***Study characteristics***		
Methods	Random allocation: using SAS RANUNI function	
Data	1st year students at the Katholieke Universiteit Leuven, Belgium	
Comparisons	1. Personalised salutations 2. Non‐personalised salutations	
Outcomes	Response period not specified	
Topic	Non‐health: adolescents' attitudes towards marriage and divorce	
Mode of Administration	Electronic: Web survey	
Notes	Method of allocation ascertained through contact with author	
***Risk of bias***		
**Item**	**Authors' judgement**	**Support for judgement**
Allocation concealment?	Yes	A ‐ adequate

**Table:** Heerwegh 2006

***Study characteristics***		
Methods	Random allocation: using SAS RANUNI function	
Data	Freshmen at the Katholieke Universiteit Leuven, Belgium	
Comparisons	1. Personalised salutations 2. Non‐personalised salutations	
Outcomes	Response period not specified	
Topic	Non‐health: attitudes towards immigrants and asylum seekers	
Mode of Administration	Electronic: Web survey	
Notes	Method of allocation ascertained through contact with author	
***Risk of bias***		
**Item**	**Authors' judgement**	**Support for judgement**
Allocation concealment?	Yes	A ‐ adequate

**Table:** Hendrick 1972

***Study characteristics***		
Methods	Random allocation: method not specified	
Data	Individuals listed in the city directory (Akron, Ohio)	
Comparisons	1. 1‐page questionnaire; solicitor ingratiate (SI); respondent ingratiate (RI) 2. 1‐page questionnaire, SI; respondent no ingratiate (RNI) 3. 1‐page questionnaire; solicitor no ingratiate (SNI); RI 4. 1‐page questionnaire; SNI; RNI 5. 7‐page questionnaire; SI; RI 6. 7‐page questionnaire; SI; RNI 7. 7‐page questionnaire; SNI; RI 8. 7‐page questionnaire; SNI; RNI	
Outcomes	—	
Topic	Health: repression ‐ sensitisation personality scale	
Mode of Administration	Postal	
Notes	—	
***Risk of bias***		
**Item**	**Authors' judgement**	**Support for judgement**
Allocation concealment?	Unclear	B ‐ unclear

**Table:** Hendriks 2001

***Study characteristics***		
Methods	Random allocation: alternation	
Data	784 consecutively discharged patients from 8 randomly chosen hospital wards at the Academic Medical Center, Amsterdam	
Comparisons	1. 10‐step evaluation scale (E10) 2. 5‐step evaluation, tick box scale (E5‐B) 3. 5‐step evaluation, circle answer scale (E5‐W) 4. 5‐step satisfaction, tick box scale (S5‐B) 5. 5‐step satisfaction, circle answer scale (S5‐W)	
Outcomes	Response period not specified	
Topic	Health: satisfaction with hospital care	
Mode of Administration	Postal	
Notes	—	
***Risk of bias***		
**Item**	**Authors' judgement**	**Support for judgement**
Allocation concealment?	No	C ‐ inadequate

**Table:** Henley 1976

***Study characteristics***		
Methods	Random allocation: method not specified	
Data	Residents of Fort Worth, Texas	
Comparisons	1. 1 by 4‐inch slip of paper saying 'Please return by April 7th' stapled to the questionnaire 2. No deadline slip	
Outcomes	Response within 14 days	
Topic	Non‐health: civil issues	
Mode of Administration	Postal	
Notes	—	
***Risk of bias***		
**Item**	**Authors' judgement**	**Support for judgement**
Allocation concealment?	Unclear	B ‐ unclear

**Table:** Hensley 1974

***Study characteristics***		
Methods	Random allocation: alternation	
Data	Members of the National Forensic League	
Comparisons	1. Outer‐envelope (OE) commemorative Inner‐envelope (IE) commemorative 2. OE commemorative; IE regular 3. OE commemorative; IE metered 4. OE regular; IE commemorative 5. OE regular; IE regular 6. OE regular; IE metered 7. OE metered; IE commemorative 8. OE metered; IE regular 9. OE metered; IE metered	
Outcomes	Response period not specified	
Topic	Non‐health	
Mode of Administration	Postal	
Notes	—	
***Risk of bias***		
**Item**	**Authors' judgement**	**Support for judgement**
Allocation concealment?	No	C ‐ inadequate

**Table:** Hewett 1974

***Study characteristics***		
Methods	Random allocation: coin toss	
Data	Individuals listed in a telephone directory	
Comparisons	1. Hand‐stamped outgoing envelope; hand‐stamped return envelope 2. Hand‐stamped outgoing envelope; first‐class postal permit business‐reply envelope	
Outcomes	Response period not specified	
Topic	Not specified	
Mode of Administration	Postal	
Notes	—	
***Risk of bias***		
**Item**	**Authors' judgement**	**Support for judgement**
Allocation concealment?	No	C ‐ inadequate

**Table:** Hickey 2021

***Study characteristics***		
Methods	Random allocation: computer‐generated randomisation	
Data	A random sample of 500 emergency physicians in Canada	
Comparisons	Postal pre‐notification letter vs. no pre‐notification letter	
Outcomes	Response after 6 weeks (4 reminders)	
Topic	Health (attitudes regarding organ donation)	
Mode of Administration	Postal	
Notes	24 questions; 4 sections; double‐sided; 2 sheets; demographic and practice information, attitudes regarding organ donation, acceptability of using the emergency department to promote organ donation and registration and related perceived facilitators and barriers All physicians received an unconditional incentive of a $3 coffee card and an SAE with the survey. In both groups, non‐respondents were sent reminder surveys approximately every 2 weeks for a total of 6 weeks and a special contact using Xpress post during the final contact attempt. Pre‐notification letters were hand‐signed by the principal investigator and sent approximately 1 week prior to the first questionnaire mailout.	
***Risk of bias***		
**Item**	**Authors' judgement**	**Support for judgement**
Sequence generation	Yes	Random allocation: computer‐generated randomisation
Allocation concealment?	Yes	Random allocation: computer‐generated randomisation
Blinding of participants and personnel	Yes	Personnel were blind but participants were not.
Blinding of outcome assessment	Yes	Using blinded outcome assessment, physician responses were analysed.
Incomplete outcome data	Yes	Exclusions were reported.
Selective reporting	Yes	Response after 6 weeks (4 reminders) reported in full
Other sources of bias	Yes	No grounds for concern about other sources of bias from reading the study report

**Table:** Hoffman 1998

***Study characteristics***		
Methods	Random allocation: using terminal digit of study number or house number	
Data	Individuals who had previously participated in a campaign to collect blood for a specimen bank	
Comparisons	Study 1: 1. Short questionnaire 2. Long questionnaire Study 2: 1. No incentive 2. Newspaper article 3. Pencil 4. Pencil and newspaper article Study 3: 1. Postcard reminder 2. Second questionnaire and letter	
Outcomes	—	
Topic	Health: Family history of cancer, reproductive history, medical and vitamin use, history of medical conditions and surgery	
Mode of Administration	Postal	
Notes	—	
***Risk of bias***		
**Item**	**Authors' judgement**	**Support for judgement**
Allocation concealment?	Unclear	B ‐ unclear

**Table:** Hohwu 2013

***Study characteristics***		
Methods	Random allocation: method unspecified	
Data	Random allocation: method unspecified	
Comparisons	Non‐monetary incentive vs. no incentive (Web/tablet) vs. (Web); electronic vs choice (postal/electronic) (Web) vs. (paper/Web); postal vs choice (postal/electronic) (paper) vs. (paper/Web)	
Outcomes	Response after 2 reminders	
Topic	Health (children's health and welfare)	
Mode of Administration	Postal/electronic	
Notes	NordChild questionnaire, 73 questions with sub‐questions; the paper version was 28 pages long. All 3200 were invited by mail. Two reminders were mailed to all those who had not responded within 4 and 12 weeks. The Web‐based questionnaire was a multi‐page design using SurveyXact and had the same questions as the paper version. The respondents of the Web‐based questionnaire could answer the questions in several rounds, and submit it after the last question. A 12‐character log‐in code to the Web‐based questionnaire had to be keyed in every time if it was not completed in a single round.	
***Risk of bias***		
**Item**	**Authors' judgement**	**Support for judgement**
Sequence generation	Unclear	Random allocation: method unspecified
Allocation concealment?	Unclear	Random allocation: method unspecified
Blinding of participants and personnel	Unclear	Blinding of personnel not described. Participants were not blinded to intervention.
Blinding of outcome assessment	Yes	No outcomes were assessed (counts of responses only).
Incomplete outcome data	Yes	Exclusions were reported.
Selective reporting	Yes	Response after 2 reminders reported in full
Other sources of bias	Yes	No grounds for concern about other sources of bias from reading the study report

**Table:** Hopkins 1988

***Study characteristics***		
Methods	Random allocation: method not specified	
Data	Professional school and public librarians	
Comparisons	1. $1 incentive 2. No incentive Non‐responders followed‐up after 1 month	
Outcomes	—	
Topic	Non‐health: attitudes and practices having a book in Spanish in the library	
Mode of Administration	Postal	
Notes	—	
***Risk of bias***		
**Item**	**Authors' judgement**	**Support for judgement**
Allocation concealment?	Unclear	B ‐ unclear

**Table:** Horn 2010

***Study characteristics***		
Methods	Random allocation: method not specified	
Data	Parents and carers of children and adolescents involved with CAMHS, Kent, UK	
Comparisons	No reminders vs. reminder letter with questionnaire vs. reminder letter with questionnaire plus telephone call to non‐responders 2 weeks after postal reminder	
Outcomes	Final response	
Topic	Health (experience of Service Questionnaire with Strengths and Difficulties Questionnaire)	
Mode of Administration	Postal	
Notes	Strengths and Difficulties Questionnaire (SDQ) and Experience of Service Questionnaire (ESQ). Package not described. The experimental interventions were randomly allocated to three Community Child and Adolescent Mental Health Services (CAMHS) teams. These teams were configured to have a broadly equivalent staff mix of mental health nurses, psychologists, psychiatrists and other therapists, weighted in proportion to the population and referrals to each team. CAMHS mapping returns suggest that the teams were not markedly different in the crude case‐mix referred, although Team A served a less deprived population.	
***Risk of bias***		
**Item**	**Authors' judgement**	**Support for judgement**
Sequence generation	Unclear	Random allocation: method not specified
Allocation concealment?	Unclear	Random allocation: method not specified
Blinding of participants and personnel	Unclear	Blinding of personnel not described. Participants were not blinded to intervention.
Blinding of outcome assessment	Yes	No outcomes were assessed (counts of responses only).
Incomplete outcome data	Yes	Exclusions were reported.
Selective reporting	Yes	Final response reported in full
Other sources of bias	No	Randomisation was clustered at CAMHS‐team level, with some differences between populations served that may have been associated with questionnaire response.

**Table:** Hornik 1981

***Study characteristics***		
Methods	Random allocation: method not specified	
Data	Individuals listed in a telephone directory (Chicago, US)	
Comparisons	1. Given time cue of 20 mins 2. Given time cue of 40 mins 3. Not given time cue	
Outcomes	Response within 4 weeks	
Topic	Non‐health: attitudes to TV advertising	
Mode of Administration	Postal	
Notes	—	
***Risk of bias***		
**Item**	**Authors' judgement**	**Support for judgement**
Allocation concealment?	Unclear	B ‐ unclear

**Table:** Hornik 1982

***Study characteristics***		
Methods	Random allocation: method not specified	
Data	Individuals listed in a telephone directory (Chicago, US)	
Comparisons	1. Ingratiation appeal made in pre‐notification telephone call (IA) ‐ male telephone pre‐notified to male respondent (M/M) 2. IA ‐ M/F 3. IA ‐ F/M 4. IA ‐ F/F 5. Polite imperative (PI) ‐ M/M 6. PI ‐ M/F 7. PI ‐ F/M 8. PI ‐ F/F 9. Rhetorical question (RQ) ‐ M/M 10. RQ ‐ M/F 11. RQ ‐ F/M 12. RQ ‐ F/F 13. Statement (S) ‐ M/M 14. S ‐ M/F 15. S ‐ F/M 16. S ‐ F/F 17. No pre‐notification	
Outcomes	—	
Topic	Non‐health: people's attitudes to television and advertising	
Mode of Administration	Postal	
Notes	—	
***Risk of bias***		
**Item**	**Authors' judgement**	**Support for judgement**
Allocation concealment?	Unclear	B ‐ unclear

**Table:** Horowitz 1974

***Study characteristics***		
Methods	Random allocation: method not specified	
Data	Faculty members of the University of Maryland (US)	
Comparisons	1. Status of researcher 'professor' (P); ink Signature (I); reproduction photocopied (R‐P) 2. Graduate student (GS); I; R‐P 3. P; non‐Ink (N‐I); R‐P 4. GS; N‐I; R‐P 5. P; I; Reproduction mimeographed (R‐M) 6. GS; I; R‐M 7. P; N‐I; R‐M 8. GS; N‐I; R‐M 9. P; I; R‐T 10. GS; I; R‐T	
Outcomes	Response period not specified	
Topic	Non‐health: College professors Questionnaire	
Mode of Administration	Postal	
Notes	—	
***Risk of bias***		
**Item**	**Authors' judgement**	**Support for judgement**
Allocation concealment?	Unclear	B ‐ unclear

**Table:** Houston 1975

***Study characteristics***		
Methods	Random allocation: method not specified	
Data	New car buyers in Scott County, Iowa (US)	
Comparisons	1. Personalised letter and questionnaire; ballpoint pen incentive 2. Personalised letter and questionnaire; no incentive 3. Non‐personalised; ballpoint pen incentive 4. Non‐personalised; no incentive	
Outcomes	—	
Topic	Non‐health: sources of information used by the respondent in purchasing their new car	
Mode of Administration	Postal	
Notes	—	
***Risk of bias***		
**Item**	**Authors' judgement**	**Support for judgement**
Allocation concealment?	Unclear	B ‐ unclear

**Table:** Houston 1977

***Study characteristics***		
Methods	Random allocation: method not specified	
Data	Households listed in a telephone directory (Madison)	
Comparisons	1. University sponsor; Social Utility Appeal 2. Commercial sponsor, Social Utility Appeal 3. University sponsor, Help the Sponsor Appeal 4. Commercial sponsor, Help the Sponsor Appeal 5. University sponsor, Egoistic Appeal 6. Commercial sponsor, Egoistic Appeal 7. University sponsor, Combined Appeal 8. Commercial sponsor, Combined Appeal	
Outcomes	Response within 1 month	
Topic	Non‐health: tap consumer images and behaviour with respect to 5 Madison‐area shopping	
Mode of Administration	Postal	
Notes	—	
***Risk of bias***		
**Item**	**Authors' judgement**	**Support for judgement**
Allocation concealment?	Unclear	B ‐ unclear

**Table:** Hubbard 1988a

***Study characteristics***		
Methods	Random allocation: method not specified	
Data	Residents of a major midwestern metropolitan area	
Comparisons	1. No incentive 2. Promise of $1 donation to charity of respondent's choice 3. 25 cents cash enclosed 4. $1 cash enclosed 5. Opportunity to win $200 cash prize	
Outcomes	Response within 3 weeks	
Topic	Non‐health: satisfaction with banking and financial services	
Mode of Administration	Postal	
Notes	—	
***Risk of bias***		
**Item**	**Authors' judgement**	**Support for judgement**
Allocation concealment?	Unclear	B ‐ unclear

**Table:** Hubbard 1988b

***Study characteristics***		
Methods	Random allocation: method not specified	
Data	Residents of a major midwestern metropolitan area	
Comparisons	1. Control 2. Pre‐paid 25 cent incentive 3. Pre‐paid $1 incentive 4. Opportunity to win cash prize of $50 5. Opportunity to win cash prize of $100 6. Opportunity to win cash prize of $150 7. Opportunity to win cash prize of $200 8. Opportunity to win cash prize of $250	
Outcomes	Response within 3 weeks	
Topic	Non‐health: satisfaction with banking and financial services	
Mode of Administration	Postal	
Notes	—	
***Risk of bias***		
**Item**	**Authors' judgement**	**Support for judgement**
Allocation concealment?	Unclear	B ‐ unclear

**Table:** Huck 1974

***Study characteristics***		
Methods	Random allocation: method not specified	
Data	Students living in residence halls at the University of Tennessee	
Comparisons	1. First mailing with a 25 cent incentive 2. Second mailing (to non‐respondents) with a 25 cent incentive 3. Third mailing (to non‐respondents) with a 25 cent incentive 4. First, second and third mailings without a 25 cent incentive	
Outcomes	Response within 2 weeks	
Topic	Non‐health: Rokeach Dogmatism scale	
Mode of Administration	Postal	
Notes	—	
***Risk of bias***		
**Item**	**Authors' judgement**	**Support for judgement**
Allocation concealment?	Unclear	B ‐ unclear

**Table:** Hyett 1977

***Study characteristics***		
Methods	Random allocation: method not specified	
Data	Residential telephone subscribers	
Comparisons	1. Double‐sided questionnaire 2. Single‐sided questionnaire After 2 weeks, all non‐responders received another questionnaire. 1 week later, all those who still had not responded were followed up by telephone.	
Outcomes	—	
Topic	Non‐health: not specified	
Mode of Administration	Postal	
Notes	—	
***Risk of bias***		
**Item**	**Authors' judgement**	**Support for judgement**
Allocation concealment?	Unclear	B ‐ unclear

**Table:** Iglesias 2000

***Study characteristics***		
Methods	Random allocation: alternation	
Data	Women aged 70 years and over	
Comparisons	1. 4‐page questionnaire 2. 5‐page questionnaire 3. 7‐page questionnaire	
Outcomes	—	
Topic	Health: clinical questionnaire, EuroQoL, SF‐12	
Mode of Administration	Postal	
Notes	Age: Above 70 years; mainly females	
***Risk of bias***		
**Item**	**Authors' judgement**	**Support for judgement**
Allocation concealment?	No	C ‐ inadequate

**Table:** Iglesias 2001

***Study characteristics***		
Methods	Random allocation: alternation	
Data	Women aged 70 years or over selected from 2 general practices in North Yorkshire	
Comparisons	1. Questionnaire with an individual item format 2. Questionnaire with a stem & leaf format	
Outcomes	Response period not specified	
Topic	Health: SF‐12	
Mode of Administration	Postal.	
Notes	Age: Above 70 years; mainly females	
***Risk of bias***		
**Item**	**Authors' judgement**	**Support for judgement**
Allocation concealment?	No	C ‐ inadequate

**Table:** Iversen 2020

***Study characteristics***		
Methods	Random allocation: method unspecified	
Data	Patients aged 16 years and older registered with a GP, Norway	
Comparisons	A pen‐and‐paper questionnaire with the option to answer electronically (mixed) vs. purely electronic protocol	
Outcomes	Response after 2 reminders (6 weeks)	
Topic	Health (user experiences with healthcare).	
Mode of Administration	Postal or electronic	
Notes	Norwegian Patient Experiences with General Practitioner Questionnaire; 47 questions on 6 pages. Additional page was included to allow comments. Mailed invitation with both pen‐and‐paper and electronic response options. The invitation included a cover letter describing the purpose of the study, a paper questionnaire, an SAE, and information and a login code to be able to respond electronically. The patients in Group B received an email invitation with an electronic response option only. The email invitation included information about the purpose of the study, a link to the online survey and a login code. Two reminders were sent to non‐respondents in both samples using the same contact mode as the first invitation. The first reminder was sent to both groups around 3 weeks after the first contact. The second reminder was sent around 6 weeks after the first contact. All reminders to Group A were sent by mail and included a new invitation, the paper questionnaire, the SAE and the login code to enable electronic responses. Group B were sent a new email invitation with a link to the survey and a login code in both reminders.	
***Risk of bias***		
**Item**	**Authors' judgement**	**Support for judgement**
Sequence generation	Unclear	Random allocation: method unspecified
Allocation concealment?	Unclear	Random allocation: method unspecified
Blinding of participants and personnel	Unclear	Blinding of personnel not described. Participants were not blinded to intervention.
Blinding of outcome assessment	Yes	No outcomes were assessed (counts of responses only).
Incomplete outcome data	Yes	Exclusions were reported.
Selective reporting	Yes	Response after 2 reminders (6 weeks) reported in full
Other sources of bias	Yes	No grounds for concern about other sources of bias from reading the study report

**Table:** Jacob 2012

***Study characteristics***		
Methods	Random allocation: assigned a random number to all eligible principals in each strata and then sorted by random number within strata and assigned each quarter to different strata. Randomisation method unclear	
Data	High school principals, Michigan, US	
Comparisons	Email pre‐notification letter vs. paper pre‐notification letter; unconditional $10 monetary incentive vs. no incentive; electronic vs. postal	
Outcomes	Response after 2 reminders (2 weeks)	
Topic	Non‐health (online learning and virtual education)	
Mode of Administration	Electronic (web‐based) or postal	
Notes	42 questions; 15 mins completion time All four groups were sent a copy of the advance letter (either by mail or email). Surveys sent between 1 (postal) and 2 (email) weeks later. Both groups then received two follow‐up emails/postcards reminding them of the incentive (if they received one) that had been provided and asking them to return the questionnaire. We designed the format of the web survey to match the format of the paper survey as closely as possible; and both versions had identical questions.	
***Risk of bias***		
**Item**	**Authors' judgement**	**Support for judgement**
Sequence generation	Unclear	Random allocation: method unclear
Allocation concealment?	Unclear	Random allocation: method unclear
Blinding of participants and personnel	Unclear	Blinding of personnel not described. Participants were not blinded to intervention.
Blinding of outcome assessment	Yes	No outcomes were assessed (counts of responses only).
Incomplete outcome data	Yes	Exclusions (ineligible and undeliverable) reported in full
Selective reporting	Yes	Response after 2 reminders (2 weeks) reported in full
Other sources of bias	Yes	No grounds for concern about other sources of bias from reading the study report

**Table:** Jacobs 1986

***Study characteristics***		
Methods	Random allocation: method not specified	
Data	Public school teachers (Indiana, US)	
Comparisons	1. Short questionnaire; optical scan form 2. Short questionnaire; instructed to respond directly to questionnaire 3. Long questionnaire; optical scan form 4. Long questionnaire; instructed to respond directly to questionnaire Postcard follow up after 2 weeks	
Outcomes	Response period not specified	
Topic	Non‐health: attitudes and opinions concerning discipline in the public schools	
Mode of Administration	Postal	
Notes	—	
***Risk of bias***		
**Item**	**Authors' judgement**	**Support for judgement**
Allocation concealment?	Unclear	B ‐ unclear

**Table:** Jacoby 1990

***Study characteristics***		
Methods	Random allocation: method not specified	
Data	Individuals from 10 parliamentary areas (ISSMC questionnaires) Individuals from the electoral register (FPC questionnaires)	
Comparisons	1. Long questionnaire; sent by ISSMC 2. Long questionnaire; sent by FPC 3. Short questionnaire; sent by ISSMC 4. Short questionnaire; sent by FPC 1. Questionnaire included sensitive question; sent by ISSMC 2. Questionnaire did not include sensitive question; sent by ISSMC 3. Questionnaire included sensitive question; sent by FPC 4. Questionnaire did not include sensitive question; sent by FPC	
Outcomes	—	
Topic	Not specified	
Mode of Administration	Postal	
Notes	—	
***Risk of bias***		
**Item**	**Authors' judgement**	**Support for judgement**
Allocation concealment?	Unclear	B ‐ unclear

**Table:** James 1990a

***Study characteristics***		
Methods	Random allocation: method not specified	
Data	Cable television subscribers (Fairfax County, Virginia, US)	
Comparisons	1. No incentive 2. $0.25 3. $50 4. $1 5. $2 3 follow‐up reminders sent without further monetary incentive at 3‐week intervals	
Outcomes	—	
Topic	Non‐health: personal information	
Mode of Administration	Postal	
Notes	Author contacted: on the third follow up, participants were randomised to receive the questionnaire by first‐class or certified mailing but no data given for results.	
***Risk of bias***		
**Item**	**Authors' judgement**	**Support for judgement**
Allocation concealment?	Unclear	B ‐ unclear

**Table:** James 1990b

***Study characteristics***		
Methods	Random allocation: method not specified	
Data	Cable television subscribers who had failed to respond to 2 previous follow‐up attempts	
Comparisons	1. Reminder by certified mail 2. Reminder by first‐class mail	
Outcomes	Response period not specified	
Topic	Non‐health: personal information	
Mode of Administration	Postal	
Notes	—	
***Risk of bias***		
**Item**	**Authors' judgement**	**Support for judgement**
Allocation concealment?	Unclear	B ‐ unclear

**Table:** James 1992

***Study characteristics***		
Methods	Random allocation: method not specified	
Data	Members of a national trade association of owners of construction subcontracting companies who were not currently enrolled in the association's health insurance programme	
Comparisons	1. No incentive 2. $1 cash 3. $5 cash 4. $5 cheque 5. $10 cheque 6. $20 cheque 7. $40 cheque 8. Promise of $50 1‐page questionnaire, cover letter and business reply envelope. Reminders sent to non‐respondents at 3‐week intervals	
Outcomes	—	
Topic	Health: health insurance	
Mode of Administration	Postal	
Notes	—	
***Risk of bias***		
**Item**	**Authors' judgement**	**Support for judgement**
Allocation concealment?	Unclear	B ‐ unclear

**Table:** James 2011

***Study characteristics***		
Methods	Random allocation: method unspecified	
Data	1154 US physicians who had not responded to two waves of a survey	
Comparisons	Monetary incentive vs. non‐monetary incentive; unconditional vs. conditional non‐monetary incentive ($25 cheque); cheque not requiring social security number (SSN) vs. cheque requiring SSN	
Outcomes	Final response	
Topic	Health (ethical and moral beliefs of physicians)	
Mode of Administration	Postal	
Notes	8‐page questionnaire. The mailing of all survey materials in the third wave was co‐ordinated and carried out by our external vendor. Physicians in all four groups received a cover letter printed on Mayo Clinic letterhead signed by the principal investigator; the survey; and a stamped, pre‐addressed return envelope that routed all completed surveys to the Mayo Clinic Survey Research Center. To avoid institutional constraints, we contracted with an external vendor to provide the cash, immediate checks, and promised checks that did not require an SSN. The promised checks requiring an SSN were processed and disbursed by the Mayo Clinic accounting department.	
***Risk of bias***		
**Item**	**Authors' judgement**	**Support for judgement**
Sequence generation	Unclear	Random allocation: method unspecified
Allocation concealment?	Unclear	Random allocation: method unspecified
Blinding of participants and personnel	Yes	The mailing of all survey materials in the third wave was co‐ordinated and carried out by our external vendor.
Blinding of outcome assessment	Yes	No outcomes were assessed (counts of responses only).
Incomplete outcome data	Yes	Exclusions were reported.
Selective reporting	Yes	Final response reported in full
Other sources of bias	Yes	No grounds for concern about other sources of bias from reading the study report

**Table:** James 2019

***Study characteristics***		
Methods	Random allocation: generated using STATA	
Data	Participants in a trial of occupational therapist‐led home assessment and modification for the prevention of falls (aged over 65 years at risk of falling)	
Comparisons	Pen vs. no pen Standard letter vs. letter emphasising to the participant that their questionnaire responses were noted (social incentive text cover letter)	
Outcomes	First response	
Topic	Health (falls in the elderly)	
Mode of Administration	Postal	
Notes	Questionnaire not described. An unconditional £5 note was included with the questionnaire for all participants.	
***Risk of bias***		
**Item**	**Authors' judgement**	**Support for judgement**
Sequence generation	Yes	Random allocation: generated using STATA
Allocation concealment?	Yes	Random allocation: generated using STATA
Blinding of participants and personnel	Yes	Participants were blind to their participation. Research administrators and research team members posting the questionnaire packs were not blind to the intervention; however, administrators who recorded the outcome data were blind to allocation.
Blinding of outcome assessment	Yes	No outcomes were assessed (counts of responses only).
Incomplete outcome data	Yes	Exclusions were reported.
Selective reporting	Yes	First response reported in full
Other sources of bias	Yes	No grounds for concern about other sources of bias from reading the study report

**Table:** Jamtvedt 2008

***Study characteristics***		
Methods	Random allocation: block randomisation by computer‐generated table	
Data	Norwegian physiotherapists from private practice	
Comparisons	1. Dark chocolate 2. Control group	
Outcomes	Response period within 9 months	
Topic	Health: treatment provided to 1 patient with osteoarthritis of knee through 12 treatment sessions	
Mode of Administration	Postal	
Notes	Randomisation was generated by Doris Tove Kristoffersen, who was not involved with any other aspect of the trial.	
***Risk of bias***		
**Item**	**Authors' judgement**	**Support for judgement**
Sequence generation	Yes	Random allocation: computer‐generated
Allocation concealment?	Yes	Random allocation: computer‐generated
Blinding of participants and personnel	Unclear	Blinding of personnel not described. Participants were not blinded to intervention.
Blinding of outcome assessment	Yes	No outcomes were assessed (counts of responses only).
Incomplete outcome data	No	Exclusions not reported
Selective reporting	Yes	Response after 9 months reported in full
Other sources of bias	Yes	No grounds for concern about other sources of bias from reading the study report

**Table:** Jenkinson 2003

***Study characteristics***		
Methods	Random allocation: method not specified	
Data	Recently discharged patients from 2 English inner city NHS Trusts	
Comparisons	1. 4‐page questionnaire 2. 12‐page questionnaire	
Outcomes	Response after 4 weeks (2 reminders)	
Topic	Health: Picker Patient Experience Questionnaire	
Mode of Administration	Postal	
Notes	Shorter (4‐page) vs. longer (12‐page) questionnaire The four‐page questionnaire contained the PPE‐15 nested within 31 questions covering demographics, health status and other aspects of patient experience. The 12‐page questionnaire contained the PPE‐15 nested within 108 questions; i.e. almost three and a half times as many items as on the four‐page version. Reminders were sent out after 2 weeks if no reply had been received; a second questionnaire was sent after a further 2 weeks if questionnaires remained unreturned.	
***Risk of bias***		
**Item**	**Authors' judgement**	**Support for judgement**
Sequence generation	Unclear	Random allocation: method unspecified
Allocation concealment?	Unclear	Random allocation: method unspecified
Blinding of participants and personnel	Unclear	Blinding of personnel not described. Participants were not blinded to intervention.
Blinding of outcome assessment	Yes	No outcomes were assessed (counts of responses only).
Incomplete outcome data	Yes	Exclusions were reported.
Selective reporting	Yes	Response after 4 weeks (2 reminders) reported in full
Other sources of bias	Yes	No grounds for concern about other sources of bias from reading the study report

**Table:** Jensen 1994

***Study characteristics***		
Methods	Random allocation: method not specified	
Data	Female graduates from the doctoral program in education from a private west‐coast university (US)	
Comparisons	1. Open‐ended questions first; closed questions next; demographic questions last 2. Open; demographic; closed 3. Closed; open; demographic 4. Closed; demographic; open 5. Demographic; open; closed 6. Demographic; closed; open Reminders sent at 6 and 12 weeks. Some graduates were living out of the country at the time of the study. They were sent postal vouchers and an envelope instead of a stamped envelope on the initial mailing and first follow‐up. On the second follow‐up, US citizens received a telephone call while overseas received another postal mailing.	
Outcomes	—	
Topic	Non‐health: Graduate school experiences	
Mode of Administration	Postal	
Notes	Age: 31‐65; mainly females	
***Risk of bias***		
**Item**	**Authors' judgement**	**Support for judgement**
Allocation concealment?	Unclear	B ‐ unclear

**Table:** Jepson 2005a

***Study characteristics***		
Methods	Random allocation: using random number generated in Excel	
Data	US Primary care physician members of the American Medical Association	
Comparisons	1. Word count ‐ 849 2. Word count ‐ 1145 3. Word count ‐ 1163 4. Word count ‐ 1164 5. Word count ‐ 1215 6. Word count ‐ 1216 7. Word count ‐ 1234 8. Word count ‐1423 9. Word count ‐ 1424 10. Word count ‐ 1447 11. Word count ‐ 1449 12. Word count ‐ 1461 13. Word count ‐ 1462 14. Word count ‐ 1494 15. Word count ‐ 1496 16. Word count ‐ 1519 17. Word count ‐ 1520 18. Word count ‐ 1560 19. Word count ‐ 1561 20. Word count ‐ 1703 21. Word count ‐ 1706 22. Word count ‐ 1737 23. Word count ‐ 1744 24. Word count ‐ 1756 25. Word count ‐ 1776 26. Word count ‐ 1785 27. Word count ‐ 1788 28. Word count ‐ 1807 29. Word count ‐ 1855 30. Word count ‐ 1867	
Outcomes	Response period not specified	
Topic	Health: attitudes towards cost quality trade‐offs in clinical practice	
Mode of Administration	Postal	
Notes	Short length: from word count 849 ‐ 1234; long length: from word count 1423 ‐ 1867; method of allocation ascertained through contact with author	
***Risk of bias***		
**Item**	**Authors' judgement**	**Support for judgement**
Allocation concealment?	Yes	A ‐ adequate

**Table:** Jepson 2005b

***Study characteristics***		
Methods	Random allocation: using random numbers generated in Excel	
Data	US Primary care physician members of the American Medical Association	
Comparisons	1. Word count ‐ 564 2. Word count ‐ 574 3. Word count ‐ 649 4. Word count ‐ 703 5. Word count ‐ 711 6. Word count ‐ 715 7. Word count ‐ 719 8. Word count ‐730 9. Word count ‐ 749 10. Word count ‐ 753 11. Word count ‐ 754 12. Word count ‐ 762 13. Word count ‐ 782 14. Word count ‐ 849 15. Word count ‐ 905 16. Word count ‐ 988	
Outcomes	Response period not specified	
Topic	Health: attitudes towards cost quality trade‐offs in clinical practice	
Mode of Administration	Postal	
Notes	Short length: from word count 564 to 730; long length: from word count 749 to 905; method of allocation ascertained through contact with author	
***Risk of bias***		
**Item**	**Authors' judgement**	**Support for judgement**
Allocation concealment?	Yes	A ‐ adequate

**Table:** Jobber 1983

***Study characteristics***		
Methods	Random allocation: method not specified	
Data	Textile companies listed in the Kompass‐Directory of UK Companies and 'Times Top 500'	
Comparisons	1. Prior letter; white questionnaire 2. Prior letter; blue questionnaire 3. No prior letter; white questionnaire 4. No prior letter; blue questionnaire	
Outcomes	Response period not specified	
Topic	Non‐health: information about the marketing strategies employed by the company	
Mode of Administration	Postal	
Notes	—	
***Risk of bias***		
**Item**	**Authors' judgement**	**Support for judgement**
Allocation concealment?	Unclear	B ‐ unclear

**Table:** Jobber 1985

***Study characteristics***		
Methods	Random allocation: method not specified	
Data	Senior marketing executives	
Comparisons	1. Cover letter contained offer of a free copy of results as the final paragraph of the body of the letter 2. Same cover letter but offer made in typed postscript 3. Same cover letter but offer made in handwritten postscript 4. Cover letter with no offer	
Outcomes	Response period not specified	
Topic	Non‐health: explore the design and extent of implementation of marketing information system	
Mode of Administration	Postal	
Notes	—	
***Risk of bias***		
**Item**	**Authors' judgement**	**Support for judgement**
Allocation concealment?	Unclear	B ‐ unclear

**Table:** Jobber 1988

***Study characteristics***		
Methods	Random allocation: method not specified	
Data	Building Society chief executives	
Comparisons	1. 20 pence incentive; no booklet 2. 20 pence incentive; booklet explaining survey included 3. No incentive; no booklet 4. No incentive; booklet explaining survey included	
Outcomes	Response period not specified	
Topic	Non‐health: ascertain management practices, and contextual and structural characteristics of societies	
Mode of Administration	Postal	
Notes	Number of subjects allocated to each intervention group ascertained through contact with author	
***Risk of bias***		
**Item**	**Authors' judgement**	**Support for judgement**
Allocation concealment?	Unclear	B ‐ unclear

**Table:** Jobber 1989

***Study characteristics***		
Methods	Random allocation: method not specified	
Data	Industrial goods companies	
Comparisons	1. Short questionnaire; 1‐sided printing 2. Short questionnaire; 2‐sided printing 3. Long questionnaire; 1‐sided printing 4. Long questionnaire; 2‐sided printing The long questionnaire comprised 2 different versions of a short questionnaire. The 2 versions were allocated randomly between treatments.	
Outcomes	Response period not specified	
Topic	Non‐health: selling approach and orientations used by the sample firms, evaluate the sale persons, size of firm, industry category, number of sales persons employed	
Mode of Administration	Postal	
Notes	—	
***Risk of bias***		
**Item**	**Authors' judgement**	**Support for judgement**
Allocation concealment?	Unclear	B ‐ unclear

**Table:** Jobber D 1985

***Study characteristics***		
Methods	Random allocation: systematic random sampling	
Data	Quality control managers of textile companies randomly selected from a directory of UK companies	
Comparisons	1. No pre‐notification 2. Telephone pre‐notification	
Outcomes	Response prior to second wave of the experiment	
Topic	Non‐health: quality management systems used by UK textile companies	
Mode of Administration	Postal	
Notes	Method of allocation confirmed through contact with author	
***Risk of bias***		
**Item**	**Authors' judgement**	**Support for judgement**
Allocation concealment?	Unclear	B ‐ unclear

**Table:** Johansson 1997a

***Study characteristics***		
Methods	Random allocation: method not specified	
Data	Norwegian citizens aged 16‐79 years	
Comparisons	1. No reward offered 2. Reward offered 1 reminder sent after 4 weeks	
Outcomes	Response period not specified	
Topic	Health: Quantitative Food Frequency Questionnaire (QFFQ)	
Mode of Administration	Postal	
Notes	Mean age: 42‐47	
***Risk of bias***		
**Item**	**Authors' judgement**	**Support for judgement**
Allocation concealment?	Unclear	B ‐ unclear

**Table:** Johansson 1997b

***Study characteristics***		
Methods	Random allocation: method not specified	
Data	Norwegian citizens aged 16‐79 years	
Comparisons	1. No reward offered 2. Reward offered 1 reminder sent after 4 weeks	
Outcomes	Response period not specified	
Topic	Health: Quantitative Food Frequency Questionnaire (QFFQ)	
Mode of Administration	Postal	
Notes	Mean age: 42‐47	
***Risk of bias***		
**Item**	**Authors' judgement**	**Support for judgement**
Allocation concealment?	Unclear	B ‐ unclear

**Table:** Johansson 1997c

***Study characteristics***		
Methods	Random allocation: method not specified	
Data	Norwegian citizens aged 16‐79 years who had not responded to a questionnaire	
Comparisons	1. Follow‐up by telephone 2. Follow‐up by post	
Outcomes	Response period not specified	
Topic	Health: Quantitative Food Frequency Questionnaire (QFFQ)	
Mode of Administration	Postal	
Notes	Mean age: 42‐47	
***Risk of bias***		
**Item**	**Authors' judgement**	**Support for judgement**
Allocation concealment?	Unclear	B ‐ unclear

**Table:** John 1994

***Study characteristics***		
Methods	Random allocation: method not specified	
Data	Cosmetologists aged 22 to 36 years	
Comparisons	1. $1 incentive in first mailing 2. $1 incentive in second mailing 3. No incentive 2‐page questionnaire, cover letter, survey fact sheet and stamped addressed envelope. Reminder postcard sent 1 week after first mailing	
Outcomes	Response period not specified	
Topic	Health: screening questions ‐ recent health problems, reproductive history; outcome of the most recent pregnancy	
Mode of Administration	Postal	
Notes	Age: 22‐36; mainly females	
***Risk of bias***		
**Item**	**Authors' judgement**	**Support for judgement**
Allocation concealment?	Unclear	B ‐ unclear

**Table:** Joinson 2005a

***Study characteristics***		
Methods	Random allocation: using random numbers generated in Excel	
Data	Students at the Open University, UK	
Comparisons	1. Salutation ‐ 'Dear Student' 2. Salutation ‐ 'Dear Open University Student' 3. Salutation ‐ 'Dear Forename' (e.g. Dear John) 4. Salutation ‐ 'Dear Forename Surname' (e.g. Dear John Doe)	
Outcomes	Response within 14 days	
Topic	Non‐health: volunteering to become a member of a survey panel (PRESTO)	
Mode of Administration	Electronic: email	
Notes	Method of allocation ascertained through contact with author	
***Risk of bias***		
**Item**	**Authors' judgement**	**Support for judgement**
Allocation concealment?	Yes	A ‐ adequate

**Table:** Joinson 2005b

***Study characteristics***		
Methods	Random allocation: using random numbers generated in Excel	
Data	Students at the Open University, UK	
Comparisons	1. Salutation ‐ 'Dear Student' 2. Salutation ‐ 'Dear Open University Student' 3. Salutation ‐ 'Dear Forename' (e.g. Dear John) 4. Salutation ‐ 'Dear Forename Surname' (e.g. Dear John Doe)	
Outcomes	Response within 14 days	
Topic	Non‐health: inviting the existing panel members to exit the panel	
Mode of Administration	Electronic: email	
Notes	Method of allocation ascertained through contact with author	
***Risk of bias***		
**Item**	**Authors' judgement**	**Support for judgement**
Allocation concealment?	Yes	A ‐ adequate

**Table:** Joinson 2005c

***Study characteristics***		
Methods	Random allocation: using random numbers generated in Excel	
Data	Students at the Open University, UK	
Comparisons	1. Salutation ‐ 'Dear Student' 2. Salutation ‐ 'Dear Forename' (e.g. Dear John) 3. Salutation ‐ 'Dear Forename Surname' (e.g. Dear John Doe) 4. High Power ‐ "From Professor (name), Pro‐Vice chancellor, (strategy, planning and partnerships), the OU 5. Neutral Power ‐ "From (name), (strategy, planning and partnerships), the OU	
Outcomes	Response within 14 days	
Topic	Non‐health: inviting the panel members to complete the survey	
Mode of Administration	Electronic: online survey	
Notes	Method of allocation ascertained through contact with author	
***Risk of bias***		
**Item**	**Authors' judgement**	**Support for judgement**
Allocation concealment?	Yes	A ‐ adequate

**Table:** Joinson 2007a

***Study characteristics***		
Methods	Random allocation: using random numbers generated in Excel	
Data	Members of an online student panel at the Open University, UK	
Comparisons	1. High Power ‐ "From Professor (name), Pro‐Vice chancellor, (strategy, planning and partnerships), the OU 2. Neutral Power ‐ "From (name), (strategy, planning and partnerships), the OU 3. Salutation ‐ 'Dear Forename' (e.g. Dear John) 4. Salutation ‐ 'Dear Presto panel member'	
Outcomes	Response within 14 days	
Topic	Non‐health: socioeconomic status	
Mode of Administration	Electronic: online survey	
Notes	Method of allocation ascertained through contact with author; mean age: 41.8 years; mainly females	
***Risk of bias***		
**Item**	**Authors' judgement**	**Support for judgement**
Allocation concealment?	Yes	A ‐ adequate

**Table:** Joinson 2007b

***Study characteristics***		
Methods	Random allocation: using random numbers generated in Excel	
Data	Members of an online student panel at the Open University, UK	
Comparisons	1. Personalised URL (unique URL with identifier encoded in the link) 2. Authentication required (URL requires logon to access the survey)	
Outcomes	Response within 14 days	
Topic	Non‐health: part‐time student costs and fees	
Mode of Administration	Electronic: online survey	
Notes	Method of allocation ascertained through contact with author; mean age: 43.6 years; mainly females	
***Risk of bias***		
**Item**	**Authors' judgement**	**Support for judgement**
Allocation concealment?	Yes	A ‐ adequate

**Table:** Jones 1978

***Study characteristics***		
Methods	Random allocation: method not specified	
Data	Individuals who had planned group conventions and/or meetings	
Comparisons	1. Science appeal (SA); commemorative stamp (CS); J&L sponsor (J&L) 2. SA; CS; University sponsor (US) 3. SA; CS; Government sponsor (GS) 4. SA; regular stamp (RS); J&L 5. SA; RS; US 6. SA; RS; GS 7. SA; business‐reply envelope (BR); J&L 8. SA; BR; US 9. SA; BR; GS 10. User appeal (UA); CS; J&L 11. UA; CS; US 12. UA; CS; GS 13. UA; RS; J&L 14. UA; RS; US 15. UA; RS; GS 16. UA; BR; J&L 17. UA; BR; US 18. UA; BR; GS 19. Resort park appeal (RA); CS; J&L 20. RA; CS; US 21. RA; CS; GS 22. RA; RS; J&L 23. RA; RS; US 24. RA; RS; GS 25. RA; BR; J&L 26. RA; BR; US 27. RA; BR; GA	
Outcomes	Response within 6 weeks	
Topic	Non‐health: characteristics of the group, respondent attitudes towards meeting facilities, demographic factors	
Mode of Administration	Postal	
Notes	—	
***Risk of bias***		
**Item**	**Authors' judgement**	**Support for judgement**
Allocation concealment?	Unclear	B ‐ unclear

**Table:** Jones 2000

***Study characteristics***		
Methods	Random allocation: method not specified	
Data	Adult patients admitted for treatment between 14/09/98 and 12/12/98	
Comparisons	1. No incentive 2. $2 3. $5 4. SF‐36 5. SF‐12 6. MH‐5 7. MH‐1	
Outcomes	Response period not specified	
Topic	Health: health status survey	
Mode of Administration	Postal	
Notes	—	
***Risk of bias***		
**Item**	**Authors' judgement**	**Support for judgement**
Allocation concealment?	Unclear	B ‐ unclear

**Table:** Junghans 2005

***Study characteristics***		
Methods	Random allocation: using minimisation software	
Data	2 general practices in England	
Comparisons	1. Opt‐in (asked to actively signal willingness to participate in research) 2. Opt‐out (contacted repeatedly unless they signalled unwillingness to participate)	
Outcomes	Response period within 2 weeks	
Topic	Health: patients with angina	
Mode of Administration	Postal	
Notes	The identity of the trial was kept in a sealed envelope and was known only to the research assistant.	
***Risk of bias***		
**Item**	**Authors' judgement**	**Support for judgement**
Allocation concealment?	Yes	A ‐ adequate

**Table:** Juszczak 2021

***Study characteristics***		
Methods	Random allocation: method unspecified	
Data	Parents of preterm babies from participating neonatal units, UK	
Comparisons	Conditional non‐monetary incentive (£15 voucher) vs. unconditional non‐monetary incentive (£15 voucher)	
Outcomes	Response after one reminder	
Topic	Health (neurodevelopmental disability at 2 years of age)	
Mode of Administration	Mixed	
Notes	Questionnaire not described. Questionnaires were sent to all parents where infant(s) vital status and address could be confirmed by both post and as a link to an online submission form via email and text message, where these contact details were available. Parents were also reminded to complete the questionnaire via text and/or email during the follow‐up window. All parents were offered the option of completing the questionnaire online or, as a last resort, via telephone.	
***Risk of bias***		
**Item**	**Authors' judgement**	**Support for judgement**
Sequence generation	Unclear	Random allocation: method unspecified
Allocation concealment?	Unclear	Random allocation: method unspecified
Blinding of participants and personnel	No	SIFT office staff at the NPEU Clinical Trials Unit were aware of allocation due to the nature of the interventions and the practicalities involved in sending out the letters and the vouchers.
Blinding of outcome assessment	Yes	No outcomes were assessed (counts of responses only).
Incomplete outcome data	Yes	Exclusions reported
Selective reporting	Yes	Response after one reminder reported in full
Other sources of bias	Yes	No grounds for concern about other sources of bias from reading the study report

**Table:** Kahle 1978

***Study characteristics***		
Methods	Random allocation: method not specified	
Data	Psychiatrists and clinical psychologists	
Comparisons	Experiment 1: 1. Dillman's 3‐wave mailing design 2. As (1) except first 2 waves received non‐profit bulk rate permit number printed where stamp had been 3. As (2) except pre‐printed labels used to address envelopes rather than addresses typed individually on envelopes Experiment 2: As experiment 1 but in final wave questionnaire sent by: 1. Certified mail 2. First‐class mail	
Outcomes	Response period not specified	
Topic	Health: involuntary civil commitments	
Mode of Administration	Postal	
Notes	—	
***Risk of bias***		
**Item**	**Authors' judgement**	**Support for judgement**
Allocation concealment?	Unclear	B ‐ unclear

**Table:** Kalafatis 1995

***Study characteristics***		
Methods	Random allocation: using random number generation	
Data	Danish participants in a non‐price‐based promotion that utilised an American sporting theme	
Comparisons	1. No incentives 2. Unconditional 5% value of coupon 3. Unconditional 10% value of coupon 4. Unconditional 15% value of coupon 5. Conditional 5% value of coupon 6. Conditional 10% value of coupon 7. Conditional 15% value of coupon 8. Free gift 9. No free gift	
Outcomes	Response period not specified	
Topic	Non‐health: sports ‐ promotional offers, viewing patterns of sports programmes, shopping habits of sports goods and perceptions of different sports	
Mode of Administration	Postal	
Notes	—	
***Risk of bias***		
**Item**	**Authors' judgement**	**Support for judgement**
Allocation concealment?	Unclear	B ‐ unclear

**Table:** Kalantar 1999

***Study characteristics***		
Methods	Random allocation: computerised random number generation	
Data	Residents of Western Sydney, Australia	
Comparisons	1. Long questionnaire (7 pages) 2. Short questionnaire (1 page) 3. Scratch lottery worth $1 to win up to $2500 4. No lottery	
Outcomes	Response period not specified	
Topic	Health: bowel function and faecal incontinence	
Mode of Administration	Postal	
Notes	Method of allocation ascertained through contact with author	
***Risk of bias***		
**Item**	**Authors' judgement**	**Support for judgement**
Allocation concealment?	Yes	A ‐ adequate

**Table:** Kaplan 1970a

***Study characteristics***		
Methods	Random allocation: using alternation	
Data	Residents of Boston and Brockton, Massachusetts, USA	
Comparisons	1. Long questionnaire 2. Short questionnaire 3. Impersonal letter (no inside address and the salutation is 'Dear Madam') 4. Personal letter (with complete address and the salutation is 'Dear Mrs. name') 5. Stamped return envelope 6. Franked return envelope 7. Non‐specific signer 8. Jewish signer 9. Irish signer	
Outcomes	Response period not specified	
Topic	Health: reproductive history and the use of oral contraceptives	
Mode of Administration	Postal	
Notes	Age: 20‐70 years; mainly females	
***Risk of bias***		
**Item**	**Authors' judgement**	**Support for judgement**
Allocation concealment?	No	C ‐ inadequate

**Table:** Kaplan 1970b

***Study characteristics***		
Methods	Random allocation: using alternation	
Data	Residents of Boston and Brockton, Massachusetts, USA	
Comparisons	1. Irish signer 2. Irish, Professor signer 3. Non‐specific signer	
Outcomes	Response period not specified	
Topic	Health: reproductive history and the use of oral contraceptives	
Mode of Administration	Postal	
Notes	Age: 20‐70 years; mainly females	
***Risk of bias***		
**Item**	**Authors' judgement**	**Support for judgement**
Allocation concealment?	No	C ‐ inadequate

**Table:** Kaplowitz 2004

***Study characteristics***		
Methods	Random allocation: using alternation	
Data	Residential and agricultural landowners in the Sycamore creek watershed, USA	
Comparisons	1. Survey instrument ‐ colour 2. Survey instrument ‐ black and white	
Outcomes	Response period not specified	
Topic	Non‐health: homeowner preferences for watershed management practices	
Mode of Administration	Postal	
Notes	—	
***Risk of bias***		
**Item**	**Authors' judgement**	**Support for judgement**
Allocation concealment?	No	C ‐ inadequate

**Table:** Kasprzyk 2001

***Study characteristics***		
Methods	Random allocation: method not specified	
Data	Sample of the general internists listed on the American Medical Association files who spend time on direct patient care, deal with STD diagnosis and have a listed mailing address	
Comparisons	1. First‐class mailing; no incentive 2. First‐class mailing; $15 cash 3. First‐class mailing; $25 cash 4. FedEx mailing; no incentive 5. FedEx mailing; $15 cash 6. FedEx mailing; $25 cash	
Outcomes	Response after final reminder mailing, 8 weeks after initial survey	
Topic	Health: physician and practice characteristics, STD diagnosis. treatment and control practice, opinions about STD reporting requirements and partner notification	
Mode of Administration	Postal	
Notes	—	
***Risk of bias***		
**Item**	**Authors' judgement**	**Support for judgement**
Allocation concealment?	Unclear	B ‐ unclear

**Table:** Kawash 1971

***Study characteristics***		
Methods	Random allocation: using table of random numbers	
Data	Faculty members of University of Illinois	
Comparisons	1. Personal signature 2. Mimeographed facsimile	
Outcomes	Response period not specified	
Topic	Non‐health: attitudes towards audiovisual instructional materials	
Mode of Administration	Postal	
Notes	Method of allocation ascertained through contact with author	
***Risk of bias***		
**Item**	**Authors' judgement**	**Support for judgement**
Allocation concealment?	Yes	A ‐ adequate

**Table:** Keating 2008

***Study characteristics***		
Methods	Random allocation: method unspecified	
Data	Physicians caring for patients in the Cancer Care Outcomes Research and Surveillance (CanCORS) Consortium	
Comparisons	Lower non‐monetary incentive ($20 cheque) vs. higher non‐monetary incentive ($50 cheque)	
Outcomes	Response after 13 weeks (2 postal reminders and one phone call)	
Topic	Health (physicians' beliefs regarding treatments, their style of practice, barriers to accessing care for their patients, practice characteristics, financial arrangements, and demographics)	
Mode of Administration	Postal (optional online response)	
Notes	10‐13 pages, 10‐15 minutes to complete. Cover letter was printed on letterhead from the Northern California Cancer Center and was signed by the centre's Chief Scientific Officer and co‐signed by the Director of the National Cancer Institute and the Medical Director of the American Cancer Society. Each packet also included one or more letters of endorsement matched to the recipient's specialty. The surveys were mailed by priority mail with a stamped, pre‐addressed return envelope. Physicians were also given the option of responding to the survey via a secure website, after logging in with a username and password. Three weeks after the initial mailing, another copy of the survey and cover letter (without another cheque) was sent by first‐class mail to all non‐responders. Approximately 2 weeks later, a research assistant placed phone calls to the offices of non‐responding physicians to verify that the survey had been received, encourage completion, and offer to mail or fax a replacement questionnaire. Up to 10 attempts were made to reach each non‐responding physician. After another 6‐8 weeks, a third mailing of the survey and cover letter was sent to non‐responding physicians with another incentive (of the same amount as the first).	
***Risk of bias***		
**Item**	**Authors' judgement**	**Support for judgement**
Sequence generation	Unclear	Random allocation: method unspecified
Allocation concealment?	Unclear	Random allocation: method unspecified
Blinding of participants and personnel	Unclear	Blinding of personnel not described. Participants were not blinded to intervention.
Blinding of outcome assessment	Yes	No outcomes were assessed (counts of responses only).
Incomplete outcome data	No	Exclusions were not reported.
Selective reporting	Yes	Response after 13 weeks (2 postal reminders and one phone call) reported in full
Other sources of bias	Yes	No grounds for concern about other sources of bias from reading the study report

**Table:** Keding 2016a

***Study characteristics***		
Methods	Random allocation: method unspecified	
Data	Consenting participants within the Acupuncture and Depression (ACUDep) trial from primary care practices in Yorkshire and the North of England	
Comparisons	Pre‐notification SMS vs. no pre‐notification	
Outcomes	Response after 13 weeks	
Topic	Health (quality of life measures)	
Mode of Administration	Postal	
Notes	12 A4 pages long; contained quality of life measures, including Patient Health Questionnaire 9 (PHQ‐9) and EuroQoL. Pre‐notification text messages were sent on the day the postal questionnaire was sent out to participants.	
***Risk of bias***		
**Item**	**Authors' judgement**	**Support for judgement**
Sequence generation	Unclear	Random allocation: method unspecified
Allocation concealment?	Unclear	Random allocation: method unspecified
Blinding of participants and personnel	Yes	Participants did not know that they were part of an RCT of text messaging so they were blind to the study hypothesis.
Blinding of outcome assessment	Yes	No outcomes were assessed (counts of responses only).
Incomplete outcome data	Yes	Exclusions reported
Selective reporting	Yes	Response after 13 weeks reported in full
Other sources of bias	Yes	No grounds for concern about other sources of bias from reading the study report

**Table:** Keding 2016b

***Study characteristics***		
Methods	Random allocation: method unspecified	
Data	Consenting participants within the Acupuncture and Depression (ACUDep) trial from primary care practices in Yorkshire and the North of England.	
Comparisons	Pre‐notification SMS vs. post‐notification	
Outcomes	Response after 13 weeks.	
Topic	Health (quality of life measures)	
Mode of Administration	Postal	
Notes	12 A4 pages long; contained quality of life measures, including Patient Health Questionnaire 9 (PHQ‐9) and EuroQoL. Pre‐notification text messages were sent on the day the postal questionnaire was sent out to participants. Post‐notification text messages were sent 4 days after the postal questionnaire was sent out.	
***Risk of bias***		
**Item**	**Authors' judgement**	**Support for judgement**
Sequence generation	Unclear	Random allocation: method unspecified
Allocation concealment?	Unclear	Random allocation: method unspecified
Blinding of participants and personnel	Yes	Participants did not know that they were part of an RCT of text messaging so they were blind to the study hypothesis.
Blinding of outcome assessment	Yes	No outcomes were assessed (counts of responses only).
Incomplete outcome data	Yes	Exclusions reported
Selective reporting	Yes	Response after 13 weeks reported in full
Other sources of bias	Yes	No grounds for concern about other sources of bias from reading the study report

**Table:** Keding 2016c

***Study characteristics***		
Methods	Random allocation: method unspecified	
Data	Consenting participants within the Acupuncture and Depression (ACUDep) trial from primary care practices in Yorkshire and the North of England.	
Comparisons	Post‐notification SMS vs. no notification	
Outcomes	Response after 13 weeks.	
Topic	Health (quality of life measures)	
Mode of Administration	Postal	
Notes	12 A4 pages long; contained quality of life measures, including Patient Health Questionnaire 9 (PHQ‐9) and EuroQol. Post‐notification text messages were sent 4 days after the postal questionnaire was sent out.	
***Risk of bias***		
**Item**	**Authors' judgement**	**Support for judgement**
Sequence generation	Unclear	Random allocation: method unspecified
Allocation concealment?	Unclear	Random allocation: method unspecified
Blinding of participants and personnel	Yes	Participants did not know that they were part of an RCT of text messaging so they were blind to the study hypothesis.
Blinding of outcome assessment	Yes	No outcomes were assessed (counts of responses only).
Incomplete outcome data	Yes	Exclusions reported
Selective reporting	Yes	Response after 13 weeks reported in full
Other sources of bias	Yes	No grounds for concern about other sources of bias from reading the study report

**Table:** Keeter 2001

***Study characteristics***		
Methods	Random allocation: method not specified	
Data	Attorneys, clinical social workers, college and university faculty, staff and students, employees and employers in business organisations and a sample of physicians	
Comparisons	1. White questionnaire 2. Pink questionnaire 3. Green questionnaire	
Outcomes	Response period not specified	
Topic	Health: purchase of major medical equipment Non‐health: housing market, parking and ridesharing, workload, distribution of time, attitudes	
Mode of Administration	Postal	
Notes	Results of 7 different studies all examining the same intervention over a 14‐month period	
***Risk of bias***		
**Item**	**Authors' judgement**	**Support for judgement**
Allocation concealment?	Unclear	B ‐ unclear

**Table:** Kenyon 2005

***Study characteristics***		
Methods	Random allocation: computer‐generated	
Data	Mothers of the MRC ORACLE Trial that evaluated the use of antibiotics to improve neonatal outcome after preterm labour/preterm rupture of the membrane	
Comparisons	1. £5 voucher 2. No voucher	
Outcomes	Response period not specified	
Topic	Health: child's health and development	
Mode of Administration	Postal	
Notes	12 pages, A4 size Letter with the questionnaire was individualised for the child concerned and the parents. Envelope was franked, including an SAE. Six weeks after the first questionnaire, a reminder was sent to those who had not responded and included a questionnaire and a £5 voucher incentive (or not)	
***Risk of bias***		
**Item**	**Authors' judgement**	**Support for judgement**
Sequence generation	Yes	Random allocation: computer‐generated
Allocation concealment?	Yes	Random allocation: computer‐generated
Blinding of participants and personnel	Unclear	Blinding of personnel not described. Participants were not blinded to intervention.
Blinding of outcome assessment	Yes	No outcomes were assessed (counts of responses only).
Incomplete outcome data	Yes	Exclusions were reported.
Selective reporting	Yes	First response reported in full
Other sources of bias	Yes	No grounds for concern about other sources of bias from reading the study report

**Table:** Keown 1985a

***Study characteristics***		
Methods	Random allocation: method not specified	
Data	Japanese business executives	
Comparisons	1. $1 incentive 2. No incentive	
Outcomes	Response period not specified	
Topic	Non‐health: attitudes towards business risk	
Mode of Administration	Postal	
Notes	—	
***Risk of bias***		
**Item**	**Authors' judgement**	**Support for judgement**
Allocation concealment?	Unclear	B ‐ unclear

**Table:** Keown 1985b

***Study characteristics***		
Methods	Random allocation: method not specified	
Data	Business executives (Hong Kong)	
Comparisons	1. $1 incentive 2. No incentive	
Outcomes	Response period not specified	
Topic	Non‐health: attitudes towards business risk	
Mode of Administration	Postal	
Notes	—	
***Risk of bias***		
**Item**	**Authors' judgement**	**Support for judgement**
Allocation concealment?	Unclear	B ‐ unclear

**Table:** Kephart 1958

***Study characteristics***		
Methods	Random allocation: method not specified	
Data	Women who had passed their Pennsylvania State Nursing Board exams	
Comparisons	1. Regular stamp; no preview or follow‐up 2. Preview sent 1 week prior to questionnaire 3. Follow‐up (duplicate questionnaire, letter and return envelope) 4. Preview and follow‐up 5. Airmail stamp 6. Special delivery mail 7. Incentive of a penny 8. Incentive of a nickel 9. Incentive of a dime 10. Incentive of a quarter	
Outcomes	Response period not specified	
Topic	Health: attitudes towards nursing profession	
Mode of Administration	Postal	
Notes	Mainly females	
***Risk of bias***		
**Item**	**Authors' judgement**	**Support for judgement**
Allocation concealment?	Unclear	B ‐ unclear

**Table:** Kereakoglow 2013

***Study characteristics***		
Methods	Random allocation: computer‐generated	
Data	Oncology physicians and nurses	
Comparisons	Glossy paper with colour vs. standard paper without colour	
Outcomes	Response after 8 weeks (3 reminders)	
Topic	Health (practices, preferences, and attitudes surrounding the concept of offering results of clinical trials to study participants)	
Mode of Administration	Postal	
Notes	Questionnaire not described. Each study packet included a cover letter, an SAE, outer envelope, and questionnaire. The cover letter, explaining the primary purpose of the study was printed on Cancer and Leukemia Group B (CALGB) research group letterhead to encourage increased response from the CALGB members; To maximise response, facsimile (fax) and electronic mail (email) reminders were sent 3 weeks after the initial materials were sent. Clinicians who did not return the survey within 4 weeks of the initial mailing were contacted, once again, by telephone, email, or fax. Clinicians who had not responded by 8 weeks following the initial mailing and who were able to be located were resent the study materials (either enhanced or standard, according to their original randomisation allocation).	
***Risk of bias***		
**Item**	**Authors' judgement**	**Support for judgement**
Sequence generation	Yes	Random allocation: computer‐generated
Allocation concealment?	Yes	Random allocation: computer‐generated
Blinding of participants and personnel	Yes	The potential participants did not know there were two versions of the survey, and did not know they were randomised to receive one of the versions.
Blinding of outcome assessment	Yes	No outcomes were assessed (counts of responses only).
Incomplete outcome data	No	Exclusions were not reported.
Selective reporting	Yes	Response after 8 weeks (3 reminders) reported in full
Other sources of bias	No	Results potentially confounded between glossy vs. standard paper and colour vs. white paper

**Table:** Kerin 1976

***Study characteristics***		
Methods	Random allocation: method not specified	
Data	Corporate presidents from 'Fortune 500' firms	
Comparisons	1. Individual cover letter; altruistic appeal; stamp on return envelope 2. Individual; altruistic; no stamp 3. individual; egoistic appeal; stamp 4. individual; egoistic; no stamp 5. Form cover letter; altruistic; stamp 6. Form cover letter; altruistic; no stamp 7. Form; egoistic; stamp 8. Form; egoistic; no stamp	
Outcomes	Response period not specified	
Topic	Non‐health: product recall practices	
Mode of Administration	Postal	
Notes	—	
***Risk of bias***		
**Item**	**Authors' judgement**	**Support for judgement**
Allocation concealment?	Unclear	B ‐ unclear

**Table:** Kerin 1981

***Study characteristics***		
Methods	Random allocation: method not specified	
Data	Senior marketing executives	
Comparisons	1. Offered results 2. Not offered results	
Outcomes	Response period not specified	
Topic	Non‐health: interaction between sales and advertising functions in the design and execution of promotion strategy	
Mode of Administration	Postal	
Notes	—	
***Risk of bias***		
**Item**	**Authors' judgement**	**Support for judgement**
Allocation concealment?	Unclear	B ‐ unclear

**Table:** Kernan 1971

***Study characteristics***		
Methods	Random allocation: method not specified	
Data	Residents of Cincinnati	
Comparisons	1. Personalised address; first‐class 2. Personalised address; bulk rate 3. Occupant address; first‐class 4. Occupant address; bulk rate	
Outcomes	Response within 3 weeks	
Topic	Non‐health: community's general interest in new sports stadium	
Mode of Administration	Postal	
Notes	—	
***Risk of bias***		
**Item**	**Authors' judgement**	**Support for judgement**
Allocation concealment?	Unclear	B ‐ unclear

**Table:** Khadjesari 2011a

***Study characteristics***		
Methods	Random allocation: Computer‐generated randomisation in Java	
Data	Participants in the 'Down Your Drink' online trial of a Web‐based intervention to reduce alcohol consumption	
Comparisons	Conditional non‐monetary incentive (£5 Amazon voucher) vs. £5 donation to charity vs. entry in a £250 prize draw vs. no incentive	
Outcomes	Response after 1 reminder	
Topic	Health (alcohol consumption, dependence and mental health)	
Mode of Administration	Electronic	
Notes	Participants were emailed a request to provide follow‐up data. The email contained a URL to the study questionnaires, stressed the importance of providing follow‐up data, and conveyed gratitude to participants for providing this information. The first incentive study was undertaken with participants in the DYD pilot who did not respond to an email invitation to provide follow‐up data within 1 week at its final (3‐month) follow‐up point. The second study was undertaken with all participants in the main DYD trial at its final (12‐month) follow‐up point during a defined time period of approximately 9 months.	
***Risk of bias***		
**Item**	**Authors' judgement**	**Support for judgement**
Sequence generation	Yes	Random allocation: computer‐generated randomisation in Java
Allocation concealment?	Yes	Random allocation: computer‐generated randomisation in Java
Blinding of participants and personnel	Unclear	Blinding of personnel not described. Participants were not blinded to intervention.
Blinding of outcome assessment	Yes	No outcomes were assessed (counts of responses only).
Incomplete outcome data	No	Exclusions were not reported.
Selective reporting	Yes	Response after 1 reminder reported in full
Other sources of bias	Yes	No grounds for concern about other sources of bias from reading the study report

**Table:** Khadjesari 2011b

***Study characteristics***		
Methods	Random allocation: computer‐generated randomisation in Java	
Data	Participants in the 'Down Your Drink' online trial of a Web‐based intervention to reduce alcohol consumption	
Comparisons	Conditional non‐monetary incentive (£10 Amazon voucher) vs. no incentive	
Outcomes	Response after 1 reminder	
Topic	Health (alcohol consumption, dependence and mental health)	
Mode of Administration	Electronic	
Notes	Participants were emailed a request to provide follow‐up data. The email contained a URL to the study questionnaires, stressed the importance of providing follow‐up data, and conveyed gratitude to participants for providing this information. The first incentive study was undertaken with participants in the DYD pilot who did not respond to an email invitation to provide follow‐up data within 1 week at its final (3‐month) follow‐up point. The second study was undertaken with all participants in the main DYD trial at its final (12‐month) follow‐up point during a defined time period of approximately 9 months.	
***Risk of bias***		
**Item**	**Authors' judgement**	**Support for judgement**
Sequence generation	Yes	Random allocation: computer‐generated randomisation in Java
Allocation concealment?	Yes	Random allocation: computer‐generated randomisation in Java
Blinding of participants and personnel	Unclear	Blinding of personnel not described. Participants were not blinded to intervention.
Blinding of outcome assessment	Yes	No outcomes were assessed (counts of responses only).
Incomplete outcome data	No	Exclusions were not reported.
Selective reporting	Yes	Response after 1 reminder reported in full
Other sources of bias	Yes	No grounds for concern about other sources of bias from reading the study report

**Table:** Kilsdonk 2015

***Study characteristics***		
Methods	Random allocation: computer generated	
Data	750 adult childhood cancer survivors, NL	
Comparisons	Web‐based vs. choice (web or paper‐based questionnaire) at first contact vs. choice (web or paper‐based questionnaire) at second contact vs. choice (web or paper‐based questionnaire) at third contact	
Outcomes	First response and response after one reminder	
Topic	Health (medical history, disease symptoms, medication use, lifestyle, and quality of life).	
Mode of Administration	Postal and web‐based	
Notes	The questionnaires for male and female cancer survivors contained 97 and 112 questions, respectively. All CCSs received a postal package including a cover letter signed by the local physician responsible for CCSs follow‐up care in which the login procedure for the Web‐based questionnaire was explained and login details were given. An information sheet, an informed consent form, a refusal form, and a pre‐stamped return envelope were included. Paper‐based questionnaires were added to the invitation at various time points depending on the study arm. CCSs either received the paper‐based questionnaire at first contact (study arm 3), second contact (study arm 2), or third contact (study arm 1). All CCSs receiving the Web‐based questionnaire at any of the time points had the option to apply for a copy of the paper‐based questionnaire by contacting the study co‐ordinator through email or telephone.	
***Risk of bias***		
**Item**	**Authors' judgement**	**Support for judgement**
Sequence generation	Yes	Random allocation: computer‐generated
Allocation concealment?	Yes	Random allocation: computer‐generated
Blinding of participants and personnel	Unclear	Blinding of personnel not described. Participants were not blinded to intervention.
Blinding of outcome assessment	Yes	No outcomes were assessed (counts of responses only).
Incomplete outcome data	No	Exclusions were not reported.
Selective reporting	Yes	First response and response after one reminder reported in full
Other sources of bias	Yes	No grounds for concern about other sources of bias from reading the study report

**Table:** Kindra 1985

***Study characteristics***		
Methods	Random allocation: method not specified	
Data	Households listed in a telephone directory (Montreal, Canada)	
Comparisons	1. Pre‐contact; lottery incentive 2. No pre‐contact; lottery incentive 3. Pre‐contact; no lottery incentive 4. No pre‐contact; no lottery incentive	
Outcomes	Response period not specified	
Topic	Non‐health: elicit consumer response to product advertising	
Mode of Administration	Postal	
Notes	—	
***Risk of bias***		
**Item**	**Authors' judgement**	**Support for judgement**
Allocation concealment?	No	C ‐ inadequate

**Table:** King 1978

***Study characteristics***		
Methods	Random allocation: systematic sampling procedure with random start	
Data	Registered bank holding companies	
Comparisons	1. Cover letter most personalised 2. Cover letter least personalised	
Outcomes	Response period not specified	
Topic	Not specified	
Mode of Administration	Postal	
Notes	—	
***Risk of bias***		
**Item**	**Authors' judgement**	**Support for judgement**
Allocation concealment?	No	C ‐ inadequate

**Table:** Koloski 2001

***Study characteristics***		
Methods	Random allocation: random block procedure	
Data	People aged 18 years and above listed on the 1996 Local Government electoral roll, Penrith, Australia	
Comparisons	1. Short (28‐page) questionnaire; lottery card included 2. Short questionnaire; no lottery card 3. Long questionnaire (32 pages); lottery card included 4. Long questionnaire; no lottery card	
Outcomes	Response after 8 phases of follow‐up	
Topic	Health: questions on common health problems especially on stomach and bowel, Delusions Symptoms States Inventory, SF‐12	
Mode of Administration	Postal	
Notes	Age: 41.9‐46.6 years; mainly females	
***Risk of bias***		
**Item**	**Authors' judgement**	**Support for judgement**
Allocation concealment?	Unclear	B ‐ unclear

**Table:** Koloski 2013

***Study characteristics***		
Methods	Random allocation: method unspecified	
Data	Participants in a population‐based study on functional gastrointestinal disorders in the community, Australia	
Comparisons	Unconditional non‐monetary incentive ($1 lottery ticket) vs. conditional non‐monetary incentive ($1 lottery ticket) vs. no incentive	
Outcomes	Response after the first mail out and after week‐1 reminder letter	
Topic	Health (gastrointestinal disorders)	
Mode of Administration	Postal	
Notes	32‐page questionnaire. The cover letter included several elements designed to increase the subjects' personal interest in the study. These included a personal salutation, a scanned version of the investigator's handwritten signature, an explanation of the nature and importance of the research, and reassurance of confidentiality.	
***Risk of bias***		
**Item**	**Authors' judgement**	**Support for judgement**
Sequence generation	Unclear	Random allocation: method unspecified
Allocation concealment?	Unclear	Random allocation: method unspecified
Blinding of participants and personnel	Unclear	Blinding of personnel not described. Participants were not blinded to intervention.
Blinding of outcome assessment	Yes	No outcomes were assessed (counts of responses only).
Incomplete outcome data	Yes	Exclusions reported; the sample size was reduced by 69 ineligibles (n = 50 return to senders and n = 19 deaths).
Selective reporting	Yes	Response after the first mail out and after week 1 reminder letter reported in full
Other sources of bias	Yes	No grounds for concern about other sources of bias from reading the study report

**Table:** Koo 1995

***Study characteristics***		
Methods	Random allocation: random function in Microsoft Excel programme	
Data	Girls recruited by volunteers from the Canadian Cancer Society	
Comparisons	1. Real signature on cover letter 2. Printed signature	
Outcomes	Response within 105 days	
Topic	Health: dietary and lifestyle determinants of the onset of menarche	
Mode of Administration	Postal	
Notes	Allocation was not concealed; age: 7.5‐14.9 years; mainly females	
***Risk of bias***		
**Item**	**Authors' judgement**	**Support for judgement**
Allocation concealment?	No	C ‐ inadequate

**Table:** Koo 1996

***Study characteristics***		
Methods	Random allocation: method not specified	
Data	Girls recruited by volunteers from the Canadian Cancer Society who had not responded in a previous study	
Comparisons	1. Regular reminder letter 2. Reminder letter with telephone reminder indicated 3. Reminder letter with telephone interview indicated	
Outcomes	Response within 16 days (prior to telephone interview)	
Topic	Health: dietary and lifestyle determinants of the onset of menarche	
Mode of Administration	Postal	
Notes	Age: 8.7‐16.2 years; mainly females	
***Risk of bias***		
**Item**	**Authors' judgement**	**Support for judgement**
Allocation concealment?	Unclear	B ‐ unclear

**Table:** Kost 2018

***Study characteristics***		
Methods	Random allocation: method unspecified	
Data	2228 volunteers from ResearchMatch (US research volunteer registry)	
Comparisons	Ultrashort vs. short vs. long Conditional non‐monetary incentive (smaller $10 Amazon voucher vs. larger $20)	
Outcomes	e‐login and e‐completed	
Topic	Non‐health (understanding of research participant experience ‐ motivation and satisfaction)	
Mode of Administration	Web survey	
Notes	Research Participant Perception Survey, RPPS: ultrashort (13 questions), short (25 questions), and long (72 questions) Volunteers were randomised to receive an email from the investigator providing a personalised hyperlink to one of the 3 surveys. Individuals clicking on the survey link encountered informed consent information including an estimate of the time required to complete the survey.	
***Risk of bias***		
**Item**	**Authors' judgement**	**Support for judgement**
Sequence generation	Unclear	Random allocation: method unspecified
Allocation concealment?	Unclear	Random allocation: method unspecified
Blinding of participants and personnel	Unclear	Blinding of personnel not described. Participants were not blinded to intervention
Blinding of outcome assessment	Yes	No outcome assessment used (counts of responses only)
Incomplete outcome data	Yes	Exclusions reported
Selective reporting	Yes	Outcomes reported in full
Other sources of bias	Yes	No grounds for concern about other sources of bias from reading the study report

**Table:** Kropf 2005

***Study characteristics***		
Methods	Random allocation: method not specified	
Data	Members of the Health Maintenance Organizations (HMOs) in Maryland	
Comparisons	1. $5 incentive 2. No incentive 3. Cover letter ‐ norms of co‐operation (answering the survey would help many other people) 4. Cover letter ‐ norms of self‐interest (co‐operation would help the survey respondent himself or herself)	
Outcomes	Response period not specified	
Topic	Health: healthcare and other current issues	
Mode of Administration	Postal	
Notes	—	
***Risk of bias***		
**Item**	**Authors' judgement**	**Support for judgement**
Allocation concealment?	Unclear	B ‐ unclear

**Table:** Kurth 1987

***Study characteristics***		
Methods	Random allocation: method not specified	
Data	Employees of the Maricopa Community Colleges with email accounts	
Comparisons	1. Type‐written; sensitive question 2. Type‐written; no sensitive question	
Outcomes	Response within 30 days	
Topic	Non‐health: supervisory management	
Mode of Administration	Postal	
Notes	—	
***Risk of bias***		
**Item**	**Authors' judgement**	**Support for judgement**
Allocation concealment?	Unclear	B ‐ unclear

**Table:** Kuskowska‐Wolk 1992

***Study characteristics***		
Methods	Random allocation: method not specified	
Data	Women aged 40‐70 years from 2 medium‐sized towns in Uppsala Health Care Region invited for mammography over the period 13 October 1986 to 20 March 1987	
Comparisons	1. Increasing order of food frequencies; no column on portion sizes; no extra page of questions 2. Decreasing order of food frequencies; no column on portion sizes; no extra page of questions 3. Increasing order of food frequencies; column on portion sizes; no extra page of questions 4. Decreasing order of food frequencies; column on portion sizes; no extra page of questions 5. Increasing order of food frequencies; no column on portion sizes; extra page of questions 6. Decreasing order of food frequencies; no column on portion sizes; extra page of questions 7. Increasing order of food frequencies; column on portion sizes; extra page of questions 8. Decreasing order of food frequencies; column on portion sizes; extra page of questions	
Outcomes	Response period not specified	
Topic	Health: Food Frequency Questionnaire	
Mode of Administration	Postal	
Notes	Age: 54.9‐55.6 years	
***Risk of bias***		
**Item**	**Authors' judgement**	**Support for judgement**
Allocation concealment?	Unclear	B ‐ unclear

**Table:** Kypri 2003

***Study characteristics***		
Methods	Random allocation: method not specified	
Data	Tertiary students at the University of Otago	
Comparisons	1. Ballpoint pen worth $0.50 2. Pen + cookie voucher worth $1 3. Pen + lunch voucher worth $5 4. Pen + lunch voucher worth $5 on completion of the survey	
Outcomes	Response after one week (1 reminder)	
Topic	Health (alcohol consumption)	
Mode of Administration	Electronic: Web survey	
Notes	Age: 16‐29 years; mainly females A personally addressed and signed letter on university letterhead was mailed to sampled students, inviting them to participate in a confidential Alcohol Use Survey via the Web. The letter notified the recipients that in 2 days time an email message would be sent to their student email address, and that a hypertext link contained in the message, when clicked, would open their computer web browser at the site hosting the survey. One week after the first email message was sent, the survey database was checked to determine whether the student had responded. A polite personalised reminder email was sent to students who had not yet responded. This also contained a hypertext link to the web questionnaire in case the previous email had been deleted.	
***Risk of bias***		
**Item**	**Authors' judgement**	**Support for judgement**
Sequence generation	Unclear	Random allocation: method not specified
Allocation concealment?	Unclear	Random allocation: method not specified
Blinding of participants and personnel	Unclear	Blinding of personnel not described. Participants were not blinded to intervention.
Blinding of outcome assessment	Yes	No outcomes were assessed (counts of responses only).
Incomplete outcome data	Yes	Exclusions were reported.
Selective reporting	Yes	Response after one week (1 reminder) reported in full
Other sources of bias	Yes	No grounds for concern about other sources of bias from reading the study report

**Table:** Kypri 2016

***Study characteristics***		
Methods	Random allocation: method unspecified	
Data	Individuals drawn at random from Maori electoral roll and individuals drawn at random from general NZ electoral roll	
Comparisons	Conditional non‐monetary incentive (entry into prize draw for a $500 supermarket voucher) vs. no incentive; conditional non‐monetary incentive ($5 donation to charity) vs. no incentive	
Outcomes	First response	
Topic	Health (alcohol consumption and problems)	
Mode of Administration	Postal	
Notes	8‐page booklet containing 44 questions requiring tickbox (or checkbox) or short‐text responses. Package not described	
***Risk of bias***		
**Item**	**Authors' judgement**	**Support for judgement**
Sequence generation	Unclear	Random allocation: method unspecified
Allocation concealment?	Unclear	Random allocation: method unspecified
Blinding of participants and personnel	Unclear	Blinding of personnel not described. Participants were not blinded to intervention.
Blinding of outcome assessment	Yes	No outcomes were assessed (counts of responses only).
Incomplete outcome data	Yes	Exclusions were reported.
Selective reporting	Yes	First response reported in full
Other sources of bias	Yes	No grounds for concern about other sources of bias from reading the study report

**Table:** La Garce 1995

***Study characteristics***		
Methods	Random allocation: method not specified	
Data	Goodyear tyre and rubber dealers	
Comparisons	1. Standard questionnaire printed in black and white 2. Standard questionnaire printed in blue and yellow 3. User‐friendly format questionnaire printed in black and white 4. User‐friendly format questionnaire printed in blue and yellow	
Outcomes	Response period not specified	
Topic	Non‐health: industry	
Mode of Administration	Postal	
Notes	—	
***Risk of bias***		
**Item**	**Authors' judgement**	**Support for judgement**
Allocation concealment?	Unclear	B ‐ unclear

**Table:** Labarere 2000

***Study characteristics***		
Methods	Random allocation: method not specified	
Data	400 people recently discharged from hospital	
Comparisons	1. Postal questionnaire, no follow‐up 2. Postal questionnaire with follow‐up	
Outcomes	Response within 2 months	
Topic	Health: patient satisfaction	
Mode of Administration	Postal	
Notes	—	
***Risk of bias***		
**Item**	**Authors' judgement**	**Support for judgement**
Allocation concealment?	Unclear	B ‐ unclear

**Table:** Labrecque 1978

***Study characteristics***		
Methods	Random allocation: method not specified	
Data	Service customers of a marina	
Comparisons	1. Owner's signature (OS); personalised (P); commemorative stamp (CS) 2. Service manager's signature (SMS); P; CS 3. OS; not personalised (NP); CS 4. SMS; NP; CS 5. OS; P; No CS 6. SMS; P; No CS 7. OS; NP; No CS 8. SMS; NP; No CS	
Outcomes	Response within 4 weeks	
Topic	Non‐health: reaction of customers to the performance of its service department	
Mode of Administration	Postal	
Notes	—	
***Risk of bias***		
**Item**	**Authors' judgement**	**Support for judgement**
Allocation concealment?	Unclear	B ‐ unclear

**Table:** Lagerros 2012

***Study characteristics***		
Methods	Random allocation: method unspecified	
Data	637 people from the population register at Statistics Sweden who had agreed to participate in a study on physical activity	
Comparisons	Postal vs. Web vs. SMS	
Outcomes	Final response after 1‐3 reminders	
Topic	Health (physical activity)	
Mode of Administration	Postal/Web/SMS	
Notes	4 questions (body weight, height and two questions on physical activity). In the postal group, the four questions were mailed to their home address. In the web group, an email was sent to the participant with a link to a website together with a username and a password. The participants were asked to log on to the website and answer the four questions. In the SMS group, the participant got one initial SMS explaining that she or he would soon receive the questions; after a few minutes, the four questions were sent in four separate SMS messages with 5 to 10 mins between them. All non‐responders in the three groups were reminded in a sequence that was considered appropriate for the technique. Thus, one reminder was sent out to the postal group (a letter after 3 weeks), three reminders were sent out to the web group (emails 2, 3 and 6 days after the first email, respectively), and two reminders were sent out to the SMS group (an SMS 1 and 2 days after the first SMS, respectively).	
***Risk of bias***		
**Item**	**Authors' judgement**	**Support for judgement**
Sequence generation	Unclear	Random allocation: method unspecified
Allocation concealment?	Unclear	Random allocation: method unspecified
Blinding of participants and personnel	Unclear	Blinding of personnel not described. Participants were not blinded to intervention.
Blinding of outcome assessment	Yes	No outcomes were assessed (counts of responses only).
Incomplete outcome data	Yes	Exclusions were reported.
Selective reporting	Yes	Outcomes reported in full
Other sources of bias	Yes	No grounds for concern about other sources of bias from reading the study report

**Table:** Langenderfer‐Magruder 2020

***Study characteristics***		
Methods	Random allocation: computer‐generated random numbers using Microsoft Excel	
Data	Non‐respondents to Wave 2 of the Florida Study of Professionals for Safe Families (a longitudinal study of child welfare workforce retention), US.	
Comparisons	SMS reminder including web‐survey link (condition 2) vs. SMS reminder without survey link (condition 1) vs. SMS reminder with offer of SMS including web‐survey link (condition 3)	
Outcomes	Response after 3 reminders (44 days)	
Topic	Health (worker personal or organisational characteristics that may impact job satisfaction and retention).	
Mode of Administration	Online survey	
Notes	45‐60 minutes to complete. Reminder messages were sent at 5 days and at 10 days for those who did not complete the survey. At 14 days, participants who had not yet opened the survey link were emailed to ensure that the survey emails were not sent to a spam folder. If there was no response to the individual email, project staff telephoned the non‐respondents. At 21 days, project staff attempted to email or call the non‐respondents. The publication also reports results of a second RCT with the same participants and is thus not independent of the first study; it has thus not been included in the 2023 review update.	
***Risk of bias***		
**Item**	**Authors' judgement**	**Support for judgement**
Sequence generation	Yes	Random allocation: computer‐generated random number using Microsoft Excel
Allocation concealment?	Yes	Random allocation: computer‐generated random number using Microsoft Excel
Blinding of participants and personnel	Unclear	Blinding of personnel not described. Participants were not blinded to intervention.
Blinding of outcome assessment	Yes	No outcomes were assessed (counts of responses only).
Incomplete outcome data	Yes	Exclusions reported
Selective reporting	Yes	Response after 3 reminders (44 days) reported in full
Other sources of bias	Yes	No grounds for concern about other sources of bias from reading the study report

**Table:** Lavelle 2008

***Study characteristics***		
Methods	Random allocation: computer generated using SPSS	
Data	Patients attending breast clinics in Greater Manchester between 1/10/2002 ‐ 31/7/2003	
Comparisons	1. First‐class stamp on addressed reply envelope 2. Pre‐paid addressed reply envelope	
Outcomes	Response period not specified	
Topic	Health: functional health status ‐ ELPHS ADL; generic health status ‐ SF‐12; health‐related quality of life ‐ EORTC QLQ‐C30	
Mode of Administration	Postal	
Notes	6 pages, package not described	
***Risk of bias***		
**Item**	**Authors' judgement**	**Support for judgement**
Sequence generation	Yes	Random allocation: computer‐generated
Allocation concealment?	Yes	Random allocation: computer‐generated
Blinding of participants and personnel	Unclear	Blinding of personnel not described. Participants were not blinded to intervention.
Blinding of outcome assessment	Yes	No outcomes were assessed (counts of responses only).
Incomplete outcome data	No	Exclusions were not reported.
Selective reporting	Yes	First response reported in full
Other sources of bias	Yes	No grounds for concern about other sources of bias from reading the study report

**Table:** Leece 2004

***Study characteristics***		
Methods	Quasi‐random allocation: alternately assigned members to receive a postal or an Internet questionnaire.	
Data	442 surgeon‐members of the Orthopaedic Trauma Association	
Comparisons	Postal vs. electronic	
Outcomes	Response after 22 weeks (2 reminders)	
Topic	Health (preferences and practice patterns of orthopaedic traumatologists in the operative treatment for femoral neck fractures)	
Mode of Administration	Postal and electronic	
Notes	8 pages. The Web questionnaire was 6 pages and took approximately 5 minutes to complete, and had 38 questions. Advanced notification by post (mail group) or email (Internet group) 2 to 5 days prior to receiving the survey; then a mailed copy of the survey, or an email with a link to the Internet survey; then another mailed copy or email with link to the survey at 6 weeks; then a further copy or link at 12 weeks; and finally a copy of the survey sent by mail only to all non‐respondents in both groups (22 weeks for the mail group and 19 weeks for the Internet group)	
***Risk of bias***		
**Item**	**Authors' judgement**	**Support for judgement**
Sequence generation	No	Quasi‐random allocation: alternately assigned members to receive a postal or an Internet questionnaire
Allocation concealment?	No	Quasi‐random allocation: alternately assigned members to receive a postal or an Internet questionnaire
Blinding of participants and personnel	Unclear	Blinding of personnel not described. Participants were not blinded to intervention.
Blinding of outcome assessment	Yes	No outcomes were assessed (counts of responses only).
Incomplete outcome data	Yes	Exclusions were reported.
Selective reporting	Yes	Response after 22 weeks (2 reminders) reported in full
Other sources of bias	Yes	No grounds for concern about other sources of bias from reading the study report

**Table:** Leece 2006a

***Study characteristics***		
Methods	Random allocation: using alternation	
Data	Surgeon members of the Orthopaedic Trauma Association	
Comparisons	1. Standard cover letter 2. Test cover letter (more personal)	
Outcomes	Response period 6 weeks	
Topic	Health: preferences in the treatment of femoral neck fractures	
Mode of Administration	Postal	
Notes	Method of allocation ascertained through contact with author	
***Risk of bias***		
**Item**	**Authors' judgement**	**Support for judgement**
Allocation concealment?	No	C ‐ inadequate

**Table:** Leece 2006b

***Study characteristics***		
Methods	Random allocation: using alternation	
Data	Surgeon members of the Orthopaedic Trauma Association	
Comparisons	1. Standard cover letter 2. Test cover letter (more personal)	
Outcomes	Response period 6 weeks	
Topic	Health: preferences in the treatment of femoral neck fractures	
Mode of Administration	Electronic: email	
Notes	Method of allocation ascertained through contact with author	
***Risk of bias***		
**Item**	**Authors' judgement**	**Support for judgement**
Allocation concealment?	No	C ‐ inadequate

**Table:** Leigh Brown 1997

***Study characteristics***		
Methods	Random allocation: method not specified	
Data	Patients who had already responded a questionnaire about hospital attendance	
Comparisons	1. Aware of monthly prize draw offering £25 gift voucher 2. Unaware of monthly prize draw offering £25 gift voucher	
Outcomes	Response period not specified	
Topic	Health: health status, satisfaction with orthopaedic referral	
Mode of Administration	Postal	
Notes	—	
***Risk of bias***		
**Item**	**Authors' judgement**	**Support for judgement**
Allocation concealment?	Unclear	B ‐ unclear

**Table:** Leung 2002

***Study characteristics***		
Methods	Random allocation: computerised random number generation	
Data	Physicians randomly selected from the full and limited registration lists of the Hong Kong Medical Council	
Comparisons	1. No incentive 2. $10 cash 3. $20 cash 4. $40 cash 5. Entry into $1000 lottery 6. Entry into $2000 lottery 7. Entry into $4000 lottery	
Outcomes	Response within 30 days	
Topic	Health: nature of practice, remuneration, clinical and administrative task	
Mode of Administration	Postal	
Notes	Mainly males	
***Risk of bias***		
**Item**	**Authors' judgement**	**Support for judgement**
Allocation concealment?	Yes	A ‐ adequate

**Table:** Leung 2004

***Study characteristics***		
Methods	Random allocation: computerised random number generation	
Data	Physicians randomly selected from the full and limited registration lists of the Hong Kong Medical Council	
Comparisons	1. Prepayment HK$20 2. Post‐payment HK$20	
Outcomes	Response within 60 days	
Topic	Health: computerisation of clinical and administrative tasks	
Mode of Administration	Postal	
Notes	—	
***Risk of bias***		
**Item**	**Authors' judgement**	**Support for judgement**
Sequence generation	Yes	Random allocation: computer‐generated
Allocation concealment?	Yes	Random allocation: computer‐generated
Blinding of participants and personnel	Unclear	Blinding of personnel not described. Participants were not blinded to intervention.
Blinding of outcome assessment	Yes	No outcomes were assessed (counts of responses only).
Incomplete outcome data	Yes	Exclusions were reported.
Selective reporting	Yes	Final response after 2 postal reminders and one telephone call reported in full
Other sources of bias	Yes	No grounds for concern about other sources of bias from reading the study report

**Table:** Levy 2012

***Study characteristics***		
Methods	Random allocation: method unspecified	
Data	800 dermatologists, US	
Comparisons	Personalised handwritten note on letter vs. no note Non‐monetary incentive (mint sweets) vs. no incentive Personalised reminder postcard vs. pre‐printed reminder postcard	
Outcomes	Final response after 6 weeks (2 reminders)	
Topic	Health (physician attitudes and beliefs surrounding dysplastic nevi)	
Mode of Administration	Postal	
Notes	4 pages. Letter and questionnaire initially sent. Reminder postcard sent 1 week later, and a second mailing of the questionnaire to those who had not yet responded approximately 6 weeks after the initial mailing. The initial cover letter included the recipient's name and address and a brief summary of the study, and it concluded with preprinted signatures of the investigators.	
***Risk of bias***		
**Item**	**Authors' judgement**	**Support for judgement**
Sequence generation	Unclear	Random allocation: method unspecified
Allocation concealment?	Unclear	Random allocation: method unspecified
Blinding of participants and personnel	Unclear	Blinding of personnel not described. Participants were not blinded to intervention
Blinding of outcome assessment	Yes	No outcome assessment used (counts of responses only)
Incomplete outcome data	Yes	Exclusions reported
Selective reporting	Yes	Outcomes reported in full
Other sources of bias	Yes	No grounds for concern about other sources of bias from reading the study report

**Table:** Lewis 2017

***Study characteristics***		
Methods	Random allocation: computer‐generated	
Data	611 participants who were due to be sent a 4‐month follow‐up questionnaire for the CASPER and CASPER SHARD trials	
Comparisons	Post‐it note with printed request to respond vs. no Post‐it note	
Outcomes	Response after 8 weeks (2 reminders: one postal, one telephone)	
Topic	Health (healthcare‐related study with older people suffering from depression)	
Mode of Administration	Postal	
Notes	PHQ‐9 (9 questions on 1 page). After 4 weeks, non‐respondents were sent a reminder letter and questionnaire; if no response, a follow‐up phone call was given after a further 4 weeks.	
***Risk of bias***		
**Item**	**Authors' judgement**	**Support for judgement**
Sequence generation	Yes	Random allocation: computer‐generated
Allocation concealment?	Yes	Random allocation: computer‐generated
Blinding of participants and personnel	Unclear	Blinding of personnel not described. Participants were not blinded to intervention.
Blinding of outcome assessment	Yes	No outcomes were assessed (counts of responses only).
Incomplete outcome data	Yes	Exclusions were reported.
Selective reporting	Yes	Response after 8 weeks (2 reminders: one postal, one telephone) reported in full
Other sources of bias	Yes	No grounds for concern about other sources of bias from reading the study report

**Table:** Lewis 2017a

***Study characteristics***		
Methods	Random allocation: method unspecified	
Data	Participants in the 2015 Federal Employee Viewpoint Survey, US	
Comparisons	Initial email invitation and all reminders sent on Tuesday morning ('traditional') vs. emails sent cycling around Tuesday p.m., Wednesday a.m., Wednesday p.m., Thursday a.m., Thursday p.m. ('rotating') vs. emails sent according to multinomial logistic regression model prediction of optimal day ('dynamic adaptive')	
Outcomes	Response after 6 reminder emails	
Topic	Non‐health (range of employee perceptions, attitudes, and behaviours, serving as a valuable tool for human resource managers to determine which aspects of an organisation are working well and which may require intervention)	
Mode of Administration	Web survey	
Notes	88‐item survey (20 demographic questions and 68 items that were grouped into eleven topic headings including personal work experience, leadership, employee satisfaction, work‐life, employment demographics). The data collection protocol begins by sending all sampled individuals an initial invitation to participate. Thereafter, non‐respondents are sent weekly reminder emails. These notifications are typically sent on Tuesday mornings.	
***Risk of bias***		
**Item**	**Authors' judgement**	**Support for judgement**
Sequence generation	Unclear	Random allocation: method unspecified
Allocation concealment?	Unclear	Random allocation: method unspecified
Blinding of participants and personnel	Unclear	Blinding of personnel not described. Participants were not blinded to intervention
Blinding of outcome assessment	Yes	No outcome assessment used (counts of responses only)
Incomplete outcome data	Yes	Exclusions were reported.
Selective reporting	Yes	Outcomes reported in full
Other sources of bias	Yes	No grounds for concern about other sources of bias from reading the study report

**Table:** Linsky 1965

***Study characteristics***		
Methods	Random allocation: method not specified	
Data	Nurses	
Comparisons	1. Characteristics of cover letter: personalised (P); social utility appeal (SU); explanation of place and importance of respondent in study (RP); an appeal to help researchers of study (HR) 2. Not P; SU; RP; HR 3. P; Not SU; RP; HR 4. Not P; Not SU; RP; HR 5. P; SU; Not RP; HR 6. Not P; SU; Not RP; HR 7. P; SU; RP; Not HR 8. Not P; SU; RP; Not HR 9. P; Not SU; Not RP; HR 10. Not P; Not SU; Not RP; HR 11. P; Not SU; RP; Not HR 12. Not P; Not SU; RP; Not HR 13. P; SU; Not RP; Not HR 14. Not P; SU, Not RP; Not HR 15. P; Not SU; Not RP; Not HR 16. Not P; Not SU; Not RP; Not HR	
Outcomes	Response within 3 weeks	
Topic	Health: not specified	
Mode of Administration	Postal	
Notes	—	
***Risk of bias***		
**Item**	**Authors' judgement**	**Support for judgement**
Allocation concealment?	Unclear	B ‐ unclear

**Table:** Little 1990

***Study characteristics***		
Methods	Random allocation: using alternation	
Data	Respondents to a national magazine clip ad promotion	
Comparisons	1. 25 cents 2. Pan‐scrapper 3. Control	
Outcomes	Response period not specified	
Topic	Non‐health: perceptions of the product and follow‐up service to competing products	
Mode of Administration	Postal	
Notes	—	
***Risk of bias***		
**Item**	**Authors' judgement**	**Support for judgement**
Allocation concealment?	No	C ‐ inadequate

**Table:** Loban 2017

***Study characteristics***		
Methods	Random allocation: method unspecified	
Data	Participants in the second wave of a cohort study on health and well‐being amongst the population of Yorkshire, UK	
Comparisons	Electronic with postal follow‐up vs. postal with electronic follow‐up	
Outcomes	Response after one month (one reminder)	
Topic	Health (general health and well‐being)	
Mode of Administration	Postal and electronic	
Notes	8‐page questionnaire The electronic completion arm was contacted by email with a URL to complete the questionnaire online. If no responses were received within a month, a questionnaire containing the same questions was sent by post; the control group initially received an identical questionnaire by post; if after a month no response had been obtained, a reminder was sent via email with a URL to complete the questionnaire online.	
***Risk of bias***		
**Item**	**Authors' judgement**	**Support for judgement**
Sequence generation	Unclear	Random allocation: method unspecified
Allocation concealment?	Unclear	Random allocation: method unspecified
Blinding of participants and personnel	Unclear	Blinding of personnel not described. Participants were not blinded to intervention.
Blinding of outcome assessment	Yes	No outcomes were assessed (counts of responses only).
Incomplete outcome data	Yes	Exclusions were reported.
Selective reporting	Yes	Response after one month (one reminder) reported in full
Other sources of bias	Yes	No grounds for concern about other sources of bias from reading the study report

**Table:** London 1990a

***Study characteristics***		
Methods	Random allocation: method not specified	
Data	Electronics design engineers	
Comparisons	1. Standard cover letter 2. As (1) but also told would be entered into a prize draw for 3 calculators if responded 3. As (2) but also told that all respondents would receive a special gift from the sponsor	
Outcomes	Response period not specified	
Topic	Non‐health: potential customers needs, firm usage, sources of transformers and inductors	
Mode of Administration	Postal	
Notes	—	
***Risk of bias***		
**Item**	**Authors' judgement**	**Support for judgement**
Allocation concealment?	Unclear	B ‐ unclear

**Table:** London 1990b

***Study characteristics***		
Methods	Random allocation: method not specified	
Data	Electronics design engineers	
Comparisons	1. No incentive 2. $1 incentive	
Outcomes	Response period not specified	
Topic	Non‐health: potential customers needs, firm usage, sources of transformers and inductors	
Mode of Administration	Postal	
Notes	—	
***Risk of bias***		
**Item**	**Authors' judgement**	**Support for judgement**
Allocation concealment?	Unclear	B ‐ unclear

**Table:** Lorenzi 1988

***Study characteristics***		
Methods	Random allocation: methods not specified	
Data	Business executives, state legislators, and director of chambers of commerce from the Midwestern United States	
Comparisons	1. Unconditional $1 payoff 2. Conditional $2 payoff 3. Lottery to win $50, $30, or $20 4. No incentives	
Outcomes	Response within 14 days	
Topic	Non‐health: finance ‐ financial investment scenario, behavioural self‐description measure of propensity for risk in personal and business investments	
Mode of Administration	Postal	
Notes	Mean age: 49.7 years	
***Risk of bias***		
**Item**	**Authors' judgement**	**Support for judgement**
Allocation concealment?	Unclear	B ‐ unclear

**Table:** Lund 1998

***Study characteristics***		
Methods	Random allocation: method not specified	
Data	Norwegian women aged 34‐49 years	
Comparisons	1. Questionnaire entitled 'Women Lifestyle & Health'; 4 pages 2. Questionnaire entitled "Women & Cancer"; 2 pages 3. Questionnaire entitled "Women & Cancer"; 4 pages 4. Questionnaire entitled "Women & Cancer"; 6 pages 5. Questionnaire entitled "Oral Contraceptives & Cancer"; 2 pages	
Outcomes	Response period not specified	
Topic	Health: diet, sunbathing habits, occupational exposure, pharmaceutical drugs	
Mode of Administration	Postal	
Notes	—	
***Risk of bias***		
**Item**	**Authors' judgement**	**Support for judgement**
Allocation concealment?	Unclear	B ‐ unclear

**Table:** Lusinchi 2007

***Study characteristics***		
Methods	Random allocation: method unspecified	
Data	601 electronics engineers and engineering managers, US	
Comparisons	Pre‐notification email vs. none Scrollable web page vs. multiple web pages	
Outcomes	Final response after 3 weeks (3 reminders)	
Topic	Non‐health (integrated circuit design)	
Mode of Administration	Web survey	
Notes	26 questions. Emails sent, followed by three reminders: the first was sent two days after the beginning of the field period; the second was sent four days after the first; and the third was sent a week after the second reminder. The scroll‐type version had 2 pages: the home page and the questionnaire page; the multiple‐page version consisted of 30 pages, including the home page and the contact information page.	
***Risk of bias***		
**Item**	**Authors' judgement**	**Support for judgement**
Sequence generation	Unclear	Random allocation: method unspecified
Allocation concealment?	Unclear	Random allocation: method unspecified
Blinding of participants and personnel	Unclear	Blinding of personnel not described. Participants were not blinded to intervention.
Blinding of outcome assessment	Yes	No outcome assessment used (counts of responses only)
Incomplete outcome data	Yes	Exclusions reported
Selective reporting	Yes	Outcomes reported in full
Other sources of bias	Yes	No grounds for concern about other sources of bias from reading the study report

**Table:** MacLennan 2013

***Study characteristics***		
Methods	Random allocation: method unspecified	
Data	Patients with type 2 diabetes participating in the RECORD trial who had not responded to the one‐year follow‐up questionnaire	
Comparisons	Pre‐notification telephone call vs. no pre‐notification	
Outcomes	Response within 21 days	
Topic	Health (diabetes)	
Mode of Administration	Postal	
Notes	Questionnaire not described. Package not described	
***Risk of bias***		
**Item**	**Authors' judgement**	**Support for judgement**
Sequence generation	Unclear	Random allocation: method unspecified
Allocation concealment?	Unclear	Random allocation: method unspecified
Blinding of participants and personnel	Unclear	Blinding of personnel not described. Participants were not blinded to intervention.
Blinding of outcome assessment	Yes	No outcomes were assessed (counts of responses only).
Incomplete outcome data	No	Exclusions were not reported.
Selective reporting	Yes	Response within 21 days reported in full
Other sources of bias	Yes	No grounds for concern about other sources of bias from reading the study report

**Table:** Maheux 1989a

***Study characteristics***		
Methods	Random allocation: method not specified	
Data	Physicians who had failed to respond to a previous questionnaire (Quebec, UK)	
Comparisons	1. Follow‐up letter with handwritten postscript 2. Follow‐up letter with no postscript	
Outcomes	Response within 6 months	
Topic	Health: support for patient care issues	
Mode of Administration	Postal	
Notes	—	
***Risk of bias***		
**Item**	**Authors' judgement**	**Support for judgement**
Allocation concealment?	Unclear	B ‐ unclear

**Table:** Maheux 1989b

***Study characteristics***		
Methods	Random allocation: method not specified	
Data	Physicians who had failed to respond to 2 mailings of a questionnaire (Quebec, UK)	
Comparisons	1. Personalised mailout package 2. Non‐personalised mailout package	
Outcomes	Response within 6 months	
Topic	Health: support for patient care issues	
Mode of Administration	Postal	
Notes	—	
***Risk of bias***		
**Item**	**Authors' judgement**	**Support for judgement**
Allocation concealment?	Unclear	B ‐ unclear

**Table:** Mallen 2008

***Study characteristics***		
Methods	Random allocation: computer‐generated	
Data	Patients aged 50 and over from the Central Cheshire general practices who consulted their GP for non‐inflammatory musculoskeletal pain between September 2006–April 2007	
Comparisons	1. Small font size ‐ Arial 12 2. Large font size ‐ Arial 16 3. Thin paper ‐ 80 g 4. Thick paper ‐ 100 g	
Outcomes	Response period not specified	
Topic	Health: prognosis of older people with joint pain in general practice	
Mode of Administration	Postal	
Notes	7 questions. Questionnaire booklets A4‐sized, mailed first class in white A4‐sized envelope. Participants received identical information sheets and a prepaid envelope to return their questionnaire. Those not responding to the initial questionnaire were sent a reminder postcard at 2 weeks and a further copy of the same questionnaire at 4 weeks. 2 x 2 factorial design based on two different font sizes (Arial size 12 [small font] vs. Arial size 16 [largefont]), and two paper thicknesses (80 g [thin paper] vs.100 g [thick paper])	
***Risk of bias***		
**Item**	**Authors' judgement**	**Support for judgement**
Sequence generation	Yes	Random allocation: computer‐generated
Allocation concealment?	Yes	Random allocation: computer‐generated
Blinding of participants and personnel	Unclear	Blinding of personnel not described. Participants were not blinded to intervention.
Blinding of outcome assessment	Yes	No outcome assessment used (counts of responses only)
Incomplete outcome data	Yes	Exclusions reported
Selective reporting	Yes	Outcomes reported in full
Other sources of bias	Yes	No grounds for concern about other sources of bias from reading the study report

**Table:** Man 2011

***Study characteristics***		
Methods	Random allocation: computer‐generated	
Data	Participants in an RCT investigating the role of yoga for the treatment of lower back pain	
Comparisons	Electronic prompt (email or SMS) vs. no prompt	
Outcomes	Response after 42 days (2 reminders)	
Topic	Health (yoga for the treatment of lower back pain)	
Mode of Administration	Postal	
Notes	20 pages. Questionnaire with instructions to complete and return using the SAE. All participants also received £5 as an incentive to complete the questionnaire. Non‐responders were sent reminder letters after 2 and 4 weeks, up to a total trial follow‐up period of 42 days.	
***Risk of bias***		
**Item**	**Authors' judgement**	**Support for judgement**
Sequence generation	Yes	Random allocation: computer‐generated
Allocation concealment?	Yes	Random allocation: computer‐generated
Blinding of participants and personnel	Unclear	Blinding of personnel not described. Participants were not blinded to intervention.
Blinding of outcome assessment	Yes	No outcomes were assessed (counts of responses only).
Incomplete outcome data	No	Exclusions were not reported.
Selective reporting	Yes	Response after 42 days (2 reminders) reported in full
Other sources of bias	Yes	No grounds for concern about other sources of bias from reading the study report

**Table:** Mann 2005

***Study characteristics***		
Methods	Random allocation: method not specified	
Data	Registered voters in Maryland, New York, and Pennsylvania	
Comparisons	1. Advance letter 2. No advance letter	
Outcomes	Response period not specified	
Topic	Non‐health: voting behaviour	
Mode of Administration	Postal	
Notes	—	
***Risk of bias***		
**Item**	**Authors' judgement**	**Support for judgement**
Allocation concealment?	Unclear	B ‐ unclear

**Table:** Mann 2008

***Study characteristics***		
Methods	Random allocation: method unspecified	
Data	Parents of 292 young adults eligible for the Hutchinson Smoking Prevention Project, Washington, US	
Comparisons	Smaller unconditional monetary incentive ($1) vs. larger unconditional monetary incentive ($2) vs. no incentive	
Outcomes	First and final responses (after reminder mailings and phone calls)	
Topic	Health (smoking)	
Mode of Administration	Postal	
Notes	Questionnaire not described. Used a mixed‐mode procedure involving multiple mailings and telephone follow‐up of non‐responders	
***Risk of bias***		
**Item**	**Authors' judgement**	**Support for judgement**
Sequence generation	Unclear	Random allocation: method unspecified
Allocation concealment?	Unclear	Random allocation: method unspecified
Blinding of participants and personnel	Unclear	Blinding of personnel not described. Participants were not blinded to intervention.
Blinding of outcome assessment	Yes	No outcomes were assessed (counts of responses only).
Incomplete outcome data	No	Exclusions were not reported.
Selective reporting	Yes	First and final responses (after reminder mailings and phone calls) reported in full
Other sources of bias	Yes	No grounds for concern about other sources of bias from reading the study report

**Table:** Marcus 2007

***Study characteristics***		
Methods	Random allocation: using random number generation in SPSS	
Data	Owners of personal websites	
Comparisons	1. High topic salience ‐ motives and personality of personal website owners 2. Low topic salience ‐ psychological aspects of Internet usage 3. Long survey ‐ 359 items, 30‐60 minutes for completion 4. Short survey ‐ 91 items, 10‐20 minutes for completion 5. Lottery to win 2 Internet book store vouchers of 25 euro each 6. No lottery 7. Personalised feedback of the results (individual profile of the results) 8. Generalised (study results) or no feedback	
Outcomes	Response period not specified	
Topic	Non‐health: Internet competence	
Mode of Administration	Electronic: Web survey	
Notes	Method of allocation ascertained through contact with author	
***Risk of bias***		
**Item**	**Authors' judgement**	**Support for judgement**
Allocation concealment?	Yes	A ‐ adequate

**Table:** Marrett 1992

***Study characteristics***		
Methods	Random allocation: method not specified	
Data	Residents with histologically confirmed renal cell carcinoma (Ontario, US)	
Comparisons	1. Lottery ticket incentive 2. No incentive	
Outcomes	—	
Topic	Health: history of urinary tract infection, use of analgesic and diuretic medication, demographics	
Mode of Administration	Postal	
Notes	Allocation was not concealed from the person sending out the letters; age: 25‐69 years	
***Risk of bias***		
**Item**	**Authors' judgement**	**Support for judgement**
Allocation concealment?	No	C ‐ inadequate

**Table:** Marsh 1999

***Study characteristics***		
Methods	Random allocation: alternation	
Data	Parents of children aged 3‐12 months from general practices involved in a cluster‐randomised trial in Nottingham, UK who had responded to a previous questionnaire	
Comparisons	1. Promise of a £2 voucher for a local children's store on return of the questionnaire (postal) 2. No incentive (postal)	
Outcomes	Response period not specified	
Topic	Health: near miss and minor injuries	
Mode of Administration	Postal	
Notes	—	
***Risk of bias***		
**Item**	**Authors' judgement**	**Support for judgement**
Allocation concealment?	No	C ‐ inadequate

**Table:** Martin 1970

***Study characteristics***		
Methods	Random allocation: method not specified	
Data	Individuals listed in a telephone directory (Washington, US)	
Comparisons	1. Personalised letter (PL); appeal to importance (AI); commemorative stamp (CS); easy questionnaire (EQ) first 2. PL; AI; CS; EQ not first 3. PL; AI; business reply frank (BRF) instead of CS 4. PL; AI; BRF; EQ not first 5. PL; no AI; CS; EQ first 6. PL; no AI; CS; EQ not first 7. PL; no AI; BRF; EQ first 8. PL; no AI; BRF; EQ not first 9. Letter not personalised (NPL); AI; CS; EQ first 10. NPL; AI; CS; EQ not first 11. NPL; AI; BRF; EQ first 12. NPL; AI; BRF; EQ not first 13. NPL; no AI; CS; EQ first 14. NPL; no AI; CS; EQ not first 15. NPL; no AI; BRF; EQ first 16. NPL; no AI; BRF; EQ not first	
Outcomes	Response within 4 weeks	
Topic	Non‐health: public and judicial attitudes toward various aspects of the legal machinery	
Mode of Administration	Postal	
Notes	—	
***Risk of bias***		
**Item**	**Authors' judgement**	**Support for judgement**
Allocation concealment?	Unclear	B ‐ unclear

**Table:** Martin 1989

***Study characteristics***		
Methods	Random allocation: method not specified	
Data	University students	
Comparisons	1. Pre‐notification (PN); Follow up (FU); Personalised (PS); Stamped reply envelope (SRE) 2. PN; no FU; PS; SRE 3. PN; FU; no PS; SRE 4. PN; no FU; no PS; SRE 5. PN; FU; PS; business reply envelope (BRE) 6. PN; no FU; PS; BRE 7. PN; FU; no PS; BRE 8. PN; no FU; no PS; BRE 9. No PN; FU; PS; SRE 10. No PN; no FU; PS; SRE 11. No PN; FU; no PS; SRE 12. No PN; no FU; no PS; SRE 13. No PN; FU; PS; BRE 14. No PN; no FU; PS; BRE 15. No PN; FU; no PS; BRE 16. No PN; no FU; no PS; BRE	
Outcomes	Response period not specified	
Topic	Non‐health: information on the perceived attributes of the university	
Mode of Administration	Postal	
Notes	Mean age: 30 years	
***Risk of bias***		
**Item**	**Authors' judgement**	**Support for judgement**
Allocation concealment?	Unclear	B ‐ unclear

**Table:** Martin 1994

***Study characteristics***		
Methods	Random allocation: method not specified	
Data	Participants of a large international amateur bowling tournament	
Comparisons	1. High‐interest questionnaire 2. Low‐interest questionnaire	
Outcomes	Response period not specified	
Topic	Non‐health: interpersonal relationships with other customers in service environment	
Mode of Administration	Postal	
Notes	—	
***Risk of bias***		
**Item**	**Authors' judgement**	**Support for judgement**
Allocation concealment?	Unclear	B ‐ unclear

**Table:** Martinson 2000

***Study characteristics***		
Methods	Random allocation: method not specified	
Data	Adolescents aged 14‐17 years in the Minneapolis/St Paul metropolitan area	
Comparisons	1. $2 included with questionnaire 2. $15 promised on completion and return of questionnaire 3. Promise of entry into 10 drawings for 10 $200 cash prizes on completion and return of questionnaire 4. No incentive	
Outcomes	—	
Topic	Health: attitudes towards smoking, behavioural health‐related items	
Mode of Administration	Postal	
Notes	Reminder and second questionnaire sent to non‐responders 1 week and 3 weeks after initial mailing, respectively; age: 14‐17 years; equal male and females	
***Risk of bias***		
**Item**	**Authors' judgement**	**Support for judgement**
Allocation concealment?	Unclear	B ‐ unclear

**Table:** Mason 1961

***Study characteristics***		
Methods	Random allocation: alternation	
Data	Teachers	
Comparisons	1. Long form; name and address on form 2. Long form; code number on form 3. Short form; name and address on form 4. Short form; code number on form	
Outcomes	—	
Topic	Not specified	
Mode of Administration	Postal	
Notes	—	
***Risk of bias***		
**Item**	**Authors' judgement**	**Support for judgement**
Allocation concealment?	No	C ‐ inadequate

**Table:** Matteson 1974

***Study characteristics***		
Methods	Random allocation: method not specified	
Data	Members of a national organisation	
Comparisons	1. Semi‐personalised letter; white questionnaire 2. Semi‐personalised letter; pink questionnaire 3. Form letter; white questionnaire 4. Form letter; pink questionnaire	
Outcomes	Response period not specified	
Topic	Non‐health: significant contribution of literature in their field	
Mode of Administration	Postal	
Notes	—	
***Risk of bias***		
**Item**	**Authors' judgement**	**Support for judgement**
Allocation concealment?	Unclear	B ‐ unclear

**Table:** Mauz 2018

***Study characteristics***		
Methods	Random allocation: method unspecified	
Data	Children and adolescents registered in the local resident registries of 20 municipalities in 5 federal states of Germany, participating in the third wave of the 'German Health Interview and Examination Survey for Children and Adolescents'	
Comparisons	Choice (electronic/postal) vs. only postal response; postal vs. electronic; pre‐selected choice vs. choice (electronic/postal)	
Outcomes	Response after 1 reminder	
Topic	Health (physical and mental health as well as parent‐ or self‐reported information regarding the subjective health status, health behaviour, health care utilisation, social and migration status, living conditions, and environmental determinants of health).	
Mode of Administration	Postal/electronic	
Notes	Sociodemographic characteristics and a broad range of health indicators for children and adolescents were compared by survey design. Individuals mailed invitations. They were sent a cover letter with the invitation to participate, information about the study and data privacy, and an informed consent form. Depending on the allocated mode, the invitation comprised a username and password for participation through the Web option, along with a paper questionnaire for those allocated to the concurrent mixed‐mode design, only a paper questionnaire in the single‐mode design, or only the access data for the online questionnaire in the sequential mixed‐mode design. The SAQ‐Web questionnaire was only optimised for desktop computers. A reminder was sent by mail to respondents who had not replied within 3 weeks of the initial invitation. Participants who did not respond to the reminder were telephoned up to 5 times 4 weeks after the initial invitation. As an additional motivation for prospective participants, each parent and adolescent who had completed a questionnaire received a shopping voucher to the value of EU 10.	
***Risk of bias***		
**Item**	**Authors' judgement**	**Support for judgement**
Sequence generation	Unclear	Random allocation: method unspecified
Allocation concealment?	Unclear	Random allocation: method unspecified
Blinding of participants and personnel	Unclear	Blinding of personnel not described. Participants were not blinded to intervention.
Blinding of outcome assessment	Yes	No outcome assessment used (counts of responses only)
Incomplete outcome data	No	Exclusions not reported
Selective reporting	Yes	Outcomes reported in full
Other sources of bias	Yes	No grounds for concern about other sources of bias from reading the study report

**Table:** Maxwell 2009

***Study characteristics***		
Methods	Random allocation: method unspecified	
Data	Population‐based sample of colorectal cancer cases and their first‐degree relatives	
Comparisons	Monetary incentive vs. no incentive; non‐monetary incentive vs. no incentive; certified/special delivery vs. regular outward mailing	
Outcomes	Final response after telephone reminders	
Topic	Health (colorectal cancer screening)	
Mode of Administration	Postal	
Notes	Questionnaire not described. Initial recruitment of both cases and relatives was conducted via mail, followed by up to 10 telephone attempts if no response was received within 10 days.	
***Risk of bias***		
**Item**	**Authors' judgement**	**Support for judgement**
Sequence generation	Unclear	Random allocation: method unspecified
Allocation concealment?	Unclear	Random allocation: method unspecified
Blinding of participants and personnel	No	Although participants were unaware of incentive structures for other invitees, interviewers who did the telephone follow‐up attempts were aware of the incentive status of all subjects.
Blinding of outcome assessment	Yes	No outcomes were assessed (counts of responses only).
Incomplete outcome data	No	Exclusions were not reported.
Selective reporting	Yes	Final response after telephone reminders reported in full
Other sources of bias	Yes	No grounds for concern about other sources of bias from reading the study report

**Table:** McCaffery 2019

***Study characteristics***		
Methods	Random allocation: computer‐generated	
Data	Potential elderly participants in the OTIS trial of falls prevention, UK	
Comparisons	Handwritten name vs. printed name	
Outcomes	First response	
Topic	Health (screening questionnaire for the OTIS trial of falls prevention)	
Mode of Administration	Postal	
Notes	2 pages, 8 questions. Package not described	
***Risk of bias***		
**Item**	**Authors' judgement**	**Support for judgement**
Sequence generation	Yes	Random allocation: computer‐generated
Allocation concealment?	Yes	Random allocation: computer‐generated
Blinding of participants and personnel	Unclear	Blinding of personnel not described. Participants were not blinded to intervention.
Blinding of outcome assessment	Yes	No outcomes were assessed (counts of responses only).
Incomplete outcome data	No	Exclusions were not reported.
Selective reporting	Yes	First response reported in full
Other sources of bias	Yes	No grounds for concern about other sources of bias from reading the study report

**Table:** McCambridge 2011

***Study characteristics***		
Methods	Random allocation: computer‐generated	
Data	Participants in the DYD‐RCT (Down Your Drink), a large trial of online intervention to help hazardous drinkers reduce their alcohol consumption	
Comparisons	Longer questionnaire vs. shorter; more salience/relevant (to alcohol problems) vs. less (mental health)	
Outcomes	Response within 40 days (after 3 reminders)	
Topic	Health (alcohol consumption)	
Mode of Administration	Electronic	
Notes	23 questions (longer); 10 questions (shorter). Participants were sent email requests for follow‐up data after 1 and 3 months (study 1) and after 3 and 12 months (study 2). Up to three reminders were sent at 7‐day intervals to non‐responders. Study 1, 1‐month time point, selected for inclusion in review Hypotheses: (1) longer questionnaires (23 or 34 versus 10 items included in secondary outcome measures) will produce lower rates of follow‐up and (2) more relevant questionnaires (defined as assessing alcohol problems rather than mental health)	
***Risk of bias***		
**Item**	**Authors' judgement**	**Support for judgement**
Sequence generation	Yes	Random allocation: computer‐generated
Allocation concealment?	Yes	Random allocation: computer‐generated
Blinding of participants and personnel	Yes	Blinding of personnel not described. Participants answered two questions on confidence and intentions before arriving at a final questionnaire prior to being told their parent trial group allocation. Without their knowledge, participants had been randomly allocated to one of four different questionnaires (described below) to be completed as this final questionnaire. Participants were thus blinded to the conduct of this study.
Blinding of outcome assessment	Yes	No outcomes were assessed (counts of responses only).
Incomplete outcome data	No	Exclusions were not reported.
Selective reporting	Yes	Response within 40 days (after 3 reminders) reported in full
Other sources of bias	Yes	No grounds for concern about other sources of bias from reading the study report

**Table:** McColl 2003a

***Study characteristics***		
Methods	Random allocation: method not specified	
Data	Adults with angina from 62 family practices in Northeast England	
Comparisons	1. Version 1: Condition‐specific questionnaires (Seattle Angina Questionnaire) first, followed by generic questionnaires (SF‐36 & EQ‐5D) 2. Version 2: Generic questionnaires first, followed by condition‐specific questionnaires	
Outcomes	Response period not specified	
Topic	Health: patient‐based outcome measures	
Mode of Administration	Postal	
Notes	Reminder and second questionnaire sent to non‐responders 3 weeks and 6 weeks after initial mailing, respectively; mean age: 69.1 years; mainly male	
***Risk of bias***		
**Item**	**Authors' judgement**	**Support for judgement**
Allocation concealment?	Unclear	B ‐ unclear

**Table:** McColl 2003b

***Study characteristics***		
Methods	Random allocation: method not specified	
Data	Adults with asthma from 62 family practices in Northeast England	
Comparisons	1. Version 1: Condition‐specific questionnaires (Newcastle Asthma Symptoms Questionnaire & Asthma Quality of Life Questionnaire) first, followed by generic questionnaires (SF‐36 & EQ‐5D) 2. Version 2: Generic questionnaires first, followed by condition‐specific questionnaires	
Outcomes	Response period not specified	
Topic	Health: patient‐based outcome measures	
Mode of Administration	Postal	
Notes	Reminder and second questionnaire sent to non‐responders 3 weeks and 6 weeks after initial mailing, respectively; mean age: 48.6 years; mainly female	
***Risk of bias***		
**Item**	**Authors' judgement**	**Support for judgement**
Allocation concealment?	Unclear	B ‐ unclear

**Table:** McConochie 1985

***Study characteristics***		
Methods	Random allocation: method not specified	
Data	Males aged 18‐34	
Comparisons	1. 50 cents incentive 2. $2 incentive 3. $5 incentive	
Outcomes	Response within 1 week	
Topic	Non‐health: measurement of radio listening	
Mode of Administration	Postal	
Notes	Age: 18‐34 years; mainly males	
***Risk of bias***		
**Item**	**Authors' judgement**	**Support for judgement**
Allocation concealment?	Unclear	B ‐ unclear

**Table:** McCormack 2013

***Study characteristics***		
Methods	Random allocation: method unspecified	
Data	520 rural women followed by the South Dakota State University Study Call Center regarding eligibility for the National Children's Study	
Comparisons	Unconditional monetary incentive ($2) vs. no incentive	
Outcomes	Response within 75 days	
Topic	Health (pregnancy status and probability of becoming pregnant)	
Mode of Administration	Postal	
Notes	One page. Questionnaire sent with a business‐reply paid envelope	
***Risk of bias***		
**Item**	**Authors' judgement**	**Support for judgement**
Sequence generation	Unclear	Random allocation: method unspecified
Allocation concealment?	Unclear	Random allocation: method unspecified
Blinding of participants and personnel	Unclear	Blinding of personnel not described. Participants were not blinded to intervention.
Blinding of outcome assessment	Yes	No outcomes were assessed (counts of responses only).
Incomplete outcome data	Yes	Exclusions were reported.
Selective reporting	Yes	Response within 75 days reported in full
Other sources of bias	Yes	No grounds for concern about other sources of bias from reading the study report

**Table:** McCoy 2007

***Study characteristics***		
Methods	Random allocation: using alternation	
Data	Institute of Public Relations (IPR) members in Northern Ireland	
Comparisons	1. Handwritten address 2. Computer‐printed address 3. Brown envelope 4. White envelope	
Outcomes	Deadline for return provided	
Topic	Non‐health: practices and attitudes towards public relations evaluation	
Mode of Administration	Postal	
Notes	Method of allocation ascertained through contact with author	
***Risk of bias***		
**Item**	**Authors' judgement**	**Support for judgement**
Sequence generation	No	Random allocation: using alternation
Allocation concealment?	No	Random allocation: using alternation
Blinding of participants and personnel	Unclear	Blinding of personnel not described. Participants were not blinded to intervention.
Blinding of outcome assessment	Yes	No outcomes were assessed (counts of responses only).
Incomplete outcome data	No	Exclusions were not reported.
Selective reporting	Yes	Final response after one reminder reported in full
Other sources of bias	Yes	No grounds for concern about other sources of bias from reading the study report

**Table:** McDaniel 1980

***Study characteristics***		
Methods	Random allocation: using alternation	
Data	Major‐appliance purchasers	
Comparisons	1. 25‐cent incentive 2. No incentive	
Outcomes	Response period not specified	
Topic	Non‐health: product warranty questionnaire	
Mode of Administration	Postal	
Notes	—	
***Risk of bias***		
**Item**	**Authors' judgement**	**Support for judgement**
Allocation concealment?	Unclear	B ‐ unclear

**Table:** McDaniel 1981

***Study characteristics***		
Methods	Random allocation: using alternation	
Data	Major‐appliance purchasers in Midwestern US	
Comparisons	1. Anonymous (no name requested and no name given) 2. Non‐anonymous (name requested and given at the beginning of questionnaire)	
Outcomes	Response period not specified	
Topic	Non‐health: major appliance warranties and warranty performance	
Mode of Administration	Postal	
Notes	—	
***Risk of bias***		
**Item**	**Authors' judgement**	**Support for judgement**
Allocation concealment?	Unclear	B ‐ unclear

**Table:** McGonagle 2017

***Study characteristics***		
Methods	Random allocation: method unspecified	
Data	2473 non‐respondents in the U.S. Panel Study of Income Dynamics	
Comparisons	Larger monetary incentive (US $10) vs. smaller monetary incentive (US $5) vs. no incentive	
Outcomes	First response	
Topic	Non‐health (childhood experiences)	
Mode of Administration	Web or choice	
Notes	20 minutes (292 items) For both web and choice groups, the study invitation included the web address of the survey and provided login credentials that were unique and randomly generated. The invitation letter sent to the choice group also stated that the survey could be completed on a paper questionnaire that would be mailed to their address after two weeks. To encourage the use of the web to complete the survey, no mention of a paper questionnaire was made in the initial invitation letter sent to the web group. In the incentive treatment conditions, a US bill in the amount of US $5 or US $10 was paper‐clipped to the top of the paper questionnaire to ensure visibility.	
***Risk of bias***		
**Item**	**Authors' judgement**	**Support for judgement**
Sequence generation	Unclear	Random allocation: method unspecified
Allocation concealment?	Unclear	Random allocation: method unspecified
Blinding of participants and personnel	Unclear	Blinding of personnel not described. Participants were not blinded to intervention.
Blinding of outcome assessment	Yes	No outcomes were assessed (counts of responses only).
Incomplete outcome data	No	Exclusions were not reported.
Selective reporting	Yes	First response reported in full
Other sources of bias	Yes	No grounds for concern about other sources of bias from reading the study report

**Table:** McKee 1992

***Study characteristics***		
Methods	Random allocation: method not specified	
Data	Members of a national non‐profit professional organisation	
Comparisons	1. Coded 2. Not coded In coded group, only non‐respondents received follow‐up. In non‐coded group, all received follow‐up.	
Outcomes	Response period not specified	
Topic	Non‐health: programme of the organisation	
Mode of Administration	Postal	
Notes	—	
***Risk of bias***		
**Item**	**Authors' judgement**	**Support for judgement**
Allocation concealment?	Unclear	B ‐ unclear

**Table:** McKenzie‐McHarg 2005

***Study characteristics***		
Methods	Random allocation: computerised random number generation	
Data	Members and fellows of the Royal College of Obstetricians and Gynaecologists in the UK	
Comparisons	1. Handwritten signature in the cover letter 2. Scanned and printed signature in the cover letter	
Outcomes	Response period not specified	
Topic	Health: surgical techniques used in caesarean section operation in the UK	
Mode of Administration	Postal	
Notes	—	
***Risk of bias***		
**Item**	**Authors' judgement**	**Support for judgement**
Allocation concealment?	Unclear	B ‐ unclear

**Table:** McKillip 1984

***Study characteristics***		
Methods	Random allocation: method not specified	
Data	Underclass men from a large rural Midwestern university (US)	
Comparisons	1. Utility cover letter appeal 2. Value expression appeal 3. Knowledge appeal	
Outcomes	Response period not specified	
Topic	Health: evaluation activities for an alcohol education project	
Mode of Administration	Postal	
Notes	—	
***Risk of bias***		
**Item**	**Authors' judgement**	**Support for judgement**
Allocation concealment?	Unclear	B ‐ unclear

**Table:** McLaren 2000a

***Study characteristics***		
Methods	Random allocation: method not specified	
Data	700 Victorian general practitioners selected from a database held by Australasian Medical Publishing Company	
Comparisons	1. Telephone pre‐notification 2. Postcard pre‐notification	
Outcomes	Response within 8 weeks	
Topic	Health: management of early pregnancy, bleeding and miscarriage, referral, diagnostic methods	
Mode of Administration	Postal	
Notes	Equal male and females	
***Risk of bias***		
**Item**	**Authors' judgement**	**Support for judgement**
Allocation concealment?	Unclear	B ‐ unclear

**Table:** McLaren 2000b

***Study characteristics***		
Methods	Random allocation: method not specified	
Data	700 Victorian general practitioners selected from a database held by Australasian Medical Publishing Company	
Comparisons	1. Promise of entry into a prize draw for a holiday on response 2. No incentive	
Outcomes	Response within 8 weeks	
Topic	Health: management of early pregnancy, bleeding and miscarriage, referral, diagnostic methods	
Mode of Administration	Postal	
Notes	Equal male and females	
***Risk of bias***		
**Item**	**Authors' judgement**	**Support for judgement**
Allocation concealment?	Unclear	B ‐ unclear

**Table:** McLean 2014

***Study characteristics***		
Methods	Random allocation: random number generator in Microsoft Excel	
Data	3010 adults from the state of Victoria, Australia, randomly selected from 11 metropolitan and 6 regional electorates of the Australian Electoral Roll	
Comparisons	Pre‐notification postcard vs. no pre‐notification postcard Envelope teaser vs. no envelope teaser	
Outcomes	First response and final response after one month (1 reminder)	
Topic	Health (bulimia nervosa)	
Mode of Administration	Postal	
Notes	260 items. Questionnaires were sent with a personalised letter of invitation that fully explained the procedures of the study. Participants were offered a $10 shopping voucher, contingent on questionnaire return. Reminder questionnaires were mailed to non‐responders 1 month after the original questionnaire had been posted. Pre‐notification postcards were mailed 1 week before the initial mailing of the questionnaire. The envelope teaser was placed on the original and reminder envelopes. The 'teaser' sticker contained the text "You can share valuable information to help people with eating disorders".	
***Risk of bias***		
**Item**	**Authors' judgement**	**Support for judgement**
Sequence generation	Yes	Random allocation: random number generator in Microsoft Excel
Allocation concealment?	Yes	Random allocation: random number generator in Microsoft Excel
Blinding of participants and personnel	Unclear	Blinding of personnel not described. Participants were not blinded to intervention.
Blinding of outcome assessment	Yes	No outcomes were assessed (counts of responses only).
Incomplete outcome data	Yes	Exclusions were reported.
Selective reporting	Yes	First response and final response after one month (1 reminder) reported in full
Other sources of bias	No	No grounds for concern about other sources of bias from reading the study report

**Table:** Meadows 2000

***Study characteristics***		
Methods	Random allocation: method not specified	
Data	600 diabetes patients aged 18 years or over selected from the patient register of a hospital outpatient diabetes centre in North England	
Comparisons	1. High‐frequency response alternatives; horizontal orientation of response options 2. Medium‐frequency response alternatives; horizontal orientation of response options 3. High‐frequency response alternatives; vertical orientation of response options	
Outcomes	Response period not specified	
Topic	Health: diabetes health profile	
Mode of Administration	Postal	
Notes	The high‐frequency response alternatives are: most days, once a week, once a month, less often, never The medium‐frequency response alternatives are: once a week or more often, once a month, about every few months, less often, never Mean age: 52.2 years	
***Risk of bias***		
**Item**	**Authors' judgement**	**Support for judgement**
Allocation concealment?	Unclear	B ‐ unclear

**Table:** Meuleman 2017

***Study characteristics***		
Methods	Random allocation: method unspecified	
Data	Ghanian university students	
Comparisons	Unconditional non‐monetary incentives (EUR 1.20, 2.40 or 4.80 telephone credits) vs. conditional non‐monetary	
Outcomes	Students who started the survey and students who completed the survey	
Topic	Non‐health (the extent to which Ghana‚ National Service Scheme programme contributes to improving intergroup relations and fostering stronger national identities amongst its participants).	
Mode of Administration	Electronic (web survey)	
Notes	Questionnaire not described 5570 students who filled out the background questionnaire were invited by email and text message to participate in an online survey. For students receiving an unconditional incentive, the mobile phone top‐up‚ together with a text message indicating that the top‐up was meant as a token of gratitude for their participation in the survey‚ was sent to the respondents' phones just before they received the survey invitation by email. Respondents on a conditional incentive condition received a text message saying that they would receive a top‐up upon completing the survey. Numbers of students who started the survey and the numbers of students who completed the survey by incentive condition provided by the first author. The Ghanaian social context offered an interesting opportunity: quasi‐cash incentives as mobile phone credit, so‐called top‐up. In Ghana, top‐ups are easily transmittable and used almost universally as a mode of economic exchange. Top‐ups, thus, are a functional equivalent of cash in Ghana.	
***Risk of bias***		
**Item**	**Authors' judgement**	**Support for judgement**
Sequence generation	Unclear	Random allocation: method unspecified
Allocation concealment?	Unclear	Random allocation: method unspecified
Blinding of participants and personnel	Unclear	Blinding of personnel not specified. Participants were not blinded to intervention.
Blinding of outcome assessment	Yes	No outcome assessment used (counts of responses only)
Incomplete outcome data	Yes	Two hundred and fifty five students did not provide a valid email address, and 1130 invitation emails bounced, leaving us with a sample of 4440 students.
Selective reporting	Yes	Numbers of students who started the survey and the numbers of students who completed the survey by incentive condition provided by the author
Other sources of bias	Yes	No grounds for concern about other sources of bias from reading the study report

**Table:** Millar 2011a

***Study characteristics***		
Methods	Random allocation: method unspecified	
Data	Highly Internet‐literate population with full Internet access. 2800 undergraduate students at the main campus of Washington State University	
Comparisons	Choice (mail or Web) vs. mail only vs. Web only	
Outcomes	Final response after one reminder	
Topic	Non‐Health (students' opinions about a variety of issues related to their educational experiences at university)	
Mode of Administration	Electronic/postal	
Notes	Questionnaire contained 36 questions All groups were contacted initially via postal letters and given a $2 bill as an incentive. The choice group students received a paper questionnaire (with an SAE) as well as the Website and individualised access codes for responding online. The mail group students were given only the paper questionnaire with an SAE. The Web only groups were given only the website and individualised access codes, and no paper questionnaire.	
***Risk of bias***		
**Item**	**Authors' judgement**	**Support for judgement**
Sequence generation	Unclear	Random allocation: method unspecified
Allocation concealment?	Unclear	Random allocation: method unspecified
Blinding of participants and personnel	Unclear	Blinding of personnel not described. Participants were not blinded to intervention.
Blinding of outcome assessment	Yes	No outcome assessment used (counts of responses only)
Incomplete outcome data	Yes	Exclusions reported
Selective reporting	Yes	Outcomes reported in full
Other sources of bias	Yes	No grounds for concern about other sources of bias from reading the study report

**Table:** Millar 2011b

***Study characteristics***		
Methods	Random allocation: method unspecified	
Data	Highly Internet‐literate population with full Internet access. A random sample of 4300 students of Washington State University	
Comparisons	Unconditional monetary incentive ($2) vs. no incentive	
Outcomes	Final response after one reminder	
Topic	Non‐health (how students have been affected by the recent economic downturn and the university's resulting budget cuts)	
Mode of Administration	Electronic/postal	
Notes	Questionnaire contained 33 questions. All groups were contacted initially via postal letters and given a $2 bill as an incentive. The choice group students received a paper questionnaire (with an SAE) as well as the Website and individualised access codes for responding online. The mail group students were given only the paper questionnaire with an SAE. The Web only groups were given only the website and individualised access codes, and no paper questionnaire.	
***Risk of bias***		
**Item**	**Authors' judgement**	**Support for judgement**
Sequence generation	Unclear	Random allocation: method unspecified
Allocation concealment?	Unclear	Random allocation: method unspecified
Blinding of participants and personnel	Unclear	Blinding of personnel not described. Participants were not blinded to intervention.
Blinding of outcome assessment	Yes	No outcome assessment used (counts of responses only)
Incomplete outcome data	Yes	Exclusions reported
Selective reporting	Yes	Outcomes reported in full
Other sources of bias	Yes	No grounds for concern about other sources of bias from reading the study report

**Table:** Millar 2019

***Study characteristics***		
Methods	Random allocation: method unspecified	
Data	Adults with colorectal, breast, prostate, ovarian, and multiple myeloma cancers, reported in the Utah Cancer Registry, US	
Comparisons	Postal vs. Web Brochure describing the cancer registry vs. no brochure	
Outcomes	First and final (after 3 reminders) responses	
Topic	Health (current health, cancer recurrence, and willingness to participate in various kinds of cancer research)	
Mode of Administration	Postal vs. Web	
Notes	35 items; formatted a paper questionnaire to visually resemble the web‐based instrument. On the paper questionnaire, each item was enclosed in a box to resemble the page‐by‐page display of the web instrument. The same imagery was used on the paper questionnaire cover and the welcome screen of the web instrument. For both survey modes, the initial mode of contact was postal mail, as email addresses are not routinely obtained in cancer registry reports. Potential participants received up to four mailings (pre‐notification letter with or without brochure, invitation packet with either questionnaire and stamped return envelope or web survey instructions, thank‐you/reminder letter, and a replacement packet). All mailings utilised official University of Utah letterhead and envelopes, as well as postage stamps for outgoing and return‐envelope postage. For the paper survey arm, each of these contacts requested response by paper questionnaire, and the web response option was not offered. For the web arm, all of these contacts only mentioned response via the web‐based questionnaire, and unlike the standard web‐push approach, a paper response option was never offered. We formatted a paper questionnaire to visually resemble the web‐based instrument as much as possible to reduce mode effects.	
***Risk of bias***		
**Item**	**Authors' judgement**	**Support for judgement**
Sequence generation	Unclear	Random allocation: method unspecified
Allocation concealment?	Unclear	Random allocation: method unspecified
Blinding of participants and personnel	Unclear	Blinding of personnel not described. Participants were not blinded to intervention.
Blinding of outcome assessment	Yes	No outcome assessment used (counts of responses only)
Incomplete outcome data	Yes	Exclusions reported
Selective reporting	Yes	Outcomes reported in full
Other sources of bias	Yes	No grounds for concern about other sources of bias from reading the study report

**Table:** Miller 1994

***Study characteristics***		
Methods	Random allocation: method not specified	
Data	Full‐time professors at doctorate granting or comprehensive universities	
Comparisons	1. Incentive (decaffeinated coffee bag) 2. No incentive 3. Cover letter appeal: 'Your input into this matter is very important in determining what faculty consider scholarship to be'. 4. Cover letter appeal: 'It is important to ascertain what faculty consider scholarship to be, in order to develop models of scholarship and further knowledge'. Follow‐up at 3 and 6 weeks after initial mailing	
Outcomes	Response period not specified	
Topic	Non‐health: faculty attitudes about the personal importance of scholarly activities, institutional importance of scholarly activities, attitudes about faculty workload	
Mode of Administration	Postal	
Notes	—	
***Risk of bias***		
**Item**	**Authors' judgement**	**Support for judgement**
Allocation concealment?	Unclear	B ‐ unclear

**Table:** Mills 2019

***Study characteristics***		
Methods	Random allocation: method unspecified	
Data	Owners of agricultural or residential property, Michigan, US	
Comparisons	Single cash note ($2) vs. multiple notes (2 x $1)	
Outcomes	Response after 2 weeks (one reminder)	
Topic	Non‐health (effects of wind energy on rural landowners)	
Mode of Administration	Postal	
Notes	Questionnaire not described. Personalised pre‐notification letter on colour letterhead in a stamped (rather than metered mail) envelope. One week later, all were mailed a questionnaire and a personalised survey invitation letter with $2 prepaid incentive (affixed with handwritten thank‐you sticker) along with an SAE. A week later, all were sent a postcard reminder.	
***Risk of bias***		
**Item**	**Authors' judgement**	**Support for judgement**
Sequence generation	Unclear	Random allocation: method unspecified
Allocation concealment?	Unclear	Random allocation: method unspecified
Blinding of participants and personnel	Unclear	Blinding of personnel not described. Participants were not blinded to intervention.
Blinding of outcome assessment	Yes	No outcomes were assessed (counts of responses only).
Incomplete outcome data	No	Exclusions were not reported.
Selective reporting	Yes	Response after 2 weeks (one reminder) reported in full
Other sources of bias	Yes	No grounds for concern about other sources of bias from reading the study report

**Table:** Mitchell 2011

***Study characteristics***		
Methods	Quasi random allocation: alternation	
Data	Women aged 70‐85 years recruited from 100 GP practices into the SCOOP (SCreening Of Older women for fracture Prevention) trial	
Comparisons	Brown envelope vs. white envelope	
Outcomes	Response after 2 reminders	
Topic	Health (self‐reported fractures over last 12 months)	
Mode of Administration	Postal	
Notes	Contained three health screening questions; York‐modified SF‐12, EQ‐5D and the State‐Trait Anxiety Inventory. The invitation was either in a brown or white envelope and contained a matching pre‐paid reply envelope. A study questionnaire was sent in brown or white envelopes 1 week after consenting to participate. A reminder was sent after 14 days. Telephone reminder 30 days after first mailing	
***Risk of bias***		
**Item**	**Authors' judgement**	**Support for judgement**
Sequence generation	No	Random allocation: alternately allocated by alphabetical surname. This systematic method of allocation is as effective at producing equivalent groups as true randomisation if there is no relationship between sequence and prognostic variables and if the person sending out the envelopes is not aware of the prognostic characteristics of the participants. In this instance, both of these criteria were fulfilled.
Allocation concealment?	No	Quasi‐random allocation: alternation
Blinding of participants and personnel	Unclear	Blinding of personnel not described. Participants were not blinded to intervention.
Blinding of outcome assessment	Yes	No outcomes were assessed (counts of responses only).
Incomplete outcome data	No	Exclusions not reported
Selective reporting	Yes	Response after 2 reminders reported in full
Other sources of bias	Yes	No grounds for concern about other sources of bias from reading the study report

**Table:** Mitchell 2012

***Study characteristics***		
Methods	Random allocation: computer‐generated	
Data	Participants of the Medical Research Council's SCOOP trial of screening older women (aged 70‐84) for fracture risk from Norwich and York centres (n = 2562), UK	
Comparisons	Pre‐notification newsletter vs. none	
Outcomes	Response after one reminder.	
Topic	Health (fracture incidence, quality of life and resource use)	
Mode of Administration	Postal	
Notes	EQ‐5D (2 pages), SF‐12 (3 pages), and State‐Trait Anxiety Index (2 pages). The newsletter took the form of an A5 single sheet, which was folded into a booklet. The newsletter gave the participants an update on the trials' progress, and reminded them about the importance of returning their questionnaires. On the back of the newsletter, there was a brief description, and photograph, of the local study team, with a reminder of contact details for any queries. The newsletter was sent out to the intervention participants approximately 6 weeks before they were due to receive their 24‐month questionnaire.	
***Risk of bias***		
**Item**	**Authors' judgement**	**Support for judgement**
Sequence generation	Yes	Random allocation: computer‐generated
Allocation concealment?	Yes	Random allocation: computer‐generated
Blinding of participants and personnel	Unclear	Blinding of personnel not described. Participants were not blinded to intervention.
Blinding of outcome assessment	Yes	No outcomes were assessed (counts of responses only).
Incomplete outcome data	Yes	Exclusions reported. A number of individuals were excluded from the analysis: died before or within 1 month of mailing (n = 16, intervention; n = 9, control n = 7); participant followed up by telephone only (n = 1, control participant); or lost to follow‐up (n = 1, intervention participant)..
Selective reporting	Yes	Response after one reminder reported in full
Other sources of bias	Yes	No grounds for concern about other sources of bias from reading the study report

**Table:** Mitchell 2021a

***Study characteristics***		
Methods	Random allocation: method unspecified	
Data	Participants in the Knee Replacement Bandaging Study (KReBS) RCT, UK	
Comparisons	Personalised text message reminder vs. non‐personalised text message reminder	
Outcomes	Final response	
Topic	Health (patient‐reported outcomes in total knee replacement patients)	
Mode of Administration	Postal	
Notes	Questionnaire not described. Package not described.	
***Risk of bias***		
**Item**	**Authors' judgement**	**Support for judgement**
Sequence generation	Unclear	Random allocation: method unspecified
Allocation concealment?	Unclear	Random allocation: method unspecified
Blinding of participants and personnel	No	Neither personnel nor participants were blinded to intervention.
Blinding of outcome assessment	Yes	No outcomes were assessed (counts of responses only).
Incomplete outcome data	Yes	Five participants died or withdrew following randomisation.
Selective reporting	Yes	Completion and return rate outcomes reported in full
Other sources of bias	Yes	No grounds for concern about other sources of bias from reading the study report

**Table:** Mitchell 2021b

***Study characteristics***		
Methods	Random allocation: computer‐generated	
Data	Follow‐up of participants in a clinical trial of compression bandaging following knee arthroplasty	
Comparisons	Pen (branded with the York Trials Unit and University of York logos) vs. no pen	
Outcomes	First response	
Topic	Health (outcomes following knee arthroplasty)	
Mode of Administration	Postal	
Notes	Questionnaire not described. Questionnaire package not described. The incentive pen was branded with the York Trials Unit and University of York logos.	
***Risk of bias***		
**Item**	**Authors' judgement**	**Support for judgement**
Sequence generation	Yes	Random allocation: computer‐generated
Allocation concealment?	Yes	Random allocation: computer‐generated
Blinding of participants and personnel	No	Neither participants nor personnel were blinded.
Blinding of outcome assessment	Yes	No outcomes were assessed (counts of responses only).
Incomplete outcome data	Yes	Exclusions were reported.
Selective reporting	Yes	First response reported in full
Other sources of bias	Yes	No grounds for concern about other sources of bias from reading the study report

**Table:** Mizes 1984

***Study characteristics***		
Methods	Random allocation: method not specified	
Data	Physicians specialising in allergy randomly selected from all physicians listed under allergy or allergy/immunology in the telephone directory yellow pages of major metropolitan areas across the US	
Comparisons	1. No incentive; answer postcard 2. $1 cheque; answer postcard 3. $5 cheque; answer postcard 4. $1 cheque; answer cheque 5. $5 cheque; answer cheque	
Outcomes	Response period not specified	
Topic	Health: incidence, treatment, and the success of treatment of rhinitis medicamentosa	
Mode of Administration	Postal	
Notes	Method of allocation confirmed through contact with author	
***Risk of bias***		
**Item**	**Authors' judgement**	**Support for judgement**
Allocation concealment?	Unclear	B ‐ unclear

**Table:** Mockovak 2012

***Study characteristics***		
Methods	Random allocation: method unspecified	
Data	Random sample of 1000 purchasers of the print copy of the Bureau of Labor Statistics Occupational Outlook Handbook, selected from a list provided by the Government Printing Office, US	
Comparisons	Cover letter redesigned using the principles of Information Mapping (bold headings as questions, e.g., 'What is the purpose of this survey?' and 'How long will this take?') vs. conventional cover letter	
Outcomes	Response rate after one mailing	
Topic	Non‐health (opinion on discontinuation of publication of the print version of the Bureau of Labor Statistics‚ Occupational Outlook Handbook)	
Mode of Administration	Postal/electronic	
Notes	6 items. Respondents were contacted via mail and given the option of either mailing back a simple, 6‐item paper questionnaire or completing a Web version of the form. There was no pre‐survey notification, one mailed contact attempt, and no follow‐up attempts. Also, no incentives were used. Respondents were asked to complete and return the survey within 30 days. Respondents accessed the Web survey by entering a simple URL in their Web browser. The Web survey did not require use of either a username or password. The content of the one‐page cover letter in each group was almost identical. The primary differences were the word labels, which were bolded, and the general formatting used by the Information Mapping version. No colour was used on either version. The primary appeal for co‐operation was an egoistic one, since discontinuation of the printed copy could potentially lead to negative impacts on the users of the print version of the Handbook.	
***Risk of bias***		
**Item**	**Authors' judgement**	**Support for judgement**
Sequence generation	Unclear	Random allocation: method unspecified
Allocation concealment?	Unclear	Random allocation: method unspecified
Blinding of participants and personnel	Unclear	Blinding of personnel not described. Participants were not blinded to intervention.
Blinding of outcome assessment	Yes	No outcome assessment used (counts of responses only)
Incomplete outcome data	Yes	Exclusions reported
Selective reporting	Yes	Outcomes reported in full
Other sources of bias	Yes	No grounds for concern about other sources of bias from reading the study report

**Table:** Mond 2004

***Study characteristics***		
Methods	Random allocation: using random number generation in SPSS	
Data	Australian Capital Territory residents	
Comparisons	1. Short questionnaire (8 pages long) 2. Long questionnaire (14 pages long)	
Outcomes	Response after 2 reminders (8 weeks)	
Topic	Health (psychological distress, disability, and quality of life)	
Mode of Administration	Postal	
Notes	8 pages vs. 14 pages. A first reminder letter was sent to non‐respondents 8 weeks after initial mailout. Replacement questionnaires were posted to individuals who received the initial questionnaire by hand delivery. Amongst non‐respondents who received the initial questionnaire by post, half received a replacement questionnaire by post and the remaining half received a second copy of the questionnaire by hand delivery. Remaining non‐respondents received a second reminder letter (and replacement questionnaire) by post after a further 8 weeks.	
***Risk of bias***		
**Item**	**Authors' judgement**	**Support for judgement**
Sequence generation	Yes	Random allocation: computer‐generated
Allocation concealment?	Yes	Random allocation: computer‐generated
Blinding of participants and personnel	Unclear	Blinding of personnel not described. Participants were not blinded to intervention.
Blinding of outcome assessment	Yes	No outcomes were assessed (counts of responses only).
Incomplete outcome data	Yes	Exclusions were reported.
Selective reporting	Yes	Response after 2 reminders (8 weeks) reported in full
Other sources of bias	Yes	No grounds for concern about other sources of bias from reading the study report

**Table:** Morgan 2017

***Study characteristics***		
Methods	Random allocation: computer‐generated	
Data	Participants in the host RCT of Cool Little Kids Online were Australian parents of children aged 3 to 6 years who were assessed as high on temperamental inhibition.	
Comparisons	Conditional non‐monetary incentive (lottery with 1 in 20 chance of winning $50) vs. no incentive	
Outcomes	Response after 4 reminders	
Topic	Health (Alabama Parenting Questionnaire)	
Mode of Administration	Electronic	
Notes	Alabama Parenting Questionnaire (42 questions). The average duration to complete the questionnaire was 40 min. Participants were sent an email invitation with a link to complete the final study assessment 24 weeks after they had enrolled in the parent study. Non‐responders were sent an email reminder after 1 week, and a second reminder after a further week had passed. Those who had still not responded were telephoned 1 week later as a friendly reminder and to check if they had received the emails (a voicemail was left if the participant could not be reached). A final telephone contact was made after a further week or two had elapsed (by SMS if the participant had been reached by phone previously).	
***Risk of bias***		
**Item**	**Authors' judgement**	**Support for judgement**
Sequence generation	Yes	Random allocation: computer‐generated
Allocation concealment?	Yes	Random allocation: computer‐generated
Blinding of participants and personnel	Unclear	Blinding of personnel not described. Participants were not blinded to intervention.
Blinding of outcome assessment	Yes	No outcomes were assessed (counts of responses only).
Incomplete outcome data	Yes	Exclusions were reported.
Selective reporting	Yes	Response after 4 reminders reported in full
Other sources of bias	Yes	No grounds for concern about other sources of bias from reading the study report

**Table:** Morris 2013

***Study characteristics***		
Methods	Random allocation: computer‐generated	
Data	Women participating in a survey on experiences of fertility problems	
Comparisons	Electronic (web) vs. postal vs. telephone vs. choice	
Outcomes	Response after 3 reminders	
Topic	Health (fertility problems)	
Mode of Administration	Mixed, plus choice of telephone	
Notes	44 questions, more than 16 pages. Package not described	
***Risk of bias***		
**Item**	**Authors' judgement**	**Support for judgement**
Sequence generation	Yes	Random allocation: computer‐generated
Allocation concealment?	Yes	Random allocation: computer‐generated
Blinding of participants and personnel	Yes	Allocation to intervention was conducted by an external mailout company.
Blinding of outcome assessment	Yes	No outcomes were assessed (counts of responses only).
Incomplete outcome data	Yes	Exclusions were reported.
Selective reporting	Yes	Response after 3 reminders reported in full
Other sources of bias	Yes	No grounds for concern about other sources of bias from reading the study report

**Table:** Morrison 2003

***Study characteristics***		
Methods	Random allocation: using random number generation in SPSS	
Data	Local residents	
Comparisons	1. Study feedback information booklet 2. No information booklet	
Outcomes	Response period not specified	
Topic	Health: sociodemographics, travel behaviour, risk perception, attitudes to the local area and health	
Mode of Administration	Postal	
Notes	—	
***Risk of bias***		
**Item**	**Authors' judgement**	**Support for judgement**
Allocation concealment?	Unclear	B ‐ unclear

**Table:** Mortagy 1985

***Study characteristics***		
Methods	Random allocation: method not specified	
Data	Persons listed in electoral registers (Southampton and New Forest, UK)	
Comparisons	1. Offered lottery ticket 2. Not offered lottery ticket Reminder sent to non‐respondents after 4 weeks	
Outcomes		
Topic	Health: respiratory symptoms such as breathlessness, wheezing, cough, phlegm, hyper‐irritability of the bronchi; family illness; smoking habits; drug treatment	
Mode of Administration	Postal	
Notes	—	
***Risk of bias***		
**Item**	**Authors' judgement**	**Support for judgement**
Allocation concealment?	Unclear	B ‐ unclear

**Table:** Moses 2004

***Study characteristics***		
Methods	Random allocation: computerised random number generation	
Data	Consultants identified from the Royal College of Obstetricians and Gynaecologists (RCOG) database	
Comparisons	1. Prize draw incentive to win a personal digital assistant 2. No incentive	
Outcomes	Response period not specified	
Topic	Health: current practice for the laparoscopic diagnosis and treatment of women with pelvic pain due to endometriosis	
Mode of Administration	Postal	
Notes	Single page; 10 questions. One reminder was sent to non‐responders after 3 months.	
***Risk of bias***		
**Item**	**Authors' judgement**	**Support for judgement**
Sequence generation	Yes	Random allocation: computerised random number generation
Allocation concealment?	Yes	Random allocation: computerised random number generation
Blinding of participants and personnel	Unclear	Blinding of personnel not specified. Participants were not blinded to intervention.
Blinding of outcome assessment	Yes	No outcome assessment used (counts of responses only)
Incomplete outcome data	No	Exclusions not reported
Selective reporting	Yes	Outcomes reported
Other sources of bias	Yes	No grounds for concern about other sources of bias from reading the study report

**Table:** Moss 1991

***Study characteristics***		
Methods	Random allocation: method not specified	
Data	Members of the National Council for Educational Measurement	
Comparisons	1. Typed salutation; metered return envelope 2. Typed salutation; non‐metered return envelope 3. Handwritten salutation; metered return envelope 4. Handwritten salutation; non‐metered return envelope	
Outcomes	Response period not specified	
Topic	Non‐health: respondents belief about the frequency and credibility of criticisms of standardised test	
Mode of Administration	Postal	
Notes	—	
***Risk of bias***		
**Item**	**Authors' judgement**	**Support for judgement**
Allocation concealment?	Unclear	B ‐ unclear

**Table:** Mullen 1987

***Study characteristics***		
Methods	Random allocation: method not specified	
Data	Non‐federal office‐based members of the American Academy	
Comparisons	1. Blue and white sticker incentive only 2. Withdrawal provision only 3. Incentive and withdrawal provision 4. No treatment Non‐respondents followed up 3 times	
Outcomes	—	
Topic	Health: counselling adult patients about smoking, weight, exercise, and stress, interest in continuing education	
Mode of Administration	Postal	
Notes	—	
***Risk of bias***		
**Item**	**Authors' judgement**	**Support for judgement**
Allocation concealment?	Unclear	B ‐ unclear

**Table:** Mullner 1982

***Study characteristics***		
Methods	Random allocation: method not specified	
Data	Community hospitals registered with the AHA (US)	
Comparisons	1. Questionnaire in booklet form (QBF); most salient and relevant question first (SRF); cover letter personal in tone (CLP); results promised (RP) 2. QBF; SRF; CLP; no RP 3. QBF; SRF; cover letter impersonal in tone (CLI); RP 4. QBF; SRF; CLI; no RP 5. QBF; salient and relevant questions last (SRL); CLP; RP 6. QBF; SRL; CLP; no RP 7. QBF; SRL; CLI; RP 8. QBF; SRL; CLI; no RP 9. Questionnaire in 2‐sided form style (QF); SRF; CLP; RP 10. QF; SRF; CLP; no RP 11. QF; SRF; CLI; RP 12. QF; SRF; CLI; no RP 13. QF; SRL; CLP; RP 14. QF; SRL; CLP; no RP 15. QF; SRL; CLI; RP 16. QF; SRL; CLI; no RP	
Outcomes	Response period not specified	
Topic	Health: corporate planning, risk management programmes, expenditures for hospital supplies, admitting privileges of physicians, programmes of special services for the elderly	
Mode of Administration	Postal	
Notes	—	
***Risk of bias***		
**Item**	**Authors' judgement**	**Support for judgement**
Allocation concealment?	Unclear	B ‐ unclear

**Table:** Munoz 2017

***Study characteristics***		
Methods	Random allocation: method unspecified	
Data	A sample of 95 participants (63 English‐speakers and 32 Spanish‐speakers) at high‐risk for depression recruited online to participate in a Healthy Mood study, US	
Comparisons	(140) Telephone and email reminder vs. email only reminder	
Outcomes	First response (at 1 month)	
Topic	Health (depression)	
Mode of Administration	Online	
Notes	All participants received monetary incentives in the form of online gift certificates to a popular online megastore. Participants received US $10 for completing each of the three follow‐up assessments (1, 3, and 6 months after consenting), and a US $20 bonus if all three follow‐ups were completed. Thus, a participant could earn up to US $50. At each follow‐up point, all participants received up to three emails in one week, inviting them to complete follow‐up by following an embedded link. Response at one month (time point one) included in review Participants were randomised to two conditions: (1) Call: Those randomised to the Call condition were phoned (up to 10 attempts were made to reach them) and asked to complete the surveys by phone if they did not fill out the survey online; (2) No Call. Those randomised to the No Call condition received no phone calls.	
***Risk of bias***		
**Item**	**Authors' judgement**	**Support for judgement**
Sequence generation	Unclear	Random allocation: method unspecified
Allocation concealment?	Unclear	Random allocation: method unspecified
Blinding of participants and personnel	Yes	All participants were informed, via the consent form, that they may receive phone calls if they did not complete follow‐up surveys online; thus the participants were blind to condition. Blinding of personnel was not reported.
Blinding of outcome assessment	Yes	No outcomes were assessed (counts of responses only).
Incomplete outcome data	No	Exclusions were not reported.
Selective reporting	Yes	Outcomes reported in full
Other sources of bias	Yes	No grounds for concern about other sources of bias from reading the study report

**Table:** Murawski 1996

***Study characteristics***		
Methods	Random allocation: method not specified	
Data	Veterans aged 60‐65	
Comparisons	1. Duke questionnaire 2. SF‐36 questionnaire 3. SIP questionnaire Follow‐up sent 1 and 4 weeks	
Outcomes	—	
Topic	Health: health‐related quality of life (HRQoL)	
Mode of Administration	Postal	
Notes	Age: 60‐65 years	
***Risk of bias***		
**Item**	**Authors' judgement**	**Support for judgement**
Allocation concealment?	Unclear	B ‐ unclear

**Table:** Murdoch 2014

***Study characteristics***		
Methods	Random allocation: method unspecified	
Data	324 male Gulf War I era veterans who had applied for disability benefits, US	
Comparisons	Identifying feature on return vs. none Higher monetary incentive ($20) vs. lower monetary incentive ($10)	
Outcomes	Response after 6 weeks (3 reminders)	
Topic	Health (combat, unwanted sexual attention, and other lifetime and military experiences)	
Mode of Administration	Postal	
Notes	25‐page questionnaire. For all groups, the initial mailing included a cover letter (printed on Minneapolis VA Medical Center letter‐head and listed the study's funding agency), the cash incentive, and questionnaire. At two‐week intervals, non‐respondents were mailed postcard reminder, second mailing of the survey, and final mailing of the survey via overnight mail (Federal Express).	
***Risk of bias***		
**Item**	**Authors' judgement**	**Support for judgement**
Sequence generation	Unclear	Random allocation: method unspecified
Allocation concealment?	Unclear	Random allocation: method unspecified
Blinding of participants and personnel	No	Neither participants nor personnel were blind to intervention.
Blinding of outcome assessment	Yes	No outcomes were assessed (counts of responses only).
Incomplete outcome data	No	Exclusions were not reported.
Selective reporting	Yes	Response after 6 weeks (3 reminders) reported in full
Other sources of bias	Yes	No grounds for concern about other sources of bias from reading the study report

**Table:** Murphy 1991

***Study characteristics***		
Methods	Random allocation: alternation	
Data	International freight forwarders (US)	
Comparisons	1. Pre‐notification postcard 2. No pre‐notification postcard Follow‐up sent after 3 weeks	
Outcomes	Response within 62 days	
Topic	Non‐health: US industrial firm check	
Mode of Administration	Postal	
Notes	Age: 45‐48 years	
***Risk of bias***		
**Item**	**Authors' judgement**	**Support for judgement**
Allocation concealment?	No	C ‐ inadequate

**Table:** Murphy 2020

***Study characteristics***		
Methods	Random allocation: randomisation log generated by a biostatistician	
Data	Physicians from the American Medical Association MasterFile from haematology or oncology specialties	
Comparisons	Mixed vs. electronic	
Outcomes	Response after 6 months (2 reminders)	
Topic	Health (characteristics of physicians' practices; referral and recruitment of patients to clinical trials; and barriers to trial accrual).	
Mode of Administration	Postal/electronic	
Notes	5‐min survey. Mail‐based recruitment included a survey packet with: (1) cover letter describing the survey and inviting participation; (2) paper copy of the survey and postage‐paid return envelope; and (3) a reminder postcard with web link after 2 wks; (4) final reminder paper survey after 2 wks. Email‐based recruitment included an email describing the survey and inviting participation, along with the web link. Reminder email after 1 wk; reminder postcard 2 weeks later Most physicians assigned to mail‐based recruitment actually completed the survey online via the link provided in the cover letter, and those in email‐based recruitment did not respond until they received a reminder postcard by mail.	
***Risk of bias***		
**Item**	**Authors' judgement**	**Support for judgement**
Sequence generation	Yes	Random allocation: randomisation log generated by a biostatistician
Allocation concealment?	Yes	Random allocation: randomisation log generated by a biostatistician
Blinding of participants and personnel	Yes	Study investigators were blind to randomised assignment.
Blinding of outcome assessment	Yes	No outcomes were assessed (counts of responses only).
Incomplete outcome data	Yes	Exclusions reported
Selective reporting	Yes	Response after each reminder, and after 6 months (2 reminders) reported in full
Other sources of bias	Yes	No grounds for concern about other sources of bias from reading the study report

**Table:** Myers 1969

***Study characteristics***		
Methods	Random allocation: method not specified	
Data	Households listed in a street order telephone directory (Los Angeles, US)	
Comparisons	1. Pre‐contact letter 2. Questionnaire only 3. Questionnaire, then follow‐up letter	
Outcomes	Response period not specified	
Topic	Non‐health: reaction of public to various promotion efforts by the bank to establish the image of the bank	
Mode of Administration	Postal	
Notes	—	
***Risk of bias***		
**Item**	**Authors' judgement**	**Support for judgement**
Allocation concealment?	Unclear	B ‐ unclear

**Table:** Myhre 2019a

***Study characteristics***		
Methods	Quasi randomisation: alternation	
Data	Mothers of 6‐month‐olds; participants in a longitudinal study assessing dietary habits in infants, Norway	
Comparisons	Conditional non‐monetary incentive (50 EUR voucher) vs. conditional lottery (500 EUR and 1000 EUR) Handwritten name and address vs. printed label	
Outcomes	Response after 3 weeks (2 reminders)	
Topic	Health (national dietary survey amongst infants)	
Mode of Administration	Electronic (paper reminder questionnaire)	
Notes	15 pages, containing a total of 28‐127 questions (depended on previous choices when answering the questionnaire), 20 mins completion time For all the invitees, the invitation contained information about the study and a link to a web‐based questionnaire. Telephone reminder to non‐responders after 1 wk and written reminder after 3 weeks, including paper questionnaire	
***Risk of bias***		
**Item**	**Authors' judgement**	**Support for judgement**
Sequence generation	No	Quasi‐randomisation: alternation
Allocation concealment?	No	Quasi‐randomisation: alternation
Blinding of participants and personnel	Unclear	Blinding of personnel not described. Participants were not blinded to intervention.
Blinding of outcome assessment	Yes	No outcomes were assessed (counts of responses only).
Incomplete outcome data	Yes	Exclusions reported
Selective reporting	Yes	Response after 3 weeks (2 reminders) reported in full
Other sources of bias	Yes	No grounds for concern about other sources of bias from reading the study report

**Table:** Myhre 2019b

***Study characteristics***		
Methods	Quasi randomisation: alternation	
Data	Mothers of 12‐month‐olds; participants in a longitudinal study assessing dietary habits in infants, Norway	
Comparisons	Email invitation vs. postal	
Outcomes	Response after 3 weeks (2 reminders)	
Topic	Health (national dietary survey amongst infants)	
Mode of Administration	Electronic (paper reminder questionnaire).	
Notes	12 months 23 pages, 247‐275 questions, 40 mins completion time For all the invitees, the invitation contained information about the study and a link to a web‐based questionnaire. Telephone reminder to non‐responders after 1 wk and written reminder after 3 weeks, including paper questionnaire	
***Risk of bias***		
**Item**	**Authors' judgement**	**Support for judgement**
Sequence generation	No	Quasi‐randomisation: alternation
Allocation concealment?	No	Quasi‐randomisation: alternation
Blinding of participants and personnel	Unclear	Blinding of personnel not described. Participants were not blinded to intervention.
Blinding of outcome assessment	Yes	No outcomes were assessed (counts of responses only).
Incomplete outcome data	Yes	Exclusions reported
Selective reporting	Yes	Response after 3 weeks (2 reminders) reported in full
Other sources of bias	Yes	No grounds for concern about other sources of bias from reading the study report

**Table:** Nagata 1995

***Study characteristics***		
Methods	Random allocation: method not specified	
Data	Male owners of telephones (Gitu City, Japan)	
Comparisons	1. 1‐page; cigarette smoking and drinking 2. 2 pages; cigarette smoking and drinking; medical history 3. 2 pages; cigarette smoking and drinking; family history 4. 2 pages; cigarette smoking and drinking; family history; consanguineous marriage 5. 3 pages; cigarette smoking and drinking; medical history; family history; consanguineous marriage 6. 2 pages; cigarette smoking and drinking; medical history; family history; consanguineous marriage Follow‐up in group 1 only	
Outcomes	Response period not specified	
Topic	Health: medical history, family history, smoking, drinking	
Mode of Administration	Postal	
Notes	Age: mostly 46‐65 years	
***Risk of bias***		
**Item**	**Authors' judgement**	**Support for judgement**
Allocation concealment?	Unclear	B ‐ unclear

**Table:** Nakai 1997

***Study characteristics***		
Methods	Random allocation: alternation	
Data	People aged 40‐64	
Comparisons	1. Short questionnaire (4 pages) 2. Long questionnaire (8 pages)	
Outcomes	—	
Topic	Health: health status, health‐related practice, smoking status	
Mode of Administration	Postal	
Notes	Age: 40‐64 years	
***Risk of bias***		
**Item**	**Authors' judgement**	**Support for judgement**
Allocation concealment?	No	C ‐ inadequate

**Table:** Nakash 2007

***Study characteristics***		
Methods	Random allocation: computer‐generated	
Data	Adults with severe ankle sprain enrolled in the CAST trial of ankle supports, UK	
Comparisons	User‐friendly Trial Calendar of prompts and reminders for trial participants vs. no calendar	
Outcomes	Response after 2 reminders	
Topic	Health (Foot and Ankle Outcome Score (FAOS), SF‐12 and EQ‐5D)	
Mode of Administration	Postal	
Notes	30‐45 minutes. Follow‐up in the CAST trial was conducted at 4 weeks, 12 weeks and 9 months. We extracted response data for the 4‐week outcome.	
***Risk of bias***		
**Item**	**Authors' judgement**	**Support for judgement**
Sequence generation	Yes	Random allocation: computer‐generated
Allocation concealment?	Yes	Random allocation: computer‐generated
Blinding of participants and personnel	No	Neither participants nor personnel were blind to intervention.
Blinding of outcome assessment	Yes	No outcomes were assessed (counts of responses only).
Incomplete outcome data	Yes	Exclusions were reported.
Selective reporting	Yes	Response after 2 reminders reported in full
Other sources of bias	Yes	No grounds for concern about other sources of bias from reading the study report

**Table:** Nakazawa 2020

***Study characteristics***		
Methods	Random allocation: method unspecified	
Data	4812 bereaved family members, Japan	
Comparisons	Pen vs no pen; postal follow‐up including vs. excluding questionnaire	
Outcomes	First response (pen), final response (after reminder questionnaire or letter alone)	
Topic	Health (evaluation of end‐of‐life care)	
Mode of Administration	Postal	
Notes	Three questionnaires: Good Death Inventory (18 domains representing concepts important to a good death in a Japanese patient with cancer), the Care Evaluation Scale (10 domains representing concepts important to EOL care), and the Caregiving Consequences Inventory to evaluate caregiver burden	
***Risk of bias***		
**Item**	**Authors' judgement**	**Support for judgement**
Sequence generation	Unclear	Random allocation: method unspecified
Allocation concealment?	Unclear	Random allocation: method unspecified
Blinding of participants and personnel	Unclear	Blinding of personnel not described. Participants were not blinded to intervention.
Blinding of outcome assessment	Yes	No outcomes were assessed (counts of responses only).
Incomplete outcome data	Yes	Exclusions were reported.
Selective reporting	Yes	First response (pen), final response (after reminder questionnaire or letter alone) reported in full
Other sources of bias	Yes	No grounds for concern about other sources of bias from reading the study report

**Table:** Napoles‐Springer 2004

***Study characteristics***		
Methods	Random allocation: computerised random number generation	
Data	African‐American or white, who were at least 50 years old, and had once visited the primary care practices of an academic health centre during the previous year	
Comparisons	1. Advance notice letter 2. No advance letter	
Outcomes	Response period within 2 weeks	
Topic	Health: patient satisfaction in adult ambulatory care	
Mode of Administration	Postal	
Notes	—	
***Risk of bias***		
**Item**	**Authors' judgement**	**Support for judgement**
Allocation concealment?	No	C ‐ inadequate

**Table:** Nathenson 2019

***Study characteristics***		
Methods	Random allocation: method unspecified	
Data	405 elementary education teachers in a large urban school district in the northeastern United States	
Comparisons	Emphasis on incentive (conditional $25) vs. no emphasis; motivational appeal stresses altruism vs. other appeals (achievement, affiliation, reciprocity, commitment and consistency)	
Outcomes	e‐click and e‐completion	
Topic	Non‐health (research on how students learn mathematics)	
Mode of Administration	Web survey	
Notes	Emails contained a link to the OGAP/NSF assessment as well as a unique survey code to access the assessment. The first email was sent on a Thursday at approximately 6:05 a.m. If a teacher did not complete the survey by the following Monday afternoon, they were sent a second email under the same motivation appeal at approximately 6:05 a.m. that Tuesday. Teachers were randomised to receive one of six motivational appeals and were re‐randomised to receive a different appeal each subsequent week, conditional on not having completed the survey.	
***Risk of bias***		
**Item**	**Authors' judgement**	**Support for judgement**
Sequence generation	Unclear	Random allocation: method unspecified
Allocation concealment?	Unclear	Random allocation: method unspecified
Blinding of participants and personnel	Unclear	Blinding of personnel not described. Participants were not blinded to intervention.
Blinding of outcome assessment	Yes	No outcomes were assessed (counts of responses only).
Incomplete outcome data	Yes	Exclusions were reported.
Selective reporting	Yes	e‐click and e‐Comction reported in full
Other sources of bias	Yes	No grounds for concern about other sources of bias from reading the study report

**Table:** Nederhof 1982

***Study characteristics***		
Methods	Random allocation: method not specified	
Data	All members of the general population of a medium‐sized Dutch town	
Comparisons	1. Telephone pre‐notification 2. Mail pre‐notification	
Outcomes	Response period not specified	
Topic	Not specified	
Mode of Administration	Postal	
Notes	Method of allocation ascertained through contact with author	
***Risk of bias***		
**Item**	**Authors' judgement**	**Support for judgement**
Allocation concealment?	Unclear	B ‐ unclear

**Table:** Nederhof 1983a

***Study characteristics***		
Methods	Random allocation: using alternation	
Data	Group of members of the general public in Leyden, Netherlands	
Comparisons	1. Computer‐printed address label 2. Handwritten address label 3. Ballpoint pen worth $0.35 4. No incentive	
Outcomes	Response period not specified	
Topic	Non‐health: attitudes towards suicide	
Mode of Administration	Postal	
Notes	—	
***Risk of bias***		
**Item**	**Authors' judgement**	**Support for judgement**
Allocation concealment?	Unclear	B ‐ unclear

**Table:** Nederhof 1983b

***Study characteristics***		
Methods	Random allocation: using alternation	
Data	Group of members of the general public in Leyden, Netherlands	
Comparisons	1. Computer‐printed address label 2. Handwritten address label	
Outcomes	Response period not specified	
Topic	Non‐health: attitudes about females social roles and vegetarianism	
Mode of Administration	Postal	
Notes	—	
***Risk of bias***		
**Item**	**Authors' judgement**	**Support for judgement**
Allocation concealment?	Unclear	B ‐ unclear

**Table:** Nederhof 1988

***Study characteristics***		
Methods	Random allocation: method not specified	
Data	Biotechnologists living in the Netherlands	
Comparisons	1. Graphic illustration on cover of questionnaire largely in white 2. Graphic illustration on cover of questionnaire largely in black	
Outcomes	Response period not specified	
Topic	Health: development in biotechnology in the Netherlands	
Mode of Administration	Postal	
Notes	Method of allocation ascertained through contact with author	
***Risk of bias***		
**Item**	**Authors' judgement**	**Support for judgement**
Allocation concealment?	Unclear	B ‐ unclear

**Table:** Neider 1981a

***Study characteristics***		
Methods	Random allocation: method not specified	
Data	A sample of training and development directors who were members of the American Association for Training and Development	
Comparisons	1. Hand‐addressed outgoing envelope 2. Typed outgoing envelope 3. Computer‐generated label on outgoing envelope	
Outcomes	Response period not specified	
Topic	Not specified	
Mode of Administration	Postal	
Notes	Method of allocation ascertained through contact with author	
***Risk of bias***		
**Item**	**Authors' judgement**	**Support for judgement**
Allocation concealment?	Unclear	B ‐ unclear

**Table:** Neider 1981b

***Study characteristics***		
Methods	Random allocation: method not specified	
Data	A sample of Class I and II common carriers	
Comparisons	1. Hand‐addressed outgoing envelope 2. Typed outgoing envelope 3. Computer‐generated label on outgoing envelope	
Outcomes	Response period not specified	
Topic	Not specified	
Mode of Administration	Postal	
Notes	Method of allocation ascertained through contact with author	
***Risk of bias***		
**Item**	**Authors' judgement**	**Support for judgement**
Allocation concealment?	Unclear	B ‐ unclear

**Table:** Nesrallah 2014

***Study characteristics***		
Methods	Random allocation: computer‐generated	
Data	Practising nephrologists, Canada	
Comparisons	Non‐monetary incentive ($40 charitable donation) vs. no incentive	
Outcomes	Response after 4 weeks (2 reminders)	
Topic	Health (nephrologists' attitudes toward home dialysis)	
Mode of Administration	Web survey	
Notes	Questionnaire not described. Pre‐notification, invitation, first reminder, and final reminder letters were sent by email to both groups simultaneously over a 4‐week period. Letters were identical for both groups except for the following phrase: "By completing the survey, and in compensation for your time, a charitable donation of $40 will be made, on your behalf, to the Kidney Foundation of Canada, by our study sponsors."	
***Risk of bias***		
**Item**	**Authors' judgement**	**Support for judgement**
Sequence generation	Yes	Random allocation: computer‐generated
Allocation concealment?	Yes	Random allocation: computer‐generated
Blinding of participants and personnel	Unclear	Blinding of personnel not described. Participants were not blinded to intervention.
Blinding of outcome assessment	Yes	No outcomes were assessed (counts of responses only).
Incomplete outcome data	No	Exclusions not reported
Selective reporting	Yes	Response after 4 weeks (2 reminders) reported in full
Other sources of bias	Yes	No grounds for concern about other sources of bias from reading the study report

**Table:** Nevin 1975a

***Study characteristics***		
Methods	Random allocation: method not specified	
Data	Residents of university halls (US)	
Comparisons	1. Given deadline of 5 days 2. Given deadline of 7 days 3. Given deadline of 9 days 4. No deadline given	
Outcomes	—	
Topic	Non‐health: attitudes about residence halls	
Mode of Administration	Postal	
Notes	—	
***Risk of bias***		
**Item**	**Authors' judgement**	**Support for judgement**
Allocation concealment?	Unclear	B ‐ unclear

**Table:** Nevin 1975b

***Study characteristics***		
Methods	Random allocation: method not specified	
Data	Non‐responders to earlier survey of university hall residents	
Comparisons	1. Follow‐up letter with casual approach 2. Follow‐up letter with veiled threat	
Outcomes	Response within 18 days	
Topic	Non‐health: attitudes about residence halls	
Mode of Administration	Postal	
Notes	—	
***Risk of bias***		
**Item**	**Authors' judgement**	**Support for judgement**
Allocation concealment?	Unclear	B ‐ unclear

**Table:** Newby 2003

***Study characteristics***		
Methods	Random allocation: computerised random number generation	
Data	Businesses listed in Perth, Western Australia	
Comparisons	1. Monetary incentive worth A $20 2. Pre‐notification by telephone 3. Control	
Outcomes	Response period not specified	
Topic	Non‐health: attitudes and expectations of the self‐employed	
Mode of Administration	Postal	
Notes	—	
***Risk of bias***		
**Item**	**Authors' judgement**	**Support for judgement**
Allocation concealment?	Unclear	B ‐ unclear

**Table:** Newland 1977

***Study characteristics***		
Methods	Random allocation: method not specified	
Data	Individuals listed in electoral register (Southampton, UK)	
Comparisons	1. First‐class stamp on outgoing envelope; first‐class stamp on return envelope; white envelopes 2. Second‐class stamp on outgoing envelope; second‐class envelope on return; white envelope 3. Second‐class frank on outgoing envelope; second‐class business‐reply return envelope; white envelopes 4. First‐class stamp on outgoing envelope; first‐class stamp on return envelope; brown envelopes 5. Second‐class stamp on outgoing envelope; second‐class envelope on return; brown envelope 6. Second‐class frank on outgoing envelope; second‐class business‐reply return envelope; brown envelopes Follow up at 2 and 16 weeks, including another copy of the questionnaire	
Outcomes	—	
Topic	Health: details of headache and accompanying symptoms, general health, demographics	
Mode of Administration	Postal	
Notes	—	
***Risk of bias***		
**Item**	**Authors' judgement**	**Support for judgement**
Allocation concealment?	Unclear	B ‐ unclear

**Table:** Nichols 1966

***Study characteristics***		
Methods	Random allocation: method not specified	
Data	College students who had returned a similar questionnaire 1 year previously	
Comparisons	1. Follow‐up postcard after 3 days; further follow‐up mailings 2. Not sent postcard after 3 days; further follow‐up mailings	
Outcomes	Response within 120 days	
Topic	Not specified	
Mode of Administration	Postal	
Notes	—	
***Risk of bias***		
**Item**	**Authors' judgement**	**Support for judgement**
Allocation concealment?	Unclear	B ‐ unclear

**Table:** Nichols 1988

***Study characteristics***		
Methods	Random allocation: alternation	
Data	Individuals listed on the electoral roll (Southampton, UK)	
Comparisons	1. Information booklet sent 5 weeks before questionnaire 2. No information booklet sent	
Outcomes	Response period not specified	
Topic	Health: nutritional health education leaflet	
Mode of Administration	Postal	
Notes	—	
***Risk of bias***		
**Item**	**Authors' judgement**	**Support for judgement**
Allocation concealment?	No	C ‐ inadequate

**Table:** Nicolaas 2015

***Study characteristics***		
Methods	Random allocation: method unspecified	
Data	Patients registered with a GP practice, UK	
Comparisons	Pre‐notification letter vs. none Reminder postcard vs. none Shorter vs. longer questionnaire	
Outcomes	Final response (2 reminders)	
Topic	Health (the GP Patient Survey; patient experiences and attitudes)	
Mode of Administration	Postal	
Notes	8 pages (4 pages in shorter version). Two reminder packs, each comprising a letter and a copy of questionnaire, sent at monthly intervals to those not yet replying. Patients could complete survey on paper, online or by phone helpline. Pre‐notice letter was sent about one week before the first questionnaire mailing. Postcard reminder was sent to all sample members one week after the initial questionnaire mailing. Could not evaluate the effect of re‐designed cover letter as it included multiple components (i.e. shorter, giving prominence to the NHS England logo, using a high‐status signatory, and including different motivational statements); hence, we only evaluated the pre‐notification letter and the reminder postcard.	
***Risk of bias***		
**Item**	**Authors' judgement**	**Support for judgement**
Sequence generation	Unclear	Random allocation: method unspecified
Allocation concealment?	Unclear	Random allocation: method unspecified
Blinding of participants and personnel	Unclear	Blinding of personnel not described. Participants were not blinded to intervention.
Blinding of outcome assessment	Yes	No outcomes were assessed (counts of responses only).
Incomplete outcome data	No	Exclusions not reported
Selective reporting	Yes	Response reported in full
Other sources of bias	Yes	No grounds for concern about other sources of bias from reading the study report

**Table:** Noel 2018

***Study characteristics***		
Methods	Random allocation: method unspecified	
Data	4638 primary care physicians from the American Medical Association Physician Masterfile	
Comparisons	No incentive vs. smaller monetary incentive (US $2) vs. slightly larger monetary incentive (US $5) vs. larger monetary incentive (US $10)	
Outcomes	Response after 178 days. Participants received one mailed and one telephone reminder (after 1 month).	
Topic	Health (physicians' use of electronic health record (EHR) systems)	
Mode of Administration	Postal	
Notes	Short screener (7 questions). Non‐respondents received a second screener mailing and were called to complete the screener over the phone after 1 month.	
***Risk of bias***		
**Item**	**Authors' judgement**	**Support for judgement**
Sequence generation	Unclear	Random allocation: method unspecified
Allocation concealment?	Unclear	Random allocation: method unspecified
Blinding of participants and personnel	Unclear	Blinding of personnel not described. Participants were not blinded to intervention.
Blinding of outcome assessment	Yes	No outcomes were assessed (counts of responses only).
Incomplete outcome data	No	Exclusions not reported
Selective reporting	Yes	Response after 1 month (2 reminders) reported in full
Other sources of bias	Yes	No grounds for concern about other sources of bias from reading the study report

**Table:** O'Connor 2011

***Study characteristics***		
Methods	Random allocation: method unspecified	
Data	1200 elderly married people (65‐81 years) randomly selected through the Danish Central Person Register	
Comparisons	Shorter vs. longer questionnaire; certified delivery vs. standard; non‐monetary incentive (gift voucher 50DKr/$10) vs. none	
Outcomes	First response	
Topic	Health (depression, social support, coping style, adult attachment, life satisfaction, and personality factors).	
Mode of Administration	Postal	
Notes	133 items in the short questionnaire vs. 318 items in the full questionnaire. The letter had a university letterhead and was personally signed by researcher. No reminders were sent out.	
***Risk of bias***		
**Item**	**Authors' judgement**	**Support for judgement**
Sequence generation	Unclear	Random allocation: method unspecified
Allocation concealment?	Unclear	Random allocation: method unspecified
Blinding of participants and personnel	Unclear	Blinding of personnel not described. Participants were not blinded to intervention.
Blinding of outcome assessment	Yes	No outcomes were assessed (counts of responses only).
Incomplete outcome data	No	Exclusions were not reported.
Selective reporting	Yes	First response reported in full
Other sources of bias	No	Confounding: exclusion of sensitive material in the short questionnaire may also have increased response‐rate and data quality.

**Table:** Ogborne 1986

***Study characteristics***		
Methods	Random allocation: method not specified	
Data	Health and social service professionals who had not responded to an earlier mailing	
Comparisons	1. Second questionnaires sent 2. Telephoned by a research assistant	
Outcomes	Response period not specified	
Topic	Health: evaluation of innovative addiction assessment/referral programme	
Mode of Administration	Postal	
Notes	—	
***Risk of bias***		
**Item**	**Authors' judgement**	**Support for judgement**
Allocation concealment?	Unclear	B ‐ unclear

**Table:** Olivarius 1995

***Study characteristics***		
Methods	Random allocation: method not specified	
Data	GPs and specialists or consultants (Nordic countries)	
Comparisons	1. Questionnaire received on a Friday (GPs) 2. Questionnaire received on a Monday (GPs) 3. Questionnaire received on a Friday (Specialists) 4. Questionnaire received on a Monday (Specialists) Follow‐up sent after 14 and 28 days	
Outcomes	Response within 60 days	
Topic	Health: importance of GPs, treatment of general diseases	
Mode of Administration	Postal	
Notes	—	
***Risk of bias***		
**Item**	**Authors' judgement**	**Support for judgement**
Allocation concealment?	Unclear	B ‐ unclear

**Table:** Olsen 2012

***Study characteristics***		
Methods	Random allocation: alternation	
Data	2400 adults randomly drawn from the Norwegian Population Register	
Comparisons	Non‐monetary incentive (3 EUR scratch card) vs. no incentive	
Outcomes	Response after 6 weeks (one reminder)	
Topic	Health (oral health)	
Mode of Administration	Postal	
Notes	14 questions. A reminder‐questionnaire was mailed to non‐respondents in both groups after 6 weeks.	
***Risk of bias***		
**Item**	**Authors' judgement**	**Support for judgement**
Sequence generation	No	Random allocation: alternation
Allocation concealment?	No	Random allocation: alternation
Blinding of participants and personnel	Unclear	Blinding of personnel not described. Participants were not blinded to intervention.
Blinding of outcome assessment	Yes	No outcomes were assessed (counts of responses only).
Incomplete outcome data	Yes	Exclusions were reported.
Selective reporting	Yes	Response after 6 weeks (one reminder) reported in full
Other sources of bias	Yes	No grounds for concern about other sources of bias from reading the study report

**Table:** Osborne 1996

***Study characteristics***		
Methods	Random allocation: method not specified	
Data	General practitioners	
Comparisons	1. Received pre‐contact telephone call from non‐medical research assistant 2. No pre‐contact	
Outcomes	Response within 60 days	
Topic	Health: views about pathological test ordering	
Mode of Administration	Postal	
Notes	—	
***Risk of bias***		
**Item**	**Authors' judgement**	**Support for judgement**
Allocation concealment?	Unclear	B ‐ unclear

**Table:** Pace 2020

***Study characteristics***		
Methods	Quasi‐randomisation: alternating allocation	
Data	303 primary care providers (PCPs) nominated by study participants to disclose their BRCA1/2 mutation results obtained through the BFOR (BRCA Founder OutReach) study, US	
Comparisons	Unconditional non‐monetary incentive ($50 cheque) vs. unconditional non‐monetary incentive ($50 cash card)	
Outcomes	Response after 1st and 2nd reminders (3 and 6 weeks)	
Topic	Health (primary care physicians' knowledge of BRCA1/2 mutation and opinions on use of genetic testing in their practices)	
Mode of Administration	Postal (electronic option)	
Notes	4 pages (< 10 mins). Each initial survey mailing included a personalised cover letter, and an unconditional $50 incentive. First and second reminders were sent via mail roughly 3 and 6 weeks, respectively, after the initial mailing. These reminders contained personalised letters, a second copy of the survey, and an SAE. Because cheques and registered cash cards can be tracked more easily than cash, they may be preferable to cash or non‐registered cash cards for institutional accounting. Registered cash cards have the additional benefits of being logistically more feasible and efficient than cheques, which must be generated individually for each clinician surveyed. The cash cards were reloadable debit cards that required activation by the study managers before use. GPs received instructions accompanying the card informing them that if they wished to activate the cash card they had to email a study manager with their card number and request card activation.	
***Risk of bias***		
**Item**	**Authors' judgement**	**Support for judgement**
Sequence generation	No	Quasi‐randomisation: alternating allocation
Allocation concealment?	No	Quasi‐randomisation: alternating allocation
Blinding of participants and personnel	Unclear	Blinding of personnel not described. Participants were not blinded to intervention.
Blinding of outcome assessment	Yes	No outcomes were assessed (counts of responses only).
Incomplete outcome data	No	Exclusions not reported
Selective reporting	Yes	Response after 6 weeks (2 reminders) reported in full
Other sources of bias	Yes	No grounds for concern about other sources of bias from reading the study report

**Table:** Paolillo 1984

***Study characteristics***		
Methods	Random allocation: systematic division	
Data	A sample of professionals from the midwestern US	
Comparisons	1. Control group 2. $1 enclosed with questionnaire 3. $2 promised on return of questionnaire 4. Entry into a lottery for a cash prize promised on return of questionnaire	
Outcomes	Response within 6 weeks	
Topic	Not specified	
Mode of Administration	Postal	
Notes	—	
***Risk of bias***		
**Item**	**Authors' judgement**	**Support for judgement**
Allocation concealment?	No	C ‐ inadequate

**Table:** Parasuraman 1981

***Study characteristics***		
Methods	Random allocation: method not specified	
Data	Commercial marketing research firms (US)	
Comparisons	1. Brief cover letter 2. Detailed cover letter	
Outcomes	Response period not specified	
Topic	Non‐health: aspects of marketing research	
Mode of Administration	Postal	
Notes	—	
***Risk of bias***		
**Item**	**Authors' judgement**	**Support for judgement**
Allocation concealment?	Unclear	B ‐ unclear

**Table:** Parker 2019

***Study characteristics***		
Methods	Random allocation: method unspecified	
Data	Participants at 3‐month follow‐up in UK FROST trial of treatments for frozen shoulder in a hospital setting, UK	
Comparisons	SMS pre‐notification on day of mailing vs. SMS post‐notification 4 days following mailing	
Outcomes	Final response	
Topic	Health (Oxford Shoulder Score)	
Mode of Administration	Postal	
Notes	Oxford Shoulder Score (OSS) ‐ 12 questions. Package not described	
***Risk of bias***		
**Item**	**Authors' judgement**	**Support for judgement**
Sequence generation	Unclear	Random allocation: method unspecified
Allocation concealment?	Unclear	Random allocation: method unspecified
Blinding of participants and personnel	Unclear	Blinding of personnel not described. Participants were not blinded to intervention.
Blinding of outcome assessment	Yes	No outcomes were assessed (counts of responses only).
Incomplete outcome data	No	Exclusions not reported
Selective reporting	Yes	Final response reported in full
Other sources of bias	Yes	No grounds for concern about other sources of bias from reading the study report

**Table:** Parkes 2000a

***Study characteristics***		
Methods	Random allocation: method not specified	
Data	Adults aged 20‐74 years who are cases or controls in a Canadian case‐control study of cancer	
Comparisons	1. Enclosure of brochure with questionnaire which expands on the information provided in the covering letter about the survey 2. No brochure	
Outcomes	—	
Topic	Health: tobacco exposure, diet, physical activity, use of medications, reproductive history	
Mode of Administration	Postal	
Notes	A reminder postcard, a reminder letter and second copy of the questionnaire and a follow‐up phone call were made 1‐2,4 and 6 weeks after the initial mailing, respectively, to non‐responders. Age: 20‐74 years	
***Risk of bias***		
**Item**	**Authors' judgement**	**Support for judgement**
Allocation concealment?	Unclear	B ‐ unclear

**Table:** Parkes 2000b

***Study characteristics***		
Methods	Random allocation: method not specified	
Data	Adults aged 20‐74 years who are controls in a Canadian case‐control study of cancer	
Comparisons	1. No incentive 2. $2 sent with questionnaire 3. $5 sent with questionnaire	
Outcomes	—	
Topic	Health: tobacco exposure, diet, physical activity, use of medications, reproductive history	
Mode of Administration	Postal	
Notes	A reminder postcard, a reminder letter and second copy of the questionnaire and a follow‐up phone call were made 1‐2,4 and 6 weeks after the initial mailing, respectively, to non‐responders. Age: 20‐74 years	
***Risk of bias***		
**Item**	**Authors' judgement**	**Support for judgement**
Allocation concealment?	Unclear	B ‐ unclear

**Table:** Parsons 1972a

***Study characteristics***		
Methods	Random allocation: method not specified	
Data	Alumni from a Masters in Business Administration program at a private university	
Comparisons	1. Pre‐notification 2. No pre‐notification	
Outcomes	Response period not specified	
Topic	Non‐health: correlation between political opinions and religious belief	
Mode of Administration	Postal	
Notes	Allocation concealment ascertained through contact with author	
***Risk of bias***		
**Item**	**Authors' judgement**	**Support for judgement**
Allocation concealment?	Yes	A ‐ adequate

**Table:** Parsons 1972b

***Study characteristics***		
Methods	Random allocation: method not specified	
Data	Leaders of 2 religious sects (US)	
Comparisons	1. Pre‐notification 2. No pre‐notification	
Outcomes	Response period not specified	
Topic	Non‐health: correlation between political opinions and religious belief	
Mode of Administration	Postal	
Notes	Allocation concealment ascertained through contact with author	
***Risk of bias***		
**Item**	**Authors' judgement**	**Support for judgement**
Allocation concealment?	Yes	A ‐ adequate

**Table:** Patrick 2013

***Study characteristics***		
Methods	Random allocation: method unspecified	
Data	3000 randomly‐selected undergraduate students, participants of the Student Life Survey at large university in the midwest, US	
Comparisons	Smaller unconditional monetary incentive ($2) plus a larger conditional monetary incentive ($10) vs. larger unconditional monetary incentive ($10) only	
Outcomes	Responses (e‐logins and e‐completions) after 4 reminders	
Topic	Health (college student substance use and related behaviours)	
Mode of Administration	Web survey	
Notes	Questionnaire not described. Students received a mailed pre‐notification letter inviting them to participate and informing them that they would receive an email containing a link to the web‐based survey. Up to 4 reminder emails were sent to non‐responders. Participants were randomly assigned to a condition. However, we note that it is possible that students saw the invitation letters of other students and compared the incentive structures.	
***Risk of bias***		
**Item**	**Authors' judgement**	**Support for judgement**
Sequence generation	Unclear	Random allocation: method unspecified
Allocation concealment?	Unclear	Random allocation: method unspecified
Blinding of participants and personnel	No	It is possible that students saw the invitation letters of other students and compared the incentive structures.
Blinding of outcome assessment	Yes	No outcomes were assessed (counts of responses only).
Incomplete outcome data	No	Exclusions were not reported.
Selective reporting	Yes	Responses (e‐logins and e‐completions) after 4 reminders reported in full
Other sources of bias	Yes	No grounds for concern about other sources of bias from reading the study report

**Table:** Patrick 2018

***Study characteristics***		
Methods	Random allocation: method unspecified	
Data	4950 12th grade students who had completed the national Monitoring the Future (MTF) baseline survey in 2012 or 2013 but had not been selected to participate in the main MTF follow‐up.	
Comparisons	Mail‐only vs. mail push (mail, mail/web reminder) vs. web push (web, web/paper reminder) vs. web push + email augmentation	
Outcomes	Final response	
Topic	Health (substance use amongst adolescents and adults in the US)	
Mode of Administration	Postal/electronic	
Notes	4 pages. Mail‐only: MTF control (standard protocol), with a phone prompt for non‐respondents Mail push: (mail with web reminder) participants were sent the selection letter, newsletter, advance letter, paper questionnaire with a cheque for $25, and a reminder postcard. Each mailing mirrored the MTF main study (i.e. MTF control). The mail push reminder letter reminded participants of the paper questionnaire already sent and gave option to complete the survey online. Non‐response phone calls to all those who had not yet returned a questionnaire provided information about the paper and web response modes. A final mailing included a paper questionnaire and web‐survey option Web push condition: (web with choice reminder) participants were sent the selection letter and newsletter that mirrored the MTF control group, except language implying a paper survey was removed. Web push participants were then sent an advance letter stating that next week they would be sent an invitation to complete an online survey. A week later, they were sent web survey login information (i.e. survey URL and PIN) and a check for $25. The reminder postcard was the same as MTF control except it requested they do the web survey. The reminder letter reminded participants to do the online survey, and the option of completing the enclosed paper questionnaire instead. Non‐response phone calls to all those who had not yet returned a questionnaire provided information about the paper and web response modes. A final mailing included a paper questionnaire and information about the web survey. Web push + email condition (i.e. what Millar and Dillman 2011 call email augmentation), had identical procedures to web push with the addition of emailed versions of the advance letter, web‐survey login information, reminder postcard, and reminder letter. Participants who did not provide a usable email address received the same protocol as web push.	
***Risk of bias***		
**Item**	**Authors' judgement**	**Support for judgement**
Sequence generation	Unclear	Random allocation: method unspecified
Allocation concealment?	Unclear	Random allocation: method unspecified
Blinding of participants and personnel	Unclear	Blinding of personnel not described. Participants were not blinded to intervention.
Blinding of outcome assessment	Yes	No outcome assessment used (counts of responses only)
Incomplete outcome data	No	Exclusions not reported
Selective reporting	Yes	Outcomes reported in full
Other sources of bias	Yes	No grounds for concern about other sources of bias from reading the study report

**Table:** Paul 2005

***Study characteristics***		
Methods	Random allocation: computerised random number generation	
Data	Pharmacists in NSW, Australia, who had sold nicotine replacement therapy (NRT) or bupropion in the last month	
Comparisons	1. Gift voucher worth A$20 2. No voucher	
Outcomes	First response and response after 6 weeks (2 reminders)	
Topic	Health (attitudes toward supporting smoking cessation in the pharmacy environment, and training received regarding nicotine replacement therapy and smoking cessation)	
Mode of Administration	Postal	
Notes	28 items, 10 minutes to complete. The pharmacist in charge of each selected pharmacy was mailed a primer postcard followed by the questionnaire within the following 2 weeks. Non‐responders received one print reminder 4 weeks after receipt of the questionnaire. Telephone reminders were provided a further 2 to 6 weeks later.	
***Risk of bias***		
**Item**	**Authors' judgement**	**Support for judgement**
Sequence generation	Yes	Random allocation: random number generation
Allocation concealment?	Yes	Random allocation: random number generation
Blinding of participants and personnel	No	Neither participants nor personnel were blinded to intervention.
Blinding of outcome assessment	Yes	No outcomes were assessed (counts of responses only).
Incomplete outcome data	Yes	Exclusions were reported.
Selective reporting	Yes	First response and response after 6 weeks (2 reminders) reported in full
Other sources of bias	Yes	No grounds for concern about other sources of bias from reading the study report

**Table:** Pearson 2003

***Study characteristics***		
Methods	Random allocation: using random numbers generated in Excel	
Data	Alumni at Stanford University	
Comparisons	1. Salutation ‐ generic (Dear Stanford Alumni) 2. Salutation ‐ familiar personalisation (Dear James) 3. Salutation ‐ familiar personalisation without the dear (James) 4. Salutation ‐ formal personalised (Dear Mr. Bond)	
Outcomes	Response within 27 days	
Topic	Non‐health: Stanford University's logos, image, and branding	
Mode of Administration	Electronic: Online survey	
Notes	Method of allocation ascertained through contact with author; age: mostly 30‐49 years; mainly males	
***Risk of bias***		
**Item**	**Authors' judgement**	**Support for judgement**
Allocation concealment?	Yes	A ‐ adequate

**Table:** Peck 1981

***Study characteristics***		
Methods	Random allocation: method not specified	
Data	Men and women who had been high school juniors in spring 1973 (US)	
Comparisons	1. Prepaid $3 incentive 2. Promised $3 incentive 3. No incentive	
Outcomes	Response within 5 weeks	
Topic	Non‐health: career plans, labour market, post‐high school educational experience	
Mode of Administration	Postal	
Notes	—	
***Risk of bias***		
**Item**	**Authors' judgement**	**Support for judgement**
Allocation concealment?	Unclear	B ‐ unclear

**Table:** Pedersen 2016

***Study characteristics***		
Methods	Random allocation: method unspecified	
Data	6162 adult members of a Danish non‐probability online panel survey	
Comparisons	Cash prize lottery incentive (300 DKK) vs. conditional charitable donation (3 DKK or 10 DKK) vs. egotistic text appeal vs. altruistic text appeal vs. no incentive	
Outcomes	Response within 2 weeks	
Topic	Various topics (e.g. healthcare, finance, education)	
Mode of Administration	Electronic	
Notes	Questionnaire not described. Email encouraging participation in a brief online survey. No further description of reminders etc. provided	
***Risk of bias***		
**Item**	**Authors' judgement**	**Support for judgement**
Sequence generation	Unclear	Random allocation: method unspecified
Allocation concealment?	Unclear	Random allocation: method unspecified
Blinding of participants and personnel	Unclear	Blinding of personnel not described. Participants were not blinded to intervention.
Blinding of outcome assessment	Yes	No outcomes were assessed (counts of responses only).
Incomplete outcome data	Yes	Exclusions reported
Selective reporting	Yes	Final response reported in full
Other sources of bias	Yes	No grounds for concern about other sources of bias from reading the study report

**Table:** Pedrana 2008

***Study characteristics***		
Methods	Random allocation: method unspecified	
Data	1550 general practitioners in Victoria, Australia	
Comparisons	Registered mail vs. standard mail	
Outcomes	Response after 2 reminders (8 weeks)	
Topic	Health (antenatal screening practice)	
Mode of Administration	Postal	
Notes	10 questions. Reminder letters with a questionnaire and an SAE were sent to non‐responders after 4 weeks. A second reminder was sent to remaining non‐responders after a further 4 weeks.	
***Risk of bias***		
**Item**	**Authors' judgement**	**Support for judgement**
Sequence generation	Unclear	Random allocation: method unspecified
Allocation concealment?	Unclear	Random allocation: method unspecified
Blinding of participants and personnel	Unclear	Blinding of personnel not described. Participants were not blinded to intervention.
Blinding of outcome assessment	Yes	No outcome assessment used (counts of responses only)
Incomplete outcome data	No	Exclusions not reported
Selective reporting	Yes	Outcomes reported in full
Other sources of bias	Yes	No grounds for concern about other sources of bias from reading the study report

**Table:** Pejtersen 2020

***Study characteristics***		
Methods	Random allocation: computer‐generated (Rand function in SAS)	
Data	Children and young people (aged 11‐28) from families with severe social problems, Denmark	
Comparisons	Unconditional non‐monetary incentive (Euro 15 voucher) vs. no incentive	
Outcomes	Response after 13 weeks (one reminder)	
Topic	Health (Strengths and Difficulties Questionnaire)	
Mode of Administration	Postal	
Notes	Approx. 90 items. The questionnaire was sent to both the intervention group and the control group together with a cover letter and an SAE. Addresses on envelopes were handwritten. After 3 weeks, non‐responders in both groups were sent a reminder together with a new copy of the questionnaire.	
***Risk of bias***		
**Item**	**Authors' judgement**	**Support for judgement**
Sequence generation	Yes	Random allocation: computer‐generated (Rand function in SAS)
Allocation concealment?	Yes	Random allocation: computer‐generated (Rand function in SAS)
Blinding of participants and personnel	Unclear	Blinding of personnel not described. Participants were not blinded to intervention.
Blinding of outcome assessment	Yes	No outcomes were assessed (counts of responses only).
Incomplete outcome data	Yes	Exclusions reported
Selective reporting	Yes	Response after 13 weeks (one reminder) reported in full
Other sources of bias	Yes	No grounds for concern about other sources of bias from reading the study report

**Table:** Perneger 1993

***Study characteristics***		
Methods	Random allocation: computerised random number generation	
Data	Young adults enrolled in various insurance plans (US)	
Comparisons	1. No incentive 2. Reminder card 3. Money offer 4. Both incentives Follow‐up with the incentive found to be best after 14 days	
Outcomes	Response within 80 days	
Topic	Health: health status, risk‐taking behaviours, utilisation of health services, satisfaction with healthcare, sociodemographics	
Mode of Administration	Postal	
Notes	Method of allocation ascertained through contact with author; age: mostly 26‐30 years	
***Risk of bias***		
**Item**	**Authors' judgement**	**Support for judgement**
Allocation concealment?	Yes	A ‐ adequate

**Table:** Perry 1974

***Study characteristics***		
Methods	Random allocation: alternation	
Data	Respondents to a previous questionnaire	
Comparisons	1. Questionnaire sent in pre‐paid franked envelope 2. Questionnaire sent in hand‐stamped envelope	
Outcomes	Response period not specified	
Topic	Not specified	
Mode of Administration	Postal	
Notes	—	
***Risk of bias***		
**Item**	**Authors' judgement**	**Support for judgement**
Allocation concealment?	No	C ‐ inadequate

**Table:** Peters 1998

***Study characteristics***		
Methods	Random allocation: method not specified	
Data	People aged 35 and over registered with a general practice (Bristol, UK)	
Comparisons	1. Telephone number requested 2. Telephone number not requested	
Outcomes	Response period not specified	
Topic	Health: information about chronic conditions, sociodemographics	
Mode of Administration	Postal	
Notes	—	
***Risk of bias***		
**Item**	**Authors' judgement**	**Support for judgement**
Allocation concealment?	Unclear	B ‐ unclear

**Table:** Peterson 1975

***Study characteristics***		
Methods	Random allocation: method not specified	
Data	Individuals listed in a telephone directory	
Comparisons	1. University source (U); outgoing envelope (OE) metered; return envelope (RE) stamped; follow‐up postcard (FUP); address (A) typed 2. U; OE‐stamped; RE‐stamped; FUP; A‐typed 3. Business source (B); OE‐metered; RE‐stamped; FUP; A‐typed 4. B; OE‐stamped; RE‐stamped; FUP; A‐typed 5. U; OE‐metered; RE‐stamped; FUP; A‐label 6. U; OE‐stamped; RE‐stamped; FUP; A‐label 7. B; OE‐metered; RE‐stamped; FUP; A‐typed 8. B; OE‐stamped; RE‐stamped; FUP; A‐label 9. U; OE‐metered; RE‐stamped; no FUP; A‐typed 10. U; OE‐stamped; RE‐stamped; no FUP; A‐typed 11. B; OE‐metered; RE‐stamped; no FUP; A‐typed 12. B; OE‐stamped; RE‐stamped; no FUP; A‐typed 13. U; OE‐metered; RE‐stamped; no FUP; A‐label 14. U; OE‐stamped; RE‐stamped; no FUP; A‐label 15. B; OE‐metered; RE‐stamped; no FUP; A‐label 16. B; OE‐stamped; RE‐stamped; no FUP; A‐label 17. U; OE‐metered; RE‐Business reply (reply); FUP, typed 18. U; OE‐stamped; RE‐reply; FUP; A‐typed 19. B; OE‐metered; RE‐reply; FUP; A‐typed 20. B; OE‐stamped; RE‐reply; FUP; A‐typed 21. U; OE‐metered; RE‐reply; FUP; A‐label 22. U; OE‐stamped; RE‐reply; FUP; A‐label 23. B; OE‐metered; RE‐reply; FUP; A‐label 24. B; OE‐stamped; RE‐reply; FUP; A‐label 25. U; OE‐metered; RE‐reply; no FUP; typed 26. U; OE‐stamped; RE‐reply; no FUP; A‐typed 27. B; OE‐metered; RE‐reply; no FUP; A‐typed 28. B; OE‐stamped; RE‐reply; no FUP; A‐typed 29. U; OE‐metered; RE‐reply; no FUP; A‐label 30. U; OE‐stamped; RE‐reply; no FUP; A‐label 31. B; OE‐metered; RE‐reply; no FUP; A‐label 32. B; OE‐stamped; RE‐reply; no FUP; A‐label	
Outcomes	Response within 30 days	
Topic	Non‐health: banking and financial attitudes	
Mode of Administration	Postal	
Notes	—	
***Risk of bias***		
**Item**	**Authors' judgement**	**Support for judgement**
Allocation concealment?	Unclear	B ‐ unclear

**Table:** Petrovčič 2016

***Study characteristics***		
Methods	Random allocation: method unspecified	
Data	2500 members of an online community in Slovenia	
Comparisons	Email sent from senior authority (Chief Editor) vs. no sender specified Email contains plea for help vs. no plea Email stresses benefits for the community vs. no stress	
Outcomes	Response after 2 reminders	
Topic	Health (the role of the online community in their coping with health‐related issues in terms of access to expert and peer support)	
Mode of Administration	Electronic	
Notes	Questionnaire contained a brief informed consent page and four main sections of questions. Median length 16.45 minutes to complete An initial email invitation was sent out to all members informing them of the study, asking them to participate, and providing them with a link to the web survey. Reminders for the second and third contact attempts to non‐responders at 78 and 160 hours	
***Risk of bias***		
**Item**	**Authors' judgement**	**Support for judgement**
Sequence generation	Unclear	Random allocation: method unspecified
Allocation concealment?	Unclear	Random allocation: method unspecified
Blinding of participants and personnel	Unclear	Blinding of personnel not described. Participants were not blinded to intervention.
Blinding of outcome assessment	Yes	No outcomes were assessed (counts of responses only).
Incomplete outcome data	Yes	Exclusions reported
Selective reporting	Yes	Response after 2 reminders reported in full
Other sources of bias	Yes	No grounds for concern about other sources of bias from reading the study report

**Table:** Phillips 1951

***Study characteristics***		
Methods	Random allocation: method not specified	
Data	Graduates of Fisk University in the classes of 1924 and 1939	
Comparisons	1. Follow‐up by first‐class mail 2. Follow‐up by special delivery mail	
Outcomes	Response period not specified	
Topic	Not specified	
Mode of Administration	Postal	
Notes	—	
***Risk of bias***		
**Item**	**Authors' judgement**	**Support for judgement**
Allocation concealment?	Unclear	B ‐ unclear

**Table:** Pirotta 1999

***Study characteristics***		
Methods	Random allocation: method not specified	
Data	General practitioners	
Comparisons	1. Sent primer postcard 5 days before questionnaire 2. Not sent primer postcard before questionnaire	
Outcomes	Response within 60 days	
Topic	Health: not specified	
Mode of Administration	Postal	
Notes	Author contacted: additional unpublished data provided was slightly different to the published report; author data included	
***Risk of bias***		
**Item**	**Authors' judgement**	**Support for judgement**
Allocation concealment?	Unclear	B ‐ unclear

**Table:** Pit 2013

***Study characteristics***		
Methods	Random allocation: computer‐generated	
Data	GPs aged over 45 years, Australia	
Comparisons	Pen vs. no pen	
Outcomes	First response and final response (after 2 reminders ‐ 4 weeks)	
Topic	Non‐health (how to prolong the working lives of GPs)	
Mode of Administration	Postal/electronic	
Notes	3 pages; participants were sent an invitation package consisting of a letter of support from the local GP Network, a study information letter, a questionnaire, an online survey web‐address, and an optional invitation to take part in an interview. Because the surveys were returned anonymously, completed surveys were taken as consent. Two reminders were sent 2 and 4 weeks after the initial invitation. The "nice pen" had a mountain view with clouds, the university's name, and a statement, "Doctors working on" printed on it.	
***Risk of bias***		
**Item**	**Authors' judgement**	**Support for judgement**
Sequence generation	Yes	Random allocation: computer‐generated
Allocation concealment?	Yes	Random allocation: computer‐generated
Blinding of participants and personnel	Yes	Participants were blinded because they were not made aware in advance that the trial was carried out. The sequence allocation was not concealed from the project manager or the GP Network staff. However, participants were unaware of treatment allocation.
Blinding of outcome assessment	Yes	No outcomes were assessed (counts of responses only).
Incomplete outcome data	No	Exclusions were not reported.
Selective reporting	Yes	First response and final response (after 2 reminders ‐ 4 weeks) reported in full
Other sources of bias	Yes	No grounds for concern about other sources of bias from reading the study report

**Table:** Poe 1988

***Study characteristics***		
Methods	Random allocation: method not specified	
Data	Relatives of people who had died and whose death certificates had been filed in September and October 1984	
Comparisons	1. 'Don't know' boxes included 2. 'Don't know' boxes not included	
Outcomes	Response period not specified	
Topic	Health: healthcare in the last year of life, health practices, socioeconomics	
Mode of Administration	Postal	
Notes	—	
***Risk of bias***		
**Item**	**Authors' judgement**	**Support for judgement**
Allocation concealment?	Unclear	B ‐ unclear

**Table:** Porter 2003a

***Study characteristics***		
Methods	Random allocation: using random numbers generated in SAS	
Data	Non‐applicant high school students	
Comparisons	1. Control 2. $50 gift voucher for Amazon.com 3. $100 gift voucher for Amazon.com 4. $150 gift voucher for Amazon.com 5. $200 gift voucher for Amazon.com	
Outcomes	Response period not specified	
Topic	Non‐health: application to college	
Mode of Administration	Electronic: online survey	
Notes	Method of allocation ascertained through contact with author	
***Risk of bias***		
**Item**	**Authors' judgement**	**Support for judgement**
Allocation concealment?	Yes	A ‐ adequate

**Table:** Porter 2003b

***Study characteristics***		
Methods	Random allocation: using random numbers generated in SAS	
Data	Non‐applicant high school students	
Comparisons	1. Salutations impersonal (e.g. Dear Student) 2. Salutations personal (e.g. Dear Jane) 3. Title of signatory, high (Director) 4. Title of signatory, low (Administrative assistant) 5. Sponsorship, low‐profile office (Office of Institutional Research) 6. Sponsorship, high‐profile office (Office of Admission) 7. Source of email address, office (e.g. surveyresearch@institution.edu) 8. Source of email address, person (e.g. jsmith@institution.edu)	
Outcomes	Response period not specified	
Topic	Non‐health: perceptions of the college and the reasons for not applying	
Mode of Administration	Electronic: Online survey	
Notes	Method of allocation ascertained through contact with author	
***Risk of bias***		
**Item**	**Authors' judgement**	**Support for judgement**
Allocation concealment?	Yes	A ‐ adequate

**Table:** Porter 2005a

***Study characteristics***		
Methods	Random allocation: using random numbers generated in SAS	
Data	High school seniors who did not apply to college	
Comparisons	1. Subject‐line ‐ blank 2. Subject‐line ‐ survey 3. Subject‐line ‐ liberal arts university 4. Subject‐line ‐ request for assistance 5. Subject‐line ‐ survey, request for assistance 6. Subject‐line ‐ liberal arts university, request for assistance 7. Subject‐line ‐ liberal arts university, survey 8. Subject‐line ‐ liberal arts university, request for assistance, survey	
Outcomes	Response period not specified	
Topic	Non‐health: perceptions of the school	
Mode of Administration	Electronic: online survey	
Notes	—	
***Risk of bias***		
**Item**	**Authors' judgement**	**Support for judgement**
Allocation concealment?	Unclear	B ‐ unclear

**Table:** Porter 2005b

***Study characteristics***		
Methods	Random allocation: using random numbers generated in SAS	
Data	Undergraduates currently enrolled at the institution	
Comparisons	1. Subject‐line ‐ blank 2. Subject‐line ‐ survey 3. Subject‐line ‐ liberal arts university 4. Subject‐line ‐ request for assistance 5. Subject‐line ‐ survey, request for assistance 6. Subject‐line ‐ liberal arts university, request for assistance 7. Subject‐line ‐ liberal arts university, survey 8. Subject‐line ‐ liberal arts university, request for assistance, survey	
Outcomes	Response period not specified	
Topic	Non‐health: abilities on various capabilities and types of knowledge	
Mode of Administration	Electronic: online survey	
Notes	—	
***Risk of bias***		
**Item**	**Authors' judgement**	**Support for judgement**
Allocation concealment?	Unclear	B ‐ unclear

**Table:** Porter S 2003b

***Study characteristics***		
Methods	Random allocation: using random numbers generated in SAS	
Data	Non‐applicant high school students	
Comparisons	1. Selective statement in the email invitation 2. No selective statement in the email invitation 3. Mention of general deadline in at least 1 email 4. Mention of specific deadline in email 3 5. Mention of specific deadline in emails 2 and 3 6. Mention of specific deadline in emails 1,2, and 3 7. No general deadline 8. No specific deadline	
Outcomes	Response period not specified	
Topic	Non‐health: perceptions of the college and the reasons for not applying	
Mode of Administration	Electronic: online survey	
Notes	Method of allocation ascertained through contact with author	
***Risk of bias***		
**Item**	**Authors' judgement**	**Support for judgement**
Allocation concealment?	Yes	A ‐ adequate

**Table:** Pourjalali 1994

***Study characteristics***		
Methods	Random allocation: random number table	
Data	Students who had participated in an investment game	
Comparisons	1. Investigator perceived to be African‐American (AA); male investigator (MI); informal letter (IL); easier questions first (E1) 2. AA; MI; IL harder questions first (H1) 3. AA; female investigator (FI); IL; E1 4. AA; FI; IL; H1 5. AA; MI; formal letter (FL); E1 6. AA; MI; FL; H1 7. AA; FI; FL; E1 8. AA; FI; FL; H1 9. Investigator perceived to be Caucasian (Ca); MI; IL; E1 10. Ca; MI; IL; H1 11. Ca; FI; IL; E1 12. Ca; FI; IL; H1 13. Ca; MI; FL; E1 14. Ca; MI; FL; H1 15. Ca; FI; FL; E1 16. Ca; FI; FL; H1 17. Investigator perceived to be Hispanic (Hi); MI; IL; E1 18. Hi; MI; IL; H1 19. Hi; FI; IL; E1 20. Hi; FI; IL; H1 21. Hi; MI; FL; E1 22. Hi; MI; FL; H1 23. Hi; FI; FL; E1 24. Hi; FI; FL; H1 25. Investigator perceived to be 'foreign/alien' (Fo); MI; IL; E1 26. Fo; MI; IL; H1 27. Fo; FI; IL; E1 28. Fo; FI; IL; H1 29. Fo; MI; FL; E1 30. Fo; MI; FL; H1 31. Fo; FI; FL; E1 32. Fo; FI; FL; H1	
Outcomes	Response period not specified	
Topic	Non‐health: AT&T investment game	
Mode of Administration	Postal	
Notes	Method of allocation ascertained through contact with author; equal male and females	
***Risk of bias***		
**Item**	**Authors' judgement**	**Support for judgement**
Allocation concealment?	Unclear	B ‐ unclear

**Table:** Powers 1982

***Study characteristics***		
Methods	Random allocation: method not specified	
Data	High school juniors	
Comparisons	1. Feedback offered; long questionnaire 2. Feedback offered; short questionnaire 3. No feedback offered; long questionnaire 4. No feedback, short questionnaire	
Outcomes	Response period not specified	
Topic	Non‐health: students' reaction to the test administration and/or to the preparatory materials for SAT	
Mode of Administration	Postal	
Notes	—	
***Risk of bias***		
**Item**	**Authors' judgement**	**Support for judgement**
Allocation concealment?	Unclear	B ‐ unclear

**Table:** Pressley 1977

***Study characteristics***		
Methods	Random allocation: method not specified	
Data	Marketing research directors	
Comparisons	1. Dime incentive included 2. No incentive 3. Cartoons 4. No cartoons 5. Yellow questionnaire 6. Blue questionnaire 7. Green questionnaire 8. White questionnaire Factorial design Follow‐up sent to non‐respondents after 3 weeks	
Outcomes	Response within 6 weeks.	
Topic	Not specified	
Mode of Administration	Postal	
Notes	—	
***Risk of bias***		
**Item**	**Authors' judgement**	**Support for judgement**
Allocation concealment?	Unclear	B ‐ unclear

**Table:** Pressley 1978

***Study characteristics***		
Methods	Random allocation: method not specified	
Data	Executives employed by organisations located throughout the US	
Comparisons	1. Postscript; deadline 2. No postscript; deadline 3. Postscript; no deadline 4. No postscript; no deadline	
Outcomes	Response period not specified	
Topic	Not specified	
Mode of Administration	Postal	
Notes	—	
***Risk of bias***		
**Item**	**Authors' judgement**	**Support for judgement**
Allocation concealment?	Unclear	B ‐ unclear

**Table:** Pressley 1985

***Study characteristics***		
Methods	Random allocation: method not specified	
Data	Executives at VP‐level in firms employing more than 500 (US)	
Comparisons	1. Mailed on Friday 2. Mailed on Monday 3. Coding handwritten in black ink 4. Coding in invisible ink 5. Coding was typed room number. 6. Telephone pre‐notification without incentive 7. Postcard pre‐notification with $0.10 incentive 8. Sent in window envelope 9. Sent in regular envelope	
Outcomes	Response within 2 weeks	
Topic	Not specified	
Mode of Administration	Postal	
Notes	—	
***Risk of bias***		
**Item**	**Authors' judgement**	**Support for judgement**
Allocation concealment?	Unclear	B ‐ unclear

**Table:** Price 1996

***Study characteristics***		
Methods	Random allocation: method not specified	
Data	A random sample of African‐American women from a midwestern university minority alumni membership list	
Comparisons	1. Race‐specific stamp on return envelope 2. General stamp on return envelope	
Outcomes	Response period not specified	
Topic	Health: cervical cancer	
Mode of Administration	Postal	
Notes	Method of allocation ascertained through contact with author	
***Risk of bias***		
**Item**	**Authors' judgement**	**Support for judgement**
Allocation concealment?	Unclear	B ‐ unclear

**Table:** Price 2003

***Study characteristics***		
Methods	Random allocation: using alternation	
Data	Public health educators and university professors in health education who did not respond to the second mailing	
Comparisons	1. Signed postcard 2. Unsigned postcard	
Outcomes	Response period not specified	
Topic	Health: health education skills	
Mode of Administration	Postal	
Notes	—	
***Risk of bias***		
**Item**	**Authors' judgement**	**Support for judgement**
Allocation concealment?	No	C ‐ inadequate

**Table:** Price 2010

***Study characteristics***		
Methods	Random allocation: computer‐generated	
Data	750 school nurses selected from the National Association of School Nurses membership database, US	
Comparisons	Cover letter signed by one researcher vs. cover letter signed by three researchers	
Outcomes	First response and final response after 2 weeks (one reminder)	
Topic	Health (perceptions and practices of schools assisting students in obtaining public health insurance)	
Mode of Administration	Postal	
Notes	30 items; closed format; 4 pages. Two mailings; both used techniques to maximise response rate including; limiting questionnaire length, demographic questions at the end and first mailing included one dollar monetary incentive and personalised the letter. After 2 wks, the follow‐up mailing was sent with the appropriate covering letters to non‐responders.	
***Risk of bias***		
**Item**	**Authors' judgement**	**Support for judgement**
Sequence generation	Yes	Random allocation: computer‐generated
Allocation concealment?	Yes	Random allocation: computer‐generated
Blinding of participants and personnel	Unclear	Blinding of personnel not described. Participants were not blinded to intervention.
Blinding of outcome assessment	Yes	No outcomes were assessed (counts of responses only).
Incomplete outcome data	Unclear	Exclusions reported
Selective reporting	Yes	First response and final response after 2 weeks (one reminder) reported in full
Other sources of bias	Yes	No grounds for concern about other sources of bias from reading the study report

**Table:** Price 2014

***Study characteristics***		
Methods	Random allocation: computer generated (STATA ralloc command)	
Data	Parents of children diagnosed with autism spectrum disorder	
Comparisons	Unconditional non‐monetary incentive (chocolate frog) vs. no incentive	
Outcomes	Response after 5 weeks (2 reminders)	
Topic	Health (experiences of a child's diagnosis of autism spectrum disorder)	
Mode of Administration	Postal	
Notes	Non‐responders within 3 weeks after the survey was posted received a further survey. After 2 weeks, received up to two telephone reminders thereafter. Survey pack containing information about the study, a 10‐page parent survey and a reply‐paid envelope	
***Risk of bias***		
**Item**	**Authors' judgement**	**Support for judgement**
Sequence generation	Yes	Random allocation: computer‐generated (STATA ralloc command)
Allocation concealment?	Yes	Random allocation: computer‐generated (STATA ralloc command)
Blinding of participants and personnel	Yes	Families and the researchers involved in data collection and entry were blinded to group allocation.
Blinding of outcome assessment	Yes	No outcomes were assessed (counts of responses only).
Incomplete outcome data	No	Exclusions were not reported.
Selective reporting	Yes	Response after 5 weeks (2 reminders) reported in full
Other sources of bias	Yes	No grounds for concern about other sources of bias from reading the study report

**Table:** Pucel 1971

***Study characteristics***		
Methods	Random allocation: method not specified	
Data	Graduates (Minnesota, US)	
Comparisons	1. Control 2. Pencil incentive 3. Coffee incentive 4. Green questionnaire 5. Pre‐notification letter 6. Pencil; green questionnaire 7. Pencil; pre‐notification letter 8. Pencil; green questionnaire; pre‐notification letter 9. Coffee incentive; green questionnaire 10. Coffee incentive; pre‐notification letter 11. Coffee incentive; green questionnaire; pre‐notification letter	
Outcomes	Response within 4 weeks	
Topic	Non‐health: criteria in counselling applicants to post‐high school vocational technical schools	
Mode of Administration	Postal	
Notes	—	
***Risk of bias***		
**Item**	**Authors' judgement**	**Support for judgement**
Allocation concealment?	Unclear	B ‐ unclear

**Table:** Puffer 2004

***Study characteristics***		
Methods	Random allocation: using random number generation in SPSS	
Data	Women who returned the risk factor questionnaire for osteoporotic fracture	
Comparisons	1. Single booklet ‐ 3 sections stapled together 2. Multiple booklet 3. SIngle‐sided 4. Double‐sided	
Outcomes	Response period not specified	
Topic	Health: patient‐based outcome measures concerned with quality of life (SF‐36, EQ‐5D)	
Mode of Administration	Postal	
Notes	—	
***Risk of bias***		
**Item**	**Authors' judgement**	**Support for judgement**
Allocation concealment?	Unclear	B ‐ unclear

**Table:** Rach 2020

***Study characteristics***		
Methods	Random allocation: method unspecified	
Data	3275 participants in the German National Cohort	
Comparisons	Invitation including a study leaflet (brochure) vs. no study leaflet	
Outcomes	Response after 2 reminders	
Topic	Health (general health status, height and weight, selected disease symptoms, use of medication, smoking, menopausal status, and the occurrence of diseases (diagnosed by a physician))	
Mode of Administration	Postal	
Notes	16‐page questionnaire. Invitations sent by mail and included a pre‐stamped return envelope, reminder letters sent at 3, up to 5 telephone calls at week 5‐9 and 2nd final reminder letter	
***Risk of bias***		
**Item**	**Authors' judgement**	**Support for judgement**
Sequence generation	Unclear	Random allocation: method unspecified
Allocation concealment?	Unclear	Random allocation: method unspecified
Blinding of participants and personnel	Unclear	Blinding of personnel not described. Participants were not blinded to intervention.
Blinding of outcome assessment	Yes	No outcome assessment used (counts of responses only)
Incomplete outcome data	Yes	Exclusions reported
Selective reporting	Yes	Outcomes reported in full
Other sources of bias	Yes	No grounds for concern about other sources of bias from reading the study report

**Table:** Rath 2017

***Study characteristics***		
Methods	Random allocation: method unspecified	
Data	Members of the Truth Initiative Young Adult Cohort (adults aged 18 years and older across both the online and offline populations in the US) who had completed one or more surveys prior to Wave 5	
Comparisons	Reminder email including 'humor plus statistics' vs. reminder email including 'humor' only vs. standard email reminder	
Outcomes	Response after 2 reminders	
Topic	Health (tobacco use)	
Mode of Administration	Web survey	
Notes	Questionnaire not described. Those who did not respond to the first reminder were sent a second reminder.	
***Risk of bias***		
**Item**	**Authors' judgement**	**Support for judgement**
Sequence generation	Unclear	Random allocation: method unspecified
Allocation concealment?	Unclear	Random allocation: method unspecified
Blinding of participants and personnel	Unclear	Blinding of personnel not described. Participants were not blinded to intervention.
Blinding of outcome assessment	Yes	No outcomes were assessed (counts of responses only).
Incomplete outcome data	No	Exclusions not reported
Selective reporting	Yes	Response after 2 reminders reported in full
Other sources of bias	Yes	No grounds for concern about other sources of bias from reading the study report

**Table:** Recklitis 2009

***Study characteristics***		
Methods	Random allocation: method unspecified	
Data	Physicians from the Department of Internal Medicine at the University of Colorado Denver School of Medicine, US	
Comparisons	Monetary incentive ($20 bill) vs. non‐monetary incentive (USB flash drive) vs. both monetary ($20 bill) and non‐monetary (USB flash drive) incentives	
Outcomes	Final response after 2 weeks (one reminder)	
Topic	Health (Caring for Cancer Survivors)	
Mode of Administration	Postal/electronic	
Notes	The survey was printed in a colour booklet titled 'Caring for Cancer Survivors: Challenge and Opportunity'. The cover letter stated the survey was for primary care physicians and asked participants to complete the paper survey or an online version at a web address provided. Four days later, an email was sent to all potential participants for whom an email address was known, with an invitation to complete the survey online with a web link provided in the e‐mail. Two weeks after the initial mailing, a reminder letter and a second survey were sent to non‐responders by US mail, and a reminder email was sent to non‐responders with a known email address. The study design did not include a 'No incentive' arm. Survey packets including a cover letter, survey, and incentives were sent via Federal Express 2nd Day Airmail. Further details of the package were not reported.	
***Risk of bias***		
**Item**	**Authors' judgement**	**Support for judgement**
Sequence generation	Unclear	Random allocation: method unspecified
Allocation concealment?	Unclear	Random allocation: method unspecified
Blinding of participants and personnel	Unclear	Blinding of personnel not described. Participants were not blinded to intervention.
Blinding of outcome assessment	Yes	No outcome assessment used (counts of responses only)
Incomplete outcome data	No	Exclusions not reported
Selective reporting	Yes	Outcomes reported in full
Other sources of bias	Yes	No grounds for concern about other sources of bias from reading the study report

**Table:** Rego 2020

***Study characteristics***		
Methods	Random allocation: computer generated (Microsoft Excel's RAND function)	
Data	Adults who cared for at least one child between 6 and 60 months, had access to their own mobile phone in three informal settlements in Mwanza, Tanzania	
Comparisons	Unconditional non‐monetary incentive ($0¬∑4 US airtime payment) vs. no incentive Shorter (1 question) vs. longer (3 questions) Daily vs. fortnightly messaging	
Outcomes	Response after 2 reminders	
Topic	Health (infant diarrhoea rates)	
Mode of Administration	SMS survey	
Notes	1 question vs. 3 questions. A text message, formatted according to the randomisation schedule, was sent via SMS message to participants between 10 a.m. and 11 a.m. on days due. If participants did not respond or complete the survey, they would receive two reminders, one after 4 h, and the second after a further 4 h. Responses were not accepted beyond 12 h from the initial message.	
***Risk of bias***		
**Item**	**Authors' judgement**	**Support for judgement**
Sequence generation	Yes	Random allocation: computer‐generated (Microsoft Excel's RAND function)
Allocation concealment?	Yes	Random allocation: computer‐generated (Microsoft Excel's RAND function)
Blinding of participants and personnel	Yes	Participants were blinded to their arm allocation.
Blinding of outcome assessment	Yes	No outcomes were assessed (counts of responses only).
Incomplete outcome data	Yes	Exclusions reported
Selective reporting	Yes	Response after 2 reminders reported in full
Other sources of bias	Yes	No grounds for concern about other sources of bias from reading the study report

**Table:** Reinisch 2016

***Study characteristics***		
Methods	Random allocation: method unspecified	
Data	Members of the American Association of Plastic Surgeons	
Comparisons	Postal vs. electronic; smaller ($1 bill) vs. larger monetary incentive ($5 bill)	
Outcomes	Response after 2 weeks and response after 10 weeks	
Topic	Non‐health (authorship issues)	
Mode of Administration	Postal/electronic	
Notes	Follow‐up after 2 weeks with reminder. After further 2‐week period, the remaining non‐responders contacted by phone, offered survey by fax or mail or email. After 2 additional weeks (6 weeks after initial contact), non‐responders, regardless of group assignment, were mailed a survey.	
***Risk of bias***		
**Item**	**Authors' judgement**	**Support for judgement**
Sequence generation	Unclear	Random allocation: method unspecified
Allocation concealment?	Unclear	Random allocation: method unspecified
Blinding of participants and personnel	Unclear	Blinding of personnel not described. Participants were not blinded to intervention.
Blinding of outcome assessment	Yes	No outcome assessment used (counts of responses only)
Incomplete outcome data	Yes	Exclusions reported
Selective reporting	Yes	Outcomes reported in full
Other sources of bias	Yes	No grounds for concern about other sources of bias from reading the study report

**Table:** Renfroe 2002

***Study characteristics***		
Methods	Random allocation: method not specified	
Data	Participants of the AVID trial, USA	
Comparisons	1. Mailed out by overnight express (OE); certificate of appreciation included (CA); mailed early (E); signature of Principal Investigator on cover letter (PI) 2. OE; CA; E; signature of Study Coordinator on cover letter (SC) 3. OE; CA; mailed late (L); PI 4. OE; CA; L; SC 5. OE; No CA; E; PI 6. OE; No CA; E; SC 7. OE; No CA; L; PI 8. OE; No CA; L; SC 9. Mailed out by regular mail (RM); CA; E; PI 10. RM; CA; E; SC 11. RM; CA; L; PI 12. RM; CA; L; SC 13. RM; No CA; E; PI 14. RM; No CA; E; SC 15. RM; No CA; L; PI 16. RM; No CA; L; SC	
Outcomes	Response period not specified	
Topic	Health: patient satisfaction	
Mode of Administration	Postal	
Notes	Mean age: 63; mainly males	
***Risk of bias***		
**Item**	**Authors' judgement**	**Support for judgement**
Allocation concealment?	Unclear	B ‐ unclear

**Table:** Riesenberg 2006

***Study characteristics***		
Methods	Random allocation: method not specified	
Data	Designated Institutional Official (DIO)	
Comparisons	1. Priority stamps worth $3.85 2. First‐class stamps worth $0.60	
Outcomes	Response period not specified	
Topic	Non‐health: employment ‐ demographics, identification of roles and responsibilities, competencies, training and experience required by the DIO	
Mode of Administration	Postal	
Notes	—	
***Risk of bias***		
**Item**	**Authors' judgement**	**Support for judgement**
Allocation concealment?	Unclear	B ‐ unclear

**Table:** Rikard‐Bell 2000

***Study characteristics***		
Methods	Random allocation: computerised random number generation	
Data	Dentists practising within the central Sydney area, Australia in 1997	
Comparisons	1. Advance telephone prompt 2. Advance letter prompt	
Outcomes	Response within 65 days	
Topic	Health: not specified	
Mode of Administration	Postal	
Notes	—	
***Risk of bias***		
**Item**	**Authors' judgement**	**Support for judgement**
Allocation concealment?	Yes	A ‐ adequate

**Table:** Rimm 1990

***Study characteristics***		
Methods	Random allocation: computerised random number generation	
Data	Male health professionals who had not responded to a previous questionnaire	
Comparisons	1. Certified mail 2. United Parcel Service 3. Window envelope with personal return address 4. Typed address 5. Handwritten address 6. Window envelope with computer printed address Factorial design	
Outcomes	—	
Topic	Health: medical history, current diet and lifestyle habits	
Mode of Administration	Postal	
Notes	Method of allocation ascertained through contact with author; age: 40‐75; mainly males	
***Risk of bias***		
**Item**	**Authors' judgement**	**Support for judgement**
Allocation concealment?	Yes	A ‐ adequate

**Table:** Robb 2017

***Study characteristics***		
Methods	Random allocation: method unspecified	
Data	3872 people aged 45‐59 years, registered with one of 4 general practices in southEast England	
Comparisons	Shorter vs. longer questionnaire No incentive vs. conditional non‐monetary incentive (£2.50 voucher) vs. conditional non‐monetary incentive (£5 voucher) vs. conditional non‐monetary incentive (£250 voucher lottery)	
Outcomes	Response after one reminder	
Topic	Health (health and attitudes towards bowel cancer screening)	
Mode of Administration	Postal	
Notes	Shorter questionnaire of 4 A4 pages or longer one of 7 A4 pages. Participants were mailed a letter from their inviting GP, the study questionnaire, and a freepost reply envelope. Non‐responders were sent a reminder questionnaire after approximately 2 weeks.	
***Risk of bias***		
**Item**	**Authors' judgement**	**Support for judgement**
Sequence generation	Unclear	Random allocation: method unspecified
Allocation concealment?	Unclear	Random allocation: method unspecified
Blinding of participants and personnel	Unclear	Blinding of personnel not described. Participants were not blinded to intervention.
Blinding of outcome assessment	Yes	No outcomes were assessed (counts of responses only).
Incomplete outcome data	Yes	Exclusions reported
Selective reporting	Yes	Response after one reminder reported in full
Other sources of bias	Yes	No grounds for concern about other sources of bias from reading the study report

**Table:** Roberts 1978

***Study characteristics***		
Methods	Random allocation: computerised random number generation	
Data	General practitioners who were members of the American Dental Association	
Comparisons	1. Personalised; social appeal; deadline 2. Personalised; social appeal; no deadline 3. Personalised; no social appeal; deadline 4. Personalised; no social appeal; no deadline 5. Not personalised; social appeal; deadline 6. Not personalised; social appeal; no deadline 7. Not personalised; no social appeal; deadline 8. Not personalised; no social appeal; no deadline	
Outcomes	Response period not specified	
Topic	Not specified	
Mode of Administration	Postal	
Notes	Method of allocation ascertained through contact with author. Informed that allocation concealment was adequate	
***Risk of bias***		
**Item**	**Authors' judgement**	**Support for judgement**
Allocation concealment?	Yes	A ‐ adequate

**Table:** Roberts 1993

***Study characteristics***		
Methods	Random allocation: method not specified	
Data	Adults listed on a family health services authority register who had not responded to a previous questionnaire	
Comparisons	1. First reminder was another copy of questionnaire 2. First reminder was a postcard	
Outcomes	Response period not specified	
Topic	Health: health and lifestyle	
Mode of Administration	Postal	
Notes	Age: 16‐70 years	
***Risk of bias***		
**Item**	**Authors' judgement**	**Support for judgement**
Allocation concealment?	Unclear	B ‐ unclear

**Table:** Roberts 1994

***Study characteristics***		
Methods	Random allocation: method not specified	
Data	Nurses (Auckland, New Zealand)	
Comparisons	1. Brown re‐usable envelope out; brown re‐usable envelope return 2. Brown re‐usable envelope out; white non‐reusable envelope return 3. White non‐reusable envelope out; brown re‐usable envelope return 4. White non‐reusable envelope out; white non‐reusable envelope return Reminders sent using the same envelope combination as initially allocated	
Outcomes	Response period not specified	
Topic	Health: prevalence of back pain	
Mode of Administration	Postal	
Notes	Mean age: 37.5 years	
***Risk of bias***		
**Item**	**Authors' judgement**	**Support for judgement**
Allocation concealment?	Unclear	B ‐ unclear

**Table:** Roberts 2000

***Study characteristics***		
Methods	Random allocation: method not specified	
Data	1000 English women aged 40 to 65 years	
Comparisons	1. Entry into lottery for prize draw of £50 on response 2. Direct payment of £5 on response 3. Entry into lottery and direct payment of £5 on response 4. No incentive	
Outcomes	Response within 3 months	
Topic	Health: menopause services	
Mode of Administration	Postal	
Notes	Mainly females	
***Risk of bias***		
**Item**	**Authors' judgement**	**Support for judgement**
Allocation concealment?	Unclear	B ‐ unclear

**Table:** Roberts 2004

***Study characteristics***		
Methods	Random allocation: computerised random number generation	
Data	General practices in the North and West Birmingham area	
Comparisons	1. Lottery to win high street shopping voucher worth £100 2. Control	
Outcomes	Response period not specified	
Topic	Health: prevalence of IBS (Irritable Bowel Syndrome) using SF‐36, Rome II criteria	
Mode of Administration	Postal	
Notes	Mean age: 48 years	
***Risk of bias***		
**Item**	**Authors' judgement**	**Support for judgement**
Allocation concealment?	Yes	A ‐ adequate

**Table:** Robertson 1978

***Study characteristics***		
Methods	Random allocation: method not specified	
Data	A systematic sample of Denver area residents listed in the Metropolitan area phone directory	
Comparisons	1. Control; no incentive 2. Promise of $1 cash on return of questionnaire 3. Promise of $1 donation to charity on return of questionnaire	
Outcomes	Response period not specified	
Topic	Not specified	
Mode of Administration	Postal	
Notes	Method of allocation ascertained through contact with author	
***Risk of bias***		
**Item**	**Authors' judgement**	**Support for judgement**
Allocation concealment?	Unclear	B ‐ unclear

**Table:** Robertson 2005

***Study characteristics***		
Methods	Random allocation: using alternation	
Data	Australian general practitioners and medical specialists	
Comparisons	1. $ AU 2 scratch lottery ticket 2. No incentive	
Outcomes	Response period not specified	
Topic	Heath: exploring new drug use by GPs and medical specialists	
Mode of Administration	Postal	
Notes	Method of allocation ascertained through contact with author	
***Risk of bias***		
**Item**	**Authors' judgement**	**Support for judgement**
Allocation concealment?	Yes	A ‐ adequate

**Table:** Rodgers 2018

***Study characteristics***		
Methods	Random allocation: computer‐generated	
Data	Men and women aged over 65 who have fallen and live in the community, UK	
Comparisons	Study newsletter vs. no newsletter Personalised (handwritten) Post‐it vs. less personalised (printed) Post‐it vs. no Post‐it	
Outcomes	Response after 6 weeks (2 reminders)	
Topic	Health (falls prevention in elderly adults)	
Mode of Administration	Postal	
Notes	Questionnaire not described. The newsletter was posted to participants 3 weeks prior to posting the 12‐month questionnaire. Those participants randomised to not receive the newsletter were sent this eight weeks after the questionnaire was sent out. Date postal questionnaires sent was recorded and non‐responders within 2 weeks were sent up to two standard reminders, 2 weeks apart, by post, text or email according to the participant's preference, followed by a telephone reminder 1 week later. The newsletter contained information regarding trial progress, including the geographical location and number of participants recruited and what happens at the end of the study. The newsletter was posted to participants 3 weeks prior to posting the 12‐month questionnaire. Those participants randomised to not receive the newsletter were sent this 8 weeks after the questionnaire was sent out. The Post‐it note was placed on the top right hand corner of the questionnaire. Participants who did not return their questionnaire within 2 weeks were sent up to two reminders, 2 weeks apart, by post, text or email according to the participant's preference, followed by a telephone reminder 1 week later. All participants received an unconditional £5 note with their final follow‐up.	
***Risk of bias***		
**Item**	**Authors' judgement**	**Support for judgement**
Sequence generation	Yes	Random allocation: computer‐generated
Allocation concealment?	Yes	Random allocation: computer‐generated
Blinding of participants and personnel	Unclear	Blinding of personnel not described. Participants were not blinded to intervention.
Blinding of outcome assessment	Yes	No outcomes were assessed (counts of responses only).
Incomplete outcome data	No	Exclusions not reported
Selective reporting	Yes	Response after 6 weeks (2 reminders) reported in full
Other sources of bias	Yes	No grounds for concern about other sources of bias from reading the study report

**Table:** Rolnick 1989

***Study characteristics***		
Methods	Random allocation: method not specified	
Data	Women with sexually transmitted diseases	
Comparisons	1. Detailed questionnaire 2. Modified questionnaire	
Outcomes	Response within 2 months	
Topic	Health: gynaecological issues	
Mode of Administration	Postal	
Notes	Age: 18‐28 years; mainly females	
***Risk of bias***		
**Item**	**Authors' judgement**	**Support for judgement**
Allocation concealment?	Unclear	B ‐ unclear

**Table:** Romney 1993

***Study characteristics***		
Methods	Random allocation: method not specified	
Data	Community educators	
Comparisons	1. Open‐ended format 2. Closed‐ended format	
Outcomes	Response period not specified	
Topic	Non‐health: community educational needs assessment instrument	
Mode of Administration	Postal	
Notes	—	
***Risk of bias***		
**Item**	**Authors' judgement**	**Support for judgement**
Allocation concealment?	Unclear	B ‐ unclear

**Table:** Ronckers 2004

***Study characteristics***		
Methods	Random allocation: method not specified	
Data	Dutch patients treated for ENT condition between 1945 and 1981	
Comparisons	1. Short questionnaire (8 pages) 2. Long questionnaire (12 pages) 3. Standard consent form 4. Multi‐option consent form (choices with regard to participation in 3 phases of the overall study)	
Outcomes	Response period not specified	
Topic	Health: female reproductive history, occupational exposures, and diet	
Mode of Administration	Postal	
Notes	—	
***Risk of bias***		
**Item**	**Authors' judgement**	**Support for judgement**
Allocation concealment?	Unclear	B ‐ unclear

**Table:** Roscoe 1975

***Study characteristics***		
Methods	Random allocation: method not specified	
Data	Random sample of telephone customers	
Comparisons	1. Long questionnaire; postcard reminder follow‐up 2. Long questionnaire; telephone reminder follow‐up 3. Short questionnaire; postcard reminder follow‐up 4. Short questionnaire; telephone reminder follow‐up	
Outcomes	Response period not specified	
Topic	Non‐health: telephone behaviours, housing, mobility, demographics, socioeconomic characteristics	
Mode of Administration	Postal	
Notes	Method of allocation ascertained through contact with author	
***Risk of bias***		
**Item**	**Authors' judgement**	**Support for judgement**
Allocation concealment?	Unclear	B ‐ unclear

**Table:** Rose 2007a

***Study characteristics***		
Methods	Random allocation: using random numbers chart	
Data	Employees of a large international retailer in the US	
Comparisons	1. $1 bill 2. No incentive	
Outcomes	Response period within 10 days	
Topic	Non‐health: related to marketing skills, management, and leadership qualities	
Mode of Administration	Postal	
Notes	Surveys with incentives enclosed were identified by a one‐inch‐long, one‐fourth‐inch‐wide yellow highlighter mark within a half inch of both edges of the lower left corner of the back side of the survey. Also, one‐fourth inch of the non‐highlighted corner at the bottom of the survey was cut off.	
***Risk of bias***		
**Item**	**Authors' judgement**	**Support for judgement**
Allocation concealment?	Unclear	B ‐ unclear

**Table:** Rose 2007b

***Study characteristics***		
Methods	Random allocation: using random numbers chart	
Data	Employees of a large health care organisation in the US	
Comparisons	1. Low novelty ‐ plain bill 2. High Novelty ‐ bill with small star sticker or Sacagawea gold dollar coin 3. A penny bill 4. A quarter bill 5. A dollar bill 6. No incentive	
Outcomes	Response within 21 days	
Topic	Non‐health: training needs	
Mode of Administration	Postal	
Notes	Surveys with incentives enclosed has a one‐inch‐long, one‐fourth‐inch‐wide highlighted mark within a half inch of both edges of the lower left corner of the back side of the survey. Packets with pennies had orange marks, packets with quarters had yellow marks, packets with paper dollars had blue marks, and packets with Sacagawea dollars had green highlighter marks. In addition, one‐fourth inch of the non‐highlighted corner at the bottom of the survey was cut off.	
***Risk of bias***		
**Item**	**Authors' judgement**	**Support for judgement**
Allocation concealment?	Unclear	B ‐ unclear

**Table:** Rosoff 2005a

***Study characteristics***		
Methods	Random allocation: random block procedure	
Data	Childhood cancer survivors	
Comparisons	1. Unconditional $10 bill 2. Conditional $10 bill	
Outcomes	Response period not specified	
Topic	Health: health‐related behaviours amongst childhood cancer survivors and their parents	
Mode of Administration	Postal	
Notes	—	
***Risk of bias***		
**Item**	**Authors' judgement**	**Support for judgement**
Allocation concealment?	Unclear	B ‐ unclear

**Table:** Rosoff 2005b

***Study characteristics***		
Methods	Random allocation: random block procedure	
Data	Childhood cancer survivors	
Comparisons	1. Unconditional $10 bill 2. Conditional $10 bill	
Outcomes	Response period not specified	
Topic	Health: health‐related behaviours amongst childhood cancer survivors and their parents	
Mode of Administration	Postal	
Notes	—	
***Risk of bias***		
**Item**	**Authors' judgement**	**Support for judgement**
Allocation concealment?	Unclear	B ‐ unclear

**Table:** Rosoff 2005c

***Study characteristics***		
Methods	Random allocation: random block procedure	
Data	Parents of childhood cancer survivors	
Comparisons	1. Unconditional $10 bill 2. Conditional $10 bill	
Outcomes	Response period not specified	
Topic	Health: health‐related behaviours amongst childhood cancer survivors and their parents	
Mode of Administration	Postal	
Notes	—	
***Risk of bias***		
**Item**	**Authors' judgement**	**Support for judgement**
Allocation concealment?	Unclear	B ‐ unclear

**Table:** Roszkowski 1990a

***Study characteristics***		
Methods	Random allocation: method not specified	
Data	Students studying Wealth Accumulation Planning	
Comparisons	1. Long questionnaire 2. Short questionnaire Follow‐up sent to non‐respondents of first mailing	
Outcomes	—	
Topic	Non‐health: evaluation of financial courses	
Mode of Administration	Postal	
Notes	—	
***Risk of bias***		
**Item**	**Authors' judgement**	**Support for judgement**
Allocation concealment?	Unclear	B ‐ unclear

**Table:** Roszkowski 1990b

***Study characteristics***		
Methods	Random allocation: method not specified	
Data	Students studying Financial Services: Environment and Professions	
Comparisons	1. Long questionnaire 2. Short questionnaire Follow‐up sent to non‐respondents of first mailing	
Outcomes	—	
Topic	Non‐health: evaluation of financial courses	
Mode of Administration	Postal	
Notes	—	
***Risk of bias***		
**Item**	**Authors' judgement**	**Support for judgement**
Allocation concealment?	Unclear	B ‐ unclear

**Table:** Roszkowski 1990c

***Study characteristics***		
Methods	Random allocation: method not specified	
Data	Students studying Estate and Gift Tax Planning	
Comparisons	1. Long questionnaire 2. Short questionnaire Follow‐up sent to non‐respondents of first mailing	
Outcomes	—	
Topic	Non‐health: evaluation of financial courses	
Mode of Administration	Postal	
Notes	—	
***Risk of bias***		
**Item**	**Authors' judgement**	**Support for judgement**
Allocation concealment?	Unclear	B ‐ unclear

**Table:** Roszkowski 1990d

***Study characteristics***		
Methods	Random allocation: method not specified	
Data	Students studying Wealth Accumulation Planning	
Comparisons	1. Long questionnaire 2. Short questionnaire Follow‐up sent to non‐respondents of first mailing	
Outcomes	—	
Topic	Non‐health: evaluation of financial courses	
Mode of Administration	Postal	
Notes	—	
***Risk of bias***		
**Item**	**Authors' judgement**	**Support for judgement**
Allocation concealment?	Unclear	B ‐ unclear

**Table:** Roszkowski 1990e

***Study characteristics***		
Methods	Random allocation: method not specified	
Data	Students studying Economics	
Comparisons	1. Long questionnaire 2. Short questionnaire Follow‐up sent to non‐respondents of first mailing	
Outcomes	—	
Topic	Non‐health: evaluation of financial courses	
Mode of Administration	Postal	
Notes	—	
***Risk of bias***		
**Item**	**Authors' judgement**	**Support for judgement**
Allocation concealment?	Unclear	B ‐ unclear

**Table:** Roszkowski 1990f

***Study characteristics***		
Methods	Random allocation: method not specified	
Data	Students studying Pensions and Other Retirement Plans	
Comparisons	1. Long questionnaire 2. Short questionnaire Follow‐up sent to non‐respondents of first mailing	
Outcomes	—	
Topic	Non‐health: evaluation of financial courses	
Mode of Administration	Postal	
Notes	—	
***Risk of bias***		
**Item**	**Authors' judgement**	**Support for judgement**
Allocation concealment?	Unclear	B ‐ unclear

**Table:** Roszkowski 1990g

***Study characteristics***		
Methods	Random allocation: method not specified	
Data	Students studying Advanced Estate Planning	
Comparisons	1. Long questionnaire 2. Short questionnaire Follow‐up sent to non‐respondents of first mailing	
Outcomes	—	
Topic	Non‐health: evaluation of financial courses	
Mode of Administration	Postal	
Notes	—	
***Risk of bias***		
**Item**	**Authors' judgement**	**Support for judgement**
Allocation concealment?	Unclear	B ‐ unclear

**Table:** Roszkowski 1990h

***Study characteristics***		
Methods	Random allocation: method not specified	
Data	Students studying Financial Statement Analysis	
Comparisons	1. Long questionnaire 2. Short questionnaire Follow‐up sent to non‐respondents of first mailing	
Outcomes	—	
Topic	Non‐health: evaluation of financial courses	
Mode of Administration	Postal	
Notes	—	
***Risk of bias***		
**Item**	**Authors' judgement**	**Support for judgement**
Allocation concealment?	Unclear	B ‐ unclear

**Table:** Roszkowski 1990i

***Study characteristics***		
Methods	Random allocation: method not specified	
Data	Students studying Group Benefits and Social Insurance	
Comparisons	1. Long questionnaire 2. Short questionnaire Follow‐up sent to non‐respondents of first mailing	
Outcomes	—	
Topic	Non‐health: evaluation of financial courses	
Mode of Administration	Postal	
Notes	—	
***Risk of bias***		
**Item**	**Authors' judgement**	**Support for judgement**
Allocation concealment?	Unclear	B ‐ unclear

**Table:** Roszkowski 1990j

***Study characteristics***		
Methods	Random allocation: method not specified	
Data	Students studying Planning for Business Owners and Professionals	
Comparisons	1. Long questionnaire 2. Short questionnaire Follow‐up sent to non‐respondents of first mailing	
Outcomes		
Topic	Non‐health: evaluation of financial courses	
Mode of Administration	Postal	
Notes	—	
***Risk of bias***		
**Item**	**Authors' judgement**	**Support for judgement**
Allocation concealment?	Unclear	B ‐ unclear

**Table:** Roszkowski 1990k

***Study characteristics***		
Methods	Random allocation: method not specified	
Data	Students studying Financial Statement Analysis	
Comparisons	1. Long questionnaire 2. Short questionnaire Follow‐up sent to non‐respondents of first mailing	
Outcomes	—	
Topic	Non‐health: evaluation of financial courses	
Mode of Administration	Postal	
Notes	—	
***Risk of bias***		
**Item**	**Authors' judgement**	**Support for judgement**
Allocation concealment?	Unclear	B ‐ unclear

**Table:** Roszkowski 1990l

***Study characteristics***		
Methods	Random allocation: method not specified	
Data	Students studying Financial and Estate Planning	
Comparisons	1. Long questionnaire 2. Short questionnaire Follow‐up sent to non‐respondents of first mailing	
Outcomes	—	
Topic	Non‐health: evaluation of financial courses	
Mode of Administration	Postal	
Notes	—	
***Risk of bias***		
**Item**	**Authors' judgement**	**Support for judgement**
Allocation concealment?	Unclear	B ‐ unclear

**Table:** Roszkowski 1990m

***Study characteristics***		
Methods	Random allocation: method not specified	
Data	Students studying Financial and Estate planning	
Comparisons	1. Long questionnaire 2. Short questionnaire Follow‐up sent to non‐respondents of first mailing	
Outcomes	—	
Topic	Non‐health: evaluation of financial courses	
Mode of Administration	Postal	
Notes	—	
***Risk of bias***		
**Item**	**Authors' judgement**	**Support for judgement**
Allocation concealment?	Unclear	B ‐ unclear

**Table:** Roszkowski 1990n

***Study characteristics***		
Methods	Random allocation: method not specified	
Data	Students studying Group Benefits and Social Insurance	
Comparisons	1. Long questionnaire 2. Short questionnaire Follow‐up sent to non‐respondents of first mailing	
Outcomes	—	
Topic	Non‐health: evaluation of financial courses	
Mode of Administration	Postal	
Notes	—	
***Risk of bias***		
**Item**	**Authors' judgement**	**Support for judgement**
Allocation concealment?	Unclear	B ‐ unclear

**Table:** Rucker 1979a

***Study characteristics***		
Methods	Random allocation: method not specified	
Data	Students	
Comparisons	1. Standard questionnaire 2. Matrix questionnaire Follow‐up sent to non‐respondents after 10 days	
Outcomes	Response within 2 months	
Topic	Non‐health: attitudes towards purchasing clothes	
Mode of Administration	Postal	
Notes	—	
***Risk of bias***		
**Item**	**Authors' judgement**	**Support for judgement**
Allocation concealment?	Unclear	B ‐ unclear

**Table:** Rucker 1979b

***Study characteristics***		
Methods	Random allocation: method not specified	
Data	Students	
Comparisons	1. Standard questionnaire 2. Matrix questionnaire Follow‐up sent to non‐respondents after 10 days	
Outcomes	Response within 1 month	
Topic	Non‐health: clothing attitudes	
Mode of Administration	Postal	
Notes	—	
***Risk of bias***		
**Item**	**Authors' judgement**	**Support for judgement**
Allocation concealment?	Unclear	B ‐ unclear

**Table:** Rucker 1984

***Study characteristics***		
Methods	Random allocation: method not specified	
Data	Graduates	
Comparisons	1. Textiles student sponsor; no photo of person on cover letter 2. Textiles student sponsor; casually dressed person on cover letter 3. Textiles student sponsor; formal dressed person on cover letter 4. Textiles professor sponsor; no photo of person on cover letter 5. Textiles professor sponsor; casually dressed person on cover letter 6. Textiles professor sponsor; formal dressed person on cover letter 7. Animal science student sponsor; no photo of person on cover letter 8. Animal science student sponsor; casually dressed person on cover letter 9. Animal science student sponsor; formal dressed person on cover letter 10. Animal science professor sponsor; no photo of person on cover letter 11. Animal science professor sponsor; casually dressed person on cover letter 12. Animal science professor sponsor; formal dressed person on cover letter Postcard reminder and second questionnaire sent to non‐respondents at approximately bimonthly intervals	
Outcomes	—	
Topic	Non‐health: furniture opinion	
Mode of Administration	Postal	
Notes	—	
***Risk of bias***		
**Item**	**Authors' judgement**	**Support for judgement**
Allocation concealment?	Unclear	B ‐ unclear

**Table:** Russell 2003

***Study characteristics***		
Methods	Random allocation: computerised random number generation	
Data	Chiropractors registered with the College of Chiropractors of Alberta	
Comparisons	1. Unconditional $5 bill 2. No incentive	
Outcomes	Response period not specified	
Topic	Health: immunisation beliefs and behaviours of chiropractors	
Mode of Administration	Postal	
Notes	—	
***Risk of bias***		
**Item**	**Authors' judgement**	**Support for judgement**
Allocation concealment?	Unclear	B ‐ unclear

**Table:** Ryu 2006

***Study characteristics***		
Methods	Random allocation: using alternation	
Data	Detroit Area Study (DAS) 2001	
Comparisons	1. Cash ($5 bill) 2. In‐kind (set of passes to regional parks, or metro parks)	
Outcomes	Response period not specified	
Topic	Health: quality of life in the Metropolitan Detroit Area	
Mode of Administration	Postal	
Notes	Method of allocation ascertained through contact with author	
***Risk of bias***		
**Item**	**Authors' judgement**	**Support for judgement**
Allocation concealment?	Yes	A ‐ adequate

**Table:** Saal 2005

***Study characteristics***		
Methods	Random allocation: using alternation	
Data	Inpatients admitted for elective surgery at the St. Gallen Cantonal Hospital	
Comparisons	1. Questionnaire sent 1 week after discharge 2. Questionnaire sent 5 weeks after discharge 3. Questionnaire sent 9 weeks after discharge	
Outcomes	Response period not specified	
Topic	Health: patients' assessment of anaesthesia care	
Mode of Administration	Postal	
Notes	Survey was conducted by an independent organisation, the Picker Institute	
***Risk of bias***		
**Item**	**Authors' judgement**	**Support for judgement**
Allocation concealment?	Yes	A ‐ adequate

**Table:** Sahlqvist 2011

***Study characteristics***		
Methods	Random allocation: computer‐generated	
Data	1000 participants randomly selected from the UK electoral register	
Comparisons	More vs. less personalised; shorter vs. longer questionnaire	
Outcomes	First response	
Topic	Health (travel behaviour, physical activity and the environment)	
Mode of Administration	Postal	
Notes	The long questionnaire was 24 A4 pages and consisted of seven sections; the short questionnaire covered the same general constructs but was reduced to six sections and 15 A4 pages. Pre‐notification postcard for all groups, survey pack 1 week later with letter, information sheet, consent, questionnaire and freepost return envelope. After 2 weeks, non‐responders sent reminder postcard or reminder pack (depending on further randomisation). Conditional prize draw for £25 gift voucher for all participants and thank you postcard	
***Risk of bias***		
**Item**	**Authors' judgement**	**Support for judgement**
Sequence generation	Yes	Random allocation: computer‐generated
Allocation concealment?	Yes	Random allocation: computer‐generated
Blinding of participants and personnel	Yes	Participants were blind to their allocation status and to the fact that these survey design factors were the subject of a randomised controlled trial. Study co‐ordinators charged with receipting the return of completed surveys were not aware of a respondent's allocation status in terms of personalisation and reminder type. Nonetheless, they could not be fully blinded to a respondents' allocation status due to the different lengths (and therefore weights) of the two questionnaires.
Blinding of outcome assessment	Yes	No outcomes were assessed (counts of responses only).
Incomplete outcome data	No	Exclusions were not reported.
Selective reporting	Yes	First response reported in full
Other sources of bias	Yes	No grounds for concern about other sources of bias from reading the study report

**Table:** Sakshaug 2019

***Study characteristics***		
Methods	Random allocation: method unspecified	
Data	Public and private establishments that had previously participated in a Job Vacancy Survey and were registered as employing staff in at least one of 25 target professions	
Comparisons	Email invitation to web survey (T3 + T4) vs. postal (T1 + T2); postal vs electronic (T1 vs. T4)	
Outcomes	First response and response after one reminder	
Topic	Non‐health (factors that influence establishments‚ decision‐making process for filling job vacancies)	
Mode of Administration	Web survey	
Notes	Questionnaire not described. All invitations were addressed to the human resources (HRs) office or managerial board of the establishments. The invitation letters included the salutation 'Dear sir or madam' (even in the case of personalised email addresses) in the hope that the invitation would be forwarded to an HR officer or any person responsible for hiring. Postal and email reminders were sent at the same time according to the assigned treatment group.	
***Risk of bias***		
**Item**	**Authors' judgement**	**Support for judgement**
Sequence generation	Unclear	Random allocation: method unspecified
Allocation concealment?	Unclear	Random allocation: method unspecified
Blinding of participants and personnel	Unclear	Blinding of personnel not described. Participants were not blinded to intervention.
Blinding of outcome assessment	Yes	No outcomes were assessed (counts of responses only).
Incomplete outcome data	Yes	Exclusions reported
Selective reporting	Yes	First response and response after one reminder reported in full
Other sources of bias	Yes	No grounds for concern about other sources of bias from reading the study report

**Table:** Salim Silva 2002

***Study characteristics***		
Methods	Random allocation: method not specified	
Data	2 female Australian samples. Sample A ‐ current office workers at a university. Sample B ‐ patients seen by a consultant in rehab medicine	
Comparisons	1. Telephone reminder 2. No telephone reminder	
Outcomes	—	
Topic	Health: musculoskeletal symptoms, health service utilisation, tobacco and alcohol consumption, social support, occupational history and job satisfaction, general health, sociodemographics	
Mode of Administration	Postal	
Notes	Age: Mostly above 45 years; mainly females	
***Risk of bias***		
**Item**	**Authors' judgement**	**Support for judgement**
Allocation concealment?	Unclear	B ‐ unclear

**Table:** Sallis 1984

***Study characteristics***		
Methods	Random allocation: method not specified	
Data	Physicians who had not responded to a previous questionnaire (Monterey County)	
Comparisons	1. No incentive 2. Pencil incentive printed with an attractive design	
Outcomes	Response period not specified	
Topic	Health: not specified	
Mode of Administration	Postal	
Notes	—	
***Risk of bias***		
**Item**	**Authors' judgement**	**Support for judgement**
Allocation concealment?	Unclear	B ‐ unclear

**Table:** Salvesen 1992

***Study characteristics***		
Methods	Random allocation: using a table of random numbers	
Data	Mothers who had not responded to a previous questionnaire	
Comparisons	1. Newspaper article with description of the study 2. No article sent with the questionnaire	
Outcomes	Response within 30 days	
Topic	Health: child's health ‐ hearing, vision	
Mode of Administration	Postal	
Notes	Method of allocation ascertained through contact with author. Allocation was not concealed; mainly females	
***Risk of bias***		
**Item**	**Authors' judgement**	**Support for judgement**
Allocation concealment?	No	C ‐ inadequate

**Table:** Sang‐Wook 2005

***Study characteristics***		
Methods	Random allocation: using random numbers generated in SAS	
Data	Korean‐Vietnam veterans	
Comparisons	1. Questionnaires sent via recorded delivery 2. Questionnaires sent via standard delivery 3. Stamped‐return envelope 4. Franked‐return envelope	
Outcomes	Response period not specified	
Topic	Health: veterans socioeconomic and health status, medical check‐up	
Mode of Administration	Postal	
Notes	—	
***Risk of bias***		
**Item**	**Authors' judgement**	**Support for judgement**
Allocation concealment?	Unclear	B ‐ unclear

**Table:** Satia 2005

***Study characteristics***		
Methods	Random allocation: method unspecified	
Data	4276 African‐Americans aged 18 to 70 years, residing in 6 contiguous North Carolina counties (3 urban and 3 rural), from Department of Motor Vehicle rosters, US	
Comparisons	Culturally sensitive approach letter vs. generic; unconditional vs. conditional non‐monetary incentive; non‐monetary incentive vs. no incentive	
Outcomes	Response after one reminder (3 weeks)	
Topic	Health (cancer prevention)	
Mode of Administration	Postal with choice (mail, web, telephone)	
Notes	11 pages; printed in black and pink colour ink and designed to be attractive and interesting to potential respondents; all responses designed to be categorical. All prospective participants were sent pre‐notification postcards followed by questionnaires; the enclosed approach letter and questionnaire cover page provided information on how to participate by telephone or Internet instead. Thus, respondents selected their preferred completion method. A reminder letter was sent 2‐3 weeks later with a toll‐free number to call for a replacement questionnaire or to complete the survey by telephone and the URL for the survey website. The culturally sensitive letter was similar to the generic version but was designed to increase respondents' ethnic/cultural identification with the study by including the principal investigator's picture to identify her as African‐American. The letter also noted the paucity of information on health issues specific to African‐Americans and stressed the potential benefit of participation to others.	
***Risk of bias***		
**Item**	**Authors' judgement**	**Support for judgement**
Sequence generation	Unclear	Random allocation: method unspecified
Allocation concealment?	Unclear	Random allocation: method unspecified
Blinding of participants and personnel	Unclear	Blinding of personnel not described. Participants were not blinded to intervention.
Blinding of outcome assessment	Yes	No outcomes were assessed (counts of responses only).
Incomplete outcome data	Yes	Exclusions were reported (but not by an assigned intervention group).
Selective reporting	Yes	Response after one reminder (3 weeks) reported in full
Other sources of bias	Yes	No grounds for concern about other sources of bias from reading the study report

**Table:** Sauerland 2002

***Study characteristics***		
Methods	Random allocation: alternation	
Data	All members of the association of German surgeons	
Comparisons	1. Hernia and pain questionnaires sent together in 1 letter 2. Hernia questionnaire sent first, pain questionnaire sent 4 weeks later 3. Pain questionnaire sent first, hernia questionnaire sent 4 weeks later	
Outcomes	Response period not specified	
Topic	Health: perioperative pain management, surgical technique in incisional hernia repair	
Mode of Administration	Postal	
Notes	—	
***Risk of bias***		
**Item**	**Authors' judgement**	**Support for judgement**
Allocation concealment?	No	C ‐ inadequate

**Table:** Schmidt 2005

***Study characteristics***		
Methods	Random allocation: using random numbers generated in SPSS	
Data	Product Development and Management Association (PDMA) members	
Comparisons	1. Certified mail on outward mailing 2. First‐class mail on outward mailing	
Outcomes	Response period not specified	
Topic	Non‐health: development of new products by various organisations	
Mode of Administration	Postal	
Notes	—

**Table:** Schmuhl 2010

***Study characteristics***		
Methods	Random allocation: method unspecified	
Data	Emergency medical services (EMS) providers with current certification in Utah, US	
Comparisons	Paper only vs. Web only vs. choice (paper or Web)	
Outcomes	Response within 30 days with 2 reminder postcards (mailed 2 weeks apart)	
Topic	Health (Utah Emergency Services for Children needs assessment)	
Mode of Administration	Postal and electronic (Web survey)	
Notes	The survey contained 31 questions (17 multiple‐choice, eight fill‐in‐the‐blank or check all, and six open‐ended) regarding the topics of availability of paediatric equipment, EMS educational opportunities, cultural needs, injury prevention, and children with special healthcare needs. Questionnaire package not described. The Web survey contained the same 31 questions presented in the same order as the paper survey. The main difference was the decision to make closed‐ended questions required, meaning that individuals could not move on to the next question without answering the current question.	
***Risk of bias***		
**Item**	**Authors' judgement**	**Support for judgement**
Sequence generation	Unclear	Random allocation: method unspecified
Allocation concealment?	Unclear	Random allocation: method unspecified
Blinding of participants and personnel	Yes	All participants were blinded to the different survey modes, and the surveys were anonymous.
Blinding of outcome assessment	Yes	No outcomes were assessed (counts of responses only).
Incomplete outcome data	Yes	Exclusions reported: 212 surveys were deemed undeliverable by the post office and, as a result, 1661 surveys were included in this study.
Selective reporting	Yes	Final response reported in full
Other sources of bias	Yes	No grounds for concern about other sources of bias from reading the study report

**Table:** Schwartzenberger 2017

***Study characteristics***		
Methods	Random allocation: method unspecified	
Data	969 patients who underwent endoscopic carpal tunnel release	
Comparisons	Postal vs. electronic	
Outcomes	Response 1 year after surgery	
Topic	Health (patient‐reported outcomes 1 year following carpal tunnel release)	
Mode of Administration	Postal/electronic/telephone	
Notes	23‐question survey. In the traditional mail cohort, the study questionnaire along with instructions and a self‐addressed return envelope were sent. Patients whose questionnaire was not returned by 4 weeks were sent a second mailing at that time with contents identical to the first and given another 4 weeks to respond. Patients who did not respond by 8 weeks were considered non‐responders. In the email cohort, surveys were administered and collected data were managed using the Research Electronic Data Capture (REDCap) tools hosted at our institution. Patients were sent an email containing instructions to click on a link that automatically opened the Web‐based survey. Patients who had not responded to the first email by 4 weeks were sent a second identical email at that time and given another 4 weeks to respond. Patients who did not respond by 8 weeks were considered non‐responders. Telephone cohort not considered in this review	
***Risk of bias***		
**Item**	**Authors' judgement**	**Support for judgement**
Sequence generation	Unclear	Random allocation: method unspecified
Allocation concealment?	Unclear	Random allocation: method unspecified
Blinding of participants and personnel	Unclear	Blinding of personnel not described. Participants were not blinded to intervention.
Blinding of outcome assessment	Yes	No outcomes were assessed (counts of responses only).
Incomplete outcome data	Yes	Exclusions were reported.
Selective reporting	Yes	Response 1 year after surgery reported in full
Other sources of bias	Yes	No grounds for concern about other sources of bias from reading the study report

**Table:** Schweitzer 1995

***Study characteristics***		
Methods	Random allocation: method not specified	
Data	University staff employed for at least 6 years (Pennsylvania, US)	
Comparisons	1. Non‐form fillers; paid in advance 2. Non‐form fillers; paid on completion 3. Form‐fillers; paid in advance 4. Non‐form fillers; paid on completion Reminder sent to non‐respondents after 4 weeks	
Outcomes	—	
Topic	Health: knowledge, attitudes, and behaviours regarding the selection of employee health benefits	
Mode of Administration	Postal	
Notes	Age: 45‐48 years	
***Risk of bias***		
**Item**	**Authors' judgement**	**Support for judgement**
Allocation concealment?	Unclear	B ‐ unclear

**Table:** Scott 1957

***Study characteristics***		
Methods	Random allocation: alternation	
Data	Women aged 60 years and over from poll tax exemption lists for Travis County, Texas, USA, 1954	
Comparisons	1. Preliminary letter received on Monday, questionnaire received on Tuesday 2. Preliminary letter received on Monday, questionnaire received on Friday 3. No preliminary letter, questionnaire received on Wednesday 4. No preliminary letter, questionnaire received on Saturday	
Outcomes	Response period not specified	
Topic	Non‐health: occupational history, present income/pension payment	
Mode of Administration	Postal	
Notes	Age: above 60 years; mainly females	
***Risk of bias***		
**Item**	**Authors' judgement**	**Support for judgement**
Allocation concealment?	No	C ‐ inadequate

**Table:** Scott 2011

***Study characteristics***		
Methods	Random allocation: random numbers generated in STATA	
Data	Doctors undertaking clinical practice drawn from a national directory of all doctors in Australia	
Comparisons	Electronic vs. mailed personal invitation letter to web survey ('sequential mode') vs. choice (postal/electronic)	
Outcomes	Response after 3 weeks (one reminder)	
Topic	Non‐health (Balancing Employment and Life ‐workforce participation and its determinants amongst Australian doctors)	
Mode of Administration	Postal/electronic	
Notes	8 sections; 4 slight varied versions for different doctor types. 58 questions in 8‐page booklet (specialists in training) to 87 questions in 13‐page booklet (for specialists) Survey invitation letters indicated the University of Melbourne and Monash University as responsible for the survey.	
***Risk of bias***		
**Item**	**Authors' judgement**	**Support for judgement**
Sequence generation	Yes	Random allocation: random numbers generated in STATA
Allocation concealment?	Yes	Random allocation: random numbers generated in STATA
Blinding of participants and personnel	No	Neither personnel nor participants were blinded to intervention.
Blinding of outcome assessment	Yes	No outcomes were assessed (counts of responses only).
Incomplete outcome data	Yes	Exclusions were reported.
Selective reporting	Yes	Response after 3 weeks (one reminder) reported in full
Other sources of bias	Yes	No grounds for concern about other sources of bias from reading the study report

**Table:** Sebo 2017

***Study characteristics***		
Methods	Random allocation: computer‐generated	
Data	Community‐based GPs with a valid and available email address	
Comparisons	Postal vs electronic	
Outcomes	First response and final response after one (postal group) or two (Web group) reminders	
Topic	Health (GPs' preventive care activities)	
Mode of Administration	Postal vs. Web	
Notes	Questionnaire included 37 questions. The postal letters included a cover letter and a stamped return envelope. GPs were asked to send the completed questionnaires back to the research assistant. Participants in the Web‐based group received the same cover letter in a Web‐based format. GPs were asked to connect through a hyperlink and complete the online questionnaire. The paper questionnaire was designed first, closely following published recommendations for optimal survey content and layout. The Web‐based questionnaire was then created to be as similar as possible to the paper version, including regarding text formatting. Note that completion of all questions was not required before submission of the Web‐based questionnaire. Reminder messages (once for the postal group and twice for the Web‐based group) were sent at one‐month intervals. No monetary incentives were offered to the participating GPs. All community‐based GPs practising were eligible for the study, except those practising only complementary and alternative medicine. There were no other exclusion criteria.	
***Risk of bias***		
**Item**	**Authors' judgement**	**Support for judgement**
Sequence generation	Yes	Random allocation: computer‐generated
Allocation concealment?	Yes	Random allocation: computer‐generated
Blinding of participants and personnel	Unclear	Blinding of personnel not described. Participants were not blinded to intervention.
Blinding of outcome assessment	Yes	No outcome assessment used (counts of responses only)
Incomplete outcome data	No	Exclusions not reported
Selective reporting	Yes	Outcomes reported in full
Other sources of bias	Yes	No grounds for concern about other sources of bias from reading the study report

**Table:** See Tai 1997

***Study characteristics***		
Methods	Random allocation: method not specified	
Data	Patients who had not responded to a questionnaire (London, UK)	
Comparisons	1. Questionnaire reminder 2. Telephone reminder	
Outcomes	Response period not specified	
Topic	Health: to evaluate the use of structural computerised prompts in their management using Asthma Symptoms Questionnaire & Client Satisfaction Questionnaire (for patients with asthma), and Well‐being Questionnaire and Diabetes Treatment Questionnaire (for patients with diabetes)	
Mode of Administration	Postal	
Notes	Mean age: telephone group ‐ 47.5 years; recorded delivery group ‐ 40 years	
***Risk of bias***		
**Item**	**Authors' judgement**	**Support for judgement**
Allocation concealment?	Unclear	B ‐ unclear

**Table:** Severi 2011a

***Study characteristics***		
Methods	Random allocation: computer‐generated (Minim software)	
Data	Participants in the Txt2stop trial of smoking cessation support who were due follow‐up	
Comparisons	Stress benefits to society of participation‐up vs. no stress	
Outcomes	Response at 26 and 30 weeks	
Topic	Health (smoking)	
Mode of Administration	Postal	
Notes	Questionnaire not described, package not described RCT evaluating the impact of providing information regarding the benefits to society of participation. In addition to the standard trial follow‐up procedures, the intervention group was sent written information on a refrigerator magnet by post, between 16 and 20 weeks after randomisation into the Txt2stop trial, followed by a mobile phone text message 3 days after theTxt2stop postal follow‐up questionnaire was sent. The text message said 'Be proud of yourself for helping medical research! Thank you for filling in the Txt2stop questionnaire.'	
***Risk of bias***		
**Item**	**Authors' judgement**	**Support for judgement**
Sequence generation	Yes	Random allocation: computer‐generated (Minim software)
Allocation concealment?	Yes	Random allocation: computer‐generated (Minim software)
Blinding of participants and personnel	Yes	This was a single‐blind controlled trial, with those recording and assessing outcomes blind to the intervention.
Blinding of outcome assessment	Yes	No outcomes were assessed (counts of responses only).
Incomplete outcome data	Yes	Exclusions reported
Selective reporting	Yes	Final response reported in full
Other sources of bias	Yes	No grounds for concern about other sources of bias from reading the study report

**Table:** Severi 2011b

***Study characteristics***		
Methods	Random allocation: computer‐generated (Minim software)	
Data	Participants in the Txt2stop trial of smoking cessation support who were due follow‐up	
Comparisons	Follow‐up telephone call vs. no call	
Outcomes	Final response	
Topic	Health (smoking)	
Mode of Administration	Postal	
Notes	Questionnaire not described, package not described	
***Risk of bias***		
**Item**	**Authors' judgement**	**Support for judgement**
Sequence generation	Yes	Random allocation: computer‐generated (Minim software)
Allocation concealment?	Yes	Random allocation: computer‐generated (Minim software)
Blinding of participants and personnel	Yes	This was a single‐blind controlled trial, with those recording and assessing outcomes blind to the intervention.
Blinding of outcome assessment	Yes	No outcomes were assessed (counts of responses only).
Incomplete outcome data	Yes	Exclusions reported
Selective reporting	Yes	Final response reported in full
Other sources of bias	Yes	No grounds for concern about other sources of bias from reading the study report

**Table:** Shackleton 1980

***Study characteristics***		
Methods	Random allocation: method not specified	
Data	Partially sighted school‐leavers aged between 17 and 20 years who had left schools for the visually handicapped during the previous academic year	
Comparisons	1. £1 offered; previous examination 2. No incentive; previous examination 3. £1 offered; no examination 4. No incentive; no examination	
Outcomes	Response within 42 days	
Topic	Non‐health: occupational experience during 1st year after leaving the school	
Mode of Administration	Postal	
Notes	Method of allocation ascertained through contact with author; age: 17‐20 years	
***Risk of bias***		
**Item**	**Authors' judgement**	**Support for judgement**
Allocation concealment?	Unclear	B ‐ unclear

**Table:** Shah 2001

***Study characteristics***		
Methods	Random allocation: method not specified	
Data	Patients aged 65 to 74 years in an inner London practice who had consulted within the last 2 years	
Comparisons	1. Inclusion of questions on income; inclusion of consent form 2. Inclusion of questions on income; no consent form 3. No questions on income; inclusion of consent form 4. No questions on income; no consent form	
Outcomes	—	
Topic	Health: physical and mental health, social circumstances, social support, living arrangements, income	
Mode of Administration	Postal	
Notes	Age: 65‐74 years	
***Risk of bias***		
**Item**	**Authors' judgement**	**Support for judgement**
Allocation concealment?	Unclear	B ‐ unclear

**Table:** Shahar 1993

***Study characteristics***		
Methods	Random allocation: method not specified	
Data	Individuals who had declined to participate in a previous study	
Comparisons	1. Additional letter with first mailing requesting an explanation for not participating 2. No letter	
Outcomes	Response within 14 weeks	
Topic	Health: general health, physical activity, smoking habits, list of chronic disease, demographics	
Mode of Administration	Postal	
Notes	—	
***Risk of bias***		
**Item**	**Authors' judgement**	**Support for judgement**
Allocation concealment?	Unclear	B ‐ unclear

**Table:** Sharp 2006

***Study characteristics***		
Methods	Random allocation: computerised random number generation	
Data	Participants from the TOMBOLA (Trial Of Management of Borderline and Other Low‐grade Abnormal smears) trial	
Comparisons	1. Pen 2. No pen 3. First‐class dispatch 4. Second‐class dispatch 5. Freepost (business reply) envelope 6. Postage stamp envelope	
Outcomes	Response period not specified	
Topic	Health: psychosocial impact of having a low‐grade abnormal cervical smear and its subsequent management	
Mode of Administration	Postal	
Notes	—	
***Risk of bias***		
**Item**	**Authors' judgement**	**Support for judgement**
Allocation concealment?	Unclear	B ‐ unclear

**Table:** Shaw 2001

***Study characteristics***		
Methods	Random allocation: method not specified	
Data	Health System Minnesota enrollees aged 20‐80 years	
Comparisons	1. $5 included in survey package 2. $2 included in survey package	
Outcomes	—	
Topic	Health: Digestive Health Status instrument (DHS I), SF‐36, HADS, comorbidity checklist, healthcare utilisation	
Mode of Administration	Postal	
Notes	Age: 20‐80 years	
***Risk of bias***		
**Item**	**Authors' judgement**	**Support for judgement**
Allocation concealment?	Unclear	B ‐ unclear

**Table:** Sheikh 1982

***Study characteristics***		
Methods	Random allocation: method not specified	
Data	400 people who had completed an assessment course at an employment rehabilitation centre in London 1973‐1974	
Comparisons	1. Questionnaire including sensitive question on earnings 2. Same questionnaire as (1) without the sensitive question on earnings	
Outcomes	—	
Topic	Non‐health: employment	
Mode of Administration	Postal	
Notes	Mean age: 39 years; mainly females	
***Risk of bias***		
**Item**	**Authors' judgement**	**Support for judgement**
Allocation concealment?	Unclear	B ‐ unclear

**Table:** Shin 1992

***Study characteristics***		
Methods	Random allocation: method not specified	
Data	Faculty members in universities and 4 4‐year colleges in the United States	
Comparisons	1. Personalised (P); anonymous (A); professional appeal (Prof); university sponsored (U) questionnaire 2. P; A; Prof; private research institute sponsored (PR) 3. P; A; personal appeal (Pers); U 4. P; A; Pers; PR 5. P; Nonanonymous (NA); Prof; U 6. P; NA; Prof; PR 7. P; NA; Pers; U 8. P; NA; Pers; PR 9. Not personalised (Not P); A; Prof; U 10. Not P; A; Prof; PR 11. Not P; A; Pers; U 12. Not P; A; Pers; PR 13. Not P; NA; Prof; U 14. Not P; NA; Prof; PR 15. Not P; NA; Pers; U 16. Not P; NA; Pers; PR	
Outcomes	Response within 7 weeks	
Topic	Non‐health: student evaluation of faculty instruction	
Mode of Administration	Postal	
Notes	—	
***Risk of bias***		
**Item**	**Authors' judgement**	**Support for judgement**
Allocation concealment?	Unclear	B ‐ unclear

**Table:** Shiono 1991

***Study characteristics***		
Methods	Random allocation: method not specified	
Data	Physicians (US)	
Comparisons	1. Pre‐notification letter; stamp on return envelope 2. Pre‐notification letter; return envelope franked 3. No pre‐notification letter; stamp on return envelope 4. No pre‐notification letter; return envelope franked	
Outcomes		
Topic	Health: pregnancy amongst resident physicians	
Mode of Administration	Postal	
Notes	—	
***Risk of bias***		
**Item**	**Authors' judgement**	**Support for judgement**
Allocation concealment?	Unclear	B ‐ unclear

**Table:** Signorelli 2021

***Study characteristics***		
Methods	Random allocation: online computer‐generated (Research Randomizer tool)	
Data	Childhood cancer survivors and parents of survivors < 16 years from 11 hospitals across Australia/New Zealand	
Comparisons	Non‐monetary incentive (USB stick with video invitation) vs. no incentive	
Outcomes	Returned questionnaires	
Topic	Health (health, well‐being, information needs and health‐related quality of life)	
Mode of Administration	Choice	
Notes	Questionnaire assessed participants' health/well‐being, information needs and health‐related quality of life (using the EQ‐5D‐5L); included clinical data, relapse history, age and demographic information; 30 mins completion Study invitation packages contained a signed invitation letter from each survivor's treating oncologist, an information sheet, a questionnaire, a reply‐paid envelope and a card with a web‐link to complete the questionnaire online, if preferred. Letter invitation (control) vs. letter plus video invitation on a USB stick from survivor/paediatric oncologist/researcher. We conducted up to 4 follow‐up reminder calls to non‐respondents, 2 weeks apart, after mailout of the initial invitation and resent invitation packages up to a further 2 times on request.	
***Risk of bias***		
**Item**	**Authors' judgement**	**Support for judgement**
Sequence generation	Yes	Random allocation: online computer‐generated (Research Randomizer tool)
Allocation concealment?	Yes	Random allocation: online computer‐generated (Research Randomizer tool)
Blinding of participants and personnel	Unclear	Blinding of personnel not described. Participants were not blinded to intervention.
Blinding of outcome assessment	Yes	No outcomes were assessed (counts of responses only).
Incomplete outcome data	Yes	Exclusions reported
Selective reporting	Yes	Response after 4 reminders reported in full
Other sources of bias	Yes	No grounds for concern about other sources of bias from reading the study report

**Table:** Simon 1967a

***Study characteristics***		
Methods	Random allocation: alternation	
Data	Readers of a magazine published by a national industrial company	
Comparisons	1. Personal letter 2. Form letter	
Outcomes	Response period not specified	
Topic	Health: attitudes towards a hospital insurance plan	
Mode of Administration	Postal	
Notes	Author contacted: unable to provide further details on randomisation	
***Risk of bias***		
**Item**	**Authors' judgement**	**Support for judgement**
Allocation concealment?	No	C ‐ inadequate

**Table:** Simon 1967b

***Study characteristics***		
Methods	Random allocation: alternation	
Data	Readers of a magazine published by a national industrial company	
Comparisons	1. Personal letter 2. Form letter	
Outcomes	Response period not specified	
Topic	Health: attitudes towards a hospital insurance plan	
Mode of Administration	Postal	
Notes	Author contacted: unable to provide further details on randomisation	
***Risk of bias***		
**Item**	**Authors' judgement**	**Support for judgement**
Allocation concealment?	No	C ‐ inadequate

**Table:** Simon 1967c

***Study characteristics***		
Methods	Random allocation: alternation	
Data	Subscribers to a hospital insurance plan	
Comparisons	1. Personal letter 2. Form letter	
Outcomes	Response period not specified	
Topic	Health: attitudes towards a hospital insurance plan	
Mode of Administration	Postal	
Notes	Author contacted: unable to provide further details on randomisation	
***Risk of bias***		
**Item**	**Authors' judgement**	**Support for judgement**
Allocation concealment?	No	C ‐ inadequate

**Table:** Sizmur 2019

***Study characteristics***		
Methods	Random allocation: method unspecified	
Data	Five NHS trusts (12,723 invited staff), all participating in the main NHS staff survey for 2015 using online methodology	
Comparisons	More email reminders vs. fewer Sent or signed by more well‐known person vs. less well‐known person Included statement that others had responded vs. no statement	
Outcomes	Final response after reminders	
Topic	Health (staff experience, including health and well‐being, personal development, harassment and bullying, engagement and satisfaction, and support from managers)	
Mode of Administration	Web survey	
Notes	33 questions over 7 pages. Email reminders were sent approximately every 1.5 weeks.	
***Risk of bias***		
**Item**	**Authors' judgement**	**Support for judgement**
Sequence generation	Unclear	Random allocation: method unspecified
Allocation concealment?	Unclear	Random allocation: method unspecified
Blinding of participants and personnel	Unclear	Blinding of personnel not described. Participants were not blinded to intervention.
Blinding of outcome assessment	Yes	No outcomes were assessed (counts of responses only).
Incomplete outcome data	No	Exclusions not reported, but excluded from analysis
Selective reporting	Yes	Final response after reminders reported in full
Other sources of bias	Yes	No grounds for concern about other sources of bias from reading the study report

**Table:** Skinner 1984

***Study characteristics***		
Methods	Random allocation: method not specified	
Data	Marketing professors, Canada	
Comparisons	1. No incentive 2. $1 pre‐paid incentive 3. $1 promised incentive; respondent identified 4. $1 promised incentive; respondent not identified 5. $1 promised to charity	
Outcomes	Response period not specified	
Topic	Non‐health: needs of Canadian instructors regarding an introductory marketing text	
Mode of Administration	Postal	
Notes	—	
***Risk of bias***		
**Item**	**Authors' judgement**	**Support for judgement**
Allocation concealment?	Unclear	B ‐ unclear

**Table:** Sletto 1940

***Study characteristics***		
Methods	Random allocation: alternation	
Data	Former university students	
Comparisons	1. 10‐page questionnaire; altruistic appeal in cover letter 2. 10‐page questionnaire; cover letter requesting help 3. 10‐page questionnaire; cover letter challenging participants to respond 4. 25‐page questionnaire; altruistic appeal in cover letter 5. 25‐page questionnaire; cover letter requesting help 6. 25‐page questionnaire; cover letter challenging participants to respond 7. 35‐page questionnaire (10 and 25‐page questionnaires); altruistic appeal in cover letter 8. 35‐page questionnaire (10 and 25‐page questionnaires); cover letter requesting help 9. 35‐page questionnaire (10 and 25‐page questionnaires); cover letter challenging participants to respond	
Outcomes	Response period not specified	
Topic	Non‐health: vocational activities, needs, interest, socio‐civic activities	
Mode of Administration	Postal	
Notes	—	
***Risk of bias***		
**Item**	**Authors' judgement**	**Support for judgement**
Allocation concealment?	No	C ‐ inadequate

**Table:** Sloan 1997

***Study characteristics***		
Methods	Random allocation: method not specified	
Data	Doctors of patients with cancer	
Comparisons	1. University letterhead (UL); MD signatory (MD); handwritten note (HN) 2. Cancer agency letterhead (CL); MD; HN 3. UL; PhD signatory (PhD), HN 4. CL; PhD; HN 5. UL; MD; No HN 6. CL; MD; No HN 7. UL; PhD, No HN 8. CL; PhD, No HN NB: this was a letter requesting doctors to give consent for patients to be contacted and sent questionnaires.	
Outcomes	Response period not specified	
Topic	Health: not specified	
Mode of Administration	Postal	
Notes	—	
***Risk of bias***		
**Item**	**Authors' judgement**	**Support for judgement**
Allocation concealment?	Unclear	B ‐ unclear

**Table:** Smith 1985

***Study characteristics***		
Methods	Random allocation: sequential sampling	
Data	Patients aged 40‐59 years registered with an urban general practice, UK	
Comparisons	1. Questionnaire sent by general practitioner 2. Questionnaire sent by a doctor from the research unit	
Outcomes	Response within 9 weeks	
Topic	Health: Aggression scale, Social desirability scale, Fear survey schedule II, Situations evoking social anxiety scale, Social evaluative anxiety scale	
Mode of Administration	Postal	
Notes	—	
***Risk of bias***		
**Item**	**Authors' judgement**	**Support for judgement**
Allocation concealment?	No	C ‐ inadequate

**Table:** So 2018

***Study characteristics***		
Methods	Random allocation: computer‐generated random sequence	
Data	2000 primary care physicians from members of the Japan Primary Care Association	
Comparisons	Email subject emphasises lottery for an Amazon gift card worth 3000 yen vs. no subject emphasis on lottery Sending the invitation email on Tuesday vs. Friday	
Outcomes	e‐login and e‐completed	
Topic	Health (survey on evidence‐based medicine)	
Mode of Administration	Web survey	
Notes	DOCTOR study, participants were asked to complete a 2‐page online questionnaire about evidence‐based medicine. The questionnaire had 9 and 4 items, respectively, on the first and second pages. Package not described	
***Risk of bias***		
**Item**	**Authors' judgement**	**Support for judgement**
Sequence generation	Yes	Random allocation: computer‐generated random sequence
Allocation concealment?	Yes	Random allocation: computer‐generated random sequence
Blinding of participants and personnel	Unclear	Blinding of personnel not described. Participants were not blinded to intervention.
Blinding of outcome assessment	Yes	No outcomes were assessed (counts of responses only).
Incomplete outcome data	Yes	Exclusions reported
Selective reporting	Yes	e‐login and e‐completed reported in full
Other sources of bias	Yes	No grounds for concern about other sources of bias from reading the study report

**Table:** Solnick 2020

***Study characteristics***		
Methods	Random allocation: method unspecified	
Data	Trainees (n = 8485) from the Emergency Medicine Residents Association (EMRA) email list, US	
Comparisons	Conditional non‐monetary incentive vs. no incentive; larger non‐monetary incentive ($100 giftcard raffle) vs. smaller ($25 giftcard raffle)	
Outcomes	e‐login and e‐completion	
Topic	Non‐health (political beliefs survey sent to Emergency Medicine trainees)	
Mode of Administration	Electronic	
Notes	Questionnaire and package sent not described No incentive vs. conditional non‐monetary ($25 giftcard raffle) vs. conditional non‐monetary ($100 giftcard raffle) vs. conditional non‐monetary ($5 giftcard)	
***Risk of bias***		
**Item**	**Authors' judgement**	**Support for judgement**
Sequence generation	Unclear	Random allocation: method unspecified
Allocation concealment?	Unclear	Random allocation: method unspecified
Blinding of participants and personnel	Unclear	Blinding of personnel not described. Participants were not blinded to intervention.
Blinding of outcome assessment	Yes	No outcomes were assessed (counts of responses only).
Incomplete outcome data	Yes	Exclusions reported; after the first send (4 emails bounced)
Selective reporting	Yes	e‐login and e‐completion reported in full
Other sources of bias	Yes	No grounds for concern about other sources of bias from reading the study report

**Table:** Spry 1989a

***Study characteristics***		
Methods	Random allocation: using a table of random numbers	
Data	Residences listed in the Haines Directory (San Diego, US)	
Comparisons	1. Telephone pre‐notification; lottery entry offer 2. Telephone pre‐notification; no lottery offer 3. Postcard pre‐notification; lottery entry offer 4. Postcard pre‐notification; no lottery offer 5. No pre‐notification; lottery entry offer 6. No pre‐notification; no lottery offer	
Outcomes	Response period not specified	
Topic	Health: health and physical activity habits	
Mode of Administration	Postal	
Notes	Method of allocation ascertained through contact with author. Randomisation not concealed; mainly males	
***Risk of bias***		
**Item**	**Authors' judgement**	**Support for judgement**
Allocation concealment?	No	C ‐ inadequate

**Table:** Spry 1989b

***Study characteristics***		
Methods	Random allocation: using a table of random numbers	
Data	Residences listed in the Haines Directory (San Diego, US)	
Comparisons	1. Short questionnaire; lottery 2. Short questionnaire; no lottery 3. Long questionnaire; lottery 4. Long questionnaire; no lottery	
Outcomes	Response period not specified	
Topic	Health: health and physical activity habits	
Mode of Administration	Postal	
Notes	Method of allocation ascertained through contact with author. Randomisation not concealed; mainly males	
***Risk of bias***		
**Item**	**Authors' judgement**	**Support for judgement**
Allocation concealment?	No	C ‐ inadequate

**Table:** Spry 1989c

***Study characteristics***		
Methods	Random allocation: using a table of random numbers	
Data	Residences listed in the Haines Directory who had not responded to a questionnaire (San Diego, US)	
Comparisons	1. Promise of $5 when response 2. Promise of $1 when response 3. $1 bill enclosed 4. No incentive	
Outcomes	Response period not specified	
Topic	Health: health and physical activity habits	
Mode of Administration	Postal	
Notes	Method of allocation ascertained through contact with author. Randomisation not concealed; mainly males	
***Risk of bias***		
**Item**	**Authors' judgement**	**Support for judgement**
Allocation concealment?	No	C ‐ inadequate

**Table:** Stafford 1966

***Study characteristics***		
Methods	Random allocation: method not specified	
Data	University students (Houston, US)	
Comparisons	1. Pre‐notification letter sent 2. Pre‐notification telephone call made 3. No pre‐notification contact	
Outcomes	—	
Topic	Non‐health: collegiate clothing market	
Mode of Administration	Postal	
Notes	—	
***Risk of bias***		
**Item**	**Authors' judgement**	**Support for judgement**
Allocation concealment?	Unclear	B ‐ unclear

**Table:** Stange 2011

***Study characteristics***		
Methods	Random allocation: method unspecified	
Data	All members of the class of 2006 of a small, highly selective liberal arts school, US	
Comparisons	Pen vs. no pen	
Outcomes	First response and response after 3 weeks (one reminder)	
Topic	Non‐health (perception of job/graduate school options)	
Mode of Administration	Postal	
Notes	6 pages; 25 mins completion time. Included multiple measures of cognitive style, career choices, and demographics Pen or no pen with initial survey mailing, the non‐retractable ballpoint pen included the college logo and colours. A stamped, addressed return envelope was included.	
***Risk of bias***		
**Item**	**Authors' judgement**	**Support for judgement**
Sequence generation	Unclear	Random allocation: method unspecified
Allocation concealment?	Unclear	Random allocation: method unspecified
Blinding of participants and personnel	Unclear	Blinding of personnel not described. Participants were not blinded to intervention.
Blinding of outcome assessment	Yes	No outcomes were assessed (counts of responses only).
Incomplete outcome data	No	Exclusions were not reported.
Selective reporting	Yes	First response and response after 3 weeks (one reminder) reported in full
Other sources of bias	Yes	No grounds for concern about other sources of bias from reading the study report

**Table:** Stapulonis 2004

***Study characteristics***		
Methods	Random allocation: computerised random number generation	
Data	Members from the Welfare‐to‐Work evaluation site at Chicago	
Comparisons	1. Conditional $20 check 2. Conditional point‐of‐sale cards worth $20	
Outcomes	Response period not specified	
Topic	Non‐health: employment	
Mode of Administration	Electronic: Computer Assisted Telephone Interview (CATI)	
Notes	—	
***Risk of bias***		
**Item**	**Authors' judgement**	**Support for judgement**
Allocation concealment?	Yes	A ‐ adequate

**Table:** Starr 2015

***Study characteristics***		
Methods	Random allocation: computer‐generated	
Data	Participants of SUSPEND RCT of medical expulsive therapy for ureteric stone disease, willing to provide a mobile phone number or an email address from 24 UK sites	
Comparisons	SMS pre‐notification vs. no pre‐notification Email reminder (with URL to complete the questionnaire via Web) vs. postal reminder	
Outcomes	Responses at 4 weeks	
Topic	Health (patient‐reported outcomes in SUSPEND RCT, renal stones)	
Mode of Administration	Mixed	
Notes	Questionnaire not described. All participants randomly assigned to the intervention arm were sent an SMS text message pre‐notification of the de‐livery of the initial 4‐ and 12‐week questionnaires. Controls received no pre‐notification. Responses at 4 week time point included in this review. Non‐responders to the intervention arm received email with URL for online completion. Non‐responders to control arm received further copy by post. Both reminder types were generated 2 weeks after the initial questionnaire was sent.	
***Risk of bias***		
**Item**	**Authors' judgement**	**Support for judgement**
Sequence generation	Yes	Random allocation: computer‐generated
Allocation concealment?	Yes	Random allocation: computer‐generated
Blinding of participants and personnel	No	Owing to the nature of the intervention, it was not possible to blind the participants or trial office staff to allocation; however, the researchers remained blind.
Blinding of outcome assessment	Yes	No outcomes were assessed (counts of responses only).
Incomplete outcome data	Yes	Exclusions reported for pre‐notification and reminder comparisons
Selective reporting	Yes	Responses at 4 weeks and at 12 weeks reported in full
Other sources of bias	Yes	No grounds for concern about other sources of bias from reading the study report

**Table:** Stem 1984a

***Study characteristics***		
Methods	Random allocation: using random number table	
Data	Students	
Comparisons	1. Randomised response model 2. Direct questions	
Outcomes	Response period not specified	
Topic	Non‐health: cheating behaviours during exams	
Mode of Administration	Postal	
Notes	—

**Table:** Stem 1984b

***Study characteristics***		
Methods	Random allocation: using random number table	
Data	Automobile sales licence holders	
Comparisons	1. Randomised response model 2. Direct questions	
Outcomes	Response period not specified	
Topic	Non‐health: automobile selling practices	
Mode of Administration	Postal	
Notes	—

**Table:** Stevens 1975

***Study characteristics***		
Methods	Random allocation: alternation	
Data	Graduates from a southern university (US)	
Comparisons	1. Pre‐coded questionnaire 2. Questionnaire not pre‐coded	
Outcomes	Response within 4 weeks	
Topic	Non‐health: job hunting experience	
Mode of Administration	Postal	
Notes	—	
***Risk of bias***		
**Item**	**Authors' judgement**	**Support for judgement**
Allocation concealment?	No	C ‐ inadequate

**Table:** Stolzmann 2019

***Study characteristics***		
Methods	Random allocation: method unspecified	
Data	300 veterans randomly selected from those who had received services at a mental health clinic in the Department of Veterans Affairs, US	
Comparisons	Lean (48) vs. medium (85) vs. maximum (127) questionnaire length	
Outcomes	Response after 4 weeks (2 reminders)	
Topic	Health (healthcare quality and satisfaction surveys in veteran populations)	
Mode of Administration	Postal	
Notes	Questionnaires 48‐127 items; lean (48 questions), medium (85 items) and maximum (127 items). Standardised measures of healthcare quality, satisfaction, and outcomes in addition to demographic questions. Survey packet consisted of a cover letter with instructions, the survey, and a pre‐paid envelope for the returned questionnaire. Non‐respondents received up to two follow‐up contacts, mailed at 2‐week intervals.	
***Risk of bias***		
**Item**	**Authors' judgement**	**Support for judgement**
Sequence generation	Unclear	Random allocation: method unspecified
Allocation concealment?	Unclear	Random allocation: method unspecified
Blinding of participants and personnel	Unclear	Blinding of personnel not described. Participants were not blinded to intervention.
Blinding of outcome assessment	Yes	No outcomes were assessed (counts of responses only).
Incomplete outcome data	No	Exclusions not reported
Selective reporting	Yes	Response after 4 weeks (2 reminders) reported in full
Other sources of bias	Yes	No grounds for concern about other sources of bias from reading the study report

**Table:** Streiff 2001

***Study characteristics***		
Methods	Random allocation: alternation	
Data	Randomly selected members of the American Society of Hematology	
Comparisons	1. Business reply envelope 2. Stamped return envelope	
Outcomes	Response within 3 months	
Topic	Health: diagnosis and treatment of polycythaemia Vera	
Mode of Administration	Postal	
Notes	Method of allocation ascertained through contact with author	
***Risk of bias***		
**Item**	**Authors' judgement**	**Support for judgement**
Allocation concealment?	No	C ‐ inadequate

**Table:** Subar 2001

***Study characteristics***		
Methods	Random allocation: method not specified	
Data	900 control participants from 3 centres in the prostate, lung, colorectal and ovarian cancer screening trial	
Comparisons	1. Diet history questionnaire ‐ 36 pages 2. Food frequency questionnaire ‐ 16 pages	
Outcomes	—	
Topic	Health: food frequency questionnaire, diet history questionnaire	
Mode of Administration	Postal	
Notes	Age: 55‐74 years	
***Risk of bias***		
**Item**	**Authors' judgement**	**Support for judgement**
Allocation concealment?	Unclear	B ‐ unclear

**Table:** Sutton 1992

***Study characteristics***		
Methods	Random allocation: method not specified	
Data	Business customers who had taken advantage of an earlier rebate programme	
Comparisons	1. Pre‐notification postcard; prior telephone call 2. Pre‐notification postcard; no prior telephone call 3. No pre‐notification postcard; no prior telephone call 4. No pre‐notification postcard; no prior telephone call	
Outcomes	Response period within 43 days	
Topic	Non‐health: customer reaction to energy rebate programme	
Mode of Administration	Postal	
Notes	Author contacted: reported adequate allocation concealment	
***Risk of bias***		
**Item**	**Authors' judgement**	**Support for judgement**
Allocation concealment?	Yes	A ‐ adequate

**Table:** Suzer‐Gurtekin 2019

***Study characteristics***		
Methods	Random allocation: method unspecified	
Data	A sample of residential addresses selected from the Marketing Systems Group (MSG) US Postal Service Computerized Delivery Sequence File	
Comparisons	(concurrent) Choice between postal or online questionnaire at the first mailing vs. Web‐only at first mailing (web push); prepaid $5 incentive vs. no incentive	
Outcomes	Response after reminders	
Topic	Non‐health (University of Michigan's Surveys of Consumer attitudes and expectations)	
Mode of Administration	Postal and electronic	
Notes	20‐page booklet; large type Advance letter first, then the questionnaire mailing, then a reminder postcard, then a second questionnaire mailing, and a final reminder postcard. In the concurrent web‐mail design, a cover letter in the first questionnaire mailing included a URL. In the web‐intensive design, a separate web survey invitation letter followed the advance letter with a survey URL. The web survey letter also mentioned a paper questionnaire would be sent in a forthcoming mailing.	
***Risk of bias***		
**Item**	**Authors' judgement**	**Support for judgement**
Sequence generation	Unclear	Random allocation: method unspecified
Allocation concealment?	Unclear	Random allocation: method unspecified
Blinding of participants and personnel	Unclear	Blinding of personnel not described. Participants were not blinded to intervention.
Blinding of outcome assessment	Yes	No outcomes were assessed (counts of responses only).
Incomplete outcome data	No	Exclusions were not reported.
Selective reporting	Yes	Response after reminders reported in full
Other sources of bias	Yes	No grounds for concern about other sources of bias from reading the study report

**Table:** Svensson 2012

***Study characteristics***		
Methods	Cluster‐randomisation by company: intervention groups applied wholly to each company of employees	
Data	3876 employees (18‐65 years) at four companies in the Swedish railway sector	
Comparisons	Low (<= 6) vs. medium (7‐9) vs. high (> 9) numbers of reminders	
Outcomes	Final response	
Topic	Health (lifestyle habits, i.e. diet, sleep, physical activity, stress, and smoking)	
Mode of Administration	Web survey	
Notes	Questionnaire not described. 2 email messages, (pre‐notification invitation and URL to questionnaire), sent to all participants Reminders were given in three amounts, categorised as follows: (1) low amount of reminders: <= 6 reminders, (2)medium amount of reminders: 7‐10 reminders, and (3) high amount of reminders: > 10 reminders 3876 employees were emailed a baseline web‐based lifestyle questionnaire. 9 months later, follow‐up questionnaire sent. 4 and 11 email reminders were sent at baseline and follow‐up, respectively. Additional reminders (media articles, flyers, SMS ) also administered. Reminders (emails + additional) were given in low (<= 6 reminders), medium (7‐9 reminders) or high amount (> 9 reminders).	
***Risk of bias***		
**Item**	**Authors' judgement**	**Support for judgement**
Sequence generation	Unclear	Random allocation: method unspecified
Allocation concealment?	Unclear	Random allocation: method unspecified
Blinding of participants and personnel	Unclear	Blinding of personnel not described. Participants were not blinded to intervention.
Blinding of outcome assessment	Yes	No outcomes were assessed (counts of responses only).
Incomplete outcome data	No	Exclusions not reported
Selective reporting	Yes	Final response reported in full
Other sources of bias	No	Randomisation was at company level: the results were not adjusted for clustering.

**Table:** Svoboda 2001

***Study characteristics***		
Methods	Random allocation: central randomisation	
Data	Head injured adults in the CRASH trial (Czech Republic)	
Comparisons	1. 1‐page questionnaire 2. 3‐page questionnaire	
Outcomes	Response within 3 months	
Topic	Health: disability after traumatic brain injury	
Mode of Administration	Postal	
Notes	—	
***Risk of bias***		
**Item**	**Authors' judgement**	**Support for judgement**
Allocation concealment?	Yes	A ‐ adequate

**Table:** Swan 1980

***Study characteristics***		
Methods	Random allocation: method not specified	
Data	Individuals who had not responded to an earlier questionnaire	
Comparisons	1. Follow‐up letter only 2. Follow‐up letter and questionnaire	
Outcomes	Response period not specified	
Topic	Non‐health: perception of educational needs for the real estate profession, sale management practices, business planning, information about respondents' firm	
Mode of Administration	Postal	
Notes	—	
***Risk of bias***		
**Item**	**Authors' judgement**	**Support for judgement**
Allocation concealment?	Unclear	B ‐ unclear

**Table:** Szelényi 2005

***Study characteristics***		
Methods	Random allocation: method unspecified	
Data	250 third‐year undergraduate students at 47 colleges, US	
Comparisons	Monetary incentive vs. no incentive Larger ($5) vs. smaller ($2) monetary incentive	
Outcomes	Response after one reminder	
Topic	Non‐health (development in spirituality ‐ students perspectives on issues of meaning, purpose, and spirituality, plus college experiences)	
Mode of Administration	Postal	
Notes	The College Students' Beliefs and Values Survey. 4 pages, 234 items. Advance notification postcard, followed 2 weeks later by a mailed questionnaire. Two weeks after the first wave of surveys was mailed to students, a subset of students received an email reminder. Subsequently, a second wave of surveys (without the unconditional monetary incentives) was sent to non‐respondents. To explore the impact of differential monetary incentives, institutions were categorised by type (4‐year college versus university), control (public versus private), religious affiliation (Catholic college versus other religiously affiliated college), and selectivity (based on the average composite SAT score of the entering class). Within each category, schools were randomly assigned to one of three incentive groups ($0, $2, or $5). These monetary incentives were included with the initial survey packet.	
***Risk of bias***		
**Item**	**Authors' judgement**	**Support for judgement**
Sequence generation	Unclear	Random allocation: method unspecified
Allocation concealment?	Unclear	Random allocation: method unspecified
Blinding of participants and personnel	Unclear	Blinding of personnel not described. Participants were not blinded to intervention.
Blinding of outcome assessment	Yes	No outcomes were assessed (counts of responses only).
Incomplete outcome data	No	Exclusions were not reported.
Selective reporting	Yes	Response after one reminder reported in full
Other sources of bias	No	Cluster‐randomised trial, with college the unit of randomisation. Possible effects due to clustering were not reported.

**Table:** Szirony 2002

***Study characteristics***		
Methods	Random allocation: using random table of numbers	
Data	Faculty members from the top 100 graduate degree granting institutions in Nursing	
Comparisons	1. Cover letter signed by a graduate student 2. Cover letter signed by a faculty member	
Outcomes	Response period not specified	
Topic	Health: publication, authorship, reporting of research results, funding, demographics	
Mode of Administration	Postal	
Notes	—	
***Risk of bias***		
**Item**	**Authors' judgement**	**Support for judgement**
Allocation concealment?	No	C ‐ inadequate

**Table:** Tai 2018

***Study characteristics***		
Methods	Random allocation: random number generator	
Data	Healthcare workers who volunteered to receive information regarding future studies about influenza, with both email and a postal address, in Toronto, Canada	
Comparisons	Email invitation to web survey vs. postal invitation Shorter vs. longer Open‐ended questions vs. close‐ended questions	
Outcomes	Final response after one reminder	
Topic	Health (adverse events following immunisation with influenza vaccine)	
Mode of Administration	Web survey	
Notes	Shorter contained 24 questions, longer contained 28 questions; open‐ended version contained 5 open‐ended questions (short version), 6 open‐ended questions (long version). Each invitation was personalised (e.g. 'Dear Jane') and contained a URL to the survey, a brief introduction to the study to increase salience. Reminder emails were sent to all non‐respondents 7 days after the initial email while reminder letters were sent 14 days after the initial letter.	
***Risk of bias***		
**Item**	**Authors' judgement**	**Support for judgement**
Sequence generation	Yes	Random allocation: random number generator
Allocation concealment?	Yes	Random allocation: random number generator
Blinding of participants and personnel	Unclear	Blinding of personnel not described. Participants were not blinded to intervention.
Blinding of outcome assessment	Yes	No outcomes were assessed (counts of responses only).
Incomplete outcome data	Yes	Exclusions reported
Selective reporting	Yes	Final response after one reminder reported in full
Other sources of bias	Yes	No grounds for concern about other sources of bias from reading the study report

**Table:** Tamayo‐Sarver 2004

***Study characteristics***		
Methods	Random allocation: using random numbers generated in STATA	
Data	Practicing Physicians with American College of Emergency Physicians membership	
Comparisons	1. $2 bill 2. Lottery to win $250	
Outcomes	Response period not specified	
Topic	Health: diagnosis and treatment plan; practice environment	
Mode of Administration	Postal	
Notes	—	
***Risk of bias***		
**Item**	**Authors' judgement**	**Support for judgement**
Allocation concealment?	Unclear	B ‐ unclear

**Table:** Tambor 1993

***Study characteristics***		
Methods	Random allocation: method not specified	
Data	Physicians (US)	
Comparisons	1. Continuing medical education credits 2. No credits	
Outcomes	Response period not specified	
Topic	Health: genetic knowledge, psychometric scales, demographics	
Mode of Administration	Postal	
Notes	Mainly males	
***Risk of bias***		
**Item**	**Authors' judgement**	**Support for judgement**
Allocation concealment?	Unclear	B ‐ unclear

**Table:** Tariq 2021

***Study characteristics***		
Methods	Random allocation: random number generator in a secure database	
Data	281 non‐responders to patient‐reported outcome survey, one year after orthopaedic surgery, US	
Comparisons	Personalised letter vs. standard letter	
Outcomes	Response 5 weeks after initial letter	
Topic	Health (one year postoperative surgical assessment)	
Mode of Administration	Postal	
Notes	Questionnaire not described. Institutional Review Board‐approved letter that included personalised information on surgery site, date of surgery, surgeon picture, and scanned surgeon's signature, with a prepaid return envelope and the individual PROM form At tertiary multisite healthcare institution, validated PROMs are electronically collected on every elective knee, hip, and shoulder surgery in 9 hospitals and 59 orthopaedic surgeons utilising a prospective longitudinal cohort design.	
***Risk of bias***		
**Item**	**Authors' judgement**	**Support for judgement**
Sequence generation	Yes	Random allocation: random number generator in a secure database
Allocation concealment?	Yes	Random allocation: random number generator in a secure database
Blinding of participants and personnel	Yes	Patients were blinded to which group they were in. Data collection and processing were done at a central processing site.
Blinding of outcome assessment	Yes	No outcomes were assessed (counts of responses only).
Incomplete outcome data	Yes	Exclusions reported
Selective reporting	Yes	Response 5 weeks after initial letter reported in full
Other sources of bias	Yes	No grounds for concern about other sources of bias from reading the study report

**Table:** Taylor 1998

***Study characteristics***		
Methods	Random allocation: stratified random sampling method	
Data	Young people in the Youth Cohort Study 8 sample, England	
Comparisons	1. Preliminary notice letter 2. No preliminary notification	
Outcomes	Response within approximately 2 months	
Topic	Non‐health: attitudes and behaviour while transition from secondary school to labour market/tertiary education system	
Mode of Administration	Postal	
Notes	Mean age: 16.5 years; mainly females	
***Risk of bias***		
**Item**	**Authors' judgement**	**Support for judgement**
Allocation concealment?	Unclear	B ‐ unclear

**Table:** Taylor 2006

***Study characteristics***		
Methods	Random allocation: computerised random number generation	
Data	Participants registered in general practices in Aberdeen	
Comparisons	1. Questionnaire printed in black ink 2. Questionnaire printed in green ink 3. Questionnaire sent in white envelope 4. Questionnaire sent in brown envelope	
Outcomes	Response period within 6 months	
Topic	Health: screening questions for parkinsonism; EuroQol EQ‐5D	
Mode of Administration	Postal	
Notes	—	
***Risk of bias***		
**Item**	**Authors' judgement**	**Support for judgement**
Allocation concealment?	Unclear	B ‐ unclear

**Table:** Taylor 2019

***Study characteristics***		
Methods	Random allocation: method unspecified	
Data	197 individuals aged 6 to 24 years with smartphones recruited via posters and internet postings to participate in a 5‐week study about mood and mobile game habits in return for receiving a $100 Amazon gift card at the end of the study	
Comparisons	Postal outcome diary vs. ePRO (iOS or Android app that displayed a web view with the daily diary form) vs. novel ePRO diary with in‐game rewards (Game‐Motivated ePRO ‐ including in‐game rewards when they completed a daily diary)	
Outcomes	Completion of daily diaries	
Topic	Non‐health (mood, mobile games, and the weather)	
Mode of Administration	Postal/electronic	
Notes	6 daily multiple‐choice questions. The ePRO groups' app was a simple native iOS or Android app that displayed a web view with the daily diary form. The Game‐Motivated ePRO included the exact same web view and form. Therefore, the ePRO and Game‐Motivated ePRO groups had identical daily diaries; however, the Game‐Motivated ePRO participants received an in‐game reward (i.e. a pet) when they completed a daily diary, whereas the ePRO participants were just shown a screen thanking them for completing the diary and reminding them to return the next day. Participants in the Game‐Motivated ePRO group were informed that they did not need to play the game to fill out their daily diary.	
***Risk of bias***		
**Item**	**Authors' judgement**	**Support for judgement**
Sequence generation	Unclear	Random allocation: method unspecified
Allocation concealment?	Unclear	Random allocation: method unspecified
Blinding of participants and personnel	Yes	Participants were blind to which study group they were in until after completing the prestudy survey.
Blinding of outcome assessment	Yes	No outcomes were assessed (counts of responses only).
Incomplete outcome data	Yes	Exclusions were reported.
Selective reporting	Yes	Completion of daily diaries reported in full
Other sources of bias	Yes	No grounds for concern about other sources of bias from reading the study report

**Table:** Teisl 2005

***Study characteristics***		
Methods	Random allocation: using random numbers generated in Excel	
Data	US residents	
Comparisons	1. $1 cash 2. $2 cash 3. Phone card worth $2 4. Phone card worth $5	
Outcomes	Response period not specified	
Topic	Health: general perception of food and food processing, knowledge, and attitudes towards genetically modified foods	
Mode of Administration	Postal	
Notes	Method of allocation ascertained through contact with author	
***Risk of bias***		
**Item**	**Authors' judgement**	**Support for judgement**
Allocation concealment?	Yes	A ‐ adequate

**Table:** Temple‐Smith 1998

***Study characteristics***		
Methods	Random allocation: method not specified	
Data	General practitioners	
Comparisons	1. Pre‐contact by GP researcher 2. Pre‐contact by non‐medical researcher (older woman) 3. Pre‐contact by non‐medical researcher (younger woman) 4. Pre‐contact by non‐medical researcher (younger man)	
Outcomes	Response within 8 weeks	
Topic	Health: kowledge, attitudes, behaviour, and practice (KABP) in relation to sexually transmitted diseases	
Mode of Administration	Postal	
Notes	Age: above 65 years; mainly males	
***Risk of bias***		
**Item**	**Authors' judgement**	**Support for judgement**
Allocation concealment?	Unclear	B ‐ unclear

**Table:** Thistlethwaite 1993

***Study characteristics***		
Methods	Random allocation: method not specified	
Data	People aged 65 years and over from 7 counties in a midwestern state of the USA	
Comparisons	1. No offer of results (NO); altruistic appeal (A); no demographic omission (no D) 2. NO; A; demographic omission (D) 3. NO; egoistic appeal (E); no D 4. NO; E; D 5. Offer of results (O); A; no D 6. O; A; D 7. O; E; no D 8. O; E; D	
Outcomes	Response within 4 weeks	
Topic	Non‐health: characteristics most desired in retirement centre, leisure‐time activities	
Mode of Administration	Postal	
Notes	Method of allocation ascertained through contact with author; age: above 65 years	
***Risk of bias***		
**Item**	**Authors' judgement**	**Support for judgement**
Allocation concealment?	No	C ‐ inadequate

**Table:** Thomson 2004

***Study characteristics***		
Methods	Random allocation: computerised random number generation	
Data	Practising GPs in Lothian, Scotland	
Comparisons	1. Lottery to win 6 bottles of champagne 2. Lottery to win 1 bottle of champagne	
Outcomes	Response period not specified	
Topic	Health: GPs opinions on toenail surgery services offered by podiatrists and surgeons	
Mode of Administration	Postal	
Notes	Method of allocation ascertained through contact with author	
***Risk of bias***		
**Item**	**Authors' judgement**	**Support for judgement**
Allocation concealment?	Yes	A ‐ adequate

**Table:** Tilbrook 2014

***Study characteristics***		
Methods	Random allocation: computer‐generated	
Data	Participants in the ATLAS treatment trial of acupuncture or Alexander training or usual care for the treatment of neck pain, due to receive a 6‐month follow‐up questionnaire, UK	
Comparisons	More personalised (Post‐it note with a handwritten message encouraging return of questionnaire) vs. less (control)	
Outcomes	Response after 3 reminders (42 days)	
Topic	Health (treatment of neck pain)	
Mode of Administration	Postal	
Notes	Northwick Park Neck Pain and associated Disability Questionnaire (NPQ) (1 page ‐ 10 questions). All participants sent a paper, postal, questionnaire. Those providing mobile numbers were sent a 7‐day SMS reminder. Postal reminder after 14 days, and again after a further 10 days. Final telephone follow‐up 7 days later. The intervention was: a yellow 3" ≤ square Post‐it note with handwritten text, in black ink with the wording 'Please complete and return to us as soon as possible. Thank you. [Signed with first name].' The intervention was only used on the first questionnaire sent out and was not used on any reminder questionnaires.	
***Risk of bias***		
**Item**	**Authors' judgement**	**Support for judgement**
Sequence generation	Yes	Random allocation: computer‐generated
Allocation concealment?	Yes	Random allocation: computer‐generated
Blinding of participants and personnel	Yes	The response rate was determined by York Trials Unit data clerks who were not aware to which group the participants belonged.
Blinding of outcome assessment	Yes	No outcomes were assessed (counts of responses only).
Incomplete outcome data	Yes	Exclusions reported
Selective reporting	Yes	Response after 3 reminders (42 days) reported in full
Other sources of bias	Yes	No grounds for concern about other sources of bias from reading the study report

**Table:** Tjerbo 2005

***Study characteristics***		
Methods	Random allocation: method not specified	
Data	Medical practitioners in Norway	
Comparisons	1. Unconditional scratch lottery 2. Conditional lottery to win a holiday trip worth 8000 Norwegian Kroner 3. Control	
Outcomes	—	
Topic	Health: relationship between primary care and secondary care	
Mode of Administration	Postal	
Notes	Language of publication Norwegian	
***Risk of bias***		
**Item**	**Authors' judgement**	**Support for judgement**
Allocation concealment?	Unclear	B ‐ unclear

**Table:** Todd 2015

***Study characteristics***		
Methods	Random allocation: computer‐generated random number generation	
Data	A sample of 2048 Australian women who had recently given birth at seven maternity units in New South Wales, Australia	
Comparisons	Choice to opt‐out from study vs. none	
Outcomes	Final response after 2 reminders	
Topic	Health (expectations and recent experiences of maternity care)	
Mode of Administration	Postal	
Notes	123 questions; 20‐30 minutes completion time. Personalised pre‐notification letter, sponsored by an academic organisation, assurances of data confidentiality and anonymity, plus an SAE. 2 reminders	
***Risk of bias***		
**Item**	**Authors' judgement**	**Support for judgement**
Sequence generation	Yes	Random allocation: computer‐generated random number generation
Allocation concealment?	Yes	Random allocation: computer‐generated random number generation
Blinding of participants and personnel	Yes	A person not otherwise involved in the study co‐ordinated the mailout of pre‐notification letters, the survey packages and the reminder letters. This person had access to women's names and addresses but not their health or survey information. All completed surveys were received by the researchers, containing each woman's unique study number but no other identifying details.
Blinding of outcome assessment	Yes	No outcomes were assessed (counts of responses only).
Incomplete outcome data	Yes	Exclusions reported
Selective reporting	Yes	Final response after 2 reminders reported in full
Other sources of bias	Yes	No grounds for concern about other sources of bias from reading the study report

**Table:** Trussell 2004a

***Study characteristics***		
Methods	Random allocation: method not specified	
Data	Households in the designated market area in the US who agreed to participate in the mail survey during the screening telephone survey	
Comparisons	1. No incentives 2. One $1 bill 3. Two $1 bills 4. Three $1 bills 5. Four $1 bills 6. Five $1 bills 7. Six $1 bills 8. Seven $1 bills 9. Eight $1 bills 10. Ten $1 bills	
Outcomes	Response period not specified	
Topic	Non‐health: television viewing	
Mode of Administration	Postal	
Notes	Larger incentive: from one $1 bill to five $1 bills; smaller incentive: from six one $1 bills to ten $1 bills	
***Risk of bias***		
**Item**	**Authors' judgement**	**Support for judgement**
Allocation concealment?	Yes	A ‐ adequate

**Table:** Trussell 2004b

***Study characteristics***		
Methods	Random allocation: method not specified	
Data	Households in the designated market area in the US who we were unable to contact during the screening telephone survey	
Comparisons	1. No incentives 2. One $1 bill 3. Two $1 bills 4. Three $1 bills 5. Four $1 bills 6. Five $1 bills 7. Six $1 bills 8. Seven $1 bills 9. Eight $1 bills 10. Ten $1 bills	
Outcomes	Response period not specified	
Topic	Non‐health: television viewing	
Mode of Administration	Postal	
Notes	Larger incentive: from one $1 bill to five $1 bills; smaller incentive: from six one $1 bills to ten $1 bills	
***Risk of bias***		
**Item**	**Authors' judgement**	**Support for judgement**
Allocation concealment?	Yes	A ‐ adequate

**Table:** Trussell 2004c

***Study characteristics***		
Methods	Random allocation: method not specified	
Data	Households in the designated market area in the US who refused to participate in the mail survey during the screening telephone survey	
Comparisons	1. No incentives 2. One $1 bill 3. Two $1 bills 4. Three $1 bills 5. Four $1 bills 6. Five $1 bills 7. Six $1 bills 8. Seven $1 bills 9. Eight $1 bills 10. Ten $1 bills	
Outcomes	Response period not specified	
Topic	Non‐health: television viewing	
Mode of Administration	Postal	
Notes	Larger incentive: from one $1 bill to five $1 bills; smaller incentive: from six one $1 bills to ten $1 bills	
***Risk of bias***		
**Item**	**Authors' judgement**	**Support for judgement**
Allocation concealment?	Yes	A ‐ adequate

**Table:** Tullar 1979

***Study characteristics***		
Methods	Random allocation: method not specified	
Data	Large manufacturing firms	
Comparisons	1. No follow‐up; no incentive 2. No follow‐up; 10 cents incentive 3. Follow‐up; no incentive 4. No follow‐up; 10 cents incentive	
Outcomes	Response within 8 weeks	
Topic	Non‐health: time for development of new product	
Mode of Administration	Postal	
Notes	—	
***Risk of bias***		
**Item**	**Authors' judgement**	**Support for judgement**
Allocation concealment?	Unclear	B ‐ unclear

**Table:** Tullar 2004

***Study characteristics***		
Methods	Random allocation: computerised random number generation	
Data	Medicare recipients who underwent total hip replacement in 1995	
Comparisons	1. Handwritten addresses on the envelope of all outgoing mail 2. Computer‐printed addresses on the envelope of all outgoing mail 3. Hand‐stamped envelopes 4. Institutionally metered postage	
Outcomes	Response period not specified	
Topic	Health: pain, functional status, satisfaction, complications, general health	
Mode of Administration	Postal	
Notes	Method of allocation ascertained through contact with author	
***Risk of bias***		
**Item**	**Authors' judgement**	**Support for judgement**
Allocation concealment?	Yes	A ‐ adequate

**Table:** Turnbull 2015

***Study characteristics***		
Methods	Random allocation: method unspecified	
Data	1850 eligible US intensive care physicians from an existing database, US	
Comparisons	Conditional non‐monetary incentive (Amazon gift card up to $50) vs. no incentive	
Outcomes	e‐login and e‐completion	
Topic	Health (communication about life support with families of critically ill patients)	
Mode of Administration	Web	
Notes	Web survey; 5‐minute completion time. Invitation by email containing a unique link to the survey. All invitations included the survey topic, number of questions, expected time required to complete the survey (5 minutes), IRB approval, study confidentiality, number of follow‐up/reminder emails for non‐responders, planned date for study closing Invitations for intensivists randomised to receive an incentive to participate also included the following text: "In appreciation for your participation, the first 100 respondents to complete the survey will be offered an Amazon.com gift code at the end of the survey. The code can be redeemed immediately for any amount up to $50. In selecting the compensation amount, please consider that this is a PhD thesis project being funded by a limited student budget."	
***Risk of bias***		
**Item**	**Authors' judgement**	**Support for judgement**
Sequence generation	Unclear	Random allocation: method unspecified
Allocation concealment?	Unclear	Random allocation: method unspecified
Blinding of participants and personnel	Unclear	Blinding of personnel not described. Participants were not blinded to intervention.
Blinding of outcome assessment	Yes	No outcomes were assessed (counts of responses only).
Incomplete outcome data	Yes	Exclusions reported
Selective reporting	Yes	e‐login and e‐completion reported in full
Other sources of bias	Yes	No grounds for concern about other sources of bias from reading the study report

**Table:** Tuten 2004

***Study characteristics***		
Methods	Random allocation: method not specified	
Data	Unemployed Croatians	
Comparisons	1. No incentives 2. Offer of study results 3. Lottery of 1000 Kuna with immediate notification of the results 4. Lottery of 1000 Kuna with delayed (after 1 month) notification of the results	
Outcomes	Response period not specified	
Topic	Health: psychosocial consequences of unemployment	
Mode of Administration	Electronic: online survey	
Notes	—	
***Risk of bias***		
**Item**	**Authors' judgement**	**Support for judgement**
Allocation concealment?	Unclear	B ‐ unclear

**Table:** Ulrich 2005

***Study characteristics***		
Methods	Random allocation: computerised random number generation	
Data	Nurse practitioners and physician assistants practising in primary care in the US	
Comparisons	1. No incentive 2. Unconditional $5 prepaid token incentive 3. Conditional lottery to win one of ten $100 prize draws	
Outcomes	Response period not specified	
Topic	Health: ethical concerns in the course of practice	
Mode of Administration	Postal	
Notes	—	
***Risk of bias***		
**Item**	**Authors' judgement**	**Support for judgement**
Allocation concealment?	Unclear	B ‐ unclear

**Table:** Urban 1993

***Study characteristics***		
Methods	Random allocation: method not specified	
Data	Physicians providing primary care	
Comparisons	1. Return envelope with first‐class stamp 2. Business‐reply return envelope	
Outcomes	Response within 6 weeks	
Topic	Health: attitudes, beliefs, and practices regarding regular breast cancer screening	
Mode of Administration	Postal	
Notes	Age: 50‐75 years; mainly females	
***Risk of bias***		
**Item**	**Authors' judgement**	**Support for judgement**
Allocation concealment?	Unclear	B ‐ unclear

**Table:** van den Berg 2011

***Study characteristics***		
Methods	Quasi‐random allocation: sorting survivors alphabetically based on their address (first half was allocated to the mixed invitation group and the second half to the Web‐only invitation group)	
Data	277 female survivors of childhood cancer	
Comparisons	Electronic vs choice (postal/electronic)	
Outcomes	Response after 6 weeks (2 reminders)	
Topic	Health (reproductive function, ovarian reserve, and risk of premature menopause in female childhood cancer survivors)	
Mode of Administration	Mixed	
Notes	An adaptation of a well‐tested questionnaire used by the Department of Epidemiology of the Netherlands Cancer Institute in a large‐scale Dutch cohort study on long‐term effects of ovarian stimulation for in vitro fertilisation. Participants in the mixed invitation group received an invitation that contained a paper‐based questionnaire together with an instruction sheet for the Web‐based questionnaire. This instruction sheet contained a personalised username, the name of the website, and a login code allowing them to log in to a secured part of the website and fill out the questionnaire. The envelope containing the study information package was sealed and put in another envelope together with a cover letter, signed by the head of the relevant paediatric oncology department, in which the study was explained very briefly. The paper‐ and Web‐based version of the questionnaire were identical in terms of the questions asked, their wording, and their order of presentation. In the Web‐based version, however, questions not relevant to the participant were automatically skipped. The Web‐based version of the questionnaire was accessible to participants through a website which was specially designed for the nationwide study. Participants in the Web‐only group received the instruction sheet containing the name of the website and the login details alone. If the questionnaire was not returned within 3 weeks, a reminder was sent by postal mail. For participants in the mixed invitation group, this reminder consisted of a letter in which the relevance of the study was again stressed and in which the individual was asked to respond. For participants in the Web‐only invitation group, a paper‐based version of the questionnaire was added to this reminder letter. When, after 3 weeks, no response was still received, patients in both groups were contacted by telephone and were asked to respond.	
***Risk of bias***		
**Item**	**Authors' judgement**	**Support for judgement**
Sequence generation	No	Quasi‐random allocation: alphabetical sorting
Allocation concealment?	No	Quasi‐random allocation: alphabetical sorting
Blinding of participants and personnel	Unclear	Blinding of personnel not described. Participants were not blinded to intervention.
Blinding of outcome assessment	Yes	No outcomes were assessed (counts of responses only).
Incomplete outcome data	Yes	Exclusions were reported.
Selective reporting	Yes	Response after 6 weeks (2 reminders) reported in full
Other sources of bias	Yes	No grounds for concern about other sources of bias from reading the study report

**Table:** van der Mark 2012

***Study characteristics***		
Methods	Random allocation: computer random number generator	
Data	Parents of children at risk of developing asthma, participating in the ARCADE cohort, The Netherlands	
Comparisons	Conditional non‐monetary incentive (12.50 gift voucher) vs. no incentive Conditional non‐monetary incentive (daytrip to a popular amusement park) vs. no incentive	
Outcomes	Response after one reminder	
Topic	Health (quality of life and airway problems for children at high risk of asthma)	
Mode of Administration	Postal	
Notes	130 multiple‐choice questions. Postal personalised letter and questionnaire consisting of a bright‐coloured cover and a stamped return envelope. The letter had a logo of the academic hospital and the specific ARCADE study logo, both in red ink and signed by the researcher. Postal reminders to non‐responders after 2 weeks. The follow‐up of the trial nested in the ARCADE study lasted for 2 years, maximum of 4 questionnaires (every 6 months; T0, T6, T12 and T18). T0 and T12: 130 multiple choice questions; T6 and T18: 38 multiple choice questions. Comparison of voucher extracted only (same control)	
***Risk of bias***		
**Item**	**Authors' judgement**	**Support for judgement**
Sequence generation	Yes	Random allocation: computer random number generator
Allocation concealment?	Yes	Random allocation: computer random number generator
Blinding of participants and personnel	No	Research staff was not blinded to the randomisation during the follow‐up period. Neither participants nor personnel were blinded.
Blinding of outcome assessment	Yes	No outcomes were assessed (counts of responses only).
Incomplete outcome data	Yes	Exclusions (n = 0) reported
Selective reporting	Yes	Response after one reminder reported in full
Other sources of bias	Yes	No grounds for concern about other sources of bias from reading the study report

**Table:** Van Mol 2017

***Study characteristics***		
Methods	Random allocation: method unspecified	
Data	Full population of 15,651 higher education students of the University of Antwerp, Belgium	
Comparisons	Standard reminder email vs. standard reminder plus median response time to complete vs. standard reminder plus number of completed surveys vs. standard reminder plus median time and number completed	
Outcomes	Response after experimental reminder	
Topic	Non‐health (students' attitudes and opinions about internationalisation initiatives at the university)	
Mode of Administration	Web survey	
Notes	Questionnaire not described. Available in Dutch and English. Students initially received an email with a link to the questionnaire.	
***Risk of bias***		
**Item**	**Authors' judgement**	**Support for judgement**
Sequence generation	Unclear	Random allocation: method unspecified
Allocation concealment?	Unclear	Random allocation: method unspecified
Blinding of participants and personnel	Unclear	Blinding of personnel not described. Participants were not blinded to intervention.
Blinding of outcome assessment	Yes	No outcomes were assessed (counts of responses only).
Incomplete outcome data	No	Exclusions not reported
Selective reporting	Yes	Response after experimental reminder reported in full
Other sources of bias	Yes	No grounds for concern about other sources of bias from reading the study report

**Table:** VanGeest 2001

***Study characteristics***		
Methods	Random allocation: method not specified	
Data	Physicians randomly selected from the American Medical Association's master file of all physicians practising in the US	
Comparisons	1. $5 cash incentive 2. $10 cash incentive 3. $20 cash incentive	
Outcomes	Response period not specified	
Topic	Health: attitudes and responses in relation to utilisation and review pressure	
Mode of Administration	Postal	
Notes	Mainly males	
***Risk of bias***		
**Item**	**Authors' judgement**	**Support for judgement**
Allocation concealment?	Unclear	B ‐ unclear

**Table:** Veen 2015

***Study characteristics***		
Methods	Random allocation: method unspecified	
Data	University students, Germany	
Comparisons	Pre‐contact (mail pre‐notification to web survey) vs. none Non‐monetary incentive (EU 5 voucher ‐ conditional/unconditional) vs. no incentive Unconditional monetary incentive (EU 5 bill) (group 5) vs. no incentive (group 2)	
Outcomes	First page only (e‐login) and all pages (e‐submissions; both after 2 reminders)	
Topic	Non‐health (study conditions, self‐rated competence, and student fraud)	
Mode of Administration	Web survey	
Notes	Questionnaire not described. The pre‐notification contained general information about the study. Students were informed that their university would be emailing a link to the survey shortly. To make the study appear more legitimate and to underscore its official character, logos of the university and the funding agency were placed in the footer. Non‐respondents and dropouts were sent up to two email reminders.	
***Risk of bias***		
**Item**	**Authors' judgement**	**Support for judgement**
Sequence generation	Unclear	Random allocation: method unspecified
Allocation concealment?	Unclear	Random allocation: method unspecified
Blinding of participants and personnel	Unclear	Blinding of personnel not described. Participants were not blinded to intervention.
Blinding of outcome assessment	Yes	No outcome assessment used (counts of responses only)
Incomplete outcome data	No	Exclusions not reported
Selective reporting	Yes	Outcomes reported in full
Other sources of bias	Yes	No grounds for concern about other sources of bias from reading the study report

**Table:** Veiga 1974

***Study characteristics***		
Methods	Random allocation: method not specified	
Data	Randomly selected managers	
Comparisons	1. Stamped return envelope 2. Business‐reply return envelope 3. Internal mail return	
Outcomes	Response within 4 weeks	
Topic	Not specified	
Mode of Administration	Postal	
Notes	Method of allocation ascertained through contact with author	
***Risk of bias***		
**Item**	**Authors' judgement**	**Support for judgement**
Allocation concealment?	Unclear	B ‐ unclear

**Table:** Viera 2012

***Study characteristics***		
Methods	Random allocation: method unspecified	
Data	8477 physicians randomly drawn from mailing lists of the American Academy of Family Physicians and the American College of Physicians	
Comparisons	Conditional non‐monetary incentive (2 hours of Continuing Medical Education (CME) credit) vs. no incentive	
Outcomes	Response after 2 reminders	
Topic	Health (physicians' opinions on "new ideas in cardiovascular disease prevention.")	
Mode of Administration	Web survey	
Notes	40 items, approximately 10 mins to complete. Physicians were mailed a personalised letter describing the survey and a URL. Informed they would receive a chance to be entered into a draw for one of two $500 Visa gift cards if they completed the survey. Reminder letters to non‐responders sent at 2 and 4 weeks	
***Risk of bias***		
**Item**	**Authors' judgement**	**Support for judgement**
Sequence generation	Unclear	Random allocation: method unspecified
Allocation concealment?	Unclear	Random allocation: method unspecified
Blinding of participants and personnel	Unclear	Blinding of personnel not described. Participants were not blinded to intervention.
Blinding of outcome assessment	Yes	No outcomes were assessed (counts of responses only).
Incomplete outcome data	Yes	Exclusions reported
Selective reporting	Yes	Response after 2 reminders reported in full
Other sources of bias	Yes	No grounds for concern about other sources of bias from reading the study report

**Table:** Virtanen 2007a

***Study characteristics***		
Methods	Random allocation: using split‐panel design	
Data	Working‐age population living in rural areas in Finland	
Comparisons	1. SMS reminder 2. Traditional postcard reminder	
Outcomes	Response period within 28 days	
Topic	Non‐health: Information and Computer Technology (ICT) usage	
Mode of Administration	Postal	
Notes	—	
***Risk of bias***		
**Item**	**Authors' judgement**	**Support for judgement**
Allocation concealment?	Unclear	B ‐ unclear

**Table:** Virtanen 2007b

***Study characteristics***		
Methods	Random allocation: using split‐panel design	
Data	Welfare and health professionals in Finland	
Comparisons	1. SMS reminder 2. Traditional postcard reminder	
Outcomes	Response period within 28 days	
Topic	Health: working and welfare conditions of health and social care workers	
Mode of Administration	Postal	
Notes	—	
***Risk of bias***		
**Item**	**Authors' judgement**	**Support for judgement**
Allocation concealment?	Unclear	B ‐ unclear

**Table:** Virtanen 2007c

***Study characteristics***		
Methods	Random allocation: using split‐panel design	
Data	Members of trade union in Finland	
Comparisons	1. SMS reminder 2. Traditional postcard reminder	
Outcomes	Response period within 28 days	
Topic	Non‐health: employment	
Mode of Administration	Postal	
Notes	—	
***Risk of bias***		
**Item**	**Authors' judgement**	**Support for judgement**
Allocation concealment?	Unclear	B ‐ unclear

**Table:** Vocino 1977

***Study characteristics***		
Methods	Random allocation: method not specified	
Data	Members of the American Society for Public Administration	
Comparisons	1. Metered envelope 2. Commemorative stamp 3. Deadline 4. No deadline 5. Cover letter by well‐known person in the discipline 6. Cover letter by unknown person in the discipline Factorial design	
Outcomes	Response period not specified	
Topic	Not specified	
Mode of Administration	Postal	
Notes	—	
***Risk of bias***		
**Item**	**Authors' judgement**	**Support for judgement**
Allocation concealment?	Unclear	B ‐ unclear

**Table:** Vogel 1992

***Study characteristics***		
Methods	Random allocation: method not specified	
Data	Individuals treated at an alcohol and drug treatment centre (Norway)	
Comparisons	1. Short questionnaire; lottery ($70) incentive if respond 2. Short questionnaire; no lottery incentive 3. Long questionnaire; lottery ($70) incentive if respond 4. Long questionnaire; no lottery incentive Follow‐up after 7 months	
Outcomes	Response period not specified	
Topic	Health: post‐discharge alcohol use, health status	
Mode of Administration	Postal	
Notes	Mean age: 42.4 years; mainly males	
***Risk of bias***		
**Item**	**Authors' judgement**	**Support for judgement**
Allocation concealment?	Unclear	B ‐ unclear

**Table:** VonRiesen 1979

***Study characteristics***		
Methods	Random allocation: method not specified	
Data	Veterinarians (Texas, US)	
Comparisons	1. Postcard reminders 8 days after initial mailing 2. Second copy of questionnaire, with cover letter and business reply envelope, 8 days after initial mailing 3. No follow‐up	
Outcomes	Response period not specified	
Topic	Health: supplier configuration, reasons for patronage, dollar amounts of annual purchases	
Mode of Administration	Postal	
Notes	—	
***Risk of bias***		
**Item**	**Authors' judgement**	**Support for judgement**
Allocation concealment?	Unclear	B ‐ unclear

**Table:** Waisanen 1954

***Study characteristics***		
Methods	Random allocation: alternation	
Data	Equal numbers of families owning and not owning television sets	
Comparisons	1. Telephone pre‐contact 2. No telephone pre‐contact	
Outcomes	Response within 10 days	
Topic	Non‐health: self‐rating of personal possession, occupation, television, income, education	
Mode of Administration	Postal	
Notes	—	
***Risk of bias***		
**Item**	**Authors' judgement**	**Support for judgement**
Allocation concealment?	No	C ‐ inadequate

**Table:** Wakabayashi 2012

***Study characteristics***		
Methods	Random allocation: method unspecified	
Data	6938 female nurses and/or midwives, who were aged at least 30 years, in the fifth‐wave follow‐up survey of the Japan Nurses' Health Study	
Comparisons	Stamped return envelopes vs. business‐reply return envelopes Study newsletter with first mailing vs. study newsletter with reminder	
Outcomes	First response and final response after 12 weeks (one reminder)	
Topic	Health (effects of lifestyle and healthcare practices on women's health)	
Mode of Administration	Postal	
Notes	Questionnaire not described. A thank‐you and reminder letter was mailed out to all respondents (regardless of whether they had already returned their questionnaires) after 6 weeks.	
***Risk of bias***		
**Item**	**Authors' judgement**	**Support for judgement**
Sequence generation	Unclear	Random allocation: method unspecified
Allocation concealment?	Unclear	Random allocation: method unspecified
Blinding of participants and personnel	Unclear	Blinding of personnel not described. Participants were not blinded to intervention.
Blinding of outcome assessment	Yes	No outcomes were assessed (counts of responses only).
Incomplete outcome data	No	Exclusions not reported
Selective reporting	Yes	First response and final response after 12 weeks (one reminder) reported in full
Other sources of bias	Yes	No grounds for concern about other sources of bias from reading the study report

**Table:** Walker 1997

***Study characteristics***		
Methods	Random allocation: method not specified	
Data	General population controls in a leg ulcer study aged 40‐99 years, randomly selected from the electoral roll, Auckland, New Zealand	
Comparisons	1. Glossy brochure enclosed 2. No glossy brochure	
Outcomes	Response by post	
Topic	Health: SF‐36, HRQoL (leg ulcers)	
Mode of Administration	Postal	
Notes	Age: 40‐90 years	
***Risk of bias***		
**Item**	**Authors' judgement**	**Support for judgement**
Allocation concealment?	Unclear	B ‐ unclear

**Table:** Waltemyer 2005

***Study characteristics***		
Methods	Random allocation: method not specified	
Data	National Collegiate Athletic Association (NCAA) Division I and III assistant softball coaches	
Comparisons	1. Signed cover letter 2. Unsigned cover letter 3. White questionnaire 4. Yellow questionnaire	
Outcomes	Response period not specified	
Topic	Not specified	
Mode of Administration	Postal	
Notes	—	
***Risk of bias***		
**Item**	**Authors' judgement**	**Support for judgement**
Allocation concealment?	Unclear	B ‐ unclear

**Table:** Wan 2012

***Study characteristics***		
Methods	Random allocation: method unspecified	
Data	1000 dermatologists from American Academy of Dermatology members who self‐identified as treating psoriasis and from active National Psoriasis Foundation members	
Comparisons	Higher unconditional monetary incentive ($10) vs. lower unconditional monetary incentive ($5) vs. no incentive	
Outcomes	Response after one reminder	
Topic	Health (preferences for psoriasis treatment)	
Mode of Administration	Postal	
Notes	Questionnaire not described. Each participant to receive an initial questionnaire packet with either $5 or $10 (with a note offering this token of appreciation) or no cash. Postcard reminders and duplicate surveys sent to non‐respondents after the initial mailing	
***Risk of bias***		
**Item**	**Authors' judgement**	**Support for judgement**
Sequence generation	Unclear	Random allocation: method unspecified
Allocation concealment?	Unclear	Random allocation: method unspecified
Blinding of participants and personnel	Unclear	Blinding of personnel not described. Participants were not blinded to intervention.
Blinding of outcome assessment	Yes	No outcomes were assessed (counts of responses only).
Incomplete outcome data	Yes	Exclusions reported
Selective reporting	Yes	Response after one reminder reported in full
Other sources of bias	Yes	No grounds for concern about other sources of bias from reading the study report

**Table:** Ward 1996

***Study characteristics***		
Methods	Random allocation: method not specified	
Data	Patients from a metropolitan general practice (Sydney, Australia)	
Comparisons	1. $1 'scratchy' incentive with questionnaire 2. No incentive Follow‐up sent at 21 and 30 days	
Outcomes	Response within 30 days	
Topic	Health: SF‐36, patient satisfaction, risk factors, chronic diseases	
Mode of Administration	Postal	
Notes	—	
***Risk of bias***		
**Item**	**Authors' judgement**	**Support for judgement**
Allocation concealment?	Unclear	B ‐ unclear

**Table:** Ward 1998

***Study characteristics***		
Methods	Random allocation: method not specified	
Data	Registered medical practitioners who had at least 1500 consultations per year	
Comparisons	1. Exhaustive pre‐contact by telephone (continued until spoken to GP) 2. Gold pen incentive; University of NSW logo attached to questionnaire 3. Pre‐contact letter with University of NSW crests Follow‐up letter sent after 16 days to non‐respondents Second questionnaire sent after 23 days Telephone prompt from a non‐medical research assistant after 39 days	
Outcomes	—	
Topic	Health: cancer screening, personal and family history of cancer, sociodemographics	
Mode of Administration	Postal	
Notes	Mainly males	
***Risk of bias***		
**Item**	**Authors' judgement**	**Support for judgement**
Allocation concealment?	Unclear	B ‐ unclear

**Table:** Warriner 1996

***Study characteristics***		
Methods	Random allocation: method not specified	
Data	Households listed in the Grand River Watershed region of southwestern Ontario, Canada	
Comparisons	1. Monetary incentive 2. No monetary incentive 3. Offer to make a charitable donation or lottery 4. No offer Factorial design	
Outcomes	Response period not specified	
Topic	Non‐health: environmental issues	
Mode of Administration	Postal	
Notes	Author contacted: allocation was not concealed	
***Risk of bias***		
**Item**	**Authors' judgement**	**Support for judgement**
Allocation concealment?	No	C ‐ inadequate

**Table:** Warwick 2019

***Study characteristics***		
Methods	Random allocation: computer‐generated blocked randomisation scheme	
Data	3,000 adults that had undergone an orthopaedic procedure within 2 years, and had an email address, at one institution in US	
Comparisons	No incentive vs. non‐monetary incentive (USD 5 donation to another patient in financial need, or to a research programme) vs. stress how survey response helps to improve care quality	
Outcomes	Response after 4 weeks (2 reminders)	
Topic	Health (postoperative patient‐reported outcome measures in an orthopaedic population)	
Mode of Administration	Web survey	
Notes	Surveys included questions on patient demographics as well as Patient‐Reported Outcomes Measurement Information System computer adaptive tests assessing physical function, pain interference, pain intensity, and depression. 5‐10 mins completion time. All patients received an email invitation with the same PROM survey link. Reminders containing the same text as in the original email were sent to non‐respondents at 1 and 2 weeks. Data collection ended 4 weeks after the initial email. (1) control: no incentive (n = 750); (2) patient donation: offer of a USD 5 donation to provide medical supplies to a paediatric orthopaedic patient (n = 751); (3) research donation: offer of a USD 5 donation to a procedure‐specific research programme (n = 749); or (4) explanation: explanation that response supports quality improvement (n = 750)	
***Risk of bias***		
**Item**	**Authors' judgement**	**Support for judgement**
Sequence generation	Yes	Random allocation: computer‐generated blocked randomisation scheme
Allocation concealment?	Yes	Random allocation: computer‐generated blocked randomisation scheme
Blinding of participants and personnel	Yes	Individuals were blinded to their participation in this study and were unaware that patients in the other experimental groups received different email messages.
Blinding of outcome assessment	Yes	No outcomes were assessed (counts of responses only).
Incomplete outcome data	Yes	Exclusions reported
Selective reporting	Yes	Response after 4 weeks (2 reminders) reported in full
Other sources of bias	Yes	No grounds for concern about other sources of bias from reading the study report

**Table:** Weaver 2019

***Study characteristics***		
Methods	Random allocation: method unspecified	
Data	1224 physicians from the Minnesota Board of Medical Practice with both a postal and email address listed, US	
Comparisons	Postal with electronic follow‐up vs. electronic with postal follow‐up vs. postal only vs. electronic only	
Outcomes	Response after 3 reminders	
Topic	Health (factors that influence physicians' willingness to disclose medical errors and adverse events to patients and their families)	
Mode of Administration	Postal and electronic	
Notes	Questionnaire not described. Mail contacts included a cover letter that was printed on the University of Minnesota, Twin Cities letterhead. It was accompanied by a copy of their assigned survey booklet and a business‐reply envelope. For all web surveys, the body of the email included information that was similar to what was included in the mailed cover letters. The emails also included a URL to the survey. The mode experiment was embedded in the Medical Error Disclosure Survey (MEDS) and the Adverse Event Disclosure Survey (AEDS), which was fielded from November 2017 to February 2018.	
***Risk of bias***		
**Item**	**Authors' judgement**	**Support for judgement**
Sequence generation	Unclear	Random allocation: method unspecified
Allocation concealment?	Unclear	Random allocation: method unspecified
Blinding of participants and personnel	Unclear	Blinding of personnel not described. Participants were not blinded to intervention.
Blinding of outcome assessment	Yes	No outcomes were assessed (counts of responses only).
Incomplete outcome data	No	Exclusions not reported
Selective reporting	Yes	Response after 3 reminders reported in full
Other sources of bias	Yes	No grounds for concern about other sources of bias from reading the study report

**Table:** Webborn 2022

***Study characteristics***		
Methods	Random allocation: households randomly ordered in a table and a column repeating the numbers 1 to 12 sequentially was added to assign the test cells.	
Data	Smart Energy Research Laboratory (SERL) survey participants representative of seven regions in England and Wales	
Comparisons	Unconditional non‐monetary incentive (room thermometer) vs. conditional non‐monetary incentive (£5 Love2Shop voucher) vs. no incentive Push‐to‐web (enouraging Web response) vs. no push‐to‐Web Appeal stresses benefit to society (letter content version 1) vs. other (letter content version 2)	
Outcomes	Final response after 3 reminders	
Topic	Non‐health (household energy use)	
Mode of Administration	Web	
Notes	10 minutes to complete. All mailings were sent in an A5 envelope with three project partner logos above the tagline 'University research for public good'. The clear panel on the envelope showed the address and 'Dear Sir/Madam' at the start of the invitation (or reminder) letter. The letter includes colour photos of the two professors conducting the study with scans of their signatures. Group 1's first mailing contained headlines relating to family/ future generations with the reasons for taking part followed by the 'call to action'. In contrast, group 2 received messaging about the UK becoming more energy efficient and the potential for energy bill savings. Control group was also offered postal participation in mailings 1 and 3 while the push‐to‐web treatment group was only sent the postal response forms in mailing 3.	
***Risk of bias***		
**Item**	**Authors' judgement**	**Support for judgement**
Sequence generation	No	Random allocation: alternation
Allocation concealment?	No	Random allocation: alternation
Blinding of participants and personnel	Unclear	Blinding of personnel not described. Participants were not blinded to intervention.
Blinding of outcome assessment	Yes	No outcomes were assessed (counts of responses only).
Incomplete outcome data	No	Exclusions not reported
Selective reporting	Yes	Final response after 3 reminders reported in full
Other sources of bias	No	Sequential randomisation

**Table:** Weilbacher 1952

***Study characteristics***		
Methods	Random allocation: method not specified	
Data	University alumni members (Columbia, US)	
Comparisons	1. Personalised letter of transmittal 2. Non‐personalised letter of transmittal	
Outcomes	Response period not specified	
Topic	Not specified	
Mode of Administration	Postal	
Notes	—	
***Risk of bias***		
**Item**	**Authors' judgement**	**Support for judgement**
Allocation concealment?	Unclear	B ‐ unclear

**Table:** Weir 1999

***Study characteristics***		
Methods	Random allocation: computer algorithm	
Data	Patients with cerebrovascular disease discharged from hospital	
Comparisons	1. Questionnaire sent via GP 2. Questionnaire sent direct to participants by research group	
Outcomes	—	
Topic	Health: stroke outcomes	
Mode of Administration	Postal	
Notes	—	
***Risk of bias***		
**Item**	**Authors' judgement**	**Support for judgement**
Allocation concealment?	Unclear	B ‐ unclear

**Table:** Wells 1984

***Study characteristics***		
Methods	Random allocation: method not specified	
Data	University undergraduates	
Comparisons	1. University sponsor; business‐reply return envelope 2. University sponsor; no return postage 3. IRE sponsor; business‐reply return envelope 4. IRE sponsor; no return postage	
Outcomes	Response period not specified	
Topic	Non‐health: attitude measure ‐ degree of satisfaction with the university's contribution to personal development	
Mode of Administration	Postal	
Notes	Mainly females	
***Risk of bias***		
**Item**	**Authors' judgement**	**Support for judgement**
Allocation concealment?	Unclear	B ‐ unclear

**Table:** Weltzien 1986

***Study characteristics***		
Methods	Random allocation: method not specified	
Data	Individuals who had terminated from mental health treatment centres	
Comparisons	1. 2 cents incentive with questionnaire 2. No incentive	
Outcomes	Response within 4 months	
Topic	Health: Consumer Satisfaction Questionnaire (CSQ)	
Mode of Administration	Postal	
Notes	—	
***Risk of bias***		
**Item**	**Authors' judgement**	**Support for judgement**
Allocation concealment?	Unclear	B ‐ unclear

**Table:** Wenemark 2010

***Study characteristics***		
Methods	Random allocation: method unspecified	
Data	Parents of children with cancer, Stockholm, Sweden	
Comparisons	No incentive vs. conditional non‐monetary incentive (US $1.6 lottery ticket) vs. conditional non‐monetary incentive (US $1.6 lottery ticket plus an additional lottery ticket upon reply within 1 week)	
Outcomes	First response and final response after 3 reminders	
Topic	Health (stress in parents of children with cancer)	
Mode of Administration	Postal	
Notes	5 pages; questions on background, and three scales assessing subjective perceptions of stress, burnout and strain Letter with the logo of the Children's Cancer Foundation of Sweden, questionnaire plus an SAE. Reminders sent after 1, 3 and 6 weeks	
***Risk of bias***		
**Item**	**Authors' judgement**	**Support for judgement**
Sequence generation	Unclear	Random allocation: method unspecified
Allocation concealment?	Unclear	Random allocation: method unspecified
Blinding of participants and personnel	Unclear	Blinding of personnel not described. Participants were not blinded to intervention.
Blinding of outcome assessment	Yes	No outcomes were assessed (counts of responses only).
Incomplete outcome data	No	Exclusions not reported
Selective reporting	Yes	First response and final response after 3 reminders reported in full
Other sources of bias	Yes	No grounds for concern about other sources of bias from reading the study report

**Table:** Wensing 1999a

***Study characteristics***		
Methods	Random allocation: method not specified	
Data	Adult patients who had visited a GP	
Comparisons	1. Postal reminders 2. No reminders sent Reminder questionnaires sent at 3 weeks	
Outcomes	—	
Topic	Health: Europep ‐ patients' evaluation of general practice care	
Mode of Administration	Postal	
Notes	—	
***Risk of bias***		
**Item**	**Authors' judgement**	**Support for judgement**
Allocation concealment?	Unclear	B ‐ unclear

**Table:** Wensing 1999b

***Study characteristics***		
Methods	Random allocation: method not specified	
Data	Adult patients who had visited a GP	
Comparisons	1. Postal reminders 2. No reminders sent Reminder questionnaires sent at 3 weeks	
Outcomes	—	
Topic	Health: Europep ‐ patients' evaluation of general practice care	
Mode of Administration	Postal	
Notes	—	
***Risk of bias***		
**Item**	**Authors' judgement**	**Support for judgement**
Allocation concealment?	Unclear	B ‐ unclear

**Table:** Wensing 2005

***Study characteristics***		
Methods	Random allocation: computerised random number generation	
Data	Elderly adults registered with 26 general practitioners in the Netherlands	
Comparisons	1. Simple reminder card 2. Reminder + questionnaire 3. Reminder with request to explain non‐participation	
Outcomes	Response period not specified	
Topic	Health: health problems, health information sought, and attendance of general practice	
Mode of Administration	Postal	
Notes	—	
***Risk of bias***		
**Item**	**Authors' judgement**	**Support for judgement**
Allocation concealment?	Unclear	B ‐ unclear

**Table:** Whitcomb 2004

***Study characteristics***		
Methods	Random allocation: method not specified	
Data	High school students who did not apply to the Liberal Arts College	
Comparisons	1. Email file format ‐ text 2. Email file format ‐ HTML 3. Background colour ‐ white 4. Background colour ‐ black 5. Graphical design (header) ‐ simple (institution name only) 6. Graphical design (header) ‐ complex (mimicked University homepage ‐ institutions name, campus photograph, quotation from the University president)	
Outcomes	Response period not specified	
Topic	Non‐health: perception of the college, reason for not applying	
Mode of Administration	Electronic: Web‐survey	
Notes	—	
***Risk of bias***		
**Item**	**Authors' judgement**	**Support for judgement**
Allocation concealment?	Unclear	B ‐ unclear

**Table:** White 1997

***Study characteristics***		
Methods	Random allocation: method not specified	
Data	A random sample of marriage and family therapists from a list of all approved supervisors of the American Association of Marriage and Family Therapy	
Comparisons	1. Personalised cover letter; white questionnaire 2. Personalised cover letter; blue questionnaire 3. Generic cover letter; white questionnaire 4. Generic cover letter; blue questionnaire	
Outcomes	Response period not specified	
Topic	Health: demographics, Marriage and Family Therapist's supervision	
Mode of Administration	Postal	
Notes	—	
***Risk of bias***		
**Item**	**Authors' judgement**	**Support for judgement**
Allocation concealment?	Unclear	B ‐ unclear

**Table:** White 2005a

***Study characteristics***		
Methods	Random allocation: method not specified	
Data	Participants from the New Hampshire Women for Health (NHWH) study	
Comparisons	1. Inclusion of a pen in the second mailing study 2. No pen in the second mailing study	
Outcomes	Response period within 60 days	
Topic	Health: hormone replacement therapy, breast cancer, health‐related quality of life	
Mode of Administration	Postal	
Notes	—	
***Risk of bias***		
**Item**	**Authors' judgement**	**Support for judgement**
Allocation concealment?	Unclear	B ‐ unclear

**Table:** White 2005b

***Study characteristics***		
Methods	Random allocation: method not specified	
Data	Vanguard participants from the 13 counties of Western Washington State	
Comparisons	1. Inclusion of a pencil in the second mailing study 2. No pencil in the second mailing study	
Outcomes	Response period not specified	
Topic	Health: vitamins and lifestyle	
Mode of Administration	Postal	
Notes	—	
***Risk of bias***		
**Item**	**Authors' judgement**	**Support for judgement**
Allocation concealment?	Unclear	B ‐ unclear

**Table:** Whitehead 2011

***Study characteristics***		
Methods	Random allocation: computer‐generated (randomisation feature in Microsoft Excel 2007)	
Data	Sample of students (n = 1969) randomly selected from a database containing all students enrolled at a university, New Zealand	
Comparisons	Postal vs. electronic	
Outcomes	Response after 3 reminders	
Topic	Health (anxiety and depression in non‐psychiatric populations)	
Mode of Administration	Postal/electronic	
Notes	The questionnaire contained 41 items: the HADS (14 items), the SF‐12 v2 (12 items), a single fatigue item, and the Fatigue Symptom Inventory (FSI) (14 items). The mail group were sent a letter of introduction and the questionnaire to their home address. A stamped, self‐addressed envelope was included. 2 reminders at weeks 2 and 4. The online group was emailed an invitation to participate with a web link.	
***Risk of bias***		
**Item**	**Authors' judgement**	**Support for judgement**
Sequence generation	Yes	Random allocation: computer‐generated (randomisation feature in Microsoft Excel 2007)
Allocation concealment?	Yes	Random allocation: computer‐generated (randomisation feature in Microsoft Excel 2007)
Blinding of participants and personnel	Unclear	Blinding of personnel not described. Participants were not blinded to intervention.
Blinding of outcome assessment	Yes	No outcomes were assessed (counts of responses only).
Incomplete outcome data	Yes	Exclusions reported
Selective reporting	Yes	Response after 3 reminders reported in full
Other sources of bias	Yes	No grounds for concern about other sources of bias from reading the study report

**Table:** Whiteman 2003

***Study characteristics***		
Methods	Random allocation: computerised random number generation	
Data	Women in the Baltimore Metropolitan area who reported their history of hot flashes	
Comparisons	1. Introductory postcard mailed 1 week before the questionnaire 2. Scratch‐off lottery ticket worth $1.00 3. $1 bill 4. No incentives	
Outcomes	Response period within 95 days	
Topic	Health: risk of hot flashes in midlife women, pregnancy history, hormonal contraceptive use, menstrual history	
Mode of Administration	Postal	
Notes	Method of allocation ascertained through contact with author	
***Risk of bias***		
**Item**	**Authors' judgement**	**Support for judgement**
Allocation concealment?	Yes	A ‐ adequate

**Table:** Whiteside 2019

***Study characteristics***		
Methods	Random allocation: computer‐generated allocation sequence (using STATA)	
Data	1862 community dwelling, older adults at elevated risk of falling (eligible for the OTIS trial)	
Comparisons	Pen vs. no pen	
Outcomes	Return of questionnaire (consent form)	
Topic	Health (risk of falling)	
Mode of Administration	Postal	
Notes	Questionnaire (consent form) not described. Invitation letter, a participant information sheet, consent form, screening form, and a pre‐paid return envelope. Participants allocated to the intervention group of this embedded trial also received a York Trials Unit branded pen in their invitation pack. The control participants did not receive a pen in their invitation pack. Recipients of an invitation pack were asked to return a completed consent form and screening form if they were willing to take part in the OTIS trial.	
***Risk of bias***		
**Item**	**Authors' judgement**	**Support for judgement**
Sequence generation	Unclear	Random allocation: computer‐generated allocation sequence (using STATA)
Allocation concealment?	Unclear	Random allocation: computer‐generated allocation sequence (using STATA)
Blinding of participants and personnel	Unclear	Blinding of personnel not described. Participants were not blinded to intervention.
Blinding of outcome assessment	Yes	No outcome assessment used (counts of responses only)
Incomplete outcome data	Yes	Exclusions reported
Selective reporting	Yes	Outcomes reported in full
Other sources of bias	Yes	No grounds for concern about other sources of bias from reading the study report

**Table:** Whitmore 1976

***Study characteristics***		
Methods	Random allocation: method not specified	
Data	Individuals who had purchased a new car	
Comparisons	1. Key ring incentive with questionnaire 2. No incentive Follow‐up sent at 2 weeks	
Outcomes	—	
Topic	Not specified	
Mode of Administration	Postal	
Notes	—	
***Risk of bias***		
**Item**	**Authors' judgement**	**Support for judgement**
Allocation concealment?	Unclear	B ‐ unclear

**Table:** Wiant 2018

***Study characteristics***		
Methods	Random allocation: method unspecified	
Data	Medical oncologists drawn from the American Medical Association Physician Masterfile who had treated cancer patients within the previous 12 months	
Comparisons	Unconditional non‐monetary incentive ($50 cheque) vs. conditional non‐monetary incentive ($50 cheque)	
Outcomes	First response and response after 2 reminders and a phone call	
Topic	Health (experiences, attitudes, and recommendations concerning genomic testing in oncology)	
Mode of Administration	Postal	
Notes	12 pages. Personalised cover letter, an endorsement letter from the American Society of Clinical Oncology, a pen with the study's name printed on it, questionnaire booklet, and a business reply envelope	
***Risk of bias***		
**Item**	**Authors' judgement**	**Support for judgement**
Sequence generation	Unclear	Random allocation: method unspecified
Allocation concealment?	Unclear	Random allocation: method unspecified
Blinding of participants and personnel	Unclear	Blinding of personnel not described. Participants were not blinded to intervention.
Blinding of outcome assessment	Yes	No outcomes were assessed (counts of responses only).
Incomplete outcome data	Yes	Exclusions reported
Selective reporting	Yes	First response and response after 2 reminders and a phone call reported in full
Other sources of bias	Yes	No grounds for concern about other sources of bias from reading the study report

**Table:** Willits 1995

***Study characteristics***		
Methods	Random allocation: method not specified	
Data	Residents of Pennsylvania (US)	
Comparisons	1. No preamble; general question first 2. No preamble; general question last 3. Preamble; general first 4. Preamble; general last Follow‐up sent to non‐respondents (postcard and 2 additional mailings including another copy of the questionnaire)	
Outcomes	—	
Topic	Health: quality of life (QoL) in rural areas, QoL in relation to community spirit, healthcare services, recreational opportunities, job opportunities, air quality	
Mode of Administration	Postal	
Notes	Mean age: 42.6 years; mainly males	
***Risk of bias***		
**Item**	**Authors' judgement**	**Support for judgement**
Allocation concealment?	Unclear	B ‐ unclear

**Table:** Wilson 2010

***Study characteristics***		
Methods	Random allocation: computer‐generated	
Data	536 UK‐based principal investigators of health services and public health research completed during 2003‐2008	
Comparisons	'Knowledge of' vs. 'no knowledge of' a conditional £10 Amazon gift voucher	
Outcomes	Response after 5 reminders	
Topic	Non‐health (practices of intramural MRC Research Units)	
Mode of Administration	Web survey	
Notes	36 questions that could be completed in 20‐30 minutes. Participants were contacted by email and invited to complete an online questionnaire via URL. Four reminder emails sent out to non‐respondents (one, two, three, and four weeks), with a fifth postal reminder The email sent to the participants in the 'knowledge' group stated that those who completed the online questionnaire would receive a £10 Amazon gift voucher.	
***Risk of bias***		
**Item**	**Authors' judgement**	**Support for judgement**
Sequence generation	Yes	Random allocation: computer‐generated
Allocation concealment?	Yes	Random allocation: computer‐generated
Blinding of participants and personnel	Unclear	Blinding of personnel not described. Participants were not blinded to intervention.
Blinding of outcome assessment	Yes	No outcome assessment used (counts of responses only)
Incomplete outcome data	Yes	Exclusions reported
Selective reporting	Yes	Outcomes reported in full
Other sources of bias	Yes	No grounds for concern about other sources of bias from reading the study report

**Table:** Windsor 1992

***Study characteristics***		
Methods	Random allocation: method not specified	
Data	Individuals listed on electoral registers	
Comparisons	1. Questionnaire included questions on ethnic origin and housing tenure 2. Questionnaire included question on housing tenure only 3. Questionnaire included question on ethnic origin only 4. Neither question included 2 reminders sent	
Outcomes	Response period not specified	
Topic	Health: health and hospital survey ‐ health and hospital attendance, consultation with GPs, demographics, housing tenure	
Mode of Administration	Postal	
Notes	—	
***Risk of bias***		
**Item**	**Authors' judgement**	**Support for judgement**
Allocation concealment?	Unclear	B ‐ unclear

**Table:** Wiseman 1972

***Study characteristics***		
Methods	Random allocation: systematic allocation	
Data	Residents of a suburban Boston community, USA	
Comparisons	1. Telephone pre‐notification ‐ mail survey 2. No pre‐notification ‐ mail survey	
Outcomes	Response period not specified	
Topic	Health: birth control devices, legalising abortions, lowering the legal drinking age Non‐health: Giving state aid to Catholic schools	
Mode of Administration	Postal	
Notes	Method of allocation ascertained through contact with author	
***Risk of bias***		
**Item**	**Authors' judgement**	**Support for judgement**
Allocation concealment?	Unclear	B ‐ unclear

**Table:** Wiseman 1973

***Study characteristics***		
Methods	Random allocation: systematic allocation	
Data	Residents in statewide telephone listings, Massachusetts, USA	
Comparisons	1. 10 cent incentive (MI); postcard follow‐up 3 days after initial mailing (FU); business reply envelope (BRE); offer of survey results (OR) 2. MI; no follow‐up (no FU); BRE; OR 3. MI; FU; BRE; no offer of survey results (no OR) 4. MI; no FU; BRE; no OR 5. MI; FU; stamped return envelope (SRE); OR 6. MI; no FU; SRE; OR 7. MI; FU; SRE; no OR 8. MI; no FU; SRE; no OR 9. No monetary incentive (NI); FU; BRE; OR 10. NI; no FU; BRE; OR 11. NI; FU; BRE; no OR 12. NI; no FU; BRE; no OR 13. NI; FU; SRE; OR 14. NI; no FU; SRE; OR 15. NI; FU; SRE; no OR 16. NI; no FU; SRE; no OR	
Outcomes	Response period not specified	
Topic	Non‐health: attitudes and opinions about Massachusetts state lottery	
Mode of Administration	Postal	
Notes	Method of allocation ascertained through contact with author	
***Risk of bias***		
**Item**	**Authors' judgement**	**Support for judgement**
Allocation concealment?	Unclear	B ‐ unclear

**Table:** Wong 2021

***Study characteristics***		
Methods	Random allocation: computer‐generated	
Data	Adult patients discharged from the Royal Adelaide Hospital intensive care unit (ICU) between June 2018 and April 2019 who had spent at least 48 h in the ICU and who were still alive at the time of the follow‐up study.	
Comparisons	Postal vs. mixed‐mode, (online with paper follow‐up)	
Outcomes	First response and response after reminders	
Topic	Health (QoL, anxiety and depression)	
Mode of Administration	Postal/electronic	
Notes	EuroQoL‐5D‐5L, Hospital Anxiety and Depression Scale (HADS), and Impact of Event Scale‐Revised (IES‐R). Patients were emailed a website link to the survey; patients without email received a letter with a simple website address and a unique login. Email users were sent automated reminder emails after 3, 6, and 9 days. Forced responses were used in the online survey, requiring each question to be answered to progress. The online survey question order and wording were identical to that of the paper modality. In the paper group, patients received a paper survey with a postage‐paid reply envelope. In both groups, patients who had not completed their survey received a reminder paper survey after 14 days and a reminder phone call to return their received survey after 21 days.	
***Risk of bias***		
**Item**	**Authors' judgement**	**Support for judgement**
Sequence generation	Yes	Random allocation: computer‐generated
Allocation concealment?	Yes	Random allocation: computer‐generated
Blinding of participants and personnel	No	Patients were blinded to their group allocation; however, clinicians and researchers were not.
Blinding of outcome assessment	Yes	No outcomes were assessed (counts of responses only).
Incomplete outcome data	Yes	Exclusions were reported.
Selective reporting	Yes	First response and response after reminders reported in full
Other sources of bias	Yes	No grounds for concern about other sources of bias from reading the study report

**Table:** Woodward 1985

***Study characteristics***		
Methods	Random allocation: alternation	
Data	Household members (South Australia)	
Comparisons	1. Cover letter included offer of chance to win free dinner 2. Cover letter did not include offer Follow‐up at 1, 3 and 7 weeks	
Outcomes	Response within 10 weeks	
Topic	Health: respiratory history of the youngest child	
Mode of Administration	Postal	
Notes	—	
***Risk of bias***		
**Item**	**Authors' judgement**	**Support for judgement**
Allocation concealment?	No	C ‐ inadequate

**Table:** Woolf 2021

***Study characteristics***		
Methods	Random allocation: random number generator on a Casio calculator	
Data	Study corresponding authors included in a systematic review, whose studies were at unclear risk of bias	
Comparisons	Pre‐notification email vs. no pre‐notification	
Outcomes	Response after 4 weeks	
Topic	Non‐health (information on study design of an RCT)	
Mode of Administration	Electronic	
Notes	Questionnaire not described. All authors were emailed the questionnaire 1 day after the pre‐notification email was sent. Follow‐up contacts were sent at one and two weeks after the initial sending of the questionnaire, where a response had not been received. Other than the pre‐notification, all communication to the two arms were sent on the same day.	
***Risk of bias***		
**Item**	**Authors' judgement**	**Support for judgement**
Sequence generation	Yes	Random allocation: random number generator on a Casio calculator
Allocation concealment?	Yes	Random allocation: random number generator on a Casio calculator
Blinding of participants and personnel	Yes	No active blinding of participants or personnel occurred. However, no material risk of bias should have been introduced. Because participants were unaware of having been randomised, any effect of treatment could not be because of knowing that they had been specially selected for an intervention which others had not received. Although the participant still knew they had received the pre‐notification, this knowledge is part of the effect of a pre‐notification.
Blinding of outcome assessment	Yes	No outcomes were assessed (counts of responses only).
Incomplete outcome data	Yes	Exclusions were reported.
Selective reporting	Yes	Response after 4 weeks reported in full
Other sources of bias	Yes	No grounds for concern about other sources of bias from reading the study report

**Table:** Worthen 1985a

***Study characteristics***		
Methods	Random allocation: method not specified	
Data	School teachers listed on the Education Association Membership roster (Utah, US)	
Comparisons	1. Personalised cover letter 2. Form cover letter	
Outcomes	Response period not specified	
Topic	Non‐health: Classroom teachers' opinion about what should be taught on an educational measurement course	
Mode of Administration	Postal	
Notes	—	
***Risk of bias***		
**Item**	**Authors' judgement**	**Support for judgement**
Allocation concealment?	Unclear	B ‐ unclear

**Table:** Worthen 1985b

***Study characteristics***		
Methods	Random allocation: method not specified	
Data	School teachers listed on the Education Association Membership roster (Utah, US) who did not respond to an earlier questionnaire with a personalised letter	
Comparisons	1. Personalised cover letter 2. Form cover letter	
Outcomes	Response period not specified	
Topic	Non‐health: Classroom teachers' opinion about what should be taught on an educational measurement course	
Mode of Administration	Postal	
Notes	—	
***Risk of bias***		
**Item**	**Authors' judgement**	**Support for judgement**
Allocation concealment?	Unclear	B ‐ unclear

**Table:** Worthen 1985c

***Study characteristics***		
Methods	Random allocation: method not specified	
Data	School teachers listed on the Education Association Membership roster (Utah, US) who did not respond to an earlier questionnaire with a standard form letter	
Comparisons	1. Personalised cover letter 2. Form cover letter	
Outcomes	Response period not specified	
Topic	Non‐health: Classroom teachers' opinion about what should be taught on an educational measurement course	
Mode of Administration	Postal	
Notes	—	
***Risk of bias***		
**Item**	**Authors' judgement**	**Support for judgement**
Allocation concealment?	Unclear	B ‐ unclear

**Table:** Wotruba 1966

***Study characteristics***		
Methods	Random allocation: systematic division of a random sample	
Data	Urban household residents	
Comparisons	1. 25 cents sent with questionnaire 2. 50 cents promised on return of questionnaire 3. No incentive	
Outcomes	Response within 6 weeks	
Topic	Not specified	
Mode of Administration	Postal	
Notes	—	
***Risk of bias***		
**Item**	**Authors' judgement**	**Support for judgement**
Allocation concealment?	No	C ‐ inadequate

**Table:** Wright 1984

***Study characteristics***		
Methods	Random allocation: alternation	
Data	People listed in the latest telephone directories, New Zealand 1983	
Comparisons	1. Personal salutation (P); black and white letterhead (BW); white outward envelope (Wh); $100 cash lottery incentive (Ca) 2. P; BW; Wh; garden voucher lottery incentive (Ga) 3. P; BW; brown outward envelope (Br); Ca 4. P; BW; Br; Ga 5. P; coloured letterhead (Co); Wh; Ca 6. P; Co; Wh; Ga 7. P; Co; Br; Ca 8. P; Co; Br; Ga 9. Impersonal salutation (IP); BW; Wh; Ca 10. IP; BW; Wh; Ga 11. IP; BW; Br; Ca 12. IP; BW; Br; Ga 13. IP; Co; Wh; Ca 14. IP; Co; Wh; Ga 15. IP; Co; Br; Ca 16. IP; Co; Br; Ga	
Outcomes	Response period not specified	
Topic	Non‐health: motivation of gardeners and users of garden products, sociodemographics	
Mode of Administration	Postal	
Notes	—	
***Risk of bias***		
**Item**	**Authors' judgement**	**Support for judgement**
Allocation concealment?	No	C ‐ inadequate

**Table:** Wright 1995

***Study characteristics***		
Methods	Random allocation: systematic sample from list ordered alphabetically	
Data	New Zealand councillors who had participated in another survey 18 months previously	
Comparisons	1. Pre‐notification letter sent 2 weeks prior to questionnaire mailing 2. No pre‐contact	
Outcomes	Response after 2 follow‐up reminders	
Topic	Not specified	
Mode of Administration	Postal	
Notes	—	
***Risk of bias***		
**Item**	**Authors' judgement**	**Support for judgement**
Allocation concealment?	Unclear	B ‐ unclear

**Table:** Wunder 1988

***Study characteristics***		
Methods	Random allocation: alternation	
Data	Subscribers to a large health maintenance organisation in a major metropolitan area in the Midwestern United States	
Comparisons	1. Hand‐addressed envelope 2. Computer‐generated address on envelope	
Outcomes	Response period not specified	
Topic	Health: satisfaction benefit package, characteristics of subscribers	
Mode of Administration	Postal	
Notes	Method of allocation ascertained through contact with author	
***Risk of bias***		
**Item**	**Authors' judgement**	**Support for judgement**
Allocation concealment?	No	C ‐ inadequate

**Table:** Wynn 1985

***Study characteristics***		
Methods	Random allocation: alternation	
Data	Members, past and present, of an exercise and recreational club in a medium‐sized southwestern city (US)	
Comparisons	1. No pre‐contact by telephone 2. Telephone pre‐contact asking permission to send questionnaire (foot‐in‐the‐door manipulation) 3. Telephone pre‐contact asking questions (probe‐foot‐in‐the‐door manipulation)	
Outcomes	Response period not specified	
Topic	Health: planning of a possible expansion effort for an exercise recreational club	
Mode of Administration	Postal	
Notes	—	
***Risk of bias***		
**Item**	**Authors' judgement**	**Support for judgement**
Allocation concealment?	No	C ‐ inadequate

**Table:** Xie 2013

***Study characteristics***		
Methods	Random allocation: method unspecified	
Data	Nurses selected from the Association of Hong Kong Nursing Staff (AHKNS)	
Comparisons	Pre‐notification vs. no prenotification	
Outcomes	Response after one reminder	
Topic	Health (demographics, work status, lifestyle factors, reproductive information, and dietary habits)	
Mode of Administration	Postal	
Notes	153 items printed on a 4‐page, double‐sided booklet. The cover page provided a brief introduction to the study. An informed consent form was also attached. Two rounds of mailing were conducted. In the first round of mailing, nurses in the direct questionnaire mailing group received the questionnaire with a prepaid return envelope, whereas those in the pre‐notification group received a pre‐notification package that included an introduction leaflet with a reply slip, and a prepaid return envelope. After 3 weeks, the second round of mailing was conducted.	
***Risk of bias***		
**Item**	**Authors' judgement**	**Support for judgement**
Sequence generation	Unclear	Random allocation: method unspecified
Allocation concealment?	Unclear	Random allocation: method unspecified
Blinding of participants and personnel	Yes	The investigators and participants remained unaware about the group allocation.
Blinding of outcome assessment	Yes	No outcomes were assessed (counts of responses only).
Incomplete outcome data	Yes	Exclusions were reported.
Selective reporting	Yes	Response after one reminder reported in full
Other sources of bias	Yes	No grounds for concern about other sources of bias from reading the study report

**Table:** Yetter 2010

***Study characteristics***		
Methods	Random allocation: method unspecified	
Data	School psychologists with an email address, US	
Comparisons	Shorter vs. longer Electronic vs. postal	
Outcomes	Response after 2 reminders	
Topic	Non‐health (perceptions of pre‐referral intervention teams for addressing children's school‐related academic and behaviour difficulties)	
Mode of Administration	Electronic vs. postal	
Notes	Shorter (20 items), longer (66 items). Postal pre‐notifications were sent first. Then survey packets (for the paper survey participants) and emails (for Web participants) containing a URL were sent, followed by 2 reminders to participate. The online survey was made to look as similar as possible to the printed version. It appeared in black and white only, and the radio buttons were presented in click format. The participants were able to navigate forward and backward through the survey instrument by clicking on next and back arrow at the bottom of each screen. On completing the questionnaire, the participants were presented with a 'Success' Web page.	
***Risk of bias***		
**Item**	**Authors' judgement**	**Support for judgement**
Sequence generation	Unclear	Random allocation: method unspecified
Allocation concealment?	Unclear	Random allocation: method unspecified
Blinding of participants and personnel	Unclear	Blinding of personnel not described. Participants were not blinded to intervention.
Blinding of outcome assessment	Yes	No outcome assessment used (counts of responses only)
Incomplete outcome data	Yes	Exclusions reported
Selective reporting	Yes	Outcomes reported in full
Other sources of bias	Yes	No grounds for concern about other sources of bias from reading the study report

**Table:** Young 2015

***Study characteristics***		
Methods	Random allocation: computer‐generated	
Data	GPs in New South Wales (NSW) and Victoria, Australia	
Comparisons	Unconditional non‐monetary incentive vs. conditional non‐monetary incentive vs. no‐incentive	
Outcomes	Response after 3 reminders	
Topic	Health (cancer care)	
Mode of Administration	Web	
Notes	Questionnaire not described. GPs were mailed an advance letter approximately 1 week before the main letter of invitation. Up to 3 mailed reminder letters were sent to non‐responders at biweekly intervals. The incentive was a book voucher ($75 in NSW and $50 in Victoria)	
***Risk of bias***		
**Item**	**Authors' judgement**	**Support for judgement**
Sequence generation	Yes	Random allocation: computer‐generated
Allocation concealment?	Yes	Random allocation: computer‐generated
Blinding of participants and personnel	Unclear	Blinding of personnel not described. Participants were not blinded to intervention.
Blinding of outcome assessment	Yes	No outcomes were assessed (counts of responses only).
Incomplete outcome data	Yes	Exclusions were reported.
Selective reporting	Yes	Response after 3 reminders reported in full
Other sources of bias	Yes	No grounds for concern about other sources of bias from reading the study report

**Table:** Young 2020

***Study characteristics***		
Methods	Random allocation: computer‐generated	
Data	Participants in a cohort from a host trial of screening for lung cancer	
Comparisons	Unconditional £5 multi‐store paper gift voucher vs. conditional £5 multi‐store paper gift voucher	
Outcomes	Response after 5 weeks (2 telephone reminders and one postal reminder)	
Topic	Health (cancer, depression)	
Mode of Administration	Postal	
Notes	16 pages long and included the Hospital Anxiety and Depression Scale, Positive and Negative Affect Schedule, Illness Perceptions Questionnaire, Cancer Worry Scale, and measures of health status, health anxiety, perceived risk of lung cancer and tobacco use. A4 sized questionnaire sent folded in half and had participants' initials and unique study ID numbers handwritten on the front. Envelopes were sent with second‐class postage stamps, printed address labels, and a prepaid second‐class return envelope enclosed. One week after the first questionnaire was mailed, a telephone call was made to check receipt of the questionnaire, answer any questions, and encourage its return by emphasising the importance of the research. If telephone contact was not made after two attempts, a brief scripted voicemail was left where possible. If a questionnaire was not returned 2 weeks after mailing, an identical reminder copy was sent with another prepaid envelope. If the questionnaire was not returned 3 weeks after mailing, a reminder telephone call was attempted. Two call attempts were made and, if unsuccessful, a voicemail was left where possible. Questionnaires had a £5 multi‐store paper gift voucher attached to the front with a paper clip. Questionnaires were sent at 3, 6, and 12 months after baseline and responses recorded at each time point.	
***Risk of bias***		
**Item**	**Authors' judgement**	**Support for judgement**
Sequence generation	Yes	Random allocation: computer‐generated
Allocation concealment?	Yes	Random allocation: computer‐generated
Blinding of participants and personnel	Unclear	Blinding of personnel not specified. Participants were not blinded.
Blinding of outcome assessment	Yes	No outcomes assessed (response only)
Incomplete outcome data	Yes	Exclusions were reported.
Selective reporting	Yes	Outcomes at all time points reported
Other sources of bias	Yes	No grounds for concern about other sources of bias from reading the study report

**Table:** Yu 2017

***Study characteristics***		
Methods	Random allocation: method unspecified	
Data	35,421 non‐respondents to the 4th wave survey of participants in the World Trade Centre Health Registry	
Comparisons	Conditional $10 cash incentive vs. no incentive	
Outcomes	Final response after 10 email reminders with survey links, and 4 postcard reminders	
Topic	Health (long‐term health impact of the September 11, 2001 (9/11) terrorist attacks in New York City)	
Mode of Administration	Postal/electronic	
Notes	During the incentive experiment period, reminder phone calls and door‐to‐door outreach were also conducted concurrently, in addition to email and postcard reminders that were sent frequently throughout the data collection period.	
***Risk of bias***		
**Item**	**Authors' judgement**	**Support for judgement**
Sequence generation	Unclear	Random allocation: method unspecified
Allocation concealment?	Unclear	Random allocation: method unspecified
Blinding of participants and personnel	Unclear	Blinding of personnel not described. Participants were not blinded to intervention.
Blinding of outcome assessment	Yes	No outcome assessment used (counts of responses only)
Incomplete outcome data	No	Exclusions not reported
Selective reporting	Yes	Outcomes reported in full
Other sources of bias	Yes	No grounds for concern about other sources of bias from reading the study report

**Table:** Ziegenfuss 2010

***Study characteristics***		
Methods	Random allocation: method unspecified	
Data	770 randomly selected Olmsted County residents aged 25‐65 years	
Comparisons	Postal vs. choice (postal/Web)	
Outcomes	Response after one reminder	
Topic	Health (bowel disease)	
Mode of Administration	Mixed	
Notes	16 pages. The survey packets were equivalent except for a Web address and personal identification number in the cover letter for the Internet option. All individuals received a topically related book as an incentive. A reminder letter was mailed to non‐responders 3 weeks after the initial mailing.	
***Risk of bias***		
**Item**	**Authors' judgement**	**Support for judgement**
Sequence generation	Unclear	Random allocation: method unspecified
Allocation concealment?	Unclear	Random allocation: method unspecified
Blinding of participants and personnel	Unclear	Blinding of personnel not described. Participants were not blinded to intervention.
Blinding of outcome assessment	Yes	No outcomes were assessed (counts of responses only).
Incomplete outcome data	Yes	Exclusions were not reported.
Selective reporting	Yes	Response after one reminder reported in full
Other sources of bias	Yes	No grounds for concern about other sources of bias from reading the study report

**Table:** Ziegenfuss 2011

***Study characteristics***		
Methods	Random allocation: method unspecified	
Data	Individuals known to have diabetes mellitus in the Mayo Health System	
Comparisons	Offer of survey results as incentive vs. none	
Outcomes	Final response after one reminder	
Topic	Health (diabetes care delivery in the primary‐care setting)	
Mode of Administration	Postal	
Notes	5‐page booklet. The package contained a cover letter signed by the study principal investigator and a local diabetes physician champion. Non‐respondents were sent a second letter and questionnaire 4 weeks after the first mailing. The cover letter sent to those in the intervention arm included an additional paragraph that stated that as a 'token of appreciation', the respondent would have the opportunity to indicate in the last question of the survey whether they wanted a summary of the results sent to them after they completed the survey.	
***Risk of bias***		
**Item**	**Authors' judgement**	**Support for judgement**
Sequence generation	Unclear	Random allocation: method unspecified
Allocation concealment?	Unclear	Random allocation: method unspecified
Blinding of participants and personnel	Unclear	Blinding of personnel not described. Participants were not blinded to intervention.
Blinding of outcome assessment	Yes	No outcomes were assessed (counts of responses only).
Incomplete outcome data	No	Exclusions not reported
Selective reporting	Yes	Outcomes reported in full
Other sources of bias	Yes	No grounds for concern about other sources of bias from reading the study report

**Table:** Ziegenfuss 2012

***Study characteristics***		
Methods	Random allocation: method unspecified	
Data	2000 practising US physicians selected from the AMA Physician Masterfile who had not responded to the first wave of a survey	
Comparisons	Envelope with a brightly coloured sticker reading "$25 incentive" vs. no envelope sticker	
Outcomes	Response period not specified	
Topic	Health (moral beliefs and views on controversial healthcare topics)	
Mode of Administration	Postal	
Notes	8‐page questionnaire. Package not described	
***Risk of bias***		
**Item**	**Authors' judgement**	**Support for judgement**
Sequence generation	Unclear	Random allocation: method unspecified
Allocation concealment?	Unclear	Random allocation: method unspecified
Blinding of participants and personnel	Unclear	Blinding of personnel not described. Participants were not blinded to intervention.
Blinding of outcome assessment	Yes	No outcomes were assessed (counts of responses only).
Incomplete outcome data	No	Exclusions were not reported.
Selective reporting	No	Response period not specified
Other sources of bias	Yes	No grounds for concern about other sources of bias from reading the study report

**Table:** Ziegenfuss 2014

***Study characteristics***		
Methods	Random allocation: computer‐generated	
Data	Practising registered nurses and physicians in Minnesota, US	
Comparisons	Padded envelope vs. priority mail envelope	
Outcomes	First response	
Topic	Health (practices and perspectives on H1N1 pandemic influenza vaccination)	
Mode of Administration	Postal	
Notes	7 pages. Cover letter, an endorsement letter from the Minnesota Department of Health, a postage‐paid return envelope, and a laser pointer pen as a thank‐you gift	
***Risk of bias***		
**Item**	**Authors' judgement**	**Support for judgement**
Sequence generation	Yes	Random allocation: computer‐generated
Allocation concealment?	Yes	Random allocation: computer‐generated
Blinding of participants and personnel	Unclear	Blinding of personnel not described. Participants were not blinded to intervention.
Blinding of outcome assessment	Yes	No outcomes were assessed (counts of responses only).
Incomplete outcome data	No	Exclusions were not reported.
Selective reporting	No	Only outcomes after initial mailing reported
Other sources of bias	Yes	No grounds for concern about other sources of bias from reading the study report

**Table:** Zusman 1987

***Study characteristics***		
Methods	Random allocation: on the basis of study identification number and done without reference to subject characteristics	
Data	Undergraduate transfer students	
Comparisons	1. $1 incentive sent with first mailing 2. No incentive sent Follow‐up of non‐respondents several weeks after first mailing	
Outcomes	Response period not specified	
Topic	Not specified	
Mode of Administration	Postal	
Notes	—	
***Risk of bias***		
**Item**	**Authors' judgement**	**Support for judgement**
Allocation concealment?	Unclear	B ‐ unclear

DNA: Deoxyribonucleic acid EQ‐5D: EuroQol‐ 5 Dimension GP: General practice NHS: National Health Service SAE: Self‐addressed envelope SWAT: Study within a Trial SF‐12: 12‐Item Short Form Survey SF‐36: 36‐Item Short Form Health Survey SMS: Short Message Service

### Characteristics of excluded studies [ordered by study ID]

**Table:**

Study	Reason for exclusion
Alcaraz 2020	Not postal questionnaire
Alexander 2008a	Not postal questionnaire
Allen 1980	The comparison in this study is biased by the fact that people in the pre‐notification group were given the choice of whether to receive the questionnaire or not whereas people in the no pre‐notification group were not given this choice.
Anderson 1975	It was not possible to determine whether this study was randomised.
Anderson 2007	Not a randomised trial
Angus 2003	Not a randomised controlled trial
Antoun 2020	Not postal questionnaire
Armstrong 1975	Review article
Asch 1994	The comparison in this study was confounded ‐ the author, with reference to the several differences between the 2 mailing strategies, stated 'We cannot determine which of these differences underlies our results.'
Ash 1952	It was not possible to determine whether this study was randomised. Attempts to contact the author have been unsuccessful.
Baron 2001	The comparison in this study was confounded by the colour of the questionnaire.
Beaty 2018	Not a randomised trial
Becker 2019	Not a randomised trial
Beebe 2011	Not postal questionnaire
Berthelot 1993	Not a randomised trial
Bevis 1948	It was not possible to determine whether this study was randomised.
Beynon 2010	Not postal questionnaire
Biggar 1992	All comparisons in the study were confounded.
Blumberg 1974	It was not possible to determine whether this study was randomised and the data which would be needed were only referred to, not presented. Attempts to contact the author have been unsuccessful.
Blumenfeld 1973	It was not going to be possible to determine whether this study was randomised as the author had died.
Boucher 2015	Not a randomised trial
Brealey 2007	Not a randomised trial
Brechner 1976	It was not possible to determine whether this study was randomised. Attempts to contact the author have been unsuccessful.
Breeman 2013	Not a randomised trial
Brennan 1958	It was not possible to determine whether this study was randomised. Attempts to contact the author have been unsuccessful.
Brennan 1990	The comparison in this study was confounded.
Burkhart 2021	Not a randomised trial
Cadilhac 2012	Not postal questionnaire
Callegaro 2010	Not postal questionnaire
Cartwright 1968	The comparison of different lengths was confounded by other differences between the two questionnaires.
Cartwright 1989	It was not possible to determine whether this study was randomised. Contact details of the author were unavailable.
Cernat 2018	Not postal questionnaire
Champion 1969	It was not possible to determine whether this study was randomised. Attempts to contact the author have been unsuccessful.
Chen 2016	Not a randomised trial
Cheung 2019	Not postal questionnaire
Childs 2005	The study did not calculate the response for the different order of administration of the questionnaires.
Chin 2015	Not postal questionnaire
Choi 2017	Not a randomised trial
Chyou 2017	Not a randomised trial
Clark 2008	Not postal questionnaire
Coleman 2021	Not postal questionnaire
Coleman 2021a	Not postal questionnaire
Conrad 2007	Not postal questionnaire
Cook 1985	Incentive only given after agreement to participate in a further study, not just for returning the questionnaire.
Dal Grande 2016	Not postal questionnaire
Desborough 2008	Not postal questionnaire
Dillman 1972	No useful experimental data presented
Down 2010	Not a randomised trial
Drake 2014	Not a randomised trial
Drummond 2015	Confounded by different incentive amounts and whether they were offered on conditional or unconditional basis (unconditional scratch card (with potential to win EURO 5000) vs. conditional lottery for one of three high street shopping vouchers worth EURO 300).
Duan 2007	Not postal questionnaire
Duncan 2019	Not a randomised trial
Dunlap 1950	It was not possible to determine whether this study was testing return rate of a questionnaire. Attempts to contact the author have been unsuccessful.
Eaton 2020	Not postal questionnaire
Ebert 2018	Not a randomised trial
Egeland 2017	Confounded trial ‐ cannot separate the effect of the modifed cover letter from the effects of the name of the patient handwritten at the top and with the handwritten signature of the surgeon
Eisinger 1974	It was not possible to determine whether this study was randomised. Attempts to contact authors have been unsuccessful.
Elinson 1950	There were insufficient data presented in this paper to include it. It has also not been possible to determine whether the questionnaire in the experiment was postal. Attempts to contact authors have been unsuccessful.
Ettridge 2021	Not postal questionnaire
Everett 1997	The comparison in this study was confounded by the colour of the questionnaire.
Fang 2006	This study did not calculate the response but inspected the correlation between the material incentive and the participants' characteristics.
Fang 2012	Not postal questionnaire
Fang 2021	Not a randomised trial
Fernandez Lynch 2019	Not postal questionnaire
Ferriss 1951	It was not possible to determine whether this study was randomised. Attempts to contact the author have been unsuccessful.
Fleming 2013	Not postal questionnaire
Fu 2013	Not postal questionnaire
Furse 1981	Authors could not remember whether the study was randomised.
Gerace 1995	This study examined response rates of a postal request for more information, not a questionnaire.
Gibson 2020	Not postal questionnaire
Gillespie 1975	The comparison in this study was confounded.
Gooden 2021	Confounded trial
Groves 2011	Not postal questionnaire
Hansen 2004	Not a randomised controlled trial
Hare 1998	The comparison in this study was confounded by the colour of the questionnaire.
Harlow 1993	Examined response rates to telephone interviews not postal questionnaires
Harrison 2019	Not a randomised trial
Haugejorden 1987	Randomised controlled trial but not of methods to increase response to postal questionnaires
Hawes 1987	Author no longer has original data to be able to provide confirmation of numbers of questionnaires administered and returned.
Heads 1966	It was not possible to determine whether this study was randomised. Attempts to contact the author have been unsuccessful.
Heje 2006	The primary questionnaire was delivered personally to the patient either at the surgery or at home.
Helgeson 2002	Author no longer has original data to be able to provide confirmation of numbers of questionnaires administered and returned.
Hennrich 2021	Not postal questionnaire
Hing 2005	Not a postal questionnaire
Hinrichs 1975	It was not possible to determine whether this study was randomised. Attempts to contact the author have been unsuccessful.
Hocking 2006	Not postal questionnaire
Horevoorts 2015	Not a randomised trial
Hsu 2017	Not postal questionnaire
Hughes 1989	Author was contacted: the study records have been discarded.
Hurd 2017	Not a randomised trial
Hurd 2019	Not a randomised trial
ISRCTN16437731 2016	Trial protocol
Ives 1990	Author was contacted: confirmed that participants were not randomly allocated.
Jacobson 2016	Not postal questionnaire
Jiang 2005	Not a randomised controlled trial
Jobber 2004	Not a randomised trial
Johnson 2014	Not postal questionnaire
Johnson 2015	Not a randomised trial
Kato 2021	Not a randomised trial
Kerin 1974	It was not possible to determine whether this study was randomised. Attempts to contact the author have been unsuccessful.
Kerin 1977	It was not possible to determine whether this study was randomised. Attempts to contact the author have been unsuccessful.
Kerin 1983	It was not possible to determine whether this study was randomised. Attempts to contact the author have been unsuccessful.
Kimball 1961	It was not possible to confirm that this study was randomised. Attempts to contact the author have been unsuccessful.
Kimel 2010	Not postal questionnaire
Koetsenruijter 2015	Not postal questionnaire
Kundig 2011	Not postal questionnaire
Labovitz 2017	Not postal questionnaire
Lane 2011	Not a randomised trial
Lane 2017	Not a randomised trial
Lapane 2007	Not postal questionnaire
Larsson 1970	It was not possible to determine whether this study was randomised. Attempts to contact the author have been unsuccessful.
Lavender 2009	Not postal questionnaire
Leece 2004a	Not a randomised trial
Li 2015	Confounded trial (Table 1 shows that experimental conditions varied by timing of delivery)
Lim 2020	Not postal questionnaire
Liu 2011	Confounded trial; size of incentive was confounded by type of incentive.
Longworth 1953	Author drew six different samples, and tested a different type of intervention on each without a comparison group.
Lopez‐ Cano 2007	Not a randomised controlled trial
Lund 1988	Comparisons of questionnaires which were mailed were confounded.
Malhotra 2008	Not postal questionnaire
Maloshonok 2016	Not postal questionnaire
Marks 1981	Author could not remember whether the study was randomised.
May 1960	It was not possible to determine whether this study was randomised. Attempts to contact the author have been unsuccessful.
McCree‐Hale 2010	Not a randomised trial
McDermott 2003	Incentives were the same for all the three questionnaires.
McDermott 2018	Not postal questionnaire
Mehta 1995	Two groups received postal questionnaires, but one group received a combination of methods (monetary incentive, pre‐notification and follow‐up). Comparisons for combinations of methods have not been created in this review.
Millar 2021	Not postal questionnaire
Murdoch 2010	Not postal questionnaire
Myers 2007	Confounded trial of university sponsorship (the ‘industry group’ received a cover letter containing logos of both the university and the industry sponsor).
Neve 2021	Not a randomised trial
Nitecki 1975	It was not possible to determine whether this study was randomised. Attempts to contact the author have been unsuccessful.
Nitikman 2015	Not a randomised trial
Nitikman 2017	Not postal questionnaire
Nord 2007	Not postal questionnaire
O'Toole 2008	Not postal questionnaire
Oden 1999	The comparison in this study was confounded by the colour of the questionnaire.
Onoye 2012	Not a randomised trial
Otzen 2020	Not a randomised trial
Palmer 2018	Not a randomised trial
Pariyo 2019	Not postal questionnaire
Perneger 2003	The intervention did not include strategies to increase response to a postal or electronic questionnaire.
Perneger 2014	Not a randomised trial
Peytremann‐Bridevaux 2006a	The intervention did not include strategies to increase response to a postal or electronic questionnaire.
Pieper 2018	Not postal questionnaire
Porter 2004	The data presented in this paper were the same as those presented in an earlier paper, Porter 2003.
Pottick 1991	This study examined postal methods to improve response to a face‐to‐face survey.
Prado 2012	Not a randomised trial
Rafiq 2016	Not postal questionnaire
Rashidian 2008	Not a randomised trial
Richards 2009	Not postal questionnaire
Richards 2010	Not a randomised trial
Robbins 2018	Not postal questionnaire
Robin 1973	It was not possible to determine whether this study was randomised. Attempts to contact the author have been unsuccessful.
Robin 1976	It was not possible to determine whether this study was randomised. Attempts to contact the author have been unsuccessful.
Rocheleau 2012	Not postal questionnaire
Roeher 1963	It was not possible to determine whether this study was randomised. Attempts to contact the author have been unsuccessful.
Rudd 1980	It was not possible to determine whether this study was randomised. Attempts to contact the author have been unsuccessful.
Ryan 2018	Not postal questionnaire
Salomone 1978	The number of people allocated to each experimental group was not presented and attempts to obtain this information from the authors have been unsuccessful.
Senf 1987	Option to refuse postcards were sent to half of all participants prior to sending questionnaire. However, response rates to questionnaires could not be compared because questionnaires were returned anonymously.
Shackleton 1982	The data presented in this paper were the same as those presented in an earlier paper by Shackleton (1980).
Shah 2016	Not postal questionnaire
Shermis 1982	Comparisons of questionnaires which were mailed were confounded.
Sheth 1975	The data presented in this paper were from the same study as those presented in an included study by Roscoe and Sheth (1975).
Shin 2015	Not postal questionnaire
Short 2015	Not a randomised trial
Simcoe 2015	Not a randomised trial
Sirken 1960	Could not confirm random allocation. Author contacted: stated only that "this was not a clinical trial."
Smith 1972	It was not possible to determine whether this study was randomised. Attempts to contact the author have been unsuccessful.
Smith 1977	It was not possible to determine whether this study was randomised. Attempts to contact the author have been unsuccessful.
Smith 1987	It was not possible to determine how many participants were allocated to each experimental group and attempts to obtain this information from the authors have been unsuccessful.
Smith 2019	Not a randomised trial
Snyder 1984	It was not possible to determine whether this study was randomised. Attempts to contact the author have been unsuccessful.
Stenhammar 2011	Not a randomised trial
Suhre 1989	Analyses by means of logit analysis and no useable outcome data were available. Author contacted: no useable data obtained
Sullivan 1995	Comparison groups did not meet 'postal questionnaire' criteria.
Sutherland 1996	There were too many differences between the two groups to be able to compare any of these differences without confounding.
Tan 1997	Review article
TCTR20190814001 2019	Not a randomised trial
Trice 1985	Not a postal questionnaire
Tucker‐Seeley 2009	Not a postal questionnaire
Udby 2021	Not a randomised trial
Van Ryswyk 2016	Not postal questionnaire
von Allmen 2019	Not postal questionnaire
Walker 1977	It was not possible to determine whether this study was randomised. Attempts to contact the author have been unsuccessful.
Wang 2018	Not postal questionnaire
Ward 1994	All comparisons in the study were confounded.
Warnock 2022	Not postal questionnaire
Watson 1965	It was not possible to determine whether this study was randomised. Attempts to contact the author have been unsuccessful.
Weiss 1985	It was not possible to determine whether this study was randomised. Attempts to contact the author have been unsuccessful.
Weissenburger 1987	It was not possible to determine whether this study was randomised. Contact details of the author were unavailable.
Weston 2017	Not a randomised trial
Wildman 1977	The comparison in this study was confounded by paper quality.
Willis 2013	Not postal questionnaire
Wood 2017	Not a randomised trial
Zagumny 1996	Not a postal questionnaire
Zheng 2018	Not postal questionnaire
Ziegenfuss 2012a	Not postal questionnaire
Zuidgeest 2011	Not a randomised trial
Zwisler 2004	Review article

### Characteristics of studies awaiting classification [ordered by study ID]

**Table:** Alexander 2008

Methods	Not yet assessed
Data	Not yet assessed
Comparisons	Not yet assessed
Outcomes	Not yet assessed
Notes

**Table:** Balabanis 2007

Methods	Not yet assessed
Data	Not yet assessed
Comparisons	Not yet assessed
Outcomes	Not yet assessed
Notes

**Table:** Bauman 2016

Methods	Computer‐generated (using SAS statistical program)
Data	Adults aged 45 up to 110 years living in the state of New South Wales, Australia
Comparisons	Pre‐contact postcard, then questionnaire 2 weeks later; questionnaire then a reminder letter 2 weeks later; pre‐contact postcard then questionnaire 2 weeks later then a reminder letter 2 weeks later
Outcomes	Response after 14 days
Notes	8 pages A one‐page reminder letter and the postcard provided brief information about the study, an invitation to participate, and were signed by the Study Scientific Director. Participants were provided with reply‐paid envelopes to return their completed questionnaires.

**Table:** Bhattarai 2010

Methods	Not yet assessed
Data	Not yet assessed
Comparisons	Not yet assessed
Outcomes	Not yet assessed
Notes

**Table:** Bosnjak 2015

Methods	Random allocation: method not specified
Data	562 undergraduate and graduate students enrolled at a German university
Comparisons	Email pre‐notification vs. SMS pre‐notification vs. no pre‐notification Email invitation vs. SMS invitation
Outcomes	Web surveys were sent out each month for 3 months. One week after invitations were sent out, non‐respondents were sent a reminder in the same mode as the designated invitation.
Notes	Questionnaire not described One group was contacted by SMS, the second one by email, and the third subsample served as a control group, receiving no pre‐notice. Students were then invited to participate by email or by SMS.

**Table:** Brusseleers 2019

Methods	Not yet assessed
Data	Not yet assessed
Comparisons	Not yet assessed
Outcomes	Not yet assessed
Notes

**Table:** Burns 2015

Methods	Not yet assessed
Data	Not yet assessed
Comparisons	Not yet assessed
Outcomes	Not yet assessed
Notes

**Table:** Damberg 2020

Methods	Not yet assessed
Data	Not yet assessed
Comparisons	Not yet assessed
Outcomes	Not yet assessed
Notes

**Table:** Davidson 2019

Methods	Not yet assessed
Data	Not yet assessed
Comparisons	Not yet assessed
Outcomes	Not yet assessed
Notes

**Table:** Dommeyer 2008

Methods	Not yet assessed
Data	Not yet assessed
Comparisons	Not yet assessed
Outcomes	Not yet assessed
Notes

**Table:** Dudas 2012

Methods	Not yet assessed
Data	Not yet assessed
Comparisons	Not yet assessed
Outcomes	Not yet assessed
Notes

**Table:** Duncan 2015

Methods	Not yet assessed
Data	Not yet assessed
Comparisons	Not yet assessed
Outcomes	Not yet assessed
Notes

**Table:** Duncan 2017

Methods	Not yet assessed
Data	Not yet assessed
Comparisons	Not yet assessed
Outcomes	Not yet assessed
Notes

**Table:** Edelman 2013a

Methods	Not yet assessed
Data	Not yet assessed
Comparisons	Not yet assessed
Outcomes	Not yet assessed
Notes

**Table:** Epperson 1997

Methods	Not yet assessed
Data	Not yet assessed
Comparisons	Not yet assessed
Outcomes	Not yet assessed
Notes	Not yet assessed

**Table:** Finkelstein 2016

Methods	Not yet assessed
Data	Not yet assessed
Comparisons	Not yet assessed
Outcomes	Not yet assessed
Notes

**Table:** Goodman 2017

Methods	Not yet assessed
Data	Not yet assessed
Comparisons	Not yet assessed
Outcomes	Not yet assessed
Notes

**Table:** Griffin 2019

Methods	Not yet assessed
Data	Not yet assessed
Comparisons	Not yet assessed
Outcomes	Not yet assessed
Notes

**Table:** Ho‐A‐Yun 2007

Methods	Not yet assessed
Data	Not yet assessed
Comparisons	Not yet assessed
Outcomes	Not yet assessed
Notes

**Table:** Hopkins 1983

Methods	Not yet assessed
Data	Not yet assessed
Comparisons	Not yet assessed
Outcomes	Not yet assessed
Notes

**Table:** ISRCTN16642368 2016

Methods	Not yet assessed
Data	Not yet assessed
Comparisons	Not yet assessed
Outcomes	Not yet assessed
Notes

**Table:** ISRCTN31304930 2014

Methods	Not yet assessed
Data	Not yet assessed
Comparisons	Not yet assessed
Outcomes	Not yet assessed
Notes

**Table:** ISRCTN99859966 2018

Methods	Not yet assessed
Data	Not yet assessed
Comparisons	Not yet assessed
Outcomes	Not yet assessed
Notes

**Table:** Joinson 2007

Methods	Not yet assessed
Data	Not yet assessed
Comparisons	Not yet assessed
Outcomes	Not yet assessed
Notes

**Table:** Kaplowitz 2011

Methods	Not yet assessed
Data	Not yet assessed
Comparisons	Not yet assessed
Outcomes	Not yet assessed
Notes

**Table:** Kelly 2010

Methods	Not yet assessed
Data	Not yet assessed
Comparisons	Not yet assessed
Outcomes	Not yet assessed
Notes

**Table:** Koitsalu 2018

Methods	Not yet assessed
Data	Not yet assessed
Comparisons	Not yet assessed
Outcomes	Not yet assessed
Notes

**Table:** Kozak 2020

Methods	Not yet assessed
Data	Not yet assessed
Comparisons	Not yet assessed
Outcomes	Not yet assessed
Notes	Not yet assessed

**Table:** Langeland 2019

Methods	Not yet assessed
Data	Not yet assessed
Comparisons	Not yet assessed
Outcomes	Not yet assessed
Notes

**Table:** Maynard 1996

Methods	Not yet assessed
Data	Not yet assessed
Comparisons	Not yet assessed
Outcomes	Not yet assessed
Notes	Not yet assessed

**Table:** McCrohan 1981

Methods	Not yet assessed
Data	Not yet assessed
Comparisons	Not yet assessed
Outcomes	Not yet assessed
Notes

**Table:** Mendoza 2014

Methods	Not yet assessed
Data	Not yet assessed
Comparisons	Not yet assessed
Outcomes	Not yet assessed
Notes

**Table:** Muñoz‐Leiva 2010

Methods	Random allocation: method unspecified
Data	1654 students of Business Management in Department of Marketing and Market Research, Faculty of Business and Economics, University of Granada, Spain
Comparisons	Personalised emails vs. standard More frequent (every 10 days) reminders vs. less frequent (every 20 days) reminders
Outcomes	Response after 2 months (5 reminders)
Notes	Over 44 items. Initial invitation email, 5 reminders

**Table:** Newton 1998

Methods	Not yet assessed
Data	Not yet assessed
Comparisons	Not yet assessed
Outcomes	Not yet assessed
Notes

**Table:** O'Keefe 1987

Methods	Not yet assessed
Data	Not yet assessed
Comparisons	Not yet assessed
Outcomes	Not yet assessed
Notes

**Table:** Parast 2018

Methods	Not yet assessed
Data	Not yet assessed
Comparisons	Not yet assessed
Outcomes	Not yet assessed
Notes

**Table:** Patrick 2021

Methods	Not yet assessed
Data	Not yet assessed
Comparisons	Not yet assessed
Outcomes	Not yet assessed
Notes

**Table:** Porter 2007

Methods	Not yet assessed
Data	Not yet assessed
Comparisons	Not yet assessed
Outcomes	Not yet assessed
Notes

**Table:** Price 2004a

Methods	Not yet assessed
Data	Not yet assessed
Comparisons	Not yet assessed
Outcomes	Not yet assessed
Notes

**Table:** Price 2004b

Methods	Not yet assessed
Data	Not yet assessed
Comparisons	Not yet assessed
Outcomes	Not yet assessed
Notes

**Table:** Price 2004c

Methods	Not yet assessed
Data	Not yet assessed
Comparisons	Not yet assessed
Outcomes	Not yet assessed
Notes

**Table:** Price 2006

Methods	Not yet assessed
Data	Not yet assessed
Comparisons	Not yet assessed
Outcomes	Not yet assessed
Notes

**Table:** Rach 1994

Methods	Not yet assessed
Data	Not yet assessed
Comparisons	Not yet assessed
Outcomes	Not yet assessed
Notes

**Table:** Samel‐Kowalik 2012

Methods	Not yet assessed
Data	Not yet assessed
Comparisons	Not yet assessed
Outcomes	Not yet assessed
Notes

**Table:** Siera 1988

Methods	Not yet assessed
Data	Not yet assessed
Comparisons	Not yet assessed
Outcomes	Not yet assessed
Notes

**Table:** Strickland 1980

Methods	Not yet assessed
Data	Not yet assessed
Comparisons	Not yet assessed
Outcomes	Not yet assessed
Notes

**Table:** Treat 1996

Methods	Not yet assessed
Data	Not yet assessed
Comparisons	Not yet assessed
Outcomes	Not yet assessed
Notes

**Table:** Virtanen 2007

Methods	Not yet assessed
Data	Not yet assessed
Comparisons	Not yet assessed
Outcomes	Not yet assessed
Notes

**Table:** Wood 2015

Methods	Not yet assessed
Data	Not yet assessed
Comparisons	Not yet assessed
Outcomes	Not yet assessed
Notes	

SAS: Statistical Analysis System SMS: Short message service

## Differences between protocol and review

There are no differences between the protocol and the review.
